# International Congress of Drug Therapy in HIV Infection 23-26 October 2016, Glasgow, UK

**DOI:** 10.7448/IAS.19.8.21487

**Published:** 2016-10-23

**Abstract O114–Figure 1:**
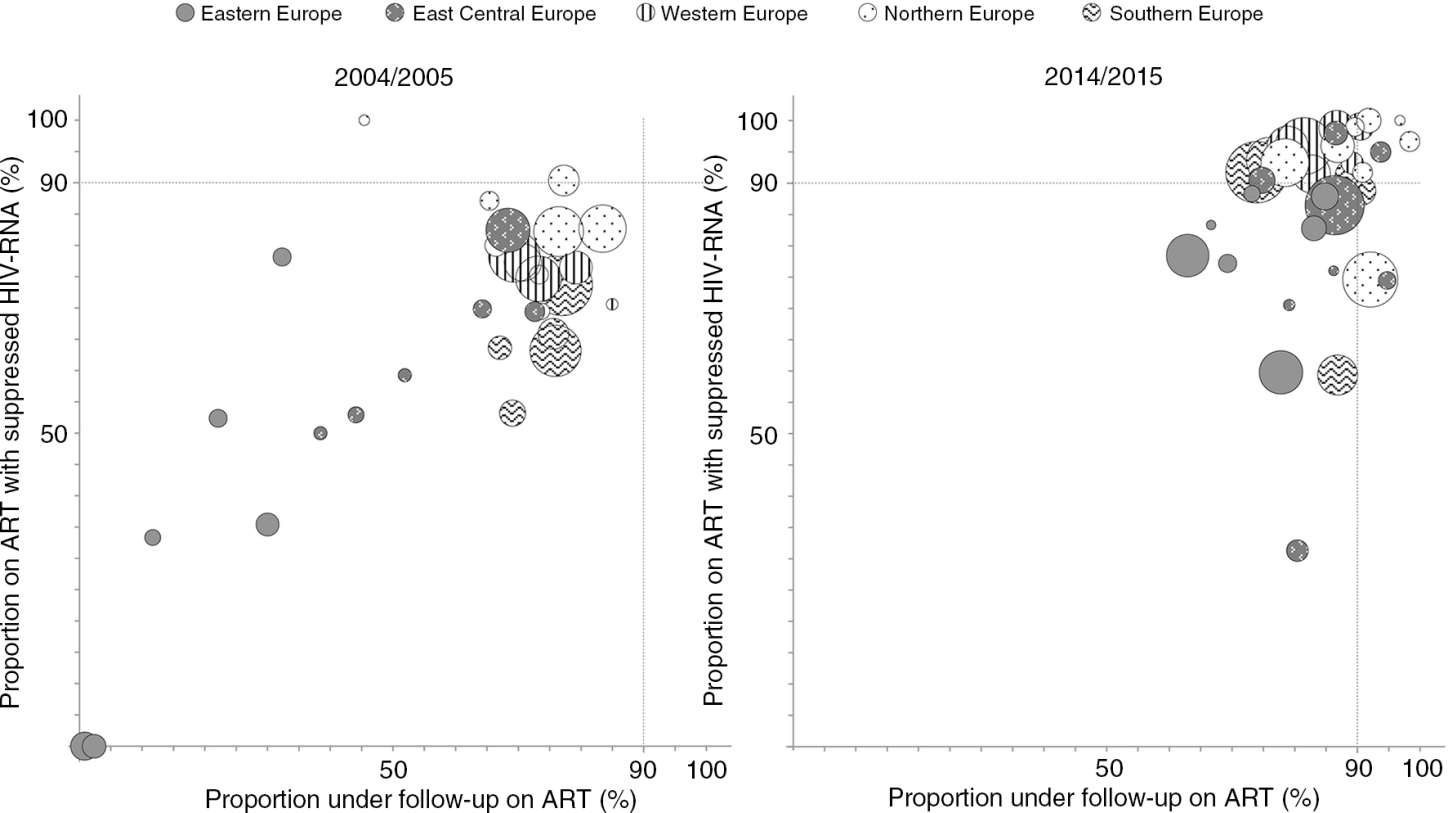
Unadjusted estimates of ART coverage and proportion with ART-induced HIV RNA suppression by EuroSIDA country and region in two different time periods. Each bubble represents a country. The area of the bubble is proportional to the number of people under follow-up in each country. The two dotted lines indicate >90% ART coverage (x-axis) and >90% ART-induced HIV RNA suppression (y-axis). **Eastern Europe:** Belarus, Estonia, Georgia*, Latvia, Lithuania, Russia, Ukraine. **East Central Europe:** Bosnia-Herzegovina*, Croatia*, Czech Republic, Hungary, Poland, Romania, Serbia, Slovakia†, Slovenia*. **Western Europe:** Austria, Belgium, France, Germany, Luxembourg, Switzerland. **Southern Europe:** Argentina, Greece, Israel, Italy, Portugal, Spain. **Northern Europe:** Denmark, Finland, Iceland*, Ireland, Netherlands, Norway, Sweden, UK. *included only in 2014/15 cohort; †included only in 2004/05 cohort.
