# Supplementary material for: International Congress of Drug Therapy in HIV Infection 23-26 October 2016, Glasgow, UK
Source: J Int AIDS Soc. 2016 Oct 23;19(8Suppl 7):21487. doi: 10.7448/IAS.19.8.21487 (PMC5080528; doi:10.7448/IAS.19.8.21487)
Supplement: Related texts

# Sub article: KL1 Ending the HIV/AIDS pandemic: follow the science

**Published:** 2016-10-23

**Authors:** Anthony Fauci

**Affiliations:** National Institute of Allergy and Infectious Diseases (NIAID)//National Institutes of Health (NIH), Bethesda, MD, USA

## Abstract

Scientific advances over the 35 years since AIDS was first recognized as a new disease, have put us on a clear path towards ending the HIV/AIDS pandemic. Scaling-up access to antiretroviral therapy (ART) and HIV prevention strategies, such as pre-exposure prophylaxis, could dramatically decrease HIV-related deaths and the rate of new HIV infections. Current and future scientific advances, notably in HIV vaccine and cure research, will accelerate this process. Two major directions in HIV vaccine development will be discussed: building on the results from RV 144, the clinical trial in Thailand that resulted in the first modest signal of efficacy for a HIV vaccine; and structure-based immunogen design to elicit broadly neutralizing antibodies. Cure research has accelerated greatly over the past few years in two areas. The first is the prospect of eradicating the HIV reservoir altogether (i.e. a classic cure), which might involve novel latency-reversing and immunotoxic regimens and gene editing techniques to create a host cellular environment that does not allow HIV replication. The second approach involves controlling viral rebound following discontinuation of ART to achieve sustained virological remission employing strategies, such as passive transfer of broadly neutralizing antibodies and therapeutic vaccination. In 2016, the arsenal of scientifically proven interventions available, as well as the hope of others to come, offer unprecedented opportunities to make major gains in the fight against HIV/AIDS. With a major global commitment to implement these scientific advances, the end of the HIV/AIDS pandemic is now achievable.

# Sub article: KL2 Treatment for cancer, HIV and viral hepatitis in Europe using low-cost generic drugs: what could be done?

**Published:** 2016-10-23

**Authors:** Andrew Hill

**Affiliations:** St Stephens AIDS Trust, Chelsea and Westminster Hospital, London, UK

## Abstract

Across Europe, high drug prices can limit access to treatment for hepatitis C, cancer and pre-exposure prophylaxis for HIV. Fifteen years ago, it was shown that antiretrovirals for HIV/AIDS could be mass produced at very low costs. This led to treatment programmes which now supply drugs to more than 17 million people with HIV worldwide. Similar analyses of drug production show that viral hepatitis, tuberculosis and certain cancers could also be treated at very low costs. Several key drugs will become generic in Europe within the next 5 years. There is a potential to expand treatment coverage for key diseases, while lowering overall costs of treatment. For mass treatment with low-cost generic drugs to be successful, five key conditions need to be met:

1. When any drug becomes generic, it should become available to publicly run health services at prices close to the cost of production, with an acceptable profit margin. These prices are freely available from India.

2. Pharmaceutical companies should not be able to inflate the prices of drugs after initial approval.

3. When a drug becomes generic and a low price is established, the effects of this lower price on the value of other drugs should be evaluated. Higher prices for newer drugs may no longer be justified.

4. Any secondary patent on a drug should be carefully evaluated for validity.

5. Pharmaceutical companies involved in bribery, false advertising or suppression of clinical trial results should pay significant fines, which are then used to sponsor national treatment access schemes.

# Sub article: KL3 Revolution in prevention in low- and middle-income settings

**Published:** 2016-10-23

**Authors:** Linda-Gail Bekker

**Affiliations:** The Desmond Tutu HIV Centre, University of Cape Town, Cape Town, South Africa

## Abstract

After 2000, we saw a remarkable era of HIV treatment roll out with consequent notable public health gains. This will be remembered as a treatment revolution. Most recently, with a number of important human trials marking at least partial efficacy with male circumcision, topical and systemic antiretroviral-based prophylaxis, HIV vaccines and other promising primary prevention modalities in the pipeline, this next decade could well be thought of as the prevention revolution. How the prevention revolution plays out in resource-constrained settings will depend on political will, resources and the competing need to reach the other half of the treatment pool effectively.

# Sub article: O111 HIV treatment as prevention, from a research hypothesis to a new global target and beyond

**Published:** 2016-10-23

**Authors:** Julio Montaner

**Affiliations:** British Columbia Centre for Excellence in HIV/AIDS, and University of British Columbia, Vancouver, Canada

## Abstract

Antiretroviral therapy (ART) has dramatically reduced progression to AIDS and premature death among people living with HIV (PLHIV). Furthermore, ART is highly effective in preventing HIV transmission. We refer to this combined effect of ART as treatment as prevention (TasP). HIV TasP has proven cost-effective, because beyond its impact on morbidity and mortality, TasP decreases HIV incidence, which acts as a multiplier on the return-on-investment. In 2014, under the Joint United Nations Programme on HIV/AIDS leadership, we developed the 90–90–90 target, a new TasP-inspired ambitious goal for global HIV treatment to “End the AIDS Pandemic” as a public health threat by 2030. The 90–90–90 target, proposes by 2020, ≥90% of all PLHIV will know their HIV status; ≥90% of them will have access to ART; and ≥90% of them will achieve sustained HIV viral suppression. The success of HIV-TasP has fuelled enthusiasm that this approach could be successfully exported and adapted to other infectious diseases, such as hepatitis C infection. Similarly, there is growing interest regarding a possible role TasP may play dealing with conditions where there is “social contagion” (i.e. any condition where increased prevalence is associated with increased incidence through behavioural contagion; including smoking, addiction or obesity-related diseases). We believe that TasP offers a unique means to optimize the management of selected high burden conditions, with a view to reduce morbidity and mortality, as well as prevalence and incidence within a highly cost-effective framework, and as such, to promote healthcare sustainability.

# Sub article: O112 Initiation of ART early in HIV infection: START to Finish

**Published:** 2016-10-23

**Authors:** Jens Lundgren

**Affiliations:** Initiation of CHIP and PERSIMUNE, Rigshospitalet, University of Copenhagen, Copenhagen, Denmark

## Abstract

The strategic question on when to initiate antiretroviral therapy (ART) was finally resolved in 2015: since all HIV-positive persons stand to benefit from ART, this treatment should be offered to all. The START study provided key evidence by demonstrating a substantial reduction in risk of disease progression by early versus deferred initiation of ART in early HIV infection (i.e. before the CD4 cell count had decreased below 500 cells/µL). A series of sub studies and secondary analysis from START has subsequently been reported. The key findings from this portfolio of research will be reviewed and will include the identification of key subgroups with varying absolute risk reduction from the early use of ART, immunological correlates of ART-induced clinical protection, ART-induced depletion of bone mineral density, the lack of benefit on arterial elasticity, pulmonary and neurocognitive function, beneficial effects from ART on opportunistic disease, invasive bacterial infections, cancer, and kidney and bone marrow function. Overall, the data demonstrate that the balance of benefits versus risks from early ART favours the benefit across a wide spectrum of pathophysiological processes. In conclusion, global consensus on evidence for universal access to ART now exists; implementation research is key, as only half of the infected population is currently receiving ART.

# Sub article: O113 Transition to adult care

**Published:** 2016-10-23

**Authors:** Pablo Rojo

**Affiliations:** Paediatric Infectious Diseases, Hospital 12 de Octubre, Complutense University, Madrid, Spain

## Abstract

The presentation is given by a Spanish paediatrician, who will be directing his presentation mainly to European adult HIV physicians. This presentation will refer to the situation of HIV-infected adolescents and young adults, mainly perinatally infected, being transferred from paediatric HIV clinics to adult HIV clinics in Europe. The presentation will focus specifically on three issues: 1) the special pattern of adolescence and young adulthood in relation to neurocognitive development, behaviour and chronic illness; 2) to review what are the main clinical, immunovirological, psychological and social characteristics of the adolescents and young adults who are being transferred currently and in the near future to the adult HIV clinics. Special attention will be on the differences between the children born before and after combined antiretroviral treatment, which was available in the paediatric population; and 3) the system where they come from: the insights of a paediatric HIV clinic in Europe.

# Sub article: O114 Persistent disparities in meeting WHO/UNAIDS targets for ART coverage and ART-induced HIV RNA suppression across Europe

**Published:** 2016-10-23

**Authors:** Kamilla Grønborg Laut, Leah Shepherd, Roxana Radoi, Igor Karpov, Milosz Parczewski, Cristina Mussini, Fernando Maltez, Marcelo Losso, Nikoloz Chkhartishvili, Hila Elinav, Helen Kovari, Anders Blaxhult, Robert Zangerle, Tatiana Trofimora, Brygida Knysz, Kai Zilmer, Elena Kuzovatova, Therese Staub, Dorthe Raben, Jens Lundgren, Amanda Mocroft, Ole Kirk

**Affiliations:** 1 CHIP-Centre for Health and Infectious Disease Research, Rigshospitalet, University of Copenhagen, København, Denmark; 2 Department of Infectious Diseases, Rigshospitalet, University of Copenhagen, København, Denmark; 3 Department of Infection and Population Health, University College London, London, UK; 4 Spitalul de Boli Infectioase si Tropical, Dr Victor Babes Hospital, Bucharest, Romania; 5 Department of Infectious Diseases, Belarus State Medical University, Minsk, Belarus; 6 Department of Infectious and Tropical Diseases, and Immune Deficiency, Pomeranian Medical University, Szczecin, Poland; 7 Department of Infectious and Tropical Diseases, University of Modena and Reggio Emilia, Modena, Italy; 8 Servico de Doencas Infecciosas, Hospital de Curry Cabral, Lisbon, Portugal; 9 Servicio de Immunocomprometidos, Hospital JM Ramos Mejia, Buenos Aires, Argentina; 10 Infectious Diseases, AIDS & Clinical Immunology Research Center, Tbilisi, Georgia; 11 Department of Clinical Microbiology and Infectious Diseases, Hadassah Hospital, Jerusalem, Israel; 12 Division of Infectious Diseases and Hospital Epidemiology, University Hospital Zürich, Zürich, Switzerland; 13 Department of Infectious Diseases, Södersjukhuset, Venhälsan, Stockholm, Sweden; 14 Department of Dermatology and Venerology, Medical University Innsbruck, Innsbruck, Austria; 15 Infectious Diseases, Novgorod Centre for AIDS Prevention and Control, Velikij Novgorod, Russian Federation; 16 Department of Infectious Diseases, Wroclaw Medical University, Wroclaw, Poland; 17 Centre of Infectious Diseases, West-Tallin Central Hospital, Tallin, Estonia; 18 Nizhny Novgorod Scientific and Research Institute of Epidemiology and Microbiology named after Academician IN Blokhina, Privolzhsky Fed Dist AIDS Control and Prevention C, Nizhny Novgorod, Russian Federation; 19 Service des Maladies Infectieuses, Centre Hospitalier de Luxembourg, Luxembourg City, Luxembourg

## Abstract

**Introduction**: Direct comparisons between countries in core HIV care parameters are often hampered by different data collection. We compared temporal changes in country-specific rates of the UNAIDS/WHO targets of >90% ART coverage and >90% ART-induced HIV RNA suppression for a given population.

**Materials and methods**: EuroSIDA participants under follow-up between the periods 1 January 2004 to 31 December 2005 and 1 January 2014 to 31 December 2015 were followed from first visit until latest of CD4, HIV RNA or follow-up visit. Based on the included EuroSIDA centres, country-specific proportions of persons on ART (≥3 antiretrovirals) and HIV RNA suppression (<500 copies/mL) among patients on ART were assessed. Missing HIV RNA was considered as unsuppressed. Temporal changes were analyzed using generalized estimating equations, accounting for repeated measurements and adjusting for age, gender, mode of infection, CD4 at first visit, HBV and HCV status.

**Results**: A total of 11,975 people were under follow-up in the 2014/15 cohort (n=8978 in 2004/05), in 105 clinics in Eastern Europe (EE) (n=1748), East Central (EC) Europe (n=1884), Western Europe (WE) (n=2512), Southern Europe (SE) (n=3109) and Northern Europe (NE) (n=2722). Overall ART coverage within EuroSIDA increased from 68.0% in 2004/05 to 82.4% in 2014/15 [adjusted odds ratio (aOR) of being on ART in 2014/15 versus 2004/05: 1.90 (95% CI 1.77–2.03)], and among those on ART, the proportion with suppressed HIV RNA increased from 74.5 to 86.9% [aOR 2.09 (1.91–2.27)]. Overall odds of being on ART and virologically suppressed doubled from 2004/05 to 2014/15 [aOR 2.13 (2.00–2.26)]. Improvements in ART coverage and HIV RNA suppression varied significantly across regions (p<0.001) and were greatest in EE where ART coverage and HIV RNA suppression was low in 2004/05: in EE, aOR of being on ART and virologically suppressed in 2014/15 versus 2004/05 was 15.78 (11.75–21.19), compared with EC aOR 2.39 (2.04–2.80), WE aOR 2.69 (2.38–3.04), SE aOR 1.97 (1.77–2.19) and NE aOR 1.75 (1.53–1.99). In 2014/15, 6/35 (17%) countries had >90% ART coverage and >90% ART-induced HIV RNA suppression [0/7 (0%) EE, 1/8 (13%) EC, 1/6 (17%) WE, 4/8 (50%) NE and 0/6 (0%) SE countries]. However, the pattern differed significantly between participating clinics across countries, with country-specific proportions of ART coverage ranging from 63 to 98%, and viral suppression from 31 to 100% of those on ART (Figure 1).

**Conclusions**: Despite marked improvements over the last decade, we observed persistent large variation among countries in our cohort in meeting the UNAIDS/WHO targets for treatment coverage and virological suppression. The representativeness of clinics and patients, as well as underlying factors differentiating individual countries’ ability to meet the targets, are under investigation.

# Sub article: O121 Simplification to atazanavir/ritonavir+lamivudine versus maintaining atazanavir/ritonavir+2NRTIs in virologically suppressed HIV-infected patients: 96-week data of the ATLAS-M trial

**Published:** 2016-10-23

**Authors:** Roberta Gagliardini, Massimiliano Fabbiani, Eugenia Quiros Roldan, Alessandra Latini, Gabriella D'Ettorre, Andrea Antinori, Antonella Castagna, Giancarlo Orofino, Daniela Francisci, Pierangelo Chinello, Giordano Madeddu, Pierfrancesco Grima, Stefano Rusconi, Barbara Del Pin, Annalisa Mondi, Alberto Borghetti, Emanuele Focà, Manuela Colafigli, Roberto Cauda, Simona Di Giambenedetto, Andrea De Luca

**Affiliations:** 1 Institute of Clinical Infectious Diseases, Catholic University of Sacred Heart, Rome, Italy; 2 Division of Infectious and Tropical Diseases, University of Brescia, Brescia, Italy; 3 Infectious Dermatology and Allergology, IFO San Gallicano Institute (IRCCS), Rome, Italy; 4 Department of Infectious Diseases, La Sapienza University, Rome, Italy; 5 Department of Infectious Diseases, National Institute for Infectious Diseases Lazzaro Spallanzani IRCCS, Rome, Italy; 6 Department of Infectious and Tropical Diseases, San Raffaele Hospital, Vita-Salute San Raffaele University, Milan, Italy; 7 Infectious and Tropical Diseases Unit, Amedeo di Savoia Hospital, Torino, Italy; 8 Infectious Diseases Clinic, University of Perugia, Perugia, Italy; 9 Systemic Infections and Immunodeficiency Unit, National Institute for Infectious Diseases Lazzaro Spallanzani IRCCS, Rome, Italy; 10 Department of Clinical and Experimental Medicine, University of Sassari, Sassari, Italy; 11 Division of Infectious Diseases, S. Caterina Novella Hospital, Galatina, Italy; 12 Dipartimento di Scienze Biomediche e Cliniche, Luigi Sacco, Infectious Disease Unit, University of Milan, Milan, Italy; 13 Unit of Infectious Diseases, M. Annunziata Hospital, Florence, Italy; 14 Infectious Dermatology and Allergology Unit, IFO San Gallicano Institute (IRCCS), Rome, Italy; 15 Infectious Diseases Unit, Siena University Hospital, Siena, Italy

## Abstract

**Objectives**: To explore 96-week non-inferior efficacy of treatment simplification to atazanavir/ritonavir+lamivudine versus continuing atazanavir/ritonavir +2NRTI.

**Materials and methods**: ATLAS-M is a 96-week, multicentre, open-label, randomized study. Subjects on atazanavir/ritonavir+2NRTIs, without previous virologic failures, with HIV RNA <50 copies/mL for >3 months and CD4 >200 cells/mm^3^ for >6 months were eligible. At baseline, ' atazanavir/ritonavir+lamivudine (dual therapy, DT) or to continue the baseline regimen (triple therapy, TT). At 48 weeks, DT showed a higher proportion of patients free of treatment failure (primary study endpoint) when compared to TT, demonstrating superiority of DT strategy. Here we analyze the treatment failure, including virologic failure (two consecutive HIV RNA >50 copies/mL or a single value >1000 copies/mL), and other outcomes at 96 weeks.

**Results**: A total of 266 patients (78% males, median age 44 years, median CD4 603 cells/µL, 79% treated with tenofovir) were enrolled. Ninety-six-week data were available for 254 (126 in DT and 128 in TT). At baseline, subjects in the two arms did not differ for the main characteristics (Table 1). At 96 weeks, the proportion of patients free of TF were 77.8% (95% CI 70.5–85.1) in the DT and 65.6% (95% CI 57.4–73.8) in the TT arm (difference +12.2%, 95% CI +1.2, +23.2). VF was observed in two (1.6%) patients randomized to DT and eight (6.3%) to TT (p=0.056). Clinical adverse events occurred at similar rates in the two arms, mostly transient and not leading to treatment discontinuation. More frequent in the DT arm were new-onset grade 3 to 4 hypertriglyceridemia (7.6% vs. 1.6%, p=0.027) and hyperbilirubinemia (59.6% vs. 35.8%, p=0.001). No significant differences in CD4 changes from baseline at week 96 were observed between the two arms (mean +83 cells/µL in DT vs. +49 in TT, p=0.233). A greater increase in total cholesterol (+15 vs. +0 mg/dL, p=0.005) and HDL (+5 vs. +0 mg/dL, p=0.002) was observed in the DT arm without differences of other lipid parameters. Change from baseline estimated glomerular filtration rate was significantly better in the DT arm as compared to the TT arm (+5 vs. −3 mL/min/1.73 m^2^, p<0.001). No significant differences in other laboratory parameters were observed between the study arms.

**Conclusions**: This 96-week data demonstrated non-inferiority and even superior efficacy of treatment simplification to atazanavir/ritonavir+lamivudine as compared to continuation of atazanavir/ritonavir+2NRTIs in virologically suppressed patients. A numerically higher rate of VF was observed in the TT arm. Switch to DT was associated with improved renal function over TT continuation, but also with increased total cholesterol and bilirubin levels.

**Abstract O121–Table 1:** Baseline patients characteristics based on randomization arm (interim 96-week population n = 254)

	ATV/r + 3TC (DT arm) n = 126	ATV/r+2NRTIs (TT arm) n=128	p
Age, years^a^	43.4 (35.7–49.2)	44.2 (36.2–51.0)	0.963
Male gender	107 (84.9)	96 (75.0)	0.069
IDU (risk factor)	8 (6.3)	11 (8.6)	0.659
HCV co-infection	12 (9.5)	14 (10.9)	0.836
Previous AIDS events	17 (13.5)	11 (8.6)	0.961
Years from HIV diagnosis^a^	4.2 (2.1–8.6)	4.9 (2.5–10.4)	0.102
Years from first cART initiation^a^	2.7 (1.7–4.8)	2.7 (1.6–6.5)	0.207
Therapeutic line^a^	2 (1–3)	2 (1–3)	0.628
Months from last regimen initiation^a^	27.6 (17.9–52.5)	28.7 (16.1–52.0)	0.352
TDF-containing backbone	100 (79.4)	109 (85.2)	0.296
CD4 nadir, cells/µL^a^	277 (132–359)	257 (144–349)	0.848
CD4, cells/µL^a^	621 (466–777)	614 (485–784)	0.806
Months with viral load <50 copies/mL^a^	23.4 (12.6–46.7)	21.0 (12.5–44.9)	0.403

a Values are expressed as n (%) except for median (interquartile range, IQR). 3TC, lamivudine; ATV/r, atazanavir/ritonavir; HCV, hepatitis C virus; NRTI, nucleos(t)ide reverse transcriptase inhibitors; TDF, tenofovir.

# Sub article: O122 Dual therapy with a boosted protease inhibitor plus lamivudine is an effective maintenance strategy in patients on second-line antiretroviral therapy in Africa: the ANRS 12286/MOBIDIP trial

**Published:** 2016-10-23

**Authors:** Laura Ciaffi, Sinata Koulla-Shiro, Adrien Sawadogo, N Fatou Ngom Gueye, Vincent Le Moing, Sabrina Eymard-Duvernay, Suzanne Izard, Jacques Zoungrana, Pretty Mbouyap, Mamadou Diallo, Guillaume Bado, Koumba Toure Kane, Avelin Aghokeng, Martine Peeters, Jacques Reynes, Eric Delaporte

**Affiliations:** 1 IRD INSERM, UMI 233, Montpellier, France; 2 Faculty of Medicine and Biomedical Sciences, University of Yaounde 1, Yaounde, Cameroon; 3 Hôpital de Jour, Centre Hospitalier Universitaire de Souro Sanou, Bobo Dioulasso, Burkina Faso; 4 Hôpital de Jour, Centre Hospitalier Universitaire de Fann, Dakar, Senegal; 5 Projet MOBIDIP, Agence Nationale de Recherche sur le SIDA et les hépatites virales (Cameroun Site), Yaounde, Cameroon; 6 Centre Hospitalier Universitaire de Fann, CRCF, Dakar, Senegal; 7 Laboratoire de Bactériologie-Virologie, Centre Hospitalier Universitaire Aristide Le Dantec, Dakar, Senegal; 8 Virology laboratory IMPM-IRD, Centre de Recherche sur les Maladies Emergentes et, Yaounde, Cameroon; 9 Maladies Infectieuses, Centre Hospitalier Universitaire Montpellier, Montpellier, France

## Abstract

**Introduction**: Second-line ART regimens with ritonavir-boosted protease inhibitor (PI/r) plus nucleoside reverse-transcriptase inhibitors (NRTIs) have shown good efficacy in resource-limited settings [1–3]. But issues of costs, toxicity and future options make a simplified maintenance treatment a strategy of interest. We aimed to compare two maintenance treatments with PI/r in mono- or dual therapy [plus lamivudine (3TC)] in a group of virally suppressed patients on second-line ART.

**Material and methods**: A randomized, open-label, multicentre clinical trial was conducted in Cameroon, Senegal and Burkina Faso. HIV-1 positive patients followed in the ANRS 12186 2LADY trial (3) on stable PI plus NRTIs second-line ART with HIV-1 RNA [viral load (VL)] below 200 copies/mL, CD4 above 100 cells/mm^3^ and adherence ≥90%, were included in a two arms trial comparing monotherapy with the ongoing PI/r: darunavir (DRV/r) or lopinavir (LPV/r) – mono arm – with the same PI/r associated with 3TC 300 mg – dual arm. The primary outcome was failure rate at 96 weeks. Treatment failure was defined as 1) a confirmed VL above 500 copies/mL, 2) reintroduction of the NRTI backbone or 3) the interruption of PI.

**Results**: From March 2014 to January 2015, 265 patients were randomized (133 in mono arm and 132 in dual arm). Included patients were mainly women (73%), with a median age of 42 years [interquartile range (IQR) 36–50]; median CD4 was 475 cells/mm^3^ (IQR 379–652) and median time on second line was 37 months (IQR 30–47). At the failure of first line, 96% had the M184V mutation. For the Data Safety Board meeting in March 2016, week 48 data were analyzed. The Board advised for the interruption of the mono arm. In the ITT analysis, 3.0% (95% CI 0.8–7.6) and 22.6% (95% CI 15.8–30.6) of patients failed in the dual and mono arm respectively (p<0.001). Median time to failure was 24 weeks. All failing patients, except one, resuppressed to less than 200 copies/mL in a median time of 12 weeks after reintroduction of the NRTI backbone. Increase in CD4 was significantly higher in the dual arm (48 vs. 7 cells/mm^3^). No differences in adverse events were observed. Neither adherence, nor nadir CD4 count, nor PI drug were associated with failure.

**Conclusions**: After viral suppression with PI plus NRTIs in second-line therapy, maintenance with PI/r plus 3TC is associated with a high rate of success despite the presence of M184V while PI/r monotherapy cannot be recommended.

**References:**

1. SECOND-LINE Study Group, Boyd MA, Kumarasamy N, Moore CL, Nwizu C, Losso MH, et al. Ritonavir-boosted lopinavir plus nucleoside or nucleotide reverse transcriptase inhibitors versus ritonavir-boosted lopinavir plus raltegravir for treatment of HIV-1 infection in adults with virological failure of a standard first-line ART regimen (SECOND-LINE): a randomised, open-label, non-inferiority study. Lancet. 2013;381:2091–9. doi: http://dx.doi.org/10.1016/S0140-6736(13)61164-2

2. Paton N, Kityo C, Hoppe A, Reid A, Kambugu A, Lugemwa A, et al. Assessment of second-line antiretroviral regimens for HIV therapy in Africa. N Engl J Med. 2014;371:234–47. doi: http://dx.doi.org/10.1056/NEJMoa1311274

3. Ciaffi L, Koulla-Shiro S, Sawadogo A, le Moing V, Eymard-Duvernay S, Izard S, et al. Efficacy and safety of three second-line antiretroviral regimens in HIV-infected patients in Africa: the ANRS 12169/2LADY randomised trial. AIDS. 2015;29:1473–81. doi: http://dx.doi.org/10.1097/QAD.0000000000000709

# Sub article: O123 Resistance profile analysis of treatment-experienced HIV-1-infected patients switching to elvitegravir/cobicistat/emtricitabine/tenofovir alafenamide (E/C/F/TAF) plus darunavir (DRV)

**Published:** 2016-10-23

**Authors:** Nicolas Margot, Renee Ram, Moupali Das, Marshall Fordyce, Scott McCallister, Michael Miller, Christian Callebaut

**Affiliations:** 1 Clinical Virology, Gilead, Foster City, CA, USA; 2 Clinical Research, Gilead, Foster City, CA, USA

## Abstract

**Introduction**: In study GS-US-292-0119, virologically suppressed, treatment-experienced patients on complex multi-tablet regimens (1) were switched to a simpler, more convenient antiretroviral regimen. After 48 weeks, viral suppression was maintained in 94.4% of patients who switched to E/C/F/TAF+DRV compared to 76.1% in the DRV-containing “Stay on Baseline Regimen” arm. All patients had documented resistance to >2 classes of antiretroviral (ARV) agents at baseline. Detailed ARV regimens and the resistance profile of the study population are described.

**Methods**: Historical genotypic reports were analyzed for resistance-associated mutations (RAMs) to ARVs. The Stanford HIVdb algorithm version 8.01 was used to calculate genotypic susceptibility scores (GSS). For each drug, a 5-point scale was used: susceptible, potential low-level resistance, low-level resistance, intermediate-level resistance and high-level resistance were scored as 1, 0.75, 0.5, 0.25 and 0, respectively. The total GSS for a given regimen was calculated as the sum of the scores for each individual drug.

**Results**: A total of 94.8% had documented resistance to >2 classes of ARVs, including protease inhibitors (PIs; 34.8%), non-nucleoside RT inhibitors (NNRTIs; 88.1%) and NRTIs (94.8%). The most common PI-RAMs were L90M (15.6%) and V82A/F/L/S/T (14.8%), the most common NNRTI-RAMs were K103N/S (63%) and Y181C/I/V (19.3%) and the most common NRTI-RAMs were M184V/I (83%) and K65R (23.7%). Thymidine analog mutations (TAMs) were present in 42.2% of patients (59.6% with one or two TAMs and 40.4% with three TAMs). The distribution of GSS at study entry was similar across treatment groups. Patients in the E/C/F/TAF+DRV arm maintained virologic suppression similarly, regardless of the DRV dosage received before switching (33/33 and 51/56 with treatment success in the 600 mg BID and 800 mg QD groups, respectively). In the E/C/F/TAF+DRV arm, 11/89 patients (12.4%) had GSS <2, 51/89 patients (57.3%) had GSS ≥2 and <3, and 27/89 patients (30.3%) had GSS ≥3. Within each treatment group, patients maintained virologic suppression similarly regardless of their GSS at study entry.

**Conclusions**: Despite the high incidence of pre-existing resistance in this population, including resistance to ≥2 classes of ARV agents and presence of K65R or ≤3 TAMs, strategic simplification to E/C/F/TAF+DRV was statistically superior to staying on the baseline regimen. Patients benefited from switching regimen regardless of their prior DRV dose and their GSS. Treatment with E/C/F/TAF+DRV offers a simpler and more convenient option for treatment-experienced patients on complex multi-tablet regimens.

**Reference:**

1. Huhn G, Tebas P, Gallant J, Wilkin T, Cheng A, Yan M, et al. Strategic simplification: the efficacy and safety of switching to Elvitegravir/Cobicistat/Emtricitabine/Tenofovir Alafenamide (E/C/F/TAF) Plus Darunavir (DRV) in treatment-experienced HIV-1-infected adults [Oral Presentation #726]. IDWeek™, Oct 7–11 2015, San Diego, CA.

# Sub article: O124 Switching from rilpivirine/emtricitabine/tenofovir disoproxil fumarate (RPV/FTC/TDF) to rilpivirine/emtricitabine/tenofovir alafenamide (RPV/FTC/TAF): safety and efficacy through 48 weeks

**Published:** 2016-10-23

**Authors:** Chloe Orkin, Edwin DeJesus, Moti Ramgopal, Gordon Crofoot, Peter Ruane, Anthony LaMarca, Anthony Mills, Bernard Van der Cam, Joseph De Wet, Jurgen Rockstroh, Adriano Lazzarin, Bart Rijnders, Daniel Podzamczer, Anders Thalme, Marcel Stoeckle, Danielle Porter, Hui Liu, Andrew Cheng, Erin Quirk, Devi SenGupta, Huyen Cao

**Affiliations:** 1 Infection and Immunity, Royal London Hospital, Barts Health NHS Trust, London, UK; 2 Private Practice, Orlando Immunology Center, Orlando, FL, USA; 3 Research Facility, Midway Immunology and Research Center, Fort Pierce, FL, USA; 4 Research Facility, The Crofoot Research Center, Houston, TX, USA; 5 Private Practice, Peter Jerome Ruane, Los Angeles, CA, USA; 6 Research Facility, Therafirst Medical Center, Fort Lauderdale, FL, USA; 7 Private Practice & Research Facility, Mills Clinical Research, Los Angeles, CA, USA; 8 Department of Internal Medicine, Cliniques Universitaires St Luc, Bruxelles, Belgium; 9 Private Practice, Dr Joseph J de Wet, Vancouver, Canada; 10 Department of Medicine, Bonn University Hospital, Bonn, Germany; 11 Dipartimento di Malettie Infettive, Ospedale San Raffaele, Milan, Italy; 12 Deptartment of Internal Medicine, Erasmus MC, Rotterdam, Netherlands; 13 AIDS Unit in Infectious Diseases Service, Hospital Universitari de Bellvitge, Barcelona, Spain; 14 Division of Infectious Diseases, Department of Medicine, Karolinska University Hospital, Stockholm, Sweden; 15 Division of Infectious Diseases and Hospital Epidemiology, University Hospital Basel, Basel, Switzerland; 16 Clinical Virology, Gilead Sciences, Inc., Foster City, CA, USA; 17 Biostatistics - HIV, Gilead Sciences, Inc., Foster City, CA, USA; 18 Clinical Research - HIV, Gilead Sciences, Inc., Foster City, CA, USA

## Abstract

**Introduction**: Tenofovir alafenamide (TAF) is a novel tenofovir (TFV) prodrug that achieves 91% lower plasma TFV levels than seen with TDF, reducing the risks of renal and bone toxicities. The impact of switching from TDF (300 mg) to TAF (25 mg), as a fixed-dose combination with RPV (25 mg) and FTC (200 mg), was evaluated in this first phase 3 clinical trial of RPV/FTC/TAF. Primary endpoint (week 48) results are presented.

**Materials and methods**: A randomized (1:1), double-blind, active-controlled, phase 3 study was conducted in virologically suppressed (HIV-1 RNA <50 copies/mL) and HIV-infected adults with estimated glomerular filtration rate (eGFR) >50 mL/min taking RPV/FTC/TDF for at least 6 months. Eligible study participants were randomized to switch to RPV/FTC/TAF or to continue RPV/FTC/TDF. Primary endpoint was virologic suppression (HIV-1 RNA <50 copies/mL) at week 48 by FDA snapshot algorithm with a pre-specified non-inferiority margin of 8%. Bone and renal safety, and tolerability endpoints were evaluated.

**Results**: A total of 630 patients were enrolled (RPV/FTC/TAF 316 vs. RPV/FTC/TDF 314): median age 45 years, 10% women and 19% black. Through week 48, switching to RPV/FTC/TAF was non-inferior to remaining on RPV/FTC/TDF (94% vs. 94%; exact difference: −0.3%; 95% CI −4.2% to +3.7%). General safety was similar between the arms with low rates of grade 3 to 4 adverse events (AEs). The rate of discontinuations due to AEs was 0.1% in both groups. Improvement in bone mineral density was observed in the RPV/FTC/TAF group compared to the RPV/FTC/TDF group, with higher mean changes from baseline: hip +1.04% versus −0.25% (p<0.001) and spine +1.61% versus +0.08% (p<0.001), respectively. Median eGFR increased +4.5 mL/min for RPV/FTC/TAF and +0.7 mL/min for RPV/FTC/TDF (p=0.002). Improvements in quantitative proteinuria, including tubular proteinuria, were seen in patients switching to RPV/FTC/TAF (p<0.001) (Table 1). No cases of Fanconi syndrome or proximal renal tubulopathy were reported.

**Conclusion**: Through 48 weeks, virologically suppressed patients switching to RPV/FTC/TAF maintained high rates of virologic suppression, with improved markers of bone and renal safety compared to those remaining on RPV/FTC/TDF.

**Table 1:** Changes in proteinuria at week 48

	PV/FTC/TAF	RPV/FTC/TDF	p
**Urine protein: creatinine ratio**			
Median baseline value (mg/g)	53.2	50.0	0.69
Median % changes at week 48	−18.8	+7.3	<0.001
**Urine albumin: creatinine ratio**			
Median baseline value (mg/g)	5.5	5.4	0.98
Median % changes at week 48	−7.8	+16.8	<0.001
**Urine retinol binding protein: creatinine ratio**			
Median baseline value (µg/g)	101.2	111.1	0.12
Median % changes at week 48	−18.0	+21.5	<0.001
**Urine beta-2-microglobulin: creatinine ratio**			
Median baseline value (µg/g)	111.6	116.1	0.72
Median % changes at week 48	−29.0	+12.0	<0.001

# Sub article: O125 Long-term (96-week) efficacy and safety after switching from tenofovir disoproxil fumarate (TDF) to tenofovir alafenamide (TAF) in HIV-infected, virologically suppressed adults

**Published:** 2016-10-23

**Authors:** Francois Raffi, Chloe Orkin, Amanda Clarke, Laurence Slama, Joel Gallant, Eric Daar, Mingjin Yan, Michael E Abram, Sandra Friborg, Andrew Cheng, Martin Rhee

**Affiliations:** 1 C.H.U. de Nantes, Infectious & Tropical Diseases, Nates, France; 2 Barts Health NHS Trust, Infection and Immunity, London, UK; 3 Brighton & Sussex University Hospitals NHS Trust, HIV Unit, Brighton, UK; 4 Hôpital Tenon, Infectious and Tropical Diseases, Paris, France; 5 Southwest Care Center, Specialty Services, Santa Fe, NM, USA; 6 Los Angeles Biomedical Research Institute at Harbor-UCLA, Division of Adult Infectious Diseases, Torrance, CA, USA; 7 Gilead Sciences, Biostatistics - HIV, Foster City, CA, USA; 8 Gilead Sciences, Clinical Virology, Foster City, CA, USA; 9 Gilead Sciences, Clinical Operations - HIV, Foster City, CA, USA; 10 Gilead Sciences, Clinical Research - HIV, Foster City, CA, USA

## Abstract

**Introduction**: Recent HIV treatment guidelines have either replaced TDF with TAF or included both as part of recommended initial regimens. This study assesses long-term efficacy, safety and tolerability of switching emtricitabine FTC/TDF to FTC/TAF, each with various third agents, through Week (W) 96.

**Methods**: In this double-blind, active-controlled study, virologically suppressed HIV-infected participants receiving FTC/TDF-containing regimens were randomized (1:1) to switch to FTC/TAF versus continue FTC/TDF while remaining on the same third agent. Virologic suppression (HIV-1 RNA <50 c/mL) by FDA snapshot analysis, markers of bone and renal safety, and safety and tolerability were assessed up to W96.

**Results**: A total of 663 participants were randomized and treated (FTC/TAF N=333, FTC/TDF N=330): median age 49 years, 15% women, median estimated glomerular filtration rate (eGFR, Cockcroft Gault) 100 mL/min. Third agents included boosted protease inhibitors (46%), integrase inhibitors (28%) and non-nucleoside reverse transcriptase inhibitors (25%). Median duration of FTC/TDF use prior to enrolment was 5.1 years. Through W96, virologic suppression was maintained in 89% of participants in both groups [difference −0.5%; 95%CI (−5.3%, 4.4%)]. One FTC/TAF participant developed M184V in the first 48 weeks. Drug-related serious adverse events (AEs) were rare (FTC/TAF: 0 vs. FTC/TDF: 0.3%). Drug discontinuation due to AEs was low (FTC/TAF: 2.4% vs. FTC/TDF: 1.2%). No cases of Fanconi syndrome or proximal renal tubulopathy occurred with FTC/TAF; one FTC/TDF participant discontinued study drug due to proximal tubulopathy. Biomarkers of renal safety favoured FTC/TAF (Table 1). Lumbar spine and hip bone mineral density (BMD) increased in the FTC/TAF group, while decreasing in the FTC/TDF group (Table 1), with ≥3% improvement at W96: lumbar spine BMD 40% versus 18%, hip BMD 29% versus 11%, respectively. There were greater increases in lipids [including high-density lipoproteins (HDL)] with FTC/TAF versus FTC/TDF but no difference in total cholesterol to HDL ratio (Table 1) or initiation of lipid-lowering agents (FTC/TAF: 7% vs. FTC/TDF: 6%).

**Conclusion**: In virologically suppressed participants switching from FTC/TDF to FTC/TAF high rates of virologic suppression were maintained, while renal and bone safety parameters improved. These long-term data support FTC/TAF as a safe and durable backbone, which can be used in combination with various third agents for treatment of HIV-1 infection.

**Table 1:** Changes in renal, bone and lipid parameters from baseline to week 96

Parameters^a^^b^	FTC/TAF (N=333)	FTC/TDF (N=330)
eGFR, mL/min (Cockcroft Gault)	10.0	4.0
Urine protein: creatinine ratio, %	−26.0	2.7
Urine albumin: creatinine ratio, %	3.4	27.0
Urine beta-2-microglobulin: creatinine ratio, %	−29.7	46.8
Urine retinol binding protein: creatinine ratio, %	−4.1	42.6
Lumbar spine BMD, %	2.153	−0.167
Hip BMD, %	1.853	−0.331
Total cholesterol, mg/dL	14	1
LDL cholesterol, mg/dL	14	4
HDL cholesterol, mg/dL	1	−1
Triglycerides, mg/dL	11	2
Total cholesterol: HDL ratio	0.1	0.1

a Mean is used to summarize BMD; otherwise, median is used

b p-values were <0.05 for all, except total cholesterol to HDL ratio (p = 0.26).

# Sub article: O131 Confidentiality matters: innovative HIV testing

**Published:** 2016-10-23

**Authors:** Cheryl Johnson

**Affiliations:** World Health Organization, Geneva, Switzerland

## Abstract

The global scale-up of HIV testing services (HTS) has been tremendous; however, many of these tests never reach those with undiagnosed HIV and at high ongoing risk, for example, key populations, men and adolescents. Approximately 40% of people with HIV remain undiagnosed, and thus unable to receive life-saving treatment or effective prevention to stop onward transmission. To achieve the United Nation's 90–90–90 goals, greater efforts and innovations are needed, starting with the first 90 goal, which calls for the diagnosis of 90% of all people with HIV by 2020. Globally, 35% of new HIV infections are among key populations and their partners. Yet, HTS coverage and uptake among key populations remains poor and irregular worldwide. Men also remain unreached and untested, and evidence shows men present late in disease stage and have higher HIV-related mortality compared with women. Young people in high incidence settings, particularly sub-Saharan Africa, also remain untested and unlinked to prevention and treatment. It is well documented that among these populations, unfriendly services, fear of stigma and discrimination, and lack of privacy and confidentiality are barriers to HTS uptake. In many environments, this is further exacerbated by restrictive policies, such as age of consent laws and policies which criminalise key populations for their behaviour; deterring HTS uptake among those with greatest need. HTS approaches must evolve and utilize innovative methods that are effective, acceptable and meet the patient's need for confidentiality, such as HIV self-testing, anonymous and assisted HIV partner notification, and community- and facility-based models which take place in discreet locations, offer night-time hours, use trusted peers and lay providers, and are designed to be friendly and attractive to key populations, men and adolescents. Placing people at the highest risk of HIV at the centre of HIV testing programmes is essential, and this is the only way to reach and go beyond the first 90 goal.

# Sub article: O132 Convenience matters: catalogue STI testing and PrEP

**Published:** 2016-10-23

**Authors:** Patrick S Sullivan

**Affiliations:** Rollins School of Public Health, Emory University, Atlanta, USA

## Abstract

Mathematical modelling suggests that reducing HIV incidence among men who have sex with men (MSM) will require achieving high coverage of multiple HIV prevention interventions [e.g. HIV testing, sexually transmitted infections (STI) testing, condom promotion and pre-exposure prophylaxis (PrEP)]. For reasons of convenience and to minimise the burden on healthcare providers, we have developed systems to offer self-service options for HIV self-testing with telemedicine counselling, if requested; home specimen collection with mail-in processing of tests for HIV, urethral and rectal STIs; and at-home self-monitoring of behaviours and laboratory screens for MSM on PrEP. Acceptability has been high among MSM and their healthcare providers for these programmes. However, some challenges remain in the evaluation of programmes and in bringing programmes to a broader scale in the United States. Mail-out kits for STI testing and for PrEP monitoring offer important options to reach the highest risk MSM with a higher frequency of STI testing, and to lower the burden of follow-up PrEP care.

# Sub article: O133 Context matters: one-stop medical care from Eastern Europe to downtown London

**Published:** 2016-10-23

**Authors:** Jeffrey V Lazarus

**Affiliations:** CHIP, Centre for Health and Infectious Disease Research and WHO Collaborating Centre on HIV and Viral Hepatitis, Rigshospitalet, University of Copenhagen, Copenhagen, Denmark

## Abstract

Despite great advances in HIV medicine, people living with HIV (PLHIV) in many European settings are not attaining optimal health outcomes. This situation raises important questions about how well national and local health systems are meeting the full spectrum of PLHIV health needs. The public health community's increasing interest in health system performance in recent years presents important opportunities for researchers, policy-makers, community stakeholders and others to explore how PLHIV healthcare can be advanced in tandem with efforts to improve overall health system functioning. A key issue in this realm is the goal of making health systems more people-centred. As health system experts continue to explore what constitutes a “people-centred health system” in theory and in practice, the HIV field stands poised to make unique contributions to this emerging body of knowledge, which is greatly needed by policy-makers who seek to make health systems more cost-efficient and more equitable. Providing integrated “one-stop” medical care is one important aspect of people-centred health systems, but how can critical practices stemming from integrated care be transferred to HIV care and applied effectively in markedly different settings within and across countries? This presentation draws on the paradigm of people-centred health systems to provide strategic thinking into how to utilize local and national healthcare contexts to drive forward this next step in HIV care, which includes how to improve the quality of life of PLHIV.

# Sub article: O134 Choice matters: differentiated models of care

**Published:** 2016-10-23

**Authors:** Helen Bygrave

**Affiliations:** SAMU (Southern Africa Medical Unit), Médecins Sans Frontières, Cape Town, South Africa

## Abstract

Differentiated care is a patient-centred approach that simplifies and adapts HIV services across the cascade to reflect the preferences and expectations of various groups of people living with HIV, while reducing unnecessary burdens on the health system. By providing differentiated care, the health system can refocus resources to those most in need. Antiretroviral therapy (ART) delivery may be differentiated according to the medical needs of the patient, subpopulation and contextual factors. Using the “building blocks” of differentiated care, a model may be built to determine where, how often and to whom ART is provided to. Differentiated ART delivery for stable patients has demonstrated positive outcomes for both health systems and patients. In South Africa, HIV Adherence Clubs, where groups of 20 to 30 patients meet at either a facility or community location to receive their ART, have demonstrated higher rates of both retention (97% vs. 85%), virological uptake and suppression than those in conventional care. Community ART groups in Tete, Mozambique, where self-formed groups of patients on ART collect medication for each other, demonstrated retention within the model of 98%, 96%, 93% and 91% at 12, 24, 36 and 48 months, respectively. Such group models of ART delivery have also demonstrated an impact on reducing clinical visits, along with enhancing peer and community support. Moving forward, differentiated ART delivery must be adapted beyond stable patients and the principles applied across the HIV cascade. By adopting such patient-centred approaches, differentiated care will be a part of the solution to reach the United Nation's 90–90–90 target in the era of start all.

# Sub article: O211 Helping the HIV physician through the challenges of co-morbidities

**Published:** 2016-10-23

**Authors:** Edouard Battegay

**Affiliations:** Center of Competence Multimorbidity, University Hospital Zurich, Zurich, Switzerland

## Abstract

Between 20 and 30% of the population and about 90% of inpatients hospitalized in General Internal Medicine have multiple concurrent acute or chronic diseases, that is, multi-morbidity (MM). Complexity increases proportionally with the number of concurrent diseases, probably partially due to disease–disease interactions (DDIs). Risk factors for HIV, such as intravenous (IV) drug use and successful near-normalization of life expectancy in HIV, have increased the likelihood of other concurrent diseases to occur and to determine life expectancy. In one study, people living with HIV/AIDS had a prevalence of one or more co-morbidity of 29%, a rate similar to the population at large. Concurrent diseases associated with IV drug use include hepatitis and sometimes severe mental disorders. However, in ageing HIV patients especially, diseases constitute very typical MM clusters that include vascular risk factors and disease, heart and pulmonary disease, major mental disorders and a broad array of various other medical diseases and conditions. These occur sometimes in characteristic dyadic or triadic, or higher combinations (painful syndromes and depression; non-adherence and depression, and hypertension and HIV; pain treatment for arthritis and hypertension; non-adherence and mental disorders, etc.). Some of these conditions and combinations of interactions interact with HIV to worsen the prognosis. There are limited evidence-based guidelines for MM, be it without or with HIV, even for more prevalent forms of MM and frequent interacting combinations (mentioned earlier). This leaves MM care heavily reliant upon clinical guidelines intended for the treatment of single diseases. However, these guidelines do not adequately address the combined risk to multi-morbid patients and tend to ignore adverse DDI's (disease–disease, drug–disease and drug–drug interactions, due to multiple drug regimens, i.e. polypharmacy), especially if a condition is outside the usual realm of those specialists from the same field of expertise that wrote the guidelines. Decision-making concerning therapeutic conflicts due to adversely interacting treatments usually remains to be resolved at the discretion of involved clinicians. These conflicts typically demand prioritizing and reconciling adverse DDIs with the most suitable, best acceptable and sometimes surprising therapeutic strategy. They also require medical doctors to communicate these dilemmas and the corresponding lack of evidence-based security to patients in order to allow for shared decision-making, if possible and if wished. Furthermore, decision-making in dilemma situations can induce psychological stress upon patients, especially on conscientious medical doctors that they need to consciously deal with.

# Sub article: O212 HIV patients today and 10 years ago: do they have the same needs? Results from cross-sectional analysis of ANRS CO3 Aquitaine cohort

**Published:** 2016-10-23

**Authors:** Fabrice Bonnet, Fabien Le Marec, Olivier Leleux, Charles Cazanave, Estibaliz Lazaro, Pierre Duffau, Marie-Anne Vandenhende, Patrick Mercie, Didier Neau, François Dabis

**Affiliations:** 1 Service de Médecine Interne et Maladies Infectieus, Centre Hospitalier Universitaire de Bordeaux, Bordeaux, France; 2 INSERM U1219, ISPED, Université de Bordeaux, Bordeaux, France; 3 Service de Maladies Infectieuses et Tropicales, Centre Hospitalier Universitaire de Bordeaux, Bordeaux, France

## Abstract

**Introduction**: Nowadays, people living with HIV (PLHIV) live longer, due to highly effective ART. Also, due to age, risk factors exposure, ART and HIV-related factors, they are more likely to develop comorbidities, potentially requiring different, long-term healthcare management. This study aimed to describe the PLHIV characteristics, HIV markers, comorbidities and their risk factors and scores, in the same patients 10 years apart.

**Materials and methods**: The ANRS CO3 Aquitaine cohort prospectively collects epidemiological, clinical, biological and therapeutic data on PLHIV in the French Aquitaine region. Inclusion criterion for this analysis was ≥1 visit in both calendar years. Two cross-sectional analysis were performed (2004 and 2014), regarding patient characteristics, HIV markers, the prevalence of comorbidities [chronic kidney disease (CKD), fractures, cardiovascular disease (CVD), diabetes, dyslipidaemia and hypertension, defined via ICD-10 diagnosis code, treatments or values for these comorbidities] and treatment (ART and comedication).

**Results**: A total of 3289 PLHIV had at least a visit registered in the cohort in 2004 and 3880 in 2014, out of which 2138 had a visit in both years. Seventy-one percent of those were male, and in 2014 the median age was 52.2 (IQR 47.6–58.1). When compared to 2004, in 2014 there were more patients virologically suppressed (91.5% in 2014 vs. 50.9%; p<0.0001) and 72.0% in 2014 versus 43.6% patients in 2004 had CD4 count ≥500 cells/mL (p<0.0001). Table 1 shows a statistically significant increase in the prevalence of diagnosed CKD, fractures (anywhere), CVD events, hypertension, diabetes and dyslipidaemia, but also for their treatment: statins use for dyslipidaemia (9.2% in 2004 vs. 24.0% in 2014; p<0.0001); clopidogrel and aspirin use for CVD events prevention (clopidogrel: 0.8% vs. 4.1%; p<0.0001; aspirin: 0.9% vs. 8.0%; p<0.0001; 2004 and 2014, respectively). This is also reflected in the higher proportion of patients in the high risk or very high groups in the different disease risk scores for CKD, CVD and bone fracture score.

**Conclusions**: As PLHIV life expectancy increases, age-related comorbidities are increasing, leading to different needs in today's HIV disease management. Even for the patients in this analysis who present favourable HIV disease progression, there is still a significant increase on the comorbidity burden, and therefore a need for a more holistic, long-term, multidisciplinary approach that considers not only the ART, but also lifestyle, to manage HIV patients, potentially leading to improved outcomes.

**Abstract O212–Table 1:** Prevalence of comorbidities, their treatments and risk factors in 2004 and 2014

	2004; N = 2,138	2014; N = 2,138	p
Patients with CD4 count ≥500 cells/mL, %	43.6	72.0	<0.0001
Patients with HIV RNA >50 copies/mL, %	50.9	91.5	<0.0001
Prevalent CKD (diagnose or 2 consecutive eGFR <60), %	3.6	18.3	<0.0001
DAD CKD high risk score, %	29.9	50.7	<0.0001
Prevalent fractures (anywhere), %	0.7	7.0	<0.0001
10-year FRAX high risk score group, %	0.3	2.9	<0.0001
Prevalent CVD events (ever), %	3.6	14.0	<0.0001
DAD CVD very high risk score group, %	5.3	19.9	<0.0001
Framingham high risk score group, %	11.6	26.2	<0.0001
Patients on clopidogrel, %	0.8	4.1	<0.0001
Patients on aspirin, %	0.9	8.0	<0.0001
Prevalent hypertension, %	18.8	56.3	<0.0001
On blood-lowering treatment, %	6.0	22.7	<0.0001
Prevalent diabetes, %	8.4	18.5	<0.0001
On antidiabetics, %	2.4	5.8	<0.0001
Prevalent dyslipidaemia, %	14.3	54.5	<0.0001
On treatment with statins, %	9.2	24	<0.0001

CKD, chronic kidney disease; CVD, cardiovascular disease; eGFR, glomerular filtration; DAD, Data Collection on Adverse Events of Anti-HIV Drugs; FRAX, WHO Fracture Risk Assessment tool.

# Sub article: O213 Long-term impact of lipodystrophy on the risk of morbidity and mortality: a 20-year longitudinal cohort study

**Published:** 2016-10-23

**Authors:** Gemma Sanchez, Ana Gonzalez-Cordon, Jhon Rojas, Jose Blanco, Jordi Blanch, Montserrat Lonca, Berta Torres, Maria Martinez-Rebollar, Montserrat Laguno, Amparo Tricas, Ana Rodriguez, Josep Mallolas, Jose Gatell, Judit Peñafiel, Elisa de Lazzari, Esteban Martinez

**Affiliations:** Hospital Clínic-Institut d'Investigacions Biomèdiques August Pi i Sunyer, University of Barcelona, Infectious Diseases Unit, Barcelona, Spain

## Abstract

**Introduction**: Lipodystrophy is considered to accelerate the process of aging in HIV-infected persons, but long-term data showing an impact on morbidity and mortality are lacking. We hypothesized that lipodystrophy would increase the risk of comorbidities and death.

**Methods**: Within a previously well-defined cohort 1, including all consecutive antiretroviral-naive HIV-infected adults who began two nucleoside reverse transcriptase inhibitors plus at least one protease inhibitor from October 1996 to September 1999, moderate or severe body fat changes were clinically assessed and categorized as lipoatrophy (LA), lipohypertrophy (LH) or both lipodystrophy (LD). Clinical and laboratory data were periodically registered into a specific database. Patients were followed until December 2015, death or lost-to-follow-up, whichever came first. A person-years analysis was used to calculate the incidence of specific non-AIDS comorbidities (first diagnosis), AIDS events (new events) or death. Incidences were compared with Poisson or negative binomial regression models.

**Results**: Of 494 patients included, 118 (24%) developed LA only, 20 (4%) LH only, and 89 (18%) both; 71 (14%) patients died and 106 (21%) were lost to follow-up. Increasing age, HIV acquisition through injecting drug use and lack of hepatitis C co-infection were factors significantly associated with any LA (n=207, 42%), any LH (n=109, 22%) or any LD (n=227, 46%). Both patients with any LA or any LH had significantly higher total cholesterol and triglycerides at the end of follow-up relative to patients without any LA or any LH, respectively. Patients with any LA (but no patients with LH) also had significantly higher CD4 cell counts (572 vs 492 per mm^3^, p=0.0025), higher proportion of viral suppression in plasma (87% vs. 69%, p<0.0001) and higher haemoglobin (146 vs. 145 g/dL, p=0.0210) at the end of follow-up compared with patients without any LA. Incidence rate ratios (IRR) (95% CI) of any LA, any LH or any LD for non-AIDS comorbidities, AIDS events or death are shown in Table 1 (IRR 1 is the reference for patients without both LA and LH).

**Conclusion**: Hypertension and diabetes were the only non-AIDS comorbidities showing a higher risk in patients with any LA, any LH or any LD. However, contrary to our hypothesis, any LA, any LH or any LD were associated with a lower risk of death; in addition, any LA or any LD (but not any LH) were also associated with a lower risk of AIDS events and hepatic decompensation.

**Reference:**

1. Martinez E, Mocroft A, García-Viejo MA, Pérez-Cuevas JB, Blanco JL, Mallolas J, et al. Risk of lipodystrophy in HIV-1-infected patients treated with protease inhibitors: a prospective cohort study. Lancet. 2001;357:592–8. doi: http://dx.doi.org/10.1016/S0140-6736(00)04056-3

**Abstract O213–Table 1:** Incidence rate ratios (IRR) (95% CI) of any lipoatrophy (LA), any lipohypertrophy (LH) or both lipodystrophy (LD) for non-AIDS comorbidities, AIDS events or death

Outcomes	Any LA (n=207)	Any LH (n=109)	Any LD (n=227)
Any non-AIDS comorbidity (n=379)	1.05 (0.81–1.36)	1.25 (0.93–1.68)	1.06 (0.82–1.38)
Hypertension (n=70)	1.77 (1.10–2.86)	2.41 (1.49–3.90)	2.00 (1.22–3.28)
Non-AIDS neoplasia (n=65)	1.19 (0.73–1.93)	1.21 (0.70–2.11)	1.04 (0.64–1.70)
Cardiovascular events (n=56)	1.30 (0.77–2.19)	1.38 (0.78–2.47)	1.53 (0.89–2.61)
Diabetes mellitus (n=42)	2.27 (1.19–4.31)	2.51 (1.35–4.65)	2.51 (1.28–4.90)
Bone fractures (n=39)	0.53 (0.27–1.03)	0.61 (0.26–1.46)	0.58 (0.31–1.11)
Chronic kidney disease (n=38)	1.09 (0.58–2.05)	1.05 (0.50–2.22)	1.06 (0.56–2.00)
Chronic obstructive pulmonary disease (n=34)	0.58 (0.29–1.17)	1.06 (0.48–2.34)	0.57 (0.29–1.15)
Hepatic decompensation (n=18)	0.31 (0.10–0.93)	–	0.27 (0.09–0.82)
Neurocognitive impairment (n=17)	0.58 (0.22–1.58)	0.73 (0.21–2.52)	0.51 (0.19–1.38)
AIDS events (n=104)	0.53 (0.33–0.86)	0.68 (0.37–1.24)	0.53 (0.33–0.85)
Death (n=71)	0.41 (0.25–0.70)	0.48 (0.25–0.92)	0.43 (0.26–0.71)

# Sub article: O214 Higher rates of neuropsychiatric adverse events leading to dolutegravir discontinuation in women and older patients

**Published:** 2016-10-23

**Authors:** Michael Sabranski, Christoph Wyen, Christian Hoffmann, Tanya Welz, Michael Kolb, Eva Wolf, Hans-Juergen Stellbrink

**Affiliations:** 1 Infectious Diseases, ICH Study Center Hamburg, Hamburg, Germany; 2 Infectious Diseases, Praxis am Ebertplatz, Cologne, Germany; 3 Infectious Diseases, MUC Research, Munich, Germany

## Abstract

**Introduction**: Dolutegravir (DTG), a second-generation integrase strand transfer inhibitor (INSTI), is now among the most frequently used antiretroviral agents. However, recent reports have raised concerns about potential neurotoxicity.

**Methods**: We performed a retrospective analysis of a cohort of HIV-infected patients who had initiated an INSTI in two large German outpatient clinics between 2007 and 2016. We compared discontinuation rates due to adverse events within 2 years of starting between DTG, raltegravir (RAL) or elvitegravir (EVG)/cobicistat. We also evaluated factors associated with DTG discontinuation.

**Results**: A total of 1950 INSTI-based therapies were initiated amongst 1704 patients eligible for analysis within the observation period. The estimated rates of any adverse event (AE) and of neuropsychiatric AEs leading to discontinuation within 12 months were 7.6% and 5.6% respectively for DTG (n=985), 7.6% and 0.7% for EVG (n=287) and 3.3% and 1.9% for RAL (n=678). Neuropsychiatric AEs leading to DTG discontinuation were observed more frequently in women (hazard ratio [HR] 2.64; 95% CI 1.23–5.65, p=0.012), in patients older than 60 years (HR 2.86; 95% CI 1.42–5.77, p=0.003) and in HLA-B*57:01-negative patients who initiated abacavir at the same time (HR 2.42; 95% CI 1.38–4.24, p=0.002).

**Conclusion**: In this large cohort, the discontinuation rate of DTG due to neuropsychiatric AEs was significantly higher than with the other INSTIs at almost 6% within 12 months. Almost three-fold higher discontinuation rates observed amongst women and older patients underscore the need for further investigation, especially in patient populations usually under-represented in clinical trials.

# Sub article: O215 Cognitive function and depression in HIV-positive individuals and matched controls

**Published:** 2016-10-23

**Authors:** Davide De Francesco, Jonathan Underwood, Marta Boffito, Frank Post, Patrick Mallon, Jaime Vera, Ian Williams, Jane Anderson, Margaret Johnson, Caroline Sabin, Alan Winston

**Affiliations:** 1 Department of Infection & Population Health, University College London, London, UK; 2 Division of Infectious Diseases, Imperial College London, London, UK; 3 Pharmacokinetic Research Unit, Chelsea and Westminster Hospital, London, UK; 4 HIV Research Centre, King's College Hospital, London, UK; 5 School of Medicine, University College Dublin, Dublin, Ireland; 6 Lawson Unit, Brighton and Sussex Medical School, Brighton, UK; 7 Centre for the Study of Sexual Health and HIV, Homerton University Hospital, London, UK; 8 HIV/AIDS services, Royal Free Hospital, London, UK

## Abstract

**Introduction**: Cognitive disorders and depression remain prevalent in people living with HIV (PLWH) [1, 2]; however, few studies have investigated the interaction between these comorbidities. We describe overall cognitive function in a large cohort of PLWH compared to an appropriate control population and explore factors associated with cognitive performance, including depression and lifestyle factors.

**Methods**: One thousand two hundred and sixty-six individuals (643 PLWH aged ≥50 years, 343 PLWH <50 years and 280 HIV-negative controls ≥50 years) were enrolled in the Pharmacokinetic and Clinical Observations in People over Fifty study and completed a computerised assessment (CogState) of cognitive function covering six domains. Raw test scores were standardized into Z-scores (mean 0, SD 1) and averaged to obtain domain and global Z-scores. Depression was evaluated via the Patient Health Questionnaire (PHQ-9) and classified as none (score 0–4), mild (5–9), moderate (10–14) or severe (15–27). Differences between the three groups and the effect of depression, socio-demographic and lifestyle factors on cognitive performance were evaluated using median regression. All analyses accounted for age, gender, ethnicity and level of education.

**Results**: PLWH aged ≥50 and <50 years and HIV-negative controls aged ≥50 were predominantly male (88%, 81% and 65%, respectively), of white ethnicity (87%, 81% and 90%, respectively), with a median age (IQR) of 56 (53–62), 43 (37–47) and 58 (53–63) years, respectively. Current alcohol consumption and recreational drugs use were reported in 80%, 81% and 87% (p=0.009) and 26%, 34% and 15% (p<0.001), respectively, of PLWH aged ≥50 and <50 years and HIV-negative controls. After adjusting for socio-demographics, PLWH aged ≥50 and <50 years had reduced global cognitive scores than HIV-negative controls [adjusted difference between medians (95% CI) was −0.090 (−0.149, −0.032), p=0.003, and −0.080 (−0.166, 0.007), p=0.07, respectively]. Moderate or severe depression was more prevalent in PLWH aged ≥50 (27%, p<0.001) and <50 years (22%, p<0.001) compared to HIV-negative controls (8%). Depression (p<0.001), years of drinking (p=0.003), smoking status (p=0.01) and use of marijuana (p=0.02) were associated with cognitive function in univariate analyses, but only depression (p<0.001) and years of drinking (p=0.008) remained significantly associated in multivariable analyses. After further adjusting for depression and years of drinking, differences between groups were not statistically significant (Figure 1).

Unadjusted (dotted line), adjusted for age, gender, ethnicity and level of education only (dashed line) and adjusted for age, gender, ethnicity, level of education, depression and years of drinking.

**Conclusion**: Reduced cognitive performance in HIV infected individuals appears to be partially mediated by depressive disorders. However, this may also indicate that depression and cognitive impairment in PLWH share a common aetiology and could be related to similar underlying inflammatory processes.

**References:**

1. Heaton RK, Franklin DR, Ellis RJ, McCutchan JA, Letendre SL, LeBlanc S, et al. HIV-associated neurocognitive disorders before and during the era of combination antiretroviral therapy: differences in rates, nature, and predictors. J Neurovirol. 2011;17:3–16. doi: http://dx.doi.org/10.1007/s13365-010-0006-1

2. Magidson JF, Skeer MR, Mayer KH, Safren SA. Prevalence of psychiatric and substance abuse symptomatology among HIV-infected gay and bisexual men in HIV primary care. Psychosomatics. 2015;56:470–8. doi: http://dx.doi.org/10.1016/j.psym.2014.08.004

**Abstract O215–Figure 1:** Differences (with 95% CI) in the median global Z-score across cohorts (≥50 PLWH, <50 PLWH and HIV-negative controls).

# Sub article: O221 Novel HCV therapies: what have we achieved and remaining challenges?

**Published:** 2016-10-23

**Authors:** Andri Rauch

**Affiliations:** Bern University Hospital, University of Bern, Bern, Switzerland

## Abstract

Novel hepatitis C virus (HCV) therapies have revolutionized the management of hepatitis C in only half a decade. The new direct-acting antiviral agents (DAAs) inhibit the HCV protease, the NS5A protein or the NS5B polymerase, and hereby block viral replication, assembly and release. These drugs have greatly improved treatment tolerability and efficacy compared with the previous interferon-based therapies. Fortunately, the promising results of clinical trials hold true in real-life settings. Even previously difficult-to-treat populations, including cirrhosis, can now be treated with safe and extremely effective regimens. However, despite the unprecedented success of novel HCV treatments, challenges remain: the most urgent priority is to greatly increase treatment uptake, which is only possible if DAA costs are lowered substantially. The remarkable success in scaling-up HCV treatments in Egypt exemplifies how coordinated efforts involving patients, physicians and public health authorities can impact the global burden of HCV infection. Another challenge is the emergence of HCV epidemics among HIV-infected men who have sex with men, and the surge in HCV infections among people who inject drugs in Eastern Europe and Southeast Asia. These epidemics can only be controlled if advances in treatment uptake and efficacy are accompanied by reductions in high-risk behaviour. Finally, it is important to note that cure of HCV infection substantially reduces the risk of liver-related complications, but does not eliminate it, particularly in those with multiple liver-related risk factors. Despite remaining challenges, the future of HCV therapy is bright with the potential to eliminate one of the most common infectious diseases worldwide.

# Sub article: O222 HPV-associated malignancies in HIV

**Published:** 2016-10-23

**Authors:** Deborah Konopnicki

**Affiliations:** Service des Maladies Infectieuses, CHU Saint-Pierre, Brussels, Belgium

## Abstract

Oncogenic human papillomaviruses (HPV) are responsible for the development of cancer and pre-cancerous lesions in the anogenital area (namely in the cervix, vulva and vagina, penis and anus) and in the oropharyngeal cavity. These lesions are more frequent and more difficult to treat in HIV-positive patients. In Europe, less than 30% of HIV-positive men and less than 70% of HIV-positive women have access to anal and cervical cancer screening, respectively. We will discuss the different strategies of screening for these cancers, which have been recently developed or are currently under investigation in randomized controlled studies and how to implement them. Preventive vaccines against HPV have been available for almost a decade, and their use in primary and secondary prophylaxis should be proposed to HIV-positive patients. Issues on vaccines schedules, costs and HPV genotypes coverage will be raised. Non-invasive therapy of HPV-related lesions, such as local administration of antiviral drugs or the use of therapeutic vaccines, will be presented.

# Sub article: O223 Screening for malignancies: what is new?

**Published:** 2016-10-23

**Authors:** Jean-Philippe Spano

**Affiliations:** Department of Medical Oncology, University Institute of Oncology (IUC)/University Pierre and Marie CURIE (UPMC), Paris, France

## Abstract

Since the emergence of the HIV pandemic, several viral-induced cancers were frequently diagnosed: Kaposi's sarcoma associated with human herpesvirus 8, non-Hodgkin lymphoma mostly associated with Epstein–Barr virus and cervical cancer associated with oncogenic human papillomavirus types. However, in the late 1990s, several population-based cohort studies, comparing the incidence of cancers in the HIV population and in the general population, found higher rates of other cancers (defined as non-AIDS-related malignancies) in people living with HIV (PLWHIV), such as anal cancer, Hodgkin lymphoma (HL), skin cancers, lip cancer, liver cancer and lung cancer. In the era of highly active antiretroviral therapy, additional registry studies and meta-analyses have also shown an increased rate of cancers in PLWHIV compared with the general population. Nowadays, the most frequent are HL, cancers of the lung, anus and liver. Factors implicated in this increased incidence, such as higher rates of smoking, chronic immunodeficiency and oncogenic virus, are among the main ones. Strategies to increase cancer survival in PLWHIV are needed. Beyond the recommendations in terms of therapeutic strategies likely used in the general population, screening programme and prevention actions can provide a positive impact on survival, such as in the general population. For instance, for lung cancer screening with low-dose chest computed tomography has shown to be efficient in the general population at risk, so why not in PLWHIV? The objective of this presentation is to discuss the main screening for malignancies in PLWHIV and to present some guidelines, already approved for some cancer types.

# Sub article: O224 Differences in virological and immunological risk factors for non-Hodgkin lymphoma (NHL) and Hodgkin (HL): the D:A:D study

**Published:** 2016-10-23

**Authors:** Leah Shepherd, Lene Ryom, Matthew Law, Camilla Hatleberg, Stephane de Wit, Antonella d'Armini Monforte, Manuel Battegay, Andrew Phillips, Fabrice Bonnet, Peter Reiss, Christian Pradier, Andrew Grulich, Caroline Sabin, Jens Lundgren, Amanda Mocroft

**Affiliations:** 1 Department of Infection and Population Health, University College London, London, UK; 2 Department of Infectious Diseases, CHIP, Section 2100, Rigshospitalet, University of Copenhagen, Copenhagen, Denmark; 3 The Kirby Institute, University of New South Wales, Sydney, Australia; 4 Division of Infectious Diseases, Saint Pierre University Hospital, Université Libre de Bruxelles, Brussels, Belgium; 5 Dipartimento di Scienze della Salute, Clinica di Malattie Infectitive e Tropicali, Azienda Ospedaliera-Polo Universitario San Paolo, Milan, Italy; 6 Division of Infectious Diseases & Hospital Epidemiology, University Hospital Basel, Basel, Switzerland; 7 Université de Bordeaux, CHU de Bordeaux and INSERM U897, Talence, France; 8 Division of Infectious Diseases, Academic Medical Centre, University of Amsterdam, Amsterdam, Netherlands; 9 Department of Global Health, HIV Monitoring Foundation, Amsterdam, Netherlands; 10 Department of Public Health, Nice University Hospital, Nice, France

## Abstract

**Introduction**: Non-Hodgkin (NHL) and Hodgkin lymphomas (HL) are common in HIV+ people. Since the introduction of cART, a decline in NHL but not HL incidence has been observed. Factors affecting risk of NHL and HL appear to differ in HIV+ persons.

**Materials and methods**: D:A:D participants were followed from the earliest of study entry or January 1, 2004 until first NHL or HL diagnosis, last visit plus 6 months, death, or February 1, 2015. Crude incidence rates (IR) of NHL and HL were calculated. Adjusted incidence rate ratios (aIRR) were calculated using Poisson regression with generalized estimating equations. Both current and historical measures of HIV viral load (VL) (current level, area under the curve [AUC] during follow-up) and CD4 (current and nadir level, AUC) were considered. Other risk factors investigated are listed in the footnote to Figure 1.

**Results**: About 41,583 persons were included contributing 337,020 person-years of follow-up (PYFU) [median of 9 (IQR 6–11) years per person]. Of which, 392 developed NHL (IR 1.2/1000 PYFU, 95% CI 1.1–1.3) and 149 developed HL (IR 0.4/1000 PYFU, 95% CI 0.4–0.5). In age-adjusted analyses, NHL incidence declined by 15% (95% CI 12–17)/year from 2004–2015, whereas IR of HL was stable (change/year: −3% (95% CI −8–2%). At diagnosis, persons who developed HL versus NHL were of similar age (46.2 vs. 46.9 years, p=0.25), with a higher current CD4 (400 vs. 325 cells/mm^3^, p<0.01), lower current log_10_ VL (1.7 vs. 2.3 log_10_ copies/mL, p<0.01), and lower log_10_ VL AUC (4.8 vs. 5.1 log_10_ copies/mL, p<0.01). After adjustment, the IR of NHL and HL was over 50% lower in females relative to males (Figure 1). Persons of older age had higher IR of NHL only. Lower current CD4 was the strongest predictor of higher NHL IR; however, higher current VL and VL AUC were also associated. Nadir and CD4 AUC were not associated with NHL after adjustment for current CD4 (both p>0.05). The declining trend over time in NHL IR attenuated, but remained after adjustment (−8% (95% CI −4%–11%)/year). HL IR was also associated with lower current CD4, but not with other markers of VL or CD4 in addition to current CD4. HL IR remained stable over time (aIRR 1.01; 95% CI 0.95–1.07).

**Conclusion**: NHL incidence was associated with lower current CD4 and both current and historical exposure to viral replication, suggesting historical exposure to uncontrolled viral replication may play a part in NHL development in addition to current immunodeficiency. Conversely, HL incidence was elevated in those with current immunodeficiency, but current and historical exposure to uncontrolled HIV replication were not associated when adjusting for current CD4.

**Abstract O224–Figure 1:** Figure adjusted incidence rate ratios (aIRR) for NHL and HL.

# Sub article: O233 Tackling the HCV epidemic in the EECA region: a physician's perspective

**Published:** 2016-10-23

**Authors:** Nikoloz Chkhartishvili

**Affiliations:** Infectious Diseases, AIDS and Clinical Immunology Research Center, Tbilisi, Georgia

## Abstract

Eastern Europe and Central Asia (EECA) has the largest hepatitis C virus (HCV) epidemic in the World Health Organization European region. The epidemic in EECA has been closely linked to economic, social and public health dislocations in the 1990s that created an environment supportive of the rapid spread of HCV. It is estimated that 6 million people are living with chronic hepatitis C infection in the region, including up to 2.5 million people who inject drugs (PWID). While EECA is home to a large drug epidemic, the coverage with prevention interventions, such as needle exchange and opioid substitution therapy, is very low, and this contributes to sustainment and further growth of the HCV epidemic. There is a significant burden of HCV among people living with HIV in EECA, with prevalence exceeding 80% among HIV-positive PWID. There are differences in the HCV prevalence between countries, with Georgia having the highest adult viraemic prevalence. Access to HCV therapy in the region is limited and only a small proportion of patients receive appropriate treatment. Georgia is an exception to this situation, where the national HCV elimination programme was launched in April 2015 as a result of support from the US Centers for Disease Control and Prevention and commitment from Gilead Sciences to donate direct-acting antiviral agents (DAAs). The goal of the programme is to eliminate HCV primarily through the test and treat approach, strengthened by prevention interventions, such as harm reduction and infection control. While accessibility and affordability of DAAs in the EECA region is a key issue, effective response to the HCV epidemic will also require expansion of prevention programmes, particularly among PWID.

# Sub article: O311 Update on the evidence for PrEP effectiveness

**Published:** 2016-10-23

**Authors:** Sheena McCormack

**Affiliations:** MRC Clinical Trials Unit, University College London, London, UK

## Abstract

Nine randomized controlled clinical trials have demonstrated a reduction in HIV incidence when antiretrovirals are offered to HIV-negative individuals as tenofovir-based oral regimens, tenofovir vaginal gel or dapivirine released from a vaginal ring. Three trials failed to show benefit; all three were placebo-controlled, and all three were conducted in women in low-income settings in sub-Saharan Africa. The diversity of populations studied in the nine trials, with a positive result, underscores the breadth of impact that adding antiretrovirals to the toolkit for HIV-negative individuals could have. The impact is apparent in some cities in the United States but yet to be realized elsewhere as other countries have been slow to accept the evidence and implement models of delivery. The commonest reason for this delay is the cost of the drug as demand for pre-exposure prophylaxis (PrEP) is uncertain, although countries that do not currently fund sexual health services or HIV prevention services tailored to key populations are also struggling to work out the feasibility and cost of delivering PrEP. Feasibility and cost of delivery are not reasons for delay in the UK as a well-established, professionally linked network of sexual health clinics already exists, albeit with ever-diminishing funding. As well as the cost of the drug, policy makers cite concerns about an increase in sexually transmitted infections as a result of PrEP. The evidence from pre-PrEP Europe demonstrates that syphilis and gonorrhoea have been increasing for a decade in men who have sex with men, and this mirrors an increase in new HIV infections. PrEP may contribute further to the increase in STIs but at least HIV will be curtailed. Whilst policy makers deliberate, key populations are purchasing PrEP for themselves and services have already changed practice to support them.

# Sub article: O312 Brief overview of cost-effectiveness of PrEP

**Published:** 2016-10-23

**Authors:** Valentina Cambiano

**Affiliations:** Department of Infection and Population Health, University College London, London, UK

## Abstract

HIV incidence among men who have sex with men (MSM) remains high despite the widespread use of antiretroviral therapy (ART) and high rates of virological suppression. Pre-exposure prophylaxis (PrEP) has been shown to be highly effective in preventing infections in MSM. Healthcare systems are facing the decision of whether to introduce it. The overall goal of the cost-effective analyses is to maximize the health of the population with the available budget. Previous modelling studies based around MSM in North America and Australia found PrEP to be generally not cost-effective. However, there are issues to consider over appropriate costs, time horizon considered and groups targeted. We present results from two cost-effective evaluations of introducing PrEP among MSM in the UK (which I led) and in The Netherlands (led by Brooke Nichols). The two evaluations take into account a time horizon long enough to evaluate the full benefit of PrEP, target similar groups to those in the PROUD and IPERGAY studies, and consider a realistic uptake. While the UK and Dutch models differ greatly in structure, the primary conclusions are the same – PrEP can generally be considered cost-effective (or even cost-saving) for use among MSM in Europe, when appropriately considering a long time scale, given that each infection averted is saving the health service ART costs for many decades to come. However, if the time horizon under consideration is – we would argue inappropriately – restricted to the short-medium term, significant reductions in drug prices are required for PrEP to be considered cost-effective.

# Sub article: O313 Lessons from implementation in France

**Published:** 2016-10-23

**Authors:** Jean-michel Molina

**Affiliations:** Department of Infectious Diseases, University of Paris Diderot, Paris, France

## Abstract

In the wake of the striking efficacy results of the PROUD and IPERGAY trials, France has approved the use of TDF/FTC for PrEP to reduce the risk of sexual acquisition of HIV among adults in January 2016. In July 2016, more than 1000 patients have been registered on the Gilead website put in place at the request of the French Drug Agency to collect information on patients’ characteristics, PrEP efficacy and safety. PrEP is fully covered by the National Health System. Visits to doctors and tests for PrEP monitoring are partly reimbursed. PrEP can only be prescribed in hospitals and Sexual Health Clinics. More than 90 clinics are currently offering PrEP throughout France. The vast majority of people requesting PrEP self-select and are MSM (96.4%), although PrEP is also recommended in other high-risk individuals but prevention campaigns on the use of PrEP have not yet started. Interestingly, more than 60% of people receive PrEP on demand according to the dosing regimen used in the IPERGAY trial, and so far only two seroconversions have been reported in patients most likely infected at the time they started PrEP. Also 30% of patients had experienced STIs before starting PrEP, and the implementation of PrEP is a clear opportunity for a better prevention, diagnosis and treatment of STIs. Finally, the implementation of PrEP programmes represents a challenge for hospitals and STI clinics since no additional resources have been provided, suggesting more research is needed in defining the best models of care delivery and monitoring.

# Sub article: O314 Utilization of emtricitabine/tenofovir (FTC/TDF) for HIV pre-exposure prophylaxis in the United States by gender (2013-1Q2016)

**Published:** 2016-10-23

**Authors:** Staci Bush, Keith Rawlings, David Magnuson, Patty Martin, Olga Lugo-Torres, Robertino Mera-Giler

**Affiliations:** 1 Medical Affairs, Gilead Sciences, Foster City, CA, USA; 2 Drug Safety & Public Health, Gilead Sciences, Foster City, CA, USA; 3 Epidemiology, Gilead Sciences, Foster City, CA, USA

## Abstract

**Introduction**: The availability of FTC/TDF for pre-exposure prophylaxis (PrEP) in combination with other strategies to reduce the risk of sexually acquired HIV-1 in adults at high-risk has altered the HIV prevention landscape in the United States. While previous analyses have shown gender differences among individuals started on FTC/TDF for PrEP, this is the first study to explore the characteristics and differences in utilization trends by gender and race.

**Materials and methods**: National electronic patient-level data were collected from 82% of all US retail pharmacies that dispensed FTC/TDF between January 1, 2013 and March 31, 2016. A previously described algorithm identified use of FTC/TDF for PrEP. De-identified prescription refill data, medical claims and patient demographics were analyzed through categorical methods. Data were projected to all retail pharmacies in the United States.

**Results**: During this time period, a total 67,403 unique individuals started FTC/TDF for PrEP in the United States. Overall, women accounted for 9685 (14.4%) of total FTC/TDF for PrEP users. Age distribution revealed that 9.8% of men and 24.1% of women were <24 years of age. Between 1Q2013 and 1Q2016 quarter-over-quarter utilization grew 770%; with growth of 72% for women and 1350% for men. The Northeast was the region with both the largest percentage of overall FTC/TDF for PrEP starts (29.3%) as well as unique starts among women (35.2%), while the Western US had the highest percentage of starts in men (89.9%). Table 1 shows the gender differences in FTC/TDF for PrEP utilization by race.

For the five quarters ending March 2016, White women were 3.4 and 4.2 times more likely to start PrEP than Black or Hispanic women, while White men were 8.1 and 6.6 times more likely to start than their Black or Hispanic counterparts.

**Conclusion**: The overall population of PrEP user increased steadily since 2012. The number of starts among males significantly increased while female uptake remained flat for all racial groups. Despite having some of the highest lifetime risks of acquiring HIV (1 in 2 for Black men and 1 in 48 among Black women) PrEP starts have been disproportionately low. HIV prevention messaging and services need to be gender and racially focused to decrease new infections in populations with the most severe burden of HIV in the United States.

**Table 1:** Gender differences in FTC/TDF for PrEP utilization by race

	Black	White	Hispanic	Asian
Female	17%	65%	15%	3%
Male	9%	76%	11%	3%

# Sub article: O315 InterPrEP: internet-based pre-exposure prophylaxis (PrEP) with generic tenofovir DF/emtricitabine (TDF/FTC) in London – analysis of pharmacokinetics, safety and outcomes

**Published:** 2016-10-23

**Authors:** Xinzhu Wang, Nneka Nwokolo, Roxanna Korologou-Linden, Andrew Hill, Gary Whitlock, Isaac Day-Weber, Myra McClure, Marta Boffito

**Affiliations:** 1 Faculty of Medicine, Imperial College London, London, UK; 2 Chelsea and Westminster Hospital, London, UK; 3 St Stephen's AIDS Trust, Chelsea and Westminster Hospital, London, UK

## Abstract

**Introduction**: The UK National Health Service (NHS) does not provide TDF/FTC for PrEP in the UK. This forces people to purchase generic versions on the internet (e.g. www.iwantprepnow.co.uk [1], which is legal under UK import laws. However, there are concerns about the authenticity of medicines purchased online. In February 2016, we established an innovative service offering plasma tenofovir (TFV) and FTC therapeutic drug monitoring (TDM) for people buying generic PrEP online, to ensure that drug concentrations were consistent with previously published data.

**Materials and methods**: HIV-negative individuals who attended the GUM clinic (from February to June 2016) who reported purchasing PrEP on the internet were offered TDM. TFV and FTC plasma concentrations were measured by ultra-performance liquid chromatography coupled with UV detection, with a linear range of 25 to 10,000 ng/mL. Evaluation of renal function and testing for HIV and STIs (chlamydia, gonorrhoea, syphilis) were also performed, at baseline and every 3 months, with risk reduction advice provided.

**Results**: About 161 individuals presented after purchasing PrEP on the internet: 74% were White, 94% were taking daily PrEP and 6% event-driven. The majority of patients received generic TDF/FTC from Cipla Ltd. There were 118 patients with TDM results: median (range) TFV concentration was 104 ng/mL (23–1400 ng/mL); median (range) FTC concentration was 157 ng/mL (27–1876 ng/mL). All TFV and FTC concentrations were above our established median plasma TFV and FTC cut-offs of 19 ng/mL and 22 ng/mL, respectively, at 24 hours post-dose, based on previously published data [2, 3]. Seven samples were repeated; six were confirmed to be above cut-off. Baseline eGFR was normal in all evaluable individuals. Thirty-nine (26%) had an STI at baseline or within 3 months of starting PrEP and 13 had an STI at a follow-up visit. No new cases of HIV, hepatitis B/C were seen in this cohort.

**Conclusions**: In a population at high-risk of STI who cannot yet access PrEP from the UK NHS, concentrations of TFV and FTC in generic formulations purchased over the internet were similar to those on the original formulation from Gilead, which have demonstrated high levels of protection against HIV infection in previous clinical trials.

**References:**

1. I Want PrEP Now. I Want PrEP Now. [cited 2016 July 30] Available from: http://www.iwantprepnow.co.uk

2. Dickinson L, Yapa HM, Jackson A, Moyle G, Else L, Amara A, et al. Plasma tenofovir, emtricitabine and rilpivirine and intracellular tenofovir diphosphate and emtricitabine triphosphate pharmacokinetics following drug intake cessation. Antimicrob Agents Chemother. 2015;59(10):6080–6. doi: http://dx.doi.org/10.1128/AAC.01441-15

3. Donnell D, Baeten J, Bumpus NN, Brantley J, Bangsberg DR, Haberer JE, et al. HIV Protective efficacy and correlates of tenofovir blood concentrations in a clinical trial of PrEP for HIV prevention. J Acquir Immune Defic Syndr. 2014;66(3):340–8. doi: http://dx.doi.org/10.1097/QAI.0000000000000172

# Sub article: O316 New approaches and new technologies to improve access to HIV testing

**Published:** 2016-10-23

**Authors:** Teymur Noori

**Affiliations:** European Centre for Disease Prevention and Control, Stockholm, Sweden

## Abstract

The epidemiological trends of HIV among men who have sex with men (MSM) in Europe will be discussed in this presentation. The speaker, Teymur Noori, who works at the European Centre for Disease Prevention and Control (ECDC), and is responsible for monitoring the HIV response in Europe and Central Asia through the Dublin Declaration monitoring process, will make the case that additional tools for HIV prevention among MSM are needed in order to reduce HIV infections. The latest data on the status of pre-exposure prophylaxis (PrEP) implementation in Europe will also be discussed. In addition, results from a PrEP survey conducted in collaboration with the Hornet Gay Social Network on the informal use of PrEP among Hornet network users in Europe will be shown.

# Sub article: O317 Implementation from the community perspective

**Published:** 2016-10-23

**Authors:** Bruno Spire

**Affiliations:** 1 French National Institute for Medical Research (INSERM), Marseilles, France; 2 AIDES, Marseilles, France

## Abstract

In France, over the last decade, AIDES has been strongly involved in research to support early community access to biomedical prevention tools. As a community-based organization founded in 1984 and engaged in advocacy as well as in the provision of services to people living with HIV and most at-risk groups, AIDES has extensively contributed to major shifts in the French HIV Public Health policy. Among its achievements are the implementation or rapid HIV and HCV tests, a drug users’ peer education programme on safe injecting practices and, more recently, access to HIV pre-exposure prophylaxis (PrEP). From a community perspective, implementing PrEP in real life is far more complex than in research. Despite scientific evidence and a wide consensus among researchers, clinicians and the community, months of constant advocacy work were necessary to reach a legal frame around access to PrEP. Scaling-up PrEP was impeded by existing regional inequalities in access to care, along challenges at the level of care centres. Also, the implementation of PrEP requires an increase of human resources and dedicated funding, which creates new challenges for community-based organizations: how can we implement a new programme without limiting other pre-existing ones - in a context of ongoing budget cuts? Finally, it is essential to overcome hesitations and reluctance among community leaders and healthcare workers; and to adequately inform those people who would benefit the most from PrEP, and encourage them to use PrEP.

# Sub article: O321 Immunology of HIV persistence: implications for the development of a cure

**Published:** 2016-10-23

**Authors:** Steven G Deeks

**Affiliations:** Department of Medicine, University of California, San Francisco, CA, USA

## Abstract

Given the challenge of delivering complex, expensive and potentially harmful antiretroviral therapy on a global level, there is intense interest in the development of short-term, well-tolerated regimens that allow individuals to interrupt therapy indefinitely without experiencing a rebound in viraemia. Recent heroic interventions, such as haematopoietic stem cell transplant, suggest that dramatic reductions in the reservoir size can be achieved, but that complete eradication will be challenging, if not impossible. Experience with ‘elite’ and post-treatment controllers suggest that disease remission may be achievable even if replication-competent HIV persists. Achieving a sustained remission will likely require a low reservoir size and a potent and durable HIV-specific immune response. Cancer and HIV persistence share a number of similarities. In each case, a rare population of cells with the capacity to cause harm becomes established in difficult to reach tissues. The local environment in each case is reshaped to prevent immune mechanisms from clearing the diseased cell. Specifically, a chronic inflammatory environment stimulates an immunosuppressive response, and therapies that target these immune pathways have either been very successful (in cancer) or are now entering the clinic (in HIV disease). These interventions have the potential to enable successful repurposing of preventative vaccines into the HIV cure arena, and will be reviewed. It is hoped that advances in cancer therapy will be translated into the HIV arena, accelerating our discovery of a potential HIV cure.

# Sub article: O322 Where next for ARVs?

**Published:** 2016-10-23

**Authors:** Roy M Gulick

**Affiliations:** Weill Cornell Medicine, New York, NY, USA

## Abstract

Currently, there are 29 antiretroviral (ARV) drugs approved for the treatment of HIV infection in six mechanistic classes. ARV therapy (ART) guidelines worldwide recommend an initial treatment regimen consisting of a combination of two nucleoside analogue reverse transcriptase inhibitors (NRTIs) and a third drug, either a non-nucleoside reverse transcriptase inhibitor (NNRTI), a boosted protease inhibitor (PI) or an integrase inhibitor (II). Current ART regimens are highly potent, safe, tolerable and convenient. Current virological suppression rates can exceed 90% in clinical trials and cohort studies, and one pill, once-daily regimens are widely available. Newer strategies, formulations and investigational ARV agents continue to move ARVs forward. Although a three-drug ART regimen is standard, potent two-drug regimens are under investigation. Long-acting compounds are under study, including an injectable investigational formulation of the approved NNRTI, rilpivirine, and an investigational II, cabotegravir, that can be dosed together every 1 to 2 months. Other investigational formulations include implantable devices that provide sustained release of ARVs, and other newer technologies. The investigational ARV pipeline contains new agents in existing classes (NRTI, NNRTI, PI, II) and some of these are associated with either less toxicity [e.g. NRTI, tenofovir pro-drug TAF (tenofovir alafenamide fumarate); NNRTI, doravirine] or different resistance profiles (II, bictegravir) than current drugs. Two new mechanistic ARV classes under investigation are the CD4 attachment inhibitors and the HIV maturation inhibitors, and candidate drugs in each class are in clinical development. Currently, we can control HIV infection long-term with potent, safe and convenient ART that leads to prolonged healthy survival in our patients.

# Sub article: O331 Non-inferiority of dual-therapy (DT) with darunavir/ritonavir (DRV/r) plus 3TC versus triple-therapy (TT) with DRV/r plus TDF/FTC or ABC/3TC for maintenance of viral suppression: 48-week results of the DUAL-GESIDA 8014 trial

**Published:** 2016-10-23

**Authors:** Federico Pulido, Esteban Ribera, Maria Lagarde, Ignacio Pérez-Valero, Jesús Santos, Jose Iribarren, Antonio Payeras, Pere Domingo, José Sanz, Miguel Cervero, Adrian Curran, Francisco Rodriguez, María Téllez, Pablo Ryan, Pilar Barrufet, Hernando Knobel, Antonio Rivero, Belén Alejos, María Yllescas, José Arribas, Study Group GESIDA-8014-DUAL

**Affiliations:** 1 HIV Unit, Hospital Universitario 12 de Octubre, Madrid, Spain; 2 Servicio de Enfermedades Infecciosas, Hospital Vall d'Hebron, Barcelona, Spain; 3 HIV Unit, Hospital La Paz, IdiPAZ, Madrid, Spain; 4 Unidad de Enfermedades Infecciosas, Hospital Virgen de la Victoria, Málaga, Spain; 5 Servicio de Enfermedades Infecciosas, Hospital Donostia, San Sebastián, Spain; 6 Servicio de Medicina Interna, Hospital Son Llatzer, Palma de Mallorca, Spain; 7 Servicio de Medicina Interna, Hospital de la Santa Creu i Sant Pau, Barcelona, Spain; 8 Servicio de Medicina Interna, Hospital Príncipe de Asturias, Alcalá de Henares, Spain; 9 Servicio de Medicina Interna, Hospital Severo Ochoa, Leganés, Spain; 10 Unidad de Enfermedades Infecciosas, Hospital Infanta Elena, Huelva, Spain; 11 Servicio de Medicina Interna, Hospital Clínico San Carlos, Madrid, Spain; 12 Servicio de Medicina Interna, Hospital Infanta Leonor, Madrid, Spain; 13 Servicio de Medicina Interna, Hospital de Mataró, Mataró, Spain; 14 Servicio de Enfermedades Infecciosas, Hospital del Mar, Barcelona, Spain; 15 Sección de Enfermedades Infecciosas, Hospital Reina Sofia, Córdoba, Spain; 16 Fundación de Investigación, SEIMC-GESIDA, Madrid, Spain; 17 GESIDA-8014-DUAL Study Group, Spain

## Abstract

**Introduction**: DT with DRV/r plus 3TC may be as effective, better tolerated and substantially less costly than TT with DRV/r plus TDF/FTC or ABC/3TC for maintenance of viral suppression.

**Methods**: DUAL is a 48-week multicentre, randomized, open-label, non-inferiority (margin – 12%) clinical trial. HIV-1 infected patients with <50 copies/mL for ≥6 months on TT with DRV/r plus TDF/FTC or ABC/3TC, with no resistance to DRV/r or 3TC/FTC, were randomized (1:1) to continue TT or switch to DT with DRV/r and 3TC QD. Primary endpoint: proportion of patients with HIV-1 RNA <50 copies/mL at week 48 after randomization (FDA snapshot algorithm) in the exposed-ITT population (e-ITT, excluding never exposed patients). Secondary endpoints: proportion of patients with persistently suppressed viraemia, overall tolerability, emerging resistance, changes in CD4 cells, renal function and lipids.

**Results**: Two hundred and forty-nine patients (75% with TDF/FTC, 25% with ABC/3TC) were randomized to switch to DT (n=126) or to continue TT (n=123). Baseline characteristics were balanced between arms except a significant shorter duration of virologic suppression in the DT group (21 vs. 28 months). Proportion of patients with HIV-1 RNA <50 copies/mL at week 48 (DT vs. TT): in the e-ITT population 89% (112/126), versus 93% (114/123), difference −3.8 (95% CI −11 to +3.4); in the observed population (censoring discontinuations due to non-virologic reasons) 97% (112/116), versus 98% (114/116), difference −1.7 (95% CI −5.8 to +1.4). The proportion of patients (e-ITT) maintaining HIV RNA-1 <50 copies/mL in all determinations was 89% in DT and 87% in TT (difference 1.9%; 95% CI −6.2 to +10). Severe adverse events occurred in 5% of patients in DT and 5% of patients in TT. Study drug discontinuations due to adverse events in 1% and 2% (p=NS). There was no detection of emergent resistance during the trial and no significant differences in CD4 cell recovery. Switching to DT was not associated with significant changes in e-GFR and TC/HDL ratio relative to continuing TT.

**Conclusions**: DT with DRV/r plus 3TC was non-inferior and as well tolerated as DRV/r plus TDF/FTC (or ABC/3TC) for maintenance of viral suppression. DT has the added benefit of preserving options and reducing the cost.

# Sub article: O332 French national survey of resistance to integrase inhibitors shows high differences of resistance selection rate in case of virological failure in a context of routine hospital care (ANRS-AC11 virology network)

**Published:** 2016-10-23

**Authors:** Anne-Genevieve Marcelin, Maxime Grude, Charlotte Charpentier, Pantxika Bellcave, Audrey Rodallec, Coralie Pallier, Stephanie Raymond, Audrey Mirand, Laurence Bocket, Laurence Morand-Joubert, Constance Delaugerre, Brigitte Montes, Helene Jeulin, Thomas Mourez, Samira Fafi-Kremer, Corrine Amiel, Catherine Roussel, Julia Dina, Marie-Anne Trabaud, Helene Le Guillou-Guillemette, Sophie Valet, Anne Signori-Schmuck, Anne Maillard, Anne Krivine, Philippe Flandre, Diane Descamps, Vincent Calvez

**Affiliations:** 1 Virology, Pitie-Salpetriere Hospital, Paris, France; 2 Inserm U1136, Pitie-Salpêtriere Hospital, Paris, France; 3 Virology, Bichat Hospital, Paris, France; 4 Virology, Bordeaux Hospital, Bordeaux, France; 5 Virology, Nantes Hospital, Nantes, France; 6 Virology, Paul Brousse Hospital, Paris, France; 7 Virology, Toulouse Hospital, Paris, France; 8 Virology, Clermont-Ferrand Hospital, Clermond-Ferrand, France; 9 Virology, Lille Hospital, Lille, France; 10 Virology, Saint Antoine, Paris, France; 11 Virology, Saint Louis Hospital, Paris, France; 12 Virology, Montpellier Hospital, Paris, France; 13 Virology, Nancy Hospital, Nancy, France; 14 Virology, Rouen Hospital, Rouen, France; 15 Virology, Strasbourg Hospital, Strasbourg, France; 16 Virology, Tenon Hospital, Paris, France; 17 Virology, Amiens Hospital, Amiens, France; 18 Virology, Caen Hospital, Caen, France; 19 Virology, Lyon Hospital, Lyon, France; 20 Virology, Angers, Angers, France; 21 Virology, Brest Hospital, Brest, France; 22 Virology, Grenoble Hospital, Grenoble, France; 23 Virology, Rennes Hospital, Rennes, France; 24 Virology, Cochin Hospital, Paris, France

## Abstract

**Introduction**: Integrase inhibitors (INIs) are now one of the most important drug classes in clinical practice. With potential for INI cross-resistance, there is a need to get more resistance related data in patients failing an INI-containing regimen in a context of routine hospital care. Doing so with more cases than observed in clinical trials to date would provide a more precise description about the robustness to resistance selection of the three INIs used in clinical practice.

**Materials and methods**: This national survey of resistance to INIs was conducted through the ANRS AC11 virology network: patients who failed to any INI-containing regimens were included to search for selection of resistance to INI and associated factors. Virological failure was defined as two consecutive plasma viral load >50 copies/mL. All the genotypic resistance tests were performed on the second plasma sample with detectable viral load and interpreted following the ANRS V25 algorithm. Patients who failed to RAL and EVG did not fail to any INI before. However, DTG was used either as the first INI or in patients who failed before to RAL or EVG.

**Results**: About 489 patients failing to INI (270 to RAL, 111 to EVG and 96 to DTG)-containing regimen were analyzed (median age 48 years, CD4 398/mm^3^, viral load 3.13 log copies/mL at time of failure). In combination with one INI, 250 (51%) patients received two NRTIs, 34 (7%) one NNRTI, 47 (9%) one PI, 76 (16%) one NRTI + one PI, 19 (4%) one NRTI + one NNRTI, 22 (4%) one NNRTI + one PI and 41 other regimens. Among patients failing to RAL, 32% harboured a virus resistant to RAL and among patients failing to EVG, 40% harboured a virus resistant to EVG. Among patients failing to DTG (used as the first INI or used in patients previously exposed to RAL- or EVG-containing regimen) 19% harboured a virus resistant to DTG. Among the 96 patients failing to DTG, 49 received DTG as the first INI, neither INI resistance mutations among the major pathways (92, 118, 121, 140, 143, 148, 155) nor the R263K mutation were present at failure.

**Conclusions**: In this national survey, RAL and EVG are associated with 32 to 40% resistance at failure. However, in INI-nave patients, failing to DTG when used as the first INI, no resistance to INI was detected whatever the antiretroviral associated to DTG.

# Sub article: O333 Switching from cART to dolutegravir (DTG) maintenance monotherapy in virologically suppressed HIV-1 infected adults: a randomized multicenter, non-inferiority clinical trial (DOMONO)

**Published:** 2016-10-23

**Authors:** Ingeborg Wijting, Casper Rokx, Charles Boucher, Jeroen Van Kampen, Dorine De Vries-Sluijs, Karin Schurink, Hannelore Bax, Maarten Derksen, Elrozy Andrinopoulou, Ineke Van der Ende, Eric Van Gorp, Jan Nouwen, Annelies Verbon, Wouter Bierman, Bart Rijnders

**Affiliations:** 1 Infectious Diseases Internal Medicine, Erasmus MC, Rotterdam, Netherlands; 2 Virology, Erasmus MC, Rotterdam, Netherlands; 3 Department of Biostatistics, Erasmus MC, Rotterdam, Netherlands; 4 Department of Internal Medicine and Infectious Diseases, Universitair Medisch Centrum Groningen, Groningen, The Netherlands

## Abstract

**Introduction**: DTG-containing cART showed equal or superior viral suppression when compared with raltegravir-, efavirenz- or darunavir-containing cART and is one of the preferred regimens in current treatment guidelines. As short- and long-term side effects of cART remain a concern, maintenance HIV therapy with fewer drugs is an attractive goal. Given the high genetic barrier to resistance, DTG is a potential candidate for maintenance monotherapy.

**Materials/methods**: In a randomized, open-label, multicentre study, we compared DTG maintenance monotherapy (50 mg QD=DOLUMONO) with continued cART (con-cART). After 24 weeks, the con-cART patients switched to DOLUMONO as well (‘delayed switch’). Eligible patients were on cART and suppressed (<50 c/mL) for >6 months, had a CD4 nadir >200 cells/µL, HIV RNA zenith <100,000 copies/mL, no history of virologic failure and HBV immune. The primary endpoint was the proportion of patients with virologic suppression at 24 weeks defined as a viral load (VL) <200 copies/mL in the on treatment population. With an anticipated viral suppression of 95% on con-cART, 104 patients were needed to demonstrate non-inferiority of DOLUMONO (delta 0.12, power 80%, alfa=0.025). Predefined secondary endpoints were (1) proportion with a VL <50 copies/mL after 24 weeks of DOLUMONO versus con-cART and (2) proportion with a VL <200 copies/mL and <50 copies/mL after 12, 24 and 48 weeks of DOLUMONO in the entire study population (=immediate + delayed switchers combined). The study was registered as NCT02401828.

**Results**: The 104 patients included were predominantly male (89%), had a median age of 45, a HIV RNA zenith of 21,840 copies/mL (IQR 7045–59,550), CD4 nadir of 345 cells/µL (IQR 270–500) and on cART for 40 months. One patient discontinued DTG at 12 weeks (with VL <50 copies/mL) for disturbed sleep. Of 103, 102 patients had a VL <200 copies/mL at week 24: 98% (49/50) on DOLUMONO and 100% (53/53) on con-cART. DOLUMONO was therefore non-inferior to con-cART (delta 2% with exact 95% CI 12–5%). The single patient on DOLUMONO with virologic failure had a VL at 4 weeks of 70,000 copies/mL despite 100% compliance by pill-count and therapeutic DTG plasma levels of 1.3 mg/L. Integrase sequencing on stored pre-cART plasma and at DTG failure did not reveal resistance-associated mutations. At 24 weeks, more patients on DOLUMONO had low level viraemia (VL of 50–200 copies/mL in 3/49 vs. 0/53, p=0.12). Week 24 results of all 104 patients on DOLUMONO (=immediate and delayed switchers combined) will be presented.

**Conclusions**: In a carefully selected HIV-1 population on suppressive cART, DTG monotherapy was well tolerated and non-inferior to cART. Although these results are promising, longer follow-up is needed as more patients on DOLUMONO had low-level viraemia.

# Sub article: O334 Subgroup analyses from ONCEMRK, a phase 3 study of raltegravir (RAL) 1200 mg once daily versus RAL 400 mg twice daily, in combination with tenofovir/emtricitabine, in treatment-naive HIV-1 infected subjects

**Published:** 2016-10-23

**Authors:** Pedro Cahn, Richard Kaplan, Paul Sax, Kathleen Squires, Jean-Michel Molina, Anchalee Avihingsanon, Winai Ratanasuwan, Evelyn Rojas, Mohammed Rassool, Xia Xu, Anthony Rodgers, Sandy Rawlins, Bach-Yen Nguyen, Randi Leavitt, Hedy Teppler

**Affiliations:** 1 Fundacion Huesped, Juan A. Fernandez Hospital, Buenos Aires, Argentina; 2 Desmond Tutu HIV Foundation, University of Cape Town, Cape Town, South Africa; 3 Brigham & Women's Hospital, Harvard Medical School, Boston, MA, USA; 4 Infectious Diseases, Thomas Jefferson University, Philadelphia, PA, USA; 5 Hopital Saint-Louis, University of Paris Diderot, Paris, France; 6 Medical Department, HIV-NAT Research Collaboration, Bangkok, Thailand; 7 Faculty of Medicine Siriraj Hospital, Mahidol University, Bangkok, Thailand; 8 Infectious Diseases, Cericap Multiclinicas, Guatemala City, Guatemala; 9 University of Witwatersrand, Helen Joseph Hospital, Johannesburg, South Africa; 10 Research, Merck & Co., Inc., Kenilworth, NJ, USA

## Abstract

**Introduction**: In HIV-1-infected treatment-naïve subjects receiving tenofovir/emtricitabine (TDF/FTC), reformulated RAL 1200 mg (two 600-mg tablets) given once daily (QD) demonstrated potent and non-inferior efficacy at week 48 compared to RAL 400 mg given twice daily (BID).

**Methods**: ONCEMRK is a phase 3, multicentre, double-blind, randomized controlled trial comparing reformulated RAL 1200 mg QD to RAL 400 mg BID, both given with TDF/FTC, for up to 96 weeks in treatment-naive HIV-1-infected subjects. Randomization was stratified by screening HIV-1 RNA (vRNA) and chronic hepatitis B/C status. Results for the primary endpoint (% achieving vRNA <40 copies/mL at week 48) and secondary endpoint (change from baseline in CD4 count) were summarized within several pre-specified subgroups in order to further characterize the effect of RAL 1200 mg QD. Summary statistics were calculated by treatment group within each subgroup using the observed failure approach for missing data. The difference between treatment groups and associated 95% CI were also calculated for each subgroup.

**Results**: Baseline demographics were balanced across the two treatment groups. Of 797 treated subjects, 85% were male; racial origin was 59% white, 17% black and 15% Asian; and the mean age was 35.9 years. At baseline, mean CD4 count was 415/mm^3^ and mean plasma vRNA was 4.6 log_10_ copies/mL; 28% of subjects had baseline vRNA >100,000 copies/mL, 3% had hepatitis B and/or C co-infection and 34% had non-B subtype HIV infection. Results for the primary efficacy endpoint across selected baseline demographic and prognostic factors are shown below (Table 1). The change in CD4 counts was also similar across subgroups (results not shown).

**Conclusions**: Reformulated RAL 1200 mg QD demonstrated potent and consistent virologic and immunologic efficacy across demographic and baseline prognostic factors, including baseline plasma vRNA level >100,000 copies/mL, baseline CD4 count =200 cells/mm^3^, hepatitis co-infection, gender, viral subtype and geographic region.

**Abstract O334–Table 1:** Proportion of subjects with HIV-1 RNA <40 copies/mL at week 48 by prognostic and demographic factors (observed failure approach)

Virologic response by subgroup					

	RAL 1200 mg QD		RAL 400 mg BID		Difference (QD - BID)
			
	N	% <40 c/mL	N	% <40 c/mL	% (95% CI)^a^
All subjects	501	94.2	251	93.6	0.6 (−2.8–4.7)
Baseline plasma HIV RNA (copies/mL)					
≤100,000 copies/mL	358	97.2	177	97.7	−0.5 (−3.2–3.1)
>100,000 copies/mL	143	86.7	74	83.8	2.9 (−6.5–14.1)
Baseline plasma HIV RNA (copies/mL)					
≤500,000 copies/mL	479	95.2	237	95.8	−0.6 (−3.6–3.1)
>500,000 copies/mL	22	72.7	14	57.1	15.6 (−15.6–45.9)
Baseline CD4 cell counts (cells/mm3)					
≤200 cells/mm3	67	85.1	33	87.9	−2.8 (−16.0–14.0)
>200 cells/mm3	434	95.6	218	94.5	1.1 (−2.2–5.3)
Hepatitis co-infection^b^					
Hepatitis B or C positive	13	100	7	85.7	14.3 (−11.7–52.2)
Hepatitis B and C negative	488	94.1	244	93.9	0.2 (−3.3–4.3)
Gender					
Male	415	94.7	220	93.6	1.1 (−2.6–5.5)
Female	86	91.9	31	93.5	−1.7 (−11.1–13.3)
Viral subtype					
Clade B	313	94.6	175	93.7	0.9 (−3.3–5.9)
Non-clade B	187	93.6	74	93.2	0.3 (−5.7–8.9)
Geographic region					
Africa	41	95.1	12	100	−4.9 (−16.3–20.0)
Asia/Pacific	84	94.0	43	93.0	1.0 (−7.7–13.3)
Europe	190	96.3	108	94.4	1.9 (−2.9–8.2)
Latin America	73	94.5	26	100	−5.5 (−13.3–7.7)
North America	113	90.3	62	88.7	1.6 (−7.5–12.7)

a 95% CIs by Miettinen and Nurminen's method;

b Hepatitis B surface antigen and/or HCV RNA by PCR.

N=number in subgroup.

# Sub article: O335A HIV-1 attachment inhibitor prodrug BMS-663068 in antiretroviral-experienced subjects: week 96 subgroup analysis

**Published:** 2016-10-23

**Authors:** Enrique Rafael Granados-Reyes, Louis Sloan, Jerome Ernst, Mey León, David Stock, Cyril Llamoso, Samit Joshi, George Hanna, Max Lataillade

**Affiliations:** 1 Centro de Investigación Clinica Gramel SC, Mexico City, Mexico; 2 Research Department, North Texas Infectious Diseases Consultants, P.A., Dallas, TX, USA; 3 AIDS Community Research Initiative of America, New York, NY, USA; 4 Infectious and Tropical Diseases, Asociacion Civil Impacta Salud y Educacion, Lima, Peru; 5 Research and Development, Bristol-Myers Squibb, Wallingford, CT, USA; 6 Research and Development, ViiV Healthcare, Wallingford, CT, USA; 7 Research and Development, Bristol-Myers Squibb, Princeton, NJ, USA

## Abstract

**Introduction**: BMS-663068 is a prodrug of BMS-626529, an attachment inhibitor that binds directly to HIV-1 gp120, preventing initial viral attachment and entry into the host CD4+ T-cell. AI438011 is an ongoing, phase IIb, randomized, active-controlled trial investigating the safety, efficacy and dose-response of BMS-663068 versus atazanavir/ritonavir (ATV/r) in treatment-experienced, HIV-1-positive subjects. Through week 96, BMS-663068 showed generally similar efficacy to ATV/r. We report a subgroup analysis of viral efficacy and immunologic response through week 96.

**Materials and methods**: Treatment-experienced subjects (exposure to ≥1 antiretroviral for ≥1 week) with susceptibility to all study drugs and BMS-626529 IC_50_ <100 nM were randomized to four BMS-663068 arms (400 or 800 mg BID; 600 or 1200 mg QD) or control arm (ATV/r 300/100 mg QD), with tenofovir disoproxil fumarate 300 mg (QD)+raltegravir 400 mg (BID). Pooled data for BMS-663068 are presented as BMS-663068 1200 mg QD was selected as the open-label continuation dose after week 48. Efficacy (observed data) was evaluated following stratification by age, gender, race, baseline viral load (VL) and baseline CD4+ T-cell count. The study was not powered to detect statistically significant differences between subgroups.

**Results**: A total of 251 subjects were treated (BMS-663068: 200; ATV/r: 51). Median age was 39 years, 60% were male and 38% white. Median baseline VL was 4.85 log_10_ copies/mL (43% >100,000 copies/mL) and median CD4+ T-cell count 230 cells/mm^3^ (38% <200 CD4 cells/mm^3^). At week 96, response rates (HIV-1 RNA <50 copies/mL) were generally similar between BMS-663068 and ATV/r arms regardless of gender, age, race, baseline VL (<100,000 copies/mL vs. ≥100,000 copies/mL) and baseline CD4+ T-cell count (<200 cells/mm^3^ vs. ≥200 cells/mm^3^) (Table 1). Mean increases in CD4+ T-cell count from baseline to week 96 were similar among BMS-663068 arms regardless of age, gender, race and baseline CD4+ T-cell count. Increase in CD4+ T-cell counts appeared greater for subjects with a baseline VL ≥100,000 copies/mL versus <100,000 copies/mL across BMS-663068 and ATV/r arms (Table 2).

**Conclusions**: At week 96, virologic response was generally similar for BMS-663068 and ATV/r in treatment-experienced subjects, regardless of gender, age, race, baseline VL or baseline CD4+ T-cell count. Mean increases in CD4+ T-cell count from baseline to week 96 were similar for BMS-663068 regardless of age, gender, race and baseline CD4+ T-cell count. These results are mostly consistent with those reported at week 48. A phase III trial is underway to evaluate BMS-663068 for use in heavily treatment-experienced adults with limited therapeutic options (NCT02362503).

**Abstract O335A–Table 1:** Response rates (HIV-1 RNA <50 copies/mL) at week 96 by subgroup (observed)

	BMS-663068 pooled^a^ (N=136) No. of responders (%)		ATV/r (N=30) No. of responders (%)
**Subgroups**		**Subgroups**	
**Age**		**Age**	
<40 years (n=65)	60 (92.3)	<40 years (n=14)	12 (85.7)
≥40 years (n=71)	62 (87.3)	≥40 years (n=16)	15 (93.8)
**Gender**		**Gender**	
Male (n=78)	69 (88.5)	Male (n=17)	16 (94.1)
Female (n=58)	53 (91.4)	Female (n=13)	11 (84.6)
**Race**		**Race**	
Black (n=40)	37 (92.5)	Black (n=8)	7 (87.5)
White (n=54)	49 (90.7)	White (n=12)	11 (91.7)
Other (n=42)	36 (85.7)	Other (n=10)	9 (90.0)
**Baseline viral load**		**Baseline viral load**	
<100,000 copies/mL (n=84)	73 (86.9)	<100,000 copies/mL (n=20)	19 (95.0)
≥100,000 copies/mL (n=52)	49 (94.2)	≥100,000 copies/mL (n=10)	8 (80.0)
**Baseline CD4+ T-cell counts**^b^		**Baseline CD4+ T-cell counts**	
<200 cells/mm^3^ (n=42)	37 (88.1)	<200 cells/mm^3^ (n=13)	12 (92.3)
≥200 cells/mm^3^ (n=93)	85 (91.4)	≥200 cells/mm^3^ (n=17)	15 (88.2)

a Pooled data are presented because BMS-663068 1200 mg QD was selected as the open-label continuation dose after week 48

b One subject did not have a baseline CD4 value.

**Abstract O335A–Table 2:** Mean increases in CD4+ T-cell count from baseline to week 96 by subgroup (observed)^a^

	BMS-663068 pooled^b^ (N=134) cells/mm^3^		ATV/r (N=31) cells/mm^3^
**Subgroups**		**Subgroups**	
**Age**		**Age**	
<40 years (n=64)	215.5	<40 years (n=15)	244.0
≥40 years (n=69)	221.8	≥40 years (n=16)	255.8
**Gender**		**Gender**	
Male (n=75)	210.1	Male (n=18)	151.9
Female (n=58)	230.0	Female (n=13)	386.0
**Race**		**Race**	
Black (n=38)	213.9	Black (n=8)	415.4
White (n=54)	231.0	White (n=13)	259.8
Other (n=41)	207.2	Other (n=10)	105.2
**Baseline viral load**		**Baseline viral load**	
<100,000 copies/mL (n=81)	184.2	<100,000 copies/mL (n=21)	208.0
≥100,000 copies/mL (n=52)	272.6	≥100,000 copies/mL (n=10)	338.4
**Baseline CD4+ T-cell counts**		**Baseline CD4+ T-cell counts**	
<200 cells/mm^3^ (n=42)	202.5	<200 cells/mm^3^ (n=13)	216.4
≥200 cells/mm^3^ (n=91)	226.2	≥200 cells/mm^3^ (n=18)	274.4

a One subject did not have a baseline CD4 value

b Pooled data are presented because BMS-663068 1200 mg QD was selected as the open-label continuation dose after week 48.

# Sub article: O335B HIV-1 attachment inhibitor prodrug BMS-663068 in antiretroviral-experienced subjects: week 96 safety analysis

**Published:** 2016-10-23

**Authors:** Cyril Llamoso, Johannes Bogner, Larissa Afonina, Mey León, Alexey Yakovlev, David Stock, Samit Joshi, George Hanna, Max Lataillade

**Affiliations:** 1 Research and Development, ViiV Healthcare, Wallingford, CT, USA; 2 Section for Infectious Diseases, Med. IV, Hospital of the University of Munich, Munich, Germany; 3 Clinical Research and Medical Information, Republic Hospital of Infectious Diseases, St Petersburg, Russian Federation; 4 Infectious and Tropical Diseases, Asociacion Civil Impacta Salud y Educacion, Lima, Peru; 5 Department of Infectious Diseases, St Petersburg Botkin Clinical Infectious Diseases Hospital, St Petersburg, Russian Federation; 6 Research and Development, Bristol-Myers Squibb, Wallingford, CT, USA; 7 Research and Development, Bristol-Myers Squibb, Princeton, NJ, USA

## Abstract

**Introduction**: BMS-663068 is a prodrug of BMS-626529, an attachment inhibitor that binds directly to HIV-1 gp120, preventing initial viral attachment and entry into host CD4+ T-cells. AI438011 is an ongoing, phase IIb, randomized, active-controlled trial investigating the safety, efficacy and dose-response of BMS-663068 versus atazanavir/ritonavir (ATV/r) in treatment-experienced, HIV-1-positive subjects. Through week 96, BMS-663068 showed generally similar efficacy to ATV/r. We report the complete safety profile through week 96.

**Materials and methods**: Treatment-experienced subjects (exposure to ≥1 antiretroviral for ≥1 week) with susceptibility to all study drugs and BMS-626529 IC_50_ <100 nM were randomized to four BMS-663068 arms (400 or 800 mg BID; 600 or 1200 mg QD) or a control arm (ATV/r 300/100 mg QD), with tenofovir disoproxil fumarate (TDF) 300 mg (QD)+raltegravir (RAL) 400 mg (BID). Pooled safety data for BMS-663068 are presented because BMS-663068 1200 mg QD was selected as the open-label continuation dose after week 48.

**Results**: A total of 251 subjects received treatment (BMS-663068: 200; ATV/r: 51). No BMS-663068-related adverse events (AEs) led to discontinuation (Table 1). Grade 2–4 drug-related AEs occurred in 17/200 (8.5%) BMS-663068 treated subjects; these were all single instances. In the ATV/r arm, grade 2–4 drug-related AEs occurred in 19/51 (37%) subjects: most were attributable to gastrointestinal and/or hepatobiliary disorders. Across BMS-663068 arms no trends for grade 3–4 laboratory abnormalities were observed. Serious AEs (SAEs) occurred in 24/200 (12%) and 7/51 (14%) subjects receiving BMS-663068 and ATV/r, respectively; most were attributable to infections (BMS-663068: nine [5%]; ATV/r: three [6%]). One unrelated death occurred (gunshot wound). The most common AE reported for BMS-663068 was grade 1–4 transient headache (32/200, 16%), which was reported in 5/51 (10%) subjects for ATV/r.

**Conclusions**: BMS-663068 was generally well tolerated, with no BMS-663068-related AEs leading to discontinuation, and no new safety signals from the previously described safety profile. No trends were observed for grade 2–4 AEs or clinical laboratory abnormalities. These results support the ongoing phase III trial evaluating BMS-663068 for use in heavily treatment-experienced adults with limited therapeutic options (NCT02362503).

**Abstract O335B–Table 1:** Week 96 pooled safety results

Parameter, n (%)	BMS-663068^a^+TDF (300 mg QD)+RAL (400 mg BID) N=200	ATV/r (300/100 mg QD)+TDF (300 mg QD)+RAL (400 mg BID) N=51
Subjects with ≥1 AE (grade 1–4)	181 (91)	50 (98)
Related grade 1–4 AEs	64 (32)	28 (55)
Related grade 2–4 AEs	17 (8.5)	19 (37)
SAEs	24 (12)	7 (14)
AEs leading to discontinuation^b^	5 (2.5)	5 (10)
Deaths^b^	1 (0.5)	0

a Pooled data are presented because BMS-663068 1200 mg QD was selected as the open-label continuation dose after week 48;

b None were related to BMS-663068.

# Sub article: O336 Efficacy and safety of long-acting HIV fusion inhibitor albuvirtide in antiretroviral-experienced adults with HIV-1: interim 48-week results from the randomized, controlled, phase 3, non-inferiority TALENT study

**Published:** 2016-10-23

**Authors:** Hao Wu, Cheng Yao, Tong Zhang, Qingxia Zhao, Weiping Cai, Min Wang, Hongzhou Lu, Hui Wang, Yuhuang Zheng, Biao Zhu, Jianhua Yu, Yongtao Sun, Min Zhao, Wenhui Lun, Wei Xia, Qingshan Zheng, Haiyan Peng, Rongjian Lu, Jianhua Hu, Hui Xing, Yiming Shao, Meixia Wang, Dong Xie

**Affiliations:** 1 Infectious Diseases, Beijing You'an Hospital, Capital Medical University, Beijing, China; 2 Clinical Development, Frontier Biotechnologies Co., Nanjing, China; 3 AIDS, Infectious Disease Hospital of Henan Province, Zhengzhou, China; 4 Infectious Diseases, The Eighth People's Hospital of Guangzhou, Guangzhou, China; 5 AIDS, The First Hospital of Changsha, Changsha, China; 6 Infectious Diseases, Shanghai Public Health Clinical Center, Shanghai, China; 7 AIDS, The Third People's Hospital of Shenzhen, Shenzhen, China; 8 AIDS, The Second Xiangya Hospital of Central South University, Changsha, China; 9 Infectious Diseases, The First Affiliated Hospital, Zhejiang University, Hangzhou, China; 10 AIDS, Xixi Hospital of Hangzhou, Hangzhou, China; 1 Infectious Diseases, Tangdu Hospital of The Fourth Military Medical University, Xian, China; 2 AIDS, 302 Military Hospital of China, Beijing, China; 3 AIDS, Beijing Ditan Hospital, Capital Medical University, Beijing, China; 4 Center for Drug Clinical Research, Shanghai University of Traditional Chinese Medicine, Shanghai, China; 5 Clinical Operation, Beijing Co-CRO Medical Development Co., Ltd, Beijing, China; 6 Research and Development, Frontier Biotechnologies Co., Nanjing, China; 7 Virology, National Center for AIDS/STD Control and Prevention, Chinese Center for Disease Control and Prevention, Beijing, China; 8 Clinical Research, Beijing You'an Hospital, Capital Medical University, Beijing, China; 9 Research and Development, Frontier Biotechnologies Co., Nanjing, China

## Abstract

**Introduction**: Albuvirtide is a once-weekly injectable HIV-1 fusion inhibitor. We present interim data of the TALENT study (ClinicalTrials.gov, NCT02369965), that assessed the safety and efficacy of albuvirtide plus lopinavir-ritonavir in antiretroviral-experienced adults with HIV-1.

**Materials and methods**: We carried out the 48-week, phase 3, randomized, controlled, open-label non-inferiority trial at 12 sites in China. Adults on WHO-recommended first-line treatment for >6 months with plasma viral load >1000 copies/mL were enrolled and randomly assigned (1:1) to receive albuvirtide (once weekly) plus ritonavir-boosted lopinavir (albuvirtide) or WHO-recommended second-line treatment (control). The primary endpoint was the proportion of patients with plasma viral load <50 copies/mL at 48 weeks. Non-inferiority was pre-specified with a margin of 12%.

**Results**: At the time of analysis, 1185 patients were screened and 372 were enrolled. For the modified intention-to-treat population, 24 weeks data were available for 83 and 92 patients, and 48 weeks for 46 and 50 patients in the albuvirtide and control groups respectively. At 48 weeks, 80.4% patients in the albuvirtide group had HIV-1 RNA <50 copies/mL versus 66.0% in the control group (difference 14.4%, 95% CI −3.0%–31.9%), meeting the non-inferiority criteria. For the per-protocol population, superiority of albuvirtide to control was demonstrated (94.9% vs. 74.4%, difference 20.5%, 95% CI 5.7–35.2, p=0.01). Grade 3–4 adverse event frequencies were similar across groups (14.0% vs. 11.1%); the most common adverse events for albuvirtide versus control were diarrhoea (8.6% vs. 14.1%), upper respiratory tract infection (4.3% vs. 6.1%), grade 3-4 elevated triglycerides (6.5% vs. 4.0%). Renal function impairment was significantly less at 12 weeks in patients of the albuvirtide group compared with those of the control group who received TDF (mean change in eGFR −11.47 vs. −1.22 mL/min/1.73 m^2^, p=0.02).

**Conclusions**: TALENT study is the first phase 3 trial of an injectable long-acting HIV drug. This interim analysis suggests that once-weekly albuvirtide in combination with ritonavir-boosted lopinavir is clinically practical, well tolerated and non-inferior to WHO-recommended second-line regimen in patients failed first-line treatment. Analysis is scheduled when all enrolled patients complete 48 weeks treatment to confirm these data.

# Sub article: P001 High prevalence of hepatitis C co-infection and adverse pregnancy outcomes among HIV-infected pregnant women in Switzerland

**Published:** 2016-10-23

**Authors:** Karoline Aebi-Popp, Tracy Glass, Christoph Rudin, Begoña Martinez de Tejada, Irene Hoesli, Claudia Grawe, Tina Fischer, Andrea Duppenthaler, Andri Rauch, Christian Kahlert

**Affiliations:** 1 Infectious Diseases, University Hospital Bern, Bern, Switzerland; 2 Department of Epidemiology and Public Health, Swiss Tropical and Public Health Institute, Basel, Switzerland; 3 Nephrology, University Children's Hospital, Basel, Switzerland; 4 Obstetrics and Gynecology, University Hospital Geneva, Geneva, Switzerland; 5 Obstetrics and Gynecology, University Hospital Basel, Basel, Switzerland; 6 Obstetrics and Gynecology, University Hospital Zurich, Zurich, Switzerland; 7 Obstetrics and Gynecology, Cantonal Hospital St. Gallen, St.Gallen, Switzerland; 8 Infectious Diseases, University Children's Hospital Bern, Bern, Switzerland; 9 Pediatric Infectious Diseases, Cantonal Hospital St. Gallen, St. Gallen, Switzerland

## Abstract

**Introduction**: Hepatitis C virus (HCV) infection is more prevalent in HIV-infected compared to HIV-negative women. Limited data on pregnancy outcomes in HIV/HCV co-infected women are available. This study aimed to investigate prevalence, immunological parameters and pregnancy outcomes associated with HCV-HIV co-infected pregnant women.

**Methods**: Prospectively collected data of the Swiss Mother and Child HIV Cohort Study including pregnancies and deliveries documented between 2000 and 2015 were analysed. Hepatitis C co-infection was defined as HCV antibody positivity. Multiple pregnancies were excluded.

**Results**: Of 548 women, 75 (13.7%) were HCV seropositive. Compared with HCV seronegative women those with HIV/HCV co-infection reported more often a history of injecting drug use (IDU) (65.3% vs. 1.5%, p<0.001), were older at conception (>31 years: 74.7% vs. 56.9%, p<0.001) and more were Caucasian (90.7% vs. 30.2%, p<0.001). More co-infected women were already on antiretroviral therapy (ART) at time of conception (80.8% vs. 55.3%, p<0.001), had more ART switches during pregnancy, but did not differ in terms of first CD4 cell count during pregnancy or viral suppression (<400 copies/µL) at time of delivery (92.6% and 92.9% respectively). Strikingly, HIV/HCV co-infected women were twice as likely to deliver preterm (<37 weeks) compared to HIV mono-infected women (28% vs. 16.5%, OR 2.0, 95% CI 1.14–3.50, p=0.015). Older age, cigarette smoking and a history of IDU were associated with preterm delivery in univariable analysis. After adjustment for those factors and for duration of ART, HIV/HCV co-infected women still had a higher odds of preterm delivery, but the result was no longer statistically significant (aOR 1.60, 95% CI 0.59–4.33, p=0.36). We found more newborns of HIV/HCV-infected mothers with birth weight below 2500 g (63.4% vs. 36.6%, p<0.001) and any development delay at 6 months of age (aOR 6.07, 95% CI 1.34–27.38, p=0.019). Regarding vertical transmission, 7/526 (1.3%) babies were HIV infected (none of these from HIV/HCV co-infected mothers), and 2/71 (2.8%) were HCV infected.

**Conclusion**: We found a high HCV co-infection rate in HIV-infected pregnant women in Switzerland. Women with HCV were more likely to have an adverse pregnancy outcome in terms of preterm delivery and lower birth weight of the newborn. Treatment of hepatitis C in HIV co-infected women at childbearing age before pregnancy should be evaluated to avoid adverse pregnancy outcomes including vertical transmission of HCV.

# Sub article: P002 A mother-to-child HIV-1 transmission bottleneck? A new understanding of the selection biases underlying successful perinatal infection

**Published:** 2016-10-23

**Authors:** Christie Noble, Emily Adland, Vanessa Naidoo, Thumbi Ndung'u, Philip Goulder

**Affiliations:** 1 Department of Paediatrics, University of Oxford, UK; 2 Doris Duke Medical Research Institute, University of KwaZulu-Natal, Durban, South Africa

## Abstract

**Introduction**: Heterosexual HIV-1 transmission has been described as a severe genetic bottleneck, with a stringent selection bias favouring the transmission of a high fitness viral variant [1]. Recent work has also suggested that the selection bottleneck present during transmission in men who have sex with men (MSM) has notable differences [2]. There is poorer understanding of the mechanisms underlying successful mother-to-child transmission, where rates of transmission are much higher. In this study, we tested the hypothesis that a similar bottleneck is seen in mother-to-child transmission, and that a viral variant with high viral replicative capacity (VRC) is responsible for establishing infection in the child.

**Materials and methods**: Thirty-eight mother-child HIV transmission pairs were included in the study (26 children were infected by intra-uterine [IU] transmission and 12 by intra-partum [IP] transmission). These pairs were taken from a previously described, treatment-naïve cohort recruited in Durban, South Africa, between 2003 and 2005 [3]. Samples were taken from the mothers antenatally, within 4 months of delivery, and from the child up to 14 weeks after birth. Authenticity of the mother-child transmission pairs was validated by phylogenetic analysis of the viral sequences. Gag-protease chimeric viruses were generated from mother and child plasma samples and used in a VRC assay as previously described [4].

**Results**: The VRC of chimeric viruses derived from the children was compared with those of their respective mothers. In an unexpected finding, we show that, overall, the VRC in the children was lower than in the mothers (mean VRC 0.70. vs 0.80; p<0.0001). Although this difference was noted for both IU and IP transmission, it was more notable in the pairs where IU transmission had taken place (IP: mean VRC difference −0.06, p=0.027; IU: mean VRC difference −0.12, p=0.0002). Mother and child VRCs were very strongly correlated (r=0.760, p<0.0001). Interestingly, we also observed that female children tended to have lower VRCs than male children, although this did not reach significance.

**Conclusions**: Future treatment interventions will require a detailed understanding of the mechanisms involved in successful mother-to-child HIV transmission. Here, we present the new and surprising finding that, overall, VRC in children is significantly lower than in their mothers. This finding is the opposite of what is seen during horizontal HIV transmission. It offers a new understanding of the selection pressures involved in mother-to-child transmission and will potentially indicate new therapeutic approaches.

## References

[1] Carlson JM, Schaefer M, Monaco DC, Batorsky R, Claiborne DT, Prince J (2014). HIV transmission. Selection bias at the heterosexual HIV-1 transmission bottleneck. Science.

[2] Tully DC, Ogilvie CB, Batorsky RE, Bean DJ, Power KA, Ghebremichael M (2016). Differences in the selection bottleneck between modes of sexual transmission influence the genetic composition of the HIV-1 founder virus. PLoS Pathog.

[3] Prendergast A, Mphatswe W, Tudor-Williams G, Rakgotho M, Pillay V, Thobakgale C (2008). Early virological suppression with three-class antiretroviral therapy in HIV-infected African infants. AIDS.

[4] Adland E, Paioni P, Thobakgale C, Laker L, Mori L, Muenchhoff M (2015). Discordant impact of HLA on viral replicative capacity and disease progression in paediatric and adult HIV infection. PLoS Pathog.

# Sub article: P003 Raltegravir in HIV-1-infected pregnant women: MTCT prophylaxis and children safety case series

**Published:** 2016-10-23

**Authors:** Melissa Soares Medeiros, Henrique Pires Moreira, Erico Antonio Gomes Arruda, Eduardo Austregesilo Correa, Yasmin Camelo de Sales, Gisele Facanha Diogenes Teixeira, Ederson Aragao Ribeiro, Raquel Silveira Dantas

**Affiliations:** 1 Medicine/Infectious Diseases, Hospital Sao Jose/Unichristus University, Fortaleza, Brazil; 2 Infectious Diseases, Unichristus University, Fortaleza, Brazil; 3 Infectious Diseases, Hospital Sao Jose/Ceara State University (UECE), Fortaleza, Brazil

## Abstract

**Introduction**: Antiretroviral therapy reduced perinatal transmission for less than 1% in several countries, but mother-to-child transmission (MTCT) was not eliminated [1]. Raltegravir (RAL) has demonstrated good tolerability during pregnancy in previous studies [2] and could be beneficial to HIV mothers detected in late pregnancy without prenatal care and demanding rapid viral load control, in association regimens for HIV mothers failing therapy during pregnancy or resistant virus [3]. In developing countries, where HIV late detection in pregnancy is still a serious problem, associate RAL in third trimester could be a valid prevention approach to MTCT.

**Materials and methods**: Seven women were followed in Hospital Geral de Fortaleza between 2014 and 2016. They received RAL 400 mg twice daily. Four were HIV late detected in third trimester; two had previous diagnosis with antiretroviral exposes and viral load higher than 1000 copies; and one presented viral resistance. Mothers were followed during pregnancy and children after birth for 6 months.

**Results**: Mean age was 26.3 years (var 20–36). Previous therapy exposure: naïve (four), TDF/3TC/EFZ (one), TDF/3TC/NVP (one) and ZDV/3TC/ATV 300 mg/r 100 mg (one). Mean weeks of pregnancy at RAL initiation 32.3 (var 21–37). Associated medications in regimens with RAL: ZDV/3TC/LPVr (four), TDF/3TC/LPVr (two) and TDF/3TC/NVP (one). Mean CD4 before RAL 486 cells/mm^3^, and at birth time 616 cells/mm^3^. Mean CD8 before RAL 884 cells/mm^3^, and at birth time 1015 cells/mm^3^. Five patients had undetectable viral load at birth, one had 45 copies/mL and one had 2662 copies/mL. Mean falling viral load was 3.56 log. One patient documented viral load falls of 3.8 log in 21 days, another 3.08 log in 7 days and one with 2.2 log in 23 days. Mothers tolerated regimen with RAL without adverse effects. Six children were not infected, and all seven did not present malformation. One child was infected, her mother initiated ZDV/3TC/LPVr/RAL just 5 days prior to birth (viral load 2662). Child presented viral load after birth of 308,718 (2 months) and 386,152 (3 months), and genotypic test HIV subtype B, 211K mutation in reverse transcriptase and 41K, 63P, 71V, 77I, 93L in protease. These results suggest intrauterine transmission and transmitted resistance mutations in HIV.

**Conclusions**: We detected rapid viral load falls during pregnancy with RAL in standard doses, good tolerability in women and safety in children. Alert for previous transmitted resistance in mother-to-child prophylaxis, suggesting importance of genotypic test in pregnancy and early HIV diagnosis.

## References

[1] Trahan M-J, Lamarre V, Metras M-E, Lapointe N, Kakkar F Raltegravir for prevention of mother-to-child transmission of HIV. [Abstract TUAB0105.].

[2] Westling K, Pettersson K, Kaldma A, Navér L (2012). Rapid decline in HIV viral load when introducing raltegravir-containing antiretroviral treatment late in pregnancy. Aids Patient Care STDs.

[3] Blonk MI, Colbers AP, Hidalgo-Tenorio C, Kabeya K, Weizsäcker K, Haberl AE (2015). Raltegravir in HIV-1-infected pregnant women: pharmacokinetics, safety, and efficacy. Clin Infect Dis.

# Sub article: P004 Prophylactic antiretroviral treatment in new-born infants from HIV-positive mothers in 2012 to 2015, for the North-Eastern part of Romania

**Published:** 2016-10-23

**Authors:** Carmen Manciuc, Andrei Vata, Cristina Nicolau, Alexandra Largu

**Affiliations:** 1 Infectious Diseases, Gr. T. Popa University of Medicine and Pharmacy, Iasi, Romania; 2 HIV/AIDS, Sf. Parascheva Infectious Diseases Clinical Hospital, Iasi, Romania; 3 Behavioral Sciences, Gr. T. Popa University of Medicine and Pharmacy, Iasi, Romania

## Abstract

**Introduction**: In the North-Eastern region of Romania most of the female HIV-positive population is sexually active and at child-bearing age. In Romania, there is a strict protocol regarding HIV vertical transmission [1]. We aim to evaluate the efficiency of this protocol and the degree of appliance, reflected in the HIV status of new-born infants from HIV-positive mothers for a period of 3 years.

**Materials and methods**: Of the 1424 patients actively monitored in the HIV/AIDS Regional Center in Iasi, Romania, 46.5% (663) are female. We evaluated retrospectively the files of all new-born infants from HIV-positive mothers for a period of 4 years (January 2012–December 2015).

**Results**: In the period mentioned above, 127 children were born (36 in 2012, 38 in 2013, 26 in 2014, 27 in 2015); one death occurred 10 days after birth, due to multiple organ malformations; the lowest weight at birth was 750 g; three of the children (3%) had a detectable viral load at birth; in two cases we could not evaluate the viral load; 125 children (98%) were born through caesarean section; two were born through natural labour (2%), one of which at home. Mothers received treatment with lopinavir/ritonavir + zidovudine/lamivudine through the whole pregnancy in 81 cases, other antiretroviral regimens in 27 cases, and in nine cases the mothers did not receive any treatment, being tested for HIV at birth. For all new-borns prophylaxis was made with zidovudine+lamivudine for 6 weeks. Three children remained positive at 18 months, and therapy for them consisted of zidovudine+lamivudine+nevirapine.

**Conclusions**: Evaluation of pregnant HIV-positive women and prophylaxis for new-born infants in the evaluated period was conducted according to protocols, which resulted in a small percentage of HIV-positive children (2.3%) [2,3].

## References

[1] Ambia J, Mandala J (2016). A systematic review of interventions to improve prevention of mother-to-child HIV transmission service delivery and promote retention. J Int AIDS Soc.

[2] Buzdugan R, Dufour MK, Mccoy SI, Watadzaushe C, Dirawo J, Mushavi A (2016). Option A improved HIV-free infant survival and mother to child HIV transmission at 9–18 months in Zimbabwe. AIDS.

[3] Livingston EG, Huo Y, Patel K, Tuomala RE, Scott GB, Stek A (2016). Complications and route of delivery in a large cohort study of HIV-1-infected Women- IMPAACT P1025. J Acquir Immune Defic Syndr.

# Sub article: P005 Determinants and risk of HIV infection among HIV-exposed infants in western Ethiopia

**Published:** 2016-10-23

**Authors:** Eyasu Ejeta Duken, Dabsu Regea, Obsa Siyum

**Affiliations:** 1 Medical Laboratory Sciences and Pathology, Jimma University, Jimma, Ethiopia; 2 Medical Laboratory Sciences, Wollega University, Nekemte, Ethiopia

## Abstract

**Introduction**: Ethiopia is one of the sub-Saharan African countries hard-hit by HIV/AIDS and unfortunately, one of every three children born to those women gets infected with HIV in the country. Monitoring and evaluation of the rate and its risk factors for HIV infection among infants born to HIV-positive mothers are among the major indicators of the performance of a national HIV control program. However, this is not well documented in Oromia Regional State of Ethiopia which is the first largest, most populous and one of the hardest HIV/AIDS-hit regions in the country. Hence, this institutional-based retrospective study was conducted in 43 health facilities from November 2014 to January 2015 in selected administrative zones of western Oromia, Ethiopia. The study participants were HIV-exposed infants enrolled between June 2012 and October 2014 in the institution.

**Method**: Medical records of HIV-exposed infants and their mothers enrolled into the program were reviewed to collect the data.

**Results**: A total of 492 HIV-exposed infants having HIV DNA/PCR test result were included in the study. The overall prevalence of HIV among HIV-exposed infants was 7.70%.

**Conclusion**: Failure to receive either antiretroviral therapy or prophylaxis for more than 4 months (AOR 4.2, 95% CI 1.4–12.6), not receiving co-trimoxazole preventive therapy (AOR 7.8, 95% CI 2.6–23.7), failure to receive prophylaxis at birth (AOR 18.1, 95% CI 5.2–63.4) and mixed feeding (AOR 2.302, 95% CI 1.167–4.539) were the factors that increase the risk of mother-to-child transmission of HIV. In conclusion, the risk of HIV infection among infants born to HIV-infected mothers is high in the study area. Therefore, education and promotion for seeking obstetric care and HIV services during their course of pregnancy, focusing on exclusive breast feeding counselling and promotion, and early initiation of antiretroviral treatment to HIV-infected pregnant women are recommended to curb the devastating consequences of HIV on pregnant women and their newborns.

# Sub article: P006 Preventing mother-to-child transmission of HIV in a general hospital: are we following the guidelines?

**Published:** 2016-10-23

**Authors:** Funmi Awosusi, Maha Al-Harbi

**Affiliations:** 1 Pharmacy, Barts Health NHS Trust, London, UK

## Abstract

**Introduction**: Mother-to-child transmission of HIV has declined to less than 2% in the UK [1,2]. This decline was a result of the development in perinatal care and preventive measures offered to HIV-positive pregnant women [3,4]. In the UK, management of HIV-infected pregnant women is according to the British HIV Association (BHIVA) pregnancy guidelines, recently reviewed in 2014. The aim of the audit was to measure the extent of adherence to the BHIVA pregnancy guidelines.

**Methods**: A total of 117 women were identified as HIV-positive pregnant women registered to deliver at the hospital, between May 2012 and December 2015. A total of seven maternal and neonatal factors were evaluated to measure extent of adherence to the guidelines. Data were collected retrospectively from the hospital database and medical notes of these women and their neonates. Eleven women had miscarriages, ten delivered in other different hospitals and one infant died at birth. These were excluded from analysis, leaving a total of 95 patients. Data were compiled onto a spreadsheet and analyzed.

**Results**: Adherence to the guidelines varied in all the aspects examined. Areas where less optimal adherence were observed included viral load testing at delivery for women taking HAART and HIV polymerase chain reaction (PCR) testing for neonates at 12 weeks. Majority of women were undetectable at 36-week gestation (80%), but only 60% delivered vaginally, even though appropriate mode of delivery was planned for the patients at 36 weeks. For neonatal antiretroviral, 55% were documented to have received zidovudine monotherapy or triple therapy, as appropriate, within 4 hours.

**Conclusion**: There are variations in adherence of the care provided at the hospital, to the BHIVA guidelines. Areas for development include improvement in documentation, maternal viral load testing at delivery and neonatal HIV PCR testing at 12 weeks.

## References

[1] Agmon-Levin N, Elbirt D, Asher I, Torten D, Cohen Y, Gradestein S (2009). Prevention of human immunodeficiency virus mother-to-child transmission in Israel. Int J STD AIDS.

[2] Taylor G, Clayden P, Dhar J, Gandhi K, Gilleece Y, Harding K (2012). British HIV Association guidelines for the management of HIV infection in pregnant women 2012. HIV Med.

[3] Kouanda S, Tougri H, Cissé M, Simpore J, Pietra V, Doulougou B (2010). Impact of maternal HAART on the prevention of mother-to-child transmission of HIV: results of an 18-month follow-up study in Ouagadougou, Burkina Faso. AIDS Care.

[4] Townsend C, Byrne L, Cortina-Borja M, Thorne C, de Ruiter A, Lyall H (2014). Earlier initiation of ART and further decline in mother-to-child HIV transmission rates, 2000–2011. AIDS.

# Sub article: P007 Influence of HIV infection in choosing to terminate pregnancy

**Published:** 2016-10-23

**Authors:** Elsa Campoa, Liliana Pedro, Filipa Azevedo, Raquel Pinho, Manuela Simão, Conceição Santos, Domitilia Faria, Carlos Santos, Luisa Arez

**Affiliations:** 1 Medicine, Algarve Hospital Center, Portimão, Portugal; 2 Obstetrics and Gynaecology, Algarve Hospital Center, Portimão, Portugal

## Abstract

**Introduction**: Pregnant HIV patients are often concerned with foetal development and IU transmission of HIV. Therefore, they frequently opt for an abortion. Antiretroviral therapy brought new options to these women due to reduced risk of mother-foetal transmission [1,2]. We estimated the incidence of abortion in HIV-infected pregnant women in our clinic, for the last 15 years.

**Material and methods**: Data were retrospectively collected. The incidence of abortions in women with a known diagnosis of HIV between 2000 and 2015 was compared with the incidence of abortions in women who were newly diagnosed with HIV during pregnancy. Statistical analysis with linear regression was done with SPSS version 23.

**Results**: Seventy-six women were included, with an average age at HIV diagnosis of 26.6 (standard deviation 5.5) years. Of 110 pregnancies, 27 (24.5%) ended in a planned abortion (Table 1). There was a significant relationship between the number of women with known HIV infection prior to pregnancy and the choice for abortion (R=0.276, p=0.004). There was no association between the prevalence of abortion and the maternal age at the time of HIV diagnostics.

**Conclusion**: This study shows that, in our sample, women already infected with HIV choose to terminate pregnancy more often than those who are already pregnant at the time of first diagnosis. Although the use of HAART reduces HIV transmission to the foetus, the presence of an HIV infection seems to play a role in deciding to terminate pregnancy. It is necessary to develop more studies about this and thoroughly inform these patients and help them to make informed decisions about termination of pregnancy.

**Table 1:** Frequencies of abortion and the moment of HIV diagnosis

	No abortion	Abortion
HIV+ previous to pregnancy	38 (64.4%)	21 (35.6%)
HIV+ during pregnancy	45 (88.2%)	6 (11.8%)

## References

[1] Maccarthy S, Rasanathan JJ, Crawford-Roberts A, Dourado I, Gruskin S (2014). Contemplating abortion: HIV-positive women's decision to terminate pregnancy. Cult Health Sex.

[2] Pilecco FB, Teixeira LB, Vigo Á, Knauth DR (2015). Post-diagnosis abortion in women living with HIV/Aids in the south of Brazil. [Article in English, Portuguese]. Cien Saude Colet.

# Sub article: P008 The association between preterm delivery and the risk of mother-to-child transmission of HIV

**Published:** 2016-10-23

**Authors:** Svitlana Posokhova, Sergey Petrovych, Svitlana Shevchenko

**Affiliations:** 1 Perinatal Center, Odessa Oblast Clinical Hospital, Odessa, Ukraine

## Abstract

**Introduction**: The prevalence of HIV among Ukrainian women is estimated to be more than 1%. Increased preterm delivery has been reported among HIV-infected women in several industrialized countries. Recent studies have identified HIV as a leading contributor to preterm delivery. Premature babies born to HIV-positive mothers are at an increased risk of vertical transmission. The aim of the study was to evaluate the rate and the risk factors for the mother-to-child transmission (MTCT) of HIV in women who had preterm delivery (PTB; <37 weeks of gestation).

**Methods**: A retrospective study looking at all premature babies born to HIV-infected mothers between 2010 and 2014 years in Odessa Regional Perinatal Center. The prematurity risk factors and the rate of MTCT of HIV were analyzed.

**Results**: In total, 122 HIV-infected women out of the 1115 eligible for inclusion into the study had spontaneous preterm delivery (10.9%) of which 40% were severe preterm (<34 weeks of gestation). The rate of MTCT in children born prematurely was 21.3%. All women with preterm birth were divided into two groups. The first group included 26 women whose children were HIV positive and the second group consisted of 96 women whose children were healthy. Mothers who gave birth to HIV-infected children more frequently had a combination of several STDs such as trichomoniasis (38.4%), syphilis (15.4%), herpes simplex (23.1%), presence of opportunistic infection (6.5%) and absence of antenatal care (9.8%). The length of time of rupture of membranes (ROM) in the Group I before delivery >12 hours was in 69.3% cases. In the Group I viral loads >10,000 copies/mL were in 23.1%, <1000 copies/mL − 15.4% (3.5 times more than in the second group), unknown − 61.5% of women.

**Conclusions**: Thus, we have found an increased incidence of MTCT observed in HIV-infected women who gave birth <32 weeks of gestation (p<0.001), had several STD (RR 1.81; 95% CI 1.20–2.56), the vaginal delivery and length of ROM before delivery >12 hours (RR 1.43; 95% CI 1.10–1.86), a high viral load >10,000 copies/mL (RR 1.62; 95% CI 1.25–2.23) and who were untreated with HAART (p<0.001).

**Table 1:** Maternal baseline characteristics

Maternal baseline characteristics	Group I HIV-positive babies (n = 26)	Group II HIV-negative babies (n = 96)	p value
Median age, years	25.8	26.5	
Intravenous drug users	3 (11.5%)	0	<0.001
Smoking	16 (61.5%)	57 (59.3%)	>0.05
Marital status, no. (%)			
Single	16 (61.5%)	62 (64.5%)	
Gravida including current pregnancy, no. (%)			
1	8 (30.7%)	43 (44.7%)	
2	12 (46.2%)	38 (39.5%)	
3	4 (15.4%)	8 (8.4%)	
4 and more	2 (7.7%)	7 (7.4%)	
Gestational age at study enrolment			
26–28 weeks	4 (15.4%)	0%	
29–31 weeks	16 (61.5%)	10 (10.4%)	<0.001
32–34 weeks	4 (15.4%)	14 (14.6%)	
35–37 weeks	2 (7.7%)	72 (75%)	<0.001
Sexually transmitted diseases			
Trichomoniasis	10 (38.4%)	16 (16.6%)	<0.01
Chlamydiosis	8 (30.7%)	20 (20.8%)	
Syphilis	4 (15.4%)	0%	<0.001
Herpes simplex	6 (23.1%)	6 (9.1%)	<0.01
Viral load >10,000 copies/mL	6 (23.1%)	20 (20.8%)	
<1000 copies/mL	4 (15.4%)	52 (54.1%)	<0.001
Unknown	16 (61.5%)	4 (4.1%)	<0.001
CD4 cell count >500 cells/µL	4 (15.4%)	68 (70.8%)	<0.001
200–499 cells/µL	2 (7.7%)	12 (12.5%)	
<200 cells/µL	4 (15.4%)	12 (12.5%)	
Unknown	16 (61.5%)	4 (4.2%)	<0.001
The length of time of rupture of membranes (ROM) <12 hours	8 (30.7%)	67 (69.7%)	<0.01
>12 hours	18 (69.3%)	29 (30.3%)	<0.01
Mode of delivery			
Vaginal	20 (76.9%)	44 (45.8%)	<0.05
Elective caesarean section	0%	36 (37.5%)	
Emergency caesarean section	6 (23.1%)	16 (16.7%)	
Triple-combined HAART	14 (53.8%)	92 (95.8%)	
Untreated	12 (46.2%)	4 (4.2%)	<0.001

# Sub article: P009 Efficacy of prophylactic antiretroviral treatment in new-born infants from HIV-positive mothers in 2012–2014, for the North-Eastern part of Romania

**Published:** 2016-10-23

**Authors:** Carmen Doina Manciuc, Liviu Prisacariu, Cristina Nicolau, Alexandra Largu

**Affiliations:** 1 HIV/AIDS, Sf. Parascheva Infectious Diseases Clinical Hospital, Iasi, Romania

## Abstract

**Introduction**: In the North-Eastern region of Romania most of the female HIV-positive population is sexually active and at child-bearing age [1]. In Romania, there is a strict protocol regarding HIV vertical transmission [2]. We aim to evaluate the efficiency of this protocol and the degree of appliance, reflected in the HIV status of new-born infants from HIV-positive mothers for a period of 3 years.

**Material and methods**: Of the 1424 patients actively monitored in the HIV/AIDS Regional Center in Iasi, Romania, 46.5% (663) are female. We evaluated retrospectively the files of all new-born infants from HIV-positive mothers for a period of 3 years (January 2012–December 2014).

**Results**: In the period mentioned above, 100 children were born (36 in 2012, 38 in 2013, 26 in 2014); one death occurred 10 days after birth, due to multiple organ malformations; the lowest weight at birth was 750 g; three of the children (3%) had a detectable viral load at birth; in one case we could not evaluate the viral load; 98 children (98%) were born through caesarean section; two were born through natural labour (2%), one of which at home. Mothers received treatment with lopinavir/ritonavir+zidovudine/lamivudine through the whole pregnancy in 81 cases; in eight cases the mothers did not receive any treatment, being tested for HIV at birth. For all new-borns prophylaxis was made with zidovudine+lamivudine for 6 weeks. Three children remained positive at 18 months, and therapy for them consisted of zidovudine+lamivudine+nevirapine.

**Conclusions**: Evaluation of pregnant HIV-positive women and prophylaxis for new-born infants in the evaluated period was conducted according to protocols, which resulted in a small percentage of HIV-positive children (3%). This is one of the aspects that make the Romanian HIV-positive population different from that of other countries [3].

## References

[1] Manciuc C, Largu A (2014). Impact and risk of institutionalized environments on the psycho-emotional development of the HIV-positive youth. Environ Eng Manag J.

[2] Manciuc C, Nicolau C, Vâţă A, Prisăcariu LJ, Matei D, Boghian A (2010). Mother to child HIV transmission in the north-east of Romania. Therap Pharmacol Clin Toxicol.

[3] Adeniyi VO, Thomson E, Ter Goon D, Ajayi IA (2015). Disclosure, stigma of HIV positive child and access to early infant diagnosis in the rural communities of OR Tambo District, South Africa: a qualitative exploration of maternal perspective. BMC Pediatr.

# Sub article: P010 Compliance of fixed-dose single tablet EVG/COBI/FTC/TDF (Stribild) regimen versus LPV/r /d4T/3TC for PEP in sexual assault victims: a retrospective sequential period study

**Published:** 2016-10-23

**Authors:** Stéphane De Wit, Agnès Libois, Pierre Mols, Stefano Malinverni

**Affiliations:** 1 Infectious Diseases, Saint-Pierre University Hospital, Brussels, Belgium; 2 Emergency, Saint-Pierre University Hospital, Brussels, Belgium

## Abstract

**Introduction**: Post-exposure prophylaxis (PEP) is recommended in most cases of sexual assaults. Due to many reasons, compliance is poor in this situation. A single-tablet regimen for PEP showed good results in terms of adherence and completion [1]. Following a previous study in our institution indicating a low compliance (40%) to PEP for sexual assault victims [2], drug regimen has been changed on 1 January 2015 from LPV/r /d4T/3TC to single tablet EVG/COBI/FTC/TDF aiming to improve compliance. In this study, we evaluate the impact of this change on compliance.

**Materials and methods**: We conducted a retrospective sequential period analysis between January 2011 and December 2015 of persons consulting at our institution for PEP following sexual assault. Data were extracted from a prospective PEP registry. Patients receiving 28 days of treatment were considered compliant. Compliance was extracted from medical records and calculated from pharmacy records.

**Results**: A total of 368 cases were included, 283 received PEP. Ninety-six percent were female with a mean age of 27 years, 50% were migrant. Exposure was vaginal receptive in 82% of cases, anal receptive in 21% and oral receptive in 27%. Seventy-one patients received a single tablet EVG/COBI/FTC/TDF and 212 received a multi-tablet regimen LPV/r /d4T/3TC twice daily. Baseline characteristics of the two groups were not statistically significantly different. Compliance was higher in EVG/COBI/FTC/TDF compared with LPV/r /d4T/3TC (52% vs. 42% respectively, p=0.158), but this did not reach statistical significance.

**Conclusion**: Switching to a well-tolerated single-pill regimen (EVG/COBI/FTC/TDF) modestly improves compliance suggesting that in sexual assault victims other drug regimens and other interventions should be implemented.

## References

[1] Foster R, McAllister J, Read TR, Pierce AB, Richardson R, McNulty A (2015). Single-tablet emtricitabine-rilpivirine-tenofovir as HIV postexposure prophylaxis in men who have sex with men. Clin Infect Dis.

[2] Malinverni S, Libois A, Gennotte AF, La Morté C, Mols P (2016). Prescription of non-occupational post-exposure HIV prophylaxis by emergency physicians: an analysis on accuracy of prescription and compliance. PLoS One.

# Sub article: P011 Increased rate of *C. trachomatis* infection after being prescribed PrEP

**Published:** 2016-10-23

**Authors:** Vinh-Kim Nguyen, Helen Trottier, Hermione Gbego Tossa, Louise Charest, Danièle Longpré, Stéphane Lavoie, Martha Cadieux, Réjean Thomas

**Affiliations:** 1 Département de Médecine Sociale et Préventive École de Santé Publique de l'Université de Montréal, Montréal, Canada; 2 Clinic and Outpatient Services, Clinique l'Actuel, Montréal, Canada; 3 Épidémiologie, Clinique l'Actuel, Montréal, Canada

## Abstract

**Introduction**: Increasing use of HIV pre-exposure prophylaxis (PrEP) using Truvada following clinical trials that have shown nearly 100% efficacy for high-risk men who have sex with men (MSM) raises concerns that resulting decreased condom use could increase sexually transmitted infections (STIs). Clinique l'Actuel (CA) is a sexual health clinic in Montréal, Québec, Canada and the largest provider of health care for MSM and people living with HIV in the city; in 2015, CA treated 60% of *N. gonorrhoea* (NG) cases in Québec province; a marked increase in NG treatments has been noted at CA since January 2016. Since August 2015 PrEP has been offered to MSM coming for STI screening and assessed as high risk by physicians; as of July 2016, 1059 MSM are receiving PrEP (85% continuous regimen, 15% intermittent regimen) at CA. PrEP may lead to a shift away from condoms as a prevention strategy, or alternatively offers a new prevention strategy for those who already engage in condom-less sex.

**Methods**: To assess whether PrEP increases condom-preventable STIs, we enrolled patients with >1 year of follow-up before and after PrEP prescription. Hundred and thirty-three patients were included. Patients were seen every 3 months for a physician evaluation, behavioural questionnaire and full STI screen that included PCR swabs for anal, oral or urethral NG and *C. trachomatis* (CT). Cases were ascertained by electronic results with a manual chart review to confirm positives. The proportion of individuals infected with CT and NG were compared before and after exposure to PrEP using two-sided chi squared (χ^2^) test.

**Results**: The proportion of individuals infected with anal, oral or urethral CT in the year prior to PrEP were 10%, 2%, 3%, respectively and in the year post-PrEP were 20%, 2%, 11% in the exposure period (p-value 0.00, 1.00, 0.01, respectively). The percentage of individuals infected with CT at any site pre- and post-PrEP were 13% and 26% (p=0.01). The proportion of individuals infected with anal, oral or urethral NG in the year prior to PrEP were 9%, 8% and 8%, respectively, and in the year after PrEP were 14%, 11%, 6% (p-value 0.24, 0.29, 0.62, respectively). The percentage of individuals infected with NG pre- and post-PrEP were 17% and 26% (p=0.07), respectively.

**Conclusion**: Increased rates of CT post-PrEP suggest a shift away from condom use. Increased rates of asymptomatic STIs such as CT but not NG post-PrEP warrant further study.

# Sub article: P012 Risk perception in MSM taking PrEP

**Published:** 2016-10-23

**Authors:** Stefano Boccino, Ushaa Kanagalinghan, Nneka Nwokolo, Gary Whitlock

**Affiliations:** 1 Chelsea and Westminster Hospital, London, UK

## Abstract

**Introduction**: There are concerns that those taking pre-exposure prophylaxis (PrEP) may increase their frequency of condomless sexual intercourse and hence increase their risk of acquiring STIs other than HIV. We wished to survey the perception of these concerns for those on PrEP in the UK.

**Materials and methods**: Between 6 February and 7 June 2016, we surveyed MSM attending a central London sexual health clinic for PrEP monitoring. The survey was a self-reported anonymous paper questionnaire asking about respondents’ PrEP regimen, length of time on PrEP and how respondents’ sexual activity and partner selection had changed and how they felt about the risk of acquiring STIs and HIV since starting PrEP. P-values were computed using Fisher's exact test.

**Results**: Hundred questionnaires were completed: all respondents were MSM. The majority (77%) were taking daily PrEP, 19% event-driven and four not-specified. The median time on PrEP was 3 months. Since starting PrEP most respondents indicated they were more relaxed about their risk of acquiring HIV (83%). With regard to risk of acquiring other STIs, 20% were less relaxed and most “felt the same” (78%). Most (63%) indicated that the number of times they had had condomless sex had not changed since starting PrEP; 30% indicated it had increased. There was a reported increase in condomless intercourse in those taking PrEP for longer than 4 months compared with those more recently starting (p=0.049) (Table 1).

**Conclusions**: Our survey suggests that MSM taking PrEP in London, UK are more relaxed about acquiring HIV but less so about other STIs. There appears to be an association between length of time on PrEP and increase of condomless sexual activity. It is important that PrEP guidelines incorporate regular STI screening and risk reduction interventions.

**Table 1:** Since you've started PrEP how do you think the number of times you have had condomless sex has changed?

Length on PrEP	Decreased	No change	Increased	Total
4 months or more	3	22	18	43
Less than 4 months	2	41	12	55
Total	5	63	30	98*

* Two respondents did not indicate length on PrEP.

# Sub article: P013 Attitudes towards pre-exposure prophylaxis against HIV infection among individuals seeking voluntary counselling and testing for HIV in Taiwan

**Published:** 2016-10-23

**Authors:** Lan-Hsin Chang, Wen-Chun Liu, Cheng-Hsin Wu, Yi-Chieh Lee, Hsin-Yun Sun, Chien-Ching Hung, Shan-Chwen Chang

**Affiliations:** 1 Internal Medicine, National Taiwan University Hospital, Taipei, Taiwan; 2 Internal Medicine, Lotung Poh-Ai Hospital, Medical Lo-Hsu Foundation, Internal Medicine, Yilan City, Taiwan

## Abstract

**Introduction**: Pre-exposure prophylaxis (PrEP) using tenofovir disoproxil fumarate (TDF)/emtricitabine (FTC) against HIV infection have been shown to be efficacious in clinical trials. Successful implementation of PrEP in the field settings requires understanding of the knowledge and opinions of PrEP among the individuals who will benefit most from the intervention. This survey aimed to understand the attitudes towards PrEP among individuals in Taiwan who sought voluntary counselling and testing (VCT) for HIV at a university hospital, where the HIV incidence rates among men who have sex with men (MSM) was estimated 5.5 per 100 person-years of follow-up between 2007 and 2015.

**Materials and methods**: Between April and June, 2016, a survey was conducted among VCT clients who completed an anonymous questionnaire interview with the assistance of a trained counsellor to inquire about the risks for HIV infection or other sexually transmitted infections and the knowledge of PrEP against HIV infection. We collected information on demographics, educational achievement, occupation, income and risk exposures that prompted the VCT visits. Tests for anti-HIV antibody and syphilis were performed. Multivariate logistic regression analysis was performed to identify the associated factors with consideration to initiate PrEP. All of the variables with p<0.2 in univariate analysis when comparisons were made between those who would consider PrEP and those who would not were entered into multivariate analysis.

**Results**: During the 3-month study period, all 611 individual clients with a mean age of 30.1 years (SD 8.3) seeking VCT service agreed to participate in this survey; 87.6% were male, 68.2% MSM, and 74% had full-time or part-time jobs, and 20% were students. About one-third of the clients reported unprotected anal sex with fixed (32.5%) or unfixed partners (35.0%) within 3 months and use of recreational drugs (6.4%) or alcohol consumption (18.0%). Less than 40% (37.5%) knew PrEP while 68.8% knew post-exposure prophylaxis before this survey. Overall, 309 individuals (50.6%) would consider to initiate PrEP and 83.5% of them would choose event-driven PrEP strategy. In multivariate analysis, knowledge of PrEP (adjusted odds ratio [AOR] 5.252; 95% CI 1.066–25867) and having unprotected anal sex with unfixed partners (AOR 19.574; 95% CI 2.259–169.575) before this survey was statistically significantly associated with consideration to initiate PrEP with TDF/FTC.

**Conclusions**: To facilitate successful implementation of PrEP against HIV infection, increase of awareness by providing information, education and communication with respect to PrEP is important to the population at risk.

# Sub article: P014 “Self-perceived” pre-exposure prophylaxis adherence and its relationship to self-reported “actual adherence” among Thai men who have sex with men and transgender women

**Published:** 2016-10-23

**Authors:** Tarandeep Anand, Stephen Kerr, Tanakorn Apornpong, Chattiya Nitpolprasert, Jintanat Ananworanich, Praphan Phanuphak, Nittaya Phanuphak

**Affiliations:** 1 Adam's Love, Thai Red Cross AIDS Research Centre, Bangkok, Thailand; 2 Biostatistics, HIV Netherlands Australia Thailand Research Collaboration, Thai Red Cross AIDS Research Centre, Bangkok, Thailand; 3 Therapeutics Research, US Military HIV Research Program, Walter Reed Army Institute of Research, Silver Spring, MD, USA; 4 Thai Red Cross AIDS Research Centre, Bangkok, Thailand; 5 HIV Prevention, Thai Red Cross AIDS Research Centre, Bangkok, Thailand

## Abstract

**Introduction**: In Bangkok, one in three Thai men who have sex with men (MSM) are HIV positive [1] and prevalence among transgender (TG) women ranges from 10 to 17% [2]. Pre-exposure prophylaxis (PrEP) is a safe and effective HIV prevention method [3]. Thai MSM and TG have shown high interest in PrEP uptake, but data on PrEP adherence and retention remain suboptimal. We sought to understand how self-perceived adherence related to actual adherence, to inform the development of future PrEP adherence interventions.

**Methods**: Between January 10 and 11 April 2016, the Thai Red Cross AIDS Research Centre (TRCARC)'s Adam's Love (www.adamslove.org) enrolled MSM and TG into free PrEP services using its real-time PrEP eCounselling and novel Online-to-Offline (O2O) model at four sites in Bangkok including TRCARC Anonymous Clinic, Adam's Love private clinic and two community-based drop-in centres. Data were gathered on their self-perceived adherence at PrEP initiation and compared with self-reported adherence 1 month post PrEP use. Logistic regression was used to calculate the OR for demographic and behavioural characteristics associated with 100% self-reported adherence. Factors significant in univariate analysis at p<0.1 were adjusted for in a multivariate model. Participants who withdrew or were lost to follow-up were imputed as non-adherent.

**Results**: A total of 168 participants were enrolled into the programme. Data from 132 participants with available adherence data after 1 month of PrEP were analyzed. At enrolment, 105 (79.5%) participants believed they were likely or extremely likely to be adherent to daily PrEP, but only 13 (9.8%) had reported taking all seven pills in the week prior to the month 1 visit. After adjusting for selling sex and cannabis use in a multivariate model, two factors were independently associated with perfect (100%) self-reported adherence. These were having no income because they were students or unemployed (OR 5.6, 95% CI 1.3–23.4; p=0.02) and being aware of sex partners’ HIV status (OR 6.7, 95% CI 1.6–28.7; p=0.01).

**Conclusions**: Although most Thai MSM and TG believed they would be highly adherent to PrEP at enrolment, this perception correlated poorly with actual adherence. Participants without income and those aware of the HIV status of their sexual partners were significantly more likely to report 100% adherence. Innovative PrEP adherence interventions to help overcome daily adherence barriers and promote consistent high levels of PrEP adherence are urgently needed.

## References

[1] van Griensven F, Varangrat A, Wimonsate W, Tanpradech S, Kladsawad K, Chemnasiri T (2010). Trends in HIV prevalence, estimated HIV incidence, and risk behavior among men who have sex with men in Bangkok, Thailand, 2003–2007. J Acquir Immune Defic Syndr.

[2] Guadamuz TE, Wimonsate W, Varangrat A, Phanuphak P, Jommaroeng R, McNicholl JM (2011). HIV prevalence, risk behavior, hormone use and surgical history among transgender persons in Thailand. AIDS Behav.

[3] Grant RM, Lama JR, Anderson PL, McMahan V, Liu AY, Vargas L (2010). Preexposure chemoprophylaxis for HIV prevention in men who have sex with men. N Engl J Med.

# Sub article: P015 Polish infectious diseases physicians’ attitudes and beliefs about pre-exposure prophylaxis for HIV prevention

**Published:** 2016-10-23

**Authors:** Jacek Kolodziej, Tomasz Mikula, Alicja Wiercinska-Drapalo

**Affiliations:** 1 Department of Infectious and Tropical Diseases and Hepatology, Medical University of Warsaw, Warsaw, Poland

## Abstract

**Introduction**: Pre-exposure prophylaxis (PrEP) for HIV prevention is rarely implemented in Polish setting. It remains controversial among infectious diseases specialists, yet no research was undertaken to identify the concerns. Providing deeper understanding of the exact nature of physicians’ attitudes about PrEP should help direct further efforts into resolving the controversies.

**Materials and methods**: Anonymous questionnaires (150) were distributed among Polish infectious diseases specialists with questions addressing their attitudes, beliefs, expectations and experiences related to PrEP. Data were collected in regard to sex, age, years of practice, city of practice and whether they were members of academic faculty.

**Results**: Overall, 62 physicians (41.3%) returned the questionnaires (mean age 42.5, SD 10.5; 69.4% women). Only nine doctors (14.7%) had ever prescribed PrEP to patients. Majority considered there were indications for use of PrEP in seronegative women (70.5%) and seronegative men (65.6%) planning pregnancy with a serodiscordant partner and consequently believed PrEP should be funded by the state in these circumstances (63.94% and 60.7%, respectively). No other presented situation (such as: men who have sex with men with a history of unprotected anal intercourse, serodiscordant couples and intravenous drug users) was considered an indication for PrEP or a justification for state funding. In the sample, 63.9% of doctors agreed or strongly agreed with a statement that PrEP was a well-known issue to them compared to 8.2% who disagreed or strongly disagreed. Majority (67.7% vs. 11.3%) believed PrEP was efficacious in preventing new infections and 56.5% (vs. 21%) shared the view it was a major accomplishment in HIV prevention. Common concerns included: PrEP leading to abandonment of safer sex practices (64.5% vs. 16.1%) or serving as an encouragement to risky sexual behaviours (58.1% vs. 24.2%) and potential drug-resistance emergence (54.1% vs. 24.6%). Vast majority (77.4% vs. 11.3%) believed that patients should radically alter their behaviours instead of relying on pharmacologic interventions.

**Conclusions**: Polish physicians involved in HIV treatment hold generally favourable views of PrEP and majority consider themselves sufficiently educated in this regard. Concerns are raised, however, in terms of encouragement to risky sexual behaviours, which should be addressed in research in Central and Eastern European population of potential PrEP users.

# Sub article: P016 Pre-exposure prophylaxis awareness in individuals accessing a non-occupational post-exposure prophylaxis (NPEP) program

**Published:** 2016-10-23

**Authors:** John Peel, Nathan Lachowsky, David Moore, Shahab Jabbari, Wendy Zhang, Silvia Guillemi, Marianne Harris, Troy Grennan, Jason Wong, Val Montessori, Gina Ogilvie, Julio Gonzalez Montaner, Mark Hull

**Affiliations:** 1 Providence Health Care, British Columbia Centre for Excellence in HIV/AIDS, Vancouver, Canada; 2 Infectious Disease, BC Centre for Disease Control, Vancouver, Canada

## Abstract

**Introduction**: Biomedical prevention strategies are an important aspect of combination prevention interventions for HIV infection. Programmes targeting populations at highest HIV risk are likely to be cost-effective. Individuals who require post-exposure prophylaxis (PrEP) have been shown to be at high risk for subsequent HIV seroconversion, yet their knowledge of alternate prevention strategies is unclear. We undertook to assess overall awareness of PrEP amongst individuals accessing an NPEP program in Vancouver, Canada.

**Methods**: Individuals accessing an NPEP pilot program offered at five sites (four community-based and via an emergency department of a tertiary care hospital) were sequentially recruited to complete a self-administered HIV knowledge questionnaire between July 2012 and March 2014. Awareness of PrEP was initially dichotomized as a yes/no response, and for individuals reporting PrEP awareness, level of self-reported knowledge was further categorized using a four-point Likert scale. Factors associated with PrEP awareness were determined using Fisher's exact test for categorical values and Wilcoxon rank sum for continuous variables (p<0.05 considered significant).

**Results**: Overall 134 individuals were included, 95% male, 70% white, with median age of 36 years (interquartile range [IQR] 28–42). Baseline exposure for NPEP included condomless anal sex (CAS) in 84% of individuals (81% MSM) and IDU in 4% of individuals. Overall n=75 (56%) individuals reported being aware of PrEP, with 47 (63%) reporting moderate/high levels of knowledge, and 51 (68%) were willing to use PrEP. PrEP awareness was positively associated with reporting MSM partners (89% vs. 72%, p=0.018), being non-IDU (100% vs. 84%, p=0.004) and reporting high levels of knowledge regarding role of HIV viral load in transmission (29% vs. 8%, p<0.001). In a multivariate logistic model, high levels of knowledge regarding HIV viral load in transmission remained the only factor significantly associated with awareness of PrEP (aOR 3.34, 95% CI 1.12–9.90, p=0.042).

**Conclusions**: MSM individuals accessing NPEP services report both high levels of HIV risk and PrEP awareness. Combination NPEP/PrEP programs should be considered for at-risk individuals.

# Sub article: P017 PrEP use in Lisbon while waiting for a policy

**Published:** 2016-10-23

**Authors:** Paula Meireles, Miguel Rocha, Maria José Campos, Henrique Barros

**Affiliations:** 1 Epidemiology Research Unit, Instituto de Saúde Pública da Universidade do Porto, Porto, Portugal; 2 Grupo de Ativistas em Tratamentos, CheckpointLX, Lisboa, Portugal; 3 Faculdade de Medicina, Universidade do Porto, Porto, Portugal

## Abstract

**Introduction**: Pre-exposure prophylaxis (PrEP) with tenofovir/emtricitabine (TDF/FTC) has shown to be effective in preventing HIV among high-risk HIV-negative men who have sex with men (MSM). These overwhelming results of the trials put pressure on countries to make PrEP available. Few are already dispensing TDF/FTC as PrEP, Portugal is not one of those, although we remain one of the most affected populations in Western Europe. However, some individuals report the use of PrEP. We aimed to assess the frequency of PrEP use and the characteristics of users in an HIV-negative MSM cohort [1].

**Materials and methods**: Using data from the Lisbon Cohort of MSM, an open prospective cohort of 4243 adult HIV-negative MSM, we performed a case-cohort analysis; 28 (0.7%) reported to have used PrEP, and we randomly selected 112 controls. Proportions were compared using the chi-square or Fisher's exact test and medians using the Mann-Whitney.

**Results**: PrEP users had a significantly higher median number of visits in the cohort (3 vs. 1, p<0.001), were more frequently born in countries other than Portugal (39.1% vs. 17.0%, p=0.025), reported more frequently to know and to have used post-exposure prophylaxis (PEP) at baseline (34.6% vs. 52.3% did not know about PEP and 11.5% vs. 0.9% used PEP, p=0.014). Always using condom with an occasional partner were more frequently reported among PrEP users (68% vs. 48%, p=0.091). Groups were similar in terms of age, education and condom use with an HIV-positive steady partner and a STI diagnosis in the previous 12 months to baseline.

**Conclusions**: In the absence of an official policy some MSM are already using TDF/FTC in Portugal, particularly those born abroad and are concerned with their protection.

## References

[1] Meireles P, Lucas R, Carvalho C, Fuertes R, Brito J, Campos MJ (2015). Incident risk factors as predictors of HIV seroconversion in the Lisbon cohort of men who have sex with men: first results, 2011–2014. Euro Surveill.

# Sub article: P018 Increasing ART coverage and viral suppression are associated with a substantial decline in new HIV infections in the Austrian Tyrol

**Published:** 2016-10-23

**Authors:** Gisela Leierer, Ard van Sighem, Mario Sarcletti, Maria Kitchen, Martin Gisinger, Michaela Rappold, Bruno Ledergerber, Robert Zangerle

**Affiliations:** 1 Department of Dermatology and Venereology, Medical University of Innsbruck, Innsbruck, Austria; 2 Research, Stichting HIV Monitoring, Amsterdam, Netherlands; 3 Division of Infectious Diseases, University Hospital Zurich, Zurich, Switzerland

## Abstract

**Introduction**: UNAIDS has set the goal that 90% of people living with HIV (PLHIV) are diagnosed, that 90% of all people diagnosed receive ART and 90% of all people receiving ART have viral suppression. Apart from being a challenging goal, there is controversy whether the 90–90–90 UNAIDS targets suffice to curb the HIV epidemic.

**Methods**: Patients from the University Hospital Innsbruck (UHI; covers ≥99% patients with ART) who had their last residency in the Austrian Tyrol. PLHIV estimates were obtained using back-calculation models [1] to estimate HIV incidence and the undiagnosed fraction from the patients referred to UHI. The proportion ever diagnosed and still living in Tyrol who ever initiated ART and the proportion of them who were virally suppressed (≤200 copies/mL) were assessed for the years 2001 to 2015. Missing HIV RNA was considered as unsuppressed.

**Results**: PLHIV were estimated to be 271 in 2001 and 501 in 2015. The fraction undiagnosed decreased from 18% (95% CI 12–24%) in 2011 to 11% (95% CI 7–27%) in 2015. The proportion of diagnosed patients who have ever started ART increased from 64% in 2001 to 91% in 2015 (Figure 1). Among those who started ART, the proportion of individuals virally suppressed improved from 79 to 97% between 2001 and 2015 (Figure 1). The fraction of the virally suppressed among PLHIV increased from 39% in 2001 to 79% in 2015, which is well above the 90–90–90 target of 73%. Estimates of the number of new HIV infections decreased from 26 (95% CI 18–34) in 2009 to eight (95% CI 0–76) in 2015 (Figure 2).

**Conclusions**: Although the relationship between the fraction of virally suppressed and HIV infections does not demonstrate causality it provides strong supportive evidence that treatment as prevention can reduce the epidemic. In addition, our data support that in this setting the 90–90–90 targets may indeed curb the epidemic.

**Figure 1:** Temporal trends of the proportion on ART of those diagnosed and the proportion with suppressed HIV-RNA of those on ART in Tyrol.

**Abstract P018–Figure 2:** Number of new HIV infections in Tyrol over time.

## References

[1] European Centre for Disease Prevention and Control (ECDC) HIV modelling tool [software application]. Version 1.0.2.

# Sub article: P019 Implementation of isoniazid preventive therapy for people living with HIV in Northwestern Nigeria: integration challenges and issues

**Published:** 2016-10-23

**Authors:** Abiola Adepoju, Ginika Egesemba, Umar Kangiwa, Peace Igene

**Affiliations:** 1 HIV Clinical Care, Management Sciences for Health, Birnin Kebbi, Nigeria; 2 Prevention Organization AIDS Care and Treatment, Management Sciences for Health, Abuja, Nigeria; 3 Department of Hematology, Federal Medical Center, Birnin Kebbi, Nigeria; 4 Science, Loveworld International, Lagos, Nigeria

## Abstract

**Introduction**: The risk of acquiring tuberculosis (TB) by people living with HIV (PLHIV) could be drastically reduced through provision of isoniazid preventive therapy (IPT). In Nigeria, there is paucity of data on the routine implementation of this intervention and its effectiveness in low-resource settings. The MSH model provides 6 months of IPT at 2-month intervals for eligible PLHIV. This study assessed pattern of IPT provision and impact among PLHIV in six USAID-funded hospitals in Kebbi state, Nigeria.

**Methodology**: Data were collected in November 2015 by reviewing a total of 1653 folders of adult and paediatric PLHIV placed on IPT across six health facilities between January 2013 and October 2015. Descriptive and inferential statistics were used to analyze findings.

**Results**: Of the 1653 folders reviewed, 1134 have completed IPT while 519 were still on IPT. Only 13 (1.1%) of those who completed IPT developed TB after average period of 11 months. Only one (0.1%) developed TB while on IPT. Individuals who completed IPT without developing TB have the same IPT default rate (31%) as those who developed TB after IPT completion. Those who completed IPT without developing TB were found to have lower rate (10%) of ART default compared with higher rate (60%) in those who developed TB after IPT completion. IPT default was highest in the first few weeks of ART initiation.

**Conclusion**: ART and IPT have combined effect of reducing TB incidence among PLHIV. Adherence to ART as measured by default rate has greater impact compared with adherence to IPT in the reduction of the incidence of TB among PLHIV. Coupled with enhanced adherence services, pre-packaging IPT and ART as single prescription will help to overcome integration challenges and reduce TB burden among PLHIV.

# Sub article: P020 Cascade of care in TAK project: strong in right side, but weak in left side

**Published:** 2016-10-23

**Authors:** Justyna Kowalska, Leah Shepherd, Magdalena Ankiersztejn-Bartczak, Ewa Firlag-Burkacka, Andrzej Horban, Amanda Mocroft

**Affiliations:** 1 Hospital for Infectious Diseases in Warsaw, Medical University of Warsaw, Warsaw, Poland; 2 Royal Free Campus, University College Medical School, London, UK; 3 Foundation of Social Education, Foundation for Social Education (FES), Warsaw, Poland; 4 HIV Outpatient Clinic, Hospital for Infectious Diseases in Warsaw, HIV Outpatient Clinic, Warsaw, Poland

## Abstract

**Introduction**: Early cART remains the most effective HIV prevention strategy, yet poor linkage to care after HIV diagnosis may compromise this benefit [1]. It is important to better understand patient characteristics and risk for HIV acquisition and association with response to cART.

**Method**: The TAK project collects all information on patients diagnosed with HIV in community-based testing facilities (CBVCTs) in central Poland, follows their linkage to care and ongoing routine clinical care [2]. Data collected for persons diagnosed from 2010 to 2013 in CBVCTs were linked with HIV clinic records. Individuals linked to care were followed from first CBVCTs visit until last visit in the HIV clinic or 5 December 2015. Cox proportional hazard models were used to identify factors associated with viral suppression (VS) (first VS: HIV RNA <50 copies/mL).

**Results**: Two hundred and thirty-two persons were HIV+, 144 (62%) linked to care, 116 (80% of those linked to care) started cART during follow-up, of which 113 (97%) achieved VS with median time to VS of 5 (IQR 4–5) months. Median time from linkage to starting cART was 6 (IQR 3–9) months. In those who started cART 111 (96%) were men, 98 (84%) homosexual, 26 (22%) had syphilis at baseline, 34 (29%) had a partner who had never tested for HIV, 33 (28%) had a HIV+ partner, 66 (57%) always using condoms with casual partners, 24 (21%) had sex on drugs or alcohol. Median age was 32 (IQR 28–37) years, baseline CD4 count 352 (259–415) cells/µL and HIV RNA 4.5 (3.9–5.1) log copies/mL. After adjustment, factors associated with higher rate of VS were bisexual orientation, non-PI-based regimen, HLA B5701(+) and with lower rate were unknown syphilis status and higher HIV RNA at cART start (Figure 1).

**Conclusions**: In this community-based setting, although a low proportion of persons were linked to care, almost all those linked to care started cART and achieved rapid VS during follow-up. We observed high rates of VS, irrespective of prior HIV-associated risk behaviours. Linkage to care remains the highest priority in prevention strategies in central Poland.

**Figure 1:** HR for time to viral suppression after starting cART.

## References

[1] Phillips AN, Munderi P, Revill PA, El-Sadr WM and, Lundgren JD (2014). Antiretroviral therapy recommendations for the global community: aspiration versus reality. AIDS.

[2] Shepherd L, Ankiersztejn-Bartczak M, Cybula A, Czeszko-Paprocka H, Firlag-Burkacka E, Horban A Poor linkage to care despite significant improvement in access to early cART – data from Test and Keep in Care (TAK) project. [Abstract PS 8/4.].

# Sub article: P021 Durability and tolerability of first-line combination including two NRTI and RAL or ATV/r or DRV/r in patients enrolled in the ICONA Foundation cohort

**Published:** 2016-10-23

**Authors:** Antonella d'Arminio Monforte, Patrizia Lorenzini, Alessandro Cozzi-Lepri, Cristina Mussini, Antonella Castagna, Franco Baldelli, Massimo Puoti, Francesco Mazzotta, Nicola Abrescia, Sergio Lo Caputo, Nicola Gianotti, Andrea Antinori, Study Group on behalf of Icona Foundation

**Affiliations:** 1 Department of Health Sciences, Clinic of Infectious and Tropical Diseases, University of Milan, ASST Santi Paolo e Carlo, Milan, Italy; 2 Clinical Department, National Institute for Infectious Diseases, IRCCS, Lazzaro Spallanzani, Rome, Italy; 3 Department of Infection and Population Health, Royal Free Campus, University College London Medical School, London, UK; 4 Infectious Disease Clinic, University of Modena and Reggio Emilia, Modena, Italy; 5 Department of Infectious Diseases, San Raffaele Scientific Institute, Università Vita-Salute San Raffaele, Milan, Italy; 6 Unit of Infectious Diseases, Department of Medicine, University of Perugia, Perugia, Italy; 7 Department of Infectious Diseases, Ospedale Niguarda Ca’ Granda, Milan, Italy; 8 Division of Infectious Diseases, Ospedale Santa Maria Annunziata, Florence, Italy; 9 Infectious Diseases Unit, D. Cotugno Hospital, AORN dei Colli, Naples, Italy; 10 Department of Infectious Diseases, University of Bari, Bari, Italy; 11 Department of Infectious Diseases, San Raffaele Scientific Institute, Milan, Italy; 12 Icona Foundation, Milan, Italy

## Abstract

**Introduction**: Although several randomized studies have shown that PI/r-including first-line regimens have lower efficacy as compared to those including integrase inhibitors, they are still used in a number of conditions, mainly due to their high genetic barrier. We aimed to reproduce in a real-life setting results of the ACTG 5257 trial, comparing virologic response, durability and tolerability of first line raltegravir (RAL)-including regimens to regimens including either darunavir/ritonavir (DRV/r) or atazanavir/r (ATV/r).

**Materials and methods**: Patients from the Icona cohort initiating their first ART regimen from January 2008 (date of availability of RAL in Italy) with TDF/FTC or ABC/3TC plus ATV/r or DRV/r or RAL with at least one visit and one CD4 and VL determination in follow-up were included in the analyses. Primary endpoint: treatment failure (TF): virologic failure (VF) (HIV RNA >200 copies/mL ≥6 months of therapy) or discontinuation of RAL, ATV/r or DRV/r whatever first occurs. Secondary endpoints: VF50 (HIV RNA >50 copies/mL ≥6 months of therapy); discontinuation (TD) of RAL, ATV/r or DRV/r due to all causes and discontinuation due to toxicity/tolerability (TDT). Discontinuation of backbone does not count as endpoint. Survival analysis with Kaplan-Meier curves and Cox regression model.

**Results**: A total of 2072 patients were analyzed: 939 (45.3%) started ATV/r-including regimens, 932 DRV/r (45.0%) and 202 (9.7%) RAL. Several differences in demographic and clinical characteristics according to the regimen used were identified (see Table 1). In a median follow-up of 1.4 years (IQR 0.6–2.7), TF occurred in 1028 patients, 28.2/100 PYFU (95% CI 25.6–30), VF50 in 372, 7.9/100 PYFU (95% CI 7.2–8.8), TD in 978 26.3/100 PYFU (95% CI 24.7–28), and TDT in 326 patients, 8.8/100 PYFU (95% CI 7.8–9.8). In the multivariable analysis, no differences in TF according to regimens was found. RAL-including regimens resulted in 46% lower risk of VF50 compared to ATV/r. Both DRV/r-including and RAL-including regimens resulted in significantly lower risk of TDT compared to ATV/r-including regimens (Table 2).

**Conclusions**: Concerning the risk of discontinuation for toxicity our results are consistent with those observed in A5257. When considering virologic failure, only with the threshold of 50 copies/mL results were somewhat different from those observed in the randomized comparison, suggesting lower rate of virologic failure for RAL than ATV/r, although the difference was small and likely due to methodology discrepancy and residual confounding cannot be ruled out.

**Table 1:** Characteristics of the included patients

	ATV/r (N = 939)	DRV (N = 931)	RAL (N = 202)	p
**Male gender, n (%)**	717 (76.4%)	764 (82.1%)	165 (81.7%)	0.007
**Age, years, median (IQR)**	39 (32–47)	40 (32–49)	43 (35–51)	0.002
**Italians, n (%)**	716 (76.2%)	749 (80.4%)	173 (85.6%)	0.004
**Mode of HIV transmission, n (%)**				
Heterosexual	430 (45.8%)	388 (41.7%)	89 (44.1%)	<0.001
IVDU	113 (12.0%)	57 (6.1%)	8 (4.0%)	
Homosexual	339 (36.1%)	396 (42.5%)	89 (44.1%)	
Other/unknown	57 (6.1%)	90 (9.7%)	16 (7.9%)	
**AIDS, n (%)**	81 (8.6%)	148 (15.9%)	21 (10.4%)	<0.001
**HCV co-infection, n (%)**				
Positive	120 (12.7%)	74 (7.9%)	11 (5.4%)	<0.001
Negative	723 (77.0%)	737 (79.2%)	161 (79.7%)	
Not known	96 (10.2%)	120 (12.9%)	30 (14.8%)	
**HBV co-infection, n (%)**				
Positive	40 (4.3%)	33 (3.5%)	13 (6.4%)	0.224
Negative	760 (80.9%)	745 (80.0%)	152 (75.2%)	
Not known	139 (14.8%)	153 (16.4%)	37 (18.3%)	
**CD4 cell/mm**^**3**^**, n (%)**				
0–200	98 (10.4%)	11 (1.2%)	13 (6.4%)	<0.001
201–350	350 (37.3%)	264 (28.4%)	32 (15.8%)	
351–500	348 (37.1%)	386 (41.5%)	51 (25.2%)	
501 +	143 (15.2%)	270 (29.0%)	106 (52.5%)	
Missing	54 (5.7%)	81 (8.7%)	17 (8.4%)	
**CD4 cell/mm**^**3**^**, median (IQR)**	302 (168–413)	250 (87–393)	351 (206–504)	<0.001
**HIV RNA copies/mL, n (%)**				
50–20,000	235 (25.0%)	176 (18.9%)	54 (26.7%)	<0.001
20,000–100,000	272 (29.0%)	225 (24.2%)	60 (29.7%)	
100,000–250,000	145 (15.4%)	164 (17.6%)	29 (14.4%)	
250,000 +	187 (19.9%)	230 (24.7%)	35 (17.3%)	
Missing	100 (10.6%)	136 (14.6%)	24 (11.9%)	
**HIV RNA log10 copies/mL, median (IQR)**	4.8 (4.2–5.3)	5.0 (4.4–5.5)	4.7 (4.1–5.3)	<0.001
**Year of cART start**				
2008–2009	98 (10.4%)	11 (1.2%)	13 (6.4%)	<0.001
2010–2011	350 (37.3%)	264 (28.7%)	32 (15.8%)	
2012–2013	348 (37.1%)	386 (41.5%)	51 (25.2%)	
2014–2015	143 (15.2%)	270 (29.0%)	106 (52.5%)	
**NRTIs combination**				
FTC + TDF	808 (86.1%)	803 (86.2%)	173 (85.6%)	0.973
ABC + 3TC	131 (13.9%)	128 (13.8%)	29 (14.4%)	

**Abstract P021–Table 2:** Relative hazards of reaching various outcomes from fitting a Cox regression model

Outcomes	Unadjusted HR (95% CI)	p	Adjusted HR (95% CI)	p
**TF (VF >200 copies/mL or discontinuation)**				
ATV/r	1.00		1.00	
DRV/r	0.99 (0.87–1.12)	0.821	0.92 (0.80–1.05)	0.222
RAL	1.01 (0.80–1.28)	0.930	0.84 (0.66–1.08)	0.175
**VF >50 copies/mL**				
ATV/r	1.00		1.00	
DRV/r	0.92 (0.74–1.13)	0.431	0.88 (0.70–1.10)	0.270
RAL	0.51 (0.30–0.86)	0.011	0.54 (0.32–0.93)	0.026
**All-cause discontinuation**				
ATV/r	1.00		1.00	
DRV/r	1.00 (0.88–1.15)	0.935	0.93 (0.81–1.07)	0.296
RAL	1.08 (0.85–1.38)	0.509	0.88 (0.69–1.13)	0.321
**Discontinuation due to toxicity**				
ATV/r	1.00		1.00	
DRV/r	0.70 (0.56–0.89)	0.003	0.65 (0.51–0.82)	<0.001
RAL	0.44 (0.26–0.76)	0.003	0.34 (0.20–0.60)	<0.001

Models were adjusted for gender, age, nation of birth, mode of HIV transmission, hepatitis co-infection status, AIDS diagnosis, nucleoside pair started, baseline CD4 count and viral load and year of starting cART.

# Sub article: P022 HIV-1 Combinectin GSK3732394: a long-acting inhibitor with multiple modes of action

**Published:** 2016-10-23

**Authors:** Mark Krystal, David Wensel, Yongnian Sun, Jonathan Davis, Thomas McDonagh, Zhufang Li, Sharon Zhang, Matt Soars, Mark Cockett

**Affiliations:** 1 Discovery, ViiV Healthcare, Wallingford, CT, USA; 2 Bristol-Myers Squibb, Molecular Discovery Technologies, Waltham, MA, USA; 3 Virology, Bristol-Myers Squibb, Wallingford, CT, USA; 4 Preclinical Candidate Optimization, Bristol-Myers Squibb, Wallingford, CT, USA

## Abstract

**Introduction**: Long-acting antiretrovirals could provide a useful alternative to daily oral therapy for HIV-1 infected individuals. However, the need for combination therapy and the potential for multiple IV/IM injections or tolerability issues may create roadblocks to this type of therapy. Adnectins are small proteins derived from the 10th type III domain of the human fibronectin protein that possess modifiable binding loops akin to the complementarity determining region of an antibody. Using Adnectins, we have developed the Combinectin inhibitor GSK3732394 (BMS-986197), a long-acting biologic with three independent and synergistic modes of HIV entry inhibition that potentially could be self-administered as a long-acting subcutaneous injection.

**Methods**: Adnectins targeting CD4 and a region of gp41 were isolated and optimized for antiviral potency and biophysical characteristics. The anti-gp41 Adnectin was joined at its amino terminus to the anti-CD4 Adnectin via a peptide linker. A third inhibitor, an alpha-helical peptide fusion inhibitor, was linked to the carboxyl end of the anti-gp41 Adnectin via another linker. Finally, a human serum albumin (HSA) molecule was attached to amino terminus of the anti-CD4 Adnectin to optimize in vivo PK.

**Results**: The EC50s of the isolated anti-CD4 Adnectin, anti-gp41 Adnectin and fusion inhibitor peptide were 8.5, 5.4 and 0.4 nM, respectively. Various synergies were obtained through linking all three inhibitors into a single molecule. Optimally combining the two Adnectins increased potency over 100-fold to ~30 pM. Addition of the fusion inhibitor peptide resulted in an increased resistance barrier compared to the separate components, as virus resistant to any one of the three components did not affect the potency of GSK3732394. Addition of HSA to the amino terminus decreased potency to 0.27 nM, but improved PK, leading to a projected weekly human dose. GSK3732394 exhibited broad spectrum activity and was efficacious in a mouse model of HIV-1 infection.

**Conclusions**: GSK3732394 is a novel recombinant biologic molecule containing three independent HIV inhibitors that has been developed as a potential single long-acting regimen for HIV-1. This molecule has the biophysical characteristics amenable for a self-administered subcutaneous weekly injection.

# Sub article: P023 Mono- and dual suppressive antiretroviral regimens in a real-life setting: the experience of Pitié-Salpêtrire HIV Centre

**Published:** 2016-10-23

**Authors:** Fabienne Caby, Rachid Agher, Roland Tubiana, Christine Blanc, Marie Jaspard, Yasmine Dudoit, Ruxandra Calin, Vincent Calvez, Anne Simon, Marc-Antoine Valantin, Christine Katlama

**Affiliations:** 1 Infectious Diseases, Pitié-Salpêtrière, INSERM U1136, Paris, France; 2 Virology Laboratory, Pitié-Salpêtrière, Paris, France; 3 Internal Medicine, Pitié-Salpêtrière, Paris, France

## Abstract

**Context**: Controlling viral replication with fewer drugs. Identifying light suppressive ART strategies – one-drug (1-DR) or two-drug (2-DR) regimen – has become a key issue in the long-term management of HIV-infected patients for various reasons: cumulative toxicity, unnecessary drugs and cost saving.

**Objectives**: To evaluate the ART regimen profile in patients with suppressed HIV viraemia in a single large HIV care and research centre in 2015.

**Methods**: All HIV-infected patients with a suppressed HIV-1 plasma viral load (pVL<50 copies/mL) in 2015 were included in this observational study through the NADIS computerised medical database which aims to describe suppressive ART profiles by comparing a 1-DR and a 2-DR to a standard triple-drug regimen (3-DR).

**Results**: Out of the 4129 HIV-infected patients for whom HIV RNA was available, 3807 (92%) had HIV RNA <50 copies/mL. The ART regimen consisted of a 1-DR in 140 patients (4%), a 2-DR in 710 patients (19%), a 3-DR in 2898 patients (77%) and ≥4-DR in 59 patients (1.5%). PIs were the most frequent single-drug regimen (69%). The 2-DR consisted of INSTI+NNRTI (40%), two NRTIs (13%) and NRTI+PI (11%). When compared to a 3-DR, patients with a 1- or 2-DR were older (p<0.001) with longer ART duration (p<0.0001) and those on a 2-DR had a lower nadir (p=0.006) (Table 1).

**Conclusion**: Mono- and dual therapies represent, in real life, over 20% of suppressive ART strategies in our centre. These options with fewer drugs warrant further large-scale investigation.

**Abstract P023–Table 1:** Suppressive ART strategies in Pitié-Salpêtrière Hospital in 2015

Median (%) IQR	1-drug regimen		2-drug regimen		3-drug regimen	
	n = 140 (4%)		n = 710 (19%)		n = 2898 (77%)	
**M/F (%)**	69% / 21%		68% / 32%		69% / 31%	
**Age**	52 [46–59]		53 [48–61]		49 [42–56]	
						
**ART regimen**			INSTI+ NNRTI	283 (40%)		
			RAL/NNRTI	193 (27%)		
			DTG/NNRTI	90 (13%)		
	DRV/r	97 (69%)	2 NRTI	94 (13%)	2NRTI + NNRTI	1240 (43%)
	DTG	24 (17%)	NRTI + PI/r	77 (11%)	2NRTI + INI	831 (29%)
	ATV/r	10 (7%)	INSTI + PI/r	66 (9%)	2NRTI + PI/r	630 (22%)
	LPV/r	9 (6%)	NRTI + INI	60 (8%)	2NRTI + PI	72 (2.5%)
			NRTI + PI/r	49 (7%)		
			Other	81 (11%)	Other	125 (4.4%)
						
**ART duration (3 years)**	16.8 [9–20]		18 [8–21]		10.7 [5–18]	
**Current regimen duration (mos)**	23 [10–54]		15 [7–39]		28.2 [12–61]	
**CD4/mm**^**3**^**(IQR)**	663 [525–824]		627 [474–828]		611 [449–805]	
**CD4 nadir/mm**^**3**^**(IQR)**	227 [159–319]		196 [93–319]		225 [109–341]	
**First line ART**	3 (2.1%)		53 (7.5%)		384 (13.3%)	
**Switch ART**	137 (98%)		657 (93%)		2514 (87%)	

n = 3748 patients.

# Sub article: P024 Safety and antiviral effect of Elpida (VM-1500), a novel NNRTI (+Truvada) in treatment-naïve HIV-1-infected patients at 24- to 48-week therapy

**Published:** 2016-10-23

**Authors:** Alexey Kravchenko, Elena Orlova-Morozova, Fiyara Nagimova, Oleg Kozirev, Tatyana Shimonova, Vadim Bichko, Natalya Vostokova, Olga Zozulya

**Affiliations:** 1 Russia AIDS Federal Centre, Moscow, Russian Federation; 2 Moscow Region AIDS Center, Moscow, Russian Federation; 3 Republic Tatarstan AIDS Center, Kazan, Russian Federation; 4 Volgograd Region AIDS Center, Volgograd, Russian Federation; 5 Moscow City AIDS Center, Moscow, Russian Federation; 6 Viriom, Moscow, Russian Federation; 7 IPHARMA, Moscow, Russian Federation

## Abstract

**Introduction**: In treatment-naïve patients, Elpida 20 and 40 mg QD (with TDF/FTC) at week 12 demonstrated potent antiviral activity, comparable to EFV, and favourable safety/tolerability profile. Elpida 20 mg QD was selected for study.

**Objective**: To evaluate safety and antiviral effect for treatment regimens with Elpida+TDF/FTC in comparison with EFV+TDF/FTC in treatment-naïve HIV-1 infected patients.

**Methodology**: A randomized, placebo-controlled, double-blind study in patients with HIV infection who are antiretroviral therapy-naïve with median of HIV-1 RNA 4.7–4.8 log10 copies/mL and CD4-lymphocytes – 349 to 379 cells/mm^3^. A total of 120 patients were randomized to Elpida (20 mg, group 1) or EFV (600 mg, group 2) with 1:1 ratio. All patients received TDF/FTC. Hundred percent of patients completed 24 weeks of treatment and 50% of patients completed 48 weeks of treatment.

**Results**: After 24 weeks of treatment, the fraction of patients with <50 HIV-1 RNA copies/mL in 1st gr. was 84.5% and 2nd gr. 66.7% (p=0.031, MTTI-analysis). At 48 weeks therapy - 93.3% and 83.5%, respectively. The median CD4-lymphocytes increased from 379 to 486 cells/mm^3^ (gr.1), from 349 to 491 cells/mm^3^ (gr.2) at 24 weeks treatment and to 549 cells/mm^3^ (gr.1) and to 510 cells/mm^3^ (gr.2) at 48 weeks. AEs (grade 1–4) were observed in 78.3% and 86.2% of patients from cohorts 1 and 2, respectively, including drug-related AEs (36.7% and 77.6%, respectively). For the CNS AEs, those numbers were 30% and 62.1% (p<0.001), including grade 3 to 4 AEs − 1.7% and 8.6%, respectively.

**Conclusions**: In treatment-naïve patients, Elpida 20 mg QD (with TDF/FTC) at 24 to 48 weeks demonstrated potent antiviral activity, comparable to EFV+TDF/FTC, and favourable safety/tolerability profile. Fewer drug-related AEs were observed for Elpida compared with EFV. The study will be completed at November 2016.

# Sub article: P025 The integrase strand transfer inhibitor bictegravir has a long integrase/DNA dissociation half-life

**Published:** 2016-10-23

**Authors:** Kirsten White, Anita Majka, Nikolai Novikov, Michael Miller, Manuel Tsiang

**Affiliations:** 1 Biology, Gilead, Foster City, CA, USA

## Abstract

**Introduction**: The HIV integrase strand transfer inhibitor (INSTI) bictegravir (formerly GS-9883) has a high barrier to resistance selection in vitro [1]. Bictegravir has an improved in vitro resistance profile for most HIV isolates with resistance to the other INSTIs raltegravir, elvitegravir and dolutegravir [1,2]. The apparent dissociation rate constant of dolutegravir from integrase/DNA complexes was previously shown to be longer than raltegravir and elvitegravir and was predicted to correlate with potent antiretroviral activity and a higher genetic barrier to resistance [3]. Here, the dissociation kinetics of bictegravir was evaluated.

**Methods**: The apparent association and dissociation kinetics of 3H-labelled INSTIs raltegravir, elvitegravir, dolutegravir and bictegravir were measured using wild-type HIV integrase/DNA complexes and a scintillation proximity assay as previously described [3]. Single exponential decay functions were used to analyze both the binding and competition binding phases yielding apparent association doubling times (t_d_) and dissociation half-lives (t_1/2_). However, the competition binding phases deviated significantly from the single exponential decay function due to the gradual sedimentation of the SPA beads, necessitating modelling of the equilibrium binding with on- and off-rate constants as decreasing functions of time with k_on_ and k_off_ as initial values of each function, respectively.

**Results**: The INSTIs bictegravir, dolutegravir, elvitegravir and raltegravir showed rapid association with integrase/DNA complexes with apparent association doubling times (t_d_) ranging from 14 to 34 minutes: elvitegravir (14±4 minutes), raltegravir (22±11 minutes), bictegravir (31±4 minutes) and dolutegravir (34±1 minutes). The apparent dissociation half-lives (t_1/2_) of INSTIs from integrase/DNA complexes ranged from 3.6 to 122 hours: elvitegravir (3.6±0.9 hours), raltegravir (15±2 hours), dolutegravir (71±13 hours) and bictegravir (122±14 hours); p=0.0018 for bictegravir versus dolutegravir. The initial values of the dissociation rate constants (k_off_) were determined using the model and converted to a t_1/2_ which may be more representative of the actual t_1/2_: elvitegravir (1.6±0.2 hours), raltegravir (5.4±0.4 hours), dolutegravir (11±2 hours), and bictegravir (35±19 hours); p=0.046 for bictegravir versus dolutegravir.

**Conclusions**: The (t_1/2_) of bictegravir is the longest reported for any INSTI that is approved or in development. Long residence times of INSTIs on the integrase/DNA complex have been correlated with potent antiretroviral activity against wild-type HIV-1 integrase and a high barrier to resistance in vitro [3]. The barrier to clinical resistance for bictegravir is being assessed in ongoing phase 3 studies with the once-daily, unboosted bictegravir/emtricitabine/tenofovir alafenamide single-tablet regimen.

## References

[1] Jones G, Goldsmith J, Mulato A, White K, Hansen D, Stray K A Novel HIV-1 Integrase Strand Transfer Inhibitor (INSTI) with optimized in vitro resistance profile. Poster 413. ASM Microbe. 2016 June 16.

[2] White K, Cihlar T, Miller MD Potent activity of bictegravir (BIC; GS-9883), a novel unboosted HIV-1 Integrase Strand Transfer Inhibitor (INSTI), against patient isolates with INSTI-resistance.

[3] Hightower KE, Wang R, DeAnda F, Johns BA, Weaver K, Shen Y (2011). Dolutegravir (S/GSK1349572) exhibits significantly slower dissociation then raltegravir and elvitegravir from wild-type and integrase inhibitor-resistance HIV-1 integrase-DNA complexes. Antimicrob. Agents Chemother.

# Sub article: P026 Durability and prescribing patterns of initial HIV regimens in treatment-naïve patients

**Published:** 2016-10-23

**Authors:** Ellen Eaton, Ashutosh Tamhane, Girish Prajapati, Bridgett Goodwin, Michael Saag

**Affiliations:** 1 Medicine, Infectious Diseases, University of Alabama, Birmingham, AL, USA; 2 Center for Observational Research and Real-world, Evidence, Merck & Co., Inc., Rahway, NJ, USA; 3 Center for Observational Research and Real-world E, Merck & Co., Inc., Upper Gwynedd, PA, USA

## Abstract

**Introduction**: Literature on ARV regimen durability (persistency) in real-world settings is outdated owing to the introduction of new drug classes and combinations in recent years. We evaluated the composition and durability of contemporary ARV regimens prescribed for treatment-naïve patients in a clinical setting.

**Methods**: Treatment-naïve HIV-infected patients who initiated ART between January 2007 and January 2016 at the HIV clinic affiliated with the University of Alabama, Birmingham, were included. Data on all initial ARV compositions and durations were extracted from the electronic medical record with administrative censoring on 8 June 2016. Manual abstraction was performed to confirm ARV regimen start and stop dates. ARV regimen durability (time to discontinuation) was estimated using Kaplan-Meier survival curves that incorporate censoring and its association with various characteristics by Cox proportional hazard analyses.

**Results**: Among 589 patients (mean age, 37 years; 79% male; 65% African American), efavirenz/emtricitabine/tenofovir (193, 33%) was the most commonly prescribed initial ARV regimen (Table 1). Median durability of all initial ARV regimens was 45 months (95% CI 41–51). The regimen was discontinued in 332 (56%) patients and a majority of them (203, 61%) had an undetectable viral load at the time of discontinuation. A decrease in durability of ARV regimens was found in more recent years (Table 1, p=0.046). After adjusting for various covariates in multivariable analysis, patients initiating ART from 2010 to 2012 (aHR 1.4, 95% CI 1.1–1.8; p=0.02) and 2013 to 2015 (aHR 1.5, 95% CI 1.1–2.1; p=0.01) were more likely to discontinue ART than those initiating from 2007 to 2009.

**Conclusions**: Overall durability of most ARV regimens in our cohort was almost 4 years. Two multi-tablet regimens and two regimens recently removed from US first-line treatment guidelines were quite durable. Decreased durability of ARV regimens occurring in more recent years appears to be due to patient and provider preferences for newer regimens and not due to virologic failure.

**Abstract P026–Table 1:** Summary of median durability of ARV regimen by regimen composition and year of initiation in treatment-naïve HIV patients initiating care at an academically affiliated HIV clinic

ARV regimen composition	Treatment share N (%)	Median durability, months (95% CI)
Efavirenz/emtricitabine/tenofovir	193 (33)	59 (47–63)
Elvitegravir/cobicistat/emtricitabine/tenofovir	115 (20)	* (34–*)
Rilpivirine/emtricitabine/tenofovir	55 (9)	48 (26–*)
Raltegravir/emtricitabine/tenofovir	42 (7)	50 (28–66)
Atazanavir/ritonavir/emtricitabine/tenofovir	31 (5)	27 (19–76)
Darunavir/ritonavir/emtricitabine/tenofovir	27 (5)	44 (36–*)
Dolutegravir/emtricitabine/tenofovir	13 (2)	7 (3–*)
Dolutegravir/abacavir/lamivudine	7 (1)	26 ( *)
Other	106 (18)	32 (26–36)
Year of ARV regimen initiation	Treatment share N (%)	Median durability, months (95% CI)
2007–2009	177 (30)	57 (45–66)
2010–2012	200 (34)	41 (34–49)
2013–2015	212 (36)	38 (32–*)

* not estimable.

# Sub article: P027 Integrase inhibitor-based antiretroviral therapy in vulnerable populations

**Published:** 2016-10-23

**Authors:** Brian Conway, Ghazaleh Kiani, Rajvir Shahi, Tyler Raycraft, Arpreet Singh, Syune Hakobyan, Arshia Alimohammadi

**Affiliations:** 1 Clinical Research, Vancouver Infectious Diseases Centre, Vancouver, Canada

## Abstract

**Introduction**: Current treatment guidelines favour the use of integrase inhibitor (II)-based regimens in the majority of settings where ART is required, based on the results of clinical trials demonstrating the superiority of such approaches. Vulnerable inner-city populations have often been excluded from clinical trials of these agents. There is a need to generate data to better inform the applicability of treatment guidelines in these populations.

**Methods**: We have conducted a retrospective analysis of the database of a large clinic catering to HIV-infected patients with a high prevalence of people who inject drugs (PWID). We have abstracted records of subjects receiving II-based therapy and evaluated response to therapy. Demographic and clinical correlates of success were evaluated, with a view to comparing the relative efficacy of raltegravir (RAL), elvitegravir (ELV) and dolutegravir (DOL)-based regimens.

**Results**: A total of 247 patients received IIs (141 RAL, 68 ELV, 38 DOL). Baseline characteristics include: 85.8% male, 38.5% intravenous drug users, 5.3% previously treatment naïve, 45.2% HCV co-infected, and 13.6% on opiate substitution therapy. Median baseline CD4 count and plasma viral load were 410 (range 30–1380) cells/mm^3^ and 43 (range <40–300,000) copies/mL. After a median follow up of 44 (3–141) months, virologic suppression was achieved in 93.0%/86.7%/97.3% patients on RAL/ELV/DOL with most current median CD4 count of 555 (range 60–1700) cells/mm^3^. No treatment-limiting toxicity was observed and response rates in PWID (80.7%/66.7%/90.0%) were equivalent to those observed in non-PWID.

**Conclusion**: II-based therapies are as effective in “real life” and in PWID as they have been reported to be in clinical trials, justifying their selection as regimens of choice for all patients. Medium- and long-term efficacies of all three agents in this class are comparable, and the selection of one agent over another should be based on other criteria than virologic potency and tolerability.

# Sub article: P028 Inhibition of HIV-1 protease and plasmodium falciparum by a modified digold chloroquine derivative

**Published:** 2016-10-23

**Authors:** Ntombenhle Gama, Kamlesh Kumar, Janette Reader, Bhavna Gordhan, Lyn-Marie Birkholtz, Bhavesh Kana, James Darkwa, Debra Meyer

**Affiliations:** 1 Biochemistry, University of Pretoria, Pretoria, South Africa; 2 Chemistry, University of Johannesburg, Johannesburg, South Africa; 3 DST/NRF Center of Excellence for Biomedical TB Res, Wits University, Johannesburg, South Africa; 4 Dean's Office, University of Johannesburg, Johannesburg, South Africa

## Abstract

**Introduction**: HIV-1 remains a major threat to public health [1]. Sub-Saharan Africa has the highest prevalence of HIV/AIDS and the overlap of this infection with other major pandemics that plague the region has become a major challenge in the treatment of the infection. These include tuberculosis and malaria [2]. Although HAART has been successful in the treatment of the virus, these drugs are associated with a number of adverse effects often resulting in non-compliance by the patients and increasing an individual's susceptibility to these opportunistic infections that are prevalent in the region [3]. It is therefore crucial to develop novel treatments that are not only effective against HIV, but opportunistic infections as well. Indeed, multi-target drugs are becoming increasingly popular with the rise in syndemic diseases in most parts of the world. Finding novel drugs that are effective against two or more different infections or diseases, targeting various pathways within the microorganism / pathology is where research is heading, and in this study metal modified chloroquine (Complex 1) was investigated in a type of repurposing approach for this known malaria drug [4].

**Materials and methods**: Complex 1 was synthesized and purified in good yield [5]. The complex was screened for inhibition of the enzyme HIV-1 protease using a recombinant enzyme (Bachem, Switzerland) and a fluorescent substrate (Sigma Aldrich, USA). The complex was also evaluated against the drug-susceptible strain of *M. tuberculosis*, H37Rv (ATCC27264) and the 3D7 strain of *P. falciparum*. Its effects on cell viability were assessed on TZM-bl cells using a tetrazolium dye and confirmed by real-time cell analysis (RTCA).

**Results**: Complex 1 showed HIV-1 protease inhibition values of above 50% at 25 µg/mL. The complex showed remarkable inhibition of *M. tuberculosis* with a minimal inhibitory concentration of 5 µM after 14 days of incubation with the bacterium. The IC50 of 1 on *P. falciparum* was 0.593 µM, and the CC50 of the complex on TZM-bl cells was 24.34±0.68 µg/mL. RTCA showed non-toxicity of the complex at all tested concentrations, with treatment profiles similar to those produced by untreated cells.

**Conclusion**: A complex with inhibitory abilities against HIV-1 replication, *M. tuberculosis* and *P. falciparum* is presented here. Tuberculosis and malaria play a major role in the mortality of HIV-infected patients, and the development of drugs with dual activities that can control both the viral and opportunistic infections could contribute to the alleviation of the fatal HIV prognosis.

## References

[1] World Health Organisation (WHO) (2014). World Health Statistics Report.

[2] Canaday DH, Wu M, Lu S, Aung H, Peters P, Baseke J (2009). Induction of HIV type 1 expression correlates with T cell responsiveness to mycobacteria in patients coinfected with HIV type 1 and Mycobacterium tuberculosis. AIDS Res Hum Retroviruses.

[3] Luma HN, Doulla M-S, Choukem S-P, Temfack E, Ashuntantang G, Joko HA (2012). Adverse drug reactions of Highly Active Antiretroviral Therapy (HAART) in HIV infected patients at the General Hospital, Douala, Cameroon: a cross sectional study. Pan Afr Med J.

[4] Oprea TI, Mestres J (2012). Drug repurposing: far beyond new targets for old drugs. AAPS J.

[5] Gama N, Kumar K, Ekengard E, Haukka M, Darkwa J, Nordlander E (2016). Gold(I) complex of 1,1’-bis(diphenylphosphino) ferrocene-quinoline conjugate: a virostatic agent against HIV-1. Biometals.

# Sub article: P029 Treatment failure of chronic HCV infection with the new direct-acting antivirals: experience of a Portuguese central hospital

**Published:** 2016-10-23

**Authors:** Karen Pereira, Sara Dias Grazina, Ana Cláudia Miranda, Teresa Baptista, Fernando Borges, Jaime Nina, Susana Peres, Isabel Aldir, Maria José Campos, Isabel Antunes, João Pereira, Fernando Ventura, Kamal Mansinho

**Affiliations:** 1 Hospital de Egas Moniz, Centro Hospitalar de Lisboa Ocidental, EPE, Serviço de Doenças Infecciosas e Medicina Tropical, Lisbon, Portugal; 2 Hospital de Egas Moniz, Centro Hospitalar de Lisboa Ocidental, EPE, Serviço de Urgência Geral, Lisbon, Portugal; 3 Hospital de Egas Moniz, Centro Hospitalar de Lisboa Ocidental, EPE, Serviço de Medicina Interna, Lisbon, Portugal

## Abstract

**Introduction**: Since the emergence of new direct-acting agents, hepatitis C (HCV) treatment has undergone a rapid evolution, bringing about a radical change in the clinical paradigm. Oral treatment regimens with high virologic efficacy, great tolerability, favourable safety profiles, high genetic barriers, and an easy posology are now available. Despite this, challenges remain to patients who fail the treatment. This study aims to characterize this group of patients in an infectious diseases clinic in Lisbon.

**Methods**: Retrospective observational study of a cohort of HCV chronically infected patients with or without HIV infection, given new direct-acting agents (DAAs), from 1 January 2015 to 30 April 2016. Demographic, epidemiologic, clinical and laboratory data were collected. Statistical analysis was performed using Microsoft Office^®^ Excel 2012.

**Results**: During the analysis period, 426 patients were eligible to start HCV treatment with DAA regimens (138 HCV mono-infected and 288 HIV co-infected). Two hundred and nineteen patients have concluded it (56 HCV mono-infected and 163 HIV co-infected), of whom 134 patients had their viral load evaluated 12 weeks after treatment ended: sustained viral response in 126 patients (94%) and detectable viral load in eight patients (6%). In the latter, all patients were male with a mean age of 51 years. Mean time of HCV diagnosis was 12 years. Patients mainly acquired the infection through parenteric drug use (75%). Seven were HIV co-infected. Regarding genotype characterization, the most common was genotype 1a (50%). Evaluation of IL28B polymorphism revealed CC predominance (50%). At baseline, mean HCV RNA was 7,103,018 IU/mL. Real-time elastography data, using METAVIR score, revealed a fibrosis F2 in one patient, F2/F3 in three patients and F3 in four patients. Concerning previous treatment for HCV, six were treatment experienced, of whom five were null responders. The most requested treatment was sofosbuvir/ledipasvir (63%). Five patients were proposed for 12 weeks and three for 24 weeks of treatment, for which all had a good adherence. One patient died at the end of treatment. The others are waiting for retreatment options.

**Conclusions**: In this preliminary analysis, the eight patients with non-SVR12 were male and had HIV infection. These factors may be associated with response and outcome with the new direct-acting agents. Regarding the efficacy of these drugs, uncertainties and challenges remain, in addition to continued necessity of identifying response predictive factors and individual strategies for special patient groups.

# Sub article: P031 Genetic variants in CYP2B6 and CYP2A6 explain interindividual variation in efavirenz plasma concentrations in routine care of HIV-infected children with diverse ethnic origin

**Published:** 2016-10-23

**Authors:** Sandra Soeria-Atmadja, Emma Österberg, Lars Gustafsson, Marja-Liisa Dahl, Jaran Eriksen, Johanna Rubin, Lars Navér

**Affiliations:** 1 Division of Pediatrics, CLINTEC, Karolinska Institutet, Stockholm, Sweden; 2 Division of Clinical Pharmacology, Deparmentt of Laboratory Medicine, Karolinska Institutet, Stockholm, Sweden

## Abstract

**Introduction**: Approximately 2.6 million children live with HIV globally, and efavirenz (EFV) is one of the most widely used antiretroviral agents for HIV treatment in children and adults. There are concerns about the appropriateness of current EFV dosing, and it has been discussed whether EFV dosing should be adapted according to genotype in children as suggested for adults. The aim of the study was to investigate if paediatric EFV dosing should be guided by genetic variation in drug-metabolizing enzymes rather than by body weight only.

**Materials and methods**: EFV plasma concentrations measured for clinical purposes from all children (<18 years old) at Karolinska University Hospital, Stockholm, Sweden, treated with EFV were collected retrospectively. They were genotyped for 11 polymorphisms in genes coding for drug-metabolizing enzymes and P-glycoprotein, of potential importance for EFV disposition. Data on origin, sex, age, weight, HIV RNA, viral resistance patterns, CD4 cells, adherence to treatment, subjective health status and adverse events were collected.

**Results**: Thirty-six patients and 182 (mean 5 samples/patient) EFV plasma concentration measurements were included. EFV plasma concentration varied 21-fold between measurements (n=182) (2.7–61 µmol/L) and 9-fold measured as mean EFV plasma concentration across the subjects (4.9–42.4 µmol/L). A multivariate mixed-effects REML regression model, including multiple gene polymorphisms, identified CYP2B6*6 T/T (p<0.0005), CYP2B6*11 G/G (p<0.0005), CYP2A6*9 A/C (p=0.001) genotypes, age at treatment initiation (p=0.002) and time from treatment initiation (p<0.0005) as independent factors significantly related to log_e_ concentration/ (dose/weight). The contribution of the studied gene polymorphisms to the intra- and interindividual variation were 6% and 75%, respectively (Bryk/Raudenbush R-squared level). Asian origin was significantly related to lower log_e_ mean concentration/ (dose/weight) compared to African (p=0.0085) and Hispanic origin (p=0.038).

**Conclusions**: Genetic polymorphisms in CYP2B6 and CYP2A6 explained a significant proportion of variability in EFV plasma concentration and Asian origin gave substantially lower plasma concentration in HIV-infected children in a multi-ethnic outpatient clinic. Knowledge about individual variants in key drug-metabolizing enzymes could improve clinical safety and be a way to achieve more predictable EFV plasma concentrations in HIV-infected children.

# Sub article: P032 Relative bioavailability and food effect of a paediatric dispersible tablet formulation of the non-nucleoside reverse transcriptase inhibitor (NNRTI) rilpivirine (RPV)

**Published:** 2016-10-23

**Authors:** Herta Crauwels, Nico Goyvaerts, Simon Vanveggel, Rodica Van Solingen

**Affiliations:** 1 Janssen Infectious Diseases BVBA, Beerse, Belgium

## Abstract

**Introduction**: The NNRTI RPV is approved in several countries as a 25 mg once-daily tablet in combination with other antiretrovirals for treating HIV-1-infected, treatment-naïve patients aged ≥12 years with a viral load ≤100,000 copies/mL. Further evaluation of the paediatric use of RPV, including a dose evaluation, is ongoing in children aged ≥6 to <12 years in cohort 2 of the PAINT study (NCT00799864). The current trial evaluated the relative bioavailability and food effect of an age-appropriate RPV formulation for use in children, the RPV dispersible tablet formulation (2.5 mg RPV).

**Methods**: Open-label, randomized, crossover study in two panels of 16 healthy adults each (NCT02561936). In panel 1, participants received a single 25 mg dose of RPV administered as the Edurant^®^ tablet (reference 1) or as 10 dispersible 2.5 mg tablets (dispersed in water), in fed conditions (standardised breakfast). In panel 2, participants received a single 25 mg dose of RPV administered as 10 dispersible 2.5 mg tablets (dispersed in water) in fed conditions (reference 2) or in fasted conditions, or dispersed in orange juice in fed conditions. In each panel, there was a 14-day washout in between treatments. Plasma samples (over 168 hours after dosing) were analyzed for RPV using a validated LC-MS/MS method (LLOQ 1.00 ng/mL). RPV pharmacokinetic parameters were determined using non-compartmental analysis. Least square means and associated 90% confidence intervals of treatment ratios (test/reference) were calculated based on log-transformed pharmacokinetic parameters. Safety and tolerability were assessed throughout the study.

**Results**: Table 1 summarizes the statistical results. The RPV exposure (AUC, C_max_) with the dispersible tablet was 21 to 33% higher than the reference tablet (Edurant^®^), in fed conditions. When taken fasted, the RPV exposure with the dispersible tablet was 28 to 34% lower compared with fed conditions. Dispersion in orange juice (acidic beverage) compared with water increased the RPV exposure by 11 to 14%, in fed conditions. One participant discontinued early before dosing in the last session in panel 1 for a grade 3 adverse event (bronchitis), considered not related to RPV. There were no other grade 3 or 4 adverse events and no serious adverse events. Administration of RPV as the dispersible tablet formulation was generally well tolerated in fed and fasted conditions.

**Conclusions**: A RPV dispersible tablet with good bioavailability was developed for potential use in the ongoing paediatric trial in HIV-infected children aged <12 years. Consistent with the Edurant^®^ tablet formulation, intake of the dispersible tablet with a meal improved the bioavailability.

**Abstract P032–Table 1:** Statistical analysis summary

Panel	Test	Reference	N/N	C_max_	AUC_last_	AUC_inf_
1	Dispersible tablet, fed	Edurant^®^, fed	15/16^a^	1.28 (1.14–1.43)	1.21 (1.10–1.34)	1.33 (1.15–1.53)
2	Dispersible tablet, fasted	Dispersible tablet, fed	16/16^b^	0.66 (0.56–0.77)	0.72 (0.63–0.82)	0.69 (0.57–0.83)
	Dispersible tablet, fed (dispersed in orange juice)	Dispersible tablet, fed (dispersed in water)	16/16^c^	1.11 (0.96–1.30)	1.14 (1.00–1.30)	1.12 (0.94–1.35)

a N/N: 11/9 for AUC_inf_

b N/N: 11/11 for AUC_inf_

c N/N: 12/11 for AUC_inf_.

Data was presented as test/reference least square mean ratio (90% CI). N/N: number of participants in the test/reference; one volunteer discounted before trial completion. C_max_=maximum plasma concentration; AUC_last_=area under the plasma concentration-time curve (AUC, calculated by linear = linear trapezoidal summation) from time of administration up to the last timepoint with a measurable concentration post-dose; AUC_inf_=AUC from time of administration to infinity.

# Sub article: P033 Predictors of plasma HIV RNA suppression in a cohort of perinatally HIV-infected individuals

**Published:** 2016-10-23

**Authors:** German Contreras, Gilhen Rodriguez, Gabriela Del Bianco, Norma Perez, Laura Benjamins, James Murphy, Gloria Heresi

**Affiliations:** 1 Pediatric Infectious Diseases, University of Texas McGovern Medical School, Houston, TX, USA; 2 Adolescence Medicine, University of Texas McGovern Medical School, Houston, TX, USA

## Abstract

**Introduction**: Knowledge of factors associated with viral suppression (VS) among HIV-infected children and adolescents is central to reduce transmission and improved health outcomes.

**Materials and methods**: We included individuals attending the UT Health Paediatric HIV Clinic who were ART naïve and subsequently started on cART, followed for ≥1 year from the start of cART and maintained on the same cART regimen during this interval. VS was defined as maintenance of HIV RNA <400 copies/mL for 1 year after initial VS was achieved. Cumulative HIV RNA was defined as the overall HIV replication burden in an individual. Median rates of VS were calculated using Kaplan–Meir (KM). A Cox regression model was used to determine predictors of VS.

**Results**: Thirty perinatally HIV-infected children were included. The study population was mainly black (76.7%) and male (56.7%); 22 individuals (73.3%) were enrolled into clinical care within the 12 months of birth. The median age at cART initiation was 5.1 months (IQR 2.7–27.3). The study population had a CD4 percentage of 33 (24–39), a plasma HIV RNA log10 copies/mL of 5.5 (4.8–5.9) and a cumulative HIV RNA copy-years/mL of 7.1 (6.8–7.7) at the time of cART initiation. The median time from cART initiation to VS was 4.4 months (KM estimate). Time to VS was markedly shorter for those who did not miss a scheduled clinical appointment compared with those who missed at least one scheduled clinical appointment (4.4 vs. 22.9 months). Time to VS was shorter for Hispanics compared with black non-Hispanics (3.7 vs. 10.7 months) and for those living inside the Houston beltway 610 (2.6 vs. 10.7 months) than those living outside the beltway 610. The adjusted Cox analysis showed that there was a lower rate of VS for each log10 of increase in cumulative HIV RNA (HR 0.11; 95% CI 0.02–0.81; p=0.023) and for each month of delay in initiating routine HIV care (HR 0.87; 95% CI 0.78–0.98; p=0.01). Individuals born after 2003 had higher rates of VS per unit of time (HR 158.11; 95% CI 2.88–8718.54; p=0.013).

**Conclusions**: Early initiation and sustained enrolment in clinical care plays a paramount role for successful treatment of perinatally HIV-infected children and adolescents. Cumulative HIV RNA offers a robust predictive value for detecting individuals at risk of not reaching VS.

# Sub article: P034 Characteristics and outcome of HIV-positive children internationally adopted in France

**Published:** 2016-10-23

**Authors:** Olivier Lesens, Stéphane Blanche, Violaine Corbin, Christine Jacomet

**Affiliations:** 1 Infectious Diseases, Centre Hospitalier Universitaire de Clermont-Ferrand, Clermont-Ferrand, France; 2 Unité d'Immunologie, AP-HP, Hôpital Necker-Enfants Malades, Hématologie et Rhumatologie p, Paris, France

## Abstract

**Introduction**: HIV-positive children comprise a larger percentage of the children eligible for international adoption [1]. Little is known about the clinical, immunologic and viral outcomes of these children.

**Materials and methods**: Twenty-five medical centres agreed to participate. Children living in France and internationally adopted between 1 January 2005 and 1 January 2016 were included after informed consent. Socio-demographic, medical and biologic variables were collected during the first medical evaluation in France and 6 months later. The yearly percentage of HIV-positive adoptees was calculated among new adoptees or new HIV-positive children diagnosed.

**Results**: Of the 25 medical centres that agreed to participate, 14 gave care to at least one HIV-positive adoptee. Forty-one HIV-positive adoptees were included (female: 56%; median age at arrival: 3.91 years). The majority came from East Asia. HIV-positive adoptees represented about half of newly diagnosed HIV-positive children in 2014 versus less than 20% the preceding years. They represented also about 5% of new adoptees in 2014 versus about 1% the previous years. For three children, a new diagnosis of latent chronic hepatitis B, cured hepatitis B and chronic active hepatitis C was made at arrival in France. Other clinical diagnoses made at the first consult were benign diseases, mainly skin diseases. The mean CD4 percentage was 32.8±9% (range 13–49%). Only one child had a CD4 percentage below 15%. Forty percent had a detectable viral load (VL) >20 copies/mL at arrival. Among those, resistance to NRTIs was documented in 10%, resistance to NNRTIs in 12.5% and resistance to PIs in 2.4%. Thirty-four children received ART in their country of origin. Among those, 24 continued on the same ARV in France. At 6 months, the mean CD4 percentage was 35.6±8%, and the VL was still detectable in 29% children. Of them, one acquired resistance to NRTI and NNRTI during the 6 months of follow-up.

**Conclusions**: An increasing number of HIV-infected children have been internationally adopted in France since 2005. The immune status was good but detectable VL was frequent at arrival and at 6 months. It can be suspected that adoptive parents will face difficulties to maintain enough adherence to ART in the long term, especially during adolescence [2]. Prolonged support from healthcare providers is needed to face this difficult challenge that combines the management of adoption and HIV disease [3].

## References

[1] Ampofo K (2013). Infectious disease issues in adoption of young children. Curr Opin Pediatr.

[2] Pursuing Later Treatment Options II (PLATO II) project team for the Collaboration of Observational HIV Epidemiological Research Europe (COHERE) (2011). Risk of triple-class virological failure in children with HIV: a retrospective cohort study. Lancet.

[3] Sciauvaud J, Rigal E, Pascal J, Nourrisson C, Poirier P, Poirier V (2014). Transmission of infectious diseases from internationally adopted children to their adoptive families. Clin Microbiol Infect.

# Sub article: P035 Efficacy of dolutegravir/abacavir/lamivudine (DTG/ABC/3TC) fixed-dose combination (FDC) compared with ritonavir-boosted atazanavir (ATV/r) plus tenofovir disoproxil fumarate/emtricitabine (TDF/FTC) in treatment-naïve women with HIV-1 infection (ARIA study): subgroup analyses

**Published:** 2016-10-23

**Authors:** Margaret Johnson, Caroline Gatey, Weeraweet Manosuthi, Adriano Lazzarin, Daniel Podzamczer, Choy Man, Alicia Aylott, Annie Buchanan, Brian Wynne, Cindy Vavro, Michael Aboud, Kim Smith

**Affiliations:** 1 Royal Free Hospital, Centre for HIV Medicine, London, UK; 2 Service des Maladies Infectieuses et Tropicales, Hôpital Saint Louis, Paris, France; 3 Department of Medicine, Bamrasnaradura Institute, Nonthaburi, Thailand; 4 Dipartimento di Malattie Infettive, IRCCS Ospedale San Raffaele, Milan, Italy; 5 HIV Unit, Infectious Disease Service, Ciudad Sanitaria y Universitaria de Bellvitge, Barcelona, Spain; 6 Clinical Sciences, ViiV Healthcare, Durham, NC, USA; 7 Clinical Statistics, GlaxoSmithKline, London, UK; 8 Clinical Sciences, ViiV Healthcare, Collegeville, PA, USA; 9 Clinical Virology, ViiV Healthcare, Durham, NC, USA; 10 Global Medical Sciences, ViiV Healthcare, London, UK; 1 Global Medical Strategy, ViiV Healthcare, Durham, NC, USA

## Abstract

**Introduction**: Built around an unboosted integrase-strand transfer inhibitor (INSTI), the FDC of DTG/ABC/3TC offers a complete regimen for treatment of HIV-1 infection, with good tolerability and a high barrier to resistance. To gain additional data for women on this regimen, we conducted ARIA, an international, randomized, open-label study to evaluate the safety and efficacy of DTG/ABC/3TC versus ATV/r+FTC/TDF (ClinicalTrials.gov: NCT01910402).

**Materials and methods**: Treatment-naïve adult women, with HIV-1 RNA ≥500 copies/mL, were randomized 1:1 to 48 weeks of treatment with DTG/ABC/3TC or ATV/r+FTC/TDF once daily. The primary endpoint was the proportion of women achieving an HIV-1 RNA <50 copies/mL at week 48 (snapshot algorithm). Women who became pregnant were withdrawn and were possibly offered entry into a DTG/ABC/3TC pregnancy study. Additional analyses were performed to evaluate efficacy based on geographic region and baseline characteristics.

**Results**: Four hundred and ninety-five women were randomized and treated. Subjects were well matched for demographic and baseline characteristics. Median age was 37 years; 45% of the subjects were white and 42% African heritage. Overall results showed that DTG/ABC/3TC was superior to ATV/r+FTC/TDF, with 82% and 71%, respectively, achieving HIV-1 RNA <50 copies/mL at week 48 (adjusted difference 10.5%, 95% CI 3.1–17.8%, p=0.005). Differences were driven by lower rates of both discontinuations due to adverse events (AEs) and snapshot virologic non-response in the DTG/ABC/3TC group. In subgroup analyses conducted based on region and baseline characteristics, higher response rates were consistently observed in the DTG/ABC/3TC group compared to ATV/r+TDF/FTC group (Table 1). There were fewer drug-related AEs and fewer withdrawals due to AEs in the DTG/ABC/3TC group. There were no treatment-emergent primary INSTI or ABC/3TC resistance mutations in the DTG/ABC/3TC group.

**Conclusions**: DTG/ABC/3TC demonstrated superior efficacy and a favourable safety profile compared to ATV/r+FTC/TDF in treatment-naïve women, after 48 weeks of treatment. Subgroup analyses performed based on baseline characteristics and geographic region were consistent with overall results.

**Table 1:** Proportion of subjects with HIV-1 RNA <50 copies/mL (snapshot)

	Subgroup (n)	DTG/ABC/3TC, N = 248 (%)	ATV/r + TDF/FTC, N = 247 (%)
Overall		82	71
Age (years)	<50 (424)	80	71
	≥50 (71)	92	74
CD4 + cell count (cells/mm3)	≤350 (253)	85	72
	>350 (242)	78	71
	≤200 (113)	81	69
	>200 (382)	82	72
Baseline HIV-1 RNA (copies/mL)	≤100,000 (360)	83	74
	>100,000 (135)	80	64
HIV-1 subtype	B (206)	80	69
	Non-B (271)	84	73
Geographic region	Western Europe (131)	83	71
	US and Canada (153)	77	70
	Other (211)	85	72

# Sub article: P036 Sex and gender differences in rilpivirine-based ART: data from the HIV Center Frankfurt

**Published:** 2016-10-23

**Authors:** Gundolf Schüttfort, Kathrin Philipp, Hans-Reinhard Brodt, Philipp de Leuw, Siri Göpel, Eva Herrmann, Pavel Khaykin, Christoph Stephan, Timo Wolf, Annette Haberl

**Affiliations:** 1 Infectious Diseases, University Hospital Frankfurt, Frankfurt am Main, Germany; 2 Department for Biostatistics, University Hospital Frankfurt, Frankfurt am Main, Germany; 3 MainFachArzt, Outpatient Center, Frankfurt am Main, Germany

## Abstract

**Introduction**: Rilpivirine is a second-generation once-daily non-nucleoside reverse transcriptase inhibitor (NNRTI), which has shown high overall response rates in treatment-naïve patients without sex- and gender-specific differences in clinical trials [1–3]. Sex- and gender-specific data in treatment-experienced patients receiving a rilpivirine-based regimen are still limited. We conducted a 48-week efficacy and safety analysis in treatment-naïve and treatment-experienced men and women using retrospective data from the University Hospital Frankfurt HIV Center.

**Materials and methods**: Between March 2011 and December2015, all patients from the HIV Center of the University Hospital Frankfurt receiving a rilpivirine-based regimen were analyzed in this retrospective observational study. The primary endpoint was the proportion of patients with any discontinuation of a rilpivirine-based therapy at week 48. Furthermore, virologic response rates (FDA snapshot analysis; HIV-1 RNA <50 copies/mL) were assessed at week 48.

**Results**: A total of 188 patients (33% female) were included in the analysis. Seventy-four percent were treatment experienced and 26% were treatment naïve (Table 1). Regarding sex differences, the proportion of discontinuations was significantly higher in women than in men (23% vs. 12%; p=0.028; odds ratio 2.24; CI 1.02–4.91). There was no significant sex difference regarding discontinuations between treatment-experienced and treatment-naïve patients (25% vs. 16% and 23% vs. 12%, respectively). Virologic response rates (FDA snapshot analysis; HIV-1 RNA <50 copies/mL) were assessed at week 48 and revealed a higher rate of discontinuations due to virologic failure in Caucasian women versus non-Caucasian women (36% vs. 16%; p=0.071; odds ratio 1.33; CI 0.20–8.71).

**Conclusions**: While overall response rates to rilpivirine-based regimens were high for both treatment-experienced and treatment-naïve patients, the proportion of discontinuations was significantly higher in female patients. The total number of patients with virologic failure was low (16%); race appeared to influence the efficacy of a rilpivirine-based ART as Caucasian women showed a higher rate of virologic failure than non-Caucasian women. Therefore, it should be an interdisciplinary approach to identify and reduce possible barriers for successful antiretroviral treatment in non-Caucasian female HIV-positive patients.

**Abstract P036–Table 1:** Baseline parameters

	Females (n = 63)		Males (n = 125)	
		
	Treatment experienced	Treatment naïve	Treatment experienced	Treatment naïve
	
Variable	(n = 49)	(n = 14)	(n = 90)	(n = 35)
Age (years: mean±SD)	41±9	45±11	51±11	37±11
Range (years)	34–46	35–55	41–58	30–46
Race (n Caucasian/non-Caucasian)	18/31	4/10	85/4	24/12
(% Caucasian/non-Caucasian)	36.7/63.3	28.6/71.4	94.4/5.6	68.6/31.4
CD4/µl (mean±SD)	562±290	386±149	600±251	449±188
CD4% (mean±SD)	27.6±10.8	21.4±7.9	28.5±9	22±7
CD4/CD8 ratio (mean±SD)	0.66±0.4	0.37±0.19	0.71±0.40	0.42±0.22
Patients with viral load <50 copies/mL (n/%)	32/65.3	(0/0)	74/82.2	(0/0)
Patients with viral load >50 copies/mL (n/%)	17/34.7	(14/100)	16/17.7	(35/100)
Viral load (copies/mL; mean±SD)^a^	–	15,082±14699	–	29,501±22,597
Viral load range (copies/mL)^b^	723–755,348	874–49,500	806–235,000	379–73,032

a In treatment-naïve patients

b in treatment-experienced patients with viral-load >50 copies/mL and treatment-naïve patients.

## References

[1] Cohen C, Molina J-M, Cahn P, Clotet B, Fourie J, Grinsztejn B Pooled week 48 efficacy and safety results from ECHO and THRIVE, two double-blind, randomized, phase III trials comparing TMC278 versus efavirenz in treatment-naïve, HIV-1-infected patients.

[2] Pozniak AL, Morales-Ramirez J, Katabira E, Steyn D, Lupo SH, Santoscoy M (2010). Efficacy and safety of TMC278 in antiretroviral-naïve HIV-1 patients: week 96 results of a phase IIb randomized trial. AIDS.

[3] Wilkin A, Pozniak AL, Morales-Ramirez J, Steyn D, Santoscoy M, Grinsztejn B TMC278 shows favorable tolerability and non-inferior efficacy compared to efavirenz over 192 weeks in HIV-1 infected treatment-naïve patients.

# Sub article: P038 Treatment outcomes and comorbidities in German female HIV-infected patients in 2015: a comparison to 2008 and to a male population

**Published:** 2016-10-23

**Authors:** Katja Roemer, Susanne Usadel, Katrin Graefe, Michaela Steib-Bauert, Miriam Speer, Sarah Fischer, Nikola Hanhoff, Gaby Knecht

**Affiliations:** 1 Private Practice, Gemeinschaftspraxis Gotenring, Cologne, Germany; 2 Infektiologikum Freiburg, General Medicine – Infectious Diseases, Freiburg, Germany; 3 Private Practice, Infektionsmedizinisches Centrum Hamburg, Hamburg, Germany; 4 Infectious Diseases, Universitätsklinikum Freiburg, Freiburg, Germany; 5 Private Practice, Praxis Kreuzberg, Berlin, Germany; 6 Private Practice, Infektiologikum Frankfurt, Frankfurt, Germany; 7 Research Co-ordination, Deutsche Arbeitsgemeinschaft niedergelassener Ärzte in der Versorgung HIV-Infizierter, Berlin, Germany; 8 Infektiologikum Frankfurt, Internal Medicine – Infectious Diseases, Frankfurt, Germany

## Abstract

**Introduction**: We characterized HIV-infected women in Germany, evaluated ART use and comorbidities, and compared results to 2007/2008 as well as to a male population in order to further identify special requirements of women living with HIV.

**Materials and methods**: Cross-sectional multicentre evaluation of HIV-positive women and men receiving medical care was performed in Germany between October 2014 and June 2016. All HIV-specialty practices and ambulatory care centres in Germany were invited to participate. Data acquisition was performed using an online questionnaire. Results were compared to a similar analysis performed in 2007/2008 (n=1557).

**Results**: Seven hundred and eighty-one HIV-positive women (f) (n=447 from 10 centres, n=334 from anonymous centres) and 200 HIV-positive men (m) (five centres) were included. Mean age was 45 (f)/44 (m) years, 30.5% (f)/47.7% (m) smoked (p<0.001), 66.7% (f)/60.8% (m) had a partner (of which 64.6% (f)/53.3% (m) were HIV-discordant, p=0.006) and 60.7% (f)/14.7% (m) had child(ren) (p<0.001). Half the women had a migration background, the majority (34%) from Africa, compared to 39% of men (12% from Africa) (p<0.001). 91.7% (f)/95.0% (m) were currently on ART (77% in 2008). Half the men and 28.5% of women received INSTIs (p<0.001); 20.5% (m) versus 38.7% (f) PIs (p<0.001) and 32.6% (m) versus 41.3% (f) received NNRTIs (p=0.03). Sixteen percent of women started ART due to pregnancy. Toxicity was the primary reason for ART discontinuation in both women (37% of discontinuations) and men (36%). 30.7% of women and 16.8% of men reported at least one side effect on ART (p<0.001), with lipodystrophy being more prevalent in females (in 16.9% vs. 8.9% of persons on ART, p=0.006; 24.5% in 2007/8). HIV-1 RNA was <50 copies/mL in 88.1% (f)/90.5% (m) on ART (48% in 2008 with 82% <400 copies/mL). Median detectable viral load in treated individuals was 68 (f)/74 (m) copies/mL (IQR 38–350 (f)/32–1821 (m)). Median CD4 cell count was 621 (f)/628 (m) cells/µL (IQR 437–828 (f)/419–868 (m)).

**Conclusions**: Though certain disease parameters were comparable between HIV-infected women and men, we found significant differences not only socio-demographically, but also in ART use and adverse events. We further noted an improvement in the treatment of HIV-infected women since 2008 as reflected by an increase in both the number of ART-treated women and, more importantly, in the number of successfully treated women. We attribute this to an increasing awareness of women issues, to specific measures taken, to the updates in treatment guidelines and to novel treatment options.

# Sub article: P039 Discontinuation of first-line ART is associated with female sex and migration background – data from a German outpatient clinic

**Published:** 2016-10-23

**Authors:** Sylwia Bilinski, Philipp De Leuw, Siri Göpel, Pavel Khaykin, Gundolf Schüttfort, Hans-Reinhard Brodt, Timo Wolf, Christoph Stephan, Eva Herrmann, Annette Haberl

**Affiliations:** 1 Department of Infectious Diseases, HIV centre, University Clinic Frankfurt, Frankfurt, Germany; 2 Medical Practice, MainFachArzt, Frankfurt, Germany; 3 Institute for Biostats and Mathematical Modelling, Goethe University Frankfurt, Frankfurt, Germany

## Abstract

**Introduction**: Despite individually tailored ART in high-income countries, some patients still discontinue first-line regimens within the first year. This study aimed to characterize treatment discontinuations as well as factors that might be associated with changes in first-line ART.

**Methods**: Patients who initiated first-line ART between January 2009 and December 2013 at the HIVcenter Frankfurt were enrolled in this study and analyzed for treatment discontinuations and changes in ART during the first 60 weeks of therapy. Statistical comparisons were done with non-parametric tests using a significance level of alpha=5%.

**Results**: Overall 557 patients, 420 (75.4%) men and 137 (24.6%) women, were included in this retrospective analysis. Table 1 shows the baseline characteristics of the study population. One hundred and thirty-eight (24.8%) patients discontinued ART within the first 60 weeks, 43 (31.4%) out of 137 women and 95 (22.6%) out of 420 men. ART was interrupted after a mean of 142 days (+/−124). 81.4% of women who experienced a discontinuation were migrants, mostly from African countries. African origin was significantly associated with discontinuation of first-line ART in men and women (p= 0.007). Overall, patients with therapy interruptions had lower CD4 cell counts at baseline and were more likely to be CDC stage C compared to patients with continuing ART. Most common reasons for ART discontinuation were adverse events (56.4%), comorbidities (21%), virological failure (13.5%), adherence issues (12%) and pregnancies (12%).

**Conclusions**: Even the potential of individualized ART cannot prevent treatment changes. In 557 patients starting their first-line ART between 2009 and 2013, we identified 24.8% treatment discontinuations. Patients with female sex and African origin showed the highest rate of discontinuations, and despite guideline recommendations, pregnancy still seems to be an issue for ART modification.

**Abstract P039–Table 1:** Baseline characteristics

	All patients (n = 557)	Male patients (n = 420)	Female patients (n = 137)
Age (years)	40.8 (+/ − 11.4)	41.5 (+/ − 11.2)	38.3 (+/ − 11.5)
Migration background	30.2% (including 15% African origin)	19.3% (including 7% African origin)	63.5% (including 39% African origin)
CDC stage			
A	61.8%	58.9%	69.6%
B	19.5%	20.7%	16.3%
C	18.6%	20.3%	14.1%
CD4 count	292 (+/ − 207)	290 (+/ − 213)	298 (+/ − 184)
HIV RNA	67.600	73.700	41.450
ART components apart from NRTI backbone:			
Protease inhibitor	55.7%	53.3%	62.8%
NNRTI	27.3%	28.6%	23.4%
Integrase inhibitor	20.5%	22.1%	15.3%

# Sub article: P040 Risk factors for late presentation over the last 6 years in Athens, Greece (2009–2015)

**Published:** 2016-10-23

**Authors:** Angeliki Spanos, Dimitra Kavatha, Konstantinos Protopapas, Georgios Siakalis, Maria Argyropoulou, Charalampos Moschopoulos, Emmanouil Antalis, Paraskevi Fragkou, Nikolaos Melachrinopoulos, Sotirios Tsiodras, Anastasia Antoniadou, Antonios Papadopoulos

**Affiliations:** 1 4th Internal Medicine Unit, Attikon University Hospital, Athens, Greece; 2 Medical School, Kapodistrian University of Athens, Athens, Greece

## Abstract

**Introduction**: Late presentation (LP) is a major public health concern for HIV disease. Higher rate of disease progression and disease burden are the main reasons for aiming to reduce late presentation. Although there is an ongoing high interest, the exact parameters for LP have yet to be fully described. This study aims to identify risk factors for HIV-infected patients presenting late at an academic tertiary care centre in Athens, Greece, over the last 6 years in order to define methods for better controlling the epidemic.

**Materials and methods**: We conducted a retrospective case-control study using socio-demographic, behavioural and medical data from patients’ files that were cross-examined by two medical observers. We recorded all new HIV diagnoses presenting between the years 2009 and 2015 at our clinic. Patients enrolled were grouped based on their CD4 cell count that was defined as <350 cells/mm^3^ or an HIV-defining disease on presentation for LPs and >350 cells/mm^3^ for non-LPs. Acute and recent infections with CD4 <350 cells/mm^3^ were reclassified as non-LPs to avoid overestimation of LP due to transient CD4 cell count [1].

**Results**: Five hundred and seven patients were enrolled of which 90% were males and 52.3% were LPs. Heterosexuals were more likely to present late at care versus MSM and IVDUs (p<0.001). Seven out of ten immigrants presented late at care (p<0.001). A linear relation is observed between lower education level and late presentation (p<0.001). There is no significant difference between LPs and non-LPs for employment, and patients living with their parents were more likely to present early at care. LPs requiring hospitalization on diagnosis were 34% versus 12% of non-LPs (p<0.001). Fifty-seven patients presented an HIV-defining disease on diagnosis representing 23.7% of LPs. Total mortality rate was 4.6% with LPs presenting a two times higher mortality rate versus non-LPs (0.06% LPs, 0.03% non-LPs) (p<0.001). Over time, a negative correlation was found for CD4 cell count from 2009 towards 2015 (Kendall's τβ=−0.083, p=0.22).

**Conclusions**: Our data suggest that heterosexuals access medical care later than MSM and IVDUs, implying a gap in current preventive approach and that HIV testing should be offered more frequently for this group. People with lower education and immigrants should be accessed and orientated towards better prevention control. Our results demonstrate that the rate of late presentation tends to increase, a conclusion that demands immediate attention and action in order to succeed an actual control of the HIV epidemic.

## References

[1] Sasse A, Florence E, Pharris A, De Wit S, Lacor P, Van Beckhoven D (2016). Belgian Research AIDS & HIV Consortium (BREACH). Late presentation to HIV testing is overestimated when based on the consensus definition. HIV Med.

# Sub article: P041 Late presenters: how do they look after ART nowadays?

**Published:** 2016-10-23

**Authors:** Catarina Oliveira Paulo, Frederico Duarte, Eduarda Ruiz Pena, Mário Guimarães, Ricardo Correia Abreu, Sofia Jordao, Isabel Neves

**Affiliations:** 1 Infectious Diseases, Hospital Pedro Hispano – Unidade Local de Saúde de Matosinhos, Matosinhos, Portugal

## Abstract

**Introduction**: Currently across Europe, Portugal included, one-third of patients newly diagnosed with HIV infection are late presenters. In spite of multiple ART options, there are still no clear differences in immunogenicity between them, more so in severe immunodeficiency.

**Materials and methods**: Retrospective study of HIV-infected patients of the Infectious Diseases Unit of Hospital Pedro Hispano, starting treatment between January 2010 and January 2016, with European guidelines advised ART, naïve or more than 6 months since ART interruption. Exclusion criteria: <18 years old, virologic failure, ART switch in the first year. Primary outcome: immunologic recovery in absolute and relative count up to 48 weeks in late presenters (lymphocytes T CD4+ cells (TCD4+) <200/µL), relating to backbone and third agent. Statistical evaluation was performed with IBM SPSS version 22 using ANOVA test, excluding drugs with n<3; a significance of 0.05 was considered as statistically relevant.

**Results**: Of a total of 75 patients included, 77% male, average age 45±13.4 years, 56% (n=42) with AIDS, sexual risk in 75% (mostly heterosexual) and 97% (n=73) had type 1 HIV; 23% (n=17) HCV co-infected. At start of treatment TCD4+ count average was 84/µL (1–201). At week 4, there was an average increment of 108±98 TCD4+/µL, week 12 126±89/µL, week 24 156±100/µL and 231±156/µL (n=60) at week 48. Final absolute average was 308/µL. All patients were virologically suppressed since week 24. Concerning backbone agents used, ABC/3TC showed a higher increment in absolute TCD4+ compared to TDF/FTC at week 12 – 204 (95% CI 72–336) versus 108 (95% CI 55–162), p=0.034 – and week 24 – 216 (95% CI 72–361) versus 188 (95% CI 102–274), p=0.031. Regarding the third agent, an increment in absolute TCD4+ count, DRV/R showed statistical superiority to RAL at week 4 – 234 (95% CI 56–411) versus 58 (95% CI −49–164), p=0.014. At week 12, DRV/R was superior to EFV – 223 (95% CI 91–355) versus 117 (95% CI 25–209), p=0.036 – while still being superior to RAL – versus 118 (95% CI 33–203), p=0.047. No drug showed superiority at week 48 or by percentage increase in TCD4+ at any timing. There was no association between evolution of TCD4+ count and initial CDC stage.

**Conclusions**: In this study, we have concluded that ABC/3TC and DRV/R may be the best options for a faster increase in TCD4+ count, although the benefit was not sustained and the small sample may limit the study. Late presenters are at higher risk of considerable comorbidity and mortality, demanding the most immunogenic option and these might be favoured. Even with optimized ART, the TCD4+ recovery observed was not optimal, stressing the importance of early diagnosis.

# Sub article: P042 The utility of enfuvirtide revisited

**Published:** 2016-10-23

**Authors:** Raquel Pinho, Elsa Campoa, Domitilia Faria, Carlos Santos, Luisa Arez

**Affiliations:** 1 Unidade de Portimão Serviço de Medicina, Centro Hospitalar do Algarve, Portimão, Portugal

## Abstract

**Introduction**: Cryptosporidiosis’ main symptom is watery diarrhoea. It is caused by a parasite called cryptosporidium. In persons with AIDS and in other immunocompromised patients, cryptosporidiosis can be serious, long lasting and sometimes fatal. If CD4+ cell count is below 200/mm^3^, cryptosporidiosis is more likely to cause severe symptoms and complications, including prolonged diarrhoea, dehydration and possibly death. The incidence of cryptosporidiosis in patients with HIV has decreased since the introduction of highly active antiretroviral therapy (HAART) [1]. Enfurvitide (T20) is a fusion inhibitor indicated for use in combination with other antiretroviral agents for the treatment of HIV-1 infection in treatment-experienced patients with HIV-1 as part of a salvage regimen. Because of its subcutaneous administration, in severe cases with cryptosporidiosis where the absorption of oral therapeutic is doubtful, enfurvitide may be a therapeutic option.

**Materials and methods**: We extracted details of three individuals with a laboratory confirmed IC and HIV diagnosis between January 2013 and December 2014. The diagnosis of cryptosporidiosis was made by stool sample examination.

**Results**: A.M., 36-year-old female, L.G., 30-year-old male and A.L., 59-year-old male were all admitted to the emergency room because of abdominal pain, nausea, vomiting, weight loss and watery diarrhoea. The symptoms had been present for more than 2 months in all cases. On laboratory examination, all patients were low immunity HIV positive: A.M. with 86 cells/µL CD4 (17%) and viral load (VL) 2,010,000 copies/mL; L.G. with 3 cells/µL CD4 (0.3%) and VL of 318,000 copies/mL; and A.L. with 4 cells/uL CD4 and VL 217,000 copies/mL. They started antiretroviral therapy with emtricitabine and tenofovir/raltegravir but remained symptomatic. This was attributed to the impaired enteric absorption of these drugs. We then decided to add subcutaneous enfuvirtide in order to allow for better bioavailability. After this, all three patients improved their symptoms and immunologic status (CD4: A.M. 741 cells/µL; L.G. 327 cells/µL; A.L. 231 cells/µL) (Figure 1). Treatment with subcutaneous enfuvirtide was stopped after 8 to 12 weeks, after clinical improvement and undetectable viremia (Figure 2).

**Conclusions**: The importance of an immunologic recovery is well established for the cryptosporidium infection in HIV-positive patients. We document with this case series the usefulness of alternative therapies with different routes of administration in order to improve outcomes, when gastrointestinal absorption is impaired.

**Figure 1:** CD4 count (cells/uL) at baseline, month 2, 4 and 6 of treatment with HAART and at June 2016.

**Figure 2:** Viral load (copies/mL) at baseline, month 2, 4 and 6 of treatment with HAART and at June 2016.

## References

[1] O'Connor RM, Shaffie R, Kang G, Ward HD (2011). Cryptosporidiosis in patients with HIV/AIDS. AIDS.

# Sub article: P043 Pre-existing HIV-1 integrase polymorphisms do not impact treatment response to elvitegravir-containing fixed-dose combination regimens in treatment-naïve patients

**Published:** 2016-10-23

**Authors:** Michael Abram, Renee Ram, Kirsten White, Michael Miller, Christian Callebaut

**Affiliations:** 1 Biology, Gilead Sciences, Inc., Foster City, CA, USA

## Abstract

**Introduction**: Across 14 phase 2 or 3 clinical studies, analyses of virologic suppression (HIV-1 RNA <50 copies/mL) of elvitegravir(EVG)/cobicistat(C)/emtricitabine(F)/tenofovir alafenamide (E/C/F/TAF; six studies) or E/C/F/tenofovir disoproxil fumarate (E/C/F/TDF; eight studies) has been evaluated in antiretroviral treatment-naïve, HIV-infected patients at weeks 48, 96 and/or 144. Here, the prevalence of pre-existing integrase (IN) mutations was evaluated relative to subtype, integrase strand transfer inhibitor (INSTI) susceptibility and virologic suppression on EVG-containing fixed-dose combinations.

**Methods**: HIV-1 IN genotypes were obtained from plasma samples before initiation of therapy. IN variability (by position and amino acid change) was compared between patients harbouring B and non-B subtypes, and to the proportion of patients achieving virologic suppression on E/C/F/TAF or E/C/F/TDF. INSTI susceptibility of site-directed mutant viruses was characterized.

**Results**: Two thousand one hundred and seventy-seven of 3033 treated patients across 14 studies were analyzed by population sequencing for pre-existing or transmitted drug resistance in integrase (E/C/F/TAF, n=915; E/C/F/TDF, n=1262). Most patients harboured subtype B (85.1%) versus non-B (14.9%). No primary EVG resistance-associated mutations (RAMs), known to confer reduced EVG susceptibility (T66I/A/K, E92Q/G, S147G, Q148R/H/K, N155H), were detected in any treatment-naïve patient. Some secondary INSTI RAMs were detected as naturally occurring IN polymorphisms, mostly at very low prevalence (0.1–1%: H51Y, L68I/V, V72A/T, L74M, Q95K, T97A, A128T, E138K, S153A, E157K, G163K/R) with some exceptions (≥1%: M50I, L74I, S119G/P/R/T, E157Q); of these, only V72A conferred low-level reduced EVG susceptibility (5-fold). Some secondary INSTI RAMs were more prevalent in subtype B (M50I, S119G/P/R/T, E157Q) or non-B (L74I/M, T97A) subtypes (p<0.05). Overall, 112 of 288 IN amino acid positions had ≥1 variants with a prevalence of ≥1%. Most IN variants (585 of 978) had a prevalence of ≥0.1% and were detected more often in non-B subtypes. Distribution of subtypes and pre-treatment IN variants were comparable between E/C/F/TAF and E/C/F/TDF treated patients. Treated patients (with or without pretreatment IN genotype data) achieved and maintained high rates of virologic suppression. HIV-1 subtype and pre-existing IN polymorphisms at any IN position did not influence treatment response to E/C/F/TAF or E/C/F/TDF (p>0.01).

**Conclusions**: Pre-existing genotypic INSTI resistance is extremely rare in treatment-naïve patients, confined only to a select few minor secondary INSTI RAMs that generally do not confer reduced EVG susceptibility. Natural IN variability, observed more often in non-B subtypes, does not influence virologic suppression rates to EVG-containing fixed-dose combinations. IN genotyping before consideration of EVG-based therapy is currently not warranted unless transmitted drug resistance with primary EVG RAMs is suspected.

# Sub article: P044 Does ethnic origin influence timing, choice and response to first-line antiretroviral therapy in France?

**Published:** 2016-10-23

**Authors:** Caroline Gatey, Alexandre Brun, Isabelle Turpault, Pierre Sellier, Olivier Bouchaud, Olivier Patey, Valerie Garrait, Vincent Jeantils, Amelie Chabrol, Sylvain Diamantis, Valerie Gregoire-Faucher, Eric Froguel, Olivia Son, Sylvia Lamy, Corinne Routier, Hocine Ait-Mohand, Luc Turner, Gwenn Hamet, Constance Delaugerre, Willy Rozenbaum, Jean-Michel Molina, COREVIH Ile de France

**Affiliations:** 1 Infectious Diseases Department, Hopital St Louis, Paris, France; 2 Hopital St Louis, COREVIH Ile de France Est, Paris, France; 3 Internal Medicine, Hopital Lariboisiere, Paris, France; 4 Infectious Diseases Department, Hopital Avicennes, Bobigny, France; 5 Infectious Diseases Department, Hopital Intercommunal de Villeneuve St Georges, Villeneuve St Georges, France; 6 Internal Medicine, Hopital Intercommunal de Créteil, Creteil, France; 7 Infectious Diseases Unit, Hopital Jean Verdier, Bondy, France; 8 Internal Medicine, Hopital Sud Francilien, Corbeil Essonne, France; 9 Infectious Diseases Department, Centre Hospitalier Marc Jacquet, Melun, France; 10 Infectious Diseases Unit, Centre Hospitalier de Meaux, Meaux, France; 1 Infectious Diseases Department, Centre Hospitalier de Marne la Vallée, Jossigny, France; 2 Internal Medicine, Hopital de Longjumeau, Longjumeau, France; 3 Internal Medicine, Centre Hospitalier d'Arpajon, Arpajon, France; 4 Internal Medicine, Centre Hospitalier de Fontainebleau, Fontainebleau, France; 5 Medicine Unit, Centre Medico Chirurgical de Bligny, Bligny, France; 6 Internal Medicine, Centre Hospitalier de Dourdan, Dourdan, France; 7 Microbiology Unit, Hopital St Louis, Paris, France

## Abstract

**Introduction**: African migrants, originating mostly from West Africa, represented up to 39% of new HIV diagnosis in France in 2014. We wished to assess whether timing of ART initiation in naïve patients, the choice of ART and the response to a first-line ART differed in African migrants versus European natives.

**Materials and methods**: We performed a retrospective analysis of prospectively collected data in the COREVIH Ile de France Est, a large network of 26 hospitals using the same electronic database (Nadis^®^). All naïve patients seen from 1 January to 31 December, 2014, and who did not participate in clinical trials were enrolled in this study. We compared baseline characteristics (age, sex, mode of HIV transmission, ethnic origin, centre, CDC stage, CD4 cell counts, plasma HIV RNA level, HBV/HCV co-infection) among patients starting or deferring ART in 2014, among those receiving PI- or non-PI-based ART, and among those experiencing virologic failure over 48 weeks of follow-up. Univariate and multivariate analyses were performed.

**Results**: Nine hundred and twelve naïve patients were enrolled in this study, 446 of whom (49%) were African migrants. Five hundred and eighty-four patients (64%) initiated ART during the study period, and ART initiation was significantly and independently associated with centre, higher baseline viral load, lower baseline CD4+ cell count and ethnic origin. African migrants had an odds ratio (OR) of 1.85 (95% CI 1.22–2.85, p=0.004) to remain ART naïve as compared to European natives. Among the 584 patients who started ART during the study period, the majority (45%) received a PI-based ART and the choice of a PI-based ART was associated significantly and independently with centre, higher baseline viral load, lower baseline CD4+ cell count and ethnic origin. African migrants had an OR of 0.44 (95% CI 0.29–0.66, p<10^−4^) to receive a non-PI-based ART as compared to European natives. Finally, when assessing virologic failure during follow-up, it was significantly and independently associated with centre, higher baseline viral load and ethnic origin. African migrants had an OR of 1.90 (95% CI 1.09–3.32, p=0.02) to develop virologic failure as compared to European natives.

**Conclusion**: African migrants who represent a large proportion of ART-naïve HIV-infected patients in France do not seem to receive the same ART management as other ethnic groups, and also seem to fail ART more frequently. Determinants of these differences should be analyzed in order to provide homogenous care management among HIV-infected patients regardless of their ethnic origin.

# Sub article: P045 Efficacy and safety of tenofovir alafenamide versus tenofovir disoproxil fumarate in treatment-naïve Asian adults

**Published:** 2016-10-23

**Authors:** Shinichi Oka, Ploenchan Chetchotisakd, Amanda Clarke, Khuanchai Supparatpinyo, Sasisopin Kiertiburanakul, Julie Ryu, David Piontkowsky, Susan Guo, Thai Nguyen-Cleary, Moupali Das, Scott McCallister

**Affiliations:** 1 Infectious Diseases, National Center for Global Health and Medicine Hospital, Tokyo, Japan; 2 Infectious Diseases, Khon Kaen University, Khon Kaen, Thailand; 3 Infectious Diseases, Brighton and Sussex University Hospitals NHS Trust, Brighton, UK; 4 Infectious Diseases, Chiang Mai University, Chiang Mai, Thailand; 5 Infectious Diseases, Ramathibodi Hospital Mahidol University, Bangkok, Thailand; 6 Medical Affairs, Gilead Sciences, Foster City, CA, USA; 7 Biometrics, Gilead Sciences, Foster City, CA, USA; 8 Clinical Research, Gilead Sciences, Foster City, CA, USA

## Abstract

**Introduction**: Tenofovir alafenamide (TAF) is non-inferior in efficacy to tenofovir disoproxil fumarate (TDF) and has an improved renal and bone safety profile. In this sub-analysis, we describe the efficacy and safety of TAF compared to TDF in treatment-naïve Asian adults.

**Materials and methods**: This analysis consisted of pooled data from two phase 3, randomized, double blind studies (GS-US-292-0104 and GS-US-292-0111) of HIV-infected, treatment-naïve adults who initiated a single-tablet regimen (STR) of elvitegravir, cobicistat and emtricitabine coformulated with tenofovir alafenamide (E/C/F/TAF) or tenofovir disoproxil fumarate (E/C/F/TDF). Efficacy and safety endpoints through week 96 by self-identified Asian versus non-Asian race within and between treatment groups were examined.

**Results**: One thousand seven hundred and thirty-three adults were randomized and treated: 10% Asians (91 TAF vs. 89 TDF): ex-US (89% vs. 83%), median age (30 vs. 31 years), female (45% vs. 36%), median BMI (22 vs. 22), HIV-1 RNA ≥100,000 copies/mL (26% vs. 33%), CD4 count <200 cells/µL (15% vs. 17%), median eGFR_CG_ (109 vs. 105 mL/min), and proteinuria (8% vs. 8%). At week 96, 97% of Asians on TAF versus 93% on TDF achieved virologic suppression by FDA snapshot algorithm, compared to 85% of non-Asians on TAF and 84% on TDF. Increases in CD4 cell count were numerically higher for Asians on TAF compared to TDF. Both STRs were well tolerated with 1% discontinuations due to adverse events (AEs) for TAF and 2% for TDF. There were no discontinuations due to renal AEs in any Asian participants. There were similar declines in eGFR between TAF and TDF among Asians, consistent with cobicistat's reversible inhibition of creatinine secretion. Changes in other markers of renal safety including urine RBPCR and B2MCR favoured the TAF group, suggesting less impact on renal tubular function. There were minimal decreases in spine and hip bone mineral density (BMD) for the TAF-treated group versus larger decreases in BMD for Asians treated with TDF. Efficacy and safety results for non-Asian participants are shown below (Table 1).

**Conclusions**: TAF and TDF STRs have high and durable efficacy in treatment-naïve Asian adults, with changes in markers of renal and bone safety that consistently favoured TAF over TDF. These data support the use of a TAF-based regimen for the initial treatment of HIV in Asian adults.

**Abstract P045–Table 1:** Week 96 Results

	E/C/F/TAF	E/C/F/TAF	E/C/F/TDF	E/C/F/TDF
	
Week 96 results	Asian (N = 91)	Non-Asian (N = 775)	Asian (N = 89)	Non-Asian (N = 778)
Proportion with HIV-1 RNA <50 copies/mL^a^,^b^	97%	85%	93%	84%
Mean change from baseline in CD4 cell count, cells/µL	+287	+279	+250	+268
Discontinuations	1%	1%	2%	2%
Discontinuations due to renal adverse events, n	0	0	0	6
Median change from baseline in eGFR, mL/min	−7	−1	−9	−7
Median % change from baseline in proteinuria				
Urine protein: creatinine ratio (UPCR)^b^,^d^	0%	−10%	34%	15%
Urine albumin: creatinine ratio (UACR)^d^	<1%	−6%	18%	3%
Urine retinol binding protein: creatinine ratio (RBPCR)^c^,^d^	12%	14%	72%	74%
Urine beta-2-microglobulin: creatinine ratio (B2MCR)^c^,^d^	−38%	−32%	22%	35%
Mean % change from baseline in spine BMD^c^,^d^	−0.3%	−1.1%	−3.2%	−2.7%
Mean % change from baseline in hip BMD^a^,^b^,^c^,^d^	−1.5%	−0.6%	−4.6%	−3.1%

p<0.05 for differences between Asian versus non-Asian in the

a TAF group or

b TDF group; p < 0.05 for differences between TAF versus TDF in the

c Asian group or

d non-Asian group.

# Sub article: P046 Combining lopinavir/r with new ARV agents in ART-naïve HIV-infected patients: data from the German multicenter PROTEKT cohort

**Published:** 2016-10-23

**Authors:** Eva Wolf, Heribert Hillenbrand, Axel Baumgarten, Christoph Stephan, Thomas Lutz, Siegfried Koeppe, Johannes Huelsenbeck

**Affiliations:** 1 MVZ Karlsplatz, HIV Research and Clinical Care Centre, Munich, Germany; 2 MVZ, PraxisCityOst, Berlin, Germany; 3 Zentrum für Infektiologie Berlin, Prenzlauer Berg, Berlin, Germany; 4 Medical Dept. No. 2, University Hospital Frankfurt, Goethe University, Frankfurt, Germany; 5 Infektiologikum, Frankfurt-City, Frankfurt, Germany; 6 Gemeinschaftspraxis, Drs S. Koeppe/P. Kreckel, Berlin, Germany; 7 AbbVie Germany, Medical HIV/HCV, Wiesbaden, Germany

## Abstract

**Introduction**: With the approval of new ARV agents, innovative ations are possible in combination with protease inhibitors. PROTEKT, a non-interventional cohort study initiated in 2008, recruited HIV-1-infected patients receiving cART consisting of lopinavir (LPV)/r plus a “novel” agent other than NRTI, namely raltegravir (RAL), maraviroc (MVC) or etravirine (ETR), without restrictions concerning the use of additional antiretrovirals. Individual choice of cART was at the treating physician's discretion. Ethics approval had been obtained. Observation time was 144 weeks or until discontinuation of cART.

**Methods**: Evaluation of 3-year outcomes in the subgroup of ART-naïve patients of the PROTEKT cohort initiated on cART including LPV/r plus INI, ETR or MVC. Outcomes of interest were time on cART (persistence) using Kaplan-Meier (KM) analysis, time to discontinuation due to virologic failure (VF; censoring discontinuations not related to VF), HIV RNA <50 (400) copies/mL at week 48 using ITT (snapshot; discontinuation=failure; missing data excluded) and as-treated (AT) analyses (missings excluded), as well as CD4 cell changes.

**Results**: Between 2008 and 2014, 501 patients were included in PROTEKT, 90 of them initiated on first-line cART. Baseline characteristics of the ART-naïve study population are shown in Table 1: 37% presented with HIV-related diseases, 7% with hepatitis C, 7% with renal disease, 68% with HIV RNA >100,000 copies/mL, 32% with <200 CD4 cells/µL. LPV/r was combined with either RAL (88 patients, 98%), MVC (one patient, 1%) or ETR (one patient, 1%); 63 patients (70%) received dual therapy LPV/r+RAL, 18 (20%) LPV/r+RAL+TDF/FTC. HIV RNA levels <50 (<400) copies/mL at week 48: ITT, 51% (71%) (N=40/79 (56/79)); AT, 67% (93%) (N=40/60 (56/60)). Median time to discontinuation of cART was 113 weeks; persistence at weeks 48, 96 and 144 was 73%, 54% and 42%, respectively; KM estimates regarding discontinuation due to VF were 94%, 87% and 82%. Median CD4 increases at weeks 48, 96 and 144 were 200/µL (IQR 98–422), 309 (206–449) and 286 (196–473), respectively. Median time to CD4 increase of ≥100 cells/µL was 12 weeks. Most common reasons for discontinuation of study drugs until week 144 were treatment simplification (10/90; 11%), virologic failure (10/90 patients; 11%; 3/10 with HIV RNA <200 copies/mL) and adverse events (9/90 patients, 10%).

**Conclusion**: In the German PROTEKT cohort, LPV/r+RAL as dual therapy or part of cART was used in ART-naïve HIV-infected patients with advanced HIV disease. Median persistence of >2 years in the PROTEKT cohort suggests that non-NRTI-based regimens offer an alternative approach for treatment initiation in specific situations.

**Table 1:** Baseline characteristics of the ART-naïve PROTEKT study population initiated on LPV/r + new agent±other antivirals

Baseline characteristics	
Total, n	90
Gender, n (%)	
Male	82 (91.1%)
Female	8 (8.9%)
Median age, years (IQR)	40 (31–47)
Ethnicity^a^, n (%)	
Caucasian	72 (82.8%)
African	10 (11.5%)
Asian	2 (2.3%)
Other	3 (3.4%)
Median HIV RNA level, log copies/mL (IQR)	5.3 (4.7–6.0)
HIV RNA >100,000 copies/mL, n (%)	61 (67.8%)
Median CD4 cell count, cells/µL (IQR)	302 (162–450)
CD4 cell count <200/µL, n (%)	29 (32.2%)
Median rel. CD4 cell count,% (IQR)	15 (10–22)
Patients with comorbidities	
Renal disease/impairment, n (%)	6 (6.7%)
Liver disease, n (%)	1 (1.1%)
Chronic hepatitis B, n (%)	1 (1.1%)
Chronic hepatitis C, n (%)	6 (6.7%)
CDC B/C	33 (36.7%)

# Sub article: P047 Outcome of antiretroviral regimens prescribed by following the regulations on combination antiretroviral therapy by Taiwan Centers for Disease Control

**Published:** 2016-10-23

**Authors:** Mao-Song Tsai, Chia-Jui Yang, Jun-Yu Zhang, Pei-Ying Wu, Shang-Ping Yang, Yu-Zhen Luo, His-Yen Chang, Kuan-Yin Lin, Hsin-Yun Sun, Chien-Ching Hung

**Affiliations:** 1 Internal Medicine, Far Eastern Memorial Hospital, New Taipei City, Taiwan; 2 Center of Infection Control, National Taiwan University Hospital, Taipei, Taiwan; 3 Department of Internal Medicine, National Taiwan University Hospital, Taipei, Taiwan

## Abstract

**Introduction**: To curb the increasing medical cost for HIV care, Taiwan Centers for Disease Control (CDC) implemented regulations on cART according to the monthly cost for each regimen on 1 June 2012. By following the regulations, many individuals commence thymidine analogue (TA)-based regimens. Prior authorization is needed for the regimens containing protease inhibitors (PIs), integrase strand transfer inhibitors (INSTIs) or rilpivirine (RPV) plus non-TA backbones (abacavir/lamivudine; tenofovir disoproxil fumarate (TDF)/emtricitabine; or TDF plus lamivudine). We aimed to describe the outcome of the regimens prescribed by following the regulations in Taiwan.

**Materials and methods**: Between June 2012 and June 2016, antiretroviral-naïve HIV-positive patients who initiated cART were included. We retrospectively collected information on clinical characteristics, regimens, treatment responses, causes for switch and genotypic resistance at baseline and follow-up. We investigated the predictors of treatment modification with Cox proportional hazards model.

**Results**: During the 48-month study period, 1390 patients with baseline median CD4 count of 282 cells/µL and plasma HIV RNA load (PVL) 4.85 log10 copies/mL initiated cART: 31.1% TA backbones plus nevirapine, efavirenz or RPV (first category); 59.1% non-TA backbones plus nevirapine or efavirenz (second category); 6.3% TA plus PIs, INSTI or RPV (third category); and 3.7% non-TA plus boosted PI, II or RPV (fourth category). Overall, 65.6% (n=912) had to change the initial regimens at a median interval of 41 days (range 1–1140) because of regimen simplification (33.6%), rash (20.5%), neuropsychiatric adverse effect (14.0%), genotypic resistance/virologic failure (10.8%), gastrointestinal intolerance (9.3%), anaemia (9.2%) or hepatitis (4.9%). The rates of regimens modification for drug-related adverse reactions were 61.4%, 34.1%, 53.6% and 29.4% in patients in the first, second, third and fourth category of regimens, respectively. Except for regimen simplification, 6-month modification-free survival rates were 67.6% (range 64.2–70.1%) in the patients on regimens containing non-TA backbones and 48.9% (44.4–53.1%) in the patients on regimens containing TA backbone (hazard ratio (HR) 1.82 (95% CI 1.55–2.14)) (Figure 1). In multivariate analysis, use of regimens containing TA backbone (HR 1.84; 95% CI 1.57–2.16) and higher PVL (HR, per 1-log10 copies/mL increase, 1.15; 95% CI 1.03–1.29) were independent predictors of cART switch.

**Conclusions**: A significant proportion of patients on cART regimens, especially the regimens containing TA backbone that were prescribed by following the regulations of Taiwan CDC, had to be changed due to toxicities, transmitted drug resistance or unsatisfactory virologic response.

**Abstract P047–Figure 1:** Time to regimens modification during the first 24 weeks after starting antiretroviral treatment according to the regimen (TA backbone vs. non-TA backbone).

# Sub article: P048 Prescription pattern and determinants of dolutegravir use in an antiretroviral-naïve HIV-infected population in Italy: data from the ICONA Foundation cohort study

**Published:** 2016-10-23

**Authors:** Andrea Antinori, Alessandro Cozzi Lepri, Antonella Castagna, Sergio Lo Caputo, Cristina Mussini, Stefano Rusconi, Antonio Di Biagio, Giulia Marchetti, Silvia Nozza, Antonella Cingolani, Andrea De Luca, Antonella d'Arminio Monforte, Study Group on behalf of Icona Foundation

**Affiliations:** 1 HIV/AIDS Department, National Institute for Infectious Diseases, Roma, Italy; 2 Infection and Population Health, University College London, London, UK; 3 Infectious Diseases, San Raffaele Hospital, Milano, Italy; 4 Infectious Diseases, University of Bari, Bari, Italy; 5 Infectious Diseases, University of Modena, Modena, Italy; 6 Infectious Diseases, Luigi Sacco Hospital, University of Milano, Milano, Italy; 7 Infectious Diseases, University of Genova, Genova, Italy; 8 Health Sciences, San Paolo Hospital, University of Milano, Milano, Italy; 9 Infectious Diseases, Catholic University, Roma, Italy; 10 Infectious Diseases, University of Siena, Siena, Italy

## Abstract

**Introduction**: Dolutegravir (DTG), an integrase strand transfer inhibitor (INSTI), is currently recommended for treatment initiation in antiretroviral-naïve by most of clinical guidelines. Data from randomized trials demonstrated superiority of DTG over NNRTI and PI/r, supporting its clinical use especially in first-line regimens with high viral load, regardless of which NRTI pair (TDF/FTC or ABC/3TC) was initiated. Aim of this analysis was to describe DTG use in a representative unselected naïve population in Italy and identify its determinants and patterns of prescription.

**Methods**: All patients enrolled in ICONA Foundation cohort starting a DTG-based regimen from ART-naïve, and those concomitantly starting other third drugs (control group) after 1 January 2014 all using as NRTI pair TDF/FTC or ABC/3TC were selected. Cross-sectional analysis was performed, and characteristics at the time of starting cART compared using chi-square test and univariable and multivariable logistic regression.

**Results**: A total of 1944 ARV-naïve individuals starting cART were included (DTG=195; NNRTI=715; PI/r=576; other INSTI=458). The crude prevalence of DTG use was 10%. Median HIV-1 RNA was 4.7 log10/mL in DTG and 4.6 log10/mL in controls (p=0.13); median CD4 counts were 386 cells/mm^3^ and 376 cells/mm^3^, respectively (p=0.78). Proportion of patients with HIV-1 RNA values >100,000 was 36.6% among DTG and 29.3% among controls (p=0.06). Fitting a logistic regression, a significantly increased probability of starting DTG was found with more recent calendar years and higher baseline HDL cholesterol values, and a decreased probability in sites located in central Italy, with a trend towards more frequent use with a pVL >100,000 (Table 1). NRTI pair was TDF/FTC in 65% of DTG and 90% of controls, and ABC/3TC in 35% of DTG and 10% of controls, with a significantly higher probability of starting ABC/3TC in the DTG group (Table 1). Proportion of ABC/3TC combination with DTG was 44% in pVL ≤100,000, but only 24% in pVL >100,000 stratum (interaction p=0.37).

**Conclusions**: During the first 2 years after its introduction in Italy, DTG prescriptions in ART-naïves showed an increase over time, although patterns were not different from those observed for control regimens, except for a trend for starting DTG at high VL. ABC/3TC was more frequently prescribed with DTG than in controls, even though TDF/FTC remains the most prescribed NRTI option combined with DTG in people with high viral load. Concerns on ABC/3TC potency, toxicity and lack of rapid HLA results might have affected clinicians’ choices.

**Abstract P048–Table 1:** Odds ratios of starting DTG versus control from fitting a logistic regression

	Odds ratios of starting dolutegravir versus control			
	
Characteristics	Unadjusted OR (95% CI)	p	Adjusted^a^ OR (95% CI)	p
Viral load, log10 copies/mL				
>100,000 vs. ≤100,000	1.39 (0.98–1.97)	0.061	2.17 (0.96–4.93)	0.063
HDL cholesterol, mg/dL				
Per 100 higher	2.22 (0.52–9.49)	0.283	8.02 (1.13–56.88)	0.037
Site geographical location				
North	1.00			
Centre	0.66 (0.44–0.99)	0.043	0.34 (0.12–0.99)	0.048
South	1.05 (0.62–1.76)	0.866	1.18 (0.52–2.71)	0.693
NRTI pair				
ABC/3TC vs. TDF/FTC	5.83 (4.03–8.43)	<0.001	14.30 (6.81–30.04)	<0.001
Period of initiation				
January–December 2014	1.00		1.00	
January–June 2015	13.57 (6.94–26,55)	<0.001	15.49 (5.83–41.20)	<0.001
July 2015–March 2016	24.87 (12.62–49.04)	<0.001	19.92 (6.63–59.84)	<0.001

a Besides factors shown in the table, also adjusted for gender, nationality, age, education, employment, mode of HIV transmission, time from HIV first diagnosis, AIDS diagnosis, HCV status, HBV status, baseline CD4 count, baseline CD8 count, diagnosis of diabetes dyslipidaemia and psychological disorders, and eGFR.

# Sub article: P049 The RESINA data support the individualized therapy based on primary resistance testing

**Published:** 2016-10-23

**Authors:** Elena Knops, Eugen Schuelter, Nadine Luebke, Maria Neumann-Fraune, Eva Heger, Saleta Sierra-Aragon, Claudia Mueller, Mark Oette, Gerd Faetkenheuer, Martin Hower, Heribert Knechten, Niels Schuebel, Stefan Esser, Stefan Scholten, Dieter Haeussinger, Rolf Kaiser, Bjoern Jensen

**Affiliations:** 1 Institute of Virology, University of Cologne, Cologne, Germany; 2 Institute for Virology, University of Duesseldorf, Duesseldorf, Germany; 3 Department of Gastroenterology, Krankenhaus der Augustinerinnen, Cologne, Germany; 4 Department of Internal Medicine, University of Cologne, Cologne, Germany; 5 Department of Internal Medicine, City Hospital Dortmund, Dortmund, Germany; 6 Private Practice, Private Practice Dr. Knechten, Aachen, Germany; 7 Department of Internal Medicine, Klinikum Osnabrueck, Osnabrueck, Germany; 8 Department of Dermatology, University of Essen, Essen, Germany; 9 Private Practice, Private Practice Hohenstauffenring, Cologne, Germany; 10 Department of Gastroenterology, Hepatology and Inf, University of Duesseldorf, Duesseldorf, Germany

## Abstract

**Introduction**: The RESINA study started in 2001 and was originally focused on the evaluation of primary resistance in patients at the time point of first therapy. Additionally, we could follow up these patients (RESINA cohort) since cART start by collecting the clinical, virologic and immunologic data.

**Materials and methods**: The clinical, virologic and immunologic data were collected from 38 centres since 2001. Genotypic analysis of resistance-associated mutations (RAMs) was performed from viral RNA exclusively until 2012, since then additionally from proviral DNA and/or total NA. Resistance-associated mutations were detected by Sanger sequencing and recently by next-generation sequencing by the Illumina MiSeq technology. Additionally, we collect data from any therapy-experienced patient within the AREVIR project.

**Results**: Meanwhile the RESINA cohort consists of more than 3800 patients from almost 40 HIV centres in North-Rhine-Westphalia. Furthermore, we performed a total number of more than 13,000 resistance tests from therapy-naïve and therapy-experienced patients (RESINA and AREVIR data). During this time, we could observe a decline in prevalence of resistance-associated mutations in treatment-experienced patients as documented in the AREVIR database. In contrast to the decline of RAMs in therapy-experienced patients, the frequency of primary resistance-associated mutations at the beginning of cART remains relatively stable. The majority of the primary RAMs were NRTI resistance mutations throughout the whole time of observation. NNRTI resistance-associated mutations did not increase over time although the use of NNRTI increased in our cohort since 2001. We did not observe an increase in primary PI resistance-associated mutations and almost no primary INI resistance mutations.

**Conclusions**: Despite the declining frequency of resistance-associated mutations in therapy-experienced patients the frequency of primary resistance mutations is still high and justifies routine primary resistance testing. We can further conclude from our data that the individualized therapies according to the DAIG therapy guidelines for therapy-naïve patients translate in a low number of NNRTI and PI resistance-associated mutations in therapy-naïve and therapy-experienced patients.

Treatment Strategies – Target Populations: Primary Infection

# Sub article: P050 The acute/recent HIV infection cohort from Hospital Clinic, Barcelona: epidemiological trends and evolution from 1997 to 2015

**Published:** 2016-10-23

**Authors:** David Nicolás Ocejo, Juan Ambrosioni, Christian Manzardo, Fernando Agüero, Mar Mosquera, Marta Parera, Sonsoles Sanchez-Palomino, Carmen Ligero, Emma Fernandez, Elisa de Lazzari, Montserrat Plana, Jose Gatell, Jose Miró

**Affiliations:** 1 Infectious Diseases, Hospital Clinic Barcelona, Barcelona, Spain; 2 Microbiology, Hospital Clinic Barcelona, Barcelona, Spain; 3 Institut d'Investigacions Biomèdiques August Pi i Sunyer, Retrovirology and Viral Immunopathology Laboratory, Barcelona, Spain

## Abstract

**Introduction**: The epidemiology and clinical management of primary/recent HIV infection (PHI) has undergone significant changes over the last 20 years. The epidemiological profile of PHI patients has probably also varied, although published evidence is scarce.

**Methods**: Since 1997, data have been collected from every patient with an acute/recent HIV infection (less than 180 days) visiting our hospital. The inclusion criteria were a negative antibody assay with a detectable viral load or a positive p24 antigen, a positive antibody assay with a negative, indeterminate or incomplete western blot, or a documented negative test in the preceding 6 months. Patients were stratified into four time periods (1997–2001, 2002–2006, 2007–2011, 2012–2015).

**Results**: In the last 19 years, 337 patients were included with a median follow-up of 81 months. The main risk factor was men having sex with men (MSM) with an increasing trend in the most recent periods (p<0.001). Intravenous drug use (IDU) decreased from 23% in the earliest period to 1% in the last. Positive HCV serology at diagnosis was higher in the first period, while syphilis diagnosis remained stable over time. In the first period, 23% of patients were immigrants, increasing to 40% in the last period. Non-B subtypes also increased but not significantly. Nineteen percent of patients presented with a Fiebig I-III stage at diagnosis and the median time from infection to diagnosis was 40 days (IQR 28–79) with little change over time (Table 1). Seventy percent of patients presented with a symptomatic PHI, and 25% of them required hospital admission. Ninety-three percent of patients started ART. Of them, 27% started during the first 3 months, and 45% during the first 6 months as of the infection date. Median CD4 at ART initiation remained steady and median time from diagnosis to treatment underwent a temporary increase during the middle periods (p<0.001) (Figure 1). The chosen ART regimen varied among periods, with a substitution of PIs with INSTI in the last period (Figure 2).

**Conclusions**: Our results show significant changes in the profile of patients with acute/recent HIV infection in Barcelona with an increase of MSM in the most recent periods. The interval between diagnosis and ART start increased during the central calendar period (2002–2011), reflecting conservative guideline recommendations of that time. The INSTI-based ART regimens were the preferred starting option in the last years.

**Figure 1:** Median and IQR CD4 at ART start during the four study periods.

**Abstract P050–Figure 2:** Evolution of the ART chosen as the third drug as starting scheme among periods.

**Abstract P050–Table 1:** Epidemiological characteristics of the PHI cohort

	Global	1997–2001	2002–2006	2007–2011	2012–2015	p
Men, n (%)	314 (93.2%)	23 (76.7%)	49 (87.5%)	145 (97.3%)	97 (95.1%)	<0.001
Age (years)	33.7	33.3	33.2	34.6	34.4	0.164
Transmission, n (%) – MSM	291 (86.9%)	19 (63.3%)	42 (75%)	138 (93.9%)	92 (90.2%)	<0.001
Transmission, n (%) – IDU	17 (5.1%)	7 (23.3%)	5 (8.9%)	4 (2.7%)	1 (1%)	–
Origin – Spain	213 (63.4%)	23 (76.7%)	36 (65.5%)	93 (62.4%)	61 (59.8%)	0.391
Origin – immigrants	123 (36.6%)	7 (23.3%)	19 (34.5%)	56 (37.6%)	41 (40.2%)	–
Drugs use, n (%) – IV	10 (3.1%)	5 (17.2%)	2 (3.8%)	3 (2.1%)	0 (0%)	<0.001
Drugs use, n (%) – inhaled	79 (24.5%)	1 (3.4%)	13 (25%)	38 (27%)	27 (26.7%)	–
Drugs use, n (%) – oral	16 (5%)	0 (0%)	2 (3.8%)	6 (4.3%)	8 (7.9%)	–
Follow-up (months), median (IQR)	81 (54–118)	200 (178–217)	134 (120–157)	78 (65–91)	40 (27–48)	–
Time from infection to diagnosis (days), median (IQR)	40 (28–79)	31 (29–77)	42 (29–64)	40 (26–86)	39 (27–74)	<0.001
Fiebig I-III, n (%)	64 (19%)	9 (30%)	14 (25%)	30 (20.1%)	11 (10.8%)	0.004
HCV+ at diagnosis, n (%)	19 (4.9%)	7 (23.3%)	4 (7.1%)	3 (2.1%)	3 (2.1%)	<0.001
VDRL+ at diagnosis, n (%)	40 (12.8%)	5 (17.2%)	6 (10.9%)	16 (11.2%)	13 (15.3%)	0.684
Time from infection to ART (days), median (IQR)	204 (84–523)	99 (63–156)	109 (51–1140)	336 (155–646)	122 (80–239)	<0.001
Time from diagnosis to ART (days), median (IQR)	133 (41–472)	56 (22–74)	62 (19–1052)	284 (103–622)	79 (36–172)	<0.001

# Sub article: P051 Neurological involvement in patients with acute/recent HIV-1 infection

**Published:** 2016-10-23

**Authors:** Francisca Artigues, Juan Ambrosioni, David Nicolás, Judit Peñafiel, Fernando Agüero, Christian Manzardo, Mar Mosquera, Sonsoles Sánchez-Palomino, Elisa de Lazzari, Maria Angeles Marcos, Montserrat Plana, Jose Miro

**Affiliations:** 1 School of Medicine, University of Barcelona, Barcelona, Spain; 2 Infectious Diseases, Hospital Clinic, Barcelona, Spain

## Abstract

**Introduction**: Neurological involvement during primary HIV-1 infection (PHI) has been poorly studied. Early antiretroviral therapy (ART) has shown to improve symptoms, to promote a better immunological recovery and to reduce the size of viral reservoir [1]. Little is known, however, about ART in the context of neurological involvement during PHI [2,3]. The aim of this study was to describe the clinical characteristics and outcomes of patients presenting neurological symptoms during PHI and to compare them with a control group without neurological involvement.

**Materials and methods**: We described the 14 patients (3.02% of the whole PHI cohort) with neurological symptoms that were enrolled in the acute/recent hospital clinic PHI cohort (documented infection <6 months) between 1997 and 2016. A retrospective case-control study was developed, matching each case with three controls. Matching criteria included age (±10 years), gender, year of the diagnosis (±4 years) and same Fiebig stage. Statistical analyses were performed using R software version 3.2.3. The conditional logit model was used to compare variables between the matched cases and controls.

**Results**: Fever and headache with at least one other neurological symptom were the most frequent manifestations among cases: 28.5% presented as meningitis and 71.5% as meningoencephalitis. Cerebrospinal fluid showed pleocytosis with lymphocyte predominance and increased protein levels. Adenosine deaminase was elevated in 42.8% of cases. No other pathogen was identified in any case. Case-control comparisons can be seen in Table 1. All cases required hospitalization, whereas only 19% of the controls did. CD4/CD8 ratio was significantly lower in the case group (p=0.039) and plasmatic viral load was significantly higher in the case group (p=0.028). There were no differences regarding risk factors, HIV-1 tropism, subtype distribution or prescribed ART regimens. After 6 months on ART, 92% of cases had undetectable HIV-1 viral load, similar to controls. All cases recovered rapidly with ART and were discharged without sequels.

**Conclusions**: Neurological involvement during PHI is an unusual but serious condition, always requiring hospitalization. Early diagnosis might be difficult because of the wide range of neurological symptoms and similarities with other viral aetiologies. Neurological manifestations during PHI are associated with a lower CD4/CD8 ratio and with a higher viral load at diagnosis than controls. Immediate initiation of ART to rapidly decrease the viral load is required in this scenario. Dolutegravir, together with lamivudine and abacavir, seems a reasonable regimen due to its potency in reducing viral load and its high CNS penetration.

**Abstract P051–Table 1:** Comparison between cases and controls

Variable	Cases N = 14	Controls N = 42	p
PHI symptoms	14 (100%)	35 (83.3%)	–
Fever	14 (100%)	29 (69.0%)	–
Headache	12 (85.7%)	11 (26.2%)	<0.01
Need of hospitalization	14 (100%)	8 (19.0%)	–
Days of hospitalization (n = 20), median (IQR)	6.0 (2.75–7.75)	7.0 (5.25–8.0)	0.496
Tropism R5 (n = 22)	6 (85.7%)	8 (53.3%)	0.229
Subtype B (n = 38)	10 (100%)	26 (92.9%)	–
Days diagnosis – ART (n= 53), median (IQR)	11.5 (5.0–40.8)	140.0 (38.2–284.0)	0.041
NNRTI-based ART	4 (28.6%)	10 (23.8%)	0.732
Integrase inhibitor-based ART	2 (14.3%)	9 (21.4%)	0.500
Protease inhibitor-based ART	8 (57.1%)	19 (45.2%)	0.448
Transmitted drug resistance	1 (7.14%)	5 (12.2%)	0.621
Log10 VL diagnosis, median (IQR)	5.99 (5.29–6.59)	5.24 (4.62–5.86)	0.019
Log10 VL ART starting (n = 53), median (IQR)	5.50 (4.80–6.19)	4.96 (4.37–5.50)	0.028
Patients with VL <50 copies/mL at 6 months ART (n = 47)	11 (92%)	23 (66%)	0.133
Patients with VL <50 copies/mL at 12 months ART (n = 46)	11 (92%)	30 (88%)	0.520
CD4 cells/mm3 diagnosis (n = 55), median (IQR)	328 (253–443)	468 (316–568)	0.184
CD4 cells/mm3 ART starting (n = 51), median (IQR)	368 (263–441)	404 (273–580)	0.190
CD4 cells/mm3 6 months ART (n = 48), median (IQR)	581 (493–787)	634 (440–791)	0.569
CD4 cells/mm3 12 months ART (n = 46), median (IQR)	804 (642–889)	630 (555–864)	0.117
CD4/CD8 ratio diagnosis (n = 55), median (IQR)	0.33 (0.22–0.44)	0.53 (0.36–0.80)	0.039
CD4/CD8 ratio ART starting (n = 51), median (IQR)	0.30 (0.24–0.45)	0.49 (0.36–0.67)	0.060
CD4/CD8 ratio 6 months ART (n = 48), median (IQR)	0.82 (0.65–0.89)	0.76 (0.53–0.99)	0.738
CD4/CD8 ratio 12 months ART (n = 46), median (IQR)	1.00 (0.82–1.04)	0.88 (0.76–1.40)	0.780

NNRTI, non-nucleoside reverse-transcriptase inhibitors; VL, viral load. p-value was not calculated in those variables with 100% or 0%.

## References

[1] Cohen M, Shaw G, McMichael A, Haynes B (2011). Acute HIV-1 infection. N Engl J Med.

[2] del Saz S, Sued O, Falcó V, Agüero F, Crespo M, Pumarola T (2008). Acute meningoencephalitis due to human immunodeficiency virus type 1 infection in 13 patients: clinical description and follow-up. J Neurovirol.

[3] Letendre S, Ellis R, Ances B, McCutchan J (2010). Neurologic complications of HIV disease and their treatment. Top HIV Med.

# Sub article: P052 Single-tablet regimen with elvitegravir, cobicistat, emtricitabine and tenofovir for the treatment of early HIV infection

**Published:** 2016-10-23

**Authors:** Ramon Teira, Maria Galindo, Marta Montero, Raquel Portilla, Ana Ferrer, Elisa Martinez

**Affiliations:** 1 Infectious Diseases, Hospital de Sierrallana, Torrelavega, Spain; 2 Infectious Diseases, Hospital Clinico, Valencia, Spain; 3 Infectious Diseases, Hospital La Fe, Valencia, Spain; 4 Internal Medicine, Hospital de Sierrallana, Torrelavega, Spain; 5 Hospital de Albacete, Internal Medicine, Albacete, Spain

## Abstract

**Introduction**: Treatment of early HIV infection (EHI) (within the 6-month period after the transmission event) has been recommended, based on pathophysiologic considerations and on surrogate-marker outcomes. The rationale supporting this recommendation (the accelerated pace of pathophysiologic events in this early phase of the infection) argues also for an immediate initiation of treatment. Stribild^®^, the single-tablet regimen (STR) including elvitegravir, cobicistat, emtricitabine and tenofovir, seems particularly well suited for this indication, due to its not needing additional laboratory data and the convenience of the STR formulation.

**Methods**: We reviewed the medical records of those patients attending the participating clinics with a diagnosis of EHI who started treatment with Stribild^®^. We classified them as 1) definite EHI if a) they had a negative serologic test within the 6 months prior to the diagnosis and any positive test at presentation or b) they had a simultaneous negative serologic test and a positive HIV RNA test or p24 antigen; or 2) probable EHI if they had not a negative serologic test within the previous 6 months, but they had an epidemiologic and clinical syndrome suggestive of acute HIV infection plus an HIV RNA plasma concentration (VL) higher than 100,000 copies/mL.

**Results**: Stribild^®^ has been available in Spain since January 2014. In this period, 21 patients attending the participating clinics were diagnosed with EHI and started treatment with Stribild^®^, of whom 17 were definite and 4 were probable diagnoses. Nineteen (90.5%) were male. The median age (95% interquartile range (IQR)) was 34 (30–42). HIV infection had been transmitted through sexual contact in all cases: in two women (100%), in 17 men who had sex with men (89.5%) and in two men (10.5%) who did not report sex with men. The median CD4 cell count and VL at the time of diagnosis were 450 (IQR 290–567) and 520,000 (153,000–1,900,000), respectively. We have data from 19 patients after 3 months of treatment, and from 12 patients after 1 year. At 3 months, the median VL was 46 copies/mL (IQR <20–254). At 12 months, one patient had withdrawn and had a VL of 6162, while the remaining 11 were still on treatment, all of whom, except one, had VL <50 (the other was 106 copies/mL). We recorded no major or renal adverse effects.

**Conclusions**: Our results support the hypothesis that Stribild^®^ is safe and effective for the treatment of EHI. The possible benefit of immediate initiation warrants further investigation.

# Sub article: P053 Comparison of (TDF+FTC) associated with either darunavir/ritonavir+raltegravir or dolutegravir: virological efficacy of two different treatment strategies for primary HIV infection (PHI)

**Published:** 2016-10-23

**Authors:** Carmela Pinnetti, Isabella Abbate, Patrizia Lorenzini, Nicoletta Orchi, Caterina Gori, Alessandra Amendola, Raffaella Libertone, Gabriella Rozera, Maria Maddalena Plazzi, Gabriele Fabbri, Maria Rosaria Capobianchi, Andrea Antinori, Adriana Ammassari

**Affiliations:** 1 Clinical Department, National Institute for Infectious Diseases, Rome, Italy; 2 Laboratory of Virology, National Institute for Infectious Diseases, Rome, Italy; 3 Epidemiology Department, National Institute for Infectious Diseases, Rome, Italy; 4 Antiretroviral Drug Monitoring Unit, National Institute for Infectious Diseases, Rome, Italy

## Abstract

**Introduction**: Optimal therapy for PHI is unknown. Although INI as fourth drug has often been used, RCT failed to demonstrate virological benefit in respect to conventional cART. Aim was to compare virological efficacy of (TDF+FTC) with darunavir/ritonavir 800/100 mg+raltegravir 400 mg BID (DRV/r+RAL) BID or dolutegravir 50 mg QD (DTG) for PHI therapy.

**Methods**: SIREA is a monocentre, prospective, observational study. PHI patients were treated consecutively with TDF/FTC associated with DRV/r+RAL (July 2013 to May 2015) or DTG (May 2015 to April 2016). Follow-up was until first virological response (VR) defined as HIV RNA 40 copies/mL or death, last observation, whichever came first. Factors associated with HIV RNA 40 copies/mL during the first year were evaluated from fitting Cox proportional hazard regression model retaining significant variables at univariable analysis (p<0.10).

**Results**: Eighty-seven patients: males 94.2%, mean age 34 years. HIV diagnosis was 6 days before (range 4–10) and acquired by MSM 78.2%, heterosexual 19.5%, IVDU 2.3%. Fiebig: II/III 28.7%, IV 32.5%, V 15.0%, VI 23.7%. Median baseline (BL): HIV RNA log 5.5 copies/mL (IQR 4.6–6.6), HIV DNA x106 PBMC log 4.1 (IQR 3.7–4.8), CD4 570/mm3 (IQR 387–739). cART contained DRV/r+RAL in 63 (72.4%) cases and DTG in 24 (27.6%). At GRT, no resistance mutations to the prescribed drugs were found. BL characteristics, particularly HIV RNA (5.8 (95% CI 5.1–6.7) log copies/mL for DRV/r+RAL vs. 5.1 (4.1–6.4) for DTG; p=0.12) and HIV DNA (4.2 (3.7–4.8) log copies/106 PBMC for DRV/r+RAL vs. 4.1 (3.8–4.8) for DTG; p=0.90), were not different between groups. During 296 PYFU 79 patients (90.1%) achieved VR, response rate for DRV/r+RAL 22.5 x100 PYFU (95% CI 17.4–29.1) and for DTG 57.7 (95% CI 37.2–89.4) (p<0.001) (Figure 1). No grade 3–4 adverse events were seen. In multivariable analysis, BL HIV RNA (OR 0.60; 95% CI 0.48–0.75; p<0.001) and BL CD4 350–500 versus >500/mm^3^ (OR 0.41; 95% CI 0.22–0.74; p=0.003) were associated with reduced risk of VR. On the contrary, BL CD4/CD8 >1 versus <1 (OR 2.22; 95% CI 1.28–3.85; p=0.004) and DTG versus DRV/r+RAL (OR 3.42; 95% CI 1.83–6.42; p<0.001) had a higher chance for VR. Age, gender, transmission mode, Fiebig and days from diagnosis were not associated.

**Conclusions**: In PHI, a three-drug cART based on DTG+(TDF+FTC) seems to perform better than a four-drug therapy with DRV/r+RAL+(TDF+FTC) in time to achieve HIV RNA 40 copies/mL in the first year. Besides potency, also lower pill burden and simpler schedule may have contributed. Results have to be taken cautiously because of potential confounder by indication and prescription bias.

**Abstract P053–Figure 1:** Proportion of patients reaching HIV RNA 40 copies/mL at various time points.

# Sub article: P054 Safety and efficacy of dolutegravir plus rilpivirine (DTG/RPV) in treatment-experienced HIV-infected patients: preliminary results at 24 weeks of the DORIVIR study

**Published:** 2016-10-23

**Authors:** Rosario Palacios, Marisa Mayorga, Carmen-María González-Domenech, Carmen Hidalgo-Tenorio, Carmen Gálvez, Leopoldo Muñoz-Medina, Javier de la Torre, Ana Lozano, Manuel Castaño, Mohamed Omar, Jesús Santos

**Affiliations:** 1 UGC Enfermedades Infecciosas y Microbiología, Hospital Virgen de la Victoria, Málaga, Spain; 2 UGC Enfermedades Infecciosas y Microbiología, Hospital Carlos Haya, Málaga, Spain; 3 Servicio de Enfermedades Infecciosas, Complejo Hospitalario Universitario Granada, Granada, Spain; 4 UGC Enfermedades Infecciosas, Hospital Torrecárdenas, Málaga, Spain; 5 UGC Enfermedades Infecciosas, Hospital Costa del Sol, Málaga, Spain; 6 UGC Enfermedades Infecciosas, Hospital de Poniente, Almería, Spain; 7 UGC Enfermedades Infecciosas, Hospital de Jaén, Jaén, Spain

## Abstract

**Introduction**: DTG/RPV is a two-pill nucleoside reverse transcriptase inhibitor (NRTI) and protease inhibitor (PI)-sparing regimen with very good tolerance. It is currently in phase 3 clinical trials being developed as two-drug “maintenance therapy”. The aim of this study is to analyze the efficacy and safety of this regimen in HIV-infected patients who switched from any other ART combination.

**Methods**: Open-label, multicentre, non-controlled study in seven hospitals from Andalusia, southern Spain. Patients who switched from any regimen to DTG/RPV from February 2015 to February 2016 were included. Epidemiological, clinical and antiretroviral data in addition to immediate reasons for switching were collected. Lipids, liver and renal tests were measured at baseline and at 24 weeks. The primary endpoint was the proportion of patients with plasma HIV RNA <50 copies/mL at 24 weeks (missing=failure), and secondary endpoints included adverse events and rate of discontinuation related with adverse events of dual therapy after switching and metabolic changes at 24 weeks.

**Results**: Hundred and five patients started DTG/RPV during the study period: 82 (78.1%) virologically suppressed, 22 (20.9%) non-virologically suppressed (eight failures and 14 restart of ART) and one naïve, who was not included for analysis. There were 70.5% men, mean age was 51.9 years, mean time of HIV infection 214.7 (IQR 140.4–288.9) months, and mean time on the prior ART was 37.0 (IQR 7.8–68.2) months. The most frequent reasons for switching were toxicity or intolerance (41.9%), convenience (27.6%) and drugs interactions (17.1%). Prior regimens were based on PI (56.9%), integrase inhibitors (26.5%) or non-NRTI (16.7%). At this time 85 patients have completed 24 weeks and all were still taking the same regimen, 82 (96.5%) of them with undetectable viral load; the three cases with detectable HIV RNA (532, 316 and 75 copies/mL, respectively) were not considered virological failures. Mean CD4 cells count increased (622 vs. 552 cells/µL; p=0.008), and a mean decrease in fasting triglycerides (−34.6 mg/dL; p=0.005) and glomerular filtration (−5.2 mL/min; p=0.004) were observed, with no changes detected in total cholesterol, HDL-c, LDL-c, creatinine and GPT. No patient stopped DTG/RPV due to adverse events.

**Conclusions**: DTG/RPV is effective and safe in a cohort of patients with long time of HIV infection and prior ART. Most patients changed from more complex regimens. Toxicity, intolerance, convenience and interactions were the main reasons for changing. At 24 weeks lipid profile improved with a decrease in triglycerides.

# Sub article: P055 Efficacy, safety and patient-reported outcomes from treatment-experienced subjects in routine clinical practice switching to EVG/COBI/FTC/TAF (GenvoyaÂ^®^): the German TAFNES cohort

**Published:** 2016-10-23

**Authors:** Benjamin Schleenvoigt, Heiko Jessen, Michael Waizmann, Ramona Pauli, Ansgar Rieke, Nils Postel, Thomas Heuchel, Christian Schulz, Markus Mueller, Arend Moll, Christoph Stephan, Christoph Spinner, Richard Haubrich, Marion Heinzkill, Carolin Wieszner, Hans-Juergen Stellbrink

**Affiliations:** 1 University Hospital, Jena, Germany; 2 Gemeinschaftspraxis Jessen, Berlin, Germany; 3 Praxis Waizmann, Leipzig, Germany; 4 Gemeinschaftspraxis Becker/Pauli, Munich, Germany; 5 Gemeinschaftsklinikum Koblenz-Mayen, Kemperhof, Koblenz, Germany; 6 Prinzmed, Munich, Germany; 7 MEDCENTER, Medizinisches Versorgungszentrum, Chemnitz, Germany; 8 Otto-von-Guericke, Universitätsklinikum, Magdeburg, Germany; 9 Gemeinschaftspraxis Dres., Ulmer, Frietsch, Mueller, Roll, Stuttgart, Germany; 10 Praxiszentrum Kaiserdamm, Berlin, Germany; 1 HIV Center, Universitätsklinikum Frankfurt, Frankfurt, Germany; 2 University Hospital, Klinikum der Rechts der Isar, Munich, Germany; 3 Gilead Sciences, Foster City, CA, USA; 4 Gilead Sciences, Martinsried, Germany; 5 ICH Study Center, Hamburg, Germany

## Abstract

**Introduction**: Tenofovir alafenamide (TAF), a novel prodrug of TFV with equal virologic potency of TDF and 91% lower circulating levels of plasma TFV and fewer off-target effects on renal and bone, was approved based on large controlled clinical trials of antiretroviral therapy (ART) naïve and experienced subjects. As no data are available in patients in routine clinical practice, TAFNES was developed to evaluate the effectiveness and safety of TAF-based regimens, starting with Genvoya^®^ (EVG/COBI/FTC/TAF), the first approved combination including TAF, in treatment-experienced (TE) and treatment-naïve (TN) HIV-infected patients.

**Methods**: TAFNES is an ongoing prospective, observational cohort study, which planned to enrol approximately 150 TN and 150 TE adult subjects initiated or switched to Genvoya^®^ (EVG/COBI/FTC/TAF) in routine clinical practice in accordance with the summary of product characteristics (SmPC). Of clinical outcome variables, only data assessed during the usual management of patients were captured in the electronic case report form (eCRF). Self-reported health-related quality of life (HRQOL) was evaluated using the SF-36, HIV symptom index (SI) and HIV treatment satisfaction (TS) questionnaires. Here we present preliminary results in TE patients with 3-month follow-up at time of data analysis.

**Results**: The analysis population consists of 115 TE patients without documented baseline resistance to any component of EVG/COBI/FTC/TAF and with available follow-up data at month 3. The corresponding baseline characteristics, including reasons for treatment switch, are shown in Table 1. The majority of patients (90%) were switched from TDF-containing ART (55% from EVG/COBI/FTC/TDF) with 67% exposed to TDF for ≥1 year; 64%, 15% and 15% had been on INI-, NNRTI- or PI-based ART (3% NRTI-sparing, 4% other); 91% of patients (104/114) were switched from suppressed ART (HIV RNA <50 copies/mL). Estimated GFR (MDRD; mL/min/1.73 m^2^) and creatinine clearance (CrCl; Cockroft-Gault; mL/min) were <60 in 8.0% and 9.4%, respectively. At month 3, HIV RNA was <50 copies/mL in 90% of patients (94/104; as-treated analysis). Median changes in serum creatinine and CrCl (eGFR) were 0.0 mg/dL and −0.2 mL/min (−0.1 mL/min/1.73 m^2^) (p=n.s.), respectively. Overall, HIV SI significantly decreased (−2.2 (mean), p=0.046); mean changes in SF-36 mental (+1.9) and physical scores (+0.2) were non-significant. The mean post-BL TS change of +14.4 (general satisfaction/clinical subscale +7.5; lifestyle/ease subscale +6.9) reflected a significant improvement, overall, irrespective of previous ART (p<0.001).

**Conclusion**: Of the treatment-experienced patients switching to Genvoya^®^ (EVG/COBI/FTC/TAF), 55% switched from Stribild^®^ (EVG/COBI/FTC/TAF) and 45% from other regimens. While eGFR and CrCl remained stable in this preliminary analysis of 3-month follow-up data, HIV symptom index and treatment satisfaction improved significantly.

**Abstract P055–Table 1:** Baseline characteristics in TE experienced patients

		Previous ART	
			
	EVG/COBI/FTC/TDF (Stribild^®^, STB)	TDF-based ART (not STB)	Non-TDF-based ART
N (%)	63 (54.8)	41 (35.7)	11 (9.6)
Male gender, n (%)	60 (95.2)	36 (87.8)	10 (90.9)
White race, n (%)	59 (93.7)	36 (87.8)	11 (100.0)
Median age, years (IQR)^a^	41 (34–52)	46 (35–54)	56 (48–64)
Age <50 years, n (%)^a^	44 (69.8)	24 (58.5)	3 (27.3)
Median CD4 count, cells/µL (IQR)	648 (480–886)	671 (519–892)	678 (609–915)
HIV RNA level <50 cp/mL, n (%)	57 (91.9)	37 (90.2)	10 (90.9)
Reasons for switch to E/C/F/TAF, n (%) (multiple responses allowed)			
Simplification of ART	13 (20.6)	22 (53.7)	4 (36.4)
Patients preference	25 (39.7)	18 (43.9)	2 (18.2)
Side effects of current ART	22 (34.9)	21 (51.2)	6 (54.5)
Other	8 (12.7)	3 (7.3)	2 (18.2)
Median serum creatinine, mg/dL (IQR) (range)	1.0 (0.9–1.1) (0.6–2.4)	1.0 (0.9–1.2) (0.5–1.7)	1.1 (0.8–1.2) (0.7–1.5)
Median eGFR (MDRD), mL/min/1.73m2 (IQR) (range)	87 (76–100) (34–145)	84 (69–92) (45–219)	79 (66–107) (50–123)
eGFR (MDRD) < 60 mL/min/1.73m2, n (%)	4 (6.6)	4 (10.0)	1 (9.1)
Median CrCl (Cockroft-Gault), mL/min (IQR) (range)	102 (84–122) (38–162)	92 (74–114) (46–179)	99 (62–118) (53–235)
CrCl (Cockroft-Gault) < 60 mL/min, n (%)	4 (7.0)	5 (12.8)	1 (10.0)
Mean SF-36 score mental component (±SD)^b^	45.4 (12.0)	41.5 (13.9)	44.7 (15.2)
Mean SF-36 score – physical component (±SD)^a^,^b^	56.0 (6.9)	51.3 (11.9)	47.0 (11.8)
Mean HIV SI^c^ (±SD)	14.0 (13.7)	18.3 (15.1)	18.7 (12.9)
Mean HIV TS score (±SD)	50.7 (10.5)	49.0 (9.8)	51.9 (5.5)

IQR, interquartile range; SD, standard deviation.

a p < 0.05 (for 3-group comparison)

b norm-based scoring, higher scores indicate higher HRQOL

c range 0–80, higher scores indicate more bothering symptoms; range 0–60, higher scores indicate greater satisfaction.

# Sub article: P056 Frailty improves in both young and old HIV patients undergoing atazanavir-based regimens

**Published:** 2016-10-23

**Authors:** Giovanni Guaraldi, Andrea Malagoli, Giovanni Dolci, Federica Carli, Marianna Menozzi, Antonella Santoro, Stefano Zona, Cristina Mussini

**Affiliations:** 1 Infectious Diseases Unit, University of Modena and Reggio Emilia, Modena, Italy

## Abstract

**Introduction**: Biologic ageing is a stochastic process that can be characterized by the number of health deficits individuals accumulate. Probabilities of health transitions with age can be summarized using a transition model based on the frailty index (FI). Though health generally worsens with age, the relationship between ageing and health is dynamic, and periodic improvement and stability in health are common. Useful models of biologic ageing allow for changes in health that include improvement, maintenance and deterioration. The objective of this analysis was to describe frailty index change in HIV patients, below and above the age of 50 years, undergoing effective atazanavir (ATV)-based regimens.

**Materials and methods**: Design: Secondary analysis of prospective cohort data. We analyzed baseline and 4-year follow-up data from participants in the Modena HIV Metabolic Clinic cohort study, undergoing boosted or unboosted ATV-based regimens and experiencing HIV viral load below 40 copies/mL. Patients were stratified according to the duration of HIV infection (>20, 10–20 and <10 years). Frailty was quantified using a 31-item frailty index. The outcome measure was probability to reduce frailty index score <0.3 which identify the transition from a frail to a fit health status.

**Results**: A total of 406 patients were included: 262 (64.53%) undergoing boosted-ATV and 144 (35.47%) unboosted-ATV regimens. Table 1 describes the study population, stratified below and above 50 years of age. In Figure 1 panel A depicts the FI change in the follow up period and panel B shows the logistic regression model to identify predictors of a frail to fit health status transition.

**Discussion**: HIV patients on stable ATV-based regimen experience improvement in health status depicted by a reduction in FI. Longer duration of HIV infection and baseline frailty index but not age or boosted versus unboosted ATV regimen were associated with a frail to fit health status transition. This study underlines the versatility of ATV-based regimens in both younger and older HIV patients with different spectrum of health profile.

**Abstract P056–Figure 1:** (a) FI change in the follow-up period; (b) logistic regression model to identify predictors of a frail to fit health status transition.

**Abstract P056–Table 1:** Study population, stratified below and above 50 years of age

	Total	<50 years	>50 years	p
Number of patients (%)	406	262 (64.53%)	144 (35.47%)	
Age (SD)	47.91 (7.8)	43.55 (4.58)	55.85 (5.96)	
Females (%)	146 (35.96%)	103 (39.31%)	43 (29.86%)	0.073
BMI (SD)	23.84 (3.59)	23.41 (3.29)	24.63 (3.97)	0.001
Pack-years (IQR)	15.81 (0.5–26.12)	12.75 (0–22.44)	17.05 (2.88–33)	0.003
CD4 nadir (cells/µL) (IQR)	200 (100–300)	204 (104–300)	189.5 (80–292.25)	0.162
Current CD4 (cells/µL) (IQR)	596 (440–800)	620 (447–812.5)	575.5 (431.75–756.25)	0.265
CD4/CD8 (SD)	0.8 (0.43)	0.82 (0.43)	0.77 (0.44)	0.089
ART exposure (months) (IQR)	34.5 (12–60)	39.5 (15.25–64.75)	21 (9–49.5)	0.003
CVD (%)	19 (4.68%)	7 (2.67%)	12 (8.33%)	0.001
Hypertension (%)	139 (34.24%)	68 (25.95%)	71 (49.31%)	0.001
Osteoporosis (%)	83 (20.44%)	38 (14.5%)	45 (31.25%)	0.001
Diabetes (%)	48 (11.82%)	15 (5.73%)	33 (22.92%)	0.001
CKD (%)	42 (10.34%)	12 (4.58%)	30 (20.83%)	0.001
Cancers (%)	32 (7.88%)	13 (4.96%)	19 (13.19%)	0.001
Multimorbidity (%)	32 (7.88%)	4 (1.53%)	28 (19.44%)	0.001
Frailty index (SD)	0.34 (0.1)	0.33 (0.1)	0.37 (0.09)	0.001

# Sub article: P057 High-level HIV drug resistance mutations in patients with unsuppressed viral load from Northern South Africa

**Published:** 2016-10-23

**Authors:** Elizabeth Etta, Cecile Manhaeve, Keanan McGonigle, David Rekosh, Marie-Louise Hammarskjold, Denis Tebit, Pascal Bessong

**Affiliations:** 1 Microbiology/ Infectious Diseases, University of Virginia, Charlottesville, VA, USA; 2 Microbiology, Bela-Bela Clinic, HIV/AIDS Prevention Group, Polokwane, South Africa; 3 Microbiology/Infectious Diseases & Cancer Biology, University of Virginia, Charlottesville, VA, USA; 4 Microbiology/Virology, University of Venda, Polokwane, South Africa

## Abstract

**Introduction**: Access to cART has significantly improved in the developing world, with significant reduction in morbidity and mortality. However, the sustainability of cART may be compromised by development of drug resistance as evidenced by unsuppressed viral load. The current study examined drug resistance mutations (DRMs) in individuals under first-line cART with suspected unsuppressed viral load.

**Materials and methods**: Sixty patients on first-line cART were recruited between March 2014 and September 2015, from two rural treatment sites in Limpopo Province, South Africa if they met either of the following criteria: 1) two consecutive viral loads measurements greater than 1000 copies/mL after a previous suppression or 2) one viral load greater than 1000 copies/mL after 180 days. Nested PCR gene products from viral RNA (plasma) and proviral DNA (peripheral blood mononuclear cells (PBMCs)) were directly sequenced to determine subtype and examined for protease and reverse transcriptase inhibitors resistance mutations according to the Standard HIV Drug Resistance Interpretation Algorithm.

**Results**: Sequences obtained from 57 patients were examined for subtype and DRMs. Fifty-two (91.2%) of the 57 were HIV-1 subtype C in the polymerase gene with one each (1.8%) of subtype B, K/C, C/B recombinants and unclassified. These 52 (91.2%) patients harboured at least one major DRM which were distributed as follows: NRTI (n=13; 25.0%), NNRTI (n=17; 32.7%) and PI (n=3; 5.7%). The most common mutations were M184V (56%), K103N (50%), V106M (17.3%), K65R (11.5%), D67N (9.6%). Mutation scores suggest that the viruses were mostly resistant to 3TC, FTC and NVP, and most susceptible to d4T, TDF and AZT. Two subjects with viral load of 1000 copies/mL carried DRM in PBMC but not in plasma.

**Conclusion**: A very high prevalence of drug resistance-associated mutations was recorded in patients still on first-line cART. The differences in circulating DRM in plasma and PBMCs in some subjects suggest the presence of archived drug resistant variants. PBMC is therefore an interesting compartment for analyzing the dynamics of drug resistance in a given patient.

# Sub article: P058 Real-life experience of switching to protease inhibitor-based dual antiretroviral therapy (PIDAT)

**Published:** 2016-10-23

**Authors:** Neal Marshall, Marie McNulty, Colette Smith, Leonie Swaden, Fiona Burns, Chloe Orkin

**Affiliations:** 1 Ian Charleson Centre for HIV Medicine, Royal Free London NHS Foundation Trust, London, UK; 2 Department of HIV Medicine, Bart's Health NHS Trust, London, UK; 3 Infection and Population Health, University College London, London, UK

## Abstract

**Introduction**: PI-based dual antiretroviral therapy (PIDAT) strategies in suppressed patients have shown variable virological efficacy, depending partly on accompanying drug class choice. We reviewed outcomes of PIDAT switches within two metropolitan HIV centres.

**Materials and methods**: Retrospective evaluation of all patients switching to a boosted protease inhibitor (bPI) with a drug from another ARV class from 1 January 2009 till 1 July 2014 with subsequent follow-up until 1 June 2016. Baseline demographics and treatment history were identified, with follow-up data at 48 and 96 weeks. Primary analysis included percentage remaining on any PIDAT strategy (i.e. switch within paradigm allowed), with secondary analysis considering any change of ART in the PIDAT regimen as a switch. Data were analyzed using Fisher's exact test.

**Results**: Of 255 patients identified, 239 (94%) and 226 (89%) remained under follow-up at 48 and 96 weeks, respectively. Hundred and ninety-nine (78%) were male, 171 (67%) white ethnicity, 167 (65%) MSM, with median age and time on ART of 47 and 12.1 years, respectively. Seventy-seven percent (196) had VL <50 copies/mL at switch with median (IQR) CD4 count 632 (439–839) cells/mm^3^ and nadir CD4 125 (32–207). Two hundred and twenty-six of 255 (89%) switched from PI-based ART with 64 (25%) switched or intensified from another nucleoside-sparing regimen, including 36/255 (14%) PI monotherapy. NNRTI-based PIDAT was initiated in 103 (40%) patients, 87/103 (84%) with etravirine. Maraviroc was used for 81/255 (32%), 50 (20%) a NRTI (30/50 as 3TC or FTC) and 21 (8%) with raltegravir. Darunavir/r was used in 196 (77%) cases. At 48 and 96 weeks respectively, 177/210 (84%) and 145/184 (79%) remained on any PIDAT regimen with VL <50 copies/mL respectively (switch from PIDAT=failure, missing=excluded). Using ITT for the initial PIDAT regimen, 188/210 (90%) and 169/184 (92%) were VL <50 copies/mL at 48 and 96 weeks. There were statistically significant differences in VL outcomes by choice of second ARV at both time points (Table 1), with no differences by CD4 nadir. Nineteen of 239 (8%) and 42/226 (19%) discontinued PIDAT by 48 and 96 weeks, respectively, 9/19 (47%) and 22/42 (52%) with VL >50 copies/mL and 2/226 with available genotypes demonstrating emergent resistance by 96 weeks (Y181C on DRV/r/ETR at week 39, and K103N, V106A on LPV/r/NVP at week 20).

**Conclusions**: Real-life outcomes of PI-based dual ARV therapy appear broadly favourable in clinical practice. The ongoing utility of this paradigm in the advent of TAF and PI-sparing regimens is unclear.

**Abstract P058–Table 1:** Switch and VL outcomes of individuals starting PI/r-based dual therapy regimens

Second ARV	N starting ARV (%)	48 weeks (n = 239)			96 weeks (n = 226)		

		ITT: VL <50 copies/mL^a^	On PIDAT and VL <50 copies/mL^b^	On specific PIDAT regimen and VL <50 copies/mL^c^	ITT: VL <50 copies/mL^a^	On PIDAT and VL <50 copies/mL^b^	On specific PIDAT regimen and VL <50 copies/mL^c^
		p = 0.0013	p = 0.005	p = 0.02	p = 0.60	p = 0.004	p = 0.03
CCR5 inhibitor	81	62/69 (90%)	58/69 (84%)	57/69 (83%)	56/62 (90%)	45/62 (73%)	43/62 (69%)
INSTI	21	7/13 (54%)	6/13 (46%)	6/13 (46%)	6/7 (86%)	2/7 (29%)	2/7 (29%)
NNRTI	103	89/94 (95%)	84/94 (89%)	80/94 (85%)	77/82 (94%)	71/82 (87%)	65/82 (79%)
NRTI	50	30/34 (88%)	29/34 (85%)	29/34 (85%)	30/33 (91%)	27/33 (82%)	25/33 (76%)

a Among all with available VL measurement, ignoring ART changes

b numerator is number with VL <50 copies/mL who remain on PIDAT strategy, denominator is those under follow-up with available VL measurement at 48/96 weeks

c numerator is number with VL <50 copies/mL who remain on original specific PIDAT regimen, denominator is those under follow-up with available VL measurement at 48/96 weeks.

# Sub article: P059 Long-term virological outcomes of replacing zidovudine or stavudine with tenofovir in the absence of routine virological monitoring in Kumasi, Ghana

**Published:** 2016-10-23

**Authors:** Giovanni Villa, Richard Odame Phillips, Colette Smith, Alexander Stockdale, Apostolos Beloukas, Lambert Tetteh Appiah, David Chadwick, Alessandra Ruggiero, Fred Stephen Sarfo, Anna Maria Geretti

**Affiliations:** 1 Institute of Infection and Global Health, University of Liverpool, Liverpool, UK; 2 Department of Medicine, Kwame Nkrumah University of Science and Technology and Komfo Anokye Teaching Hospital, Kumasi, Ghana; 3 Infection and Population Health, University College London, London, UK; 4 Department of Medicine, Komfo Anokye Teaching Hospital, Kumasi, Ghana; 5 Centre for Clinical Infection, James Cook University Hospital, Middlesbrough, UK

## Abstract

**Introduction**: Whilst access to ART is successfully expanding in Africa, long-term outcomes remain poorly investigated. This study addressed the outcomes of introducing tenofovir (TDF) in place of zidovudine (ZDV) or stavudine (d4T) among Ghanaian adults receiving HIV care in the absence of routine virological monitoring and determined the associated clinical and psychosocial dimensions.

**Methods**: The Hepatitis B Infection in Kumasi (HEPIK) study has prospectively followed HIV/HBV co-infected adults since 2010. This cross-sectional analysis comprised subjects that had previously started ZDV or d4T plus lamivudine and efavirenz or nevirapine, and at the time of HBV diagnosis (T0), replaced ZDV or d4T with TDF in the absence of virological monitoring. A median of 7.9 (IQR 6.0–9.2) years after starting ART and 4.0 (3.8–4.1) years after introducing TDF (T1, November 2015), patients were invited to attend for assessment, including HIV-1 RNA load, and offered a researcher-administered questionnaire about adherence (visual analogue scale and targeted questions); socio-economic, social support and disclosure status; and physical and mental health. Plasma viral load at T0 was determined retrospectively using stored (−80°C) samples.

**Results**: A total of 101/180 (56%) invited participants (66% females) attended the T1 assessment. Of the remaining, 47 (26%) were no longer contactable (≥3 attempts), 17 (9%) declined to attend and 15 (8%) had died. At T1, mean age was 45 (±9) years; 90% were still receiving efavirenz (n=87) or nevirapine (n=4); 10% were on lopinavir/ritonavir (LPV/r). CD4 counts were median 572 (383–716) cells/mm^3^. Suboptimal adherence was reported by 42% of participants; in univariable analysis, it was more prevalent among men (p<0.01), those in a relationship (p=0.02) and those with higher socio-economic status (p=0.04). Moderate-to-severe depression/anxiety was reported by 27%; 64% described moderate-to-severe physical distress. HIV-1 RNA was detectable (>40 copies/mL) in 21%, and >1000 copies/mL in 14%, with median levels of 4.2 (2.1–5.1) log_10_ copies/mL. In univariable analysis, predictors of lack of virological suppression comprised the CD4 cell count at diagnosis (p=0.03), T0 viral load (p=0.05), suboptimal adherence (p<0.01), lack of partner disclosure (p<0.01) and LPV/r use (p=0.03). Lack of virological suppression was also associated with lower T1 CD4 cell counts (p<0.01). There was no association with socio-economic/social support status, or physical/mental health.

**Conclusion**: One in five subjects receiving long-term ART showed suboptimal virological suppression with reduced CD4 cell count recovery. The findings highlight the importance of viral load testing at key management time points, coupled with targeted interventions to support adherence and facilitate partner disclosure.

# Sub article: P060 Non-adherence in HIV patients is caused by specific reasons: results from the German adherence cohort study

**Published:** 2016-10-23

**Authors:** Johanna Boretzki, Carmen Wiese, Celia Oldenbuettel, Ivanka Krznaric, Anja Meurer, Alexander Zink, Christian Lersch, Annamaria Balogh, Eva Wolf, Christoph Spinner

**Affiliations:** 1 Department of Medicine II, University Hospital Klinikum rechts der Isar, Munich, Germany; 2 Private Practice, MVZ Karlsplatz, Munich, Germany; 3 Private Practice, Zentrum für Infektiologie Berlin, Berlin, Germany; 4 Private Practice, Zentrum fuer Innere Medizin und Infektiologie, Munich, Germany; 5 Department of Dermatology and Allergology, University Hospital Klinikum rechts der Isar, Munich, Germany; 6 MUC Research, Munich, Germany

## Abstract

**Introduction**: Adherence to antiretroviral treatment (ART) in HIV patients plays a crucial role for treatment success. Our study aimed to identify reasons for non-adherence in a large HIV cohort, including known subjects with difficulties in ART adherence.

**Methods**: A cross-sectional, non-interventional, multicentre adherence study in treated HIV-infected patients from September 2014 to April 2015 in Germany was performed after ethic committee's approval. Study physicians were asked to recruit patients from all adherence levels and perform an adherence assessment for each subject (good, unstable or poor adherence). Questionnaires based on the SMAQ-MASRI-Hybrid [1] were given to the patient and treating physician to evaluate factors associated with poor adherence. Covariables of interest were age, sex, time since HIV diagnosis, time on ART, current ART regimen, transmission route, comorbidity, HIV-1 RNA viral loads (VLs) and CD4 cell count. Furthermore, specific reasons for non-adherence were assessed. For statistical analysis, extended Fisher's exact test and Kruskal–Wallis test were used.

**Results**: A total of 215 patients were included: 80% male, median age 47 years (IQR 37–54), median time since HIV diagnosis 9 years (IQR 4–18) and median CD4 cell count 607 c/L (IQR 410–850). HIV transmission risk was as follows: 50% men having sex with men, 14% origin of high prevalence countries (HPC), 7% intravenous drug use (IVDU), 13% other and 19% unknown. Subjects were grouped by physicians’ adherence assessment: A, “good adherence” n=162; B, “unstable adherence” n=36; C, “poor adherence” n=17. Physicians’ assessment of poor adherence correlated in univariate analyses with lower median age (A: 48 years vs. B: 42 vs. C: 46, p=0.020), origin of HPC (A: 11% vs. B: 19% vs. C: 29%, p<0.01), IVDU (A: 1.9% vs. B: 22% vs. C: 24%, p<0.01), hepatitis C infection (A: 3.7% vs. B: 17% vs. C: 5.9%, p=0.013), psychiatric disorders (A: 25% vs. B: 42% vs. C: 48%, p=0.03), longer time since HIV diagnosis (A: 9 years vs. B: 10 vs. C: 19, p<0.01), longer time on ART (A: 6 years vs. B: 5 vs. C: 14, p=0.022), AIDS-defining events (A: 6.8% vs. B: 25% vs. C: 24%, p<0.01), prescription of a protease inhibitor (A: 28% vs. B: 47% vs. C: 71%, p<0.01), higher median VL (A: 19 copies/mL vs. B: 49 vs. C: 4824, p<0.01) and lower median CD4 cell count (A: 680 c/L vs. B: 503 vs. C: 315, p<0.01). Sex, comedication and pill burden were not significantly associated. Physicians’ assessment of poor adherence correlated in univariate analyzes with self-reported, specific reasons (Table 1).

**Conclusion**: Adherence evaluation remains challenging. Frequent self-reported reasons for non-adherence were reminding of disease, concerns about interaction between ART and alcohol/party drugs and HIV stigma. A good patient–provider relationship is needed to face those topics and to remove common barriers to adherence.

**Abstract P060–Table 1:** Overview of questionnaire items and correlation with adherence levels

	“Good adherence” (n = 162)	“Unstable adherence” (n = 36)	“Poor adherence” (n = 17)	p-value (Fisher's exact test)
The ART intake reminds me of my disease	n = 7 (4.3%)	n = 4 (11%)	n = 5 (29%)	<0.01
I want to go out/I think my medication does not go well with alcohol/party drugs	n = 3 (1.9%)	n = 5 (14%)	n = 4 (24%)	<0.01
I'm afraid that others see me taking the ART medication	n = 4 (2.5%)	n = 4 (11%)	n = 2 (12%)	0.019
I think that the ART dose is too high	n = 3 (1.9%)	n = 4 (11%)	n = 1 (5.9%)	0.037
Sometimes the copayment fee to my ART is too much for me/other financial reasons	n = 2 (1.2%)	n = 3 (8.3%)	n = 1 (5.9%)	0.037

## References

[1] Kuhlmann B, Liess H Leitlinien der DAGNAE zur Unterstuetzung der Adhaerenz im Rahmen einer antiretroviralen Therapie bei HIV-Infektion.

# Sub article: P061 Prevalence of HIV virological failure in a multidisciplinary centre treating high-risk vulnerable populations

**Published:** 2016-10-23

**Authors:** Arshia Alimohammadi, Tyler Raycraft, Ghazaleh Kiani, Rajvir Shahi, Arpreet Singh, Syune Hakobyan, Brian Conway

**Affiliations:** 1 Clinical Research, Vancouver Infectious Diseases Centre, Vancouver, Canada

## Abstract

**Introduction**: The introduction of new ART has considerably increased the efficacy of HIV regimens and decreased mortality rates associated with the infection [1,2]. However, adherence to even the simplest and most effective regimens remains challenging in people who inject drugs (PWID) [2], and this often limits their access to treatment as compared to “lower risk” populations [3]. Provision of care in a dedicated multidisciplinary setting may help increase success of HIV treatment in this population.

**Methods**: An observational, retrospective study was conducted among HIV-infected patients seen at the Vancouver Infectious Diseases Centre (VIDC), including all PWID who received HIV therapy. All individuals had access to multidisciplinary care to address medical, psychiatric, addiction-related and social needs with maintenance in long-term follow-up at our centre. Virological failure was defined as initial suppression of HIV viral load, followed by a confirmed measure >200 copies/mL, comparing PWID and non-PWID in this analysis.

**Results**: Since 2013, 521 HIV+ patients have initiated treatment at VIDC, with a mean age of 51.6 years, 11% female. Overall, 179 (32%) are active PWID or have a history of recent injection drug use, with HIV/HCV co-infection prevalence of 94% in this subgroup. Within the PWID population, 63% used heroin, 59% used cocaine, 70% used other stimulants and 49% were on opiate substitution therapy. In total, rates of virological suppression were 77% and 84% in PWID and non-PWID subgroups (p<0.05). Only five (3%) PWID and two non-PWID (0.6%) experienced true virological failure while on treatment. All individuals who experienced virological failure were switched to new regimens and subsequently achieved virological suppression.

**Conclusion**: When HIV care and treatment are implemented within a multidisciplinary setting, high-risk populations are highly likely to achieve HIV RNA suppression, at rates nearly comparable to those achieved in the general HIV-infected population. Those who did not were all successfully treated with readily available second-line regimens. Novel models of care will be needed to allow HIV-infected PWID to achieve the promise of “90–90–90” within the global response to the HIV pandemic.

## References

[1] Arnsten JH, Demas PA, Farzadegan H, Grant RW, Gourevitch MN, Chang C-J (2001). Antiretroviral therapy adherence and viral suppression in HIV-infected drug users: comparison of self-report and electronic monitoring. Clin Infect Dis.

[2] Spire B, Lucas GM, Carrieri MP (2007). Adherence to HIV treatment among IDUs and the role of opioid substitution treatment (OST). Int J Drug Policy.

[3] Petersen Z, Myers B, van Hout M-C, Plüddemann A, Parry C (2013). Availability of HIV prevention and treatment services for people who inject drugs: findings from 21 countries. Harm Reduct J.

# Sub article: P062 Methadone maintenance treatment and efficiency of ART in HIV-positive injecting drug users in Ukraine

**Published:** 2016-10-23

**Authors:** Dmytro Zhyvytsia, Vitali Kazeka

**Affiliations:** 1 Department of Infectious Diseases, SI Zaporizhia Medical Academy of Post-Graduate Education Ministry of Health of Ukraine, Zaporizhia, Ukraine

## Abstract

**Introduction**: The current state of the HIV epidemic process in Ukraine is characterized by the prevalence of HIV among different contingents of the population, especially among people who belong to the high-risk groups and by change in the dominant routes of HIV transmission. Although the most common way of HIV transmission is the sexual one, injecting is still the most important in its impact on the epidemic. The study evaluated the impact of methadone maintenance treatment (MMT) on efficiency of ART in HIV-infected injecting drug users (IDUs).

**Materials and methods**: The study included 65 HIV IDUs who were divided into two groups. First group included 33 HIV IDUs who were on MMT. The average time of MMT was 23.7 months (1–60). The second group included 32 HIV IDUs who did not receive MMT. The average age of patients was 37 years (24–52). There were 16 (25%) women and 49 (75%) men. The average level of viral load in the studied groups of patients was not statistically different, and in the first group it was 4.89 (4.1–5.2) log copies/mL, in the second 5.0 (4.2–5.6) log copies/mL. After the enrollment in the study, the ART has been prescribed to all patients in accordance with the Ukraine clinical protocol.

**Results**: After 6 months of ART, the proportion of patients with complete suppression of HIV (HIV RNA1 <50 copies/mL) in the first group was higher than in the second group; however, this difference was not significant (75.8% and 61.3%, respectively, p=0.21). When assessing efficiency of ART after 12 months of observation, the significantly higher (p<0.01) percentage of patients in the first group who achieved a complete viral suppression compared with the second group were 93.9% and 58.1%, respectively. In the second group of patients within 6–12 months of treatment, there was a decrease in the proportion of patients with virologic efficiency of ART – from 61.3% to 58.1% of patients with the full viral suppression.

**Conclusions**: The study indicates that the use of MMT in HIV-infected IDUs greatly increases the efficacy of ART, which can be associated with a significant increase in adherence to the treatment in this category of patients with mental and behavioural disorders due to the use of psychoactive substances containing opioids.

# Sub article: P063 Community pharmacy dispensed ART alongside opiate replacement therapy in Glasgow's HIV outbreak

**Published:** 2016-10-23

**Authors:** Seija Erica Peters, Kathryn Brown, Amanda Laird, Robert Gillespie, David Thomson, Catriona Milosevic

**Affiliations:** 1 Brownlee Centre, Gartnavel General Hospital, Glasgow, UK; 2 Brownlee Centre Pharmacy, Gartnavel General Hospital, Glasgow, UK; 3 Addictions, Advanced Pharmacist, Possilpark Health Centre, Glasgow, UK; 4 Community Pharmacy Development & Governance, NHS Greater Glasgow and Clyde, Glasgow, UK; 5 Community Pharmacy Development & Governance, West Glasgow Ambulatory Care Hospital, Glasgow, UK; 6 Public Health, Gartnavel Royal Infirmary, Glasgow, UK

## Abstract

**Introduction**: There is currently an outbreak of HIV in people who inject drugs (PWID) in Glasgow, UK. Improved access to care/ART is essential for individuals, and for reduction of onward transmission. Traditionally, ARV medication is prescribed and dispensed via a hospital-based service. Patients are required to attend a single hospital in the city for HIV care. Addiction services including opiate replacement therapy (ORT) are well developed in Glasgow inclusive of homelessness. We developed a method of delivering ART alongside opiates using community pharmacies. We describe the development process and will present the first 6 months of this project.

**Materials and methods**: One strategy to manage this outbreak was to improve access to clinical care and ART. Our hospital HIV pharmacist led a group to develop pathways for ARV dispensing via community pharmacies. This was done in consultation with community and hospital pharmacies, drug companies, hospital and addiction healthcare workers. A funding model was agreed. An HIV liaison nurse from the hospital but working in the community facilitates patient engagement. An HIV nurse-led clinic has been set up in the homeless health centre to support monitoring. Prescriptions are generated from the hospital physician and the patient receives the medication from the pharmacy. There is no restriction on ARV choice. ART is dispensed daily with patients receiving supervised consumption if required. Patients requiring a twice-daily regimen are provided with the second dose to take at home. The patient does not have to attend the hospital. Checks are in place to inform prescribers of poor adherence/disengagement in care.

**Results**: A total of 79 patients' records have been reviewed to see if they would potentially benefit from community prescribing. Most report a history of homelessness and have links with addiction services. We will present: (1) a description of the model of care including monitoring and safety checks, (2) evaluation of the first 6 months of patient data from April 2016.

**Conclusions**: ART has individual and public health benefits. For PWID, traditional models of hospital-based care can be challenging. Homelessness limits engagement in care. Addiction services and involvement of the community pharmacy network with this patient group are well established in Glasgow. Adapting our ARV prescribing to fit in with existing community ORT models may improve access to ART and HIV care.

# Sub article: P064 HIV testing and care in prisoners: the first year results of opt-out BBV testing in Glasgow, UK

**Published:** 2016-10-23

**Authors:** Seija Erica Peters, Brenda Bissett, Yvonne Cassells, Jennifer Paton, Celia Aitken

**Affiliations:** 1 Brownlee Centre, Gartnavel General Hospital, Glasgow, UK; 2 Addictions, HMP Barlinnie, Glasgow, UK; 3 Health Centre, HMP Low Moss, Bishopbriggs, UK; 4 Regional Virus Laboratory, Glasgow Royal Infirmary, Glasgow, UK

## Abstract

**Introduction**: In Glasgow, there is an ongoing outbreak of HIV amongst people who inject drugs (PWIDs). It is well recognized that the prison population has high numbers of PWIDs. In Scotland, there is a national plan for identifying and treating prisoners with HCV [1,2]. As part of increased testing for HCV, an opt-out Blood Borne Viruses (BBV) testing service (including HIV testing) was developed. Barlinnie HMP is the largest prison in Scotland and centrally located in Glasgow. It has >1200 inmates at any one time. We have evaluated our first year figures of opt-out testing in relation to HIV.

**Materials and methods**: All prisoners have a medical within 24 hours of incarceration. From April 2015, a prison nurse and healthcare assistant saw prisoners at the same time as their medical to offer BBV testing to them. We record the uptake of this testing monthly. We reviewed the HIV outbreak data base to identify the number of new HIV diagnoses from the prison sector. We retrospectively reviewed the electronic case record for data on attendance and ARV prescribing.

**Results**: From April 2015 to March 2016, 1492 were BBV tested. Just over 100 refused (data not collected for 3 months). If prisoners had been tested within the last 6 months, a second test was not offered unless at particular risk. In total, 11 cases of HIV were identified. Of that, 10 patients with HIV were seen by the BBV consultant during their incarceration. All were HCV co-infected with only one being PCR negative. Seven were on prescribed opiates. Six were started on ARV therapy within the prison. Four failed to attend any OP clinics after liberation. Only two prisoners have attended all their appointments so far.

**Conclusions**: BBV testing in the prison setting including HIV testing is a feasible way to identify infected PWIDs. This may be the first sign of an HIV outbreak emerging in this population. HIV-positive prisoners are usually HCV co-infected. Good links to specialist care and therapy within the prison may encourage prisoners to test. ARV therapy can be started early and safely. A comprehensive approach to “throughcare” for this population is required to address clinical engagement after liberation. It is hoped that ARV therapy in this group may stem the current outbreak. Further review of retention in care is required.

## References

[1] Scottish Government Hepatitis C action plan for Scotland: phase II: May 2008–March 2011. http://www.scotland.gov.uk/Resource/Doc/222750/0059978.pdf.

[2] Scottish Government The sexual health and blood borne virus framework 2011–15. http://www.gov.scot/Resource/Doc/356286/0120395.pdf.

# Sub article: P065 Correlates of HIV virological non-suppression at a tertiary clinic

**Published:** 2016-10-23

**Authors:** Arpreet Singh, Tyler Raycraft, Arshia Alimohammadi, Ghazaleh Kiani, Rajvir Shahi, Brian Conway

**Affiliations:** 1 HIV/AIDS and HCV, Vancouver Infectious Diseases Centre, Vancouver, Canada

## Abstract

**Introduction**: We wanted to determine which variables and characteristics were associated with HIV virological non-suppression at a tertiary clinic located in Downtown Vancouver.

**Materials and methods**: The multidisciplinary programme developed at the Vancouver Infectious Diseases Centre (VIDC) provides ongoing, long-term access to specialty medical care and support services in order to target the clinical and social factors associated with HIV suppression and maintenance. A retrospective analysis of HIV treatment responses was conducted to study the factors associated with virological non-suppression.

**Results**: We divided the population into four separate cohorts. Group 1 (n=241) included individuals who showed HIV RNA suppression and an increase/no change in CD4 count from baseline. Within this cohort, there were 222 males with a mean age of 50.9 years (range 24–74) and 21 females with a mean age of 51.7 years (range 33–63). Group 2 (n=88) included individuals who showed HIV RNA suppression and a decrease in CD4 count from baseline. Within this cohort, there were 73 males with a mean age of 54.5 years (range 22–82) and 15 females with a mean age of 50.1 years (range 31–64). Group 3 (n=9) included individuals who showed HIV RNA non-suppression and a decrease in CD4 count from baseline. Within this cohort, there were seven males with a mean age of 51.4 years (range 36–71) and two females with a mean age of 46 years (range 44–48). Group 4 (n=7) included individuals who showed HIV RNA non-suppression and an increase/no change in CD4 count from baseline. Within this cohort, there were seven males with a mean age of 57.3 years (range 34–73). Relevant demographic characteristics are shown in Table 1.

**Conclusions**: Virological non-suppression was uncommon, but individuals in these groups showed higher rates of injection drug use, homelessness and unemployment. CD4 cell count trajectories were not associated with any clinical or demographic characteristics measured in this cohort. Acting on characteristics such as drug use and homelessness may help reduce the rate of non-response to antiretroviral therapy in our cohort.

**Table 1:** Socio-demographic characteristics dichotomised into four separate cohorts to present our study population (n = 345)

	Socio-demographic characteristics	Group 1	Group 2	Group 3	Group 4
Ethnicity	Caucasian	34	1	9	1
	First Nations	10	2	8	–
	Other	15	–	9	–
Risk factors	Men who have sex with men	136	4	40	3
	People who inject drugs	74	5	32	5
	Cocaine	49	2	14	1
	Amphetamines	34	4	7	2
	Opioids	33	3	14	–
	Benzodiazepines	14	1	7	–
	Homeless	41	3	20	–
	Not homeless	42	3	20	2
	Employed	45	1	15	1
	Unemployed	30	4	12	2
	Alcohol	67	3	31	1
	No alcohol	49	3	14	1

# Sub article: P066 The effect of relationship status and housing stability on adherence to combination antiretroviral therapy among people living with HIV who use illicit drugs in British Columbia, Canada

**Published:** 2016-10-23

**Authors:** Arpreet Singh, Kate Salters, Surita Parashar, Cathy Puskas, Lu Wang, Julio Montaner, Robert Hogg, Hasina Samji

**Affiliations:** 1 Faculty of Health Sciences, Simon Fraser University, Burnaby, Canada; 2 HIV/AIDS, British Columbia Centre for Excellence, Vancouver, Canada; 3 Epidemiology, BC Centre for Disease Control, Burnaby, Canada

## Abstract

**Introduction**: High adherence to cART is essential for long-term viral load suppression among people living with HIV (PLHIV). While a variety of socio-economic, demographic and clinical characteristics have been associated with suboptimal adherence to cART, we have focussed on the previously unexplored association between relationship status and housing stability on adherence among people who use illicit drugs.

**Methods**: Sociodemographic survey data collected between July 2007 and January 2010 as part of the Longitudinal Investigation into Supportive and Ancillary health services (LISA) cohort and clinical data collected through the provincial Drug Treatment Program (DTP) were used in this study. Study participants were PLHIV ≥19 years of age who used illicit drugs (heroin, crack, cocaine and/or methamphetamine) within 3 months prior to the interview, and currently accessing cART, with pharmacy refill compliance data in the 6-month period prior to the interview. Optimal adherence (≥95%) was the main outcome of interest. The main explanatory variables included housing status (stable vs. unstable housing) and relationship status (single/separated/divorced/widowed (SSDW) or legally married/common law/regular partner/non-regular partner (LCRN)). Separate logistic regression confounder models were used to determine the effect of relationship status and housing stability on the association between illicit drug use and adherence to cART. The combined current crack use and relationship status variable was the main interest in the first model, while the combined current crack use and housing status variable was the main interest in the second model. Confounders were controlled for in both models.

**Results**: This study included 405 individuals, of whom 115 (28%) were women and 5 (1%) were transgender. A total of 261 (65%) of participants achieved optimal adherence, 317 (78%) were currently using crack, 208 (51%) were unstably housed and 122 (30%) were LCRN. The first confounder model showed relationship status (LCRN and SSDW) combined with current crack use, were significantly associated with suboptimal adherence (aOR 2.88, 95% CI 1.22–6.79; and aOR 2.42; 95% CI 1.08–5.42, respectively), as were current crack use and unstable housing in the second confounder model (aOR 2.83; 95% CI 1.332–6.025).

**Conclusion**: Relationship status and housing status did not predict optimal cART adherence independently; however, when combined with current crack use, they were significantly associated with suboptimal adherence to cART. Interventions, particularly those focussed on housing and addictions support services, need to be targeted towards current crack users in order to remove barriers to cART adherence.

# Sub article: P067 HIV support group within a multidisciplinary healthcare delivery model as a treatment strategy for people who inject drugs

**Published:** 2016-10-23

**Authors:** Rajvir Shahi, Ghazaleh Kiani, Arshia Alimohammadi, Tyler Raycraft, Arpreet Singh, Syune Hakobyan, Brian Conway

**Affiliations:** 1 Clinical Research, Vancouver Infectious Diseases Centre, Vancouver, Canada

## Abstract

**Introduction**: Among the 18,000 residents living on the Downtown East Side (DTES) of Vancouver, over 20% are infected with HIV. An innovative approach is needed to engage these individuals in care to fulfill the goals of the “90–90–90” program endorsed by the World Health Organization to address the global HIV pandemic.

**Materials and methods**: In 2013, structured HIV support groups were designed as an innovative strategy to engage this vulnerable population in a multidisciplinary program of care. The group is held once a week for 4 hours, led by medical doctors, nurses and other community-based workers. Individuals attending the group are given a presentation on a topic related to HIV/AIDS, such as HIV transmission, therapy or substance abuse. They are also able to ask medical questions and voice their health-related concerns in an open and safe environment. Two meals are provided as well as services to address medical, psychological, social and addiction-related needs. A retrospective analysis to assess characteristics of individuals attending the group regularly (at least once per month) was performed, and commitment to HIV treatment was evaluated.

**Results**: A total of 74 HIV-infected patients (mean age 52 years, 12% females, 14% First Nations) regularly attended the group. Among these HIV-positive individuals attending the group, 55 (74.3%) were people who actively inject drugs (PWID), 36 (48.6%) self-identified as being homeless and 25 (33.8%) had an underlying psychiatric disease. The majority (73/74, 98.6%) were receiving antiretroviral therapy and 58/73 (79.4%) of these individuals had a suppressed HIV plasma viral load (<40 copies/mL). The remaining 15/73 (20.5%) are on treatment, but have not yet suppressed.

**Conclusions**: This HIV support group model has shown to be effective at engaging and retaining HIV-infected patients in care, with the vast majority having a maximal response to antiretroviral therapy. These individuals were previously undiagnosed or not receiving care. Approaches such as the one we have developed will be essential to reaching the goal of “90–90–90” especially in more vulnerable populations.

# Sub article: P068 Adherence to cART and low-level viremia in a large single-centre clinical cohort

**Published:** 2016-10-23

**Authors:** Franco Maggiolo, Elisa Di Filippo, Annapaola Callegaro, Giorgio Colombo, Sergio Di Matteo, Daniela Valenti, Marco Rizzi

**Affiliations:** 1 Infectious Diseases, ASST Papa Giovanni XXIII, Bergamo, Italy; 2 Laboratory of Microbiology and Virology, ASST Papa Giovanni XXIII, Bergamo, Italy; 3 Drug Sciences, University of Pavia, Pavia, Italy; 4 Health Economics & Outcomes Research, Studi Analisi Valutazioni Economiche (SAVE) Studi, Milano, Italy

## Abstract

**Introduction**: To monitor the use of cART is relevant to evaluate outcomes, adherence to prescriptions and therapeutic strategies.

**Materials and methods**: We analyzed the full cohort of a large reference hospital in Northern Italy. Data on drug use were derived from pharmacy records and were cross-linked with clinical data from the outpatient clinic database. The period from 1 October 2012 to 30 September 2014 was considered for a mean individual follow-up of 1.18 years and a total follow-up of 2676 person-years. Adherence was calculated on the basis of pharmacy refill. We defined virologic response by categories: always <3 copies/mL (K<3), sometimes <3 but always <50 copies/mL (V<3), always >3 but always <50 copies/mL (K>3) and sometimes >50 copies/mL (V>50).

**Results**: Over the considered 2 years, 2589 HIV+ subjects were prescribed ARV drugs. According to univariate analysis, adherence correlated with several baseline variables: hepatitis co-infection (p=0.002), nationality, risk factor for HIV infection, third drug included into the regimen, line of therapy, time on cART and age (p<0.0001 for all). When entered in a multivariate model, only nationality (p=0.002), time on cART (p=0.005), age (p<0.0001) and the third drug into the regimen (p<0.0001) retained statistical significance. Adherence was higher for NNRTI-based regimens (93.4%; p<0.0001), when compared with all other regimens, similarly adherence was lower for PI-based cART (89.3%; p<0.0001) and entry inhibitors-based cART (85.1%; p=0.001), while there was no difference between regimen with or without INI (90.2% vs. 91.5%; p=0.20). Adherence correlated with the virologic outcome, too. The mean adherence rate resulted of 91% in the K<3 and V<3 groups and lowered to 88% in the K>3 and V>50 groups (p<0.001). When insufficient (<90%) adherence was considered, a steady adherence level <90% was more frequently present in K>3 patients (26.3% of them), while sporadic drug holidays were typically observed in V>50 patients (17.9%; p<0.0001).

**Conclusions**: We demonstrated a relationship between virologic outcomes and adherence even when very low residual levels of HIV RNA (LLV) are considered. This fact is in favour of an active viral replication at least in some patients with LLV. However, the virologic outcome highly depends on forgiveness of modern ARV regimens as demonstrated by virologic responses in patients with insufficient adherence. Adherence is influenced by several demographic factors, but it is significantly linked to the choice of drugs, too. Although we observed a high standard of actual adherence, adherence rates could and should be improved.

# Sub article: P069 Developing a patient-reported outcome measure (PRO) for HIV care on perceived barriers to antiretroviral adherence: assessing the needs of HIV clinicians though typological analysis

**Published:** 2016-10-23

**Authors:** Isabelle Toupin, Kim Engler, David Lessard, Andràs Lènàrt, François Raffi, Bruno Spire, Bertrand Lebouché

**Affiliations:** 1 Department of Family Medicine, McGill University, Montreal, Canada; 2 Infectious Diseases, University Hospital, Nantes, France; 3 Université Aix-Marseille, SESSTIM, Marseille, France; 4 Chronic Viral Illness Service, Research Institute of the McGill University Health Centre (MUHC), Montreal, Canada

## Abstract

**Introduction**: Today, while many potent antiretroviral treatments (ART) and strategies are available, their clinical efficacy depends on patients adhering to them as prescribed. However, obstacles to adherence are common, multiple, recurrent and can be inadequately dealt with in clinical care. Tools are needed to address this apparent gap in HIV care. The use of a new PRO in HIV clinical care, based on patients’ perceived barriers to ART adherence, could prove helpful. In creating this PRO (I-Score Study/CTN 283), it is essential to take the needs of clinicians into consideration from the outset, given the crucial role these stakeholders play in their successful use in practise.

**Objective**: To identify HIV-specialized clinicians’ needs in regards to the clinical use of a new PRO which would be based on patients’ self-identified barriers to taking their ART.

**Materials and methods**: Five focus groups were conducted including 32 clinicians from across France. The focus groups were transcribed verbatim, coded vertically with Atlas.ti and, as the method was deemed appropriate, submitted to a typological analysis producing ideal types.

**Results**: The typological analysis identified seven patient profiles (ideal types), each tied to different barriers to adherence and indicating distinct needs for the PRO's content and data collection strategies. For the patient who: (1) is *passive*, the PRO must collect information on ART knowledge with closed questions and visual scales; (2) *never forgets a dose*, adherence must be verified with indirect questions; (3) *tolerates the intolerable*, questions must target problems experienced by patients with their ART; (4) *doesn't care, as long as they live life to the fullest*, interactive questions must be integrated on lifestyle and risk taking; (5) is *obsessive*, quality of life and life events must be assessed and space provided for textual responses for qualifying answers; (6) *must prioritize*, family/domestic life should be evaluated with Likert scales; (7) *lives precariously*, simple questions constructed with basic vocabulary and emoticons should be used to capture life circumstances and socio-economic context.

**Conclusions**: The clinicians’ needs for the new PRO were articulated in relation to different patient profiles, with multiple implications for the tool's content. The challenge will be to respond to both these needs and those that will be identified by patients in another component of the I-Score Study.

# Sub article: P070 Patient acceptance of a web-linked smartphone app to assess treatment compliance in HIV-infected subjects: a pilot study

**Published:** 2016-10-23

**Authors:** Ferran Sala-Piñol, Angels Andreu-Crespo, Josep Llibre, Josep Coll, Jordi Grasa, Angels Calvet, Bonaventura Clotet, Xavier Bonafont-Pujol

**Affiliations:** 1 Pharmacy, Hospital Universitari Germans Trias i Pujol, Badalona, Spain; 2 Lluita contra la SIDA, HIV Unit, Hospital Universitari Germans Trias i Pujol, Badalona, Spain; 3 Institutional Relations Department, Laboratorios Dr Esteve, Barcelona, Spain; 4 Informatics, Hospital Universitari Germans Trias i Pujol, Badalona, Spain

## Abstract

**Introduction**: Lack of adherence to ART increases therapeutic failure, resistance selection and health system expenditures through more expensive regimens. It is a public health problem, since uncontrolled viremia boosts transmission of both the infection and resistance. The study aim is to evaluate acceptance of a smartphone app, linked to a web, managed by the healthcare team (Figure 1).

**Materials and methods**: expertSalud^®^ is a validated free app including all Spanish registered drugs with predefined and free clinical controls and intake timetables. Patients confirm drug intakes on their smartphones. App links to a website where patients and healthcare practitioners track in real time their compliance without waiting for the next visit to detect adherence failures. The device maintains protection of confidential data. Patients were asked to report about all benefits and difficulties encountered. System usability was tested through two satisfaction surveys about combined app and web done at first and third months, using two different 18-question (scored 0–10) surveys.

**Results**: It is the first trial of a web-linked app used in real time by patients and their clinical team. From August 2015 to January 2016, hospital pharmacists recruited 81 smartphone users among subjects who started or changed ART, asking them to download and evaluate app. A total of 50 (62%) patients downloaded expertSalud^®^. Poor mobile phone coverage and troublesome registration hindered downloading. Before leaving the study, some patients noticed app dysfunctions. In total, 40 subjects answered at least one of the surveys and 21 both of them. The mean satisfaction score with the app in the first survey was 8.05/10, and 7.7/10 in the second one. For the web, the mean scores were 7.37 and 5.73, respectively (Figure 2). In both surveys, the app received the highest scores for allowing to include all their treatments (8.45–8.68) and selecting pill image and colour avoiding treatment confusion (8.30–8.33). Patients appreciated record easiness (8.06–7.89) and checking visualization (7.77–8.06), and they considered the app could be useful to improve their adherence (NA–7.90).

**Conclusions**: In this pilot study, patient's acceptance of an app to monitor ART intakes and adherence by the healthcare team was high. However, only half of the app users completed all the evaluation forms. App-linked web could be useful in detecting early treatment non-compliance and driving the implementation of targeted strategies. Suggestions received will improve system friendliness in the app development in new fully powered studies.

**Figure 1:** App screenshot.

**Abstract P070–Figure 2:** Survey results.

# Sub article: P071 The moderating role of treatment engagement on the relationship between neurocognitive impairment and antiretroviral treatment (ART) adherence

**Published:** 2016-10-23

**Authors:** Roman Shrestha, Pramila Karki, Tania Huedo-Medina, Michael Copenhaver

**Affiliations:** 1 Community Medicine and Health Care, University of Connecticut Health Center, Farmington, CT, USA; 2 Allied Health Sciences, University of Connecticut, Storrs, CT, USA

## Abstract

**Introduction**: Prior research has recognized neurocognitive impairment (NCI) and treatment engagement as important predictors of ART adherence [1–4]. No studies to date, however, have explored the possible ways and the extent to which a similar outcome can occur when these factors operate together, particularly among people who use drugs (PWUDs). This study sought to answer whether treatment engagement moderates the relationship between NCI and ART adherence.

**Methods**: A total of 116 HIV-positive opioid-dependent individuals enrolled in a methadone maintenance treatment (MMT) and reporting drug- and/or sex-related HIV risk behaviours were recruited from MMT clinic in New Haven, Connecticut. Participants completed an audio-computer-assisted self-interview (ACASI) that measured NCI (Brief Inventory of Neurocognitive Impairment), ART adherence (Visual Analogue Scale) and treatment engagement. An ordinary least squares regression-based path analytic framework was used to test whether treatment engagement (moderator) moderates the relationship between NCI (predictor) and ART adherence (outcome).

**Results**: Results showed that NCI (B=−0.745, p=0.004) was negatively associated with ART adherence. The interactive effect between NCI and treatment engagement was significantly associated with ART adherence (B=0.086, p=0.023), which supports the moderation effect. Post hoc analyses revealed that at low levels of treatment engagement, NCI was significantly negatively associated with ART adherence, while at high levels of treatment engagement, the relationship was non-significant.

**Conclusions**: The findings make an important contribution to our understanding of the applicability of a moderated model, such that NCI had an increased negative influence on ART adherence for individuals with lower treatment engagement. This highlights the need for future interventions to accommodate individuals’ NCI and improve treatment engagement in order to improve adherence to ART and thus health-related quality of life among opioid-dependent individuals living with HIV.

## References

[1] Meade CS, Conn NA, Skalski LM, Safren SA (2011). Neurocognitive impairment and medication adherence in HIV patients with and without cocaine dependence. J Behav Med.

[2] Malee K, Williams PL, Montepiedra G, Nichols S, Sirois PA, Storm D (2009). The role of cognitive functioning in medication adherence of children and adolescents with HIV infection. J Pediatr Psychol.

[3] Nicholas PK, Willard S, Thompson C, Dawson-Rose C, Corless IB, Wantland DJ (2014). Engagement with care, substance use, and adherence to therapy in HIV/AIDS. AIDS Res Treat.

[4] Farrell L, Ingersoll K, Ceperich SD (2009). Enhancing patient adherence: promoting engagement via positive patient-provider relationships in HIV/AIDS care. Med Encount.

# Sub article: P072 Real-world persistence and outcomes for HIV-1 treatment: single- versus multiple-tablet regimen comparison

**Published:** 2016-10-23

**Authors:** Donna Sweet, David Budd, Josh Cohen

**Affiliations:** 1 Department of Medicine, University of Kansas, Wichita, KS, USA; 2 Medical Affairs – Health Economics and Outcomes Re, Gilead Sciences, Foster City, CA, USA; 3 Tufts Center for the Study of Drug Development, Tufts University, Boston, MA, USA

## Abstract

**Introduction**: Advances in ART for HIV treatment have reduced patient morbidity and mortality [1]. Once-daily single-tablet regimens (STRs) may improve adherence and persistence by reducing pill burden compared to multi-tablet regimens (MTRs) [1,2. Persistence and adherence have been correlated with improved patient outcomes [3]. This retrospective study evaluated real-world persistence among HIV-1 infected patents comparing STRs versus MTRs using the Truven MarketScan^®^ database.

**Materials and methods**: Adults (≥18 years), diagnosed with HIV-1, with ≥1 prescription for ART during the index period (1 January 2011 through 31 December 2015) were identified. Patients were required to have continuous enrollment for 6-month baseline and follow-up periods until the end of enrollment or end of the study period, whichever came first. Primary outcome was index regimen persistence, defined as time from index regimen start date to end of first 90-day gap between fills for any ART in the index regimen, or to the start date of an ART not in index regimen. Kaplan–Meier and Cox proportional hazard models evaluated persistence and risk of discontinuation or switch across treatments controlling for age, gender, health-plan type, US region, Charlson comorbidity index (CCI) and baseline comorbidities.

**Results**: Index regimens are listed in Table 1. STRs were the index regimen for two-thirds of patients. A majority of patients were male (83%). Patients prescribed MTRs were older (mean age: 43.2 vs. 39.8 years, p<0.0001). MTR patients had higher rates of diabetes (7.0% vs. 5.7%, p=0.03), chronic kidney disease (2.4% vs. 1.1%, p<0.0001) and cardiovascular disease (25.6% vs. 22.2%, p=0.001). STRs demonstrated significantly greater persistence compared to MTRs. Controlling for baseline differences, MTRs were at twice the risk of discontinuation/switch (hazard ratio (HR) 1.95) compared to STRs. Median time to discontinuation/switch was 37.5 months for STRs, compared to 21.4 months among MTRs (p<0.0001). Among STRs, EVG/COB/FTC/TDF had a greater propensity for persistence compared to RPV/FTC/TDF (HR 1.14) and ABC/3TC/DTG (HR 1.24). Median time to switch for FTC/TDF + DTG was 22.1 months (p<0.0001). FTC/TDF + DTG had a significantly increased risk for discontinuation/switch compared to EVG/COB/FTC/TDF (HR 1.54), but a comparable risk compared to ABC/3TC/DTG (HR 0.98).

**Conclusions**: STRs improve persistence and reduce switching among HIV patients. EVG/COB/FTC/TDF had the highest persistency among STRs. STRs are likely to result in better patient outcomes compared to MTRs due to improved outcomes associated with persistence.

**Abstract P072–Table 1:** Index regimens

Single-tablet regimens (STRs)	n = 4156	61.3%
elvitegravir/cobicistat/emtricitabine/tenofovir disoproxil fumarate	EVG/COB/FTC/TDF	31.9%
rilpivirine/emtricitabine/tenofovir disoproxil fumarate	RPV/FTC/TDF	23.0%
abacavir/lamivudine/dolutegravir	ABC/3TC/DTG	6.4%
Multi-tablet regimens (MTRs)	n = 2622	38.7%
emtricitabine/tenofovir disoproxil fumarate + dolutegravir	FTC/TDF + DTG	3.8%
emtricitabine/tenofovir disoproxil fumarate + darunavir/ritonavir	FTC/TDF + DRV/r	16.8%
emtricitabine/tenofovir disoproxil fumarate + atazanavir/ritonavir	FTC/TDF + ATV/r	12.0%
abacavir/lamivudine + dolutegravir	ABC/3TC + DTG	1.9%
abacavir/lamivudine + darunavir/ritonavir	ABC/3TC + DRV/r	1.4%
abacavir/lamivudine + atazanavir/ritonavir	ABC/3TC + ATV/r	2.8%

## References

[1] Armstrong B, Chan DJ, Stewart MJ, Fagan D, Smith D (2015). Single tablet regimen usage and efficacy in the treatment of HIV infection in Australia. AIDS Res Treat.

[2] Sutton SS, Hardin JW, Bramley TJ, D'Souza AO, Bennett CL (2016). Single- versus multiple-tablet HIV regimens: adherence and hospitalization risks. Am J Manag Care.

[3] Cohen CJ, Meyers JL, Davis KL (2013). Association between daily antiretroviral pill burden and treatment adherence, hospitalisation risk, and other healthcare utilisation and costs in a US Medicaid population with HIV. BMJ Open.

# Sub article: P073 Factors influencing clinicians’ choices of ARVs in the US

**Published:** 2016-10-23

**Authors:** Rebecca Hahn, Bridgett Goodwin, Susan Hogue, Miranda Murray, Kimberly Davis

**Affiliations:** 1 Healthcare Marketing and Outcomes Research, Nielsen, Rochester, NY, USA; 2 Health Outcomes Research, GlaxoSmithKline, Research Triangle Park, NC, USA; 3 Health Economics Outcomes Research, RTI Health Solutions, Research Triangle Park, NC, USA; 4 Global Health Outcomes, ViiV Healthcare, Brentford, UK

## Abstract

**Introduction**: While the majority of the published literature has focussed on the role of patients’ attitude regarding adherence, the healthcare provider (HCP) has a significant role in patient adherence with ART. This is a great concern to HCPs as a great level of adherence is needed for most patients to achieve full and sustained viral suppression. This study was designed to better understand the factors that impact clinicians’ prescribing patterns for ART that will optimize patient adherence to HIV therapy in the US.

**Methods**: In mid-2014, two cross-sectional internet-based surveys were conducted in the US with 400 patients prescribed with an ARV and with 200 physicians who treat HIV. The 30-minute online surveys were pretested with a small group of respondents (n=5 patients and n=5 physicians). The patient survey included HIV treatment experience, medication side effects, adherence behaviours, treatment satisfaction and interaction with HCPs. The physician survey addressed similar topics to allow for direct comparisons between HIV patients and physicians.

**Results**: The patient sample were primarily males (79.3%) with an average age of 41.1 (SD 13.2) years with 61% homosexual, 28.1% heterosexual and 11.1% bisexual. A total of 59% of patients were diagnosed with comorbidities, the most common being depression, hypertension and hyperlipidemia. The physician sample included 119 primary care physicians (PCPs) and 81 infectious disease (ID) specialists. The factors most important for IDs and PCPs in ARV prescribing, were virologic control, followed by adherence and side effects. PCPs were more concerned with affordability/insurance coverage than IDs. Moreover, there were significant differences between the choices of ARV for patients with comorbidities, for IDs compared to PCPs. These included kidney and liver diseases; hyperlipidemia and diabetes most affected HCPs’ treatment decisions (Figure 1).

**Conclusion**: Adherence challenges persist for both IDs and PCPs in prescribing ARVs. Understanding the optimal prescribing patterns for ARVs and ensuring that HCPs consider both the polypharmacy related to both HIV as well as comorbid conditions is important, especially as HIV is being treated as a chronic disease.

**Abstract P073–Figure 1:** Differentiating factors influencing HCPs’ choices of ARVs.

# Sub article: P075 MATH study: migrants adherence to therapy at an HIV outpatient clinic

**Published:** 2016-10-23

**Authors:** Catarina Rodrigues, Yohana Martins, Umbelina Caixas, Fatima Lampreia, Isabel Germano

**Affiliations:** 1 Serviço Medicina 1.4, Hospital São José, Lisbon, Portugal

## Abstract

**Introduction**: Strict adherence to ART is essential for HIV suppression – to reduce the risk of treatment resistance, improve health and quality of life of those infected, reduce mortality and minimize risk of transmission. By contrast, poor adherence to therapy is a major cause of treatment failure. It is essential to identify factors for poor adherence in order to a customized action. The aim of this study is to determine adherence differences to HIV therapy between the migrant and the Portuguese population on follow-up in our HIV outpatient clinic.

**Material and methods**: Cross-sectional, descriptive study including 719 patients followed in a Portuguese HIV outpatient clinic since the year 2000. Of these, 651 patients met the inclusion criteria (over 18 years old, on follow-up since 2000, with one or more appointments in 2015); 428 Portuguese patients and 223 migrants – 143 from Africa (mostly Angola, Cap Verde and Guinea), 58 from South America (almost all from Brazil), 13 from Europe and 2 from Asia. Adherence was defined by undetectable viral load in the last two evaluations. Results between the two groups were compared using Chi-squared test.

**Results**: Total of 651 patients, mean age of 44.8 years (Portuguese 45.6 vs. migrants 43.2), 450 males and 201 females (Portuguese 315 males/113 females vs. migrants 135 males/88 females). Non-adherence in the Portuguese group was present in 28 patients (6.5%; n=428) and in the migrants group in 13 patients (5.8%; n=223). The reasons for non-adherence in the Portuguese group were mostly alcohol and drug abuse or depression and other mental disorders; while in the migrant group were low level of health education and low social support. We saw that usually the regime prescribed did not influence adherence – number of doses/tablets, adverse effects or relation with/without meal.

**Conclusions**: Even though the adherence in the Portuguese group was lower in percentage than in the migrant group, the reasons for this suboptimal adherence might improve with interventions in our outpatient clinic, such as better social support and reinforcement of health education with enhanced risk perception and better knowledge of this disease.

# Sub article: P076 Predictors of retention to care among HIV-infected patients in Northern Greece

**Published:** 2016-10-23

**Authors:** Olga Tsachouridou, Eirini Christaki, Lemonia Skoura, Aadia Rana, Pantelis Zempekakis, Symeon Metallidis

**Affiliations:** 1 1st Internal Medicine Department, AHEPA University Hospital, Medical School, Aristotle University of Thessaloniki, Thessaloniki, Greece; 2 Microbiology Department, AHEPA University Hospital, Medical School, Aristotle University of Thessaloniki, Thessaloniki, Greece; 3 Department of Medicine, Alpert Medical School of Brown University, Providence, RI, USA

## Abstract

**Introduction**: Retention to care is vital in order to improve HIV outcomes. Data regarding retention to care of HIV-positive individuals in Greece are scarce. The objective of this study was to assess retention to care and identify factors associated with incomplete retention in a longitudinal cohort of HIV-infected patients in Northern Greece.

**Materials and methods**: We conducted a retrospective cohort study in Thessaloniki, Northern Greece of 1450 newly diagnosed HIV patients >18 years old who entered care from 1990 to 2015 and followed until present. Retention to care was defined as having at least one visit each year of care throughout the entire follow-up period. Also, we studied predictors of gaps in care and the analysis was divided into three distinct time periods. A secondary analysis was done to determine the relationship between demographic and clinical variables and the number of years out of care.

**Results**: Of the 1450 patients included, 38.41% had at least one gap in care during the study period. Patients with complete retention to care were older (37.10±10.71 vs. 35.85±11.00), fully insured (71.8% vs. 53.1%, p<0.005), more likely to be registered to care between 2010 and 2015 (48.5% vs. 23.5%, p<0.005), to have higher educational level (30.5% vs. 25.1%, p=0.029) and were more likely to receive HAART (81.9% vs. 71.8%, p<0.005) and have viral suppression (91% vs. 80%, p<0.005), than those displaying gaps in care. In the adjusted analysis, older age, Greek origin, full insurance, HAART intake and viral suppression were all associated with increased likelihood of retention. Clinic registration between 1990 and 2000 (p=0.044) and 2000 and 2010 (p<0.005) were also predicting factors of retention to care. Ten-year survival between non-retained (63%) and retained patients (92%) was statistically significant (p<0.005). Retention was associated with decreased likelihood of death (aHR 0.37, 95% CI 0.26–0.52; p<0.005). Patients with high education level and Greek origin presented less years out of care (p=0.002 and p<0.005, respectively). Patients with one or more hospital admissions had more years out care compared with those no hospital admission (p<0.05).

**Conclusions**: Foreign origin, lack of insurance and type of transmission other than MSM are predictors of incomplete retention to care. Retention to care is associated with better HIV infection outcomes and survival. Our results suggest that one-third of newly diagnosed HIV-infected patients will experience at least one gap in care. Interventions should focus on prevention of gaps and maintenance of continuous follow-up of HIV patients.

# Sub article: P077 Treatment of HIV and acute myeloid leukaemia by allogeneic CCR5-d32 blood stem cell transplantation

**Published:** 2016-10-23

**Authors:** Elena Knops, Guido Kobbe, Rolf Kaiser, Nadine Luebke, Gabor Dunay, Annemarie Wensing, Javier Martinez-Picado, Monique Nijhuis, Johannes Fischer, Falk Huettig, Rainer Haas, Dieter Haeussinger, Bjoern Jensen

**Affiliations:** 1 Institute of Virology, University of Cologne, Cologne, Germany; 2 Department of Hematology, Oncology and Clinical Immunology, University of Duesseldorf, Duesseldorf, Germany; 3 Institute for Virology, University of Duesseldorf, Duesseldorf, Germany; 4 Leibniz Institute for Experimental Virology, Heinrich Pette Institute, Hamburg, Germany; 5 University Medical Center, University of Utrecht, Utrecht, Netherlands; 6 AIDS Research Institute IrsiCaixa, Hospital Germans Trias i Pujol, Barcelona, Spain; 7 Institute of Transplantation Diagnostics and Cell, University of Duesseldorf, Duesseldorf, Germany; 8 Department of Gastroenterology, Hepatology and Infectious Diseases, University of Duesseldorf, Duesseldorf, Germany

## Abstract

The Berlin patient is presumed to be the only person cured from HIV infection by hematopoietic stem cell transplantation (HSCT) from a homozygous CCR5-d32 donor. Attempts to reproduce cure by HSCT have failed because of either viral rebound or death due to the underlying malignancy. We here report a patient alive, well and negative for proviral DNA 900 days after HSCT. A 41-year-old HIV-infected male patient was diagnosed acute myeloid leukaemia (AML, inv16, CBF-MYH11) in January 2011. Since the diagnosis of HIV infection in October 2010, he had been treated with TDF/FTC+DRV (January 2011 VL 44 copies/mL; CD4+ 474 cells/µL). To avoid interactions with chemotherapy DRV was switched to RAL in March 2011. He achieved CR of the AML after one induction course (ICE) and received a second induction and three consolidation courses according to AML-SG 07/04. In September 2012, AML relapsed and he was treated with A-HAM and a second cycle high-dose cytarabine. While in second CR, he received unmodified peripheral blood stem cells from a female 10/10 CCR5-d32 DKMS donor after conditioning with fludarabine/treosulfan in February 2013. Before transplant, HIV resistance analysis was performed and viral tropism was determined. There were no significant resistance mutations, and the coreceptor usage was predicted as R5-tropic (Sanger sequencing: FPR 44.5%; NGS: 0.14% X4 at 3.5% FPR; geno2pheno). The proviral DNA load was 1.45 log_10_ copies/106 PBMCs, and in the western blot, all anticipated bands could be detected. During transplant and until today, the patient remained on ART (since June 2014 ABC/3TC/DTG), and the viral load remained undetectable in plasma and liquor. He had a second relapse of AML in June 2013 but re-entered molecular remission after a total of eight courses of 5-azacytidine and four donor lymphocyte infusions. Concerning HIV, all collected samples were negative for proviral DNA by conventional and digital droplet PCR in two different labs, namely PBMCs (2014–2016), rectal biopsy (April 2015) and bone marrow (August 2015). Western blots from 2014, 2015 and 2016 showed incomplete patterns with fading bands. Viral outgrowth assays are in progress. Like in the Berlin patient, all tests from the Duesseldorf patient so far suggest that HIV may have been eradicated and that he may be the second individual cured from HIV by allogeneic CCR5-d32 HSCT. Further investigations will be performed before considering the discontinuation of ART.

# Sub article: P078 Differential efficacy of ABX464 and its primary metabolite ABX464-NGlc on HIV replication in human PBMCs and macrophages: implications for treatment strategies to eliminate virus reservoirs

**Published:** 2016-10-23

**Authors:** Didier Scherrer, Jean-Marc Steens, Paul Gineste, Noelie Campos, Aude Garcel, Erika Schlaefper, Roberto Speck, Jamal Tazi, Hartmut Ehrlich

**Affiliations:** 1 ABIVAX, Paris, France; 2 Division of Infectious Diseases, University Hospital of Zurich, Zurich, Switzerland; 3 Institute of Molecular Genetics of Montpellier, Centre National de la Recherche Scientifique, Montpellier, France

## Abstract

**Introduction**: We developed a first-in-class small molecule (ABX464) with a novel mechanism of anti-HIV involving modulation of viral RNA splicing [1]. ABX464 was demonstrated to be effective in inhibiting HIV replication *in vitro*, *in vivo* and in HIV patients [2]. Studies in humanized mice infected with HIV demonstrated that ABX464 monotherapy had an antiviral effect, which was sustained after treatment interruption. Therefore, ABX464 may have an effect on virus reservoirs. In humans, ABX464 is metabolized to one main metabolite, ABX464-NGlc. We investigated the differential effects of parent compound and metabolite on virus replication *in vitro* in both stimulated PBMCs and macrophages to investigate potential antiviral effects in macrophages, the cell population considered to be the key virus reservoir.

**Materials and methods**: Human PBMCs and monocytes were isolated from healthy donors. Cultured cells were treated with ABX464 or ABX464-NGlc and then infected with virus. Following 6–12 days of incubation, HIV p24 titration was performed on supernatants by ELISA with Ingen Innotest kit.

**Results**: Dose-dependent inhibition of HIV-1 replication by ABX464 was demonstrated in stimulated PBMCs with an IC50 ranging between 0.1 and 0.5 µM, while the metabolite did not show any efficacy in inhibiting virus replication in human PBMC *in vitro*. By contrast, although the metabolite demonstrated no antiviral effect on PBMCs, it blocked virus replication in primary macrophages reaching inhibition levels of up to 90% at 0.1 µM.

**Conclusions**: These findings have substantial implications for targeting the HIV reservoir with ABX464. Studies in healthy subjects demonstrated ABX464-NGlc's C_max_ was about 160-fold higher than those of ABX464 and had a much longer t_1/2_ (90–110 hours) than the parent compound (2–3 hours), resulting in a >1000-fold difference in AUC between the two compounds [3]. The markedly higher plasma concentrations of ABX464-NGlc, and its ability to inhibit viral replication in infected macrophage cultures with the same IC50 as the parent drug, may allow effective targeting of the reservoir in patients whose viral load is fully controlled by existing ARTs. In this case, the primary aim of the therapeutic intervention with ABX464 is to delay/prevent the viral rebound typically originating from the reservoir. This concept is being explored in an ongoing clinical trial, in which patients receive ABX464 for 4 weeks in combination with standard ART, with subsequent cessation of all treatments and intense viral load monitoring until viral rebound.

## References

[1] Campos N, Myburgh R, Garcel A, Vautrin A, Lapasset L, Nadal ES (2015). Long lasting control of viral rebound with a new drug ABX464 targeting Rev – mediated viral RNA biogenesis. Retrovirology.

[2] Scherrer D, Steens J-M, Kuanchai S, Winai R, Ruxrungtham K, Rouzier R Early evidence of antiviral activity and safety of ABX464 in HIV treatment-naïve patients.

[3] Scherrer D, Rouzier R, Barrett PN, Steens J-M, Gineste P, Murphy R Pharmacokinetics and tolerability of ABX464, a novel first in class compound to treat HIV infection, in healthy HIV-uninfected subjects. under review.

# Sub article: P079 Development of methods for *in vitro* reactivation of latent reservoir of HIV-1 infection in patients under suppressive HAART

**Published:** 2016-10-23

**Authors:** Gnaneshwer Jadav

**Affiliations:** 1 Medical Biotechnologies, University of Siena, Siena, Italy

## Abstract

**Introduction**: Latent reservoir of HIV-1 infection has a relevant impact in the management of the treatment and in the evaluation of the eradication strategies approaches. We have developed *in vitro* models by using HDAC inhibitors and PKC agonists to reactivate the transcription of proviral HIV DNA in cell culture using lymphocytes collected from HIV-1 infected patients under suppressive ART. The aims of our study were to evaluate (1) the size of the viral particles produced by lymphocytes by using a specific qPCR method and (2) the comparison of the sequences of viral regions involved in drug resistance obtained from both newly produced viral populations and proviral DNA.

**Materials and methods**: Lymphocytes from HIV-1 infected patients under successful antiretroviral treatment were harvested from whole blood and both stored as the baseline sample and resuspended in the appropriate growth medium for the evaluation of HIV RNA production by using reactivating agents PHA or PMA alone or in combination with vorinostat (VOR). We calculated the amount of genomic viral RNA in the cell culture supernatants by a specific qRT-PCR system targeting the 3' poly-adenylated tail of HIV RNA genome. We compared the sequences of the regions involved in drug resistance by the partial amplification of POL and ENV gene from both baseline HIV DNA and from supernatant viral RNA of successfully induced lymphocytes.

**Results**: We have showed that PKC agonists such as PMA or PHA, when combined with HDAC inhibitors such as VOR, effectively induced viral transcription in lymphocytes from patients on successful ART at higher levels than those obtained stimulating lymphocytes with PHA or PMA alone. From the data observed in the comparison of POL sequences between baseline HIV DNA and supernatant viral RNA, we found that 7/13 patients showed similar genotypes, among which 6 had no mutation and 1 case showed the same resistance mutations in both DNA and RNA. Interestingly, 4/13 patients had mutations detected in reactivated viral RNA but not found in the baseline proviral HIV DNA. Similarly, the comparison of the sequences of the V3 domain of the ENV showed that 2/6 patients had a different predicted coreceptor usage between proviral HIV DNA and reactivated viral RNA.

**Conclusions**: Latent HIV-1 infection of lymphocytes remains the major barrier to HIV-1 eradication and our data confirmed that could have a relevant impact in the management of HIV infection treatment as well.

# Sub article: P080 Dual regimen with dolutegravir and lamivudine maintains virologic suppression even in heavily treatment-experienced HIV-infected patients: 48-week results from a pilot study (DOLULAM)

**Published:** 2016-10-23

**Authors:** Jacques Reynes, Nadia Meftah, Brigitte Montes

**Affiliations:** 1 Infectious Diseases, Montpellier University Hospital, Montpellier, France; 2 COREVIH, Montpellier University Hospital, Montpellier, France; 3 Virology, Montpellier University Hospital, Montpellier, France

## Abstract

**Introduction**: Dolutegravir (DTG) is an HIV integrase inhibitor with a potent antiviral activity and high genetic barrier. However, few viral rebounds with emergence of integrase resistance mutations during DTG monotherapy maintenance were observed [1]. M184I/V mutations against lamivudine (3TC) could prevent the emergence of resistance mutations against DTG [2].

**Methods**: DOLULAM is a prospective cohort study. Patients on a stable antiretroviral regimen with HIV RNA <50 copies/mL for >12 months, with problems of tolerability and without resistance to integrase inhibitors, were given the opportunity to switch to dolutegravir 50 mg plus lamivudine 300 mg once daily. Visits and laboratory tests including plasma HIV-1 RNA levels (VL)(Roche Cobas Ampliprep/Taqman HIV-1 version 2.0; limit of detection, 20 copies/mL) were scheduled at baseline (BL), 6, 12, 24 (36 optional) and 48 weeks.

**Results**: We enrolled 27 patients from October 2014 to April 2015 (20 men, 7 women; median age: 59 years; plasma HIV-1 RNA zenith >100,000 copies/mL: 56%; median nadir CD4: 167/mm^3^; median baseline CD4: 601/mm^3^). Before switching to dual therapy, patients had been taking antiretroviral therapy for a median of 215 (range 22–329) months and the last HAART (TDF: 48%; PI/r: 81%; RAL: 26%) for a median of 51 (13–108) months. Ten (37%) patients had a history of genotypic test prior switch with M184V mutation. During the first 48 weeks of follow-up, no patient experienced virologic failure (defined as confirmed VL >50 copies/mL) or severe clinical or laboratory adverse event, or was lost to follow-up. Three patients experienced one blip (52, 31 and 66 copies/mL at week 12, 24 and 36). Three patients wanted to discontinue DTG+3TC combination (two at week 16 and week 24 for fatigue, one at week 18 after blip at week 12 visit). There was a small decrease from baseline in estimated glomerular filtration rate (eGFR) at first on-treatment assessment (median week 6 evolution: −9 mL/min/1.73 m^2^) but remained stable over the 48-week follow-up (−7).

**Conclusions**: These results suggest that, in this population of heavily treatment-experienced patients without or with history of M184I/V mutation, dolutegravir plus lamivudine dual therapy is an attractive strategy of maintenance.

## References

[1] Katlama C, Soulié C, Caby F, Denis A, Blanc C, Schneider L (2016). Dolutegravir as monotherapy in HIV-1-infected individuals with suppressed HIV viraemia. J Antimicrob Chemother.

[2] Oliveira M, Ibanescu RI, Pham HT, Brenner B, Mesplède T, Wainberg MA (2016). The M184I/v and K65R nucleoside resistance mutations in HIV-1 prevent the emergence of resistance mutations against dolutegravir. AIDS.

# Sub article: P081 Efficacy of antiretroviral drugs during intermittent maintenance treatment with a 4-days-a-week regimen despite low plasma concentrations (ANRS 162-4D trial)

**Published:** 2016-10-23

**Authors:** Jean Claude Alvarez, Pierre De Truchis, Emuri Abe, Lambert Assoumou, Roland Landman, Dominique Mathez, Karine Amat, Christine Katlama, Pierre Marie Girard, Damien Le Du, Martin Duracinsky, Dominique Costagliola, Jonathan Bellet, Christian Perronne

**Affiliations:** 1 Pharmacology, AP-HP and Universit de Versailles Saint-Quentin en Yvelines, Garches, France; 2 Infectious Disease, AP-HP and Universit de Versailles Saint-Quentin en Yvelines, Garches, France; 3 INSERM, Institut Pierre Louis d'Epidmiologie et de Sant Publique, UPMC Paris 6 University, Paris, France; 4 APHP CHU Bichat, IMEA, Paris, France; 5 APHP CHU R Poincare, Virology, Garches, France; 6 APHP Hopital Pitie-Salpetriere, Universite Paris 6, Infectious Diseases, Paris, France; 7 APHP Hopital St Antoine, Universite Paris 6, Paris, France; 8 APHP CHU R Poincare, Infectious disease, Garches, France; 9 APHP CHU Bicetre, Paris, France

## Abstract

**Introduction**: In the absence of virologic eradication despite combination of antiretroviral therapy, patients infected by HIV have to take the treatment throughout their life. Intermittent regimen could be an alternative for those as evocated in previous studies (FOTO, BREATHER).

**Material and methods**: An open-label, multicentre, single-arm prospective study had been conducted to evaluate the capacity of a weekly strategy of 4 consecutive days treatment (“period on") followed by 3 days without treatment (“period off"), in HIV-1 patients with undetectable viral load for at least 12 months. Patients had no treatment modification in the past 6 months, and were on two nucleosides analogues and either boosted protease inhibitor (PI/r) or a NNRTI. Plasma antiretroviral residual concentrations on “period on" collected at day 0, week 16, week 40 and on “period off" collected at week 4, week 8, week 12, week 32 and week 48 were measured using a validated turbulent flow chromatography method coupled to triple quadrupole mass spectrometry detection with electrospray ionization interface. The laps between the last medication intake and the sample collecting time over 48 hours were considered as off period samples.

**Results**: Among the 100 patients included, 12 drug combinations were used: TDF+FTC (n=89), ABC+3TC (10), ABC+TDF (1) combined with a PI/r for 29 (DRV/r: 15; ATV/r: 13; LPV/r: 1) or a NNRTI for 71 (EFV: 40; RPV: 26; ETV: 5). After 48 weeks, 96% (95% CI 90-98, Kaplan-Meier estimate) were still under intermittent 4/7 days regimen without failure. In total, 877 samples were analyzed (292 “on” and 585 “off”). A total of 94.4% of plasma samples are consistent with the timing. Significant differences had been observed between “on” concentrations and “off” concentrations for DRV (2587±1393 ng/mL vs. 17±18 ng/mL, p<0.0001), ATV (1087±644 ng/mL vs. 52±146 ng/mL, p=0.0005), LPV (39±22 ng/mL vs. <20 ng/mL), EFV (2218±1046 ng/mL vs. 692±391 ng/mL, p<0.0001) and RPV (106±51 ng/mL vs. 3920 ng/mL, p<0.0001). Many PI were undetectable on “off” period. For ETV (447±360 ng/mL vs. 269±266 ng/mL, p=0.0625) only a tendency was observed, probably because of low number of patients in this group. All “on” concentrations were in accordance with French guidelines.

**Conclusion**: A total of 96% of patients maintained viral load undetectable despite low or undetectable plasma concentrations after 3 days of treatment interruption on a 4-days-a-week regimen. PI plasma samples were almost always undetectable since NNRTI ones were decreased but above the limit of quantification according to their longer half-lives.

# Sub article: P082 Dual therapy with non-boosted atazanavir plus lamivudine is an effective simplification strategy for virologically stable patients with HIV

**Published:** 2016-10-23

**Authors:** Juan Pasquau, Samantha Elizabeth de Jesús, Coral García-Vallecillos, Carmen Hidalgo-Tenorio

**Affiliations:** 1 Infectious Diseases, Hospital Universitario Virgen de las Nieves – Complejo Hospitalario de Granada, Granada, Spain

## Abstract

**Introduction**: The main objective of this simplification therapy is the reduction of potential toxicity of long-term antiretroviral treatment. In this regard, the withdrawal of ritonavir has demonstrated advantages in terms of tolerance and metabolic toxicity. There are previous studies that establish that non-boosted ATV_400_ has similar efficacy and a better toxicity profile than ritonavir-boosted ATV (ATV/r). But we have scarce information on the efficacy and safety of the dual regimen with lamivudine (3TC)+ATV_400_.

**Methods**: This is a retrospective, single-centre, observational study in which we analyzed the evolution of our virologically stable patients who received a triple therapy or a dual therapy with 3TC+ATV/r and switched to a dual therapy with 3TC+ATV_400_ as a simplification strategy.

**Results**: A total of 46 patients received the non-boosted ATV_400_ plus 3TC combination. They had previously taken antiretroviral treatment for an average of 12.1 years and four previous treatment combinations, a mean CD4 nadir of 229 cells/mm^3^ and a baseline viral load of 95,004 copies/mL. A total of 17.4% of patients were co-infected with HCV. In all patients, the viral load had been suppressed for over 6 months and they had tolerated their previous treatment well, which in 35/46 cases was a dual therapy with ritonavir. After an observation period of 44.6 patient-years, only one patient discontinued the study combination, due to virologic failure. During the study, there were no adverse events and 157 viral loads were determined. Of these, 94.9% were <50 copies/mL and 86.6% were <20 copies/mL (61.3% were completely undetectable). A total of 67.4% of patients maintained a viral load <20 copies/mL during the whole study, and 84.8% achieved a completely undetectable viral load at some point during the study. There were eight viral loads >50 copies/mL and five of them were blips. Only one patient had virologic failure (2.2%), with two consecutive detectable viral loads (1132 and 3558 copies/mL), associated with resistance to ATV (protease mutations: 10F, 20T and 50L). This virologic failure was associated with confirmed poor treatment adherence.

**Conclusions**: Our data, and those of other studies, suggest that the 3TC+ATV_400_ dual combination, as a simplification strategy in stable patients, suppresses HIV replication and is non-inferior to triple therapy. We believe this attractive strategy can be explored safely and should be confirmed with further studies.

# Sub article: P083 Lamivudine+dolutegravir as simplification strategy in patients with suppressed HIV RNA

**Published:** 2016-10-23

**Authors:** Franco Maggiolo, Roberto Gulminetti, Cristina Mussini, Layla Pagnucco, Margherita Digaetano, Simone Benatti, Daniela Valenti, Diego Ripamonti

**Affiliations:** 1 Infectious Diseases, ASST Papa Giovanni XXIII, Bergamo, Italy; 2 Infectious Diseases, Fondazione IRCCS Policlinico San Matteo, Pavia, Italy; 3 Infectious Diseases, University of Modena, Modena, Italy

## Abstract

**Introduction**: cART is generally based on a backbone consisting of two NRTIs and on a third agent of a different class. The availability of new potent drugs raises the opportunity to change the classical NRTI-backbone paradigm and to explore less-drug regimens able to overcome adverse events due to commonly used NRTIs.

**Materials and methods**: This is a prospective, multi-centre, proof-of-concept, cohort study in patients on stable cART, with a confirmed (>6 months) viremia <50 copies/mL, absence of M184V mutation or HBsAg and with intolerance/contra-indications to current cART. Patients were switched to a dual 3TC+DTG regimen and prospectively monitored. ITT analysis results are reported.

**Results**: A total of 94 patients, mostly males (76.5%) and with a mean age of 53 years (SD 11) were enrolled. The most common risk factors for HIV infection were heterosexual (54.3%) and homosexual (22.3%) relationships. At switch, patients were on ARV drugs for a mean of 11.3 years (SD 6.8). They had been on the ongoing therapy for a mean of 50.2 months (SD 40.1) and virologically suppressed for a mean of 88.8 (SD 74.4) months. They had experienced a mean of 4.1 lines of therapy (range 1–10) and were currently treated with a variety of drugs: 91.5% were assuming a NRTI (TDF 51.1% and ABC 37.2%); 55.3% were on a NNRTI-based regimen, 30.1% were assuming PIs and 17.0% an integrase inhibitor. At switch, all of them presented an HIV RNA <50 copies/mL and a CD4 mean count of 742 cells/µL (SD 353), but 91.5% of them presented comorbidities mainly involving the cardiovascular system (35.1%), the bone (33.0%), the liver (23.4%), the nervous system (21.3%), the kidney (9.6%), the metabolic status (17.0%) or the glucose homeostasis (6.4%). During the 6 months of FU, no patient stopped therapy nor we observed any virologic failure. CD4 mean increment was of 61 cells/µL (p=0.018) without significant changes of CD8 cells or CD4/CD8 ratio. Total (−5 mg/dL) HDL and LDL cholesterol were stable, while triglycerides slightly decreased (−18 mg/dL, p=0.025). Blood creatinine increased from 0.92 to 1.00 mg/dL (p<0.0001). Finally, the dual switch therapy reduced the mean daily cost of therapy of 7.00 Euros.

**Conclusion**: A dual 3TC+DTG regimen is a feasible alternative in virologically controlled patients that may overcome the limits of a classical NRTI backbone. This alternative regimen is cost-effective. Our results indicate the opportunity of a larger controlled trial.

# Sub article: P084 In monotherapy, darunavir/cobicistat demonstrates equivalence to darunavir/ritonavir, and in selected patients is as effective as bitherapies or triple therapies

**Published:** 2016-10-23

**Authors:** Juan Pasquau, Coral García-Vallecillos, María Teresa Cruces-Moreno, Samantha Elizabeth de Jesus, Georgette Fatoul del Pino, Carmen Hidalgo-Tenorio, David Vinuesa-García, Miguel Ángel López-Ruz, José Hernández-Quero

**Affiliations:** 1 Infectious Diseases, Complejo Hospitalario Universitario de Granada, Granada, Spain

## Abstract

**Introduction**: Various studies have demonstrated the bioequivalence of darunavir/ritonavir (DRV/r) and darunavir/cobicistat (DRV/c). However, there are doubts about how the lower plasma trough concentrations achieved with DRV/c may affect therapeutic efficacy. Triple therapy is not a good model to test the equivalence of only one component of the treatment, but monotherapy is. It is for this reason that the objective for this study was to analyze the virologic efficacy achieved with DRV/c when used in monotherapy.

**Materials and methods**: This is an observational, retrospective, single-centre analysis of all patients in our hospital who received DRV/c in monotherapy. We analyzed the evolution of HIV viremia, and we have compared these results with those achieved previously with DRV/r and lopinavir/ritonavir (LPV/r) in our historic controls. In addition, we compared these results to those obtained in a recent three-way comparison (monotherapy vs. bitherapy vs. triple therapy) of contemporary patients in our centre.

**Results**: Since July 2015 to May 2016, 181 patients have received DRV/c in monotherapy (94.5% from monotherapy with DRV/r (93.6%) or LPV/r (6.4%)). Only four patients discontinued DRV/c: one due to virologic failure and three due to mild intolerance. The global exposition time for this cohort was 58.9 patient-years. During this time, 196 plasma viral loads (VL) have been determined: 91.9% (180/196) were <50 copies/mL (62.8% (123/196) were undetectable, 20.4% (40/196) were detectable but below the level of detection (<20 copies/mL) and 8.7% (17/196) were between 20 and 50 copies/mL), 6.1% (12/196) were between 50 and 200 and 2% (4/196) were >200 copies/mL. In a previous analysis of our patients (n=185) who were receiving monotherapy with DRV/r or LPV/r (2005–2013), 1003 VL have been determined: 84.1% were <50 copies/mL, 10.7% between 50 and 200, and 5.2% >200 copies/mL. Moreover, in another comparative study of parallel viral loads recorded during the same period of time (from March 2014 to April 2015) between the different strategies (monotherapy vs. bitherapy vs. triple therapy, for >6 months), our patients on monotherapy (n=49, with DRV/r or LPV/r) presented a VL <50 copies/mL in 93.4% of cases, compared with 90.8% with dual therapies (n=79) and 82.3% with triple therapies (n=93) (Table 1).

**Conclusions**: In real life, DRV/c presents an equivalent virologic efficacy to DRV/r and LPV/r. In addition, in selected patients who it is prescribed to and maintained in monotherapy, the stratified analysis of the viremias is similar to that of dual therapy and triple therapy.

**Abstract P084–Table 1:** Stratified viral load distribution between different ARV combinations

Antiretroviral combination	N patients	N VL tests	%VL undetectable	%VL detectable <20	%VL 20–50	%VL <50	%VL 50–200	%VL >200
DRV/cobicistat^a^	181	196	62.8	20.4	8.7	91.9	6.1	2
DRV/r or LPV/r^b^	185	1003	–	–	–	84.1	10.7	5.2
Monotherapy^c^	49	105	55.7	29.2	8.5	93.4	2.8	3.8
Dual therapy^c^	79	167	56.9	25.1	8.4	90.8	4.2	5.4
Triple therapy^c^	93	193	51.6	22.9	7.8	82.3	6.2	11.5

VL, HIV plasma viral load (copies/mL).

a Current analysis (monotherapy with DRV/c between July 2015 and May 2016)

b historical data of our patients on monotherapy (2005–2013)

c comparative study of our patients (between March 2014 and April 2015).

# Sub article: P085 Darunavir/cobicistat as antiretroviral treatment simplification strategy in patients with stable monotherapy with ritonavir-boosted protease inhibitors in the clinical setting

**Published:** 2016-10-23

**Authors:** José Ramón Santos, Pablo Peláez Ibañez, Isabel Bravo, Josep Maria Llibre, Roger Paredes, Bonaventura Clotet, José Moltó

**Affiliations:** 1 HIV Unit, Lluita contra la SIDA Foundation, Hospital Germans Trias i Pujol, Badalona, Spain; 2 Medicine School, Universitat Autònoma de Barcelona, Barcelona, Spain; 3 HIV Unit, Lluita contra la SIDA Foundation, IrsiCaixa Foundation, Hospital Germans Trias i Pujol, Badalona, Spain

## Abstract

Antiretroviral treatment with darunavir/ritonavir monotherapy has been extensively used as simplification NRTI-sparing strategy in the last years. Recently, co-formulated darunavir/cobicistat has become available. In comparison with ritonavir, cobicistat has no intrinsic antiretroviral activity, it is more selective than ritonavir in terms of inhibition of different isoenzymes of the cytochrome P450 system, and it does not induce CYP isoenzymes or glucuronidation. In a clinical trial, exposure to darunavir following darunavir/ritonavir and darunavir/cobicistat administration was not significantly different, although darunavir trough concentrations were 30% lower with cobicistat than with ritonavir [1]. This difference seems not to be clinically relevant in patients on triple therapy and without resistance mutations associated to darunavir. However, there are not clinical trials evaluating the effectiveness and safety of darunavir/cobicistat as monotherapy simplification strategy. We retrospectively studied all HIV-1-infected subjects treated with darunavir/ritonavir or lopinavir/ritonavir monotherapy who were switched to darunavir/cobicistat monotherapy while having plasma VL <50 copies/mL, and had at least one subsequent follow-up visit in our clinic. The primary endpoint was the percentage of patients who maintained virologic suppression (HIV-1 VL <50 copies/mL) after switching. Virologic failure was defined as a VL >50 copies/mL in two consecutive determinations or as any change in the monotherapy regimen after a single determination with a pVL >50 copies/mL. We also evaluated other reasons for treatment discontinuation. Analyses were performed considering all regimens (full data set analysis) as “treatment switch equals failure.” A total of 124 subjects on stable PI monotherapy during a median time of 380 (IQR 345–425) weeks were switched to darunavir/cobicistat, 121 (97.6%) with darunavir/ritonavir and three (2.4%) with lopinavir/ritonavir. The median time of follow-up on darunavir/cobicistat was of 20 (IQR 12–21) weeks. Two patients discontinued darunavir/cobicistat and 122/124 (98.4%) subjects maintained virologic suppression. One subject experienced virologic failure after 23 weeks on darunavir/cobicistat. One additional patient discontinued because of diarrhoea which was solved switching again to darunavir/ritonavir. Switching to monotherapy strategy with darunavir/cobicistat seems to be effective and safe in the short term in subjects from clinical setting with suppressive and long-term PI monotherapy. Data from long-term effectiveness and clinical trials are needed.

## References

[1] Kakuda TN, Opsome M, Timmers M, Iterbeke K, Van De Casteele T, Hillewaert V (2014). Pharmacokinetics of darunavir in fixed-dose combination with cobicistat compared with coadministration of darunavir and ritonavir as single agents in healthy volunteers. J Clin Pharmacol.

# Sub article: P086 Three-year efficacy and durability of simplification to single-tablet regimen (STR): a comparison between coformulated efavirenz/emtricitabine/tenofovir and rilpivirine/emtricitabine/tenofovir

**Published:** 2016-10-23

**Authors:** Roberta Gagliardini, Alessandra Bandera, Mauro Zaccarelli, Gaetana Sterrantino, Alessandra Latini, Alessandro D'Avino, Chiara Picarelli, Giuseppe Lapadula, Andrea Antinori, Roberto Cauda, Andrea De Luca, Andrea Gori, Simona Di Giambenedetto, Massimiliano Fabbiani

**Affiliations:** 1 Institute of Clinical Infectious Diseases, Catholic University of Sacred Heart, Rome, Italy; 2 Division of Infectious Diseases, San Gerardo Hospital, University of Milano-Bicocca, Monza, Italy; 3 National Institute for Infectious Diseases, Lazzaro Spallanzani IRCCS, Rome, Italy; 4 Clinic of Infectious and Tropical Diseases, Azienda Ospedaliera Universitaria Careggi, Firenze, Italy; 5 Infectious Dermatology and Allergology, Istituti Fisioterapici Ospitalieri, Rome, Italy; 6 Infectious Diseases Unit, Siena University Hospital, Siena, Italy

## Abstract

**Objectives**: To compare safety, efficacy and durability of simplification to different STR (efavirenz (EFV)/emtricitabine (FTC)/tenofovir (TDF) vs. rilpivirine (RPV)/FTC/TDF) in virologically suppressed patients.

**Materials and methods**: We retrospectively analyzed HIV+ patients with HIV RNA <50 copies/mL on a ≥3-drugs non-STR therapy switching to STR in five Italian centres. Patients were followed from baseline (time of switch) until STR discontinuation or a 3-year maximum follow-up. Time to treatment discontinuation (TD) and virologic failure (VF: HIV RNA >50 copies/mL in two consecutive determinations or >1000 copies/mL in one) and their predictors were investigated.

**Results**: A total of 1461 patients were enrolled of which 998 (68%) switching to EFV/FTC/TDF and 463 (32%) to RPV/FTC/TDF (characteristics in Table 1). TD occurred in 223 (22%) patients with an incidence of 8.7 per 100 PYFU in EFV/FTC/TDF and in 50 (11%) patients with an incidence of 5.3 per 100 PYFU in RPV/FTC/TDF. VF occurred in 34 (3.4%) patients with an incidence of 1.3 per 100 PYFU in EFV/FTC/TDF and in 24 (5.2%) patients with an incidence of 2.6 per 100 PYFU in RPV/FTC/TDF. At survival analysis, the estimated 3-year probability of remaining free from TD was 77.7% in EFV/FTC/TDF versus 89.2% in RPV/FTC/TDF (p=0.001); after excluding patients switching to EFV/FTC/TDF from EFV+TDF/FTC (sub-population 1, n=806), TD was 71.7% versus 89.2% (p<0.001), respectively. At multivariate Cox regression analysis, predictors of TD were male sex (aHR 0.73, p=0.020), age (aHR 1.01 per year more, p=0.033), switching from NNRTI-based regimen (aHR 0.53, p=0.028), STR regimen (RPV/FTC/TDF vs. EFV/FTC/TDF, aHR 0.35, p<0.001), the latter being confirmed also in sub-population 1 (aHR 0.25, p<0.001) together with time on cART (aHR 1.06, p<0.001). The 3-year probability of remaining free from VF was 96.6% EFV/FTC/TDF versus 94.8% RPV/FTC/TDF (p=0.011) in the overall population, 96.8% versus 94.8% in sub-population 1 (p=0.074). At multivariate analysis, time on cART demonstrated a possible association with VF in the overall population (aHR 1.041, p=0.088), more relevant in sub-population 1 (aHR 1.069, p=0.020). Three years after switch, a statistically significant difference in mean change of total cholesterol and HDL at 3 years and of creatinine at 2 years was observed between the two groups (Figure 1); these data were substantially confirmed until 2 years after switch in sub-population 1 and in those switching from a PI-based regimen.

**Conclusion**: Both regimens showed good safety and efficacy, although switch to RPV/FTC/TDF seemed to be better tolerated and with a better lipid profile while EFV/FTC/TDF seemed to have a lower probability of VF.

Value expressed as N (%).

**Figure 1:** Lipids and creatinine mean changes.

**Abstract P086–Table 1:** Population characteristics

	Overall population (N = 1461)	EFV/FTC/TDF (N = 998)	RPV/FTC/TDF (N = 463)	p
Male gender	1083 (74)	762 (77)	321 (69)	0.003
Risk factor				
Heterosexual	513 (35)	347 (35)	166 (36)	<0.001
Homosexual/bisexual	443 (30)	310 (31)	133 (29)	
IDU	167 (11)	97 (8)	70 (15)	
Other	232 (16)	186 (19)	46 (10)	
HCV +	215 (15)	127 (15)	88 (26)	<0.001
Prior AIDS events	336 (23)	239 (26)	97 (22)	0.144
Age (years), median (IQR)	44 (37–50)	44 (37–49)	46 (38–52)	0.014
Time from HIV diagnosis (years), median (IQR)	6.9 (2.6–12.9)	6.0 (2.4–11.4)	8.9 (3.8–16.5)	<0.001
Time of ARV (years), median (IQR)	4.6 (1.2–9.9)	3.9 (1–8.9)	6.0 (2.2–13.0)	<0.001
Baseline CD4+(cells/µL), median (IQR)	573 (424–773)	546 (400–717)	662 (480–905)	<0.001
Nadir CD4+(cells/µL), median (IQR)	224 (102–328)	213 (98–315)	248 (119–391)	<0.001

# Sub article: P087 Efficacy and safety of switch from DRV/r to DRV/COBI in HIV monotherapy

**Published:** 2016-10-23

**Authors:** Ángela Gutiérrez Liarte, Ana Gómez Berrocal, Rosa De Miguel Buckley, Jesús Sanz Sanz

**Affiliations:** 1 Infectious Diseases, Hospital La Princesa, Madrid, Spain

## Abstract

**Introduction**: DRV/r monotherapy is an effective maintenance strategy endorsed by clinical practise guidelines for selected patients. Cobicistat (COBI) is a booster that is as an alternative to ritonavir. DRV/COBI has got equivalent efficacy and safety profile as DRV/r. Coformulated DRV/COBI 800/150 mg has been recently approved for commercialization, which can simplify treatment regimens. Primary endpoint of this study was a combined efficacy endpoint of coformulated DRV/COBI 800/150 mg monotherapy as a simplification strategy in patients receiving DRV/r. Efficacy was defined as RNA HIV <50 copies/mL and the absence of treatment switch or discontinuation. Secondary endpoints were to analyze the drug safety profile and the development of resistance mutations in case of virologic failure.

**Materials and methods**: Observational, longitudinal, retrospective cohort study of 59 patients on previous monotherapy with DRV/r that were switched to coformulated DRV/COBI 800/150 mg, at the Infectious Diseases Department at La Princesa Hospital (Madrid, Spain). Efficacy and safety data from baseline and control point were collected. Failure was defined as RNA HIV >50 copies/mL in a unique determination, according to the snapshot algorithm or treatment switch or discontinuation. For safety evaluation, clinical events and laboratory data were reviewed. Adverse events were graded according to the Division of AIDS classification system. Statistical analysis was performed with IBM SPSS Statistics V22.0.

**Results**: After 9.85 weeks of median follow-up, the combined efficacy was 96.6%. There were two cases of virologic failure. Both patients presented a CD4 nadir count below 200×10^6^ cells/L, no previous history of virologic failure or resistance mutations, and RNA HIV <50 copies/mL at baseline. One of them (1.7%) acquired the E138A mutation in the reverse transcriptase, without the appearance of resistance mutations for protease inhibitors. Adverse events were described in 10.2% of the patients, all of which were grade 2. There were no treatment switches or discontinuations. Reduction of 5.38 mL/min/m^2^ in the GFR was observed. Total cholesterol levels increased 6.8 mg/dL.

**Conclusion**: DRV/COBI 800/150 mg is an effective and safe regimen that allows to simplify boosted DRV monotherapy for the treatment of HIV.

# Sub article: P088 Efficacy and safety of dual therapy with Rilpivirine and boosted Darunavir in treatment-experienced HIV patients

**Published:** 2016-10-23

**Authors:** Juan Pasquau, Samantha de Jesus, Carmen Hidalgo-Tenorio, Piedad Arazo, Maria Jose Crusells, Maria Jose Rios, Fernando Lozano, Javier de la Torre, Carlos Tornero, Coral García-Vallecillos, Guillermo Verdejo, Zaira Palacios, Gloria Samperiz, Maria Jose Galindo, Jose Alberto Terron, Miguel Garcia-Deltoro

**Affiliations:** 1 Infectious Diseases, Hospital Virgen de las Nieves, Granada, Spain; 2 Infectious Diseases, Hospital Miguel de Servet, Zaragoza, Spain; 3 Infectious Diseases, Hospital Lozano Blesa, Zaragoza, Spain; 4 Infectious Diseases, Hospital Virgen Macarena, Sevilla, Spain; 5 Infectious Diseases, Hospital de Valme, Sevilla, Spain; 6 Infectious Diseases Hospital Costa del Sol, Marbella, Spain; 7 Infectious Diseases, Hospital Francesc Borja, Gandia, Spain; 8 Infectious Diseases, Hospital Clinico de Valencia, Valencia, Spain; 9 Infectious Diseases, Hospital de Jerez, Jerez de la Frontera, Spain; 10 Hospital General de Valencia, Infectious Diseases, Valencia, Spain

## Abstract

**Introduction**: Long-term care and prevention of cumulative toxicity related to antiretroviral therapy (ART) have become main objectives of HIV patient management. Nuke-sparing regimens offer an alternative to conventional therapy, with similar efficacy and less potential toxicity. Dual therapy with rilpivirine and boosted darunavir (RPV+DRVb) is an attractive combination, currently used in clinical practise but with little data from clinical trials. For this reason, we have retrospectively analyzed why this combination is being used as well as its efficacy and safety in real-life patients.

**Methods**: Here we present preliminary data of an observational, multi-centre, retrospective study in HIV patients that have received RPV+DRVb for at least 24 weeks to optimize and/or simplify their previous ART.

**Results**: Data from 140 patients of 15 hospitals were collected with a median (m) age of 47 years, 25.7% had previous AIDS stage and CD4+ lymphocyte nadir of 163 cells/µL (m) (IQR 61–283). They had been diagnosed with HIV for 239 months (m) and had received 124 months (m) of ART, with five previous treatment combinations (m). The reason for switch was simplification/optimization (47.9%), toxicity or intolerance (20%), and insufficient efficacy (with VL<1000 copies/mL) of previous ART (7.9%). A total of 23.6% of patients presented a baseline VL between 50 and 1000 copies/mL. In 82.9%, the combination was boosted with ritonavir and 43.6% of these patients switched to cobicistat during the study. In the “*intention-to-treat*” analysis at 24 weeks, 92.6% of 122 patients continue on study treatment without virological failure (VF) criteria. Only 4.1% had VF and of the remaining 3.3%, 0.8% abandoned treatment and 2.5% presented toxicity or intolerance. Data from 18/140 patients still have to be collected. In the analysis of virological efficacy of “*observed data*,” the last observed viral load of the 140 patients who received the combination was <50 copies/mL in 87.1% of cases.

**Conclusions**: We have observed that dual therapy with RPV+DRVb is being used in clinical practise, and it has proven to be effective in a group of patients with a different profile from that of those required to test monotherapy or other dual therapies with lamivudine (advanced HIV infection, long exposure to ART, low CD4+ nadir and low level viremia).

# Sub article: P089 Simplification to dual protease inhibitor (PI)-, integrase strand transfer inhibitor (INSTI)- and non-nucleoside reverse-transcriptase inhibitor (NNRTI)-based less drugs regimens in treatment-experienced HIV-1-infected patients

**Published:** 2016-10-23

**Authors:** Antonio Mastroianni, Elisabetta Briganti, Francesco Allegrini, Carmela Grosso, Sandra Brighi, Gianfranco Ravaglia, Fabio Pieraccini, Claudio Cancellieri

**Affiliations:** 1 UO Malattie Infettive, Ospedale G.B. Morgagni – L. Pierantoni, Forlì, Italy; 2 UO Farmacia, Ospedale G.B. Morgagni – L. Pierantoni, Forlì, Italy

## Abstract

**Introduction**: A retrospective analysis was undertaken to assess viro-immunological and clinical outcomes of ritonavir (Rt)-boosted protease inhibitor (PI)-based and integrase strand transfer inhibitor (INSTI)-based regimens versus NRTI-NNRTI-based less drugs regimens (LDR) in adult patients with a previous history of continuous three-drugs PI- or NNRTI-based ART, who started dual ART, once both plasma viral load (PVL) <40 copies/mL and CD4+ cell count >200 cells/µL were achieved for at least 48 months.

**Materials and methods**: This was a monocentric, retrospective study in a large tertiary care centre in Italy. HIV-infected patients receiving Rt-boosted PI- and INSTI-based dual therapy (ATV/Rt+LMV, ATV/Rt+LMV, LPV/Rt+LMV, RAL+NVP, 3TC+RAL) versus NRTI-NNRTI-based (3TC+NVP, 3TC+EFV) LDRs were systematically identified. The primary outcome was the proportion of patients who maintained virological suppression at week 12, 24 and 48. Other primary outcomes included immunological response, treatment failure and development of drug resistance. The secondary endpoint was safety (serious adverse effects, AIDS-related events and death). Follow-up consisted of clinical assessments and routine laboratory monitoring, and neurocognitive status at baseline and regularly at weeks 4, 8, 12 and every 12 weeks.

**Results**: Using our electronic medical database (Log80 software), we exhaustively identified 130 patients that met our inclusion criteria out of 790 HIV+ patients followed at the Infectious Diseases Unit of Forlì and Cesena Hospitals, searching up to 30 June 2016. Patient median age was 54 years; 56% were men. All of the patients were treatment experienced. Median baseline CD4+ cell count was 803,065/µL and viral load was <40 copies/mL in all patients, respectively. At 12–18 months, an estimated 100% of patients maintained undetectable viral load, there were no changes in CD4+ cell count from baseline, there were no adverse events or communication of new pathology and/or adherence problems. Dual ART selection, according to the tolerability profile, the presence of comorbidities and HIV-1 drug susceptibility testing, included: DRV/Rt+LMV (41 patients), ATV/Rt+LMV (30 patients), 3TC+NVP (25 patients), LPV/Rt+LMV (11 patients), RAL+NVP (6 patients), 3TC+EFV (3 patients) and 3TC+RAL (3 patients).The most common reason for modifying ART was the development of dyslipidaemia and bone mineral density changes, and less frequently renal toxicity and hepatotoxicity.

**Conclusions**: In our experience, both PI-, INSTI- and NNRTI-based less drugs regimens provided a high proportion of durable virological suppression and the average CD4+ count has remained above 350.

# Sub article: P090 Dolutegravir and unboosted atazanavir: a dual NRTI- and booster-free antiretroviral regimen simplification in HIV-1 infected patients with viral suppression

**Published:** 2016-10-23

**Authors:** Agostino Riva, Andrea Poli, Stefano Rusconi, Stefano Bonora, Anna Maria Cattelan, Eugenia Quiros Roldan, Micol Ferrara, Vincenzo Spagnuolo, Layla Pagnucco, Silvia Cavinato, Dario Cattaneo, Roberto Gulminetti, Antonella Castagna

**Affiliations:** 1 III Division of Infectious Diseases, ASST FBF-Sacco, Milan, Italy; 2 Infectious Diseases Unit, San Raffaele Scientific Institute, Milan, Italy; 3 Department of Biomedical & Clinical Sciences, University of Milan, Milan, Italy; 4 Infectious Diseases Unit, University of Turin, Turin, Italy; 5 Infectious Diseases Unit, University of Padova, Padova, Italy; 6 Infectious Diseases Unit, Universita’ degli Studi di Brescia, Brescia, Italy; 7 Infectious Diseases Unit, Fondazione IRCCS Policlinico San Matteo, Pavia, Italy; 8 Unit of Clinical Pharmacology, ASST FBF-Sacco, Milan, Italy

## Abstract

**Introduction**: There are increasing concerns about long-term toxicity of antiretroviral treatment. NRTIs have the potential for long-term toxicities and ritonavir has negative metabolic consequences and drug-drug interactions. The combination of dolutegravir (DTG) with unboosted atazanavir (uATV) is an intriguing new NRTI- and booster-free regimen. We report a real-life experience of the simplification of different antiretroviral regimens to DTG+uATV.

**Methods**: A total of 61 patients were enrolled in our observational study; 58 subjects with at least one follow-up visit. We evaluated several laboratory parameters including CD4 T cell, HIV RNA and metabolic values. We measured ATV and DTG trough concentrations after a minimal 2-week interval from the start of the new regimen.

**Results**: Patients enrolled in the study were predominantly males (63%), CDC stage C was 22%, HCV-Ab positivity was 28% and the previous regimen included more frequently =3 drugs, mainly PIs (90%). Patients had a median time since first HIV-positive test of 16.1 years (10.2–23.6) and a median time of ART exposure of 14.3 years (9.0–19.0). The reasons for switching to uATV + DTG were several: mainly toxicities, comorbidities and simplification (Table 1). As far as uATV: 55 patients were administered 400 mg QD, two patients 300 mg QD and one patient 200 mg BID; DTG was dosed 50 mg QD, but one patient received 50 mg BID. Patients had a median follow-up of 4.9 months (IQR 2.3–7.8). At last visit, all patients on treatment had undetectable HIV RNA. Two patients presented a viral blip during follow-up (91 and 98 copies/mL), subsequently HIV RNA returned negative without treatment modification. There were three treatment discontinuations: one severe hyperbilirubinemia (grade 3), one G-I intolerance and one patient was lost to follow-up. No differences were found in laboratory parameters between baseline and the last follow-up including immuno-virologic variables, except for a significant decrease in tryglicerides (Table 2). ATV and DTG mean concentrations were 310 ng/mL (95% CI 116–504) and 3216 ng/mL (95% CI 2436–3996) respectively. ATV concentration was below 150 ng/mL in 11 out of 28 patients.

**Conclusions**: ART switch towards this dual-drug regimen NRTI and booster-sparing, although in a short follow-up, appears to be well tolerated and safe. Virologic suppression was maintained in all patients despite long-lasting HIV infection and ART treatment. DTG concentrations are high in the majority of the patients as expected from previous pharmacokinetics study. Despite low ATV concentrations in several patients, no virologic failures were observed. This NRTI- and RTV-sparing regimen appears an attractive new strategy in patients with metabolic disorders and NRTI-related toxicities.

**Table 1:** Baseline characteristics of the studied population

Characteristic	Baseline
	(n = 61)
Age (years)	52.1 (47.0–58.4)
Years since first HIV-positive test	16.1 (10.2–23.6)
Years of ARV exposure	14.3 (9.0–19.0)
CDC stage	
A1	7 (11%)
A2	12 (20%)
A3	6 (10%)
B2	13 (21%)
B3	3 (5%)
C2	1 (2%)
C3	12 (20%)
Unknown	7 (11%)
Male gender	37 (61%)
Risk factor	
Man who has sex with man	12 (20%)
Heterosexual	24 (39%)
Intravenous drug user	17 (28%)
Other/unknown	8 (13%)
White race	58 (95%)
European origin	58 (95%)
HCV-Ab positivity	17 (28%)
Genotype 1a	6
Genotype 1b	5
Genotype 3	2
Genotype 4	2
Unknown genotype	2
Type of ART regimen	
PI/r monotherapy	2 (3%)
Dual therapy	16 (26%)
Three or more drugs	43 (71%)
Type of ART regimen according to the drug class	
PI-based	55 (90%)
INSTI-based	3 (5%)
NNRTI-based	3 (5%)
Reasons for switch to ATV + DTG regimen	
Cardiovascular disease	2 (3%)
Concern of cardiovascular disease	7 (12%)
Dyslipidemia	8 (13%)
Comorbidity	4 (7%)
Immunologic failure – CD4 drop	1 (1.5%)
Non-compliance	1 (1.5%)
Simplified treatment available	15 (25%)
Toxicity – GI tract/abdomen	5 (8%)
Toxicity – liver	3 (5%)
Toxicity – kidney	8 (13%)
Toxicity – not specified	2 (3%)
Treatment failure	1 (1.5%)
Side effects	1 (1.5%)
Other	3 (5%)

**Abstract P090–Table 2:** Laboratory parameters at baseline and at last visit

Characteristic	Baseline (n = 61)	Last visit (n = 58)	Change from baseline	p
CD4+ (cells/µL)	733 (578–946)	690 (513–872)	−6 (−150; + 83)	0.395
CD8+ (cells/µL)	850 (583–1159)	840 (560–1169)	−40 (−165; + 117)	0.977
CD4+ and CD8+ ratio	0.90 (0.62–1.23)	0.90 (0.63–1.20)	+0.07 (−0.10; + 0.15)	0.988
White blood cells (109/L)	6.7 (5.5–8.4)	6.2 (5.5–7.4)	−0.2 (−0.8; + 0.6)	0.290
ALT (U/L)	26 (20–42)	29 (21–37)	+1 (−5; + 7)	0.829
AST (U/L)	26 (22–35)	25 (20–32)	0 (−4; + 4)	0.535
Gamma GT (U/L)	27 (18–49)	21 (17–39)	−1 (−7; + 3)	0.262
Total bilirubin (mg/dL)	2.03 (1.08–3.00)	1.58 (0.99–2.28)	−0.72 (−1.58; −0.01)	0.168
Direct bilirubin (mg/dL)	0.72 (0.40–0.97)	0.57 (0.36–0.72)	−0.07 (−0.19; + 0.08)	0.136
Indirect bilirubin (mg/dL)	1.21 (0.64–2.00)	0.81 (0.50–1.58)	−0.50 (−1.16; −0.42)	0.544
FIB-4	1.18 (0.91–1.61)	1.23 (0.87–1.68)	−0.04 (−0.13; + 0.10)	0.866
Creatinine (mg/dL)	0.90 (0.80–1.04)	0.96 (0.81–1.16)	0.08 (0; + 0.15)	0.240
EGFR (mL/min/1.73 m^2^)	89 (76–100)	83 (66–96)	−5 (−13;0)	0.300
Total cholesterol (mg/dL)	194 (156–219)	184 (165–216)	2 (−21; + 15)	0.790
LDL cholesterol (mg/dL)	119 (90–149)	108 (85–131)	+3 (−7; + 13)	0.363
HDL cholesterol (mg/dL)	46 (39–57)	50 (42–65)	+7 (−4; + 12)	0.091
Total and HDL cholesterol ratio	3.93 (3.08–5.29)	3.58 (2.88–4.44)	−0.25 (−0.85; + 0.29)	0.116
Triglycerides (mg/dL)	139 (86–193)	108 (84–145)	−13 (−70; + 18)	0.049
Glucose (mg/dL)	92 (84–98)	91 (84–106)	0 (−9; + 5)	0.814
Haemoglobin (109/L)	14.8 (13.9–15.9)	14.7 (13.9–15.7)	−0.2 (−0.6; + 0.4)	0.616
Platelets (109/L)	223 (174–272)	230 (180–276)	0 (−19; + 12)	0.828

Mann–Whitney tests were used to evaluate differences between BL and follow-up; results are reported as median (IQR) or frequencies (%).

# Sub article: P091 Treatment of HIV-positive patients with lamivudine plus boosted protease inhibitor in a real-world setting

**Published:** 2016-10-23

**Authors:** Chien-Yu Cheng, Shu-Hsing Cheng, Shu-Yin Chang, Mei-Hui Lin, Shin-Yen Ku, Hui-Ting Shieh, Na-Lee Sun

**Affiliations:** 1 Department of Internal Medicine, Taoyuan General Hospital, Ministry of Health and Welfare, Taoyuan City, Taiwan; 2 AIDS Care Center, Taoyuan General Hospital, Ministry of Health and Welfare, Taoyuan City, Taiwan

## Abstract

**Introduction**: Some adverse effects of nucleoside reverse transcriptase inhibitors including renal injury, myocardial injury and osteoporosis could limit the prescription of antiretroviral therapy in HIV-positive patients, especially the aging patients with non-communicable diseases. The GARDEL and OLE study of lamivudine plus lopinavir/ritonavir demonstrated effectiveness and safety of dual therapy [1]. However, the information of dual therapy in real world remains limited, so we launch an observational study to monitor effectiveness and safety of dual therapy of lamivudine plus boosted protease inhibitors.

**Materials and methods**: This prospective study was launched to evaluate the effectiveness of virologic response and safety of dual therapy of lamivudine (300 mg) plus lopinavir/ritonavir and darunavir/ritonavir, and naïve and treatment-experienced HIV-positive patients were enrolled since May 2015. Patients with positive hepatitis B antigen were excluded. In weeks 12, 24 and 48, CD4 lymphocyte cell counts and plasma HIV RNA were measured, and these data are compared with those at baseline before enrolment. All adverse effects are documented during the study.

**Results**: Since May 2015 to May 2016, 116 naïve patients (group 1), 39 (group 2) treatment-experienced patients without suppressed HIV RNA (>50 copies/mL) and 82 (group 3) treatment-experienced patients with suppressed HIV RNA (<50 copies/mL) were enrolled in the study. The median age was 34±9 and 38±8 years in group 1 & 2 and group 3 (p<0.001), and 94.2% and 79.3% (p<0.001) were male patients, respectively. Positive HCV Ab was 36.1% versus 37.3% (p=0.79). Median baseline CD4 cell counts before enrolment were 338 (5–908) and 490 (86–1071) cells/L (p<0.001). However, rate of virologic failure (plasma HIV RNA >200 copies/mL) was 10.3% (10/97) and 1.3% (1/76) (p=0.016, ORs 8.62, 95% CI 1.08–68.91) for group 1 & 2 and group 3 in week 24. Treatment failure was 20% versus 7.3% (p=0.01, ORs 3.23, 95% CI 1.10–9.49), respectively. Thirty-seven patients discontinued dual therapy, 20% patients (29/145) taking lopinavir/ritonavir had severe GI tract upset or diarrhoea and 7.3% patients (8/110) taking darunavir/ritonavir had grade 3 urticaria or diarrhoea (p=0.004, ORs 3.19, 95% CI 1.40–7.29) (Figure 1).

**Conclusions**: Lamivudine plus lopinavir/ritonavir or darunavir/ritonavir demonstrated comparable effectiveness for patients with suppressed HIV RNA in our cohort. However, adverse effects of GI tract upset and diarrhoea could impede application of dual therapy for naïve patients in real world.

**Abstract P091–Figure 1:** Virologic failure (VF) and treatment failure (TF) of dual therapy in 116 naïve patients, 39 treatment-experienced patients without suppressed plasma HIV RNA and 86 patients with suppressed plasma HIV RNA.

## References

[1] Cahn P, Andrade-Villanueva J, Arribas JR, Gatell JM, Lama JR, Norton M (2014). Dual therapy with lopinavir and ritonavir plus lamivudine versus triple therapy with lopinavir and ritonavir plus two nucleoside reverse transcriptase inhibitors in antiretroviral-therapy-naïve adults with HIV-1 infection: 48 week results of the randomised, open label, non-inferiority GARDEL trial. Lancet Infect Dis.

# Sub article: P092 Effectiveness and convenience of ATV/r+3TC dual therapy regimen in a real-life cohort of HIV-infected patients: 48-week follow-up

**Published:** 2016-10-23

**Authors:** Sergio Maria Ferrara, Alessandra Tartaglia, Salvatore Sica, Teresa Antonia Santantonio

**Affiliations:** 1 Infectious Disease Unit, Ospedali Riuniti Foggia, Foggia, Italy; 2 Infectious Disease Unit, Azienda Sanitaria Locale della Provincia di Foggia, Foggia, Italy

## Abstract

**Introduction**: Simplification to a dual therapy improves adherence by reducing pill burden, short- and long-term toxicities and lowers additional costs for clinical management. ATV/r+3TC dual therapy has recently been approved by Italian and European guidelines as switching strategies from standard cART in virologically suppressed patients. The aim of this 48-month observational study in a real-life setting was to evaluate the efficacy, safety and impact on cardiovascular event of ATV/r+3TC dual therapy.

**Methods**: We enrolled 55 HIV-positive patients on stable HIV VL suppression in the last 6 months, without resistance to PI and in absence of chronic HBV co-infection. Cardiovascular risk was evaluated using the Framingham risk score (FRS). Carotid intima-media thickness (c-IMT) was assessed by colour Doppler ultrasonography and a c-IMT >0.9 mm was considered to be pathological.

**Results**: Thirty-five patients were males (64.2%), and median age was 49 years (range 28–74 years) (Table 1). Thirteen subjects (24.5%) had AIDS and 15 (27.3%) were HCV co-infected. A total of 32 patients (58.2%) were smokers. Length of HIV diagnosis was 12.5 years (1–33 years). Cumulative exposure to HAART was 9 years (1–20 years). Median baseline CD4 count was 674 cells/mm^3^. In previous regimen, ARV backbone was TDF/FTC (81.8%) and ABC/3TC (18.2%). Reasons for switching were: 43 simplification (78.2%); 12 TDF-related toxicities (21.8%) distributed as follows: seven (58.3%) renal toxicity and five (41.7%) osteoporosis.

After 48 weeks of dual therapy all participants maintained virological suppression and no severe adverse events or treatment interruptions were registered; mean CD4 count gain was +58 cells/mm^3^. A significant increase in mean lipid concentration was reported for HDL cholesterol (+7.9 mg/dL, p<0.0001), LDL cholesterol (+2.2 mg/dL, p<0.0001) and triglycerides (+20.5 mg/dL, p<0.0001) while total cholesterol decreased (−6.5 mg/dL, p<0.0001). However, total cholesterol/HDL ratio and LDL/HDL ratio did not demonstrate any significant change (mean changes at 48 months −0.06 and −0.003). No progression of c-IMT was observed at 48 weeks leading to an improvement of FRS (p=0.003). Hyperbilirubinaemia correlated with c-IMT at 48 weeks (p=0.0009) suggesting a potential anti-inflammatory and protective role. Slow c-IMT progression correlated with CD4/CD8 ratio <0.8 (p=0.004) which seems to be a surrogate predictor of CV risk. A significant increase of eGFR (CKD-EPI mL/min/1.732) was observed in patients switching for TDF toxicity (p=0.005). Moreover, the use of ATV/r+3TC regimen permitted in our cohort to save of €242,676 per year compared with conventional therapy. (Table 2)

**Conclusions**: Dual ATV/r+3TC therapy may optimize cART and helps clinicians to avoid drawbacks and toxicities due to NRTI backbone, while maintaining the efficacy and the convenience of a robust cART.

**Table 1:** Characteristics of patients (total patients = 55)

Total N of patients = 55	
Sex, n (%)	
Females	20 (36.4%)
Males	35 (63.6%)
Age, years, median (range)	
Total	49 (28–74)
Males	50.5 (28–74)
Females	47 (32–70)
Risk factor, n (%)	
Heterosexual	28 (50.9%)
MSM	11 (20%)
TD	16 (29.1%)
CDC stage, n (%)	
C3	14 (25.4%)
Other	41 (74.5%)
HAART exposure, years, median (range)	9 (1–20)
Length of HIV infection, years, median (range)	12.5 (1–8)
Previous HAART regimens	
PI (%)	24 (43.6%)
NN (%)	5 (9.1%)
Naive	26 (47.3%)
HCV co-infection, n (%)	15 (27.3%)
Comorbidities	
Total	39/55 (70.96%)
Smoking	32/39 (82.1%)
Diabetes	3 (7.7%)
Hypertension	5 (12.8%)
Dyslipidaemia	5 (12.8%)

**Abstract P092–Table 2:** Costs and saving of dual therapy compared to conventional treatment in our cohort (Foggia, Italy)

cART regimens	Monthly costs (Ospedali Riuniti Foggia, Italy)	Monthly saving per patient (compared with dual ATV/r + 3TC therapy)	Monthly saving (compared with dual ATV/r + 3TC therapy)	Saving per year (compared with dual ATV/r + 3TC therapy)
ATV/r + TDF/FTC	€724.5	n = 45 patients: €16848.0	n = 45 patients: €16,848.0	n = 45 patients: €202,176.0
ATV/r + ABC/3TC	€687.6	€337.5	n = 10 patients: €3375.0	n = 10 patients: €40,500.0
ATV/r + 3TC	€350.1	–	–	n = 55 patients: €242,676.0

# Sub article: P093 Efficacy and safety of emtricitabine/tenofovir alafenamide (FTC/TAF) versus emtricitabine/tenofovir disoproxil fumarate (FTC/TDF) as a backbone for treatment of HIV-1 infection in virologically suppressed adults: subgroup analysis by third agent

**Published:** 2016-10-23

**Authors:** Frank Post, Yazdan Yazdanpanah, Gabriel Schembri, Adriano Lazzarin, Jacques Reynes, Franco Maggiolo, Mingjin Yan, Michael Abram, Cecilia Tran-Muchowski, Andrew Cheng, Martin Rhee

**Affiliations:** 1 King's College Hospital NHS Foundation Trust, London, UK; 2 Service des Maladies Infectieuses et Tropicales, Hopital Bichat-Claude Bernard, Paris, France; 3 Manchester Centre for Sexual Heath, Central Manchester University Hospitals NHS Foundation Trust, Manchester, UK; 4 Divisione di Malattie Infettive, IRCCS Ospedale San Raffaele, Milano, Italy; 5 Maladies Infectieuses et Tropicales, Centre Hospitalier Régional Universitaire de Montpellier, Montpellier, France; 6 Infectious Diseases, Azienda Ospedaliera Papa Giovanni XXIII, Bergamo, Italy; 7 Biostatistics - HIV, Gilead Sciences, Inc, Foster City, CAUSA; 8 Clinical Virology, Gilead Sciences, Inc., Foster City, CA, USA; 9 Clinical Operations - HIV, Gilead Sciences, Inc., Foster City, CA, USA; 10 Clinical Research - HIV, Gilead Sciences, Inc., Foster City, CA, USA

## Abstract

**Introduction**: The efficacy and safety of TAF has been mostly evaluated in the context of the coformulation of elvitegravir (E), cobicistat (C), FTC (F) and TAF (E/C/F/TAF). Multiple clinical trials of E/C/F/TAF have consistently demonstrated the advantages of TAF over TDF for markers of renal and bone safety. However, it has not been shown whether these safety advantages of TAF are seen when combined with other third agents.

**Materials and methods**: We conducted a subgroup analysis by the class of co-administered third agent (boosted protease inhibitors (PIs) vs. unboosted third agent) for efficacy (prespecified) and safety (ad hoc) from a 48-week randomized, double-blind, active-controlled study in virologically suppressed, HIV-positive participants who switched to FTC/TAF from FTC/TDF versus continuing FTC/TDF while remaining on their original third agent. The prespecified non-inferiority margin for the overall study was 10%.

**Results**: Among 663 treated (FTC/TAF n=333, FTC/TDF n=330), 47% and 46%, respectively, received boosted PIs. Median age (49 years), median CD4 count (646 cells/mL), renal laboratory parameters and bone mineral density (BMD) were similar between the subgroups. Overall, median duration of FTC/TDF use prior to dosing was 5.1 years. At week 48, significant differences in changes of renal biomarkers and BMD were observed favouring FTC/TAF over FTC/TDF (p<0.05 for all), with similar improvements within the FTC/TAF arm in those who received boosted PIs versus unboosted third agents (Table 1). Few participants discontinued study drug due to adverse events in either subgroup (boosted PI: FTC/TAF 4%, FTC/TDF 1%; unboosted third agent: FTC/TAF 1%, FTC/TDF 1%). No cases of Fanconi syndrome or proximal renal tubulopathy were reported. Switching to an FTC/TAF-containing regimen was non-inferior to staying on a baseline FTC/TDF-containing regimen in maintaining HIV-1 RNA less than 50 copies/mL for participants receiving either a boosted PI (92% vs. 93%; difference: −1.1%; 95% CI −7.1–4.9%) or an unboosted third agent (97% vs. 93%; difference: 3.3%; 95% CI −1.2–7.9%).

**Conclusions**: Regardless of third agent (boosted PI or unboosted third agent), FTC/TAF was non-inferior to FTC/TDF in maintaining virologic suppression at week 48 and renal and bone parameters significantly improved in those who switched from TDF to TAF. Overall safety was similar for FTC/TAF administered with a boosted PI or an unboosted third agent. FTC/TAF can be an important NRTI backbone option that can be used in combination with a variety of third agents.

**Abstract P093–Table 1:** Changes in measures of renal and bone safety by third agent

	**Boosted PI**	**Boosted PI**	**Unboosted third agent**	**Unboosted third agent**

	**FTC/TAF (n = 155)**	**FTC/TDF (n = 151)**	**FTC/TAF (n = 178)**	**FTC/TDF (n = 179)**
eGFR				
Median baseline (mL/min)	102.2	104.5	98.3	97.2
Median changes at week 48* (mL/min)	+7.7	+3.3	+9.3	+2.8
Urine protein: creatinine ratio				
Median baseline value (mg/g)	57.8	66.7	60.6	59.6
Median % changes at week 48*	−11.1	+12.8	−16.9	+2.1
Urine albumin: creatinine ratio				
Median baseline value (mg/g)	6.3	6.4	5.8	6.1
Median % changes at week 48*	–1.8	+21.2	−11.6	+4.4
Urine beta-2-microglobulin: creatinine ratio				
Median baseline value (µg/g)	140.3	186.5	131.9	134.2
Median % changes at week 48*	−39.3	+36.4	−40.2	+14.0
Urine retinol binding protein: creatinine ratio				
Median baseline value (µg/g)	112.4	117.5	100.9	106.8
Median % changes at week 48*	−13.5	+24.8	−17.3	+11.8
Spine BMD				
Mean baseline value (g/m^2^)	1.08	1.07	1.09	1.08
Mean % changes at week 48*	+1.48	−0.40	+1.45	−0.13
Hip BMD				
Mean baseline (g/m^2^)	0.98	0.97	0.98	0.98
Mean % changes at week 48*	+1.10	−0.08	+1.00	−0.22

* p-values for all between-group differences (FTC/TAF vs. FTC/TDF) were <0.05.

# Sub article: P094 Population pharmacokinetics (PK) of dolutegravir (DTG) alone and following treatment switch

**Published:** 2016-10-23

**Authors:** Laura Dickinson, Margherita Bracchi, Emilie Elliot, Laura Else, Saye Khoo, David Back, Mark Nelson, Marta Boffito

**Affiliations:** 1 Department of Molecular & Clinical Pharmacology, University of Liverpool, Liverpool, UK; 2 St Stephen's Centre, Chelsea & Westminster Foundation Trust, London, UK

## Abstract

**Introduction**: The integrase inhibitor DTG is a preferred antiretroviral in many treatment guidelines. Efavirenz (EFV) induces DTG UGT1A1 and CYP3A4-dependent metabolism but dose adjustments are not recommended following treatment switch with steady-state DTG reached week 4 post-switch [1,2]. Population PK analysis was performed to describe DTG PK and investigate changes in DTG after switching from an EFV-based regimen.

**Materials and methods**: Model development (NONMEM version 7.3) combined DTG concentration-time data (50 mg once daily) from two studies. Study 1 was in healthy volunteers administered DTG for 10 days with serial blood sampling performed for 216 hours following the final dose [3]. Study 2 was in HIV-infected, virologically suppressed patients switched from EFV to DTG with random single samples drawn at week 1, 2, 3 and 4 post-switch (samples between 1 and 25.75 hours post-dose). The impact of residual EFV on DTG apparent oral clearance (CL/F) after switching compared with DTG alone was determined. Covariates including weight, age, BMI, sex, ethnicity, HIV status and food consumption within 3 hours of drug intake were also assessed and the model evaluated by simulation and visual predictive check.

**Results**: Fifty-six individuals were included (n=14 female, n=35 Caucasian; n=17 healthy, n=39 HIV). DTG up to 216 hours was described by a two-compartment model parameterized by CL/F (estimate (RSE%): 0.85 L/h (5%)), central volume of distribution (Vc/F: 17 L (7%)), intercompartmental clearance (Q/F: 0.0082 L/h (20%)) and peripheral volume of distribution (Vp/F: 0.73 L (8%)) with absorption rate constant fixed to 2.24 hours^−1^ [4]. Interindividual variability was 17% (41%) and 16% (39%) for CL/F and Vc/F, respectively. Following multivariate analysis weight was the only significant covariate to remain in the model. DTG CL/F was increased by 34%, 60%, 13% and 11% at week 1, 2, 3 and 4 following switch, respectively compared with DTG alone. Based on 100 simulations DTG AUC_0–24_, C_max_ and trough (C_24_) at week 1, 2, 3, 4 post-switch were significantly lower than DTG alone (Table 1); however, all simulated C_24_ were above the protein-adjusted IC_90_ of 0.064 mg/L post-switch (median (range) 0.81 mg/L (0.25–1.75)).

**Conclusions**: Population PK parameters were comparable with previous reports [4] with between-study differences attributable to EFV. Simulated DTG PK parameters were reduced following switch even at week 3/week 4 (~20% for C_24_), potentially highlighting important PK differences between healthy and HIV-infected individuals. However, consistent with recent data [1] concentrations remained above the protein-adjusted IC_90_ post-switch, supporting findings that dose adjustments may not be required in the described patient population.

**Abstract P094–Table 1:** Changes in DTG PK parameters following switch from an EFV-based regimen expressed as geometric mean ratio (GMR; 90% CI) determined from simulations using the final model parameters (n = 100)

	GMR (95% CI; DTG as reference)			
Parameter	Week 1	Week 2	Week 3	Week 4
AUC0-24	0.73 (0.69–0.76)	0.61 (0.58–0.64)	0.87 (0.83–0.91)	0.88 (0.84–0.92)
Cmax	0.84 (0.80–0.88)	0.78 (0.74–0.82)	0.92 (0.88–0.96)	0.92 (0.88–0.97)
C24	0.57 (0.53–0.61)	0.40 (0.37–0.42)	0.79 (0.74–0.84)	0.81 (0.76–0.86)

## References

[1] de Wet J, DeJesus E, Sloan L, Koteff J, Brennan C, Adkison K (2016). Pharmacokinetics of dolutegravir after switching to abacavir/dolutegravir/lamivudine from an efavirenz-based regimen: a PK sub-study from STRIIVING. 17th International Workshop on Clinical Pharmacology of HIV & Hepatitis Therapy.

[2] Generaux G, Song I, Bowers G, Piscitelli S (2014). A mechanistic SimCYP simulation evaluating dolutegravir and efavirenz pharmacokinetics following a switch from once-daily efavirenz to once-daily dolutegravir. 15th International Workshop on Clinical Pharmacology of HIV & Hepatitis Therapy.

[3] Elliot E, Amara A, Jackson A, Moyle G, Else L, Khoo S (2016). Dolutegravir and elvitegravir plasma concentrations following cessation of drug intake. J Antimicrob Chemother.

[4] Zhang J, Hayes S, Sadler BM, Minto I, Brandt J, Piscitelli S (2015). Population pharmacokinetics of dolutegravir in HIV-infected treatment-naïve patients. Br J Clin Pharmacol.

# Sub article: P095 Virological response of HIV-infected patients virologically suppressed switching to a DTG-based regimen in an observational cohort based on the genotypic susceptibility score

**Published:** 2016-10-23

**Authors:** Charlotte Charpentier, Gilles Peytavin, Véronique Joly, Ornella Cabras, Marine Perrier, Minh Lê, Bao Phung, Yazdan Yazdanpanah, Diane Descamps, Roland Landman

**Affiliations:** 1 Laboratoire de Virologie, Hôpital Bichat-Claude Bernard, Paris, France; 2 Laboratoire de Pharmaco-Toxicologie, Hôpital Bichat-Claude Bernard, Paris, France; 3 Service de Maladies Infectieuses et Tropicales, Hôpital Bichat-Claude Bernard, Paris, France

## Abstract

**Introduction**: To assess, in a clinical cohort of virologically suppressed patients, the virologic response after switching ARV treatment to a dolutegravir (DTG)-based regimen based on the genotypic susceptibility score.

**Materials and methods**: A prospective, observational, single-centre cohort enrolling all patients with a plasma viral load (pVL) <50 copies/mL initiating a DTG-based regimen between September and December 2015. VL were performed using Cobas Taqman HIV-1 version 2.0 assay. The Genotypic Susceptibility Score (GSS) of the antiretroviral regimen was calculated using the ANRS version 25 (September 2015) algorithm including DTG, translating the interpretations “susceptible”, “possible resistance” and “resistance” into susceptibility scores of 1, 0.5 and 0, respectively.

**Results**: Among 254 patients who switched to a DTG-based regimen, 209 had historical available genotypes. Among them, taking into account all historical genotypes, 27 (13%), 61 (29%) and 121 (58%) had a GSS equal to 1 or 1.5, 2 and 3, respectively. Median time since last genotype was 9, 9 and 5 years in the 1 or 1.5, 2 and 3 GSS strata, respectively. Patients’ characteristics at time of DTG-based regimen initiation were different according to the GSS, as depicted in Table 1. At week 24, seven patients (3.3%) discontinued DTG-based regimen: neuro-psychological side effects (n=1), cutaneous side effects (n=1), pregnancy (n=1), renal toxicity (n=1), headaches (n=1) and patients’ decision (n=2). At week 12, 95%, 96% and 95% of the patients had pVL <50 copies/mL in the 1 or 1.5, 2 and 3 GSS strata, respectively. At week 24, 100%, 96% and 96% of the patients had pVL <50 copies/mL, in the 1 or 1.5, 2 and 3 GSS strata, respectively. Among the 12 patients (11.9%) displaying a pVL >50 copies/mL (median 102 copies/mL, IQR 61–417), nine experienced a viral blip, one a virologic failure (VF) and in the two remaining patients no further control sample was available. The only one patient (1%) experiencing VF was highly pre-treated including a previous VF under a RAL-based regimen with no selection of integrase resistance mutations and with a GSS=3.

**Conclusions**: In this observational cohort, patients’ characteristics at time of switching to a DTG-based regimen were different depending on the GSS strata. However, short-term follow-up showed a high level of the maintenance of virologic suppression, regardless of the baseline GSS. These data suggest that DTG remains potentially able to maintain viral suppression when combined with fully or incompletely active drugs in these long-term virologically suppressed patients.

**Abstract P095–Table 1:** Patients’ characteristics

Characteristic	GSS = 1 or 1.5 (n = 27)	GSS = 2 (n = 61)	GSS = 3 (n = 121)
Male sex, n (%)	21 (78)	35 (57)	86 (71)
Age, median years (IQR)	51 (46–55)	53 (42–59)	51 (41–58)
Time since HIV diagnosis, median years (IQR)	21 (16–24)	20 (8–21)	13 (6–21)
Duration of prior ART, median years (IQR)	17 (10–20)	18 (5–19)	11 (5–20)
Number of previous ART lines, median (IQR)	8 (5–10)	6 (2–7)	4 (2–7)
Duration of HIV-1 RNA <50 copies/mL before switch, median years (IQR)	3 (2–7)	4 (2–5)	3 (1–5)
Baseline CD4 cell count, median cells/mm^3^ (IQR)	670 (435–863)	645 (535–865)	570 (400–750)
Nadir CD4 cell count, median cells/mm^3^ (IQR)	183 (63–209)	236 (204–386)	221 (85–325)
Associated antiretroviral drugs, n (%)			
ABC/3TC	19 (70)	4 (7)	75 (62)
TDF/FTC	1 (4)	4 (7)	29 (24)
3TC	0 (0)	7 (11)	0 (0)
RPV	3 (11)	26 (43)	0 (0)
DRV/r	0 (0)	5 (8)	0 (0)
ATV/r	1 (4)	6 (10)	0 (0)
RPV + NRTI	0 (0)	3 (5)	10 (8)
Other ARV drugs	3 (11)	6 (10)	7 (6)
Number of NRTI drug resistance mutations, median (IQR)	2 (1–5)	2 (0–3)	0 (0–0)
Number of M184V, n (%)	27 (100)	32 (52)	10 (8)
Number of NNRTI drug resistance mutations, median (IQR)	1 (0–2)	0 (0–2)	0 (0–1)
Number of major PI drug resistance mutations, median (IQR)	0 (0–2)	0 (0–0)	0 (0–0)
Time since last genotypic resistance test, median years (IQR)	9 (4–12)	9 (5–14)	5 (3–12)

# Sub article: P096 Evaluation of virologic efficacy and economic savings in a Portuguese hospital after splitting the single-tablet regimen EFV/FTC/TDF (Atripla^®^) to an equivalent double-tablet regimen (efavirenz+Truvada^®^)

**Published:** 2016-10-23

**Authors:** Mafalda Guimarães, Inês Vaz Pinto, Catarina Santos, Sara Alves

**Affiliations:** 1 Cascais Hospital, HIV Unit, Cascais, Portugal

## Abstract

**Introduction**: Single-tablet regimens (STR) of ART are now widely available. The combination of fixed-dose antiretrovirals in just one tablet has been shown to improve long-term adherence and patients’ satisfaction. Also, STR eliminates the possibility of selective non-adherence. Due to the global economic crisis since 2009 and subsequent financial restraints observed in health systems worldwide, the board of Cascais Hospital decided in April 2014 to split the STR Atripla^®^ for its two constituents, FTC/TDF (Truvada^®^) and efavirenz. The switch from STR to a dual-tablet regimen (DTR) was done by each patient's doctor, after full explanation and patient consent. The purpose of our study was to retrospectively evaluate the impact of splitting a STR into its two separate components on virologic effectiveness at 24 weeks. We also looked at the direct economic benefits obtained with this global switch.

**Material and methods**: The switch from STR to DTR was made between April 1 and June 30, 2014, at the time of clinic appointment or pharmacy refill, where patients were referred to the clinic. We reviewed clinical files and present data on demographic characterization by age, sex and route of HIV transmission. Data were collected on CD4 count and HIV viral load (VL) at the time of switch and at 24 weeks thereafter.

**Results**: On 31 March 2014 a total of 1036 patients at our clinic were on ART; 230 patients were on Atripla^®^ (22.2%). A total of eight patients did not do the split (three lost to follow-up, three had virologic failure, one for lactose intolerance and one for lack of consent). A total of 222 patients were switched from STR to a DTR. At week 24, 31 (13.9%) patients were no longer taking the efavirenz+Truvada^®^ regimen, mainly due to perceived adverse effects of either of the two components of the regimen. Of the 190 patients still taking efavirenz+Truvada^®^ 24 weeks after the split of Atripla^®^, 179 (94%) had VL <20 copies/mL at week 24. Two patients were lost to follow-up, eight had virologic blips (VL >20 but <500 copies/mL) and one had virologic failure. The direct economic savings obtained by splitting a STR was _291,000 in a 9-month period.

**Conclusions**: Our centre's experience with switching all patients on a STR to an equivalent DTR proved to be effective and resulted in significant economic savings that continue to the future.

# Sub article: P097 Dolutegravir plus ritonavir-boosted darunavir in highly cART-experienced subjects: an observational cohort

**Published:** 2016-10-23

**Authors:** Amedeo Ferdinando Capetti, Gaetana Sterrantino, Maria Vittoria Cossu, Giovanni Cenderello, Stefano Rusconi, Serena Cima, GianMaria Baldin, AnnaMaria Cattelan, Giuseppe Vittorio De Socio, Niccolò Riccardi, GianCarlo Orofino, Benedetto Maurizio Celesia, Fosca Niero, Lolita Sasset

**Affiliations:** 1 1st Division of Infectious Diseases, Luigi Sacco University Hospital, Milano, Italy; 2 Division of Infectious Diseases, Careggi Hospital, Firenze, Italy; 3 Division of Infectious Diseases, Ospedali Galliera, Genova, Italy; 4 Infectious Diseases Clinic, Luigi Sacco University Hospital, Milano, Italy; 5 2nd Division of Infectious Diseases, Policlinico San Matteo, Pavia, Italy; 6 2nd Division of Infectious Diseases, Policlinico Universitario Agostino Gemelli, Roma, Italy; 7 Division of Infectious and Tropical Diseases, Azienda Ospedaliera-Universitaria di Padova, Padova, Italy; 8 Infectious Diseases Clinic, Azienda Ospedaliero-Universitaria di Perugia, Perugia, Italy; 9 Infectious Diseases Clinic, San Martino Hospital, Genova, Italy; 10 1st Division of Infectious Diseases, Amedeo di Savoia Hospital, Torino, Italy; 1 Infectious Diseases Unit, University of Catania, ARNAS Garibaldi, Catania, Italy; 2 Division of Infectious Diseases, Santa Maria della Misericordia Hospital, Rovigo, Italy

## Abstract

**Introduction**: In patients switching from complex salvage regimens for simplification or failure, the association of dolutegravir and darunavir provides high genetic barrier to HIV-1 resistance.

**Methods**: All HIV-1 infected subjects treated with dolutegravir plus boosted darunavir between March 2014 and September 2015 were included in an observational cohort. After ethics committees’ approval, no further enrolment was allowed. Only clinical events, demographic data, CD4 cell counts, HIV-1 RNA, serum creatinine and urinary proteins were deemed relevant for this study.

**Results**: Hundred and fourteen subjects were enrolled. The mean age was 51, females were 26.5% and non-Caucasians 9.7%. The main risk factor was being male homosexual (46%), followed by drug abuse (28.3%) and heterosexual intercourse (23.9%). The main reason for switching was simplification (46%), followed by viral failure (27.4%), toxicity (14.9%), persistent low-level viremia (5.3%), lack of adherence (3.5%) and immunologic failure/disease progression (2.6%). RT mutations were present in 83.2%, 80.5% had protease mutations and 10.5% had INSTI mutations. Between week 24 and 48 one subject was lost to follow-up, one died of drug abuse and one of cancer-related sepsis, one dropped out for elevation of liver enzymes, one for dyslipidaemia and one stopped all drugs for personal decision. The proportion of viremic subjects at baseline declined steadily by week 4 from 40.7% to 14.1%, with a >1 log10 decay in all but one, that had stopped the therapy for 3 weeks, and further by week 24 to 6.2% (range 53–805 copies/mL). Measurable viremia <50 copies/mL declined from 20.4% to 5.3%. Subjects at zero viremia increased from 38.9% to 59.3% by week 4 and to 73.5% by week 24. Of the 84 subjects who have a 48-week follow-up, five still have >50 copies HIV-1 RNA/mL (range 51–99), 16 have viral load <50 copies and in 63 zero viremia. Eighteen subjects had reduced sensitivity to darunavir (Stanford median score 15, range 15–40), but none failed. Also, none of the subjects who had baseline INSTI mutations failed and there was no accumulation of mutations. The median variation in serum creatinine was −0.01 (range +0.2 to −0.21), and only one subject had a new onset of mild proteinuria.

**Conclusions**: This dual regimen yielded excellent results in a complicate population, providing the simplest and safest salvage regimen ever.

# Sub article: P098 A retrospective analysis to evaluate and compare the efficacy of switching to Stribild fixed-dose combination in virologically suppressed HIV-1 infected adults with or without the archived NRTI resistance mutation M184V/I

**Published:** 2016-10-23

**Authors:** Vanessa Silebi

**Affiliations:** 1 Infectious Diseases, Mercy Hospital, Miami, FL, USA

## Abstract

**Introduction**: The prevalence of an isolated NRTI M184V/I resistance mutation in reverse transcriptase (RT) region in HIV-1 infected patients is as high as 60% [1–4] with limited switch options in patients harbouring this mutation, and it is recommended that these patients be switched to protease inhibitor-containing regimens. The aim of this retrospective pilot study is to validate the ability of elvitegravir/ cobicistat/ emtricitabine/ tenofovir disoproxil fumarate (Stribild) fixed-dose combination (FDC) to maintain virological suppression in patients harbouring M184V and/or M184I mutations to NRTIs over a one-year period.

**Methods**: Using data collected from a single site HIV clinic, we examined the ability of Stribild to maintain virological suppression in 30 patients harbouring M184V and/or M184I mutations compared with 29 patients in the M184V/I negative cohort. Eligible patients had documented VL <50 copies/mL prior to switch, with subsequent VL and CD4 counts collected at follow-up visits at 12, 24 and 48-week time points. All patients were randomly selected in both arms. The primary endpoint measured was the proportion of subjects in each arm with HIV-1 RNA <50 copies/mL at Week 12.

**Results**: The Resistant arm saw 90%, 96% and 88% VL suppression (defined at <50 copies/mL) over the 12, 24 and 48 week time points, respectively (Table 1). Of note three patients in the M184V/I cohort (10%) rebounded at week 12, all self-reported non-compliant; two of the three patients re-suppressed at week 24. For secondary endpoints, data trended with both cohorts benefiting while on Stribild.

**Conclusions**: Data indicate Stribild may be a reasonable option in virologically suppressed (HIV-1 RNA <50 copies/mL) HIV-1 infected adults harbouring an archived isolated M184V and/or M184I mutation. Future analysis and correlation with previous regimens and included mutations prior to switch will be analyzed. A prospective, multi-centre study to determine the variables that affect switch therapy is currently being planned.

**Table 1:** Results

Time	Mutation	%VL <50	CD4 X (SD)
Baseline	Resistant (n = 30)	100	483 (199)
	Sensitive (n = 29)	100	628 (253)
12 weeks	Resistant (n = 30)	90	489 (262)
	Sensitive (n = 25)	96	666 (286)
24 weeks	Resistant (n = 28)	96	491 (263)
	Sensitive (n = 27)	89	636 (294)
48 weeks	Resistant (n = 26)	88	502 (224)
	Sensitive (n = 24)	96	675 (269)

## References

[1] Riddler SA, Haubrich R, DiRienzo AG, Peeples L, Powderly WG, Klingman KL (2008). Class-sparing regimens for initial treatment of HIV-1 infection. N Engl J Med.

[2] Blanco JL, Montaner JS, Marconi VC, Santoro MM, Campos-Loza AE, Shafer RW (2014). Lower prevalence of drug resistance mutations at first-line virological failure to first-line therapy with atripla vs. tenofovir+emtricitabine/lamivudine+efavirenz administered on a multiple tablet therapy. AIDS.

[3] Saini S, Bhalla P, Gautam H, Baveja UK, Pasha ST, Dewan R (2012). Resistance associated mutations in HIV-1 among patients failing first-line antiretroviral therapy. J Int Assoc Physicians AIDS Care (Chic).

[4] Poon AF, Aldous JL, Mathews WC, Kitahata M, Kahn JS, Saag MS (2011). Transmitted drug resistance in the CFAR network of integrated clinical systems cohort: prevalence and effects on pre-therapy CD4 and viral load. PLoS One.

# Sub article: P099 Dolutegravir plus rilpivirine in cART-experienced subjects: an observational cohort

**Published:** 2016-10-23

**Authors:** Amedeo Ferdinando Capetti, Gaetana Sterrantino, Maria Vittoria Cossu, Giuseppe Vittorio De Socio, Simona Di Giambenedetto, Benedetto Maurizio Celesia, Barbara Argenteri, Antonio Di Biagio, GianCarlo Orofino, Giorgio Barbarini, Giuliano Rizzardini

**Affiliations:** 1 1st Division of Infectious Diseases, Luigi Sacco University Hospital, Milano, Italy; 2 Division of Infectious Diseases, Careggi Hospital, Firenze, Italy; 3 Infectious Diseases Clinic, Azienda Ospedaliero-Universitaria di Perugia, Perugia, Italy; 4 2nd Division of Infectious Diseases, Policlinico Universitario Agostino Gemelli, Roma, Italy; 5 Infectious Diseases Unit, University of Catania, ARNAS Garibaldi, Catania, Italy; 6 Infectious Diseases Clinic, San Martino Hospital, Genova, Italy; 7 1st Division of Infectious Diseases, Amedeo di Savoia Hospital, Torino, Italy; 8 2nd Division of Infectious Diseases, Policlinico San Matteo, Pavia, Italy

## Abstract

**Introduction**: Little is known on the efficacy and safety of the dual combination of rilpivirine and dolutegravir and randomized trials are ongoing. Our purpose is to describe an experience with this regimen in clinical practice.

**Methods**: All HIV-1 infected subjects treated with dolutegravir plus rilpivirine between October 2014 and September 2015 were included in an observational cohort. After ethics committees’ approval, no further enrolment was allowed. Only clinical events, demographic data, CD4 cell counts, HIV-1 RNA, serum creatinine and urinary proteins were deemed relevant for this study.

**Results**: We enrolled 135 subjects, mean age 52, 31.5% females and 10% non-Caucasians. Risk factors were balanced (32.4% drug abuse, 33.3% heterosexual and 34.2% male homosexual intercourse). Fifty-six (50.5%) had failed at least one regimen. The main reason for switch was simplification (50.5%), followed by toxicity (33.3%), need for anti-HCV therapy (n=6), persistent low-level viremia (n=4), non-adherence (n=3) and viral failure (n=1). Between week 24 and 48 one subject discontinued study drug for headache, two for drug interactions and two died, one of sepsis and one of illicit drug abuse. Sixty-two had reverse transcriptase mutations, 69 had protease mutations and one had full INSTI resistance. Seventeen had baseline viral replication (median 1932 copies/mL, range 55–971,654), 28 had <50 copies/mL and in 90 (67%) HIV-1 RNA could not be detected. At week 4, in 116 (86%) HIV-1 RNA was not detected, 16 had viral load below 50 copies/mL and three had 50 to 57 copies/mL. At week 24 one subject had viral rebound without mutations due to missed drug refill (11,030 copies/mL), 19 had <50 copies/mL and 115 had undetectable viremia. CD4+ T-cells increased by week 24 from 718 to 751/mmc. Of the 54 who have a 48-week follow-up, the abovementioned subject still had a treatment interruption, with 14,770 copies/mL, five had <50 copies/mL and 48 undetectable viremia. One subject had low-level resistance to rilpivirine, one intermediate and four high-level resistance (Stanford median score 50, range 15–70): none of them was failing at the 48-week follow-up. The median variation in serum creatinine was +0.1 (range +0.47 to −0.31); none developed proteinuria. The dual regimen was very well tolerated in this population.

**Conclusions**: A dual regimen of dolutegravir plus rilpivirine proved safe and effective in this cohort of non-naïve HIV-1-infected subjects.

# Sub article: P100 Switching to elvitegravir/cobicistat/emtricitabine/tenofovir disoproxil fumarate single-tablet regimen (Stribild^®^): effects on T-cell compartment and HIV reservoirs

**Published:** 2016-10-23

**Authors:** Esther Merlini, Federico Cazzaniga, Anna Casabianca, Chiara Orlandi, Giuseppe Ancona, Viola Cogliandro, Camilla Tincati, Teresa Bini, Mauro Magnani, Antonella d'Arminio Monforte, Giulia Marchetti

**Affiliations:** 1 Health Sciences, University of Milan, Milan, Italy; 2 Department of Biomolecular Sciences, University of Urbino “Carlo Bo”, Urbino, Italy

## Abstract

**Introduction**: EVG/c/FTC/TDF (Stribild) is non-inferior to PI/r-based regimens in cART switching patients. However, the effects of EVG/c/FTC/TDF on immune system and HIV reservoirs have not been fully investigated. We investigated the impact of 24 weeks EVG/c/FTC/TDF on T-cell compartment and HIV reservoirs in HIV-infected patients, switching from a suppressive PI-based regimen.

**Methods**: Thirty HIV+ patients on effective PI-based regimens (HIV RNA <40 copies/mL) were followed for 24 weeks after switching to EVG/c/FTC/TDF. At baseline (W0), after 12 (W12) and 24 (W24) weeks we analyzed: HLA-DR/CD38/Ki67/CCR7/CD45RA/CD127/PD1 on CD4/CD8, IFNg/IL2 production after HIV/SEB exposure (flow cytometry), total HIV DNA (THIV-DNA), low levels HIV residual viremia (LL-RV) (qPCR). Statistical analyses: Friedman, Wilcoxon signed rank and Spearman correlation tests.

**Results**: Patients were predominantly male (70%); median agewas 44; median HIV infection duration was 8 years; median time of HIV RNA suppression and cART duration was 5 and 6 years, respectively. At baseline, all patients were receiving TDF+FTC (for a median of 5 years) in association with either DVR/r (47%) or ATV/r (53%). No HCV/HBV co-infections were found. Upon EVG/c/FTC/TDF introduction no changes in CD4 (634 cells/mm^3^ vs. 614 cells/mm^3^ vs. 582 cells/mm^3^; p=0.465) and in LL-RV (0 copies/mL vs. 0 copies/mL vs. 0 copies/mL; p=0.081) were shown. Table 1 lists viro-immunologic results. Following EVG/c/FTC/TDF switch, we observed a significant reduction in HLA-DR+CD38+CD4+ (p=0.016) and HLA-DR+CD38+CD8+ (p=0.048). Interestingly, SEB exposure resulted in a significant reduction of IFN-g/IL-2-producing CD4+ (p=0.024) and CD8+ (p=0.003), IFNg-producing CD8+ (p=0.024) and IL-2-producing CD4+ (p=0.0001). No major differences were found after HIV stimulation. We failed to find any differences in T-cell exhaustion, proliferation and maturation, with the exception of a decrease in central memory CD4+ (p=0.030). Similarly, no changes in THIV-DNA were found. At T0, LL-RV positively correlates with THIV-DNA (r=0.60, p=0.005) and HLA-DR+CD38+CD4+ (r=0.45, p=0.022). Besides, THIV-DNA positively correlates with IFNg+IL2+CD4+ and inversely with IFNg+CD8+ (r=0.51, p=0.019; r=−0.45, p=0.039, respectively) after SEB challenge. Interestingly, at W24 only the positive correlation between LL-RV and THIV-DNA (r=0.58, p=0.030) persists.

**Conclusions**: In HIV+ virologically suppressed patients, 24 weeks of EVG/c/FTC/TDF resulted in substantial reduction of activated T-lymphocytes and *ex-vivo* T-lymphocyte susceptibility to exogenous super-stimulation. Despite failing to detect changes in HIV reservoirs following EVG/c/FTC/TDF, we capture an association between HIV DNA and both residual viremia and IFNg/IL2-producing T cells, suggesting poly-functional T-cell recruitment as a response to ongoing viral challenge. Altogether, these data would propose a favourable effect of EVG/c/FTC/TDF to preserve immune-activation-driven damage to T-cell homeostasis, in turn possibly containing cell-associated HIV viral burden in already virologically suppressed patients.

**Abstract P100–Table 1:** Immune phenotypes and HIV reservoir in a cohort of HIV-infected patients switching to Stribild

	Week 0	Week 12	Week 24	p		Week 0	Week 12	Week 24	p
T-cell activation					T-cell proliferation				
HLA-DR + CD38 + CD4+, % (IQR)	2.28 (1.44–3.78)	1.46 (1–3.18)	1.26 (0.8–2.63)	0.016	Ki67 + CD4+, % (IQR)	2.12 (1.59–3.74)	2.44 (1.84–4.65)	3.34 (0.87–5.21)	0.846
HLA-DR + CD38 + CD8+, % (IQR)	5.32 (3.23–11.36)	4.54 (2.35–6.63)	4.64 (2.98–6.85)	0.048	Ki67 + CD8+, % (IQR)	1.83 (1.22–2.22)	1.91 (1.17–2.98)	1.81 (0.77–4.65)	0.747
HLA-DR + CD4+, % (IQR)	9.4 (7.47–17.22)	9 (6.22–14.62)	8.15 (5.3–11.9)	0.006	SEB stimulation responses				
CD38 + CD8+, % (IQR)	24.02 (15.3–34.05)	16.73 (11.7–31.2)	14.9 (11.2–22.8)	0.018	CD4 + IFNg+, % (IQR)	0.18 (0–1.46)	0.34 (0–0.87)	0.46 (0.07–0.86)	0.497
T-cell exhaustion					CD4 + IFNg + IL-2+, % (IQR)	0.21 (0.02–0.64)	0.14 (0.01–0.79)	0.03 (0–0.07)	0.024
PD-1 + CD4+, % (IQR)	3.84 (2.25–5.35)	2.84 (1.47–6.01)	2.79 (2.04–3.68)	0.289	CD4 + IL-2+, % (IQR)	1.55 (0.50–3.22)	0.26 (0–2.03)	0.2 (0–0.47)	0.0001
PD-1 + CD8+, % (IQR)	4.15 (1.48–6.36)	3.16 (1.6–5.76)	3.29 (1.62–3.64)	0.339	CD8 + IFNg+, % (IQR)	2.01 (0.48–4.39)	2.79 (0.95–3.98)	1.15 (0–3.45)	0.024
T-cell homeostasis					CD8 + IFNg + IL-2+, % (IQR)	0.13 (0.04–0.26)	0.09 (0.01–0.45)	0 (0–0.05)	0.003
CD127 + CD4+, % (IQR)	26.2 (20.5–35.9)	21.4 (10.2–33.1)	28.6 (20.1–34.5)	0.129					
CD127 + CD8+, % (IQR)	18.6 (15.6–25.3)	21.8 (11.3–27.1)	23.3 (17.9–27.9)	0.072	HIV stimulation responses				
NAIVE + CD4+, % (IQR)	5.43 (2.3–7.28)	4.52 (1.49–6.49)	1.72 (0.53–6.92)	0.256	CD4 + IFNg+, % (IQR)	0 (0–0.06)	0 (0–0.06)	0.03 (0–0.09)	0.001
CM + CD4+, % (IQR)	3.26 (2.25–6.40)	3.21 (2.09–5.08)	2.09 (0.32–6.65)	0.030	CD4 + IFNg + IL-2+, % (IQR)	0 (0–0.001)	0 (0–0.02)	0 (0–0.06)	0.062
EM + CD4+, % (IQR)	53.5 (44.2–67.7)	53.5 (41–63)	54.7 (45.2–61.1)	0.482	CD4 + IL-2+, % (IQR)	0 (0–0.11)	0 (0–0)	0 (0–0.03)	0.089
TD + CD4+, % (IQR)	32.2 (15.4–40)	34.75 (24.4–44.4)	39.6 (24.3–49.2)	0.405	CD8 + IFNg+, % (IQR)	0 (0–0.39)	0 (0–0.26)	0 (0–0.04)	0.828
NAIVE + CD8+, % (IQR)	32.2 (15.4–40)	13.2 (3.03–21.4)	7.98 (4.54–18.9)	0.657	CD8 + IFNg + IL-2+, % (IQR)	0 (0–0.1)	0 (0–0)	0 (0–0.01)	0.267
CM + CD8+, % (IQR)	0.84 (0.31–3.01)	2.01 (0.64–4.41)	1.47 (0.54–2.64)	0.482					
EM + CD8+, % (IQR)	33.2 (22.3–43.8)	33.4 (25.6–47.2)	37.1 (27.5–43.5)	0.857	HIV reservoir				
TD + CD8+, % (IQR)	52.6 (34.7–60.3)	46.2 (29.4–61.0)	47.4 (35.3–58.6)	0.962	tot HIV DNA, copies/10*4 CD4 (IQR)	3 (2–13)	5 (3–12)	4 (2–7)	0.223

CM, central memory CCR7 + CD45RA-; EM, effector memory CCR7-CD45RA-; IQR, interquartile range; SEB, Staphylococcal enterotoxin B; TD, terminally differentiated CCR7-CD45RA+.

Note: Data are presented as median (IQR). Statistical analyses: Friedman test with Dunn's multiple comparison test.

# Sub article: P101 Reducing pill burden is more durable than reducing drug burden as strategy of HIV treatment simplification

**Published:** 2016-10-23

**Authors:** Mauro Zaccarelli, Patrizia Lorenzini, Nicola Gianotti, Stefano Rusconi, Pierluigi Viale, Antonio Chirianni, Laura Sighinolfi, Giustino Parruti, Antonella D'Arminio Monforte, Andrea Antinori, Study Group on behalf of Icona Foundation

**Affiliations:** 1 Clinical Department, National Institute for Infectious Diseases Lazzaro Spallanzani, Rome, Italy; 2 Department of Infectious Diseases, San Raffaele Scientific Institute, Università Vita-Salute San Raffaele, Milan, Italy; 3 Dipartimento di Scienze Biomediche e Cliniche ‘Luigi Sacco’, Division of Infectious and Tropical Diseases, University of Milan, Milan, Italy; 4 Infectious Diseases Unit, Department of Medical and Surgical Sciences, Sant'Orsola Hospital, University of Bologna, Bologna, Italy; 5 3rd Department of Infectious Diseases, D. Cotugno Hospital-AORN dei Colli, Naples, Italy; 6 Department of Infectious Diseases, S. Anna Hospital, Ferrara, Italy; 7 Infectious Diseases Unit, Pescara General Hospital, Pescara, Italy; 8 Clinic of Infectious Diseases, Department of Health Sciences, ASST Santi Paolo e Carlo, University of Milan, Milan, Italy

## Abstract

**Introduction**: Reducing antiretroviral toxicity and improving adherence involves different switching strategies as reducing pill burden by single-tablet regimen (STR) or drug burden by regimens with two or one drug (less drug regimen, LDR). We aimed to compare the two approaches in patients who switched with suppressed viremia.

**Materials and methods**: From the Italian ICONA cohort, patients with undetectable HIV RNA switching to STR or LDR from any triple regimen were selected. Drug discontinuation by any cause (DAC) was the end-point of the analysis assessed by incidence rates and Poisson regression.

**Results**: Overall, 842 patients (525 STR, 317 LDR) were analyzed. STR included TDF/FTC/EFV (36.8%), TDF/FTC/RPV (48.4%) and EVG/COBI/FTC/TDF (14.9%). LDR included dual regimens: LPV/r, ATV/r, DRV/r plus 3TC (29.7%) or any MVC, RAL, ETV (15.7%) and PI/r monotherapy (54.6%). Patients switching to STR were more frequently receiving NNRTI, changed from first line of therapy and from NNRTI or INSTI, changed without failure or toxicity, had higher haemoglobin and transaminase. In contrast, patients switching to LDR were more often on PI/r, changed for toxicity, were older, had longer history of HIV infection and regimens, higher triglycerides and creatinine (Table 1). Overall, 240 patients (107/525 STR, 133/317 LDR) discontinued therapy in 1525 patient-years of follow-up (PYFU). The crude IR of DAC was 15.7/100 PYFU (95% CI 13.9–17.9): 10.8/100 PYFU (95% CI 8.9–13.0) in STR and 24.9 (95% CI 21.0–29.6) in LDR (p<0.001). Among causes of discontinuation, toxicity was significantly higher in STR patients (57.0% vs. 28.6%, p<0.001), while in LDR discontinuation was associated with different situations: physician decision, switch to other regimen and intensification. No difference was found for discontinuation by viral failure (STR 4.7% vs. LDR 3.0%). By multivariable Poisson regression (Table 2), HCV co-infection, higher creatinine and switching from PI/r were associated with higher risk of DAC; longer duration of HIV, MSM, being at second switch versus first and change without failure or toxicity were associated with lower risk. Switching to STR was associated with significant 50% reduction of DAC (IRR 0.53; 95% CI 0.39–0.71) as compared with switching to LDR. Risk of DAC did not differ among the three STR, while, among LDR, probability of DAC was higher in mono than dual regimens (IRR 1.89; 95% CI 1.33–2.69). Excluding monotherapy, rates of DAC remained significantly lower in STR.

**Conclusions**: Switching to STR was associated with greater stability of the regimen and consequently lower treatment discontinuation. LDR can be useful in limited settings in order to reduce NRTI toxicity.

**Abstract P101–Table 1:** Characteristics and differences of patients switching to STR and LDR

	LDR	STR	p
N	317	525	
Male gender, n (%)	246 (77.6)	418 (79.6)	0.487
Age, years, median (IQR)	46 (39–52)	43 (36–50)	<0.001
Years from HIV test and first visit, median (IQR)	5.8 (2.8–15.3)	4.8 (2.4–9.8)	0.002
Mode of HIV transmission, n (%)			
Heterosexual	119 (37.5)	201 (38.3)	0.064
IVDU	51 (16.1)	54 (10.3)	0.114
MSM	134 (42.3)	238 (45.3)	0.391
Other/unknown	13 (4.1)	32 (6.1)	0.341
HCV co-infection, n (%)			
Positive	61 (19.2)	74 (14.1)	0.106
Negative	240 (75.7)	429 (81.7)	0.486
Not known	16 (5.1)	22 (4.2)	0.609
HBV co-infection, n (%)			
Positive	10 (3.1)	17 (3.2)	0.970
Negative	282 (89.0)	469 (89.3)	0.909
Not known	25 (7.9)	39 (7.4)	0.894
Number of regimens at switch, n (%)			
1	139 (43.8)	315 (60.0)	<0.001
2	71 (22.4)	100 (19.1)	0.797
3 or more	107 (33.7)	110 (20.9)	<0.001
Months of undetectable HIV RNA pre-switch, median (IQR)	25.4 (12–52)	23.2 (8–54)	0.130
Overall years of cART, median (IQR)	3 (2–8)	3 (1–6)	0.145
Laboratory data at switch, median (IQR)			
Haemoglobin, mg/dL	14.6 (13.5–15.5)	14.8 (13.8–15.6)	0.026
White blood cells/mm^3^	6300 (5100–7680)	5990 (5000–7300)	0.088
Triglicerides, mg/dL	133 (94–198)	121 (85–169)	0.004
Cholesterol, mg/dL	193 (165–221)	188 (161–217)	0.255
Creatinine, mg/dL	0.95 (0.80–1.13)	0.89 (0.78–1.00)	<0.001
ALT, mg/dL	23 (16–33)	28 (20–40)	<0.001
CD4, cells/mm^3^	598 (464–807)	606 (463–805)	0.734
Type of pre-switch regimen, n (%)			
NRTI/NNRTI	44 (13.9)	197 (37.5)	<0.001
NRTI/PI/r	265 (83.6)	296 (56.4)	<0.001
NRTI/INSTI	8 (2.5)	32 (6.1)	0.019
Reason for switching, n (%)			
Toxicity	106 (33.4)	104 (19.8)	<0.001
Adherence/patient's decision	8 (2.5)	15 (2.9)	0.831
Neither failure nor toxicity	191 (60.2)	384 (73.1)	0.005
Other/unknown	12 (3.8)	22 (4.2)	0.896

**Abstract P101–Table 2:** Rate ratio of DAC from fitting a Poisson regression

	RR	95% CI low	95% CI high	p
Age (per 10 years older)	1.12	1.12	0.98	0.105
Years from first HIV test and visit (each)	0.97	0.94	0.99	0.007
Mode of HIV transmission				
Heterosexual	1.00			
IVDU	0.77	0.46	1.29	0.321
MSM	0.71	0.53	0.96	0.026
Other/unknown	0.81	0.45	1.45	0.468
HCV co-infection				
Negative	1.00			
Positive	1.99	1.27	3.13	0.003
Unknown	0.81	0.56	1.87	0.947
Number of regimens at switch				
1	1.00			
2	0.59	0.41	0.85	0.005
3 or more	0.81	0.64	1.29	0.586
Laboratory tests				
Triglycerides (per 10 mg/dL)	1.00	0.99	1.01	0.823
Creatinine (per 10 mg/dL)	1.30	1.01	1.67	0.041
ALT (per 10 UI/mL more)	1.01	0.99	1.03	0.168
Type of pre-switch regimen				
NRTI/NNRTI	1.00			
NRTI/PI/r	1.60	1.14	2.23	0.006
NRTI/INSTI	2.09	0.98	4.45	0.055
Reason for switching				
Toxicity	1.00			
Adherence/patient's decision	1.11	0.50	2.47	0.801
Neither failure nor toxicity	0.65	0.48	0.87	0.005
Other/unknown	1.81	0.99	3.31	0.053
STR versus LDR	0.53	0.39	0.71	<0.001

# Sub article: P102 Efficacy and safety of switch to DRV/cobicistat in patients who are virologically suppressed with treatment regimens containing DRV/r

**Published:** 2016-10-23

**Authors:** Sara de la Fuente Moral, Alberto Diaz de Santiago, Carlos Folguera, Carmen De Mendoza Fernandez, Alfonso Angel-Moreno Maroto

**Affiliations:** 1 Internal Medicine, Hospital Puerta de Hierro Majadahonda-Madrid, Madrid, Spain; 2 Pharmacy Service, Hospital Puerta de Hierro Majadahonda-Madrid, Madrid, Spain

## Abstract

**Introduction**: DRV boosted with ritonavir (r) is part of preferred antiretroviral therapies. Boosting DRV with cobicistat (cobi), a new selective inhibitor of cytochrome P450, allows coformulation in a single tablet, recently approved based on favourable bioequivalence data [1]. Cobicistat inhibits the tubular secretion of creatinine, leading to mild increase of serum creatinine levels, with no effect on actual glomerular filtration rate [2]. However, safety and efficacy data about DRV/r in clinical practice are still scarce. The aim of this study was to evaluate the efficacy, safety and tolerability of switching to DRV/cobi in HIV-infected patients who are virologically suppressed on a stable regimen containing ritonavir-boosted DRV.

**Materials and methods**: Retrospective study of patients following the switch from DRV/r to DRV/cobi. Other components of the regimen remained unchanged. Eligibility criteria included HIV-1 RNA below 100 copies/mL at time of treatment switch. Patients with detectable viraemia on ART, naïve patients and patients who started treatment with DRV/cobi from treatment schemes not containing DRV/r were excluded. The primary endpoint was to determine percentage of patients who remained virologically suppressed after switch. Secondary outcomes included changes in renal function and lipid profile. Clinical data were collected from patients’ medical records.

**Results**: We analyzed 150 virologically suppressed patients switching from DRV/r to DRV/cobi from July 2015 to May 2016. Baseline features are shown in Table 1. Out of 150 patients, 15 (10%) did not continue on care, so data from 135 patients were analyzed. Mean time to control analysis was 4.16 months (0.2–6.5). Most patients remained suppressed after changing treatment. There were four (3%) patients with virological failure; three of them due to poor treatment compliance, and one in the setting of chemotherapy treatment for lymphoma. Creatinine levels were slightly higher after switching to DRV/cobi, with no statistically significant differences neither in patients with or without concomitant treatment with tenofovir (Table 2 and Figure 1). Total cholesterol, LDL and HDL cholesterol remained unchanged, while a statistically significant decrease of 14 mg/dL was observed in the triglyceride level (Figure 2).

**Conclusions**: In HIV-1-infected patients, who are virologically suppressed, switching from RTV to cobicistat, cobi-boosting DRV was effective maintaining virological suppression and well tolerated. Mild and non-progressive increase in serum creatinine was confirmed. Cobicistat does not have clinically relevant effect on lipid profile.

**Abstract P102–Figure 1:** Serum creatinine levels before and after switch to DRV/c.

**Abstract P102–Figure 2:** Lipid profile before and after switch to DRV/c.

**Abstract P102–Table 1:** Patient characteristics

	Patients (n = 135) (lost 15, 10%)
Male gender, n (%)	107 (71.3)
Age, year, mean ± SD	51.1±9.5
ART regimen, n (%)	
Triple therapy	73 (54.1)
Dual therapy	43 (31.8)
Monotherapy	19 (14.1)
Risk group, n (%)	
HSH	56 (41.5)
HTX	31 (22.9)
IDU	45 (33.4)
Transfusional	3 (2.2)
TDF, n (%)	31 (23)

**Abstract P102–Table 2:** Serum creatinine levels and lipid profile before and after switch to DRV/c

	DRV/r	DRV/c	p
Creatinine (all)	0.93±0.05	1.01±0.05	1
On TDF	0.89±0.02	0.98±0.03	1
Not TDF	0.94±0.06	1.02±0.07	1
Total cholesterol	195.4±3.8	196.7±3.9	0.6
LDL cholesterol	109.2±3.2	115.7±3.4	0.9
HDL cholesterol	51.1±1.3	50.5±1.25	0.3
Triglycerides	177.1±11.6	162.8±13.9	0.03

## Reference

1. Kakuda TN, Opsomer M, Timmers M, Iterbeke K, Van De Casteele T, Hillewaert V, et al. Pharmacokinetics of darunavir in fixed-dose combination with cobicistat compared with coadministration of darunavir and ritonavir as single agents in healthy volunteers. J Clin Pharmacol. 2014;54:949–57. doi: http://dxdoi.org/10.1002/jcph.290

2. Temesgen Z. Cobicistat, a pharmacoenhancer for HIV treatments. Drugs Today (Barc). 2013;49:233–7. doi: http://dxdoi.org/10.1358/dot.2013.49.4.1947288

# Sub article: P103 Confirmed efficacy and safety of dual therapy based in lamivudine plus a ritonavir-boosted protease inhibitor in the clinical setting

**Published:** 2016-10-23

**Authors:** María Fontecha-Ortega, Vanessa Muñoz-Mendoza, Cristina Gómez, Matilde Sánchez-Conde, Miguel Angel Rodríguez, Maria Jesús Vivancos, Jose Luis Casado

**Affiliations:** 1 Internal Medicine, Hospital de Getafe, Getafe, Spain; 2 Internal Medicine, Hospital Obispo Polanco, Teruel, Spain; 3 Infectious Diseases, Hospital Ramón y Cajal, Madrid, Spain

## Abstract

**Introduction**: The aim of this study was to evaluate the efficacy, safety and additional benefits of a dual regimen with lamivudine (3TC) + protease inhibitor (PI) boosted with ritonavir in the clinical setting.

**Materials and methods**: Prospective study of 99 HIV-infected patients, HBV negative, without resistance to lamivudine or PI, who switched to this dual therapy because toxicity or intolerance. Routine laboratory tests, including estimated glomerular filtration rate (eGFR CKD-EPI equation), lipid parameters, immunovirological evaluation and a complete urinary determination were performed at inclusion and during follow-up.

**Results**: The mean age was 49.8 years (35–74), and 66% were male. The median time of HIV infection was 20.6 years (15.8–24.4), nadir CD4+ count was 193 cells/mm^3^ (IQR 90–306) and 42% had a previous AIDS diagnosis. Overall, patients were pre-treated with a mean of 6 regimens (1–10) for a median of 40.5 months. At the time of switch, 92% had an HIV RNA level <50 copies/L and the median CD4+ count was 555 cells/microL (IQR 394–799). Causes of switch were toxicity/intolerance in 71%, simplification in 24% and lack of adherence leading to detectable HIV load in 4%. Renal toxicity was observed in 50% of cases, followed by lipodystrophy in 10%. The main combination used was 3TC+darunavir/r (n=70) and 3TC+lopinavir/r in 21 cases. At 48 weeks, the efficacy by intention to treat was 97% (96/99), with three virological failures attributed to non-adherence. The median increase in CD4 count was +35 and +80 cells/mL at 6 and 12 months. After switching, a significant increase in total cholesterol (TC), LDL cholesterol and triglycerides was observed during the first 6 months (p=0.001, p=0.05 and p=0.07, respectively), with partial recovery at 12 months, and it was more marked in 64 patients previously receiving tenofovir disoproxil fumarate (TDF). On the contrary, there was a significant improvement in the eGFR at 6 and 12 months (p=0.03 and p=0.01), and urinary parameters improved significantly.

**Conclusions**: In this study, in the clinical setting, we demonstrate the efficacy and safety of dual therapy with 3TC associated with a boosted PI, with 97% remaining free of virological failure after 48 weeks and improvement in renal involvement. By contrast, it is expected a significant increase in total cholesterol and LDL cholesterol initially, with partial recovery at 48 weeks.

# Sub article: P104 Preferred regimens and reasons for switches in HIV-positive individuals in the German HIV-HEART cohort study over 30 years of antiretroviral treatment evolution

**Published:** 2016-10-23

**Authors:** Volker Holzendorf, Stefan Esser, on behalf of the HIVHEART Study Group

**Affiliations:** 1 Clinical Trial Centre Leipzig – KKS, University of Leipzig, Leipzig, Germany; 2 Clinic of Dermatology and Venerology, University Hospital Essen, Essen, Germany

## Abstract

**Introduction**: ART changes over the time and yields benefits in terms of virological suppression, tolerability and regimen simplification also in the contexts of ageing and polypharmacy. Compared with other countries all ART regimens are assumed by German health insurance coverage because German guidelines recommend personalized ART. We analyze the non-restrictive treatment choices and reasons for switches of the German clinicians in the HIV-HEART cohort.

**Methods**: The HIV-HEART study is an ongoing, prospective and observational cohort study in the German Ruhr area to assess the frequency and clinical course of cardiac disorders in 1538 HIV-positive individuals (HIV+). From the first diagnosis of HIV infection until 31 December 2015 ART, medical history and reasons for switches of ART were collected based on the health records, medication plans and the anamnesis. ART history was divided into five chronologic 5-year time periods (–1995, 1996–2000, 2001–2005, 2005–2010, 2011–2015), which were compared with each other

**Results**: One thousand five hundred and thirty-eight HIV+ (mean age: 49.9±11.0 years; male: 84.4%; Caucasian: 88.3%; MSM: 52.2%) were included at their last study visit. Sixteen thousand eight hundred and eighty patient-years since the first HIV diagnosis of the HIV+ were reviewed. According to the CDC classification of the HIV infection, HIV+ were distributed over the clinical categories (A: 32.3%; B: 29.2%; C: 30.5%; n.k.: 6.2%) while almost the half had an advanced immunodeficiency (I: 7.8%; II: 39.2%; III: 46.6%; n.k.: 6.2%). HIV+ were treated with ART on average for 10.2±5.8 years with mean 3.8±3.2 different regimens over the time. 90.9% of the living HIV+ had an HIV RNA below the level of detection at their last visit. Since the beginning of the HAART era the number of switches including the initiation of the ART in naïve HIV+ per 5-year time period decreased from 2.7 to 1.4. The catched main reasons (n>20) for ART switch changed: 1996 to 2001: adverse events 44.1%, virological failure 24.9% and compliance 21.1%; 2011 to 2015: adverse events 55.6%, patients request 19.3%, virological failure 9.1% and drug interactions 7.6%. In 1995, 87% of HIV+ were treated only with NRTIs; in 2001, 53% with a PI-containing regimen; in 2010, 36% with an NNRTI regimen; and in 2015, 36% take an INSTI-containing regimen.

**Conclusions**: Using new treatment options clinicians try to optimize the ART regimens over the time. More than 90% of the HIV+ achieved a viral load below the level of detection. Tolerability was still the most important reason for ART switches. Currently INSTI-containing regimens were preferred.

# Sub article: P105 Drug concentrations, adherence, patient-reported symptoms and health-related quality of life in HIV-infected, virologically controlled patients switching to maraviroc+darunavir/ritonavir or continuing the previous triple therapy: sub-studies from the randomized GUSTA trial

**Published:** 2016-10-23

**Authors:** Barbara Rossetti, Roberta Gagliardini, Lucia Lisi, Melissa Masini, Silvia Lamonica, Francesca Vignale, Gaetana Sterrantino, Giancarlo Orofino, Manuela Colafigli, Alessandra Latini, Andrea Tosti, Stefano Rusconi, Michele Trezzi, Alessandro D'Avino, Pierfrancesco Grima, Ivano Mezzaroma, Antonio Di Biagio, Pierluigi Navarra, Simona Di Giambenedetto, Andrea De Luca

**Affiliations:** 1 Infectious Diseases Clinic, Azienda Ospedaliera Universitaria Senese, Siena, Italy; 2 Infectious Diseases Clinic, Catholic University of Sacred Heart, Rome, Italy; 3 Pharmacology, Catholic University of Sacred Heart, Rome, Italy; 4 Infectious Diseases Clinic, University G. D'Annunzio, Chieti, Italy; 5 Tropical and Infectious Diseases Clinic, Azienda Ospedaliero Universitaria Careggi, Florence, Italy; 6 Infectious Diseases Unit, Amedeo di Savoia Hospital, Turin, Italy; 7 Infectious Dermatology and Allergology, IFO San Gallicano, Rome, Italy; 8 Infectious Diseases Clinic, University of Perugia, Perugia, Italy; 9 Infectious Diseases Clinic, DIBIC Luigi Sacco, University of Milan, Milan, Italy; 10 Infectious Diseases Unit, San Jacopo Hospital, Pistoia, Italy; 1 Infectious Diseases Unit, Santa Caterina Hospital, Galatina, Italy; 2 Infectious Diseases Clinic, Sapienza University, Rome, Italy; 3 Infectious Diseases Clinic, IRCCS Azienda Ospedaliera Universitaria San Martino-IST, Genova, Italy

## Abstract

**Introduction**: The randomized GUSTA trial, comparing switch to maraviroc (MVC) 300 mg + darunavir/ritonavir (DRV/RTV) 800/100 mg OAD (switch arm, S) versus continuation of the previous three-drug, virologically suppressive regimen (continuation arm, C) in patients carrying R5 virus was prematurely discontinued due to an excess of virologic failures (VF) in arm S. Aims of the sub-studies were to analyze drug levels in arm S, compare adherence, patient-reported symptoms and health-related quality of life (QoL) between arms and analyze the correlation between these parameters and treatment outcome.

**Materials and methods**: Plasma drug concentrations were measured between week 4 and 96 by a validated UPLC-MS/MS and Ctrough was calculated. In both arms, self-reported adherence (VAS 0–100% weeks 0, 4, 24 and 48), patient-reported symptoms and physical/mental QoL scores (VAS 0–100% weeks 0 and 48) were collected using validated questionnaires.

**Results**: Hundred and fourteen patients were analyzed (62 S, 52 C): 23% females, 40% heterosexuals, median age 49 years, baseline CD4 711 cells/µL, on ART since 10 years. Median Ctrough for DRV (n=292 samples) was 1333 ng/mL (IQR 777–1686), MVC (n=257) 55 ng/mL (41–106), RTV (n=285) 37 ng/mL (21–56). In nine patients with VF drug Ctrough at VF were significantly lower as compared with those during treatment success: DRV 1251 ng/mL (0–1864) versus 1328 ng/mL (787–1678), p=0.004, MVC 39 ng/mL (5–207) versus 55 ng/mL (45–104), p=0.001, and RTV 4 ng/mL (0–143) versus 42 ng/mL (21–56), p<0.001. By linear regression, DRV Ctrough associated negatively with anti-HCV+ status (mean −586 ng/mL; p=0.041) and eGFR (+10 mL/min: −8 ng/mL; p=0.011), and positively with age (+10 years: +249 ng/mL; p=0.003). Adherence, patient-reported symptoms, physical health or mental health-related QoL scores did not differ between arms (Table 1). Physical health declined significantly from baseline at 48 weeks in arm C but not in S (Table 1). Adherence was significantly lower in patients with VF (arm S) and treatment failure (arm C) (Table 1). At time points with self-reported adherence ≤80% 6/51 (11.8%) in arm S versus 0/35 (0%) in arm C showed VF (χ^2^ p=0.07). Values indicate means (standard deviations). The symptoms score reports the sum of the values of the intensity of the symptom (between 1 (absent) and 5 (very much)) divided by the number of evaluable symptoms per patient (max 30 total symptoms; ISS QoL adapted). p-value of between-arm comparisons (week 48 vs. baseline; success vs. failure); in arm S all failures were virologic; in arm C all failures were due to treatment discontinuation.

**Conclusions**: Switch to MVC+DRV/RTV OAD as compared with continuing a previous triple ART does not determine modifications in adherence, patient-reported symptoms and QoL. The excess of VF with this regimen seems associated with a lower forgiveness, not allowing to maintain virologic suppression during periods of suboptimal adherence with reduced drug exposure. Candidates to this regimen need to be carefully selected and instructed.

**Abstract P105–Table 1:** Adherence, health-related QoL and patient-reported symptoms at different time points, based on randomization arm

	Arm S (MVC + DRV/RTV)	Arm S (MVC + DRV/RTV)	Arm S (MVC + DRV/RTV)	Arm C (continuation of previous three-drug ART)	Arm C (continuation of previous three-drug ART)	Arm C (continuation of previous three-drug ART)	Between-arm comparisons	Between-arm comparisons
	Baseline (n = 34)	Week 48 (n = 34)	p	Baseline (n = 27)	Week 48 (n = 27)	p	p (baseline values)	p (week 48 values)
Self-reported adherence (VAS 0–100%)	87.35% (16.57)	87.35% (11.36)	1.00	88.88% (13.95)	89.63% (14.00)	0.80	0.70	0.48
	Baseline (n = 30)	Week 48 (n = 30)		Baseline (n = 22)	Week 48 (n = 22)			
Patient-reported symptoms score	1.39 (0.31)	1.44 (0.38)	0.21	1.41 (0.31)	1.36 (0.27)	0.35	0.80	0.42
Mental health (VAS 0–100%)	62.07% (25.54)	63.33% (22.48)	0.55	57.95% (22.34)	56.82% (25.79)	0.83	0.55	0.33
Physical health (VAS 0–100%)	65.18% (20.79)	58.33% (20.05)	0.26	63.64% (16.67)	53.64% (26.60)	0.04	0.78	0.47
	At failure (n = 9)	During treatment success (n = 129)		At failure (n = 12)	During treatment success (n = 108)			

Self-reported adherence (VAS 0–100%)	73.32% (20.00)	88.21% (12.52)	0.001	80.83% (17.82)	89.23% (13.74)	0.05		

# Sub article: P106 A comparison between tenofovir/emtricitabine/elvitegravir/cobicistat and dolutegravir-based three-drug regimens as switch strategies for virologically controlled, HIV-infected patients

**Published:** 2016-10-23

**Authors:** Gianmaria Baldin, Alberto Borghetti, Amedeo Capetti, Gaetana Sterrantino, Stefano Rusconi, Alessandra Latini, Andrea Giacometti, Maria Vittoria Cossu, Manuela Colafigli, Mariangela Micale, Sara Veloci, Francesca Lombardi, Ramona De Marco, Arianna Emiliozzi, Andrea De Luca, Simona Di Giambenedetto, Giordano Madeddu

**Affiliations:** 1 Institute of Clinical Infectious Diseases, Catholic University of Sacred Heart, Policlinico Gemelli, Rome, Italy; 2 Division of Infectious Diseases, Department of Inf, Luigi Sacco University Hospital, Milan, Italy; 3 Division of Infectious Diseases, Careggi Hospital, Florence, Italy; 4 Infectious Diseases Unit, DIBIC Luigi Sacco, University of Milan, Milan, Italy; 5 Infectious Dermatology and Allergology Unit, IFO San Gallicano Institute (IRCCS), Rome, Italy; 6 Institute of Infectious Diseases and Public Health, Università Politecnica delle Marche, Ancone, Italy; 7 Division of Infectious Diseases, Department of Inf, Luigi Sacco University Hospital, Rome, Italy; 8 University Division of Infectious Diseases, Siena University Hospital, Siena, Italy; 9 Department of Clinical and Experimental Medicine, University of Sassari, Sassari, Italy

## Abstract

**Introduction**: In cART, dolutegravir and elvitegravir are recommended first-line drugs for naïve, HIV-infected patients. Concerning their use in treatment-experienced, virologically controlled patients, a comparison of the efficacy and safety of dolutegravir- and elvitegravir-based cART is lacking.

**Materials and methods**: The primary study endpoint was to evaluate rates of virologic failure (VF, one HIV RNA >1000 copies/mL or two consecutive HIV RNA >50 copies/mL) and treatment failure (TF, discontinuation of elvitegravir or dolutegravir for any cause) in a multi-centre cohort of virologically controlled, HIV-infected patients switching to dolutegravir (plus either tenofovir/emtricitabine or abacavir/lamivudine) or to tenofovir/emtricitabine/elvitegravir/cobicistat. Predictors of TF were analyzed by Cox regression. Changes in metabolic, liver and renal functions at week 24 and 48 in each group were also evaluated.

**Results**: Two hundred and forty patients were eligible: 67 and 173 started elvitegravir and dolutegravir, respectively. Study arms did not differ for baseline characteristics except for nadir CD4 count, previous AIDS events, reasons for switching to the new regimen (see Table 1). In the tenofovir/emtricitabine/elvitegravir/cobicistat arm, VF was detected in one case over 54.5 patient-years follow-up (PYFU), whereas in the dolutegravir study arm no VF was documented over 90.8 PYFU. Conversely, 16 TF (17.6 per 100 PYFU) occurred in the dolutegravir arm, whereas four TF (7.3 per 100 PYFU) occurred with elvitegravir. Survival analysis revealed a 81.0% and a 96.4% probability of remaining with dolutegravir and elvitegravir at week 48, respectively (log-rank: 0.014). Causes of TF in the tenofovir/emtricitabine/elvitegravir/cobicistat arm were: neurologic events (two), renal toxicity (one) and unspecified reason (one). Causes of TF in the dolutegravir arm were: hypersensitivity reactions (two), gastro-intestinal (one), liver (three), renal (one) and neurologic (six) toxicities, drug interaction (one) and unspecified reasons (two). In a multivariate model, anti-HCV positive serostatus was predictive of TF (aHR 2.76, 95% CI 1.06–7.19; p=0.038), after adjusting for cART (aHR dolutegravir vs. elvitegravir 4.37, 95% CI 0.97–19.66; p=0.055), male sex (aHR vs. female 0.40, 0.16–1.01; p=0.052) and years of antiretroviral therapy (p=ns). A change in total cholesterol levels (−22 mg/dL; p=0.023) was evident in the dolutegravir but not in the elvitegravir arm (+19 mg/dL; p=0.069) at week 48. Triglycerides decreased significantly in both study arms at week 48. As expected, a decrease in eGFR was seen with tenofovir/emtricitabine/elvitegravir/cobicistat (−15 mL/min; p<0.001).

**Conclusions**: High virologic efficacy of elvitegravir and dolutegravir-based cART was confirmed in our cohort. Despite a better metabolic profile, more TFs were detected with dolutegravir than with elvitegravir, which prompts the need for further investigation.

**Abstract P106–Table 1:** Characteristics of study population at baseline

Variables	Overall n=240	Elvitegravir n=67	Dolutegravir n=173	p
Age^a^	51 (4–56)	50 (40–54)	51 (46–57)	0.082
Male sex	178 (74.2)	54 (80.6)	124 (71.7)	0.157
Risk factor for HIV				
Heterosexual	98 (40.8)	26 (38.8)	72 (41.6)	0.074
MSM	67 (27.9)	25 (37.3)	42 (24.3)	
IDU	59 (24.6)	15 (22.4)	44 (25.4)	
Other/unknown	16 (6.7)	1 (1.5)	15 (8.7)	
Italian nationality	227 (94.6)	65 (97.0)	162 (93.6)	0.582
CDC stage C	69 (28.7)	12 (17.9)	57 (32.9)	0.021
Anti-HCV	61 (25.4)	18 (26.9)	43 (24.9)	0.748
HBsAg	18 (7.5)	6 (9.0)	12 (6.9)	0.594
Time from HIV diagnosis^a^	14 (5–23)	12 (4–21)	15 (6–23)	0.273
Time on ARV therapy^a^	10 (4–18)	12 (3–17)	10 (4–18)	0.293
Nadir CD4 count^a^	213 (68–326)	308 (202–413)	175 (43–286)	<0.001
Zenith HIV RNA^a^ (log10 copies/mL)	5.06 (4.66–5.45)	5.06 (4.52–5.26)	5.06 (4.71–5.50)	0.322
Baseline CD4 count^a^	586 (445–869)	662 (494–908)	568 (421–817)	0.082
Previous virologic failure	104 (43.7)	30 (46.2)	74 (42.8)	0.640
Years of virologic suppression^a^	4 (1–8)	1 (0–5)	5 (1–9)	<0.001
Therapies before switch				
2 NRTI+INI	57 (23.8)	14 (20.9)	43 (24.9)	0.518
2 NRTI+NNRTI	56 (23.3)	18 (26.9)	38 (22.0)	0.421
2 NRTI+PI	92 (38.3)	27 (40.3)	65 (37.6)	0.697
Mono/dual therapy	24 (10.0)	3 (4.5)	21 (12.1)	0.076
Other	12 (5.0)	6 (9.0)	6 (3.5)	0.075
Reasons for previous treatment discontinuation				
Toxicity	54 (22.5)	8 (11.9)	46 (26.6)	<0.001
Simplification	90 (37.5)	45 (67.2)	45 (26.0)	
DDI	54 (22.5)	0	54 (21.2)	
Other	42 (17.5)	14 (20.9)	28 (16.2)	

DDI, drug-drug interactions; IDU, intravenous drug users; INI, integrase inhibitor; MSM, men who have sex with men; (N)NRTI, (non-) nucleoside reverse transcriptase inhibitor; PI, protease inhibitor.

a Values within brackets are expressed in percentage except for median values (interquartile range).

# Sub article: P107 Durability, metabolic impact and efficacy of switching from a PI/r- to an INI-containing regimen in a monocentric cohort of drug-experienced HIV-positive subjects

**Published:** 2016-10-23

**Authors:** Stefano Rusconi, Letizia Oreni, Tiziana Formenti, Andrea Giacomelli, Valentina Di Cristo, Angelica Lupo, Elisa Colella, Marco Franzetti, Anna Lisa Ridolfo, Massimo Galli

**Affiliations:** 1 Infectious Diseases Unit, DIBIC Luigi Sacco, Universita’ degli Studi di Milano, Milan, Italy

## Abstract

**Introduction**: Boosted protease inhibitors (PI/r) have been the cornerstone of antiretroviral therapy for many years. Patients and physicians are increasingly worried about long-term safety of anti-HIV drugs. With this aim, we analyzed our cohort of drug-experienced patients previously treated with a PI/r who were switched to an integrase inhibitor (INI).

**Methods**: Hundred and thirty-one patients were studied over time. We evaluated several parameters involved in the persistence of treatment, such as CD4+ lymphocytes, HIV RNA, previous PI/r exposure glucose, creatinine, AST, ALT, amylase, triglycerides, total and HDL cholesterol and HCV/HBV status. The probability of remaining on an INI-containing regimen was estimated using Kaplan-Meier curves, paired samples were examined with Friedman test or Cochran Q test, if the variables were quantitative or dichotomous, respectively. Baseline clinical predictors of the INI-containing regimen survival were assessed by a multivariable Cox proportional hazard regression model. We also evaluated dual-energy X-ray absorptiometry (DXA) during INI treatment.

**Results**: Reasons for interrupting PI/r were: drug interactions 8.4%, immuno-virological failure 13.7%, side effects 26.7% and simplification 51.1%. The median observation time of the cohort was 17.8 months (IQR 5.2–40.1). Among the 131 patients, 26 interrupted the INI-containing regimen and 105 continued at the last observation. The probability of maintaining an INI-containing regimen was 0.91 (95% CI 0.86–0.96) at 6 months, 0.86 (0.80–0.93) at 12 months and 0.82 (0.74–0.89) at 18 months. The treatment survival differed at the last observation according to the PI/r included in the previous regimen, with a significant difference among atazanavir (ATV)/r, lopinavir (LPV)/r, fosamprenavir (FPV)/r and darunavir (DRV)/r (p<0.0001) (Figure 1). PI/r included in the previous regimen was confirmed to be independently associated to the INI-containing regimen durability by multivariable analysis. No change in treatment survival was observed after stratifying for the three INI used in clinical practice, although there was a significant difference (p=0.014) in the switch to RAL various PI/r (FPV/r>LPV/r>ATV/r>DRV/r). Among the parameters that we followed over time, HIV RNA <37 copies/mL increased between baseline and month 12 (67% vs. 89%; p<0.0001), median triglycerides decreased (155 vs. 121 mg/dL; p<0.0001) and median total cholesterol decreased (185 vs. 174 mg/dL; p=0.013). DXA was assessed in 15 patients on both lumbar spine and bilateral hips, being normal, osteopenia or osteoporosis in 47%, 40% and 13%, and 54%, 46% and 0%, respectively.

**Conclusions**: Regimen switch to INI demonstrated an optimal durability, together with virologic efficacy and maintenance of steady-state immunologic profile. Moreover, a favourable effect on lipids as far as triglycerides and total cholesterol was seen. Of note, a longer duration of the INI-containing regimen was observed after being treated with ATV/r and LPV/r in the previous drug combination.

**Abstract P107–Figure 1:** Treatment survival at the last observation according to the PI/r included in the previous regimen (atazanavir (ATV)/r, lopinavir (LPV)/r, fosamprenavir (FPV)/r and darunavir (DRV)/r[s1]).

# Sub article: P108 Switch to dolutegravir in HIV patients responding to a first-line antiretroviral treatment: 48 weeks results

**Published:** 2016-10-23

**Authors:** Luba Tau, Tomer Ziv-Baran, Ronit Cohen-Poradosu, Ari Leshno, Danny Alon, David Shasha, Steven Rapaport, Asaf Wasserman, Talya Finn, Eugene Katchman, David Hassin, Israel Yust, Tamar Brofman, Boaz Avidor, Dan Turner

**Affiliations:** 1 Crusaid Kobler AIDS Center Tel Aviv, Tel Aviv Sourasky Medical Center, Tel Aviv, Israel; 2 School of Public Health, Sackler Faculty of Medicine, Tel Aviv University, Tel Aviv, Israel

## Abstract

**Objectives**: Dolutegravir (DTG) has shown a potent antiviral effect and favourable safety profile. This study compares retrospectively efficacy and safety between patients who had responded to an ARV regimen which was continued and prospectively those who were switched to DTG. Results at 48 weeks are presented.

**Methods**: This cohort study was performed on all patients followed in Tel Aviv who had responded to a non-DTG first-line ARV regimen (HIV-1 viral load <200 copies/mL) for at least 6 months. The prospective study group (group A) was switched to a DTG-containing regimen while the retrospective group (group B) continued their non-DTG ARV regimen. Laboratory parameters analyzed within 48 weeks include HIV viral load (VL), CD4 cell count, CD4/CD8 ratio, plasma fasting glucose, total cholesterol, HDL cholesterol, LDL cholesterol, ALT, creatinine level, WBC, haemoglobin and platelet count. Adverse clinical events were recorded after reviewing the medical records.

**Results**: Analysis included 157 patients (73 in group A; 84 in group B). Before switching to DTG 24 patients in group A were treated with raltegravir, 36 with PI and 13 with NNRTI. Amongst group B, 31 patients were treated with raltegravir, 39 with PI and 14 with NNRTI. At 48 weeks, 70 patients (96%) in group A and 76 (90.5%) in group B had VL <40 copies/mLl (p=0.183), VL >200 copies/mL was detected in one patient from group B. Median CD4 cell count in group A at baseline was 660 and 661 in group B (p=0.338). At 48 weeks, median CD4 count in group A was 707 and 734 in group B (p=0.275). Changes from baseline creatinine were higher in group B (p=0.015) but this difference was not clinically relevant. Glucose, total cholesterol, LDL and HDL cholesterol, and ALT levels were similar in both groups. Also, there was no difference in complete blood count parameters (WBC, HGB, PLT) or CD4/CD8 ratio between the groups. In group A adverse clinical events were noted in 19% of patients. DTG was stopped in two patients (2.7%) due to adverse effects. In group B adverse effects were noted in 31% of patients. Fifty percent of patients on NNRTI treatment experienced side effects, 38% of patients in PI group and 6% in the raltegravir group.

**Conclusions**: Switching treatment-responding patients to DTG was effective and safe at 48 weeks.

# Sub article: P109 Antiretroviral treatment received by patients in the Spanish VACH cohort: change over time

**Published:** 2016-10-23

**Authors:** Bernardino Roca

**Affiliations:** 1 Infectious Diseases and Medicine, VACH Cohort of Spanish Hospitals, Castellon, Spain

## Abstract

**Introduction**: Antiretroviral treatment has had a dramatic impact on HIV infection control, but continued changes in recommended treatments have occurred throughout the history of the disease. In this study, we aim to achieve knowledge on changes that have taken place over the last 20 years on antiretroviral therapy prescription.

**Materials and methods**: This study is based on the Spanish VACH cohort of HIV-infected patients. The cohort was established in 2000, and is participated by 23 hospitals, belonging to most regions of the country. Data from all patients are included in a common software, Advanced HIV, specifically developed for the follow-up of HIV-infected patients. For this study we obtain from that application sociodemographic and clinical data from all patients, and analyze treatment that patients have taken in four separate time periods. We use descriptive and basic bivariate statistics.

**Results**: On 31 January 2016 the VACH cohort was formed by a total of 33,729 patients; 25,986 (77.04%) are men. According to data from the last registered visit, mean and standard deviation of age of all patients is 45.29±12.53 years; 80.10% of patients are receiving antiretroviral treatment; mean and standard deviation of CD4 lymphocyte count is 541±348 cells/mm^3^ and 75% have HIV RNA below 200 copies/mL. Table 1 and Figure 1 show the number of patients that are taking each modality of antiretroviral treatment at each one of four time periods, according to data from the last registered visit from every patient. There is a strong association between taking treatment and survival in all four time periods (p<0.0001).

**Conclusions**: Modalities of antiretroviral treatment received by patients in the Spanish VACH cohort has substantially varied a long time. Almost all HIV-infected patients receive antiretroviral treatment now. Combination treatment with two nucleoside/tide analogue and one integrase strand transfer inhibitor is the modality of treatment most commonly used now.

**Figure 1:** Main results of the study.

**Table 1:** Main results of the study

	2NA+II	2NA+PI	2NA+NN	Other treatment	No treatment
1996–2005	0	1623	1511	1214	2858
2006–2010	81	2240	3394	1033	1043
2011–2013	325	1552	2921	1324	466
2014–2015	3018	1575	2470	1965	230

II, integrase strand transfer inhibitor; NA, nucleoside/tide analogue; NN, non-nucleoside analogue; PI, protease inhibitor.

# Sub article: P110 The RAL-AGE study: benefits of switching to raltegravir-containing regimens in the older population

**Published:** 2016-10-23

**Authors:** Paolo Pavone, Noemi Giustini, Gabriella d'Ettorre, Sara Serafino, Ivan Schietroma, Giuseppe Corano Scheri, Ivano Mezzaroma, Mauro Andreotti, Andrea Mastrangelo, Claudio Maria Mastroianni, Vincenzo Vullo

**Affiliations:** 1 Public Health and Infectious Diseases, Sapienza University, Rome, Italy; 2 Public Health and Infectious Diseases, Policlinico Umberto I, Rome, Italy; 3 Department of Therapeutic Research and Medicines Evaluation, Istituto Superiore di Sanità, Rome, Italy

## Abstract

**Introduction**: Raltegravir (RAL) is considered one of the better-tolerated antiretroviral medications, due to limited side effects and few long-term safety concerns. Furthermore RAL displays minimal drug–drug interactions, making it a good option for ageing patients on multiple medications but the use of bid regimens in the elderly is sometime avoided due to poor adherence concerns.

**Materials and methods**: We retrospectively evaluated 20 HIV+, over 60-years-old, experienced patients, who had switched from any antiretroviral drug to raltegravir-based nuc-sparing/protease inhibitors(PI)-containing regimens (n=10) or standard nucleoside-backbone regimens (n=10) because of toxicity, convenience or other reasons. Data were collected from medical records. The time horizon for patient follow-up was at least 12 months. The following information was extracted from the database of the department: age, sex, race, smoking, risk factors, AIDS history, hepatitis, comorbidities, BMI, blood count, HIV RNA, CD4+, CD8+, previous ART regimens, creatinine, cholesterol and triglycerides, and cholestasis index. SPSS software was used.

**Results**: The median age of the patients was 64.5 years (15 males, 5 females) with a median of HIV diagnosis years of 13. HIV RNA at baseline was undetectable in most of the patients except two. Median CD4+ count was 450.5 cells/mm^3^ (IQR 353–717). Twelve patients had AIDS history. Reasons to switch were renal insufficiency, dyslipidaemia, HIV drug resistance and drug–drug interactions. No adverse effect related to the use of raltegravir was reported. Only one patient presented virologic failure, whereas viremic blips were observed in four patients. After switching to RAL-containing regimens triglycerides values showed a statistically significant reduction from a median value of 165 mg/dL to 111.5 mg/dL (p=0.016). Comparing patients who switched to a standard nucleoside-backbone containing regimen (NRTI-R) versus those who switched to PI-based nuc-sparing regimens (PI-R) we found that only NRTI-R patients presented a statistically significant reduction of triglycerides (155 mg/dL at T0 vs. 95 mg/dL at T2, median values, p=0.047). Furthermore, the NRTI-R patients compared with PI-R patients presented reduced values of creatinine (0.9 mg/dL vs. 1.1 mg/dL, p=0.043), reduced values of alkaline phosphatase (47 UI/L vs. 85 UI/L, p=0.046) and higher levels of CD4+ count (635 cells/mm^3^ vs. 453 cells/mm^3^, p=0.035).

**Conclusions**: RAL-containing regimens are safe and highly effective in the older population. Reduction of trigliceryde levels is more pronounced when RAL is used with nucleoside-backbone than in PI-containing regimens. When using RAL, switching to a standard nucleoside-backbone regimen appears to be less toxic than nuc-sparing/PI-containing regimens in older patients.

# Sub article: P111 Characteristics and satisfaction survey in patients switching to darunavir/cobicistat (DRV/c)

**Published:** 2016-10-23

**Authors:** Maria Vivancos-Gallego, Ana Moreno, Carmen Quereda, Jose Luis Casado, Cristina Gomez Ayerbe, Matilde Sanchez-Conde, Santos Del Campo, Santiago Moreno, Maria Perez Elias

**Affiliations:** 1 Infectious Diseases, Instituto Ramón y Cajal de Investigación Sanitaria, Madrid, Spain

## Abstract

**Introduction**: Coformulated ARV in a single pill, as DRV/c could improve adherence and satisfaction to ARVs. No studies had explored this outcome with new boosted PI, DRV/c [1,2]. A satisfaction questionnaire was conducted to know how patients feel with the new treatment.

**Methods**: From July 2015 to January 2016, we retrospectively reviewed all patients switching to a DRV/c regimen. After switching to DRV/c a short satisfaction questionnaire was filled out by participants. All questions referred to the changes with respect to the prior regimen. Overall satisfaction and convenience were categorized as: better, equal or worse. All symptoms were evaluated and could be categorized as getting better or worse and scored 1–10.

**Results**: Hundred and sixty-nine patients started a DRV/c regimen: 76.9% men, with a median age of 52.3 years, 59.5% IDU as route of HIV transmission, AIDS stage 50.6%, HCV co-infection 56.6%, baseline CD4 count 694 and a median exposure to ART of 17 (1–27) years. Main reasons for switching were simplification 80% and toxicity 9%. Previous treatment was PI monotherapy (31%), PI+2 NRTIs and PI+3TC (15.6%). Mean reduction in the number of tablets was significant (3 to 1.8; p<0.001). After switching, boosted PI distribution was: DRV/c 34%, DRV/c+3TC 18% and DRV/c+DTG 15%. Fifty-four out of 169 patients were surveyed. Majority, 92.5% answered that they felt better than before or equal, while 7.5% felt worse. Convenience was scored, 81.1% better, 15.1% equal or 3.8% worse. Asking about specific symptoms, 26.4% referred some symptom that improved (with a scored 2.13 vs. 1.47; p=0.002), while 28.3% referred at least one worsening symptom (1.13 vs. 1.94; p<0.001). There was a statistically significant improvement in frequency of stools (1.69 vs. 1.19; p=0.002). A higher improvement was found when patients came from DRV/r compared with those who came from non-DRV/r (97.6% vs. 72.7%; p=0.005). Patients with prior non-DRV/r compared with DRV/r regimens had a higher frequency of stools score before changing to DRV/c (2.64 vs. 1.45; p=0.043), while turned to similar frequency of stools score after switching (1.14 vs. 1.36; p=0.56).

**Conclusions**: DRV/c was prescribed mostly in HIV-infected patients with longer time of exposure to ART, previous AIDS and HIV/HCV co-infection. Simplification was the main reason for switching to DRV/c. Patients switching to DRV/c valued the new treatment as better or equal and more convenient. Patients taking DRV/r before the switch referred more satisfaction comparing with those who were taking non-DRV/r.

## References

[1] Tashima K, Crofoot G, Tomaka FL, Kakuda TN, Brochot A, Vanveggel S (2014). Phase IIIb, open-label single-arm trial of darunavir/cobicistat (DRV/COBI): week 48 subgroup analysis of HIV-1-infected treatment-nave adults. J Int AIDS Soc.

[2] Kakuda TN, Crauwels H, Opsomer M, Tomaka F, van de Casteele T, Vanveggel S (2015). Darunavir/cobicistat once daily for the treatment of HIV. Expert Rev Anti Infect Ther.

# Sub article: P112 Short-term safety and tolerability of switch of backbone antiretroviral agents to coformulated tenofovir disoproxil fumarate/emtricitabine in HIV-positive Taiwanese patients

**Published:** 2016-10-23

**Authors:** Hsi-Yen Chang, Pei-Ying Wu, Jun-Yu Zhang, Shang-Ping Yang, Yu-Zhen Luo, Wen-Chun Liu, Hsin-Yun Sun, Chien-Ching Hung, Shan-Chwen Chang

**Affiliations:** 1 Center of Infection Control, National Taiwan University Hospital, Taipei, Taiwan; 2 Department of Internal Medicine, National Taiwan University Hospital, Taipei, Taiwan

## Abstract

**Introduction**: Coformulated tenofovir disoproxil fumarate (TDF)/emtricitabine (FTC) has become the recommended backbone antiretrovirals in combination with other non-nucleoside reverse transcriptase inhibitors (non-NRTIs), protease inhibitors or integrase strand transfer inhibitors. Access to this coformulation is limited, however, in many countries in Asia-Pacific regions. We aimed to assess the safety and tolerability of switch of two NRTIs backbone to TDF/FTC in treatment of HIV infection in Taiwan, where access to TDF/FTC was not available until May 2015.

**Materials and methods**: All HIV-positive patients with a mean age of 40.5 years and 88.5% being homosexual males whose backbone antiretrovirals were switched to TDF/FTC between May 2015 and May 2016 were included in this analysis. We collected information on the demographic and clinical characteristics before switch and CD4, plasma HIV RNA load (PVL), lipids, serum creatinine, glycosuria, proteinuria and beta-2 microglobulin at baseline and during follow-up. Adverse effects and causes of discontinuation were also recorded.

**Results**: During the 12-month observation period, 1164 patients switched from TDF and lamivudine (n=818), coformulated abacavir/lamivudine (n=229) and coformulated zidovudine/lamivudine (n=117) to TDF/FTC, without changes made to the third agents, after a mean exposure duration of 60 (SD, 47), 91 (SD, 32) and 40 (SD, 40) weeks, respectively. CD4 and PVL before switch were 613 cells/mm^3^ (SD, 284) and 1.50 log10 copies/mL (SD, 0.70). After an interval of 240 days (SD, 68), the mean CD4 and PVL remained stable (610 cells/mm^3^ and 1.38 log10 copies/mL, respectively), so was mean serum creatinine for TDF and lamivudine group (0.93 vs. 0.92 mg/dL) and zidovudine/lamivudine group (0.94 vs. 0.96 mg/dL), but it increased from 0.94 to 1.12 mg/dL for abacavir/lamivudine group. Mean total cholesterol, triglyceride and low-density lipoprotein-cholesterol decreased from 178.0 to 167.2, 178.1 to 134.9, and 105.0 to 99.4 mg/dL for abacavir/lamivudine group, respectively, and 161.4 to 152.3, 150 to 135.1 and 97.1 to 94.3 mg/dL for zidovudine/lamivudine group, respectively. Urine beta-2 microglubulin increased from 1.24 to 1.44, 0.68 to 1.62 and 1.37 to 2.9 mg/L, for TDF and lamivudine, abacavir/lamivudine, and zidovudine/lamivudine group, respectively. TDF/FTC was discontinued in 46 patients (4.0%), due to diarrhoea (n=5), nausea (n=6), allergy (n=5), paresthesias (n=5), increased serum creatinine (n=5), increased PVL and emergence of resistance (n=6) and other miscellaneous causes (n=16).

**Conclusions**: Coformulated TDF/FTC was generally well tolerated and safe in HIV-positive Taiwanese, with additional lipid-lowering effects in those who had been on abacavir/lamivudine- or zidovudine/lamivudine-containing regimens. Periodic renal monitoring for renal tubular dysfunction is warranted.

# Sub article: P113 Optimizing viral load testing to improve quality of care and client retention for PLHIVs in HIV clinic in Northern Nigeria: impact and experiences

**Published:** 2016-10-23

**Authors:** Emmanuel Nwabueze, Nwokedi Ndulue, Ginika Egesimba, Med Makumbi, Zipporah Kpamor, Emeka Okechukwu, Joy Kolin

**Affiliations:** 1 Management Sciences for Health, ProACT, Abuja, Nigeria; 2 USAID, Abuja, Nigeria

## Abstract

**Introduction**: PLHIVs on antiretroviral (ARV) regimen with plasma viral load above 1000 copies/mL based on two consecutive viral load measurements after 3 months, with adherence support, are said to be in virology failure. Virology failure leads to easy HIV transmission, evitable morbidity and mortality especially if antiretroviral therapy (ART) drugs are switched without initial VL testing. Unfortunately, VL testing is so costly. The few available PCR laboratories and point-of-care (POC) VL machines that are used for the testing are not evenly distributed in the country. Currently over 70% of PLHIVs have not done any VL test in the last one year since enrolled into care. In 2014, MSH ProACT, a USAID-funded programme in Nigeria, launched a PCR laboratory in Usman Danfodiyo University Teaching Hospital Sokoto to provide free VL testing. This is in alignment with PEPFAR and UNAID 90:90:90 strategies. This study was to find out the impact of the PCR laboratory on PLHIVs’ quality of care.

**Methods**: Retrospective study was done one year after the PCR launch. Clinical audit of the VL register and chart review of 268 folders of clients with detectable VL results >20 VL copies were conducted. Analysis of client retention data was also done. Update training was conducted for clinicians and other healthcare workers (HCWs) working in the ART clinic to optimize VL testing.

**Results**: The analysis of the VL register showed that 268 out of 583 recorded results (46%) had detectable VL of which 141 (53%) are >1000 copies/mL. Chart review of the 268 folders revealed that 162 were not switched and 106 were switched: 22 (21%) were rightly switched and 84 (79%) were wrongly switched (considering the retrogressive outcome documented). 66 (78%) of these wrong ART switches had >1000 copies of which 28 (42%) were in World Health Organization stages 3 and 4. These wrong switches were made prior to the launch of the PCR laboratory.

**Discussion**: After the launch, seven (100%) of the subsequent switches were done well. Currently most clients do better without requiring switching post VL testing and adherence counselling. Within a year, client retention improved to 77% as compared with 59% in the previous year. Quality of care and clients’ adherence improved.

**Conclusions**: VL testing improves quality of care for all PLHIVs. If PCR laboratories or POC VL machines are suitably deployed, health system will be strengthened, there will be reduction in wrong switches and, subsequently, reduction in mortality and morbidity among PLHIVs.

# Sub article: P114 Knowing the epidemic is the best way to define diagnosis and treatment strategies to reach the 90–90–90 goals: the experience of Portugal in using the ECDC HIV modelling tool

**Published:** 2016-10-23

**Authors:** Antonio Diniz, Jose Loff, Helena Cortes-Martins

**Affiliations:** 1 Immunodeficiency Unit, Directorate General of Health and CHLN, Lisboa, Portugal; 2 Department of Epidemiology and Surveillance, Directorate General of Health, Lisboa, Portugal; 3 Infectious Diseases, National Health Institute, Lisboa, Portugal

## Abstract

**Introduction**: In Portugal, data from the continuous of care show that in 2014 approximately 34,000 persons living with HIV (PLHIV) were in care, 82.8% of which on antiretroviral treatment and 78.4% virologically suppressed. However, updated estimates for HIV prevalence, incidence and PLHIV diagnosed are still missing, making difficult defining appropriate diagnosis and treatment strategies to reach the 90–90–90 goals.

**Material and methods**: Annual estimates (1983–2014) for the number of PLHIV, undiagnosed fraction, new infections and time to diagnosis were produced using the new software application “ECDC HIV modelling tool,” and the data from the national HIV surveillance system. The model was constructed based on the “Incidence Method” [1]. Several cut points were considered to fit the model to the Portuguese HIV epidemic.

**Results**: Estimates were produced for total HIV-infected population and for main transmission categories: heterosexual, men who have sex with men (MSM), intravenous drug users (IVDU) (Table 1). At the end of 2014, an estimated number of 44,176 individuals were living with HIV in Portugal (prevalence: 0.43%). Of those, 4298 (9.7%) were not aware of their infection.

**Conclusions**: Current estimates indicate a lower prevalence than previous assessments. Estimated undiagnosed fraction and time to diagnosis vary for different transmission modes reflecting past interventions and current trends of the epidemic. Portugal has now updated data that will allow building the “treatment cascade.” According to these results, Portugal has reached the cascade first goal (90% of the PLHIV already diagnosed) with time to diagnosis becoming progressively shorter. In order to reach all 90–90–90 goals, we must now address our efforts to define and apply new and stronger strategies related to linkage/retention in care and to treatment.

**Abstract P114–Table 1:** Estimates of PLHIV, PLHIV diagnosed and undiagnosed, undiagnosed fraction, new infections and time to diagnosis, global and by transmission categories (2014)

	Global	Heterosexual	MSM	IVDU
PLHIV	44,176 (43,175–45,154)	22,109 (21,416–23,236)	7930 (7532–8440)	13,353 (13,119–13,900)
PLHIV diagnosed	39,877 (39,476–40,295)	19,239 (18,987–19,574)	7071 (6896–7288)	13,193 (13,008–13,513)
PLHIV undiagnosed	4298 (3508–5274)	2870 (2200–3783)	859 (570–1292)	161 (95–665)
Undiagnosed fraction (%)	9.7 (8.0–11.8)	13 (10.3–16.3)	10.8 (7.4–15.5)	1.2 (0.7–4.8)
New infections	528 (36–1088)	414 (47–953)	106 (58–414)	5 (0–281)
Time to diagnosis (years)	4.1 (3.7–4.4)	4.5 (4.0–5.1)	2.8 (2.2–3.4)	3.4 (1.7–5.8)

## References

[1] Van Sighem A, Nakagawa F, De Angelis D, Quinten C, Bezemer D, de Coul EO (2015). Estimating HIV incidence, time to diagnosis, and the undiagnosed HIV epidemic using routine surveillance data. Epidemiology.

# Sub article: P115 Drug retention time: a real-life Swedish nationwide cohort study on InfCareHIV 2009-2014

**Published:** 2016-10-23

**Authors:** Amanda Häggblom, Stefan Lindbäck, Magnus Gisslén, Leo Flamholc, Bo Hejdeman, Andreas Palmborg, Amy Leval, Eva Herweijer, Sverrir Valgardsson, Veronica Svedhem

**Affiliations:** 1 Department of Infectious Diseases, County Council of Gävleborg, Gävle, Sweden; 2 Janssen Medical Affairs, Stockholm, Sweden; 3 Department of Infectious Diseases, Sahlgrenska Academy, Institute of Biomedicine, University of Gothenburg, Gothenburg, Sweden; 4 Department of Infectious Diseases, Malmö University Hospital, Malmö, Sweden; 5 Department of Infectious Diseases, Venhälsan-Södersjukhuset, Stockholm, Sweden; 6 Medical Epidemiology and Biostatistics, Karolinska Institutet, Stockholm, Sweden; 7 Janssen Medical Affairs, Oslo, Norway; 8 Department of Infectious Diseases, Karolinska University Hospital, Stockholm, Sweden

## Abstract

**Introduction**: As HIV infection requires lifelong treatment, studying drug retention times and factors influencing treatment durability is essential. The Swedish database InfCareHIV includes high-quality data from more than 99% of all patients diagnosed with HIV infection in Sweden and provides a unique opportunity to examine outcomes in a nationwide real-world cohort.

**Methods**: Adult patients who started a new therapy defined as a new third agent (all ARVs that are not NRTIs) 2009 to 2014 with more than 100 observations in treatment-naïve or treatment-experienced patients were included. Dolutegravir was excluded due to short follow-up period. Multivariate Cox proportional hazards models were used to estimate hazard ratios for treatment discontinuation.

**Results**: Two thousand five hundred and forty-one treatment-naïve patients started 2583 episodes of treatments with a third agent. Efavirenz was most commonly used (n=1096) followed by darunavir (n=504), atazanavir (n=386), lopinavir (n=292), rilpivirine (n=156) and raltegravir (n=149). In comparison with efavirenz, patients on rilpivirine were least likely to discontinue treatment (adjusted HR 0.33; 95% CI 0.20–0.54, p<0.001), while patients on lopinavir were most likely to discontinue treatment (adjusted HR 2.80; 95% CI 2.30–3.40, p<0.001).

Two thousand nine hundred and ninety-one treatment-experienced patients started 4552 episodes of treatments with a third agent. Darunavir was most commonly used (n=1285), followed by atazanavir (n=806), efavirenz (n=694), raltegravir (n=622), rilpivirine (n=592), lopinavir (n=291) and etravirine (n=262). Compared to darunavir all other drugs except for rilpivirine had higher risk for discontinuation in the multivariate adjusted analyses: rilpivirine (HR 0.66; 95% CI 0.52–0.83, p<0.001), atazanavir (HR 1.71; 95% CI 1.48–1.97, p<0.001), efavirenz (HR 1.86; 95% CI 1.59–2.17, p<0.001), raltegravir (HR 1.35; 95% CI 1.15–1.58, p<0.001), lopinavir (HR 3.58; 95% CI 3.02–4.25, p<0.001) and etravirine (HR 1.61; 95% CI 1.31–1.98, p<0.001).

Besides the ARV treatment, certain baseline characteristics of patients were independently associated with differences in drug retention time. In naïve patients, the use of other backbone than TDF/FTC or ABC/3TC increased the risk for early treatment discontinuation. In treatment-experienced patients, detectable plasma viral load at the time of switch or being highly treatment experienced increased the risk for early treatment discontinuation.

**Conclusions**: Treatment durability is dependent on several factors, among others patient characteristics and ART guidelines. The choice of third agent has a strong impact, with significant differences found between drugs.

# Sub article: P116 Discontinuation of dolutegravir (DTG)-based regimens in clinical practice

**Published:** 2016-10-23

**Authors:** Maria J Vivancos-Gallego, Ana Moreno, Maria J Perez-Elias, Cristina Gomez Ayerbe, Jose Luis Casado, Carmen Quereda, Matilde Sanchez-Conde, Sergio Serrano Villar, Fernando Dronda, Enrique Navas, Miguel Angel Rodriguez, Santiago Moreno

**Affiliations:** 1 Infectious Diseases, Instituto Ramon y Cajal de Investigación Sanitaria, Hospital Ramon y Cajal, Madrid, Spain

## Abstract

**Introduction**: Real-life data have shown a higher rate of side effects with dolutegravir (DTG)-based regimens than previously described in clinical trials. In order to confirm these observations, we have reviewed our experience in patients who discontinued DTG for any reason.

**Materials and methods**: Retrospective analysis of all patients who discontinued DTG in our hospital cohort. Pre-treated and treatment-naïve patients were included. Baseline characteristics at the time of DTG initiation and antiretroviral therapy before and after DTG were recorded. We describe any reason for dolutegravir discontinuation.

**Results**: Among 2470 HIV-infected patients, 827 (33.5%) patients received DTG (69.4% STR of ABC/3TC/DTG) from September 2014 to May 2016 for a median period of 156.8 days (4–1199). A total of 104 (12.6%) patients discontinued DTG for any reason and were switched to other ARV regimens. Of these 104 patients (60.6% STR of ABC/3TC/DTG), mean age was 49.6±10.5 years, 74 (71.2%) were men, baseline CD4 count was 574±324 cells/mm^3^, viral load was detectable before starting DTG in 17 (16.3%) and 30 (29%) had previous AIDS. Only seven (6.7%) patients were naïve. There were 41 patients (39.4%) who were lost to follow-up. Main reasons for stopping DTG-based regimen were toxicity in 36 patients (4.3% of all patients who initiated DTG, 33.9% of all discontinuations), and physician's decision in 11 patients (1.3% and 10.6%, respectively). Most frequent toxicities leading to drug interruption included headache (nine patients), high cholesterol (eight patients), insomnia (seven patients) and dizziness (six patients). One patient developed serious mood disorders with early recovery after discontinuation.

**Conclusions**: In our real-life cohort, we did find a high proportion of DTG discontinuation attributable not only to toxicity. CNS adverse events are the most frequent cause of discontinuation. Ongoing pharmacovigilance is important to identify events that might be associated with the drug.

# Sub article: P117 Dolutegravir (DTG) monotherapy treatment de-escalation in virological controlled, pre-treated HIV patients: results from the DoluMono cohort study

**Published:** 2016-10-23

**Authors:** Celia Oldenbüttel, Eva Wolf, Ayla Ritter, Sebastian Noe, Silke Heldwein, Rita Pascucci, Carmen Wiese, Ariane von Krosigk, Eva Jägel-Guedes, Hans Jäger, Annamaria Balogh, Christine Kögl, Christoph Spinner

**Affiliations:** 1 MVZ, Karlsplatz, Munich, Germany; 2 Department of Medicine II, University Hospital Klinikum rechts der Isar, Munich, Germany

## Abstract

**Introduction**: Long-term antiretroviral treatment (ART) with (potential) toxicity in HIV-infected patients requires ongoing investigation of novel strategies. Besides “nuke-free”‘ concepts and protease inhibitor monotherapy, integrase inhibitor (INSTI) monotherapy may offer a favourable safety profile. The high resistance barrier of dolutegravir (DTG) might be crucial for successful maintenance of virological control, but published data are sparse.

**Methods**: Retrospective, mono-centric cohort study. Patients on suppressive ART switched to DTG monotherapy in clinical routine practice fulfilling inclusion criteria (HIV RNA level <50 copies/mL for ≥6 months at time of switch [one accepted blip <200 copies/mL with re-suppression], no known INSTI resistance or prior INSTI failure, no replicative HBV infection and no history of AIDS) were enrolled.

**Results**: We identified 31 patients with week 24 follow-up data. Median time on previous ART was 26 months (24–28) including an NNRTI in 32%, a boosted PI in 6% and an INSTI in 61% of cases. At week 24, HIV RNA remained <50 copies/mL in 94% of all patients. One patient discontinued DTG monotherapy on his own wish (3%), and in another patient confirmed virological failure (3%) with HIV RNA 538 copies/mL and evolution of INSTI mutations Q148H/G140S was documented. Changes in immune, renal and metabolic status/function showed no statistically significant changes, except a significant decrease of gGT (Table 1).

**Conclusion**: Switching to DTG monotherapy in selected patients might be safe and effective. However, in one case evolution of INSTI resistance was observed. Further studies should assess risk factors for DTG monotherapy failure. Meanwhile, caution should be warranted.

**Abstract P117–Table 1:** Overview of selected study parameters at baseline and week 24 with delta and p-value

Parameter	Week 0 (n = 31)	Week 24 (n = 30)	Delta (week 24-0)	p
HIV RNA <50 copies/mL (patients), ITT snapshot	31 (100%)	29 (94%)	2 (6%)	0.500
CD4 absolute (/µL)	752 (581–970)	747 (573–913)	21.5 (−98–85)	0.959
ALT (U/L)	33 (26–44)	32 (26–42)	1.5 (−9–13)	0.758
AST (U/L)	21 (19–26)	23 (19–29)	0.5 (−5–5)	0.910
gGT (U/L)	36 (28–64)	30 (23–51)	−4.5 (−22–1)	0.014
HDL cholesterol (mg/dL)	56 (45–68)	51.5 (43–62)	−2.5 (−8–3)	0.174
LDL cholesterol (mg/dL)	115 (92–142)	122 (95–138)	0.5 (−9–26)	0.300
Total cholesterol (mg/dL)	182 (160–214)	194 (178–221)	4 (–28–33)	0.524
Triglycerides (mg/dL)	146 (96–187)	130 (98–196)	3 (−38–45)	0.681
Serum creatinine (mg/dL)	1.1 (1–1.4)	1.15 (1.03–1.43)	0.02 (−0.06–0.15)	0.225
eGFR (mL/min)	95 (81–114)	109.05 (83–120)	6 (–4–14)	0.077

# Sub article: P118 Outcomes of cabotegravir (CAB) treatment in HIV-1 ART-naive patients with chronic or acute hepatitis C virus (HCV) co-infection: data from the phase IIb programme

**Published:** 2016-10-23

**Authors:** Shanker Thiagarajah, David Dorey, Jenny Huang, Gilly Roberts, Britt Stancil, David Margolis

**Affiliations:** 1 Global Clinical Safety and Pharmacovigilance, GlaxoSmithKline, London, UK; 2 Clinical Statistics, GlaxoSmithKline, Mississauga, Canada; 3 Biostatistics, PAREXEL International, Durham, NC, USA; 4 Global Medical Strategy, ViiV Healthcare, Durham, NC, USA

## Abstract

**Introduction**: HCV co-infection is prevalent in the HIV-1 population, and acute HCV infection has been reported. CAB is a long-acting (LA) intramuscular integrase inhibitor (INI) in clinical development for the treatment of HIV in combination with LA rilpivirine. Acute reversible transaminitis has been reported with INI treatment. This report describes the safety and efficacy outcomes of CAB treatment for HIV-1 infected ART-naïve subjects with acute and chronic HCV co-infection during two ongoing phase IIb studies (LATTE and LATTE-2).

**Materials and methods**: Subjects with HCV co-infection were identified using HCV antibody results at baseline. Subjects with acute HCV infection were identified using polymerase chain reaction assay, once subjects had met prespecified criteria for liver aminotransaminase elevations (ATE). The following were assessed: protocol defined HIV virological failure (PDVF), withdrawals, emergent grade 2 or higher ATE on CAB, meeting predefined liver stopping criteria (LSC).

**Results**: In the intent-to-treat-exposed (ITT-E) population receiving CAB treatment, 22 of 490 (4.5%) subjects were HCV antibody reactive at baseline (Table 1). Of these 22 co-infected subjects, one subject developed PDVF through week 144 (LATTE, n=9), and no subject developed PDVF through week 48 (LATTE-2, n=13). Two co-infected subjects were withdrawn due to drug-related adverse events: suspected drug-induced liver injury (DILI) meeting LSC (n=1) and nausea (n=1). Three co-infected subjects had emergent grade 2 or higher ATE, two of which were withdrawn: DILI (n=1), withdrawal of consent-subject homeless with difficulty travelling (n=1). Ten of 490 (2%) subjects on LATTE and LATTE-2 developed acute HCV infections while on CAB, characterized by ATE. Five subjects were withdrawn after meeting LSC and transitioned to alternative ART. Five remaining subjects on LATTE-2 who met LSC had transaminase decline indicating stable disease or spontaneous clearance of HCV and were permitted per protocol to continue on CAB LA. All five acute HCV subjects continuing on CAB LA maintained HIV-1 viral suppression (<50 copies/mL at week 48). One (1/5) remaining acute HCV subject later discontinued CAB LA due to rebound ATE considered related to the underlying evolving HCV infection. Triumeq was started with continued HIV-1 viral suppression (<50 copies/mL at week 48).

**Conclusions**: Data from the phase IIb studies, albeit in small numbers of subjects, support the conclusion that chronic HCV co-infection does not adversely impact treatment outcomes in most subjects and uncomplicated acute HCV infection may not be a barrier to CAB LA as a treatment option.

**Abstract P118–Table 1:** Summary of PDVF and safety results by HCV infection status for LATTE and LATTE-2 subjects* exposed to cabotegravir

Hepatitis C infection status, ITT-E (N = 490)	Number of subjects with protocol defined virological failure, n (%)	Number of subjects with grade 2 or higher transaminitis, maximum post baseline emergent ALT or AST, n (%)	Number of subjects with drug-related adverse events leading to withdrawal, n (%)	Number of subjects who met protocol defined liver stopping criteria, n (%)
HCV co-infection (N = 22)	1/22 (4.5%)	3/22 (14%)	2/22 (9%)	1/22 (4.5%)
Acute HCV infection (N = 10)	0/10	10/10 (100%)	0/10	10/10 (100%)‡
Non-infected (N = 458)	12/458 (2.6%)†	45/458 (9.8%)	11/458 (2.4%)	3/458 (<1%)§

* LATTE (N = 181) used week 144 data cut and LATTE-2 (N = 309) used week 48 data cut

† Nine subjects were ineligible to enter the maintenance period (HIV-1 RNA =50 copies/mL just prior) and did not meet the confirmed PDVF criteria at time of withdrawal

‡ Five subjects with acute HCV infection met protocol defined liver stopping criteria but were permitted by protocol to continue on CAB LA

§ One additional subject who met liver stopping criteria based on local laboratory tests is not counted in the numerator.

# Sub article: P119 Antiretroviral treatment patterns in the US: an analysis of real-world data

**Published:** 2016-10-23

**Authors:** Bridgett Goodwin, Josephine (nhu) Tran, John White, Jianbin Mao, Felix Cao

**Affiliations:** 1 HIV Outcomes Research, Merck & Co., Inc., Whitehouse Station, NJ, USA; 2 Health Economics and Outcomes Research, Optum, Eden Prairie, MN, USA

## Abstract

**Introduction**: Recommended initial treatment regimens for HIV-infected individuals include a “backbone” of two nucleoside reverse transcriptase inhibitors (NRTIs) in combination with a “third agent” from another class such as integrase strand transfer inhibitor (INSTI), protease inhibitor (PI) or non-nucleoside reverse transcriptase inhibitor (NNRTI). Recent treatment guidelines have moved from recommending PI- and NNRTI-based regimens to more INSTI-based regimens. In treatment-experienced patients who fail therapy, it is recommended that the new regimen should include at least two fully active agents. This study describes the changes in third agents in the real-world setting over time.

**Methods**: This is descriptive analysis using a US insurance claims database. Patients with HIV and newly initiating a different third agent class (INSTI, PI or NNRTI) were identified from 1 July 2011 to 30 September 2015. Patients were excluded if they were not continuously enrolled in the health plan for a year.

**Results**: In total, 9525 patients with HIV started a new third agent regimen. A majority of patients were new initiators of third agents (77.0%) with the remainder of patients adding on or switching to a new third agent. Most were male (82.3%), had commercial type insurance (89.3%) and had a mean age of 45.2 (SD±11.9). In patients who newly initiated a third agent, 31.9% started on an INSTI, 24.8% on a PI and 43.3% on a NNRTI. However, the proportion of patients starting INSTI increased from 19.3% in 2012 to 50.3% in 2015, while the proportions of patients starting other third agents decreased over time (Figure 1). In third agent-experienced patients, 10.8% were on an INSTI, 59.3% on a PI and 37.5% on a NNRTI at baseline. Less than 2% of patients were on a fusion inhibitor or entry inhibitor, and 7.5% of patients were on multiple third agents at baseline. Among patients already on a third agent, 66.3% of patients added or switched to an INSTI, 10.6% to a PI and 23.1% to a NNRTI. The proportion of patients who added or switched to an INSTI increased from 49.4% in 2012 to 80.6% in 2015, while proportions of patients starting other third agents decreased over time.

**Conclusion**: There is an increase in new initiators and treatment-experienced patients starting INSTI compared to other third agents. Future studies are needed to examine the tolerability and outcomes related to these changes in third agents.

**Abstract P119–Figure 1:** The proportion of patients who initiated different third agents.

# Sub article: P120 Selected antiretroviral treatment option is associated with virological success: multicentre data from Poland

**Published:** 2016-10-23

**Authors:** Milosz Parczewski, Magdalena Leszczyszyn-Pynka, Adam Witor, Karolina Muller, Aleksandra Szymczak, Jacek Gasiorowski, Monika Bociaga-Jasik, Pawel Skwara, Anna Grzeszczuk, Elzbieta Jablonowska, Kamila Wójcik-Cichy, Juliusz Kamerys, Dariusz Bielec, Justyna Stempkowska, Aleksandra Kocbach, Wieslawa Bludzin

**Affiliations:** 1 Department of Infectious, Tropical Diseases and Immune Deficiency, Pomeranian Medical University, Szczecin, Poland; 2 Outpatients Clinic for Immune Deficiency, Regional Hospital, Chorzów, Poland; 3 Department of Infectious Diseases, Hepatology, Wroclaw Medical University, Wroclaw, Poland; 4 Department of Infectious Diseases, Jagiellonian University Medical College, Kraków, Poland; 5 Department of Infectious Diseases and Hepatology, Medical University of Bialystok, Bialystok, Poland; 6 Department of Infectious Diseases and Hepatology, Medical University of Lódz, Poland; 7 Department of Infectious Diseases, Medical University in Lublin, Lublin, Poland; 8 Department of Infectious Diseases, Medical University in Ostróda, Ostróda, Poland; 9 Department of Infectious Diseases, Regional Hospital, Opole, Poland

## Abstract

**Introduction**: Modern antiretroviral therapies allow to effectively suppress HIV-1 viral load in majority of treated cases; however, due to differences in clinical practice, virological success rates may vary significantly across the treatment centres. The aim of this study was to analyze treatment efficacy in real-life Polish cohorts and identify variables associated with undetectable HIV-1 viral load.

**Materials and methods**: Cross-sectional data on the antiretroviral treatment efficacy were collected for the 2249 (25.3% of total countrywide treated cases) patients followed up in 9/17 Polish HIV treatment centres. Data of patients on stable cART treated >6 months with at least one follow-up visit and HIV RNA measurement in 2016 were analyzed. Treatment options included nucleos(t)ide backbone (NRTI) plus protease inhibitor (PI) in 942 cases (37.7%), NRTI plus non-nucleoside reverse transcriptase inhibitor (NNRTI) in 768 (30.73%), NRTI plus integrase inhibitor (INI) in 632 (25.29%), NRTI-sparing regimen of PI+INI in 68 (2.72%) and other combinations in 89 (3.56%) individuals. Virological success was defined as HIV RNA <50 copies/mL in the last measurement taken in 2016. For statistics chi-squared test, Mann-Whitney U test and multivariate logistic regression models adjusted for gender, AIDS history, HIV viral load at baseline, lymphocyte CD4 nadir <200 cells/μL, transmission route and age were used.

**Results**: Undetectable viral load (<50 copies/mL) was observed in 2256 (90.28%) individuals. Virological success rate differed considerably across the regimens (835/942 [88.64%] for NRTI+PI, 732/768 [95.31%] for NRTI+NNRTI, 563/632 [89.08%] for NRTI+INI and 54/68 [79.41%] for PI+INI, p<0.0001). NRTI+NNRTI regimens were associated with higher adjusted odds ratio (aOR) of virological success compared to NRTI+PI [aOR 2.68 (95% CI 1.41–5.13), p=0.003], NRTI+INI [aOR 4.04 (95% CI 2.13–7.78), p<0.0001] or PI+INI [aOR 7.92 (95% CI 2.73–22.99), p<0.0001] (Figure 1). It should be noted, however, that patients receiving NRTI+NNRTI presented with lower baseline HIV viral load [median 4.64 (IQR 4.16–5.07) log copies/mL] and higher CD4 nadir [median 283 (IQR 175–403) cells/µL] compared to NRTI+PI-treated [median 4.9 (IQR 4.34–5.41) log copies/mL, p<0.001 and 190 (IQR 76–311) cells/µL, p<0.0001, respectively], NRTI+INI-treated [median 4.9 (IQR 4.41–5.44) log copies/mL, p<0.001 and 228 (IQR 90–393) cells/µL, p<0.0001, respectively] or PI+INI-treated cases [median 5.42 (IQR 4.79–5.94) log copies/mL, p<0.0001 and 155 (IQR 59–246) cells/µL, p<0.0001, respectively]. Additionally, patients on NRTI-sparing PI+INI regimen were notably older at diagnosis [median 38 (IQR 29–48) years] and treatment initiation [median 37 (IQR 31–46) years] compared to the remaining treated groups [median 31 (IQR 25–38) years, p<0.0001 and 33 (IQR 28–40) years, p<0.0001, respectively].

**Conclusions**: While NNRTI-based therapy is associated with higher virological efficacy, it may be explained by the preselection of patients with more favourable virological and immunological characteristics. Challenging-to-treat and older populations often receive PI+INI-based, NRTI-sparing regimens despite poorer efficacy in the clinical setting.

**Figure 1:** Antiretroviral regimen-associated differences in the frequency of HIV-1 viral load <50 copies/mL.

# Sub article: P121 Much less treatment modification with recently approved drugs: the Austrian HIV Cohort Study

**Published:** 2016-10-23

**Authors:** Michaela Rappold, Armin Rieger, Brigitte Schmied, Mario Sarcletti, Maria Geit, Bernhard Haas, Ninon Taylor, Manfred Kanatschnig, Gisela Leierer, Bruno Ledergerber, Robert Zangerle

**Affiliations:** 1 Dermatology and Venereology, Medical University of Innsbruck, Innsbruck, Austria; 2 Dermatology and Venereology, Medical University Vienna, Vienna, Austria; 3 Pulmonary Medicine, Otto Wagner Hospital Vienna, Vienna, Austria; 4 Dermatology and Venereology Allgemeines Krankenhaus Linz, Linz, Austria; 5 Infectious Diseases, Landeskrankenhaus Graz West, Graz, Austria; 6 Internal Medicine III, Paracelsus Medical University Salzburg, Salzburg, Austria; 7 Internal Medicine, Landeskrankenhaus Klagenfurt, Klagenfurt, Austria; 8 Infectious Diseases, University Hospital Zurich, Zurich, Switzerland

## Abstract

**Introduction**: Adverse effects and to a lesser degree viral failure of combination ART commonly result in treatment modification.

**Patients and methods**: Patients were analyzed for factors associated with treatment modification, defined as stop or as change of drugs. Dolutegravir (DGV), elvitegravir (EVG), raltegravir (RAL), darunavir (DRV) and rilpivirine (RPV) were separately analyzed. RPV and EVG were included only when taken as single-tablet regimen, and DRV only as 800 mg daily dose. All patients were limited to standard regimen, and the pre-treated patients were only included if they received the particular drug for the first time (first-use regimens). Observation period lasted from 1 July 2013 to 1 January 2016. Cox regression models were performed to identify predictors of modification and Kaplan-Meier estimates were used to calculate probabilities of modification.

**Results**: We analyzed 787 naïve patients and 1790 first-use regimens among 1590 pre-treated patients. Overall, ART was modified among 181 (23.0%) naïve patients and 330 (18.4%) individuals with first-use regimens, most of them in the first year. The overall probability of modification among the naïve patients rose from 20.0% at 1 and 30.8% at 2 years and from 17.8 to 30.2% among the pre-treated patients, respectively. Modifications of individual drugs are given in Figure 1 and Figure 2. Among the naïve patients taking RAL, DRV or other drugs showed a higher risk for modification compared to RPV, whereas in pre-treated patients, DGV showed a lower risk of modification (Table 1). Demographic and HIV-related factors were not associated with treatment modification with the exception of drug users, who had a higher risk of modification among pre-treated patients. Availability of more convenient treatment (37.0%) was the main reason for discontinuation within the naïve patients and, patients wish (19.4%) was the most cited reason for the pre-treated ones.

**Conclusion**: Much less treatment modification in individuals initiating ART with recently approved drugs which support a better tolerability and a more convenient profile also in a non-trial setting. However, there seems to be a difference between recently approved drugs in pre-treated patients, since we observed the lowest rate of modification in patients who received DGV. The low rate found for RPV may be attributed to strict selection of patients according to guidelines in regard to viral load.

**Figure 1:** Time to treatment modification by drugs.

**Figure 2:** Time to treatment modification by drugs.

**Abstract P121–Table 1:** Factors associated with treatment modification: multivariable Cox regression

	Drug-naïve patients	Multivariable Cox regression		First use of drug in pre-treated patients	Multivariable Cox regression	
	
	Number of patients with modification/all	HR	[95% CI]	Number of patients with modification/all	HR	[95% CI]
Drugs						
Dolutegravir	14/235	0.98	[0.43–2.23]	90/928	0.60	[0.43–0.84]
Elvitegravir	8/89	0.89	[0.35–2.26]	38/191	1.09	[0.73–1.65]
Raltegravir	36/83	5.71	[2.79–11.71]	63/154	2.07	[1.43–2.98]
Darunavir	60/152	4.50	[2.29–8.86]	77/224	1.63	[1.15–2.30]
Other	53/124	5.29	[2.68–10.44]			
Rilpivirine	10/104	1.00	[1.00–1.00]	62/293	1.00	[1.00–1.00]

# Sub article: P122 Integrase strand transfer inhibitors in the treatment of HIV-2 infection: report of 39 patients

**Published:** 2016-10-23

**Authors:** Filipa Sequeira, Sara Grazina, João Alves, Susana Peres, Fernando Borges, Ana Cláudia Miranda, Isabel Aldir, Kamal Mansinho

**Affiliations:** 1 E.P.E., Internal Medicine, Centro Hospitalar do Tâmega e Sousa, Penafiel, Portugal; 2 Infectious Diseases and Tropical Medicine, Centro Hospitalar de Lisboa Central – Hospital Egas Moniz, Lisboa, Portugal

## Abstract

**Introduction**: Few data are available on integrase strand transfer inhibitors (ISTI)-based regimens response among HIV-2 infected patients.

**Materials and methods**: Retrospective longitudinal study. Data on patients' demographic characteristics and ISTI response (clinical, immunological or virological) among HIV-2 infected patients were collected.

**Results**: Thirty-nine patients with HIV-2 infection were on ISTI-based regimens. Sixty-two percent were female. Seventeen patients were from Portugal, 15 from Guinea-Bissau, five from Cape Verde, one from Sao Tome and one from Spain. The median age at diagnosis was 46.5 years. The time between diagnosis of HIV-2 infection and ART initiation ranged from 1 week to 23 years (median of 5 years). The median CD4 count at diagnosis was 362.8 cells/mm^3^ (22.1%), and at ISTI initiation was 315.9 cells/mm^3^ (21.9%). The median follow-up after ISTI initiation was 2.7 years (min: 12 weeks, max: 8 years). Fourteen patients were naïve and 28 patients switched to ISTI. The reasons for switch were immune failure (n=13), cardiovascular risk (n=6), osteoporosis (n=2), nephropathy (n=4), intolerance (n=2) and HIV-1/2 infection (n=1). Thirty-one patients received RAL, and eight received DTG. Optimized background ARV regimens included DRV/r (n=10), LPV/r (n=8), SQV/r (n=5) and ATV/r (n=2) associated with two nucleoside reverse transcriptase inhibitors (NRTI). At week 12, 24 and 36 plasma HIV-2 RNA was undetectable in 92.3%, 97.2% and 94.8%, respectively, and median CD4 cell count was 362/mm^3^ (77–733), 506/mm^3^ (132–992) and 516/mm^3^ (25–989). One patient had to stop ISTI at week 36 because of immune failure due to ISTI resistance and died after 3 years.

**Conclusions**: Our series confirm the clinical effectiveness of ISTI in treatment-experienced and -naïve patients with HIV-2 infection when given with other ARVs to which the virus is susceptible. ISTI appears to perform well in treatment of HIV-2 patients as first regimen or after switch as therapy for a large proportion of patients when the first-line regimens may not have been sufficiently potent.

# Sub article: P123 Effect of probiotics on inflammation in the gut in HIV-infected individuals evaluated using PET/MR

**Published:** 2016-10-23

**Authors:** Caroline Arnbjerg, Karin Pedersen, Sofie Jespersen, Barbara Fischer, Helle Johannesen, Eva Fallentin, Adam Hansen, Andreas Kjær, Marius Trøseid, Susanne Dam Nielsen

**Affiliations:** 1 Department of Infectious Diseases, Rigshospitalet, University Hospital of Copenhagen, Copenhagen, Denmark; 2 Department of Clinical Physiology, Nuclear Medicine, Rigshospitalet, University Hospital of Copenhagen, Copenhagen, Denmark; 3 Department of Infectious Diseases, Oslo University Hospital, Oslo, Norway

## Abstract

**Introduction**: Microbial translocation in HIV-infected individuals has been associated with increased risk of non-AIDS comorbidity including cardiovascular complications. Initiation of cART often results in immune reconstruction in peripheral blood but does not lead to normalization of the gut-associated lymphoid tissue (GALT). The aim of this study was to investigate the effect of the probiotic strain *Lactobacillus rhamnosus* GG on microbial translocation both on local inflammation in the gut and on systemic inflammation.

**Methods**: The study was a prospective, clinical intervention trial and included 15 cART-naïve, and 30 cART-treated HIV-infected participants. All participants ingested probiotics (Dicoflor60^®^, Pharmaforce ApS, Denmark) in dose of 6 × 109 colony-forming units twice a day for a period of 8 weeks. Local inflammation was measured using fluoro-D-glucose positron emission tomography/magnetic resonance (FDG PET/MR) scans. Local inflammation in the bowel was assessed in five regions: terminal ileum, ascending, transverse, descending and sigmoid colon and rectum. Furthermore, fasting blood samples were obtained both at baseline and after 8 weeks of probiotics. Lipopolysaccharide (LPS), soluble inflammation markers of inflammation IL-6, IL-2, TNF-alfa and hsCRP as well as the CD4+ T-cell count were determined.

**Results**: Forty-five participants completed the study, 15 cART-naïve and 30 cART-treated participants, of which 15 participants were scanned using PET/MR before and after probiotics. On PET/MR, two out of five cART-naïve participants and 4 out of 10 cART-treated participants had evidence of decreasing inflammation on a global score. In terminal ileum, 4 out of 5 cART-naïve and 4 out of 10 cART-treated participants had decreasing inflammation (p=0.07). Among the cART-treated participants, concentration of LPS was found to increase (0.45–0.49 EU/mL, p=0.033). In contrast, no effect of LGG on markers of systemic inflammation in either cART-treated or cART-naïve HIV-infected participants was found. Finally, an increase in CD4+ T-cell count was found in the cART-treated group (659–697 cells/mL, p=0.029).

**Conclusion**: Using PET/MR to evaluate gut inflammation is feasible. The probiotic strain *Lactobacillus rhamnosus* GG did not have any beneficial effect on microbial translocation or systemic inflammation in HIV-infected individuals. However, PET/MR scans indicated a possible reduction of local inflammation. Future studies evaluating PET/MR scans as a method to assess the gut inflammation in HIV-infected individuals are warranted.

# Sub article: P124 Starting antiretroviral therapy for HIV at the first visit and early after inclusion into care: an observational study

**Published:** 2016-10-23

**Authors:** Josip Begovac, Šime Zekan, Snjezana Zidovec Lepej, Davorka Lukas

**Affiliations:** 1 Department of Infectious Diseases, University of Zagreb School of Medicine, Zagreb, Croatia; 2 Department of Molecular Diagnostics, University Hospital for Infectious Diseases, Zagreb, Croatia

## Abstract

**Introduction**: Croatia has a centralized system of care and all HIV-infected persons are treated at the University Hospital of Infectious Diseases (UHID) in Zagreb. All patients collect antiretroviral drugs from the hospital pharmacy at UHID. The centre at UHID is also the only centre in Croatia that provides psychosocial and adherence support to people living with HIV. Data on ART initiation at first clinic visit are limited [1,2]. We describe the characteristics of patients who start ART immediately and early after inclusion into care at UHID, and examine whether starting ART on the first visit (same-day starters) is equally successful as starting ART within the next 30 days (early starters).

**Materials and methods**: We included ART-naïve individuals aged 18 years or older, who entered care between January 2005 and December 2014 and started ART within 30 days of inclusion into care. Excluded were pregnant women and persons who were in HIV care elsewhere before entering care at UHID. We abstracted data from the electronic database. When ART was prescribed at the physician-patient visit the exact date of when ART was taken was recorded on the first follow-up visit. The primary outcome of the study was time to HIV1 RNA viral load <50 copies/mL, which was assessed by survival analysis. We also examined factors related to first visit ART initiation by logistic regression analysis.

**Results**: We studied 378 patients who met the eligibility criteria of whom 123 (32.5%) received ART at the first visit at UHID (Table 1).

The median time of initiation of ART in the group of early starters was 5 (Q1–Q3, 2–14) days. By 12 months, the probability of achieving an HIV1 RNA viral load <50 copies/mL was 87.7% (95% CI 81.0–92.9%) and 89.3% (95% CI, 85.0–92.8%) in the same-day starters versus early starters, respectively (Figure 1).

On multivariable analysis, the following factors were related to starting ART at the first visit: not having clinical AIDS (OR 2.96; 95% CI 1.77–4.94), a CD4 cell count ≤350/mm^3^ (OR 4.28; 95% CI 1.88–9.72), being MSM (OR 2.65; 95% CI 1.51–4.67), receiving 2NRT+1PI (OR 1.92; 95% CI 1.17–3.14) and being integrated into care after 30 days (OR 2.63; 95% CI 1.37–5.08).

**Conclusions**: In this HIV-infected patient population with predominantly advanced immunosuppression at entry into care, same-day ART was as successful as therapy given within 1 month after the first physician-patient visit.

**Abstract P124–Table 1:** Main characteristics of 378 patients who started antiretroviral therapy within one month after inclusion into care in the period 2005 to 2014

	ART initiation at first physician-patient visit	ART initiation within 30 days after the first visit	Total	
Variables	N=123	N=255	N=378	p
Age, years	39.2 (31.8–48.0)	36.4 (30.3–43.6)	38.2 (31.4–46.8)	0.043
Male gender	115 (93.5)	225 (88.2)	340 (89.9)	0.111
Residence outside Zagreb	81 (65.9)	156 (61.2)	237 (62.7)	0.378
Mode of transmission				
Men who have sex with men (MSM)	98 (79.7)	173 (67.8)	271 (71.7)	0.017*
Male to female or female to male	22 (17.9)	64 (25.1)	86 (22.8)	
Persons who inject drugs	1 (0.8)	7 (2.7)	8 (2.1)	
Unknown	2 (1.6)	11 (4.3)	13 (3.4)	
Integrated into care within 30 days†	97 (78.9)	233 (91.4)	330 (87.3)	<0.001
Period				0.194
2005–2009	37 (30.1)	94 (36.9)	131 (34.7)	
2010–2014	86 (69.9)	161 (63.1)	247 (65.3)	
Had clinical AIDS	34 (27.6)	117 (45.9)	151 (39.9)	<0.001
CD4 cell count per mm3	93 (30–255)	125 (47–260)	102 (33–255)	0.193
Categories of CD4 cell count, per mm3				0.006
<100	54 (43.9)	133 (52.2)	187 (49.5)	
100–350	59 (48.0)	82 (32.2)	141 (37.3)	
>350	10 (8.1)	40 (15.7)	50 (13.2)	
Log HIV1 RNA, copies/mL	5.5 (4.9–5.9)	5.2 (4.8–5.8)	5.4 (4.9–5.9)	0.051
HIV RNA >200,000 copies/mL	54 (43.9)	148 (58.0)	202 (53.4)	0.010
Initial ART type				
2NRT + NNRT	69 (56.1)	170 (66.7)	239 (63.2)	0.018‡
2NRT + PI	50 (40.7)	71 (27.8)	121 (32.0)	
2NRT + II	3 (2.4)	7 (2.7)	10 (2.6)	
Other	1 (0.8)	7 (2.7)	8 (2.1)	
Had hepatitis C antibody	3 (2.4)	8 (3.1)	11 (2.9)	0.761
Had hepatitis B surface antigen	5 (4.1)	13 (5.1)	18 (4.8)	0.659
Causes of failure				0.966
Virological failure§	14 (11.4)	25 (9.8)	39 (10.3)	
Died	4 (3.3)	8 (3.1)	12 (3.2)	
Lost to follow-up	1 (0.8)	2 (0.8)	3 (0.8)	
Stopped ART	0 (0.0)	2 (0.8)	2 (0.5)	

Values on the table are frequencies or medians with Q1–Q3.

* MSM versus non-MSM

† time from HIV test to first CD4 count measurement

‡ comparison of NNRT versus PI

§ at 12 months.

**Abstract P124–Figure 1:** Kaplan-Meier estimates of proportion of patients with HIV RNA < 50 copies/ml according to time of initiation antiretroviral therapy.

## References

[1] Rosen S, Maskew M, Fox MP, Nyoni C, Mongwenyana C, Malete G (2016). Initiating antiretroviral therapy for HIV at a patient's first clinic visit: the RapIT randomized controlled trial. PLoS Med.

[2] Pilcher CD, Hatano HH, Dasgupta A, Jones D, Torres S, Calderon F (2015). Providing same day, observed ART to newly diagnosed HIV+ outpatients is associated with improved virologic suppression. [Abstract WEAD0105 LB.] 8th International AIDS Society Conference on HIV Pathogenesis, Treatment, and Prevention (IAS 2015), Vancouver, Canada..

# Sub article: P125 Psychometric evaluation of a new individualised condition-specific quality of life questionnaire for HIV (HIVDQoL)

**Published:** 2016-10-23

**Authors:** Jacquelyn Romaine, Miranda Murray, Clare Bradley

**Affiliations:** 1 Health Psychology Research Unit, Royal Holloway, University of London, London, UK; 2 Global Health Outcomes, ViiV Healthcare Ltd, London, UK

## Abstract

**Introduction**: Given recent developments in HIV treatment it is important that, in addition to health status and symptoms, a more holistic view of the impact of HIV on quality of life (QoL) is obtained. The HIV Dependent Quality of Life (HIVDQoL) questionnaire is an individualized condition-specific QoL questionnaire based on a template developed by CB for the ADDQoL [1] (Audit of Diabetes-Dependent QoL) and DQoL measures for other conditions [2]. Qualitative work to design the item content of the HIVDQoL is reported elsewhere [3]. This abstract reports the psychometric evaluation of the HIVDQoL.

**Methods**: The study employed a survey design with participants (N=255) recruited from the UK (N=128) and the US (N=127), via the internet, by Opinion Health. Mean age of participants was 49 years (SD 10.64), mean time since diagnosis was 15 years (SD 9.43), 203 participants were male and 49 were female. Participants chose to complete and return the questionnaire individually (via post) or with a researcher (via phone). The HIVDQoL included two overview items which measure generic “present QoL” and “HIV-specific QoL” and 26 items that measure the impact of having HIV on specific aspects of life (e.g. family, physical appearance) and measures the importance of these aspects of life for QoL. “Not applicable” options are provided for items that do not apply to everyone (e.g. work). Exploratory factor analysis (EFA) was used to examine scale structure and reliability using Cronbach's alpha coefficient of internal consistency.

**Results**: EFA was conducted in two stages. Principal components analysis, eigenvalues >1, scree plot and parallel analysis guided the number of factors to extract, and principal axis factoring was used to determine the underlying structure. The analysis revealed a one-factor structure which included 24 of the 26 items. Two items were dropped (religious/spiritual life; having children) due to low communality and low loadings. The 24 items explained 40% of the variance. The factor matrix revealed the lowest loading item loaded at 0.442 and included seven excellent items (loading >0.71), five very good items (>0.63), six good items (>0.55) and five fair items (>0.45). Reliability was strong: alpha=0.939 for the 24 items.

**Conclusions**: The HIVDQoL is here shown to have sound psychometric properties including excellent reliability. It is suitable for use in clinical trials, other research and in routine clinical practice to evaluate the impact of HIV and its treatment on QoL with a view to identifying treatments that optimize QoL.

## References

[1] Bradley C, Todd C, Gorton T, Symonds E, Martin A, Plowright R (1999). The development of an individualized questionnaire measure of perceived impact of diabetes on quality of life: the ADDQoL. Qual Life Res.

[2] Peach G, Romaine J, Wilson A, Holt PJ, Thompson MM, Hinchliffe RJ (2016). Design of new patient-reported outcome measures to assess quality of life, symptoms and treatment satisfaction in patients with abdominal aortic aneurysms. Brit J Surg.

[3] Romaine J, Bayfield J, Plowright R, Murray M, Bradley C (2015). Design of the HIV Dependent Quality of Life (HIVDQoL) questionnaire and HIV Symptom Rating Questionnaire (HIVSRQ). Qual Life Res.

# Sub article: P126 Individualized NRTI-sparing antiretroviral regimens in a real-world clinical setting maintain virologic efficacy with significant cost reduction

**Published:** 2016-10-23

**Authors:** Sari Arponen, Alejandra Gimeno-García, Carmen Montero-Hernández, Laura Esteva, Marta Blasco, Elsa Gaspar-García, M Jose García-Navarro

**Affiliations:** 1 Servicio de Medicina Interna, Hospital Universitario de Torrejón, Torrejón de Ardoz, Spain; 2 Servicio de Farmacia, Hospital Universitario de Torrejón, Torrejón de Ardoz, Spain

## Abstract

**Introduction**: NRTI-sparing regimens have been proposed as a strategy to avoid toxicities associated with these drugs. These therapies may have an additional financial benefit. Some clinical trials have explored these NRTI-sparing mono- and dual therapies, and there is growing evidence from real-world practice in this issue.

**Materials and methods**: We retrospectively reviewed the medical records of the HIV+ patients treated with a mono- or dual therapy in our hospital. Treatment changes and simplifications were made by the responsible clinician according to patients' clinical characteristics, antiretroviral therapy (ART) history and toxicities. Clinical, virologic and immunologic data were collected, as well as lipid profile and renal function. Additionally the cost of each regimen and the saving associated with the new therapy were calculated.

**Results**: Twenty-nine patients (51.7% women) were analyzed (14% of all patients with ART in our hospital). The median age was 48 years (IQR 27–72). The median duration of infection was 17 years (IQR 5–25.5), with a median duration from the start of their first ART of 9 years (IQR 3.7–16.6). The median time of undetectability was 8.2 years (IQR 3.4–16.2). All patients were undetectable at the time of initial analysis. 27.6% had clinical category C. Initially 19 (65.5%) patients were receiving as NRTI backbone TDF+FTC, 6 (20.7%) ABC+3TC, 1 patient TDF and 4 patients had a previous NRTI-sparing regimen. The therapy was changed in 25 patients: eight (27.2%) received ritonavir- or cobicistat-boosted DRV with 3TC, 10 (34.4%) patients received ATV/r with 3TC and four patients were treated with a DRV/r monotherapy. Other therapies were DLG+3TC (one), ETR+DRV (three), RAL+DRV (one) and RAL+ETR (two). At 6 months of follow-up, there were no virologic failures. The reason for the change was renal toxicity in 12 patients (41.4%), bone disease in 6 (20.7%) and unknown in 5 cases (17.2%). There were no significant changes in the number of CD4, total cholesterol, HDL, LDL, TG and renal function. There was a significant improvement in CD4% (+2% at 6 months, 95% CI 0.02–3.83). An average monthly saving of €200 (29%) per patient was achieved.

**Conclusions**: In a real-world clinical setting, individualized NRTI-sparing antiretroviral regimens, even in patients with a long evolution of HIV infection, maintained virologic efficacy. Additionally, these strategies reduced the cost of treatment.

# Sub article: P127 A retrospective analysis of time to non-detectable HIV viral load in fixed dose combination anti-retroviral therapy in HIV-1 patients enrolled in the Mater Misericordiae University Hospital Infectious Diseases (MMUH-ID) cohort

**Published:** 2016-10-23

**Authors:** Marc Grant-Freemantle, Patrick Mallon, Geoff McCombe, Alan Macken, Willard Tinago, Gordana Avramovic, Jane O'Halloran, Aoife Cotter, Gerard Sheehan, John Lambert

**Affiliations:** 1 School of Medicine, University College Dublin, Dublin, Ireland; 2 HIV Molecular Research Group, School of Medicine, University College Dublin, HIV Molecular Research Group, Dublin, Ireland; 3 Infectious Diseases, Mater Misericordiae University Hospital, Dublin, Ireland

## Abstract

**Introduction**: Fixed Dose Combinations (FDCs) are popular HIV treatment options due to simpler dosing regimens and improved patient adherence leading to better health outcomes. Emerging evidence suggests integrase inhibitors (INSTI) such as dolutegravir (DTG) and elvitegravir (EVG) may be able to achieve non-detectable (ND) viral loads (VL) more rapidly than established treatments, and benefit certain patient groups such as pregnant women. The study investigated if Triumeq and Stribild, two FDCs containing DTG and EVG respectively, induce viral suppression more rapidly than other FDCs not containing integrase inhibitors.

**Materials and methods**: The MMUH-ID cohort collects retrospective and prospective follow up information on demographics, diagnostics, HIV acquisition risks, clinical assessments and medications (history, regimens, drug class, dose, frequency, start and stop dates). HIV RNA results are obtained weekly from the National Virus Reference Laboratory (NVRL) and are manually captured into the cohort database. Patients commenced on FDC ART of either Atripla (efavirenz/tenofovir/emtricitabine), Eviplera (emtricitabine/rilpivirine/tenofovir), Stribild (cobicistat/EVG/emtricitabine/tenofovir) or Triumeq (DTG/abacavir/lamivudine) from July 2008 to April 2016 were reviewed to determine when they reached the primary end-point of ND VL - two consecutive VL <50 copies/mL. Time to ND VL was measured from initiation of an FDC. Kaplan-Meier curves and the log-rank test were used to compare time to ND VL between FDC groups.

**Results**: A total of 124 patients were included in the analysis (84.7% male, median age 38 years). Seventy-three patients (58.9%) were commenced on Atripla, with 56 reaching ND VL with a median time to ND VL of 5.7 (4.3–8.0) months after treatment initiation. 33 patients (26.6%) were commenced on Eviplera with 29 reaching ND VL with a median time of 7.34 (6.0–16.1) months. Thirteen patients (10.5%) were commenced on Stribild with eight reaching ND VL with a median time of 3.41 (1.8–4.6) months. Five were commenced on Triumeq (4%) with four reaching ND VL with a median time of 3.67 (2.52-Na) months. Attaining ND VL was significantly different between the FDC ART groups (log-rank=11.2, p=0.003), with lesser time to ND VL attainment in subjects initiated on Stribild and Triumeq.

**Conclusions**: FDCs containing INSTIs offer a promising approach to rapidly reducing VL in HIV-1 infected patients. INSTIs are becoming the standard of care in many clinical settings and should especially be prioritized in situations in which there is a clinical need for rapid viral suppression.

# Sub article: P128 Antiretroviral treatment changes due to treatment of hepatitis C with direct-acting antivirals in co-infected patients

**Published:** 2016-10-23

**Authors:** Oscar Luis Ferrero, Sofía Ibarra, Iñigo López-Azkarreta, Mireia de la Peña, Miriam López-Martínez, Josu Mirena Baraia-Etxaburu, Josefina López de Munain, M Mar Cámara, Zuriñe Zubero, Josefa Muñoz

**Affiliations:** 1 Department of Infectious Diseases, Hospital Universitario Basurto, Bilbao, Spain

## Abstract

**Introduction**: Since the advent of new direct-acting antivirals (DAAs) for the treatment of hepatitis C virus (HCV), this has become a priority for many of our patients. The disadvantages are the numerous interactions between DAAs and antiretrovirals used by our co-infected patients that force clinicians to make changes in the ART.

**Material and methods**: The Basque Health System (Osakidetza) has adopted guidelines to treat HCV that prioritize the use of Abbvie drugs (ombitasvir–paritaprevir–ritonavir with or without dasabuvir). Since then, we have performed 219 treatments in co-infected patients. We have analyzed changes in ART in patients during treatment with DAAs.

**Results**: Two hundred and nineteen treatments with DAAs have been started in co-infected patients (123 completed, 96 still ongoing). Seventy-nine of the 219 patients who started HCV treatment have not changed their ART, while 140 required changes to avoid interactions. The most frequent changes according to antiretroviral family have been efavirenz (EFV) to raltegravir (RAL) (37) and rilpivirine (RPV) (20) and lopinavir (LPV) to darunavir (DRV) (17) and RAL (9). Changes between NRTI have been rare (five). After analyzing the 123 episodes that have completed treatment, we have observed that 45 (36.5%) have returned to prior ART, 35 (28.4%) have continued with the modified ART (of which 17 were based on EFV and 15 LPVr), in 10 (8.1%) adjustments have been made to avoid toxicities (seven) or simplify the ART (three) and in 33 cases (26.8%) there has been no need to make any changes. Regarding patients in PI-monotherapy (24) during treatment with DAAs, 17 have been changed (11 to triple therapy and six to other PI). Nineteen patients in PI-monotherapy (14 LPVr and five DRVr) have finished HCV treatment and 17 have returned to their previous regimen.

**Conclusions**: Treatment of co-infected patients with DAAs involves in many cases, changes in ART regimen to avoid interactions (in our case 64%). Most changes occur to new antiretrovirals with better tolerance profile and less interactions (RAL and RPV). Once treatment with DAAs is completed, most of the patients return to their initial ART although a significant percentage (near 30% in our series) prefers the modified ART, and in some patients adjustments in ART are made to improve the profile toxicity or simplify the regimen (8%). Virtually, all patients in PI-monotherapy return to it although many require changes during treatment with DAAs.

# Sub article: P129 The use of protease inhibitors in patients with blips is associated with virologic failure

**Published:** 2016-10-23

**Authors:** Eduardo Sanchez-Brown, Luis Enrique Soto-Ramirez

**Affiliations:** 1 Infectious Diseases, Instituto Nacional de Ciencias Medicas y Nutrición Salvador Zubiran, Mexico City, Mexico

## Abstract

**Introduction**: There is conflicting evidence whether blips are associated with virologic failure or rebound, and it has not been determined if some demographic, virologic and treatment factors in patients with blips are associated with these adverse outcomes. The main objective of this study was to determine which factors are associated with virologic failure in patients with blips.

**Materials and methods**: Retrospective, observational cohort study of patients enrolled in the HIV clinic of a hospital in Mexico City who presented with at least one blip episode between 2004 and 2012. Blip was defined as a single detectable viral load above the limit of quantitation but below 1000 copies/mL. Patients with blips were classified in two groups: failure group and suppressed group. We excluded all cases who failed to a treatment regime different from the one used at the time of the blip in the failure group. Relative risks for predictors of virologic failure were calculated, survival curves were generated to compare the rates of virologic failure according to drug history and differences were tested for statistical significance using the generalized Wilcoxon test.

**Results**: Of a total 1876 patients, 414 (22.06%) presented with at least one blip episode. Three hundred and nineteen were randomly selected for this study. Sixty-nine (21.6%) of the 319 met the definition of virologic failure (two consecutive viral loads >50 copies/mL at least 30 days apart). For statistical analysis, we used 51 cases with virologic failure and 229 with no failure (21 excluded due to a follow-up shorter than the mean time to failure in the Failure Group). The three factors associated with virologic failure were being under 20 years of age at the time of HIV diagnosis (RR 2.31 [1.22–4.38], p=0.01), overall use of protease inhibitors (RR 5.84 [2.17–15.72], p<0.0001) and use of protease inhibitor, at the moment of blip/failure (RR 2.95 [1.72–5.08], p<0.0001). Those who were on PI-based regimes at the moment of failure were more likely to achieve virologic failure earlier on than those who were not taking a PI-based regime (p=0.027).

**Conclusions**: Use of protease inhibitors and being under 20 years of age at the time of diagnosis were associated with virologic failure in patients with blips. The presence of blips may be used as a predictor of virologic rebound in those cases. While adherence can be an important determinant of failure, the forgiveness of the PI would be against that.

# Sub article: P130 Baseline characteristics of the TRIUMPH and DOL-ART cohorts: use of Triumeq^®^ (DTG/ABC/3TC) or other DTG-based ART in routine clinical care in Germany

**Published:** 2016-10-23

**Authors:** Thomas Heuchel, Nils Postel, Markus Bickel, Jürgen Brust, Stefan Scholten, Christian Schulz, Christoph Stephan, Ulrich Bohr, Heribert Hillenbrand, Tobias Glaunsinger, Albrecht Stoehr, Bernd Westermayer, Alexandra Wigger, Daniel Lüftenegger, Ravi Kumar Walli

**Affiliations:** 1 Medcenter, Chemnitz, Germany; 2 prinzmed, Munich, Germany; 3 Infektiologikum, Frankfurt, Germany; 4 Mannheimer Onkologie Praxis, Mannheim, Germany; 5 Praxis Hohenstaufenring, Cologne, Germany; 6 Universitätsklinikum, Magdeburg, Germany; 7 HIV-Center, Universitätsklinikum, Frankfurt am Main, Germany; 8 Praxiszentrum Kaiserdamm, Berlin, Germany; 9 Praxis City Ost, Berlin, Germany; 10 Praxis Prenzlauer Berg, Berlin, Germany; 1 IFI-Institute, Hamburg, Germany; 2 GlaxoSmithKline, Munich, Germany; 3 ViiV Healthcare, Munich, Germany

## Abstract

**Introduction**: TRIUMPH and DOL-ART are two consecutive, prospective and observational German cohort studies in HIV-1 infected patients initiated on integrase inhibitor-based ART with dolutegravir. DOL-ART included patients already receiving Tivicay^®^ (DTG) in combination ART. Recruitment of TRIUMPH started after DOL-ART recruitment was completed and included patients receiving Triumeq^®^, a one-pill regimen consisting of DTG/ABC/3TC.

**Methods**: In both cohorts, patients are followed in routine clinical care for 3 years with respect to monitoring measures, efficacy and safety parameters. Here, we compare the baseline characteristics of the cohorts in terms of demographics, HIV-related variables, comorbidities and comedication.

**Results**: TRIUMPH included 398 patients (32 centres), DOL-ART included 411 patients (37 centres). Characteristics of the two study populations are shown in Table 1. Patients of the TRIUMPH cohort were less frequently and less intensively pre-treated than patients in DOL-ART. In pre-treated patients, the reasons for switch (multiple responses permitted) to DTG-based ART were as follows (TRIUMPH vs DOL-ART): treatment simplification (57.0% vs. 30.1%), side effects on previous ART (24.3% vs. 31.7%), patient wish (30.6% vs. 24.7%), comorbidities or concomitant medication (11.1% vs. 12.2%) or virologic failure (1.3% vs. 9.6%). In DOL-ART, the majority of patients (84.9%) received standard triple therapy including either Kivexa^®^ or Truvada^®^. The prevalence of comorbidities was lower in TRIUMPH than in DOL-ART (>2 comorbidities 3.5% vs. 10.5%) with differences seen in both ART-naïve and pre-treated patients. In ART-naïve patients, most common comorbidity (≥10%) was depression (14.7% vs. 16.2%). In pre-treated patients, most common comorbidities (≥10%) were depression (23.4% vs. 33.3%), hypertension (11.5% vs. 18.6%) and cardiovascular diseases (6.4% vs. 11.2%). In TRIUMPH (DOL-ART), 18.4% (24.2%) of ART-naïve patients and 34.5% (36.2%) of pre-treated patients received concomitant medication other than ART. In ART-naïve patients, most common concomitant medication (≥10%) included prophylaxis/treatment of opportunistic infections 3.7% (10.1%). In pre-treated patients, most common concomitant medication included antihypertensives 14.9% (19.2%) and antidepressants 11.1% (11.9%).

**Conclusion**: The TRIUMPH and DOL-ART cohorts showed that standard triple therapy consisting of DTG plus two NRTI as single- or as multi-tablet regimen are used for both ART-naïve and pre-treated patients. “Simplification of ART” and “side effects on previous ART” were the main reasons for switch to Triumeq^®^ or DTG+X, respectively. Based on CDC classification and comorbidities, the burden of disease was somewhat lower in TRIUMPH than in DOL-ART. The majority of patients were switched from suppressive ART demonstrating an obvious need for treatment optimization in clinical practice.

**Abstract P130–Table 1:** Patient characteristics before introduction of DTG-containing ART

	TRIUMPH (N = 398)		DOL-ART (N = 411)	
	
	ART naïve (N = 163)	Pre-treated (N = 235)	ART-naïve (N = 99)	Pre-treated (N = 312)
Sex, male, N (%)	155 (95.1)	203 (86.4)	89 (89.9)	268 (85.9)
Age, years, median (range)	39 (29–48)	45 (35–52)	39 (32–48)	46 (38–53)
CDC stage C, N (%)	9 (5.5)	54 (23.0)	10 (10.1)	86 (27.6)
BL HIV RNA, log copies/mL, median (IQR)	4.4 (3.9–4.9)	1.7 (1.7–1.7)	4.7 (4.1–5.1)	1.7 (1.7–1.8)
BL HIV-1 RNA ≥100,000 copies/mL, N (%)	30 (18.4)	–	29 (29.3)	–
BL HIV-1 RNA ≥500,000 copies/mL, N (%)	6 (3.7)		8 (8.1)	
BL HIV-1 RNA 50 copies/mL, N (%)	–	198 (84.3)	–	224 (71.8)
BL CD4 cell count, cells/µL, median (IQR)	450 (282–597)	602 (434–834)	377 (245–549)	584 (422–800)
BL CD4 cell count, 200/µL, N (%)	22 (13.5)	8 (3.4)	18 (18.2)	23 (7.4)
>2 previous regimens, N (%)	–	48 (20.4)	–	135 (43.3)
Comorbidities, N (%)	49 (30.1)	123 (52.3)	35 (35.4)	193 (61.9)
Depression, N (%)	24 (14.7)	55 (23.4)	16 (16.2)	104 (33.3)
Hypertension, N (%)	12 (7.4)	27 (11.5)	6 (6.1)	58 (18.6)
Cardiovascular diseases, N (%)	2 (1.2)	15 (6.4)	3 (3.0)	35 (11.2)
Chronic HCV infection, N (%)	6 (3.7)	20 (8.5)	5 (5.1)	20 (6.4)
Pulmonary disease, N (%)	4 (2.5)	17 (7.2)	6 (6.1)	22 (7.1)
Dyslipidaemia (requiring treatment), N (%)	0 (0.0)	10 (4.3)	0 (0.0)	30 (9.6)
Diabetes mellitus, N (%)	4 (2.5)	13 (5.5)	4 (4.0)	13 (4.2)
Concomitant medication, N (%)	30 (18.4)	81 (34.5)	24 (24.2)	113 (36.2)
Antihypertensives, N (%)	13 (8.0)	35 (14.9)	6 (6.1)	60 (19.2)
Antidepressants, N (%)	6 (3.7)	26 (11.1)	5 (5.1)	37 (11.9)
Ca^2 +^/Fe^2 +^-containing supplements/multivitamin, N (%)	2 (1.2)	12 (5.1)	3 (3.0)	10 (3.2)
Prophylaxis/treatment of opportunistic infection, N (%)	6 (3.7)	7 (3.0)	10 (10.1)	13 (4.2)
Metformin, N (%)	0 (0.0)	4 (1.7)	1 (1.0)	5 (1.6)

# Sub article: P131 Long-term use of darunavir/r QD containing regimens in daily practice in Belgium: retrospective observational cohort data of 1701 HIV patients

**Published:** 2016-10-23

**Authors:** Stéphane De Wit, Eric Florence, Linos Vandekerckhove, Bernard Vandercam, Jean-Christophe Goffard, Eric Van Wijngaerden, Michel Moutschen, Remy Demeester, Hilde Piryns, Pushpike Thilakarathne

**Affiliations:** 1 Infectious Diseases, Saint-Pierre University Hospital, Brussels, Belgium; 2 Infectious Diseases, Institute of Tropical Medicine, Antwerp, Belgium; 3 Infectious Diseases, Ghent University Hospital, Ghent, Belgium; 4 Infectious Diseases, Saint-Luc University Hospital, Brussels, Belgium; 5 Infectious Diseases, University Hospital Erasme, Brussels, Belgium; 6 Infectious Diseases, University Hospital Leuven, Leuven, Belgium; 7 Infectious Diseases, Liège University Hospital, Liège, Belgium; 8 Infectious Diseases, University Hospital Charleroi, Charleroi, Belgium; 9 Janssen-Cilag NV, Medical Affairs, Beerse, Belgium; 10 Department of Statistics, Janssen, Beerse, Belgium

## Abstract

**Objectives**: To describe the use of darunavir/ritonavir (DRV/r) QD in daily practice in Belgium.

**Methods**: *Design*: Observational, non-interventional, non-comparative, retrospective, multicentre cohort study. Data were collected from existing databases from eight AIDS reference centres in Belgium. *Inclusion*: HIV-1 infected adults (treatment-naïve or experienced patients), who received at least one dose of DRV/r 800 mg/100 mg QD in various combinations from 1 January 2010 on, with a minimum follow-up of 6 months. Primary endpoints: time to treatment discontinuation (using Kaplan-Meier estimates), rate and reasons for discontinuation of DRV/r QD containing regimen. Secondary endpoints: virologic suppression, change from baseline in CD4 count, effect of DRV/r on kidney function.

**Results**: Data from 1701 HIV-infected patients were collected and analyzed. Baseline characteristics: overall, 66.5% were male, mean age of 42.9 years (±11.1), mean CD4 count 441.8 cells/mm^3^ (±287.1) and mean CD4 nadir 248.9 cells/mm^3^ (±177.3); 33.1% of patients were treatment naïve (44.2% with baseline viral load (BL VL) ≥100,000 copies/mL) and 66.9% were ART-experienced patients (48.5% with BL VL <50 copies/mL). Tenofovir-emtricitabine was used as backbone in 72.6% of naïve patients and 52.2% of experienced patients. Overall, median follow-up period (Q1–Q3) on DRV/r QD was 2.45 (1.50–3.34) years. The probability to remain on treatment (95% CI) was 87.0% (85.2–88.5%) for the first year and 78.9% (76.7–80.9%) for the second year and 69.1% (66.3–71.7%) for the third year (Figure 1).

Four hundred and fifty-nine patients (27.0%) discontinued treatment with DRV/r QD. The main reasons were simplification (6.7%), adverse events (6.9%, of which 4.0% for GI problems), patient's or physician's decision, drug-drug interactions or inclusion in a clinical trial. Reason for discontinuation was missing in 3.1%. Discontinuation for lack of efficacy was noted in only 0.8%. CD4 count increased by 164.4 cells/mm^3^ from baseline to end of follow-up. Lipids (TG, TChol, LDL-C, HDL-C) and renal (eGFR) parameters remained stable throughout the period. At the end of the follow-up period 81% of patients had HIV RNA <50 copies/mL.

**Conclusion**: This retrospective cohort analysis of patients on darunavir/r QD in Belgium confirms the long-term efficacy and good tolerability of DRV/r QD in real-life setting. The rate of discontinuation of DRV/r QD in daily practice is low and rarely due to lack of efficacy. No unexpected adverse events were reported.

# Sub article: P132 Assessment of the results at 96 weeks of a multicentric Portuguese cohort of patients treated with emtricitabine/tenofovir/rilpivirine

**Published:** 2016-10-23

**Authors:** Francisco Almeida, Filipa Sequeira, Maria Isabel Casella, Margarida Mota, Rosário Serrão, Mari Mesquita, Ana Teresa Goes, Tiago Teixeira, Carmela Piñeiro

**Affiliations:** 1 Infectious Diseases, Centro Hospitalar São João, Porto, Portugal; 2 Internal Medicine, Centro Hospitalar Tâmega e Sousa, Penafiel, Portugal; 3 Infectious Diseases, Centro Hospitalar de Setúbal, Setúbal, Portugal; 4 Internal Medicine, Centro Hospitalar Vila Nova de Gaia/Espinho, Vila Nova de Gaia, Portugal; 5 Internal Medicine, Hospital do Litoral Alentejano, Santiago do Cacém, Portugal

## Abstract

**Introduction**: Rilpivirine is a second-generation non-nucleoside reverse transcriptase inhibitor (NNRTI), included in Eviplera^®^. Clinical trials have demonstrated its effectiveness and relatively favourable profile of adverse effects compared to first-generation NNRTIs.

**Objective**: To assess the evolution of different indicators in naïve and experienced patients on ART who were started on FTC/TDF/RPV.

**Methods**: Retrospective cohort study of patients treated with FTC/TDF/RPV for more than 2 years in four Portuguese hospitals. We evaluated demographic characteristics, reasons for FTC/TDF/RPV use, adverse events and analytical parameters. In patients who switched to this regimen due to adverse effects, resolution of symptoms was evaluated.

**Results**: Two hundred and ninety-eight patients were included. Two hundred and twenty-six (76%) were male with a mean age of 46.6 years. Forty-five patients (15%) were previously ART naïve, 69 patients were under PI-containing regimens (27.2%), 183 patients under NNRTI-containing regimens (72.3%) and one under an INSTI-containing regimen (0.3%). Reasons for switching to FTC/TDF/RPV were dyslipidaemia in 66 patients (26.1%), central nervous system toxicity in 71 (28.1%), other toxicities in 12 (4.7%), therapeutic simplification in 62 (24.5%) and other reasons in 42 (16.6%). At 96 weeks, median CD4+ lymphocyte count increased (525 vs. 633 cells/mm^3^, p<0.001). Viral load was detectable in 43 patients (17%) at baseline and in seven patients (2.7%) after 96 weeks (p<0.001), of which only one had confirmed virological failure. Liver profile was unchanged during follow-up in the global sample, but the 67 patients (22.5%) who had chronic hepatitis B or C had a statistically significant improvement of both median alanine aminotransferase (ALT) (38 vs. 25.5 UI/L, p=0.001) and aspartate aminotransferase (AST) (33 vs. 28 UI/L, p=0.009). In the global sample, there was a statistically significant (p<0.001) decrease of median total cholesterol (185 vs. 175 mg/dL), LDL (111 vs. 107 mg/dL) and tryglicerides (115 vs. 98 mg/dL). This effect was mostly evident in patients who switched to FTC/TDF/RPV due to dyslipidaemia. There was a slight but statistically significant increase in creatinine values during the follow-up (p<0.0001), without clinical relevance. All patients who switched due to neuropsychiatric toxicity reported improvement of symptoms. During this time, nine patients (3%) stopped FTC/TDF/RPV due to virological failure (nine), NNRTI-related toxicity (one), other toxicity (four) or undisclosed reasons (one).

**Conclusions**: In this cohort, FTC/TDF/RPV showed good tolerability, a low rate of adverse effects and favourable lipid and hepatic profiles, mostly in patients with chronic hepatitis or previous dyslipidaemia. Immunological and virological efficacy was maintained during 96 weeks follow-up.

# Sub article: P133 The DOL-ART cohort: providing evidence from real-world data – use of dolutegravir-based regimens in routine clinical care in Germany

**Published:** 2016-10-23

**Authors:** Nils Postel, Markus Mueller, Christoph Wyen, Juergen Brust, Albrecht Stoehr, Tobias Glaunsinger, Stefan Scholten, Michael Rausch, Bernd Westermayer, Martina Herrmann, Ravi Kumar Walli

**Affiliations:** 1 prinzmed, Munich, Germany; 2 Gemeinschatfspraxis, Stuttgart, Germany; 3 Praxis am Ebertplatz, Cologne, Germany; 4 Mannheimer Onkologie Praxis, Mannheim, Germany; 5 IFI-Institute, Hamburg, Germany; 6 Praxis Prenzlauer Berg, Berlin, Germany; 7 Praxis Hohenstaufenring, Cologne, Germany; 8 Ärztezentrum Nollendorfplatz, Berlin, Germany; 9 GlaxoSmithKline, Munich, Germany; 10 ViiV Healthcare, Munich, Germany

## Abstract

**Introduction**: DOL-ART is a prospective, 3-year, German non-interventional study (NIS) initiated 2 months after EMA approval of dolutegravir (DTG, Tivicay^®^), to understand how the real-world experience with the drug compares with that from clinical trials.

**Methods**: HIV-infected patients enrolled into the study had to be on a DTG-based ART for ≥4 weeks. Primary and secondary objectives of DOL-ART comprise the evaluation of monitoring measures in routine clinical care of these patients, effectiveness and safety of DTG-based ART: frequency and type of monitoring measures (including laboratory tests and referrals to specialists), virologic effectiveness and the incidence of adverse drug reactions (ADRs).

**Results**: N=411 patients were included in DOL-ART between March and May 2014: 87% males, median age 45 years (IQR 36–52), 23% with history of AIDS; 76% pre-treated. Of ART-naïve patients, 18% had <200 CD4/µL, 29% >100,000 HIV RNA copies/mL; of pre-treated patients, 72% had <50 HIV RNA copies/mL; 85% of the study population received triple therapy consisting of DTG plus either TDF/FTC (45%) or ABC/3TC (40%); relevant comorbidities and concomitant medication were documented in 55% and 33% of patients, respectively. Median observation time until data cut was 15.8 months (IQR 15.2–16.8), with 86.4% of patients remaining under follow-up. Serum chemistry, blood count and HIV RNA/CD4 cell controls represented the overall majority of the measures (75.9%). Median number of monitoring measures per patient-year was 13.7 (IQR 10.5–17.4), in particular 14.9 (10.6–18.5) in ART-naïves and 13.6 (10.3–17.2) in pre-treated, 13.6 (10.7–17.4) in patients aged ≤50 years and 13.8 (10.2–17.2) in patients >50 years of age. Urine and microbiology tests accounted for 10.2% and 6.9% of the other measures, respectively; referrals to specialists (7.0%) were documented in 57.2% of patients (53.5% of ART-naïves, 58.3% of pre-treated). Reasons for study discontinuation (multiple responses permitted) were stopping of DTG (7.8%, incl. one virologic failure), patient wish (3.2%), loss to follow-up (2.2%), death (0.2%) and other (2.4%). During the first year, 10.7% of patients experienced ADRs; 4.4% discontinued DTG for this, including 1.2% for depression and 1% for gastrointestinal symptoms.

**Conclusion**: During a median observation time of 15.8 months, monitoring measures were mainly related to routine quarterly controls of HIV disease, consistent with recommendations of national guidelines. Discontinuation rates due to ADRs and virologic failure were 4.4% and 0.2%, respectively. These preliminary NIS data in a real-world cohort replicate the good effectiveness and tolerability of DTG shown in registration studies.

**Abstract P131–Figure 1:** Time to treatment discontinuation (Kaplan-Meier) for all patients initiated on DRV/r QD.

# Sub article: P134 First Canadian HIV+/HIV+ kidney transplantation and first results of the prospective cohort of solid organ transplantation for HIV individuals of Centre Hospitalier de l'Université de Montréal

**Published:** 2016-10-23

**Authors:** Georges Ambaraghassi, Héloïse Cardinal, Daniel Corsilli, Claude Fortin, Marie-Chantal Fortin, Jacques Malaise, Valérie Martel-Laferrière, Michel Pâquet, Danielle Rouleau

**Affiliations:** 1 Microbiologie Médicale et Infectiologie, Centre Hospitalier de l'Université de Montréal, Montréal, Canada; 2 Néphrologie, Centre Hospitalier de l'Université de Montréal, Montréal, Canada; 3 Soins Intensifs, Centre Hospitalier de l'Université de Montréal, Montréal, Canada; 4 Transplantation Chirurgicale, Centre Hospitalier de l'Université de Montréal, Montréal, Canada

## Abstract

**Introduction**: Due to careful selection of potential candidates, patients and grafts survival among HIV-infected patients is similar to non-infected patients with solid organ transplantation (SOT). Management of drug interactions is a challenge in HIV-infected patients. Protease inhibitors (PI) interact with tacrolimus, making dosage adjustments more difficult to handle, thus increasing the risk of rejection. Prospective cohorts of HIV-infected kidney and liver recipients are mostly from Europe and the USA. The aim of this study is to describe a Canadian single-centre experience with SOT of HIV-infected patients, including the first Canadian HIV+/HIV+ kidney transplantation.

**Materials and methods**: This is a prospective cohort study conducted at the Centre Hospitalier de l'Université de Montréal (CHUM). The study consists of a chart review of HIV-infected patients evaluated, listed or who received a SOT. Eligibility criteria include CD4 T-cell count above 200 cells/µL, undetectable HIV viral load, stable ART for at least 3 months and no untreatable opportunistic infections (OIs). Data were collected from clinical charts and include demographic characteristics, medical history including detailed HIV, hepatic and renal disease status and both pre- and post-transplant assessment laboratory. Study endpoints are HIV virologic escape post-transplantation, OIs, rejection and mortality during the solid organ transplant process.

**Results**: A total of 11 HIV-infected patients were recruited and five among them received a kidney transplantation and one a liver transplantation (Table 1). Medial follow-up is 6 months (range 2–49). All patients received basiliximab induction, and immunosuppressive agents consisted of tacrolimus (n=6), prednisone (n=6), mycophenolate mofetil (n=2), azathioprine (n=1) and leflunomide (n=1). Most patients were on an integrase inhibitor (II)-based regimen (n=4) while some were on a PI-based regimen (n=2). HIV viral load remained steadily undetectable post-transplantation in all patients and no HIV-associated OI was reported. One kidney recipient on darunavir/ritonavir/etravirine/raltegravir developed post-transplantation chronic rejection. All kidney recipients remain dialysis-free at this time with a post-transplant mean eGFR of 55.5 mL/min/1.73 m^2^. One patient received a kidney graft from an HIV-infected donor. Both the donor and the recipient were on a similar II-based regimen before, during and after the transplantation.

**Conclusions**: Our preliminary results demonstrate that SOT is a viable option for HIV-infected patients with terminal organ failure. ART free of drug interaction should be promoted when possible to prevent rejection. With careful selection, HIV+/HIV+ kidney transplantation can be performed without loss of virologic control.

**Abstract P134–Table 1:** Clinical characteristics of solid organ recipient and post-transplant follow-up

Characteristics	Patient 1	Patient 2	Patient 3	Patient 4	Patient 5	Patient 6
Pre-transplant assessment						
Age	57	52	40	50	56	45
Gender	M	M	M	F	F	M
Organ	Kidney	Kidney	Kidney	Liver	Kidney	Kidney
Time on dialysis (months)	40	132	83	N/A	70	54
CD4 T-cell count (cells/µl)	500	390	620	300	200	410
Post-transplant follow-up						
ART	ABC, 3TC, DTG	ABC, 3TC, RAL	DRV/r, ETR, RAL	FTC, 3TC, RAL	ABC, 3TC, RPV, DTG	DRV/r, EFV, RAL
Immunosuppressive regimen	TAC, MMF, prednisone	TAC, prednisone	TAC, LEF, prednisone	TAC, AZA, prednisone	TAC, MMF, prednisone	TAC, MMF, prednisone
CD4 T-cell count (cells/µl)	330	410	510	350	270	540
HIV viral load (copies/ml)	<40	<40	<40	<40	<40	<40
eGFR (ml/min/1,73m^2^)	53	28	39	54	98	61
Rejection (n)	0	0	1	0	0	0
Follow-up (months)	6	49	19	5	2	6

# Sub article: P135 Why do HIV/AIDS patients fail? Incidence, causes, demographic, immunologic and clinical characteristics of HIV patients who fail to achieve complete virologic suppression

**Published:** 2016-10-23

**Authors:** Daniel Elbirt, Yanina Inberg, Keren Mahlab-Guri, Ilan Asher, Shira Bezalel-Rosenberg, Michael Burke, Zev Sthoeger

**Affiliations:** 1 Kaplan Medical Center, Neve Or AIDS Center, Rehovot, Israel

## Abstract

**Introduction**: Patients treated with HAART are expected to reach complete viral suppression. Still, in “real life” about 20% of patients do not achieve this goal. The “failing” patients demand a significant part of the HIV clinic efforts and resources. We believe that characterization of these failing patients could help targeting them in a more effective way.

**Methods**: We conducted a retrospective “snap shot” analysis, seeking for failing patients. Data were obtained from charts of HIV patients in a major Israeli HIV/AIDS centre during 2015. We included adults on HIV treatment for at least 1 year. All patients had at least two viral load tests during the year prior to enrolment. Virologically suppressed patients were defined as having two consecutive undetectable viral loads (<20 copies/mL).

**Results**: Seven hundred and sixty-six patients were included. Fifty-four percent were men, mean age 47.06±11.48 years (37%, >50 years old), the mean follow-up was 11.8±6.4 years. Risk groups: 65% of patients were from endemic area (Ethiopia), 13% were men who have sex with men (MSM) and 7% intravenous drug users (IVDU). In our analysis, 85 (11%) of the patients did not achieve complete viral suppression. African patients were more prone to fail compared to other risk groups (77% of the failing patients vs. 6% [MSM] and 12% [IVDU]; p<0.05). Age, sex, follow-up, matrimonial status, working status and AIDS at diagnosis were not associated with failing, while a serodiscordant spouse was associated with a lower rate of virologic failure (7% vs. 14%; p=0.03). The failing patients had more complications (48% vs 32%; p=0.005). Patients treated with protease inhibitor (PI)-based regimen did not have higher failing rates, NNRTI-based regimen predicted a lower chance of virologic failure (31% vs. 7%; p<0.05), and an integrase-based regimen predicted higher rates of failing (46% vs 33%; p<0.05).

**Conclusions**: We found that a target of 90% (89% in our cohort) viral suppression is achieved in “real life.” HIV patients originating from Africa were more prone to fail treatment. We found no correlation between other demographic and socioeconomic factors and the chances to fail therapy. Interestingly, living with a serodiscordant couple was associated with lower rates of virologic failure. We also found a correlation between HAART regimens and virologic failure rates. While PI-based regimen had no influence, NNRTI-based treatment was associated with lower chance to fail and integrase-based treatment was associated with higher rate of failing.

# Sub article: P136 Hepatic safety during treatment with darunavir-based regimens in an Italian observational study

**Published:** 2016-10-23

**Authors:** Andrea Antinori, Andrea Gori, Roberto Cauda, Giancarlo Orofino, Fiorella Di Sora, Paolo Grossi, Maurizio Mineo, Giuseppe Airoldi, Daniela Mancusi, Roberta Termini

**Affiliations:** 1 Clinical Department, National Institute for Infectious Diseases Lazzaro Spallanzani, Rome, Italy; 2 Clinic of Infectious Diseases, San Gerardo Hospital, University of Milano-Bicocca, Monza, Italy; 3 Institute of Infectious Diseases, Catholic University of the Sacred Heart, Rome, Italy; 4 Unit of Infectious Diseases, University of Turin and Department of Medicine, Amedeo di Savoia Hospital, University of Turin, Turin, Italy; 5 Department of Clinical Immunology, Hospital San Giovanni Addolorata, Rome, Italy; 6 Infectious Diseases, Insubria University, Varese, Italy; 7 Infectious Diseases Unit, Policlinico Universitario “P. Giaccone”, Palermo, Italy; 8 Statistics, Studio Associato Airoldi, Milan, Italy; 9 Janssen-Cilag SpA, Medical Affairs, Cologno Monzese, Italy

## Abstract

**Introduction**: TMC114-HIV4042 is a non-interventional study evaluating virologic response and safety of darunavir/ritonavir (DRV/r) administered with other ARV agents in clinical practice. Here, we show the effects on liver function and safety in all HIV1-infected DRV-naïve patients.

**Methods**: Two hundred and thirty-three DRV-naïve patients, 117 ARV naïve and 116 ARV experienced, received a DRV/r-based regimen in routine practice, together with other active ARVs, and were observed for 12–40 months up to end 2012 or earlier discontinuation. Serum biochemistry including aspartate aminotransferase (AST), alanine aminotransferase (ALT), gamma-glutamyl-transferase (GGT) and bilirubin was obtained before entry and at about 3-month intervals. Reported hepato-biliary adverse events (AEs) were examined. Time trends were analyzed using repeated-measures mixed models, with values imputed after study end for hepatic AEs.

**Results**: Fifty-two patients (22.3%) had viral hepatitis (26 HCV, 16 HBV, nine both, one unspecified) active at entry (Table 1).

Background ARV therapy at entry included a fixed tenofovir-emtricitabine combination in 107 (91.5%) ARV-naïve patients and 66 (56.9%) ARV-experienced patients. Hepatic AEs (non-serious except one fatal) were reported in seven patients (3.0%), four ARV naïve and three ARV experienced: 5/52 (9.6%) in patients co-infected (three HCV, two HBV), 1/23 (4.3%) with past HBV/HCV infection not active, 1/158 (0.6%) not co-infected with HBV/HCV. An HCV co-infected ARV-experienced patient died of liver failure assessed as unrelated with ARVs, one HCV co-infected ARV-naïve withdrew for hypertransaminasemia probably DRV-related, and one HBV co-infected ARV-naïve withdrew for increased GGT possibly DRV-related. In four patients, hepatic AEs unrelated with DRV (raised AST and ALT in two patients; raised AST, ALT and GGT in one patient; cholestasis with raised GGT in one patient) withdrew without changing the DRV-based ARV therapy. Mean AST and ALT levels decreased by about 10 U/L until 6–8 months remaining constant thereafter in ARV-naïve patients and were stable throughout in ARV-experienced patients (Figure 1). Mean GGT levels in both groups decreased until 6–10 months and changed slightly thereafter. Total bilirubin levels quickly reverted to normal in 14 patients with baseline hyperbilirubinemia who switched from atazanavir at entry; mean values in the other patients, although slightly increasing, were always <0.6 mg/dL (Figure 2).

**Conclusions**: In HIV-infected patients given a DRV/r-based regimen for a mean duration >20 months, liver AEs were few and mostly related to the underlying viral hepatitis, and mean serum liver enzymes and total bilirubin levels did not worsen.

**Table 1:** Demographic characteristics, CDC clinical stage and concomitant liver diseases at entry

	ARV-naïve (N = 117)	ARV-experienced (N = 116)
Age, years		
mean±SD	42.0±11.0	44.3±9.5
Gender at birth, N (%)		
Male	99 (84.6)	91 (78.4)
Race, N (%)		
Black	5 (4.3)	3 (2.6)
Caucasian/other	112 (95.7)	113 (97.4)
CDC clinical stage C, N (%)	38 (32.5)	41 (35.3)
History of HBV-HCV hepatitis, N (%)		
No	88 (75.2)	70 (60.3)
Not active (b)	9 (7.7)	14 (12.1)
HBV	6	5
HCV	2	5
HBV/HCV	1	3
Unspecified	0	1
Active (a)	20 (17.1)	32 (27.6)
HBV	12	4
HCV	6	20
HBV/HCV	2	7
Unspecified	0	1

a HCV RNA quantitation within 6 months before entry, or positive HBV and HCV serology within 6 months before entry, or reported as an active concomitant disease at entry.

b HCV RNA quantitation more than 6 months before entry, or positive HBV and HCV serology more than 6 months before entry, or reported as a previous (not active) disease at entry.

CDC, Center for Disease Control and Prevention; HBV, hepatitis B virus; HCV, hepatitis C virus; SD: standard deviation.

**Abstract P136–Figure 1:** Time trend of serum ALT levels (geometric mean) during the study.

**Abstract P136–Figure 2:** Time trend of total bilirubinemia (geometric mean) during the study.

# Sub article: P137 Long-term use of darunavir/r QD monotherapy in daily practice: retrospective observational cohort data of 111 HIV patients in Belgium

**Published:** 2016-10-23

**Authors:** Stéphane De Wit, Eric Florence, Linos Vandekerckhove, Bernard Vandercam, Jean-Christophe Goffard, Eric Van Wijngaerden, Michel Moutschen, Remy Demeester, Hilde Piryns, Pushpike Thilakarathne

**Affiliations:** 1 Infectious Diseases, Saint-Pierre University Hospital, Brussels, Belgium; 2 Infectious Diseases, Institute of Tropical Medicine, Antwerp, Belgium; 3 Infectious Diseases, Ghent University Hospital, Ghent, Belgium; 4 Infectious Diseases, Saint-Luc University Hospital, Brussels, Belgium; 5 Infectious Diseases, University Hospital Erasme, Brussels, Belgium; 6 Infectious Diseases, University Hospital Leuven, Leuven, Belgium; 7 Infectious Diseases, Liège University Hospital, Liège, Belgium; 8 Infectious Diseases, University Hospital Charleroi, Charleroi, Belgium; 9 Janssen-Cilag NV, Medical Affairs, Beerse, Belgium; 10 Department of Statistics, Janssen, Beerse, Belgium

## Abstract

**Objectives**: To describe the use of darunavir/ritonavir monotherapy (DRV/r) in daily practice in Belgium.

**Methods**: *Design*: Observational, non-interventional, non-comparative, retrospective, multicentre cohort study. Data were collected from existing databases from eight AIDS reference centres in Belgium. A subgroup on darunavir/r QD monotherapy was analyzed. *Inclusion*: HIV-1 infected adults, on DRV/r 800 mg/100 mg QD monotherapy (received at least one dose of DRV/r from January 1, 2010 on, with a minimum follow-up of 6 months). Primary endpoints: Time to treatment discontinuation (using Kaplan-Meier estimates), rate and reason for discontinuation of DRV/r QD monotherapy.

**Results**: Hundred and eleven patients (9.8%), all treatment experienced, received DRV/r QD monotherapy, out of a group of 1701 patients exposed to DRV/r. Baseline characteristics of the monotherapy subgroup: 65.8% were male, mean age of 49.4 years (±11.0), 65.8% Caucasian, mean CD4 count 648.1 cells/ mm^3^ (±347.3) and 18% had CD4 counts <350 cells/mm^3^. Mean CD4 nadir was 223.0 cells/mm^3^ (±157.5); 21.6% had a CD4 nadir <100 cells/mm^3^, and in 51.4% of patients the CD_4_ nadir was ≥200 cells/mm^3^. 84.7% of patients had HIV RNA <50 copies/mL at baseline. Median follow-up period (Q1–Q3) was 2.55 (1.27–3.34) years. The probability to remain on treatment (95% CI) was 81.3% (72.5–87.5%) for the first year, also 81.3% (72.5–87.5%) for the second year and 74.2% (63.1–82.4%) for the third year (Figure 1).

The probability to remain on monotherapy did not differ significantly from the overall cohort on DRV/r QD containing combination regimen (log rank test p-values: 0.4496). Twenty-eight of 111 patients (25.2%) discontinued treatment with DRV/r QD monotherapy. Main reasons were adverse events such as GI toxicity (4.5%), liver toxicity (0.9%), other toxicities (1.8%), hypersensitivity reaction (1.8%), patient's decision (5.4%) or death (5.4%). Non-compliance was mentioned in 1.8% and lack of efficacy in 1.8%. At the end of follow-up period, 77% of patients had HIV RNA <50 copies/mL.

**Conclusions**: In this real-life, long-term, retrospective Belgian cohort, around 9.8% of treatment-experienced patients on DRV/r QD were on monotherapy. The results confirm the efficacy and good tolerability of DRV/r QD monotherapy. The rates of discontinuation in the DRV/r QD monotherapy group are not very different from those in the overall cohort containing DRV/r QD in combination regimen. DRV/r monotherapy is not registered and is only recommended to a selected group of patients according to the European guidelines.

**Time to treatment discontinuation (Kaplan-Meier) for patients on DRV/r QD monotherapy**

# Sub article: P138 Development of a contemporary symptom diary for patients with HIV

**Published:** 2016-10-23

**Authors:** Jessica Markowitz, Naomi Knoble, Benoit Arnould, Albert Wu

**Affiliations:** 1 Patient-Centered Outcomes, Mapi, Boston, MA, USA; 2 Patient-Centered Outcomes, Mapi, Lyon, France; 3 Health Policy and Management, Johns Hopkins School of Public Health, Baltimore, MD, USA

## Abstract

**Introduction**: The widespread adoption of modern ART has changed the landscape of HIV treatment. The most widely used patient reported outcomes (PRO) questionnaires were developed over a decade ago and may not adequately capture the experience of HIV patients today. In addition, best practices in patient symptom measurement have changed substantially, and there is growing interest in using daily symptom diaries, which may capture the patient experience more accurately. The goal of this project was to identify the HIV symptoms experienced by patients as a result of both their disease and treatment and, using that information, develop a web-based symptom diary for patients with HIV to capture the presence and impact of symptoms on a daily basis.

**Materials and methods**: A narrative review of the literature was conducted to identify PRO symptom measures in HIV and evaluate their validity in the current environment of HIV treatment. A web-based survey regarding important HIV symptoms was completed by 20 US clinicians specializing in HIV/infectious disease. Results from the literature review and clinician survey were used as the basis for diary development. Patients were recruited from four geographically diverse treatment centres in the US (CA, NM, DC, MA). Concept elicitation interviews and cognitive debriefing on the initial diary were completed with 26 patients who guided diary refinement. Next, 48 patients (inclusive of the 26) used the web-based diary daily for 1 week and completed a cognitive debriefing interview to finalize the content/format of the diary. The diary asks patients to report on symptoms (using checklists/pictures) and symptom impact.

**Results**: Participants (77% male) were White (62%) or Black/African American (34%) and Hispanic (15%). Mean participant age was 52 (range 27–69). Educational level of at least some college was 69%; 63% were employed. HIV transmission mode was primarily MSM (70%); patients reported a variety of HIV treatment regimens. The diary took 5–10 minutes to complete each day, and the majority of the feedback on the diary was positive. Patients accessed the diary by computer (42%), smartphone (33%), tablet (10%) and combination (14%). The diary enabled comprehensive and organized capture of symptoms that patients viewed as relevant, with impact measured at the individual symptom level.

**Conclusions**: This daily HIV symptom diary is brief, easy to complete and well received by patients. It provides patients of diverse background, education and treatments, and the opportunity to voice their symptom experience. Validity studies are ongoing.

# Sub article: P139 A moderated mediation model of HIV-related stigma, depression and social support on health-related quality of life among incarcerated Malaysian men with HIV and opioid dependence

**Published:** 2016-10-23

**Authors:** Roman Shrestha, Michael Copenhaver, Frederick Altice

**Affiliations:** 1 Community Medicine and Health Care, University of Connecticut Health Center, Farmington, CT, USA; 2 Allied Health Sciences, University of Connecticut, Storrs, CT, USA; 3 Internal Medicine, Yale University, New Haven, CT, USA

## Abstract

**Introduction**: Although it is well established that HIV-related stigma, depression and lack of social support are negatively associated with health-related quality of life (HRQoL) among people living with HIV (PLHIV) [1–3], no studies to date have examined how these psychosocial factors interact with each other and affect HRQoL among incarcerated PLHIV. This paper incorporated a moderated mediation model to explore whether depression mediates the effect of HIV-related stigma on HRQoL as a function of the underlying level of social support.

**Methods**: Incarcerated HIV-infected men with opioid dependence (N=301) were recruited from Malaysia's largest prison, located in Greater Kuala Lumpur. Participants were assessed using the Berger HIV Stigma Scale (40–160), the Center for Epidemiological Studies Depression (CES-D) Scale (0–60), the Medical Outcomes Study Social Support Survey (0–100) and the RAND 36-Item Health Survey (0–100). An ordinary least squares regression-based path analytic framework was used to test the moderated mediation model.

**Results**: Results showed that the effect of HIV-related stigma on HRQoL was mediated via depression (a1: β=0.1463, p<0.001; b: β=−0.8392, p<0.001), as demonstrated by the two-tailed significance test (Sobel z=−3.8762, p<0.001). Furthermore, the association between social support and HRQoL was positive (β=0.4352, p=0.0433), whereas the interaction between HIV-related stigma and depression was negatively associated with HRQoL (β=−0.0317, p=0.0133). This indicated that the predicted influence of HIV-related stigma on HRQoL via depression had negative effect on HRQoL for individuals with low social support.

**Conclusions**: Findings provide evidence of the moderated effect of social support on the translation of HIV-related stigma into HRQoL via depression. The results suggest that social support can buffer the negative impact of depression on HRQoL and highlights the need for future interventions to target these psychosocial factors in order to improve HRQoL among incarcerated PLHIV.

## References

[1] Charles B, Jeyaseelan L, Pandian AK, Sam AE, Thenmozhi M, Jayaseelan V (2012). Association between stigma, depression and quality of life of people living with HIV/AIDS (PLHA) in South India – a community based cross sectional study. BMC Public Health.

[2] Li X, Li L, Wang H, Fennie KP, Chen J, Williams AB (2015). Mediation analysis of health-related quality of life among people living with HIV infection in China. Nurs Health Sci.

[3] Tate D, Paul RH, Flanigan TP, Tashima K, Nash J, Adair C (2003). The impact of apathy and depression on quality of life in patients infected with HIV. AIDS Patient Care STDS.

# Sub article: P140 The DRIVER study: asymptomatic STI systematic screening versus targeted screening according to STI risk factors in a cohort of outpatients HIV-infected MSM seen in France – phase 1 results

**Published:** 2016-10-23

**Authors:** Sebastien Fouere, Svetlane Dimi, Julie Timsit, Christophe Lalanne, Olivier Chassany, Martin Duracinsky, Eric Farfour, Catherine Majerholc, David Zucman

**Affiliations:** 1 Centre des MST Service de Dermatologie, Hôpital Saint-Louis, Paris, France; 2 Service Médecine Interne, Hôpital Foch, Suresnes, France; 3 EA 7334 REMES, Patient-Centered Outcomes Research, University Paris Diderot Sorbonne, Paris, France; 4 Service de Biologie Médicale, Hôpital Foch, Suresnes, France

## Abstract

**Introduction**: HIV-infected men who have sex with men (MSM) are frequently affected by symptomatic sexually transmitted infections (STI). Most of those who are stabilized on treatment benefit from semestrial follow-up visits which may provide an opportunity to screen them for asymptomatic STI (ASTI). The DRIVER study seeks to determine whether this should be done on a systematic basis or oriented by risk factors (RF). Phase 1 explored the ASTI prevalence and evaluated the relevance of a series of RF for STI which could be used eventually to build a decisional score.

**Material and methods**: Patients were prospectively included at 13 hospital-based HIV clinics of the Paris (France) region. During any one of their semestrial follow-up visits, clinical data were recorded, a self-administered sociodemographic and behavioural questionnaire was filled in and patients were screened for syphilis antibodies and PCR for *Chlamydia trachomatis* (CT) and *Neisseria gonorrhoeae* (NG) (pharynx, anus and first-void urine). Statistical correlation between recorded items and the presence of STI was measured by the chi-square law.

**Results**: Four hundred and eighty-fiveasymptomatic MSM HIV-positive patients (median age 47 years, average time living with HIV 13 years, undetectable viral load in 94%, average CD4+ 679/mm^3^) were included. More than 80% had at least graduated from high school, and 72% had a stable professional situation. Nearly 63% had a history of STI (genital warts in 39%, syphilis in 45%), within the previous 12 months in 18%. An ongoing ASTI was found in 13.6% (syphilis 5.6%, CT or NG infection 8%). RF significantly associated with recent syphilis were not being engaged in a stable relationship (p=0.013), to have receptive anal sex (p=0.03) and to have a history of previous syphilis (p=0.005). Those associated with CT or NG infection were having a stable professional occupation (p<0.001) and to use illicit drugs during sex (p=0.05).

**Discussion and conclusion**: The DRIVER study shown the high frequency of past and recent STI history and confirm the relevance of our study. Out of a long and thorough list of possible RF, only a few were found to be positively correlated with ASTI, some of them counter intuitive, i.e. stable occupation and CT/NG infection. These results were comparable from the scientific literature, and they confirm the frequency of ASTI in the HIV-positive MSM population. The predictive value of an STI-screening RF-based decisional score will be tested during phase 2 of the DRIVER study.

# Sub article: P141 Metabolic safety during treatment with darunavir-based regimens in an Italian observational study

**Published:** 2016-10-23

**Authors:** Andrea Antinori, Giovanni Di Perri, Paola Meraviglia, Goffredo Angioni, Antonio Chirianni, Gaetana Sterrantino, Tiziana Quirino, Giuseppe Airoldi, Daniela Mancusi, Roberta Termini

**Affiliations:** 1 Clinical Department, National Institute for Infectious Diseases Lazzaro Spallanzani, Rome, Italy; 2 Unit of Infectious Diseases, Department of Medical Sciences Amedeo di Savoia Hospital, University of Turin, Turin, Italy; 3 Department of Infectious Disease, L. Sacco University Hospital, Milan, Italy; 4 Department of Infectious Diseases, P. O. Santissima Trinità, Cagliari, Italy; 5 Infectious Diseases, D. Cotugno Hospital, Naples, Italy; 6 Infectious Diseases, Careggi University Hospital, Florence, Italy; 7 Infectious Diseases, Busto Arsizio Hospital, Busto Arsizio, Italy; 8 Statistics, Studio Associato Airoldi, Cicogna, Ghirri, Milan, Italy; 9 Janssen-Cilag SpA, Medical Affairs, Cologno Monzese, Italy

## Abstract

**Introduction**: TMC114-HIV4042 is a non-interventional study evaluating the virologic response and safety of darunavir/ritonavir (DRV/r) administered with other ARV agents in clinical practice. Here, we show the effects on lipid and glucose metabolism in all HIV1-infected DRV-naïve patients.

**Methods**: Two hundred and thirty-three DRV-naïve patients, 117 ARV naïve and 116 ARV experienced, received a DRV/r-based regimen in routine practice, together with other active ARVs, and were observed for 12–40 months up to end 2012 or earlier discontinuation. Serum biochemistry including lipids and glucose was obtained before entry and at about 3-month intervals, or longer when allowed by stable patient conditions. Reported adverse events (AEs) related to lipid and glucose metabolism were examined.

**Results**: Patients were mostly men (99 ARV naïve and 91 ARV experienced); 38 and 41, respectively, were in Centers for Disease Control and Prevention clinical stage C at entry; mean age was 42 and 44 years, respectively. At baseline, eight patients (seven ARV experienced) were reportedly hyperlipidaemic and five (four ARV experienced) diabetic. Background ARV therapy at entry included a fixed tenofovir-emtricitabine combination in 107 (91.5%) ARV-naïve patients and 66 (56.9%) ARV-experienced patients. During the study, hyperlipidaemia was reported as AE in 14 patients (6.0%), eight ARV naïve and six ARV experienced, none serious; nine (3.9%) were assessed by the clinician as at least possibly related to DRV; one caused study discontinuation. Two patients had diabetes reactivation (one with weight increase and hyperlipaemia), one lipodystrophy causing withdrawal and one weight decrease requiring hospitalization (Table 1). Over the first 4 months, levels of triglycerides, total, low-density lipoprotein and high-density lipoprotein cholesterol increased in ARV-naïve patients then they were approximately constant; median baseline and final values were 117–147, 147–183, 91–118 and 35–40 mg/dL, respectively. In ARV-experienced patients lipid parameters remained stable; median baseline and final values were 137–134, 176–193, 114–121 and 42–42 mg/dL, respectively. Median serum-glucose levels remained stable in both groups.

**Conclusions**: In HIV-infected patients given a DRV/r-based regimen for a mean duration of >20 months, serum-glucose levels did not change. In ARV-naïve patients, lipid parameters increased during the first months of the study although remaining in the normal range except for triglycerides (+20 mg/dL). Study discontinuations due to lipid dysmetabolism were rare.

**Abstract P141–Table 1:** Metabolic AEs reported during the study, N (%) of patients

	ARV naïve (N=117)	ARV naïve (N=117)	ARV experienced (N=116)	ARV experienced (N = 116)
	
Type of metabolic AE – WHO-ART preferred term	AEs	ADRs	AEs	ADRs
Hyperlipidaemias	8 (6.8%)	5 (4.3%)	6 (5.2%)	4 (3.4%)
- Hypercholesterolemia	2	2	1	1
- Hypercholesterolemia	0	0	1 (e)	1
- Hypertriglyceridemia	1	0	3	2
- Hyperlipaemia (l)	4	2	1	0
- Hyperlipaemia (l)	1 (a)	1	0	0
Other metabolic AEs				
- Diabetes mellitus reactivated	1 (a)	0	1	0
- Weight increase	1 (a)	0	0	0
- Weight decrease	1 (s)	0	0	0
- Lipodystrophy	1 (e)	1	0	0

(l) hypercholesterolemia + hypertriglyceridemia; (a) AEs occurring in the same patient; (s) serious AE (caused hospitalization); (e) study discontinued because of AE. ADR, adverse drug reaction (AE possibly or probably related to DRV according to the clinician); WHO-ART, World Health Organization adverse reactions terminology.

# Sub article: P142 Low rifampicin and isoniazid concentrations are associated with delayed sputum conversion in HIV-positive patients co-infected with tuberculosis in Uganda

**Published:** 2016-10-23

**Authors:** Christine Sekaggya, Bruno Ledergerber, Amrei von Braun, Mohammed Lamorde, Allan Buzibye, Nadia Eberhard, Alexandra Scherrer, Ritah Nakijoba, Daniel Müller, Lars Henning, Ursula Gutteck, Natascia Corti, Joseph Musaazi, Moses Kamya, Barbara Castelnuovo, Andrew Kambugu, Jan Fehr

**Affiliations:** 1 Research, Infectious Diseases Institute, College of Health Sciences, Makerere University, Kampala, Uganda; 2 Infectious Diseases and Hospital Epidemiology, University Hospital of Zurich, University of Zurich, Zurich, Switzerland; 3 Department of Clinical Chemistry, University Hospital of Zurich, University of Zurich, Zurich, Switzerland; 4 Department of Clinical Pharmacology and Toxicology, University Hospital of Zurich, University of Zurich, Zurich, Switzerland; 5 Department of Medicine, College of Health Sciences, Makerere University, Kampala, Uganda

## Abstract

**Introduction**: HIV-positive patients co-infected with tuberculosis (TB) have anti-TB drug concentrations lower than reference ranges; however, the relationship between concentrations of anti-TB drugs and treatment response remains controversial. We sought to evaluate if there is an association between low concentrations of first-line anti-TB drugs and delayed sputum conversion in a cohort of HIV/TB co-infected Ugandan adults.

**Materials and methods**: We enrolled HIV-infected Ugandan adults diagnosed with a first episode of pulmonary TB. Patients underwent pharmacokinetic sampling 1, 2 and 4 hours after drug intake to estimate the maximum drug concentrations (eC_max_) at 2, 8 and 24 weeks of TB treatment using high-performance liquid chromatography. Low concentrations were defined as an eC_max_ below the previously described cut-offs for rifampicin <8 mg/L and isoniazid <3 mg/L. Sputum conversion was defined as conversion of sputum culture or smear from positive to persistently negative results during follow-up. Cox regression and Kaplan-Meier curves were used to determine the association between sputum conversion dynamics and anti-TB drug concentrations.

**Results**: From April 2013 to May 2015, we included 226 HIV-infected patients with positive sputum cultures or smears at baseline. The median age was 34 years (interquartile range [IQR] 29–40), 58% (133) were male, the median CD4 cell count was 191 cells/mm^3^ (IQR 70–333), and the median BMI was 19.1 kg/m^2^ (IQR 17.6–21.6). The majority (177, 78%) of all patients was ART naïve at time of TB diagnosis. Patients with low isoniazid and rifampicin concentrations were less likely to undergo sputum conversion before the end of follow-up compared to those with normal concentrations (HR 0.51; 95% CI 0.35–0.72; p<0.001 and HR 0.61; 95% CI 0.44–0.84; p=0.003 respectively). In addition, patients with ≥1 drugs below the cut-off had a higher probability of remaining culture/smear positive over time compared to those with no drug below the cut-off (Figure 1). These associations remained unchanged in models adjusted for age, sex and BMI.

**Conclusions**: Low isoniazid or rifampicin concentrations in HIV/TB co-infected patients resulted in delayed sputum conversion. This has potential implications on TB transmission.

**Abstract P137–Figure 1:** Time to treatment discontinuation (Kaplan-Meier) for patients on DRV/r QD monotherapy.

# Sub article: P143 Isoniazid preventive therapy is highly cost-effective among TB/HIV co-infected patients in Uganda

**Published:** 2016-10-23

**Authors:** Christine Sekaggya Wiltshire, Andreas Kuznik, Mohammed Lamorde

**Affiliations:** 1 Research, Infectious Diseases Institute, Kampala, Uganda; 2 Pharmaceuticals, Regeneron Pharmaceuticals, New York, NY, USA

## Abstract

**Introduction**: Isoniazid preventive therapy (IPT) for at least 6 months is recommended by the WHO for the treatment of latent tuberculosis (TB) in patients infected with HIV. This study aimed to determine the cost-effectiveness of IPT versus no treatment for latent TB among HIV-infected patients in an urban outpatient clinic in Kampala, Uganda.

**Methods**: The analysis was conducted from the perspective of the national health system. Using decision analysis, we modelled the impact of IPT versus no IPT on costs and patient outcomes, using a probability of developing TB of 2.5% in the IPT arm and 7.5% in the no-IPT arm, based on published sources [1]. We estimated the median daily price of isoniazid at $0.048 over the course of 6 months, based on international drug price lists. We also included the cost of first- and second-line TB treatment at $12.90 and $110.7, respectively [2]. The TB associated mortality rate (10.5%) and failure/relapse rate (12.4%) associated with TB treatment was obtained from a systematic review of first-line treatment of TB in HIV-infected patients [3]. We used a life expectancy of 35.1 years estimate from a study on patients on combined antiretroviral therapy in Uganda [4]. Study results were expressed in cost per disability-adjusted life years (DALY) averted and compared against WHO cost-effectiveness thresholds.

**Results**: The full course of IPT is associated with a cost of $8.64, but approximately $1.33 of which are offset due to reduced need for first- and second-line TB therapy, yielding a net cost of $7.31. IPT is also associated with a reduction in DALYs by 0.118, yielding a cost/DALY averted of $62, which is well below Ugandan per capita GDP.

**Conclusion**: Use of IPT is highly cost-effective in TB/HIV co-infected patients. National programmes should consider IPT a priority in Uganda and health providers should be encouraged to increase compliance with WHO guidelines on treatment of latent TB.

## References

[1] Akolo C, Adetifa I, Shepperd S, Volmink J Treatment of latent tuberculosis infection in HIV infected persons. Cochrane Database Syst Rev..

[3] http://dx.doi.org/10.1371/journal.pone.0039187.

[4] http://dx.doi.org/10.7326/0003-4819-155-4-201108160-00358.

# Sub article: P144 Predicting the in-hospital mortality in tuberculous meningitis

**Published:** 2016-10-23

**Authors:** Raluca Jipa, Eliza Manea, Ioana Olaru, Simona Merisor, Cristian Niculae, Adriana Hristea

**Affiliations:** 1 Clinical – Adults IV, National Institute of Infectious Diseases “Prof. Dr. Matei Bals”, Bucharest, Romania; 2 Division of Clinical Infectious Diseases, Research Center Borstel, Borstel, Germany

## Abstract

**Introduction**: Mortality in tuberculous meningitis (TBM) varies from around 20% in HIV-negative patients to more than 50% in HIV-positive patients. A prediction score for unfavourable outcome including altered consciousness, neurologic deficit, hydrocephalus, vasculitis, immunosuppression and diabetes mellitus has been recently published. The aim of our study was to assess if this score is better associated with mortality than neurologic staging in HIV-infected versus HIV-non-infected patients.

**Materials and methods**: We retrospectively analyzed patients admitted to a tertiary care facility between 2005 and 2015 with TBM. Patients were diagnosed as definite, probable and possible TBM according to a consensus definition [1]. Neurologic stages were classified according to the Medical Research Council (MRC) definitions [2]. Hamsi scoring [3] was calculated for all patients for further distribution of mortality.

**Results**: We identified 115 patients of which 55 (48%) had definite, 33 (29%) probable and 27 (23%) possible TBM. Thirty-two (28%) patients were in MRC stage 1, 58 (50%) were in stage 2 and 25 (22%) in stage 3. Fifty-two (45%) patients were immunosuppressed, of which 41 (36%) patients were HIV infected. Fifteen (37%) HIV-infected patients versus six (8%) non-HIV patients died during hospitalization (p<0.001, OR 6.5, 95% CI 2.2–18.6). In the non-HIV patients who were immunosuppressed the mortality was 25%. The median CD4 cell count in HIV-infected patients who died versus those who survived was 67 (IQR 19–145) versus 86 (IQR 45–192), respectively. Mortality rates were 9.4% in patients diagnosed in MRC stage 1, 10.3% for patients in stage 2 and 48% for those in stage 3. Mortality rates in HIV-infected patients were one (2%) for MRC stage 1, four (10%) for stage 2 and 10 (24%) for stage 3 (p=0.007). In non-HIV patients mortality was 3% in all three stages. The distribution of mortality for the Hamsi scores 1 to 6 was 0%, 5.3%, 14.8%, 25.9%, 33% and 40%, respectively. The median Hamsi score was 4, both in patients who survived (IQR 2–5) and in those who died (IQR 4–5) in the HIV-positive group. In the HIV-negative group, the median Hamsi score was 3 (IQR 2–4) in patients who died versus 3 (IQR 3–5) in those who survived. Area under ROC curve for Hamsi score versus clinical staging was 0.775 versus 0.721, respectively (Figure 1).

**Conclusions**: Immunosuppression, particularly HIV infection, is associated with higher mortality in TBM. Higher Hamsi score was associated with higher mortality. Hamsi score was similar with clinical staging in predicting in-hospital mortality. In advanced HIV disease the mortality was not associated with CD4 cell count.

**Abstract P142–Figure 1:** Kaplan-Meier showing probability of remaining smear or culture positive over time among those with 0/1/2 drugs below the cut-off.

## References

[1] Marais S, Thwaites G, Schoeman JF, Török ME, Misra UK, Prasad K (2010). Tuberculous meningitis: a uniform case definition for use in clinical research. Lancet Infect Dis.

[2] Medical Research Council (1948). Streptomycin treatment of tuberculous meningitis. Lancet.

[3] Erdem H, Ozturk-Engin D, Tireli H, Kilicoglu G, Defres S, Gulsun S (2015). Hamsi scoring in the prediction of unfavorable outcomes from tuberculous meningitis: results of Haydarpasa-II study. J Neurol.

# Sub article: P145 Tuberculosis infection in HIV patients in a Portuguese population

**Published:** 2016-10-23

**Authors:** António Maio, Daniel Coutinho, Sofia Nunes, Jorge Velez, Filomena Freitas, Célia Oliveira

**Affiliations:** 1 Infectious Diseases Department, Centro Hospitalar do Baixo Vouga, Aveiro, Portugal

## Abstract

**Introduction**: Tuberculosis (TB) is the most common opportunistic infection affecting HIV patients and remains the most common cause of death in patients with AIDS. HIV increases the risk of disease from TB and leads to more frequent extrapulmonary involvement, atypical manifestations and paucibacillary disease, which can delay diagnosis.

**Material and methods**: Retrospective analysis of files pertaining inpatients with TB and concurrent HIV infection admitted between January 2005 and December 2014. Data were analyzed using χ2 or Fisher's exact test (p<0.005=statistically significant) and odds ratio was calculated.

**Results**: From 222 patients diagnosed with TB during the study, 48 had concurrent HIV infection. Of these 15 had a pulmonary form of TB (PTB) and 36 an extrapulmonary form (ETB) (p=0.052*)*. There was an increased risk of ETB in HIV patients (OR 5.156). In both groups men were the most common gender (PTB 80.0%; ETB 80.6%) with a median age of 39.5 years (PTB 34 years (31.5–43); ETB 43 years (32–50)). CD4 count and viral load was obtained in 40 patients, with a median CD4 count of 74.50 cells/µL (13–136) and viral load of 211,006 copies/mL (1430–4,430,000). In HIV patients, most TB diagnosis were done in the context of new HIV diagnosis (n=34, 70.8%). The others were diagnosed in non-adherence patients. The most common forms of TB were pulmonary (n=15), disseminated (n=11), ganglionar (n=10), meningeal and pleural (n=8). The HIV infection was associated with the presence of disseminated (p=0.000, OR 12.64), pleural (p=0.000, OR 8.5), meningeal (p=0.000, OR 2.96) and ganglionar forms of TB (p=0.000, OR 2.38). Its absence is associated with pulmonary (p=0.000, OR 4.08) and osteoarticular forms (p=0.000, OR 5.4231). TB case was confirmed by culture or PCR test plus smear in 17 cases, was probable (PCR or smear or histology) in 16 cases and possible (only clinical) in 15 cases. Six patients died because of TB infection (12.5%, PTB n=2 vs. ETB n=5) with no prevalence of any particular form of TB.

**Conclusion**: Risk of ETB in HIV patients seems to be higher than PTB. We found an association of HIV infection with disseminated, pleural, meningeal and ganglionar forms. Definitive diagnosis is very difficult in these patients because of paucibacillary disease, requiring sometimes the use of clinical criteria and empiric therapy. Diagnosis in time and appropriate treatments can change the prognosis of these patients.

# Sub article: P147 Use of dolutegravir in combination with rifampicin-based TB therapy in HIV/TB co-infected patients: real-world experience from Leeds, UK

**Published:** 2016-10-23

**Authors:** Muge Cevik, Rachel Vincent, Hugh McGann

**Affiliations:** 1 Infectious Diseases, Western General Hospital, Edinburgh, UK; 2 Infectious Diseases, Leeds Teaching Hospitals NHS Trust, Leeds, UK

## Abstract

**Introduction**: Co-administration of TB and HIV treatment is now the standard of care. Rifampicin-based therapy is the first-line TB treatment; however, there are significant drug interactions as rifampicin is a potent inducer of cytochrome P450 and UGT. Dolutegravir is substrate of UGT1A1 and CYP34 both are induced by rifampicin therefore co-administration of rifampicin decreases dolutegravir plasma concentrations. It is recommended to use dolutegravir 50 mg twice daily when given together with rifampicin. Our HIV MDT has approved the use of dolutegravir in TB/HIV co-infected patients who had adverse reactions with efavirenz or where efavirenz was contraindicated. We aim to present real-world experience of our HIV/TB co-infected patients who were on rifampicin-based TB therapy in combination with dolutegravir-based regimen.

**Methods**: All HIV/TB co-infected patients who were on dolutegravir-based ART and were receiving rifampicin-based TB therapy were identified. Data were retrospectively collated through electronic patient records and case note review. Descriptive statistics were performed to examine demographics, baseline characteristics, CD4 count and HIV viral load. In this cohort, dolutegravir was used 50 mg BID in combination with rifampicin 600 mg OD in all patients.

**Results**: We identified seven patients (one male) who were on dolutegravir-based regimen in combination with rifampicin. Median age was 41 years (27–48) and all were black African in origin. Five patients were naïve to ART whereas two were ART experienced. There was no baseline integrase resistance mutations identified. At baseline median CD4 count was 90 cells/mL (3–365), five patients had CD4 <100 cells/mL and only one patient had undetectable viral load. At 6 months after HIV treatment median CD4 count improved to 230 cells/mL (104–625) and all except one patient had undetectable HIV viral load. This patient was not suppressed due to well-documented poor adherence and repeat resistance test revealed new M184V mutation with no integrase resistance. In our cohort all patients tolerated the treatment well without significant adverse events. Four patients successfully completed TB therapy and three are on ongoing treatment. Two patients have subsequently switched from dolutegravir due to MSK symptoms after TB treatment was completed.

**Conclusion**: In this small cohort of HIV/TB co-infected patients co-administration of twice-daily dolutegravir in combination with rifampicin was well tolerated with good virological outcome both in naïve and treatment-experienced patients.

# Sub article: P148 The practice and value of interferon gamma release assay testing for latent tuberculosis infection in people living with HIV: a retrospective review of patients at Leeds Teaching Hospitals Trust

**Published:** 2016-10-23

**Authors:** Amy Baggott, Muge Cevik, Hugh McGann

**Affiliations:** 1 Infectious Diseases and Travel Medicine, St James's University Hospital, Leeds, UK; 2 Infectious Diseases, Western General Hospital, Edinburgh, UK

## Abstract

**Introduction**: Both NICE and BHIVA guidelines recommend interferon gamma release assay (IGRA) testing for diagnosing latent tuberculosis (TB) infection in people living with HIV with exposure risk for TB [1,2]. However, “indeterminate” IGRA results are more common in HIV-infected subjects and the possibility of false negative results in those patients with very advanced immunocompromise is a concern [3,4].

**Methods**: As such, a retrospective review was conducted of all patients diagnosed with both TB and HIV in the Leeds Teaching Hospitals Trust during the past 5 years. Records were checked to see if those patients with risk factors for TB exposure had been correctly screened for latent TB with an IGRA test at the time of HIV diagnosis and, if so, how the result had influenced treatment. Demographic data were collected about the patients and their laboratory results, treatment histories and outcomes were analyzed.

**Results**: Of 31 patients, only two had ever had IGRA testing. One had been tested 7 years after being diagnosed with HIV and just 2 weeks before being diagnosed with active TB, with a positive result. The other was tested within 2 weeks of being diagnosed with very advanced HIV and had a false negative result. He was started on treatment for active TB 2 months later and died during treatment. Nine patients were diagnosed with HIV at the same time as TB, but 22 patients were diagnosed with HIV more than a month before being diagnosed with HIV, with an average interval of 4.7 years between diagnoses. Twenty of these 22 patients had exposure risk for TB and should have been screened for latent TB. Two of the patients had MDRTB which would not have been effectively prevented by chemoprophylaxis, even if they had been screened for latent infection. Two deaths occurred, but neither would have been prevented by IGRA testing. Over half of the cohort may have potentially benefited from IGRA testing to screen for latent TB as part of their routine HIV care. However, as the one case who did have an IGRA test at the time of HIV diagnosis demonstrates, IGRA testing can be unreliable in those who present with advanced HIV.

**Conclusion**: From these data, there are missed opportunities to diagnose and treat latent TB, but it is difficult to know how useful IGRA testing would truly have been, if the results had been indeterminate or falsely negative in those who presented with very low CD4 counts.

## References

[1] National Institute for Health and Care Excellence (NICE). NICE guideline (NG33): tuberculosis.

[2] Pozniak AL, Coyne KM, Miller RF, Lipman MCI, Freedman AR, Ormerod LP BHIVA guidelines for the treatment of TB/HIV coinfection 2011.

[3] Mandalakas AM, Hesseling AC, Chegou NN, Kirchner HL, Zhu X, Marais BJ (2008). High level of discordant IGRA results in HIV-infected adults and children. Int J Tuberc Lung Dis.

[4] Sester M, van Leth F, Bruchfeld J, Bumbacea D, Cirillo DM, Dilektasli AG (2014). Risk assessment of tuberculosis in immunocompromised patients. A TBNET study. Am J Respir Crit Care Med.

# Sub article: P150 Incidence and survival in HIV-infected patients with central nervous system opportunistic infections in the cART era: a 10-year Romanian single-centre experience

**Published:** 2016-10-23

**Authors:** Cristiana Oprea, Irina Ianache, Ionut Popa, Luminita Ene, Roxana Radoi, Gratiela Tardei, Eugenia Ungureanu, Simona Erscoiu, Emanoil Ceausu, Petre Calistru

**Affiliations:** 1 Infectious Diseases, Carol Davila University of Medicine and Pharmacy, Bucharest, Romania; 2 Infectious Diseases, Victor Babes Clinical Hospital for Infectious and Tropical Diseases, Bucharest, Romania; 3 Laboratory of Virology and Immunology, Victor Babes Clinical Hospital for Infectious and Tropical Diseases, Bucharest, Romania

## Abstract

**Introduction**: Despite the global decline in the cART era, HIV-associated neurologic opportunistic infections (CNS-OIs) remain an important cause of morbidity and mortality especially in resource-limited settings. The aim of our study was to evaluate the incidence of CNS-OIs (including brain tumors) and the factors related to survival in HIV-infected patients admitted in a tertiary health care facility.

**Methods**: Retrospective study on HIV-infected patients diagnosed with CNS-OIs at Victor Babes Hospital Bucharest between January 2006 and December 2015. We evaluated demographic, immunologic, virologic variables and treatment characteristics in patients with CNS-OIs. Survival distribution was estimated using Kaplan-Meier methods.

**Results**: A total of 215 patients, 56.2% males, were diagnosed with 220 CNS-OIs (incidence 12.9/1000 PY). The median age at CNS-OIs diagnosis was 29 years (IQR 23–40). The main routes of HIV acquisition were: heterosexual contact (HSX) 52.7%, parenteral in early childhood (PI) 38.5% and injecting drug use (IDU) in 6.8%. The median CD4 cell count and HIV viral load (VL) at CNS-OIs diagnosis were 35/µL (IQR 13–86) and 5.24 log10 copies/mL (IQR 4.1–5.7), respectively. The most common CNS-OIs were: cerebral toxoplasmosis 64 (29.0%), progressive multifocal leukoencephalopathy (PML) 62 (28.1%), tuberculous meningitis (TBM) 41 (18.6%), cryptococcal meningitis (CM) 37 (16.8%), primary cerebral lymphoma (PCNSL) 10 (4.5%) and CMV encephalitis (CMVE) 6 (2.7%). Ninety patients (41.8%) with therapeutic failure on cART, mainly due to non-adherence, developed CNS-OIs. In 74 (33.6%) cases, HIV and CNS-OIs were diagnosed simultaneously. PI patients were younger at both HIV and CNS-OIs diagnosis compared with HSX and IDUs (11, 22 vs. 35, 38 vs. 31, 31 years), respectively (p<0.001), and developed more often PML (43.5% vs. 20.6% vs. 0.0%, p<0.0001). Patients diagnosed with CM and CMVE had the lowest nadir and median CD4 cell count and higher VL at diagnosis (Table 1). The overall mortality rate was 42.7%. Compared to HSX and IDUs, PI had a lower mortality rate (29.4% vs. 51.7%, 46.6%, p=0.006) and a longer survival time (months) (35.3 vs. 7.4, 16.2 respectively, p=0.001). IDU and HSX mode of HIV transmission, low CD4 cell count (<100/µL), low nadir CD4 cell count (<35/µL) and high VL (>5 log10 copies/mL) were associated with shorter median survival time and higher mortality in all cases.

**Conclusions**: The incidence and mortality rate of CNS-OIs in Romanian HIV-infected patients were high. HIV-related mortality and morbidity in patients with CNS-OIs was increased due to late presentation and/or non-adherence to cART.

**Abstract P150–Table 1:** Immuno-virologic characteristics, mortality and survival in HIV-infected patients with CNS-OIs

	PML n = 62	TBM n = 41	CM n = 37	PCNSL n = 10	CMVE n = 6	Toxo n = 64	p
CD4 cell count/mm^3^, median (IQR)	39 (16–103)	65 (23–122)	21 (11–56)	40 (16–78)	23 (11–48)	29 (11–63)	0.08
Nadir CD4 cell count/mm^3^, median (IQR)	33 (12–77)	37 (19–65)	13 (8–28)	32 (11–41)	13 (8–21)	23 (11–54)	0.06
HIV RNA log10 copies/mL, median (IQR)	4.76 (2.85–5.39)	5.32 (4.46–5.84)	5.37 (4.61–5.76)	5.71 (5.14–5.87)	5.89 (5.58–5.92)	5.33 (4.97–5.80)	0.007
Survival in months, median (IQR)	22.1 (3.15–5.12)	14.9 (4.0–27.6)	16.2 (2.3–44.3)	2.3 (1.2–4.1)	38 (11.8–78.9)	17.8 (2.7–43.1)	0.08
Mortality, n (%)	23 (37.0)	20 (48.7)	18 (48.6)	7 (70.0)	2 (33.3)	24 (37.5)	0.31
Early mortality, n (%)	16 (25.8)	8 (19.5)	12 (32.4)	7 (70.0)	1 (16.6)	19 (29.6)	0.05

# Sub article: P151 In spite of international guidelines, vaccine coverage of HIV-infected patients remains low

**Published:** 2016-10-23

**Authors:** Amandine Gagneux-Brunon, Anne Frésard, Maelle Detoc, Elena Charrière, Véronique Ronat, Frédéric Lucht, Elisabeth Botelho-Nevers

**Affiliations:** 1 Infectious and Tropical Diseases, University Hospital of Saint-Etienne, Saint-Etienne, France

## Abstract

**Introduction**: HIV-infected patients are at risk of vaccine-preventable diseases. HIV care is an opportunity to improve vaccine coverage. EACS [1], BHIVA [2] and French [3] guidelines recommend immunization as in general population (tetanus (T), diphtheria (D), poliomyelitis (P), pertussis, and measles, mumps and rubella (MMR)) associated with specific vaccinations (influenza, pneumococcal infections, viral hepatitis B (HBV) and A, human papilloma virus). The aim of our study was to assess the status of immunization of HIV-infected patients for specific and non-specific vaccinations.

**Materials and methods**: Single-centre study, status of immunization was collected in patients’ charts and vaccination booklets. All patients were includable in the study.

**Results**: Five hundred and sixty-nine patients were included, mean age was 49.4±11.5 years, sex ratio was 2.67, 527 (92.6%) patients had an undetectable viral load, median CD4 positive cells count was 660/mm^3^ (53–2146), 217 (38.1%) patients had at least one significant comorbidity (liver disease in 21.2%, diabetes in 12.9%, chronic obstructive pulmonary disease in 7.3%, renal insufficiency in 5.1%, chronic cardiopathy in 4.6%, neoplasm in 1.4%). Two hundred and sixty-one patients of 425 (61.4%) were correctly immunized against D, T and P, 96/356 (27%) against pertussis, 26/279 (9.3%) against MMR. Only 17 patients of 279 with available information were correctly immunized against D, T, P, pertussis and MMR. Of 474 patients with available information about immunization against HBV, 282 (59.5%) were correctly immunized (after immunization or with natural immunity). Concerning immunization against *Streptococcus pneumoniae*, 107 patients (26.5%) (of 403 with available data) received a conjugate vaccine before the non-conjugate polysaccharide vaccine. Two hundred and twenty-nine (52.5% of the patients with data) were immunized against influenza during last winter. Patients with comorbidities were more often correctly immunized against *Streptococcus pneumoniae* and influenza than patients without a comorbidity (51/147 vs. 56/256, p=0.005, and 89/151 vs. 140/284, p=0.036, respectively).

**Conclusion**: Our data suggest that vaccine coverage in HIV-infected patients remains low (and comparable to previously published studies, and lower than in the general population), patients with a comorbidity were more likely correctly immunized against *Streptococcus pneumoniae* and influenza than patients without comorbidity. With the improvement of the condition of HIV-infected patients, physicians involved in HIV care may pay more attention to prevention.

## References

[1] European AIDS Clinical Society EACS Guidelines 8.0. http://www.eacsociety.org/guidelines/eacs-guidelines/eacs-guidelines.html.

[2] Rubin LG, Levin MJ, Ljungman P, Davies EG, Avery R, Tomblyn M (2014). 2013 IDSA clinical practice guideline for vaccination of the immunocompromised host. Clin Infect Dis.

[3] HCSP Vaccinations des personnes immunodéprimées ou aspléniques. Recommandations.

# Sub article: P152 Does syphilis impact on HIV infection when both diagnoses are concomitant?

**Published:** 2016-10-23

**Authors:** Rosario Palacios, Carmen María González-Domenech, Isabel Antequera, Josefa Ruiz-Morales, Enrique Nuño, Encarnación Clavijo, Manuel Márquez, Jesús Santos

**Affiliations:** 1 UGC Enfermedades Infecciosas, Hospital Virgen de la Victoria, Málaga, Spain

## Abstract

**Introduction**: Syphilis causes viral load blips in virologically suppressed patients on antiretroviral therapy, as well as a reduction in the CD4 lymphocyte count [1,2]. The importance of this interaction is that co-infection increases the risk of HIV transmission [3]. The aim of this study was to examine whether syphilis impacts on HIV infection when both infections are diagnosed at the same time in men who have sex with men (MSM).

**Materials and methods**: All cases of HIV-MSM diagnosed at our centre in 2009 to 2015 were reviewed. Patients were excluded from this study if they had a prior diagnosis of syphilis in order to avoid confounding factors in the serological tests. We examined epidemiological, clinical, immunological and virological variables among the patients with and without syphilis at the time of diagnosis of HIV infection. Diagnostic criteria for syphilis are: treponemal and rapid plasma reagin (RPR) both positive, except for patients with primary syphilis, who only require a positive RPR.

**Results**: During the study period, 566 patients were diagnosed with HIV infection (446 MSM); 37 patients were excluded, so the final sample included 409 MSM. Of these, 72 (17.6%) were diagnosed with syphilis at the same time as their diagnosis of HIV infection. Syphilis was asymptomatic in 34 (47.2%) cases. The epidemiological and clinical characteristics were similar in patients with or without syphilis, and no differences were found in basal viral load (4.67 vs. 4.66 log copies/mL; p=0.3) or CD4 cell count (431 vs. 428 cell/µL; p=0.7). Nor were there differences between the patients with symptomatic syphilis and the patients without syphilis.

**Conclusions**: Syphilis does not impact on the clinical presentation nor on the immunovirological parameters when the diagnoses of both syphilis and HIV are coincident. The specific weight that *Treponema pallidum* infection may have on HIV-infected patients not on antiretroviral therapy is minimum.

## References

[1] Palacios R, Jiménez-Oñate F, Aguilar M, Galindo MJ, Rivas P, Ocampo A (2007). Impact of syphilis infection on HIV viral load and CD4 cell counts in HIV-infected patients. J Acquir Immune Defic Syndr.

[2] Jarzebowski W, Caumes E, Dupin N, Farhi D, Lascaux AS, Piketty C (2012). Effect of early syphilis infection on plasma viral load and CD4 cell count in human immunodeficiency virus-infected men: results from the FHDH-ANRS CO4 cohort. Arch Intern Med.

[3] Dong Z, Xu J, Zhang H, Dou Z, Mi G, Ruan Y (2014). HIV incidence and risk factors in Chinese young men who have sex with men – a prospective cohort study. PLoS One.

# Sub article: P153 Health-related costs in chronic HIV infection: a case-control study versus general population using a claims-based approach in Germany

**Published:** 2016-10-23

**Authors:** Eva Wolf, Stefan Christensen

**Affiliations:** 1 Clinical Research, MUC Research, Munich, Germany; 2 Infectious Diseases, Center for Interdisciplinary Medicine (CIM), Munich, Germany

## Abstract

**Introduction**: Due to very effective ART available for HIV treatment, people living with HIV (PLHIV) are now older but also suffering from age-related comorbidities, transforming HIV management into chronic care. However, data on excess comorbidity burden in PLHIV are limited. This study characterizes the cost to the health system, of managing comorbidities of HIV patients, compared with a matched, HIV-negative control cohort in Germany.

**Materials and methods**: This is a retrospective health insurance claims database analysis, comparing the healthcare costs of an HIV cohort (HIV+) to a matched cohort from the general population (non-HIV). Inclusion criteria for HIV+ cohort were ≥1 HIV ICD-10-GM code in 2014, age >21 years at index date, and continuous documentation for the previous 3 years. Index date was the last available HIV diagnosis code. A control cohort was selected from general, non-HIV population, and paired (2:1 control-to-case ratio) based on age, gender, residence district, health insurance status and educational level, at index date. Level of significance was α<0.05.

**Results**: One thousand nine hundred and sixty-nine HIV+ patients were included and paired with 3938 non-HIV individuals. Mean age was 48 years and 83.5% were males. Cardiovascular disease, chronic renal disease, osteoporotic bone fractures and HBV and HCV co-infection were more prevalent in HIV+ patients. The total average per patient per year (PPPY) costs (±SD, standard deviation) excluding costs exclusively related with HIV (ART) were significantly higher (p<0.05) in HIV+ compared wiht non-HIV (8039€±42,586€ vs. 3664€±20,961€, respectively). When looking at individual categories in Figure 1, the main driver of this significant difference is the PPPY pharmaceutical cost excluding ART (3942€), which accounts for nearly 49% of total costs for HIV+, but only 32% for non-HIV (1201€). Outpatient costs and inpatient costs were also statistically higher for HIV+ compared with non-HIV. There was no difference for sick leave payments, and devices costs for HIV+ compared with non-HIV cohort.

**Conclusions**: Higher inpatient, outpatient and drug-related costs not associated with ART were observed in a German HIV+ cohort compared with a matched non-HIV cohort using health insurance claims data. With effective ART, PLHIV are ageing and developing chronic comorbidities, potentially requiring a holistic, long-term, multidisciplinary approach, including not only careful consideration of ART choice, but also screening, monitoring and treatment of comorbidities and aspects of lifestyle, potentially leading to improved outcomes.

**Abstract P144–Figure 1:** ROC curve.

# Sub article: P154 Ageing and the evolution of comorbidities among HIV patients in the EuroSIDA cohort

**Published:** 2016-10-23

**Authors:** Sara Lopes, Ole Kirk, Jens Lundgren, Kamilla Laut, Simon Edwards, Claudine Duvivier, Christoph Stephan, Helen Sambatakou, Katarzyna Maciejewska, Filipa Aragão, Amanda Mocroft

**Affiliations:** 1 Health Economics and Outcomes Research, Gilead Sciences, London, UK; 2 Department of Infectious Diseases, Centre for Health and Infectious Disease Research, Copenhagen, Denmark; 3 Academic Department of Genitourinary Medicine, Mortimer Market Centre, London, UK; 4 Service de Maladies Infectieuses et Tropicales, Hôpital Necker-Enfants Madales, Centre d'Infectiologie Necker-Pasteur, Université Paris Descartes, Paris, France; 5 Zentrum der Inneren Medizin II, JW Goethe University Hospital, HIV Haus 68, Frankfurt am Main, Germany; 6 2nd Department of Internal Medicine, Hippokration General Hospital, Athens, Greece; 7 Clinic of Infectious, Tropical Diseases and Immune Deficiencies, Pomeranian Medical University, Szczecin, Poland; 8 HIV Epidemiology and Biostatistics Unit, Department of Infection and Population Health, University College London, London, UK

## Abstract

**Introduction**: The prevalence of age-related comorbidities is likely to increase as HIV+ patients prolong survival due to availability of effective ART. We aimed to characterize the common comorbidities’ prevalence and their risk and factors, such as renal impairment, bone fractures and cardiovascular (CV) events over time after standardization for age.

**Materials and methods**: Two cross-sectional analyses (2006 and 2014) were conducted in patients within EuroSIDA cohort. Adult patients were selected if they had ≥1 clinical visit in the year of analysis. Analyzed outcomes included prevalence of comorbidities: CV events, renal impairment (chronic kidney disease (CKD); defined as a confirmed (>3 months apart) eGFR <60, nadir eGFR <60 mL/min using CKD-EPI formula) and bone fractures. Risk factors considered included diagnosis of hypertension (systolic blood pressure (BP) ≥140 mm Hg and/or diastolic BP ≥90 mmHg and/or on hypertensive drugs), dyslipidaemia (total cholesterol ≥6.2 mmol/L, HDL ≤0.9 mmol/L or triglycerides ≥2.3 mmol/L) and diabetes (clinical diagnosis and/or antidiabetics/insulin use); and the risk score for CV and CKD development using Data Collection on Adverse Events of Anti-HIV Drugs (DAD) CKD risk score, DAD CVD 5-year risk score and Framingham 10-year CVD risk score.

**Results**: Nine thousand five hundred and fifty-four patients were under follow-up in 2006 and 11,504 in 2014. 73.6% and 71.9% of patients were male in 2006 and 2014, respectively. Figure 1 summarizes the prevalence of comorbidities and risk factors for all, and patients ≥50 years in 2006 and 2014, who represent 44.0% of the 2014 cohort. Overall, the prevalence of CKD increased over time (2.0% vs. 5.1%), as did for any bone fractures (2.0% vs. 5.3%), hypertension (25.8% vs. 40.6%), diabetes (5.9% vs. 6.8%) and dyslipidaemia (69.4% vs. 72.3%). Similarly, the proportion of patients in DAD CKD high-risk group (score ≥5) increased from 50.5% to 55.6%, in Framingham high-risk group (score >20%) increased from 18.3% to 24.9% and in DAD CVD high- and very high-risk groups (scores 5–10% and >10%, respectively) increased from 9.5% to 15.3%, respectively. The increase in the prevalence of CKD, hypertension and fractures over time was notable amongst those ≥50 years.

**Conclusions**: As persons with HIV age, there is an increasing prevalence of common underlying comorbidities. Careful consideration of modifiable factors, including lifestyle and antiretroviral therapy as well as a multidisciplinary approach to managing HIV+ patients with different comorbidities, may help improve patient outcomes.

**Abstract P153–Figure 1:** Mean per person per year (PPPY) individual categories cost per cohort.

# Sub article: P155 Future challenges for clinical care of an ageing population infected with HIV: a “geriatric HIV” modelling study

**Published:** 2016-10-23

**Authors:** Davide De Francesco, Andrea Malagoli, Olga Theou, Stefano Zona, Federica Carli, Giovanni Dolci, Cristina Mussini, Susan Kirkland, Chiara Mussi, Matteo Cesari, Kenneth Rockwood, Giovanni Guaraldi

**Affiliations:** 1 Department of Infection & Population Health, University College London, London, UK; 2 Department of Medical and Surgical Sciences, University of Modena and Reggio Emilia, Modena, Italy; 3 Geriatric Medicine Research, Dalhousie University, Halifax, Canada; 4 Department of Community Health & Epidemiology, Dalhousie University, Halifax, Canada; 5 Geriatrics Division, University of Modena and Reggio Emilia, Modena, Italy; 6 Centre Hospitalier Universitaire de Toulouse, Gérontopôle, Toulouse, France

## Abstract

**Introduction**: According to a modelling study, in 15 years’ time multimorbidity in HIV patients will be the norm [1]. In this context frailty, geriatric syndromes and disability will be relevant clinical outcomes. We aimed at quantifying the scale of change in frailty and its implications for HIV care in Italy in the year 2030.

**Materials and methods**: An individual-based model of the ageing population of the Modena HIV Metabolic Clinic (MHMC) was constructed using data collected between 2009 and 2015 from 3086 patients. The model follows patients enrolled to the clinic up to 2015 and generates new entries on a yearly basis up to 2030. Number, age and gender of new entries were modelled using trends observed in the period 2009 to 2015. Patients were followed as they age and accumulate deficits, resulting in the Frailty Index (FI, quantified as the proportion of deficits present out of a total of 37). FI at enrolment was generated from a gamma distribution with age- and gender-specific parameters estimated using the MHMC 2009 to 2015 data. Patients were classified as non-frail (FI 0–0.3), frail (0.3–0.4) and most-frail (FI>0.4). Changes in the FI over a 1-year period and death rates were modelled following a validated mathematical model developed in a large Canadian ageing population [2], with parameters adjusted to best represent the changes observed in the MHMC 2009 to 2015 population. Geriatric syndrome was defined as of one or more self-reported falls in the past 12 months. Disability was assessed in eight categories of activities of daily function and defined as impairment in ≥1 categories. The relationship between age, gender, geriatric syndrome and disability, observed in 2014 to 2015 at MHMC, was postulated to constant over time.

**Results**: Our model suggests that the median age of HIV-positive patients on combination antiretroviral therapy will increase from 49 years in 2015 to 59 in 2030, with the proportion of HIV-positive patients aged ≥50 years increasing from 42% in 2015 to 95% in 2030 (Figure 1). In the same period, the proportion of frail and most-frail patients will increase from 26% to 28% and from 24% to 48%, respectively. In 2030, we predict that 30% of HIV-positive patients will have geriatric syndrome and 34% will be disabled (Figure 2).

**Conclusion**: The increasing numbers of older patients with frailty, geriatric syndromes and disability depict a “geriatric HIV” scenario. This model suggests evidence-based screening and monitoring protocols to ensure high-quality care.

**Abstract P154–Figure 1:** Prevalence of comorbidities and risk factors in 2006 and 2014 for all patients and ≥50 years.

**Abstract P155–Figure 1:** Observed (red box) and predicted distribution of disability (a) and geriatric syndromes (b) in HIV-positive patients between 2014 and 2030.

## References

[1] Smit M, Brinkman K, Geerlings S, Smit C, Thyagarajan K, van Sighem A (2015). Future challenges for clinical care of an ageing population infected with HIV: a modelling study. Lancet Infect Dis.

[2] Mitnitski A, Song X, Rockwood K (2012). Trajectories of changes over twelve years in the health status of Canadians from late middle age. Exp Gerontol.

# Sub article: P156 Quantifying the future clinical burden of an ageing HIV-positive population in Italy: a mathematical modelling study

**Published:** 2016-10-23

**Authors:** Mikaela Smit, Rachel Cassidy, Alessandro Cozzi-Lepri, Enrico Girardi, Alessia Mammone, Andrea Antinori, Gioacchino Angarano, Francesca Bai, Stefano Rusconi, Giacomo Magnani, Antonella D'Arminio Monforte, Timothy Hallett

**Affiliations:** 1 Department of Infectious Disease Epidemiology, Imperial College London, London, UK; 2 Department of Infection and Population Health, Division of Population Health, University College London, London, UK; 3 Department of Epidemiology, National Institute for Infectious Diseases “Lazzaro Spallanzani”, Rome, Italy; 4 Clinical Department, National Institute for Infectious Diseases “Lazzaro Spallanzani”, Rome, Italy; 5 Clinic of Infectious Diseases, University of Bari, Bari, Italy; 6 Department of Health Sciences, Clinic of Infectious and Tropical Diseases, ASST Santi Paolo e Carlo, University of Milan, Milan, Italy; 7 Dipartimento di Scienze Biomediche e Cliniche ‘Luigi Sacco’, Division of Infectious and Tropical Diseases, University of Milan, Milan, Italy; 8 Department of Infectious Diseases, Azienda Ospedaliera S.Maria Nuova, Reggio Emilia, Italy

## Abstract

**Introduction**: Effective HIV treatment is extending life expectancy of HIV-positive people, putting them at risk of suffering from age-related non-communicable diseases (NCDs). Health systems must prepare for the changes and forecasts are needed. As HIV epidemics across countries vary in terms of at-risk populations and lifestyle factors, it is important to develop country-specific estimates of future morbidity and disease burden. We developed a model of an ageing HIV-positive population for Italy, to provide the first ever national forecasts.

**Materials and methods**: An individual-based model of the ageing HIV-positive population was adapted to the Italian setting. The model follows patients on HIV treatment as they age, and develop NCDs, including cardiovascular disease (CVD; hypertension, hypercholesterolaemia, strokes and myocardial infractions), diabetes mellitus, chronic kidney disease and non-AIDS malignancies. The model also simulates how certain NCDs can increase the risk of developing other NCDs (e.g. how hypertension can increase the risk of CVD). The model was parameterized using data from 2774 HIV-positive patients seen for HIV care between 1997 and 2010 from the ICONA Foundation study, a large cohort encompassing 42 infectious disease centres across Italy. Extensive model validation was carried out on this dataset. National level forecasts were developed by scaling the results to national HIV surveillance and programme data. The model was used to make demographic and epidemiological forecasts from 2015 to 2030.

**Results**: The model estimates that the mean age for HIV-positive patients on treatment in Italy will increase from 45.8 years in 2015 to 48.8 years in 2020 and 54.5 by 2030, with the proportion of HIV-positive patients aged ≥50 increasing from 35% to 41% to 62%, respectively. The model predicts that, by 2030, 47% of HIV-positive patients will suffer from ≥3 NCDs (compared with 27% in 2020 and 16% in 2015) and 92% from ≥1 NCDs. This will be driven by a steep increase in the burden of CVD (Figure 1). The demographic predictions suggest faster ageing and higher predicted NCD burden for 2030 than the Netherlands.

**Conclusions**: The age of HIV-positive patients on treatment in Italy is rising and will be accompanied by a rapid increase in NCD-related multimorbidity, assuming current demographic and epidemiological trends remain constant. These changes will have important and far-reaching consequences for HIV-positive patient care, requiring future HIV care to be able to respond to rising complexity of individualized patient needs.

**Abstract P155–Figure 2:** Observed (red box) and predicted age distribution (a) and burden of frailty (b) in HIV-positive patients between 2009 and 2030.

# Sub article: P157 Quantifying the future clinical burden of an ageing HIV-positive population in the USA: a mathematical modelling study

**Published:** 2016-10-23

**Authors:** Mikaela Smit, Rachel Cassidy, Timothy Hallett

**Affiliations:** 1 Department of Infectious Disease Epidemiology, Imperial College London, London, UK

## Abstract

**Introduction**: Effective HIV treatment is extending life expectancy of HIV-positive people, putting them at risk of suffering from age-related non-communicable diseases (NCDs). Health systems must prepare for the changes and forecasts are needed. As HIV epidemics across countries vary in terms of at-risk populations and lifestyle factors, it is important to develop country-specific estimates of future morbidity and disease burden. We developed a model of an ageing HIV-positive population for the United States, to provide the first ever national forecasts.

**Materials and methods**: An individual-based model of the ageing HIV-positive population was adapted to the US setting. The model follows patients on HIV treatment as they age, and develop NCDs, including cardiovascular disease (CVD; hypertension, hypercholesterolaemia, strokes and myocardial infarctions), diabetes mellitus, chronic kidney disease, and non-AIDS malignancies. The model also simulates how certain NCDs can increase the risk of developing other NCDs (e.g. how hypertension can increase the risk of CVD). The model was parameterized using data from 3087 HIV-positive patients between 2005 and 2010 from a retrospective analysis of a cohort of commercially insured HIV-positive patients in the United States drawn from a geographically representative national sample. Extensive model validation was carried out on this dataset. National level forecasts were developed by scaling the results to national HIV surveillance and programme data. The model was used to make demographic and epidemiological forecasts from 2015 to 2030.

**Results**: The model estimates that the mean age for HIV-positive patients on treatment in the United States will increase from 49.0 years in 2015 to 51.6 years in 2020 and 56.3 by 2030, with the proportion of HIV-positive patients aged ≥50 increasing from 42% to 52% to 71%, respectively. The model predicts that, by 2030, 41% of HIV-positive patients will suffer from ≥3 NCDs (compared with 23% in 2020 and 12% in 2015) and 89% from ≥1 NCDs. This will be driven by a steep increase in the burden of CVD (Figure 1). The demographic predictions suggest faster ageing and higher predicted NCD burden for 2030 than the Netherlands.

**Conclusions**: The age of HIV-positive patients on treatment in the United States is rising and will be accompanied by a rapid increase in NCD-related multimorbidity, assuming current demographic and epidemiological trends remain constant. These changes will have important and far-reaching consequences for HIV-positive patient care, requiring future HIV care to be able to respond to rising complexity of individualized patient needs.

**Abstract P156–Figure 1:** Predicted burden of NCDs amongst HIV-positive patients on treatment in Italy, as simulated by the model. *The bar graph shows the number of people living with HIV (PLHIV) on HIV treatment with 0, 1, 2 or ≥3 comorbidities between 2010 and 2030, while the Venn diagram represents the relative number of HIV-positive patients with the three most prevalent comorbidities and their overlap in 2030*

# Sub article: P158 Silver champions from the GEPPO cohort: a case-control study of people between 65 and 75 years old and above 75 years of age addressing comorbidities, multimorbidity and polypharmacy

**Published:** 2016-10-23

**Authors:** Giovanni Guaraldi, Andrea Malagoli, Giovanni Dolci, Andrea Calcagno, Chiara Mussi, Benedetto Maurizio Celesia, Federica Carli, Stefania Piconi, Giuseppe De Socio, Anna Maria Cattelan, Giancarlo Orofino, Agostino Riva, Valentina Morena, Emanuele Focà, Silvia Nozza, Elva Bugliosi, Giovanni Di Perri

**Affiliations:** 1 Infectious Diseases Unit, University of Modena and Reggio Emilia, Modena, Italy; 2 Infectious Diseases Unit, University of Torino, Torino, Italy; 3 Centro di Valutazione e Ricerca Gerontologica, University of Modena and Reggio Emilia, Modena, Italy; 4 Department of Clinical and Molecular Biomedicine, University of Catania, Catania, Italy; 5 1st Division of Infectious Diseases, Ospedale Luigi Sacco, University of Milano, Milano, Italy; 6 Department of Infectious Diseases, Azienda Ospedaliero-Universitaria di Perugia, Perugia, Italy; 7 Unit of Infectious Diseases, Azienda Ospedaliera-Universitaria di Padova, Padova, Italy; 8 Ospedale Amedeo di Savoia, Unit of Infectious Diseases, Divisione A, Torino, Italy; 9 3rd Division of Infectious Diseases, Ospedale Luigi Sacco, University of Milano, Milano, Italy; 10 Department of Infectious and Tropical Diseases, University of Brescia, Brescia, Italy; 1 Department of Infectious Diseases, San Raffaele Scientific Institute, Milano, Italy; 2 HIV, ViiV Healthcare, Verona, Italy; 3 Unit of Infectious Diseases, University of Torino, Torino, Italy

## Abstract

**Introduction**: GEPPO is a new Italian HIV geriatric cohort which aims to describe health transition over time in HIV-positive patients above 65 years as compared with HIV-negative subjects. The objective of this analysis was to describe multimorbidity, polypharmacy and antiretrovirals’ use in the subset of people between 65 and 75 and above 75 years of age.

**Materials and methods**: Cross-sectional study comparing HIV+ patients and HIV− individuals referred to a cardiovascular screening clinic in a geriatric centre. They were matched for age (±4 years) and sex. Multimorbidity (MM) was classified as the presence of three or more of non-infectious comorbidities, polypharmacy (PP) as the use of five or more medications (excluding ART). Patients were stratified according to the duration of HIV infection (>20, 10–20 and <10 years).

**Results**: A total of 1652 patients were included (1276 HIV+ and 376 HIV−). Table 1 describes the study population between 65 and 75 years of age, whereas Table 2 describes the study population above 75 years of age.

Logistic regression analyses were performed to identify predictors of MM and PP comparing HIV patients versus controls (Figure 1).

**Discussion**: This study takes advantage of the survival bias unavoidable in any ageing cohort to describe the clinical and HIV characteristic of HIV ageing champions. In this extreme age group HIV duration >20 years is a major driver for polypharmacy.

**Abstract P158–Table 1:** Baseline characteristics of the study population between 65 and 75 years of age

	Total (n = 1111)	HIV− (n = 153)	HIV+ (n = 958)	HIV− vs. HIV +	HIV+ <10 years (n = 211)	HIV+ 10–20 years (n = 436)	HIV+ >20 years (n = 311)	HIV+ duration	HIV− vs. HIV+ duration
	
Variable	Mean (SD or%)	Mean (SD or%)	Mean (SD or%)	p	Mean (SD or%)	Mean (SD or%)	Mean (SD or%)	p	p
Age	69.11 (2.62)	68.95 (2.73)	69.12 (2.6)	0.47	69.32 (2.56)	69.13 (2.57)	68.98 (2.67)	0.26	0.37
Females	177 (15.71%)	24 (15.69%)	150 (15.66%)	1	34 (16.11%)	67 (15.37%)	49 (15.76%)	0.97	0.99
BMI	26.52 (9.83)	28.72 (3.92)	26.07 (10.63)	<0.01	28.06 (21.1)	25.83 (4.25)	25.06 (4.18)	<0.01	<0.01
Current smokers	261 (27.19%)	28 (19.18%)	230 (28.5%)	0.02	45 (25.71%)	96 (26.37%)	89 (33.21%)	0.11	0.02
Hypertension	502 (61.9%)	102 (66.67%)	399 (61.2%)	0.24	84 (54.9%)	192 (60.19%)	123 (68.33%)	0.04	0.04
T2DM	216 (27.07%)	37 (24.18%)	178 (27.86%)	0.41	32 (21.19%)	80 (25.72%)	66 (37.29%)	<0.01	<0.01
CVD	143 (18.17%)	33 (21.57%)	110 (17.52%)	0.29	17 (11.56%)	50 (16.34%)	43 (24.57%)	<0.01	<0.01
CKD	121 (16.24%)	5 (7.94%)	115 (17.01%)	0.09	25 (15.15%)	52 (15.76%)	38 (20.99%)	0.24	0.09
COPD	59 (7.63%)	17 (11.41%)	41 (6.63%)	0.07	11 (7.59%)	13 (4.38%)	17 (9.66%)	0.07	0.03
Dyslipidaemia	502 (68.67%)	37 (56.92%)	463 (70.15%)	0.04	98 (61.64%)	230 (71.43%)	135 (75.42%)	0.02	<0.01
Multimorbidity	412 (61.31%)	40 (63.49%)	370 (61.36%)	0.84	72 (51.06%)	174 (59.79%)	124 (72.51%)	<0.01	<0.01
Polypharmacy	194 (30.27%)	23 (15.03%)	170 (34.98%)	<0.01	28 (29.47%)	80 (34.48%)	62 (38.99%)	0.3	<0.01

**Abstract P158–Table 2:** Baseline characteristics of the study population above 75 years of age

	Total (n = 1111)	HIV− (n = 153)	HIV+ (n = 958)	HIV− vs. HIV +	HIV+ <10 years (n = 211)	HIV+ 10–20 years (n = 436)	HIV+ >20 years (n = 311)	HIV+ duration	HIV− vs. HIV+ duration
	
Variable	Mean (SD or%)	Mean (SD or%)	Mean (SD or%)	p	Mean (SD or%)	Mean (SD or%)	Mean (SD or%)	p	p
Age	78.66 (3.43)	78.97 (3.49)	78.44 (3.37)	0.06	78.25 (3)	78.65 (3.7)	78.25 (3.06)	0.99	0.28
Females	124 (22.7%)	61 (27.34%)	61 (19.18%)	0.03	11 (15.9%)	31 (19.8%)	19 (20.43%)	0.74	0.13
BMI	26.22 (4.68)	27.39 (5.12)	25.24 (4.01)	<0.01	26.25 (4.18)	24.97 (4.03)	24.93 (3.78)	0.11	<0.01
Current smokers	57 (12.18%)	18 (9%)	39 (14.72%)	0.08	5 (9.09%)	23 (17.97%)	11 (13.41)	0.27	0.09
Hypertension	328 (70.54%)	153 (69.23%)	173 (71.78%)	0.61	41 (70.69%)	88 (73.95%)	44 (68.75%)	0.74	0.81
T2DM	122 (26.87%)	49 (22.27%)	70 (30.3%)	0.07	10 (17.86%)	37 (33.04%)	23 (36.51%)	0.06	0.02
CVD	130 (29.28%)	68 (30.91%)	61 (27.48%)	0.49	17 (31.48%)	25 (23.58%)	19 (30.65%)	0.46	0.54
CKD	83 (23.92%)	11 (10.28%)	72 (30.38%)	<0.01	15 (25.42%)	34 (29.57%)	23 (36.51%)	0.40	<0.01
COPD	66 (15%)	45 (20.55%)	20 (9.13%)	<0.01	4 (7.69%)	9 (8.49%)	7 (11.48%)	0.75	<0.01
Dyslipidaemia	223 (65.01%)	50 (46.73%)	172 (73.5%)	<0.01	28 (49.12%)	97 (84.35%)	47 (75.81%)	<0.01	<0.01
Multimorbidity	228 (71.03%)	70 (65.42%)	156 (73.58%)	0.17	32 (62.75%)	77 (75.49%)	47 (79.66%)	0.11	0.09
Polypharmacy	168 (41.18%)	84 (37.67%)	84 (45.65%)	0.13	23 (54.76%)	33 (36.26%)	28 (54.9%)	0.04	0.02

**Abstract P157–Figure 1:** Predicted burden of NCDs amongst HIV-positive patients on treatment in the United States, as simulated by the model. *The bar graph shows the number of people living with HIV (PLHIV) on HIV treatment with 0, 1, 2 or* ≥*3 comorbidities between 2010 and 2030, while the Venn diagram represents the relative number of HIV-positive patients with the three most prevalent comorbidities and their overlap in 2030*.

# Sub article: P159 Comorbidity in chronic HIV infection: a case-control study in Germany using health insurance claims data

**Published:** 2016-10-23

**Authors:** Stefan Christensen, Eva Wolf

**Affiliations:** 1 Infectious Diseases, Center for Interdisciplinary Medicine (CIM), Munich, Germany; 2 Clinical Research, MUC Research, Munich, Germany

## Abstract

**Introduction**: ART has increased life expectancy of people living with HIV (PLHIV), transforming HIV management into chronic care. In ageing PLHIV the prevalence of comorbidities is increasing. However, data on excess comorbidity burden in PLHIV are inconclusive. This study characterizes the prevalence of comorbidities in an HIV population, compared with a matched, non-HIV control cohort from the general population in Germany.

**Materials and methods**: This is a retrospective health insurance claims database analysis, comparing the prevalence of comorbidities in an HIV cohort (HIV+) to a matched cohort from the general population (non-HIV). Inclusion criteria for HIV+ cohort were ≥1 HIV ICD-10-GM code in 2014, age >21 years at index date and continuous documentation for the previous 3 years. Index date was the last available HIV diagnosis code. A control cohort was selected from a general, non-HIV population, and paired (using a 2:1 control-to-case ratio) based on age, gender, residence district, health insurance status and educational level, at index date.

**Results**: One thousand nine hundred and sixty-nine patients were included in the HIV+ cohort and paired with 3938 individuals of the non-HIV cohort. Mean age was 48 years (SD±12.2) and 83.5% were males. Approximately 21.6% were retired and 49.5% had an educational level equivalent to technician or master craftsman certificate. When looking at specific comorbidities over the previous 12 months, there was a statistically significantly higher prevalence in the HIV+ cohort compared with the non-HIV cohort (12.8% vs. 10.4% respectively; p=0.0056) of cardiovascular disease (CVD), chronic renal disease (CKD; 4.3% vs. 2.4%; p<0.001) and osteoporotic bone fractures (OBF; 6.4% vs. 2.1%; p<0.0001). HBV and HCV co-infection were significantly more prevalent in the HIV+ cohort (p<0.0001). No significant differences were found regarding the prevalence of type II diabetes, dyslipidaemia or alcohol abuse. Hypertension was significantly more prevalent in the non-HIV cohort (29.3% for HIV+ vs. 32.6% non-HIV; p=0.0095). Major depressive disorders were doubled in the HIV cohort (recurrent single episodes) 25.0% (8.4%) versus 12.7% (4.5%).

**Conclusions**: As PLHIV age, and are treated for longer periods, more age-related comorbidities develop, some of which have been associated with ART. This requires a shift in HIV management including regular monitoring and screening for comorbidities, and optimal selection of ART. We show this is of particular relevance as CVD, CKD and OBF are more prevalent in PLHIV versus non-HIV population. Understanding the nature of these differences may optimize treatment and improve patient outcomes.

# Sub article: P160 Burden and determinants of frailty in a cohort of asymptomatic HIV ART-suppressed subjects without known comorbidities

**Published:** 2016-10-23

**Authors:** Serena Vita, Miriam Lichtner, Raffaella Rossi, Irene Pozzetto, Cecilia Tosato, Raffaella Marocco, Paola Zuccalà, Claudia Mascia, Michela Campagna, Gabriella d'Ettorre, Ombretta Turriziani, Francesca Falasca, Camilla Ajassa, Claudio Maria Mastroianni, Vullo Vincenzo

**Affiliations:** 1 Public Health and Infectious Diseases, Sapienza University, Rome, Italy; 2 Public Health and Infectious Diseases, Sapienza University, Polo Pontino, Latina, Italy

## Abstract

**Introduction**: HIV-infected persons are living longer. Survival gains have been accompanied by an incipient burden of key geriatric syndromes, such as frailty, which lead to increased hospitalization and premature death. Frailty is not yet well understood in the context of patients undergoing cART, without known comorbidities. We performed a pilot study to tentatively investigate the determinants of frailty in aviraemic, asymptomatic HIV+ patients.

**Materials and methods**: We enrolled 80 patients with >1 year of successful cART. We excluded patients with active organ disease in the previous 2 years, diabetes mellitus, renal failure, <18 years old and pregnant women. We collected clinical data and measured frailty with the SHARE-FI test, degrees of depression with patient health questionnaire (PHQ)-2 following by PHQ-9 in case of a score >3, cognitive abilities with the Montreal Cognitive Assessment (MoCA). Based on frailty phenotype, three groups were constituted: prefrails, frails and robusts. In these groups plus a group of 20 healthy donors (HD) we determined the inflammatory background, detecting plasmatic soluble (s) CD163, sCD14, IL-6 using ELISA tests. HCV, CMV serology and CMV-DNA-PCR in urine were tested. Non-parametric tests were used for statistical analysis.

**Results**: The study population included 41 males and 39 females, with a median age of 49.5 years, 82.5% Italians, 50% employed, 53.7% smokers, 78.7% heterosexuals. 12.5% presented a frail and 28.8% a prefrail phenotype, 32.5% presented a mood variation and 51.2% a cognitive impairment. The frailty phenotype was associated with male gender (p=0.05), smoker status (p<0.001), HCV serostatus (p=0.03), PI-based therapy (p=0.002) and cognitive impairment (p<0.001); no differences were found in age, CD4 nadir, actual CD4 and year living with HIV, CMV serostatus and CMV-DNA in urine. Moreover, 100% of frails were affected by mood variation, compared with 39% of prefrails and 21% of robust (p<0.001). Frails have shown abnormalities of MoCA in 33% of frails, in 77% of prefrails and in 27% of robusts (p<0.0001). Regarding the plasma levels of sCD163 and sCD14 we observed an increased level compare with HD only in frail and prefrail subjects (p<0.01 and p=0.001, respectively).

**Conclusions**: Although our study population was asymptomatic and without known organ diseases, we found a high prevalence of prefrail and frail patients with cognitive impairment and depression. Factors associated with frailty were smokers status, HCV co-infection, depression, cognitive impairment, PI-based therapy and an increased inflammatory milieu in terms of sCD163 and sCD14. Multivariate analysis with a larger sample is needed to confirm these results.

# Sub article: P161 Current models of care for the management of HIV patients with comorbidities in England: a survey

**Published:** 2016-10-23

**Authors:** Elaney Youssef, Vanessa Cooper, Eileen Nixon, Jaime Vera, Martin Fisher, Juliet Wright

**Affiliations:** 1 Medical Education, Brighton and Sussex Medical School, Brighton, UK; 2 HIV Research, Brighton and Sussex University Hospital NHS Trust, Brighton, UK; 3 Global Health & Infectious Disease, Brighton and Sussex Medical School, Brighton, UK

## Abstract

**Introduction**: The number of people aged ≥50 living with HIV in the UK is rapidly increasing. Effective treatment means HIV is usually well controlled; however, there has been an increase in individuals experiencing comorbid conditions associated with “normal” ageing. This aim of this study was to find out what models of care are currently in place for the management of patients with comorbidities.

**Materials and methods**: A link to an online questionnaire was sent via the British HIV Association (BHIVA) Audit Committee to one HIV clinician in each HIV unit in England.

**Results**: Forty-four units responded. Only 11 units (25%) provided specialized clinics for the management of comorbidities. These included: 1) Specialist clinics for the management of a non-infectious comorbidity (any age) e.g. a liver or renal clinic (n=10). These clinics utilized in-person appointments (n=3), or a combination of virtual and in-person appointments (n=7). They were managed by an HIV clinician and non-HIV clinician together (n=8), HIV clinician with an interest in the specialist area (n=4) or specialist with an interest in HIV (n=4). 2) Services for HIV patients with multiple comorbidities (any age) (n=2). 3) Dedicated clinics for older people (n=5) with eligibility determined by age (≥50 years) or the presence of a comorbidity. Additionally, two HIV units employed a GP on site and two had set up a locally enhanced service providing enhanced primary care for HIV-positive patients. Six HIV units ran nurse-led clinics for patients with comorbid conditions. Co-ordination of care for patients with comorbid conditions was conducted by an HIV specialist doctor (n=27), the patient's GP (n=18), HIV specialist nurse (n=11) or the patient themselves (n=9). Eleven clinics reported using case management for patients with multiple comorbid conditions. Self-management support (e.g. nurse-led or as part of an expert patient programme) for patients with comorbid conditions was provided at 18 HIV units.

**Conclusions**: Only a quarter of the clinics surveyed had set up clinics for the management of comorbidities in people living with HIV. While a variety of different approaches were used, services were usually focused on the management of one comorbidity, and few provided services for multiple comorbidities. This is an increasing priority in the context of an ageing population.

# Sub article: P162 The HIV patient profile in 2013 and 2003: results from the Greek AMACS cohort

**Published:** 2016-10-23

**Authors:** Nikos Pantazis, Maria Chini, Anastasia Antoniadou, Helen Sambatakou, Athanasios Skoutelis, Panagiotis Gargalianos, Vassilios Paparizos, Charalambos Gogos, George Chrysos, Mina Psichogiou, Nikolaos Sipsas, Olga Katsarou, Panagopoulos Periklis, Giota Touloumi

**Affiliations:** 1 Department of Hygiene, Epidemiology and Medical Statistics, Medical School, National and Kapodistrian University of Athens, Athens, Greece; 2 3rd Department of Internal Medicine – Infectious Disease, Red Cross General Hospital, Athens, Greece; 3 4th Department of Internal Medicine, Medical School, Attikon General Hospital, National and Kapodistrian University of Athens, Athens, Greece; 4 2nd Department of Internal Medicine, Medical School, HIV Unit, Hippokration General Hospital, National and Kapodistrian University of Athens, Athens, Greece; 5 Evangelismos General Hospital, 5th Department of Internal Medicine - Division of, Athens, Greece; 6 1st Department of Internal Medicine and Infectious Disease, G. Gennimatas General Hospital, Athens, Greece; 7 AIDS Unit, Clinic of Venereologic & Dermatologic D, Syngros Hospital, Athens, Greece; 8 Department of Internal Medicine & Infectious Diseases, Patras University General Hospital, Patras, Greece; 9 Infectious Diseases Unit, Tzaneion General Hospital of Piraeus, Athens, Greece; 10 1st Department of Internal Medicine, Medical School, National and Kapodistrian University of Athens, Athens, Greece; 1 Infectious Diseases Unit, Department of Pathophysiology, Laikon Athens General Hospital and Medical School, National and Kapodistrian University of Athens, Athens, Greece; 2 Blood Centre, National Reference Centre for Congen, Laikon Athens General Hospital and Medical School, National and Kapodistrian University of Athens, Athens, Greece; 3 Infectious Diseases Unit, 2nd University Department, University Hospital of Alexandroupolis, Democritus University of Thrace, Alexandroupolis, Greece

## Abstract

**Introduction**: ART has improved life expectancy and significantly reduced AIDS-related morbidity/mortality. As people living with HIV (PLHIV) age, prevalence of chronic comorbidities including cardiovascular disease (CVD) and chronic kidney disease (CKD) increase. The aim of this study is to describe the demographics and evolution of HIV disease markers and comorbidities prevalence in PLHIV in Greece in 2003 versus 2013.

**Materials and methods**: Data were derived from AMACS (Athens Multicenter AIDS Cohort Study), a population-based cohort, that prospectively collects anonymized epidemiologic, clinical, laboratory and treatment data for PLHIV in Greece. Two cross-sectional analyses (2003 and 2013) were performed focusing on patient demographics, HIV disease markers, ART, comorbidities prevalence, including CKD, CVD, diabetes, dyslipidaemia and hypertension. CVD risk was estimated by Framingham 10-year Event Risk calculation (FRS); eGFR calculation was based on CKD-EPI formula. Comparisons were based on population average models excluding missing values.

**Results**: Two thousand four hundred and three PLHIV were identified in 2003 and 4910 in 2013 (1730 contributing for both cross-sections). Table 1 details demographics, disease markers and comorbidities for both study years. Individuals in 2013 were on average older, and diagnosed/treated for HIV for longer, compared with those in 2003. In 2013, PLHIV were also more likely to be on ART (particularly on triple regimen), virologically suppressed and with a higher median CD4 count. CKD and dyslipidemia prevalence increased over time. There was an increase in prescription of lipid-lowering treatment (3.5% in 2003 vs. 7.7% in 2013, p<0.001), accompanied by an improvement in LDL, triglycerides and total cholesterol. Among 220 and 879 individuals eligible for FRS calculation, the median score numerically decreased (9.7% in 2003 vs. 8.2% in 2013, p=0.096) but the proportion of patients in the high-risk group (>20%) increased from 18.2% to 22.2%.

**Conclusions**: The availability of new ART and the increased treatment uptake led to significant improvements, within the 2003 to 2013 decade, in the Greek AMACS cohort patients’ immunologic status and viral suppression rates. PLHIV aged alongside an increase in prevalence of comorbidities during these 10 years. The proportion of PLHIV with high FRS increased over time, but the median CVD risk of the cohort slightly declined, which might be partially attributed to the effective lipid control measures. The shift in HIV epidemic paradigm should be addressed with appropriate monitoring and holistic management of HIV care, in terms of optimal ART selection and long-term management and prevention of comorbidities.

**Abstract P162–Table 1:** Patient demographics, HIV markers and comorbidities in 2003 and 2013

	2003 (n = 2403)	2013 (n = 4910)	p
Mean age, years (SD)	41.1 (10.6)	43.8 (11.4)	<0.001
Age ≥50	18.2%	26.9%	<0.001
Median CD4, count/mL (IQR)	493/mL (299–717)	610/mL (425–828)	<0.001
AIDS diagnosis	16.2%	13.0%	<0.001
Median time since diagnosis, years (IQR)	6.0 (2.9–9.0)	6.7 (2.8–13.1)	<0.001
HIV RNA <50 copies/mL	34.1%	72.4%	<0.001
Patients on ART	76.5%	84.4%	<0.001
Median time on ART, years (IQR)	3.8 (0.5–6.4)	4.5 (1.1–11.0)	<0.001
Triple therapy (2 NRTI + 3rd agent)	63.5%	76.9%	<0.001
eGFR <60 mL/min/1.73 m^2^	2.4%	3.4%	0.006
Overall cardiovascular events (ever)	1.8%	2.1%	<0.001
Myocardial infarction (ever)	1.3%	1.7%	0.001
Stroke (ever)	0.3%	0.4%	0.102
Median Framingham risk score (IQR)	9.7% (4.3–17.0)	8.2% (3.9–18.1)	0.096
Patients with high (FRS >20%) 10-year CVD risk	18.2%	22.2%	0.002
Dyslipidemia	64.9%	70.3%	<0.001
Patients on lipid-lowering treatment	3.5%	7.5%	<0.001
Median total cholesterol, mg/dL (IQR)	202 (169–239)	189 (161–220)	<0.001
Median HDL, mg/dL (IQR)	46 (37–55)	43 (36–53)	<0.001
Median LDL, mg/dL (IQR)	122 (97–154)	114 (91–140)	<0.001
Median triglycerides, mg/dL (IQR)	144 (96–237)	123 (86–183)	<0.001
Hypertension	27.0%	27.5%	0.819
Diabetes	6.2%	5.6%	0.028

Percentages calculated after exclusion of missing values. eGFR, estimated glomerular filtration rate; IQR, interquartile range; NRTI, nucleotide reverse transcriptase inhibitor; SD, standard deviation.

# Sub article: P163 Antiretroviral therapy in Italian geriatric patients living with HIV/AIDS: analysis of GEPPO cohort

**Published:** 2016-10-23

**Authors:** Silvia Nozza, Andrea Malagoli, Giovanni Dolci, Andrea Calcagno, Chiara Mussi, Benedetto Celesia, Federica Carli, Stefania Piconi, Giuseppe De Socio, Anna Maria Cattelan, Giancarlo Orofino, Agostino Riva, Valentina Morena, Emanuele Focà, Elva Bugliosi, Giovanni Di Perri, Giovanni Guaraldi

**Affiliations:** 1 Department of Infectious Diseases, San Raffaele Scientific Institute, Milan, Italy; 2 Infectious Diseases Clinic, University of Modena and Reggio Emilia, Modena, Italy; 3 Unit of Infectious Diseases, Department of Medical Sciences, University of Torino, Torino, Italy; 4 Centro di Valutazione e Ricerca Gerontologica, University of Modena and Reggio Emilia, Modena, Italy; 5 University of Catania, ARNAS Garibaldi, Division of Infectious Diseases, Catania, Italy; 6 University of Milano, Ospedale L Sacco, Division of Infectious Diseases Unit, Milan, Italy; 7 Infectious Diseases Clinic, Azienda Ospedaliero-Universitaria di Perugia, Perugia, Italy; 8 Unit of Infectious Diseases, Azienda Ospedaliera-Universitaria di Padova, Padova, Italy; 9 Ospedale Amedeo di Savoia, ASLTO2, Unit of Infectious Diseases, Divisione A, Torino, Italy; 10 Department of Infectious and Tropical Diseases, University of Brescia, Brescia, Italy; 1 ViiV Healthcare, Verona, Italy

## Abstract

**Introduction**: HIV-infected patients above 65 years of age have higher prevalence of comorbidities and polypharmacy. The aim of the study is to describe ARVs’ use in elderly patients living with HIV.

**Materials and methods**: Cross-sectional study analyzing HIV+ patients aged ≥65 years, recruited from 11 HIV outpatients clinics in Italy. Multimorbidity (MM) was classified as the presence of three or more of non-infectious comorbidities in the same individual, including cardiovascular disease, chronic kidney disease, dyslipidaemia, hypertension, type 2 diabetes mellitus and chronic obstructive pulmonary disease. Polypharmacy (PP) was defined as the use of five or more medications. Patients were stratified according to the duration of HIV infection (>20, 10–20 and <10 years). Separate multivariate logistic regression were built to identify predictors of “not conventional” ARV strategies, including unboosted PI, mono/dual, NRTI-spearing and TDF-free.

**Results**: We enrolled 1297 HIV-positive patients: 83.35% males; 12.47% HCV co-infected, 9.6% HBV co-infected; mean age 71.4 years (4.91 SD); mean HIV duration 16.83 years (7.6 SD); mean CD4 nadir 220.28 cells/mm^3^ (177.38 SD); mean current CD4 count 641.96 cells/mm^3^; mean CD4/CD8 ratio 0.98 (1.43 SD); 94.3% out of patients had HIV RNA <40 copies/mL. HIV duration was 280 (21.94%) less than 10 years, 592 (46.39%) between 10 and 20 years, and 404 (31.66%) more than 20 years. ARV prescription: 66.3% triple therapy (third drug: 45.76% NNRTI, 28.35% PI, 15.53% INSTI, 1.41% NRTI, 8.94% other), while 1.64% took more than three drugs. Prevalence of not conventional ARV strategies was: 25.98% dual therapy, 6.08% PI monotherapy, NRTI-sparing regimen 56.9%, unboosted PI 22.61%, TDF-free 70.2%. Total multimorbidity frequency was 49.17%, whereas polypharmacy frequency was 37.89%. Figure 1 shows multivariate logistic regression for use of not conventional ARV strategies. The presence of MM, PP and male gender were predictive for mono/dual and NRTI-sparing regimens. Furthermore, PP was significantly associated with TDF-sparing regimens.

**Conclusions**: Geriatric HIV+ patients have high prevalence of multimorbidity and polypharmacy. A significant proportion of these patients are treated with non-conventional ARV regimens: the selection of ARVs seems to be driven by several factors including MM and PP.

**Abstract P163–Figure 1:** Multivariate logistic regression for four not conventional ARV therapy.

**Abstract P158–Figure 1:** Predictors of multimorbidity and polypharmacy.

# Sub article: P164 CD4/CD8 ratio matters to age-related health outcomes in HIV-infected patients with comorbidities, frailty and disability

**Published:** 2016-10-23

**Authors:** Marianna Menozzi, Ana Rita Domingues da Silva, Andrea Malagoli, Giovanni Dolci, Antonella Santoro, Federica Carli, Cristina Mussini, Giovanni Guaraldi

**Affiliations:** 1 Department of Mother, Child and Adult Medicine and Surgical Science, Infectious Diseases Clinic, University of Modena and Reggio Emilia, Modena, Italy; 2 Department of Infectious Diseases, Hospital Beatriz Ângelo, Loures, Portugal

## Abstract

**Introduction**: Currently, about 50% of HIV-infected persons in high-income countries are older than 50, with increased risk of developing age-related diseases [1]. CD4/CD8 ratio inversion has been associated with non-communicable diseases and frailty phenotype [2,3]. The study objective was to describe associations between CD4/CD8 ratio and meaningful endpoints of ageing with HIV (multimorbidity, frailty and disability).

**Material and methods**: Cross-sectional study. Inclusion criteria: HIV-infected adults, effective antiretroviral treatment (HIV VL <40 copies/mL), access in the Modena HIV Metabolic Clinic within 2008 and 2016. CD4/CD8 ratio cutoff was set at 0.8, multimorbidity (MM) was defined as presence of >2 comorbidities among: cardiovascular events (CVD), chronic kidney disease, hypertension (HTN), chronic obstructive pulmonary disease (COPD), cancer and diabetes mellitus (DM). Frailty was defined as frailty index >0.31 [4]. Disability was defined as presence of ≥1 deficit at IADL questionnaire or SPPB score <9 or falls within the last year. Statistical considerations: after descriptive analysis according to CD4/CD8 ratio, univariate and multivariable logistic regression models adjusted for sex and age were created to assess relation between CD4/CD8 ratio and MM, frailty and disability.

**Results**: 2945 patients were included. Table 1 shows descriptive analysis. Figure 1 depicts logistic regression models to assess CD4/CD8 association with study outcomes: a protective role of CD4/CD8 ratio was confirmed regarding CVD (OR 0.69, 95% CI 0.45–0.94, p=0.038), MM (OR 0.60, 95% CI 0.41–0.88, p=0.009) and frailty (OR 0.65, 95% CI 0.55–0.77, p<0.001). Other factors with positive association were male sex and age. No association was found between CD4/CD8 ratio and disability. At further logistic regression models, higher CD4 cells nadir was positively associated with high CD4/CD8 ratio, while male sex, MM and frailty had a negative significant association.

**Conclusion**: Age-related health outcomes result from concomitant processes of ageing, inflammation, HIV infection, comorbidities and lifestyle. We found independent associations between routinely performed markers of immune reconstitution and important clinical features of ageing HIV-infected patients: low CD4/CD8 ratio was associated with comorbidities alone or aggregated in MM and frailty. This is a novelty presented by our study: previous studies found associations with isolated comorbidities and frailty phenotype. It also confirmed the reverse association between MM, frailty and low CD4/CD8 ratio. Further studies could be important to integrate clinical geriatric syndromes and immunologic elements in the ageing HIV-infected population.

**Abstract P164–Table 1:** Anthropometrical and clinical characteristics of patients

Characteristics	CD4/CD8 ratio <0.8 - N (%) or median (IQR)	CD4/CD8 ratio > = 0.8 - N (%) or median (IQR)	p
Total number of patients	1470 (49)	1475 (51)	
Female sex	391 (27)	550 (37)	<0.001
Age	49 (45–54)	50 (45–54)	0.900
Smokers	775 (53)	854 (58)	0.004
Packyear (if smoker)	20.8 (10–32)	17.9 (9.3–30)	0.004
No physical activity	717 (51)	665 (48)	0.051
Intense alcohol intake (>3/week)	14 (0.9)	16 (1.1)	0.923
BMI	23.8 (21–26)	23.3 (21–25.7)	0.001
HIV duration (months)	241 (158–299)	239 (156–299)	0.427
Risk factor for HIV			<0.001
Intravenous drug users	409 (28)	357 (24)	
Men who have sex with men	453 (31)	407 (27.5)	
Heterosexual	431 (29)	557 (37.8)	
Other	177 (12)	154 (10)	
CDC C stage	375 (25.5)	305 (20.7)	0.005
Age at ARV initiation	36 (31–43)	34 (31–42)	0.005
ARV initiation period			0.106
<1996	437 (30.4)	397 (27.4)	
1996–2005	637 (44)	696 (48)	
≥2006	363 (25)	358 (24.7)	
CD4 cells nadir cells/mm^3^	163 (60–260)	221 (109–330)	<0.001
CD8/CD38 cells count cells/mm^3^	84 (51–152)	58.5 (38–94)	<0.001
LDL cholesterol mg/dL	113 (37–57)	112 (92–136)	0.791
HDL cholesterol mg/dL	45 (35–57)	51 (41–62)	<0.001
Total cholesterol mg/dL	188 (161–226)	189 (165–217)	0.352
Glucose	94 (86–103)	93 (87–102)	0.451
GOT	24 (20–34)	22 (19–31)	<0.001
HOMA index	2 (1.2–3)	1.7 (1.1–2.8)	<0.001
C reactive protein	0.2 (0.13–0.3)	0.2 (0.18–0.22)	0.262
Cardiovascular disease	100 (6.8)	63 (4.3)	0.003
Hypertension	563 (38)	509 (34.5)	0.033
Diabetes mellitus	231 (15.7)	193 (13.1)	0.042
Chronic kidney disease	148 (10.1)	145 (9.8)	0.830
Cancer	39 (3.9)	36 (2.4)	0.026
Chronic obstructive pulmonary disease	57 (3.9)	36 (2.44)	0.026
Multimorbidity	90 (6.1)	55 (3.7)	0.003
Frailty (2643 patients)	722 (54.8)	577 (43.5)	<0.001
IADL deficit (830 patients)	94 (25.3)	118 (25.7)	0.903
SPPB <9 (459 patients)	13 (6.28)	15 (5.95)	0.884
FALLS (665 patients)	57 (19.6)	76 (20.3)	0.815

Comorbidities (CVD, HTN, DM and COPD), MM and frailty prevalence were significantly higher in the low CD4/CD8 ratio group. No differences were found in disability items prevalence.

**Abstract P164–Figure 1:** Logistic regressions for age-related health outcomes.

## References

[1] Nakagawa F, Lodwick RK, Smith CJ, Smith R, Cambiano V, Lundgren JD (2012). Projected life expectancy of people with HIV according to timing of diagnosis. AIDS.

[2] Mussini C, Lorenzini P, Cozzi-Lepri A, Lapadula G, Marchetti G, Nicastri E (2015). CD4/CD8 ratio normalisation and non-AIDS-related events in individuals with HIV who achieve viral load suppression with antiretroviral therapy: an observational cohort study. Lancet HIV.

[3] Erlandson KM, Allshouse AA, Jankowski CM, Lee EJ, Rufner KM, Palmer BE (2013). Association of functional impairment with inflammation and immune activation in HIV type 1-infected adults receiving effective antiretroviral therapy. J Infect Dis.

[4] Guaraldi G, Brothers TD, Zona S, Stentarelli C, Carli F, Malagoli A (2015). A frailty index predicts survival and incident multimorbidity independent of markers of HIV disease severity. AIDS.

# Sub article: P165 Mortality rates and excess mortality among HIV-positive persons according to age in Spain, 2004–2014

**Published:** 2016-10-23

**Authors:** Belen Alejos, Victoria Hernando, Pompeyo Viciana, Marta Montero, Juan Berenguer, Maria Rivero, Victor Asensi, Lucio Garcia-Fraile, Felix Gutierrez, Santiago Moreno, Cohort Coris

**Affiliations:** 1 Institute of Health Carlos III, National Center of Epidemiology, Madrid, Spain; 2 Hospital Virgen del Rocio, CoRIS Cohort, Sevilla, Spain; 3 Hospital La Fe, CoRIS Cohort, Valencia, Spain; 4 Hospital Gregorio Marañon, CoRIS Cohort, Madrid, Spain; 5 Hospital de Navarra, CoRIS Cohort, Pamplona, Spain; 6 Hospital Central de Asturias, CoRIS Cohort, Oviedo, Spain; 7 Hospital La Princesa, CoRIS Cohort, Madrid, Spain; 8 Hospital Elche, CoRIS Cohort, Elche, Spain; 9 Hospital Ramon y Cajal, CoRIS Cohort, Madrid, Spain; 10 Institute of Health Carlos III, CoRIS Cohort, Madrid, Spain

## Abstract

**Introduction**: Success of ART has changed the distribution of causes of death among HIV-positive patients and new causes of death have emerged, such as liver-related or non-AIDS-defining malignancies (NADM). We describe and compare the mortality rates and excess mortality rates according to age in HIV-positive persons in the Cohort of the Spanish AIDS Research Network (CoRIS) from 2004 till 2014.

**Methods**: CoRIS is a multicentre, open, prospective cohort of HIV-infected patients naïve to ART at entry. We calculated: mortality rates (per 1000 py) for overall and cause-specific death comparing aged </≥50 years and excess mortality rates (per 1000 py) comparing overall and cause-specific mortality with that of the general population of similar age and sex. Age was modelled as a time-dependent variable. Death was classified by modified CoDE procedures [1].

**Results**: Overall, 347 deaths were recorded in 9569 (34,385 py of follow-up) persons, leading to a mortality rate of 10.09 per 1000 py (95% CI 9.08–11.21). The proportion of total py aged ≥50 years increased from 8.8 to 21.2%, from 2004 to 2014. The mortality rate ratio was 2.70 (95% CI 2.15–3.38) higher for patients aged ≥50. Forty-three percent of the total deaths were attributable to AIDS, followed by 14% due to NADM, 9% liver-related, 7% other infectious diseases, 3% cardiovascular and 12% for other causes and unknown. For cause-specific deaths, the mortality rate was 4.33 (3.69–5.09) for AIDS-related, 1.40 (1.05–1.85) for NADM, 0.93 (0.66–1.32) for liver-related, 0.67 (0.44–1.01) for other infectious diseases and 0.26 (0.14–0.50) for cardiovascular. Mortality rate ratio was higher for patients ≥50 years for all causes and for cause-specific death. The overall excess mortality rate was 8.81 (per 1000 py) (95% CI 7.87–9.87), being 7.37 (6.44–8.44) for subjects <50 years and 16.65 (13.51–20.53) for ≥50 years. For NADM, excess mortality rate was 0.46 (0.27–0.79) for <50 years and 2.79 (1.67–4.65) for ≥50 years. For liver-related, it was 0.84 (0.56–1.25) for <50 years and 1.13 (0.50–2.52) for ≥50 years. And finally, for other infectious diseases, it was 0.64 (0.43–0.98) for <50 years and 0.54 (0.33–0.89) for ≥50 years.

**Conclusion**: High overall mortality rate and excess mortality rate were observed in HIV-positive subjects, especially among older patients. By cause-specific, higher NADM mortality and excess mortality was observed in patients aged ≥50. However no significant differences by age were detected by liver diseases, although it represents an important cause of death in these patients.

## References

[1] Copenhagen HIV Program Protocol Coding Causes of Death in HIV (CoDe). http://www.cphiv.dk/Portals/_default/pdf_folder/code_protocol_ver_1.0.pdf.

# Sub article: P166 Changes in the prevalence of cardiovascular, renal and bone comorbidities and related risk factors in HIV-infected patients in the Spanish VACH cohort: a cross-sectional study in 2010 and 2014

**Published:** 2016-10-23

**Authors:** Ramon Teira, Ignacio Suarez-Lozano, Maria Galindo, Marta Montero, Paloma Geijo, Agustin Muñoz-Sanz, Elisa Martinez, Pere Domingo, Fernando Lozano, Pompeyo Viciana, Belen de la Fuente, Bernardino Roca, Vicente Estrada, Pepa Muñoz-Sanchez, Study group Vach

**Affiliations:** 1 Infectious Diseases, Hospital de Sierrallana, Torrelavega, Spain; 2 Infectious Diseases, Hospital Infanta Elena, Huelva, Spain; 3 Infectious Diseases, Hospital Clinico, Valencia, Spain; 4 Infectious Diseases, Hospital La Fe, Valencia, Spain; 5 Internal Medicine, Hospital Virgen de la Luz, Cuenca, Spain; 6 Infectious Diseases, Hospital Infanta Cristina, Badajoz, Spain; 7 Internal Medicine, Hospital de Albacete, Albacete, Spain; 8 Infectious Diseases, Hospital Arnau de Vilanova, Lleida, Spain; 9 Infectious Diseases, Hospital de Valme, Sevilla, Spain; 10 Infectious Diseases, Hospital Virgen del Rocio, Sevilla, Spain; 1 Infectious Diseases, Hospital de Cabueñes, Gijon, Spain; 2 Internal Medicine, Hospital General, Castellon, Spain; 3 Infectious Diseases, Hospital Clinico de San Carlos, Madrid, Spain; 4 Infectious Diseases, Hospital de Basurto, Bilbao, Spain; 5 AM-VACH, AM-VACH, Cartaya, Spain

## Abstract

**Introduction**: In 2012, in Central and Western Europe, it was estimated that 33% of HIV patients were 50 years old or older. This will bring new challenges in the management of HIV population: patients are living longer and chronologically ageing; HIV itself has been associated with accelerated ageing and development of comorbidities; certain antiretrovirals are associated with age-related, organ-specific toxicities. Therefore, it is important to characterize the evolution of the prevalence of risk factors and comorbidities to inform management of HIV care in general, and choice of ART in particular.

**Methods**: The VACH cohort is a multicentre Spanish cohort. To be included in the cohort, a patient is required to have confirmed HIV infection, age over 16, and +1 follow-up visit in the cohort's hospitals. All patients receiving ART with at least one visit in 2010 and at least one visit in 2014 were included in this analysis. Two cross-sectional analyses (2010 and 2014) were conducted on this set of patients. Analyzed outcomes included prevalence of: 1) comorbidities: a) cardiovascular events, b) renal impairment, c) bone fractures (any location); 2) risk factors: a) hypertension, b) dyslipidemia, c) diabetes, d) secondary osteoporosis, e) alcohol abuse (exceeding 3 units/day (42g/day)). Descriptive analysis consisted of number of patients/events and its respective percentage over the available information (missing data were not considered).

**Results**: Nine thousand nine hundred and sixty patients met the inclusion criteria and were included in the analysis. Forty-three percent of patients were at least 50 years old in 2014. 73.3% were male and 39% were ever-intravenous drug users. Among ART-experienced, the proportion of patients virologically suppressed increased by 7% and the proportion with CD4 cells count >500 cells/mm^3^ increased by 8% from 2010 to 2014. Figure 1 shows the changes in the prevalence of comorbidities and risk factors between 2010 and 2014.

**Conclusions**: The proportion of patients older than 50 in the VACH cohort has increased significantly, as it has happened with the prevalences of age-related comorbidities in recent years.

**Abstract P166–Figure 1:** Changes in the prevalence of cardivascular, renal and bone comorbidities and related risk factors between 2010 and 2014 in the VACH cohort.

# Sub article: P167 Epidemiologic “snapshot” of the HIV-positive population in Belgium, 2014 to 2016

**Published:** 2016-10-23

**Authors:** Steven Callens, Stephane De Wit, Chloe Wyndham-Thomas, Jean-Christophe Goffard, Michel Moutschen, Christelle Meuris, Charlotte van den Bulcke, Karine Fombellida, Marc del Forge, Homie Razavi, Jessie Gunter

**Affiliations:** 1 Internal Medicine, University of Ghent Hospital, Ghent, Belgium; 2 Infectious Diseases, St Pierre University Hospital, Brussels, Belgium; 3 Immunodeficency Unit/AIDS Reference Center, Erasme Hospital, Brussels, Belgium; 4 Infectious Diseases, Erasme Hospital, Brussels, Belgium; 5 Infectious Diseases, University of Liege – Sart Tilman, Liege, Belgium; 6 Infectious Diseases, University of Ghent Hospital, Ghent, Belgium; 7 Clinical Research Unit, St Pierre University Hospital, Brussels, Belgium; 8 Epidemiology, Center for Disease Analysis, Lafayette, CO, USA

## Abstract

**Introduction**: In most of Western Europe, HIV is considered a chronic disease associated with reasonable life expectancy. Eleven AIDS reference centers and seven reference laboratories have been established across Belgium to handle HIV diagnosis, care and patient tracking. Although Belgium is at the forefront of HIV care, to date, country-wide analysis of data collected at those centers has not been conducted. The purpose of this analysis is to 1) provide an epidemiologic “snapshot” of the HIV-positive population in Belgium, and 2) determine the prevalence of key non-infectious comorbidities (NICMs) that may affect the quality and length of HIV-positive individuals’ lives.

**Materials and methods**: Data were analyzed from four of the largest reference centres in Belgium (St Pierre University Hospital, University of Liege – Sart Tilman, University of Ghent Hospital and Erasme Hospital). In total, 5787 patients met the inclusion criteria of receiving follow-up care at least once between 1 June 2014 and 1 July 2016.

**Results**: Out of 5787 follow-up cases in these four centers in 2014 to 2016, 95% have initiated treatment. The mean age of patients under follow-up was 46.6. The prevalence rates of diabetes mellitus, viraemic hepatitis C and renal disease were 5.9%, 3.1% and 7.7%, respectively. Cardiovascular disease, defined as the occurrence of myocardial infarction, stroke or an invasive coronary procedure, occurred in 3.3% of the patients. The combined prevalence of non-AIDS-defining cancers (defined as anal cancer, liver cancer, Hodgkin's lymphoma and lung cancer) was 0.8% among the cohort population. Specific risk factors were also relatively common among patients: 38% were former or current cigarette smokers and one-third had been diagnosed with hypertension.

**Conclusions**: NICMs in the HIV-positive population such as cardiovascular disease and renal disease are of increasing importance as AIDS-related deaths are delayed through careful patient management. A country-wide analysis of available data, including data on the prevalence of common NICMs, is critical to planning for the healthcare needs of the ageing HIV-positive population in Belgium.

# Sub article: P168 From HIV diagnosis to viral suppression in a cohort of older patients

**Published:** 2016-10-23

**Authors:** Mariana Kundro, Guillermo Viloria, Javier Toibaro, Marcelo Losso

**Affiliations:** 1 HIV Unit, JM Ramos Mejia Hospital, Buenos Aires, Argentina

## Abstract

**Introduction**: The proportion of new HIV diagnoses in later life is increasing. Older adults have clinical features that difference them from younger individuals, constituting a vulnerable population. However, data describing the steps of the care continuum for this age group are scarce.

**Materials and methods**: We conducted a retrospective analysis including all older adults (≥50 years) with a new HIV diagnosis over a 13-year period in a large public hospital in Buenos Aires. We calculated the proportion of patients presenting with advanced stage HIV disease (WHO clinical stage IV or CD4 cell count <200 cells/mL), the proportion of patients who were linked and retained in care, initiated ART according to the WHO recommendations for each year analyzed and were virally suppressed. We also determined the factors associated with loss to follow-up (LTFU) in this age group using multivariate logistic regression.

**Results**: From January 2002 to December 2014, 1239 patients were tested positive for HIV, of whom 218 (17.5%) were older adults. Fifty-three percent of these individuals had advanced HIV disease and 32% were hospitalized at diagnosis. Most patients (93.1%) were linked to care, 11 patients (5%) died before 1 year of follow-up (all of them because of AIDS-related events) and 65.5% were retained into care. Of the 123 patients who started ART, 94 (76.4%) achieved viral suppression. 30.4% of patients were LTFU. The odds of being LTFU were higher for MSM (OR 2.65 (IQR 1.21–5.77)) than for heterosexual patients. Conversely, those who were diagnosed at AIDS events (OR 0.39 (IQR 0.20–0.76)) and patients on antiretroviral therapy (OR 0.21 (IQR 0.06–0.72)) were less likely to be LTFU. Age, gender and educational level were not associated with LTFU in this cohort.

**Conclusions**: This study highlights the need for newer strategies aimed to timely diagnosis in older adults and to develop interventions to prevent LTFU focused on this age group.

# Sub article: P169 The relative impact of antiretroviral drugs and baseline immune status on bone quality in HIV-positive subjects: results from the HIV UPBEAT cohort

**Published:** 2016-10-23

**Authors:** Tara McGinty, Aoife Cotter, Caroline Sabin, Jack Lambert, Gerard Sheehan, Alan Macken, Eoin Kavanagh, Juliet Compston, Paddy Mallon

**Affiliations:** 1 School of Medicine/HIV Molecular Research Group, University College Dublin, Dublin, Ireland; 2 Department of Infection & Population Health, University College London, London, UK; 3 Infectious Diseases, Mater Misercordiae University Hospital, Dublin, Ireland; 4 HIV Molecular Research Group, University College Dublin, Dublin, Ireland; 5 Radiology, Mater Misercordiae University Hospital, Dublin, Ireland; 6 School of Medicine, University of Cambridge, Cambridge, UK

## Abstract

**Objectives**: Trabecular bone score (TBS) is a novel, non-invasive measure of bone microarchitecture that can detect differences in bone quality in individuals with similar bone mineral density (BMD). We have previously shown that lower TBS in HIV-positive subjects is influenced by the high-smoking rates in this population. We now aim to investigate HIV-specific factors associated with TBS.

**Methods**: BMD was measured by dual X-ray absorptiometry (DXA) in HIV-positive subjects from the HIV UPBEAT study; TBS was derived from baseline lumbar spine (LS) DXA images using TBS Insight software (version 2.2.1). Significant between-group differences were assessed using Wilcoxon tests. Univariate linear regression explored the impact of HIV-specific factors including: nadir CD4, current CD4 and CD8 T-cell counts, HIV RNA <40 copies, time from HIV diagnosis and being on ART, on TBS; factors associated with a lower TBS in these models were added to a multivariable model adjusted for LS BMD, demographics, body mass index (BMI) and current smoking.

**Results**: The 201 HIV-positive subjects (40% male, 39% African, median (inter-quartile range (IQR)) age 39 (33–46) years) had HIV diagnosed for a median (IQR) of 4.5 (2–8) years, a median nadir CD4 of 311 (108–306) cells/mm^3^, exposure to ART, protease inhibitors (PI) and tenofovir disproxil fumarate (TDF) was 2.7 (0.5–5), 0.3 (0–2.4) and 1.3 (0–3) years, respectively. ART-naïve patients had higher TBS than those on ART (1.438 (1.306, 1.481) vs. 1.343 (1.258, 1.421), p=0.005). While TDF exposure was not significantly associated with TBS (1.347 (1.270, 1.422) vs. 1.380 (1.265, 1.471), p=0.314), those exposed to PIs had lower TBS (1.323 (1.248, 1.382)) compared with no PI exposure (1.386 (1.289, 1.452), p=0.005). In unadjusted analysis lower TBS was associated with duration of diagnosed HIV, intravenous drug use, nadir CD4, being on ART (specifically PIs) and cumulative PI exposure (all p<0.05). HIV viraemia was not significantly associated with TBS (0.008 (−0.031, 0.048), p=0.69). In adjusted analyses, smoking status, nadir CD4 and receipt of PI-containing ART were independent predictors of lower TBS (Table 1).

**Conclusion**: While smoking remains an independent predictor of TBS in HIV-positive subjects, our results, although derived from an observational study and therefore limited in determining causality, highlight the potential impact of ART on bone quality with PIs, but not TDF, being significantly associated with lower TBS. Baseline immune status was also an independent predictor of TBS suggesting a possible effect of immune activation on bone quality. Further studies should focus on the clinical utility of TBS to monitor bone quality and in fracture risk prediction in HIV-positive persons.

**Table 1:** Results from multivariable linear regression analysis^a^ of associations between smoking status, nadir CD4 count and receipt of PI therapy with TBS

Variable	Effect on TBS	95% confidence intervals	p
Current smoker	−0.047	−0.085, −0.008	0.01
Nadir CD4 T-cell count (per 50 cells/mm^3^ higher)	0.005	0.003, 0.011	0.04
PI-containing ART	−0.045	−0.079, −0.011	0.009

a Model also adjusted for LS BMD, age, gender, ethnicity and BMI.

# Sub article: P170 Bone outcomes with EFV+TDF/FTC versus other TDF-containing antiretroviral regimens among HIV-infected veterans: a US national study

**Published:** 2016-10-23

**Authors:** Joanne LaFleur, Adam Bress, Joel Myers, Lisa Rosenblatt, Jacob Crook, Heather Nyman, Roger Bedimo, Pablo Tebas, Stephen Esker

**Affiliations:** 1 Department of Pharmacotherapy, University of Utah and Salt Lake City VA Healthcare System, Salt Lake City, UT, USA; 2 HIV Medical, Bristol-Myers Squibb, Princeton, NJ, USA; 3 Health Economics and Outcomes Research, Bristol-Myers Squibb, Princeton, NJ, USA; 4 Division of Epidemiology, University of Utah and Salt Lake City VA Healthcare System, Salt Lake City, UT, USA; 5 Infectious Diseases, VA North Texas Healthcare System and University of Texas Southwestern Medical Center, Dallas, TX, USA; 6 Perelman School of Medicine, University of Pennsylvania, Philadelphia, PA, USA

## Abstract

**Introduction**: Tenofovir disoproxil fumarate/emtricitabine (TDF/FTC) is a mainstay backbone in ART for treatment-naïve patients. Although potent and well tolerated, TDF may cause bone toxicity. The magnitude of off-target side effects is proposed to be related to tenofovir (TFV) plasma concentrations, which may be affected by drug–food and drug–drug interactions with concomitant antiretrovirals. We compared bone outcomes (osteoporosis and osteoporotic fractures) with efavirenz (EFV)+TDF/FTC versus non-EFV-based TDF/FTC regimens associated with higher TFV plasma concentrations in treatment-naïve HIV-infected veterans.

**Methods**: This historical cohort study used national Veterans Health Administration (VHA) datasets to identify veterans newly initiating ART in 2003 to 2015. We controlled for selection bias and confounding with inverse-probability treatment weighting. Covariates included baseline demographics, clinical characteristics, HIV laboratory measures, bone measures and other key diagnoses/medication exposures. We used weighted regression models to compare rates of bone adverse events between new users of TDF/FTC with EFV compared to TDF/FTC with non-EFV regimens (i.e. rilpivirine (RPV), elvitegravir/cobicistat (EVG/c) and boosted protease inhibitors (PIs)).

**Results**: Of 33,048 HIV+ veterans, 13,366 received an ART regimen of interest, and 7236 were treatment naïve (4178 EFV and 3058 non-EFV). The median age was 51, 96% were male, and 59% and 30% were Black and Caucasian, respectively. Standardized differences between groups were less than 0.1 for all baseline characteristics following weighting, indicating no significant differences between groups. Crude rates of bone outcomes and adjusted hazard ratios (aHRs) for comparisons with at least five events per treatment group are summarized in Table 1 and Table 2. Unadjusted rates of osteoporosis and osteoporotic fractures were lower in patients who received EFV+TDF/FTC versus each other treatment group. In adjusted analyses, risks were significantly lower for vertebral, upper arm and wrist/forearm fractures for EFV versus all non-EFV regimens combined and versus PIs. For EFV versus EVG/c and RPV, too few events were observed to make stable estimates for the individual outcomes, and no significant differences were observed for the composite bone outcome.

**Conclusions**: EFV+TDF/FTC was associated with a significantly lower risk for bone toxicity and fractures compared to other TDF-containing regimens in the VHA. The third agent in ART regimens can have a significant effect on the risk of bone adverse events associated with TDF.

**Abstract P170–Table 1:** Crude incidence of bone adverse events by treatment group (per 1000 PY)

Outcomes	EFV (n = 4178)	All non-EFV (n = 3059)	EVG/c (n = 234)	RPV (n = 173)	PI (n = 2651)
Any bone outcome	28.4	37.4	49.8	36.0	36.7
Osteoporosis	8.7	12.0	20.6	14.1	11.4
Any osteoporotic fracture	9.9	18.5	13.6	28.6	18.1
Vertebral fracture	2.1	4.2	6.8	7.1	3.9
Hip fracture	3.7	3.1	0.0	14.0	2.6
Upper arm fracture	1.4	3.5	0.0	0.0	3.9
Wrist/forearm fracture	3.3	8.2	6.8	7.1	8.3

**Abstract P170–Table 2:** Risk of bone adverse events by treatment group

Outcomes	EFV vs. non-EFV: aHR (95% CI)	EFV vs. EVG/c: aHR (95% CI)	EFV vs. RPV: aHR (95% CI)	EFV vs. PI: aHR (95% CI)
Any bone outcome	0.78 (0.65–0.94)	0.94 (0.70–1.25)	0.85 (0.66–1.08)	0.78 (0.65–0.94)
Osteoporosis	0.75 (0.55–1.04)	–	–	0.77 (0.55–1.07)
Any osteoporotic fracture	0.57 (0.43–0.76)	–	–	0.57 (0.43–0.77)
Vertebral fracture	0.45 (0.25–0.81)	–	–	0.47 (0.26–0.85)
Hip fracture	1.24 (0.69–2.21)	–	–	1.50 (0.80–2.83)
Upper arm fracture	0.48 (0.23–0.98)	–	–	0.43 (0.21–0.88)
Wrist/forearm fracture	0.47 (0.29–0.75)	–	–	0.45 (0.28–0.72)

–, Fewer than five events.

# Sub article: P171 Prevalence and risk factors of vertebral fractures in patients on antiretroviral treatment

**Published:** 2016-10-23

**Authors:** Ariane Estrella Weiser-Smeke, Jose Antonio Mata-Marín, Marco Adair Liña-Amador, Brenda Espinoza- Armenta, Ana Gil-Avila, Jesús Gaytán-Martínez

**Affiliations:** 1 Medicine School, Anahuac University, Mexico City, Mexico; 2 Infectious Diseases Department, Hospital de Infectología, La Raza National Medical Center, IMSS, Mexico City, Mexico; 3 Epidemiology Department, Hospital de Infectología, La Raza National Medical Center, IMSS, Mexico City, Mexico; 4 Imagenology Department, Hospital de Infectología, La Raza National Medical Center, IMSS, Mexico City, Mexico

## Abstract

**Introduction**: Low bone mineral density (BMD) and fragility fractures are common in patients infected with HIV and antiretrovirals use. In low- and middle-income countries, we don't have DEXA scan to evaluate osteopenia or osteoporosis in HIV-infected patients. The aim of this study was to determine the prevalence and associated risk factors for vertebral fractures in HIV-infected patients in a tertiary care hospital in Mexico.

**Materials and methods**: We conducted a cross-sectional study from August 2014 to February 2015 at the Hospital de Infectología, “La Raza” National Medical Center. Outpatients >40 years who were receiving ART and attending at the clinic were included. Vertebral deformities were detected with lateral spine X-ray using a semi-quantitative morphometric analysis of centrally digitized images: anterior, middle and posterior vertebral heights were measured, and height ratios were calculated. Each vertebral body fractures were defined as mild, moderate and severe on basis of height ratio decreases of 20 to 25%, 26 to 40% and >40%, respectively. The “spine deformity index” (SDI) was calculated by summing the grade of vertebral deformities, according to the semiquantitative method by Genant [1,2]: SDI >1 is indicative of vertebral fracture according to its definition.

**Results**: We included 104 patients, 87% were men. Median age was 49 years old (IQR 42.0–52.75). The most common centers for disease control (CDC) stage was B2 in 40.56 (39%), 47 (45%) had or have had protease inhibitors (PIs) in regimen and 100 (96%) had or were under treatment with nucleosides antiretroviral transcriptase inhibitors. The median years on ART was 6.5 (1.6–9.0). At the moment of the study, 83 (80%) had undetectable HIV-1 RNA viral load, 32 (31%) had a previous fracture, 4 (4%) had hepatitis C virus (HCV) co-infection, and 57 (55%) had a history of corticosteroids treatment. The prevalence of vertebral fractures was 25% (95% CI 17–34%). Risk factors associated to fractures were: female gender OR 0.50 (95% CI 0.10–2.45), p=0.39; HCV co-infection OR 3.1 (95% CI 0.42–23.7), p=0.23; previous corticosteroids use OR 0.51 (95% CI 0.20–1.25), p=0.13; AIDS OR 0.53 (95% CI 0.21–1.34), p=0.18; HIV-1 RNA viral load >100,000 copies/mL OR 1.64 (95% CI 0.29–9.12), p=0.56; and current or previous PIs OR 0.85 (95% CI 0.34–2.09), p=0.73.

**Conclusions**: The prevalence of vertebral fractures was high among HIV-infected patients. A screening for bone disease should be done in patients at risk of fragility fractures. Spine X-ray may be considered in patients at increased risk, irrespective of BMD; that is, in elderly patients majorly in low- and middle-income countries.

## References

[1] Grigoryan M, Guermazi A, Roemer FW, Delmas PD, Genant HK (2003). Recognizing and reporting osteoporotic vertebral fractures. Eur Spine J.

[2] Genant HK, Jergas M, Palermo L, Nevitt M, Valentin RS, Black D (1996). Comparison of semi quantitative visual and quantitative morphometric assessment of prevalent and incident vertebral fractures in osteoporosis *The Study of Osteoporotic Fractures Research Group*. J Bone Miner Res.

# Sub article: P172 Quantifying fracture risk in clinical practice: treatment for osteoporosis should be considered in approximately one out of four HIV+ individuals ≥40 years

**Published:** 2016-10-23

**Authors:** George Siakalis, Konstantinos Protopapas, Polyzois Makras, Ioannis Katsarolis, Dimitra Kavatha, Antonios Papadopoulos, Anastasia Antoniadou

**Affiliations:** 1 4th Department of Internal Medicine, HIV Unit, Attikon University General Hospital, Athens, Greece; 2 Department of Endocrinology, 251 Hellenic Air Force & VA General Hospital, Athens, Greece; 3 Medical Affairs, Gilead Sciences Hellas, Hellinikon, Greece

## Abstract

**Introduction**: Guidelines for bone disease in HIV recommend a screening/risk evaluation process guided by age and classical risk factors, followed by implementation of bone mineral density (BMD) and/or FRAX algorithm accordingly [1–3]. Risk-mitigating interventions should take into account country-specific risk thresholds [2]. This study assessed the fracture risk in a cohort of HIV+ individuals and described factors associated with increased fracture risk.

**Materials and methods**: Cross-sectional fracture risk evaluation was performed by BMD (osteoporosis vs. osteopenia/normal values [3]) and by Greece-specific FRAX algorithm for those ≥40 years [4] through three sequential steps: (A) =HIV not added, (B) =HIV added as a “secondary cause” and (C)=(B)+inclusion of femoral neck T-score value. Greece-specific FRAX intervention thresholds were used (major osteoporotic fracture and hip fracture risk ≥10% and ≥2.5%, respectively for those ≥40 and <75 years) [4]. Analysis was performed for factors associated with increased fracture risk (defined as osteoporosis, prevalent fragility fractures and/or FRAX score).

**Results**: Hundred and forty-three PLWH with available BMD were included: age 45 years, female 26%, stage C 10.5%, BMI 25.5 kg/m^2^, HIV duration 5.4 years, CD4 current/nadir 604/285 cells/µL, 117 on ART, ART duration 5.1 years, VL <50 copies/mL 74%, estimated Glomerular Filtration Rate calculated by CKD-EPI formula (Chronic Kidney Disease Epidemiology Collaboration formula) (eGFR-CKD-EPI) 102.7 mL/min/1.73 m^2^ (median values where applicable). Osteoporosis and osteopenia were diagnosed in 11.9% and 43.4%, respectively. Osteoporosis was associated with ART exposure (naïve 0/26 vs. 17/117, p=0.042) and CD4 nadir (182 vs. 301, p=0.05) (age and years of HIV adjusted). Median FRAX fracture risks for the 98 aged ≥40 years are shown in Table 1.

Increased FRAX score (C) was associated with age (63 vs. 50 years, p = 0.002) and eGFR (91 vs. 99 mL/min, p = 0.031). For those 40 to 49 years, FRAX calculation without BMD missed all four cases for intervention (4/44, 9.1%). For those ≥50 years, BMD measurement identified nine osteoporotic individuals and FRAX with BMD another nine at risk (in total 18/54, 33.3%). Treatment for osteoporosis should have been considered in 22/98 ≥40 years (22.5%) (age ≥50 years, p = 0.009 and menopause, p = 0.015).

**Conclusions**: HIV infection increases significantly the calculated fracture risk. About 22.5% of PLWH ≥40 years were at increased fracture risk independently of ART and labs. If BMD was not available, 13/22 (59%) cases for intervention would have been missed. A combined approach of FRAX with BMD values for everyone ≥40 years (vs. ≥50 years [2]) seems plausible for increasing the yield of fracture risk identification and allowing appropriate intervention strategies.

**Abstract P172–Table 1:** Median 10-year FRAX calculated fracture probability (A) excluding or (B) including HIV infection as a secondary cause of osteoporosis or (C) including HIV infection plus the available femoral neck T-score

10-year FRAX-calculated fracture probability	(A) Median (IQR 1–3)	(B) Median (IQR 1–3)	(C) Median (IQR 1–3)	
Hip fracture	0.2% (0.1–0.7)	0.3% (0.1–1.1)	0.4% (0.1–1.42)	A vs. B, p < 0.001, A vs. C, p < 0.001, B vs. C, p = 0.073
Major osteoporotic fracture	1.9% (1.4–3.6)	2.6% (1.9–4.9)	2.5% (2.5–4.5)	A vs. B, p < 0.001, A vs. C, p < 0.001, B vs. C, p = 0.064
Above intervention thresholds	3/98 (3.1%)	8/98 (8.2%)	14/98 (14.3%)	p for trend 0.007

## References

[1] McGinty T, Mallon P (2016). Protecting bone in long-term HIV positive patients receiving antiretrovirals. Expert Rev Anti Infect Ther.

[2] Brown TT, Hoy J, Borderi M, Guaraldi G, Renjifo B, Vescini F (2015). Recommendations for evaluation and management of bone disease in HIV. Clin Infect Dis.

[3] European AIDS Clinical Society (EACS) EACS guidelines version 8.0. June 2016. http://www.eacsociety.org/guidelines/eacs-guidelines/eacs-guidelines.html.

[4] Makras P, Athanasakis K, Boubouchairopoulou N, Rizou S, Anastasilakis AD, Kyriopoulos J (2015). Cost-effective osteoporosis treatment thresholds in Greece. Osteoporos Int.

# Sub article: P173 Bone deficits in HIV mono-infected and HIV/HCV co-infected individuals belonging to the native Sicilian and migrant/refugee populations, demonstrated by DEXA scanning

**Published:** 2016-10-23

**Authors:** Giuseppe Lo Re, Paola Di Carlo, Angelo Gambino, Nicola Serra, Claudia Colomba, Antonio Lo Casto, Giuseppe Guglielmi, Antonio Cascio, Roberto La Galla

**Affiliations:** 1 Department of Biopathology and Biotechnologies (DiBiMed), Policlinico Universital Hospital Paolo Giaccone, Palermo, Italy; 2 Health Promotion and Child Health, Policlinico Universital Hospital of Palermo, Palermo, Italy; 3 Department of Radiology, University of Naples, Naples, Italy; 4 University of Foggia, Department of Radiology, Foggia, Italy

## Abstract

**Introduction**: Migrants and refugees from countries with a high incidence of HIV and other co-infections such as hepatitis C virus (HCV) and/or hepatitis B virus represent an emerging challenge for the healthcare system. We used dual-energy X-ray absorptiometry (DEXA) to detect osteoporosis (OP) in HIV/HCV co-infected and HIV mono-infected groups in order to determine OP prevalence by age, gender and geographic origin.

**Materials and methods**: We conducted a cross-sectional study on 211 HIV mono-infected and 81 HIV/HCV co-infected patients attending the AIDS Centre of the Infectious Diseases Department of Palermo University Hospital, Italy, between January 2010 and May 2015. All patients underwent a DEXA scan.

**Results**: HIV mono-infection was prevalent among non-Europen Union (nEU) women. HIV/HCV co-infection was higher in nEU males than females. Comparing the groups split according to age, it turned out that there was a significant difference among them with respect to HIV in nEU and European Union (EU) patients (p<0.000). These differences, identified by residual analysis, were greater in nEU patients aged between 28 and 47. Stratifying by age, prevalence of OP was significantly higher in patients ≥49 years with HIV/HCV co-infection. OP prevalence was higher among HIV/HCV co-infected EU and nEU female patients than among those with HIV mono-infection (p=0.011). Comparing the groups split according to age and geographical origin, we found that there was a significant difference among them with respect to HIV/HCV and OP in nEU and EU patients (respectively, p=0.017 and p<0.000). These differences, identified by residual analysis, showed a higher prevalence of OP among nEU patients aged 28 to 47 years (p<0.000).

**Conclusions**: Our study provides evidence for the usefulness of early screening for OP in HIV/HCV co-infected patients, especially in nEU women. Significant and sustained improvements in mortality and morbidity, and control of the current HCV epidemic in HIV-infected subgroups such as migrants could then become a feasible goal.

# Sub article: P174 Cardiovascular events – a thing of the past? A real-life assessment in an inner-city Toronto clinic

**Published:** 2016-10-23

**Authors:** Fred Crouzat, Brenda Varriano, Ina Sandler, Graham Smith, Samantha Steinberg, Colin Kovacs, David Fletcher, David Knox, Barry Merkley, Benny Chang, David Tilley, Megan Ascai, Malika Sharma, Mona Loutfy

**Affiliations:** 1 Infectious Disease, Maple Leaf Medical Clinic, Toronto, Canada; 2 Institute of Medical Sciences, University of Toronto, Toronto, Canada; 3 Maple Leaf Medical Clinic, Toronto, Canada; 4 Faculty of Science, University of Guelph, Guelph, Canada

## Abstract

**Introduction**: Following co-morbid cardiovascular events (CVE) amongst HIV-positive patients is essential. Abacavir's impact on CVE is unclear and challenges clinicians. We characterized CVE at the largest Canadian HIV clinic stratified three-fold: 1) patients being antiretroviral-naïve or -experienced; 2) taking abacavir or tenofovir-disoproxil-fumerate (TDF) or switching between the two and 3) time-era (before and after 2009).

**Materials and methods**: This is a retrospective study using electronic medical records (EMRs) of all HIV-positive patients treated at Maple Leaf Medical Clinic, who started a combination ART (cART) regimen (3 to 7 drugs) with abacavir or TDF (one-switch between the two permitted). Patients were excluded if a pre-cART CVE or a second-switch between abacavir and TDF occurred. Patients were assessed as those starting cART (antiretroviral-naïve) and overall (antiretroviral-naïve and – experienced). The outcome was CVE (cardiovascular (CAE) or cerebralvascular (CEE)). There were four exposures-of-interest: always-abacavir-, always-TDF-, first-abacavir-switched-to-TDF- and first-TDF-switched-to-abacavir cART regimens. The analysis was stratified into being on cART: (1) before or (2) after 1 January 2009. Evaluation started at cART-initiation and ended at: CVE date, second switch between abacavir and TDF or last data-cut date (16 July 2015). Descriptive statistics and bivariate analyses were done using standard statistical methods. Multivariable Cox regression was carried out with time-to-CVE as the outcome and time-on-abacavir or -TDF as the exposure-of-interest. Confounders corrected for included: Framingham-score and time-on-PI.

**Results**: Of 2851 patients, 1439 were antiretroviral-naïve. Of the total, median age=40 (IQR=34–46), 92% male, 65% Caucasian, median HIV duration=5.2 years (IQR=1.5–10.6), baseline log10VL=4.02 (IQR=1.70–4.91) and CD4-count=330cells/uL (IQR=210–500); 658-on-an-abacavir-regimen, 1186-on-a-TDF-regimen, 736 switched from abacavir-to-TDF and 271-switched-from-TDF-to-abacavir. Seventy-six CVE occurred [15-in antiretroviral-naïve and 61-in antiretroviral-experienced (p<0.0001)]. 61/76 of the events were CAE and 15/76 were CEE (p<0.0001). 69/76 CVE were before 2009 and 8/76 after (p<0.0001). 40/76 CVE were on-abacavir, 15/76 on-TDF, 19/76-switched from abacavir to TDF and 2/76-switched from TDF to abacavir (p<0.0001). 21/21 switches occurred prior to 2009 and 38/40 on-abacavir remained on it after 2009. The multivariable Cox regression revealed that Framingham score and time-on-a-PI increased the CVE risk [aHR=1.10-per-1-point (95%CI=1.04–1.18) and aHR=1.11-per-year (95%CI=0.94–1.31), respectively]; time-on-abacavir (aHR=0.17-per-year (95%CI=0.09–0.34) and time-on-TDF (aHR=0.13-per-year (95%CI=0.06–0.25), both decreased the chance of a CVE.

**Conclusions**: 91% CVE occurred before 2009 and 80% in antiretroviral-experienced patients. Abacavir was associated with CVE before 2009 and in our overall-population and in univariate but not multivariable analyses. Our multivariable model showed that the Framingham score predicted CVE but the longer duration on TDF or abacavir decreased CVE risk. Abacavir's impact on CVE is still unclear but this analysis is helpful to understand CVE in our clinic.

# Sub article: P175 Risk of cardiovascular disease events with atazanavir-based antiretroviral treatment regimens among HIV-infected veterans: a US national study

**Published:** 2016-10-23

**Authors:** Joanne LaFleur, Adam Bress, Lisa Rosenblatt, Jacob Crook, Paul Sax, Joel Myers, Corey Ritchings

**Affiliations:** 1 Department of Pharmacotherapy, University of Utah and Salt Lake City VA Health Care System, Salt Lake City, UT, USA; 2 Department of Population Health Sciences, University of Utah and Salt Lake City VA Health Care System, Salt Lake City, UT, USA; 3 Health Economics and Outcomes Research, Bristol-Myers Squibb, Princeton, NJ, USA; 4 Division of Epidemiology, University of Utah and Salt Lake City VA Health Care System, Salt Lake City, UT, USA; 5 Department of Infectious Diseases, Brigham and Women's Hospital and Harvard Medical School, Boston, MA, USA; 6 HIV Medical, Bristol-Myers Squibb, Princeton, NJ, USA

## Abstract

**Introduction**: Cardiovascular disease (CVD) is a leading cause of death in HIV-infected patients. Atazanavir (ATV) has been associated with slower progression of atherosclerosis in several studies, but there is limited information on the relative impact of ATV on the risk of CVD events compared to other regimens. We examined CVD events with ATV-based regimens versus those based on other protease inhibitors (PIs), non-nucleoside reverse transcriptase inhibitors (NNRTIs) and integrase inhibitors (INSTIs) in treatment-naïve HIV-infected veterans.

**Methods**: This historical cohort study used national United States Veterans Health Administration (VHA) datasets to identify HIV+ veterans newly initiating ART in 2003 to 2015. We controlled for selection bias and confounding by indication with inverse probability of treatment weights. Covariates included baseline demographics, HIV laboratory measures, comorbidities and key concomitant medications. We used Cox proportional hazards regression models to calculate hazard ratios for incident CVD events (myocardial infarction (MI) and stroke) associated with new users of ATV compared to each non-ATV regimen.

**Results**: Of 33,048 HIV+ veterans, 21,289 received an ART regimen of interest during the study period, and 10,385 were treatment naïve including 1530 with ATV and 2459, 5785 and 611 with other PIs, NNRTIs and INSTIs, respectively. The mean (standard deviation) age was 50.0 (10.1), 93% were male, and 56% and 30% were Black and Caucasian, respectively. After weighting, standardized mean differences between groups were less than 0.1 for all baseline characteristics, indicating no significant differences between groups. Risks were significantly lower with ATV compared to other PIs for MI and for ATV compared to INSTI-based regimens for the MI/stroke composite outcome (Table 1).

**Conclusions**: In the VHA, ATV-based regimens were generally associated with a lower risk for CVD events compared to other antiretrovirals. Further research to elucidate the mechanism for a potential reduced risk of CVD events with atazanavir is warranted.

**Abstract P175–Table 1:** Risk of CVD events with ATV versus non-ATV-based regimens

Outcome	ATV crude incidence (per 1000 patient-years)	ATV vs. other PIs: HR (95% CI)	ATV vs. NNRTIs: HR (95% CI)	ATV vs. INSTIs: HR (95% CI)
MI	5.16	0.46 (0.26–0.81)*	0.72 (0.48–1.09)	0.71 (0.31–1.61)
Overall stroke	18.31	0.89 (0.66–1.22)	1.01 (0.81–1.27)	0.71 (0.47–1.07)
Ischemic stroke	17.65	0.90 (0.66–1.25)	1.03 (0.81–1.29)	0.74 (0.48–1.12)
Haemorrhagic stroke	0.64	0.50 (0.18–1.42)	0.50 (0.16–1.52)	0.18 (0.02–1.36)
MI/stroke	23.04	0.80 (0.61–1.05)	0.94 (0.77–1.15)	0.69 (0.47–1.00)*
MI/stroke/death	38.83	0.90 (0.73–1.12)	0.98 (0.84–1.15)	0.81 (0.60–1.10)
All-cause death†	16.02	1.01 (0.73–1.39)	0.91 (0.72–1.15)	1.11 (0.66–1.90)

* *P*<0.05.

† Based on VA vital status files.

# Sub article: P176 A reappraisal with meta-analysis of abacavir use and cardiovascular disease events

**Published:** 2016-10-23

**Authors:** Mario Cruciani, Saverio Parisi

**Affiliations:** 1 Infectious Diseases Service, San Bonifacio Hospital, Verona, Italy; 2 Department of Histology, Microbiology and Biotechnology, University of Padua, Padua, Italy

## Abstract

**Introduction**: Nucleoside reverse transcriptase inhibitors (NRTIs) continue to be a cornerstone of antiretroviral therapy (ART), and currently recommended regimens include the NRTIs combination of abacavir (ABC)/3TC or tenofovir (TDF)/emtricitabine. Whether the exposure to ABC contributes to cardiovascular risk remains unclear. Results from several cohort studies have identified an increase cardiovascular risk after ABC exposure, but the results of other cohort studies, of randomized clinical trials (RCTs) and of meta-analyses of RCTs did not find this association. In this study we have updated the results of a previous meta-analysis [1], focusing on trials with a head-to-head comparison of ABC and TDF.

**Methods**: A systematic review and meta-analysis was performed using Cochrane methodologies. Data extracted included: myocardial infarction (MI), any cardiovascular events and overall mortality. We used a conventional Mantel-Haenszel method, with risk ratio and 95% confidence intervals (CIs).

**Results**: We obtained data from 11 RCTs conducted from 2006 to 2015, comparing ART with ABC to TDF, both in combination with the same third agent. Risk of bias assessment showed an overall good methodological quality of included studies; however, since overall cardiovascular event and MI were not predefined outcomes in many of the included studies, we judged the quality of the evidence “moderate” for these outcomes. Data on overall cardiovascular events were available from nine RCTs (4847 patients), data on MI from nine RCTs (5130 patients) and data on mortality from six RCTs (3646 patients). Compared to the TDF, ABC use did not increase the occurrence of overall cardiovascular events (RR 1.32; 95% CI 0.67–2.59 (Figure 1)), the occurrence of MI (RR 0.74; 95% CI 0.26–2.12) and the overall mortality (RR 1.05; 95% CI 0.38–2.91).

**Conclusions**: Our meta-analysis of RCTs did not show an increase in the occurrence of overall cardiovascular events, MI and overall mortality in ABC compared to TDF recipients.

**Abstract P176–Figure 1:** Forest plot: outcome: overall cardiovascular events. Comparison: abacavir versus tenofovir.

## References

[1] Cruciani M, Zanichelli V, Serpelloni G, Bosco O, Malena M, Mazzi R (2011). Abacavir use and cardiovascular disease events: a meta-analysis of published and unpublished data. AIDS.

# Sub article: P177 Awareness and management of elevated blood pressure among HIV-infected adults receiving antiretroviral therapy in urban Zambia

**Published:** 2016-10-23

**Authors:** Sophie Bauer, Zude Zyambo, Mwanza ma Mwanza, Roma Chilengi, Mary-Ann Davies, Matthias Egger, Hansjakob Furrer, Michael Vinikoor, Gilles Wandeler

**Affiliations:** 1 Department of Infectious Diseases, University Hospital Bern, Bern, Switzerland; 2 Hepatitis Research Group, Center for Infectious Disease Research in Zambia (CIDRZ), Lusaka, Zambia; 3 Centre for Infectious Disease Epi and Research, University of Cape Town, Cape Town, South Africa; 4 Institute of Social and Preventive Medicine, University of Bern, Bern, Switzerland; 5 Department of Medicine, University of Alabama at Birmingham, Birmingham, AL, USA

## Abstract

**Introduction**: In recent years, effective ART resulted in increasing survival of HIV-infected individuals and in the emergence of comorbid non-communicable diseases (NCDs) as a global burden. We characterized the prevalence, awareness and management of elevated blood pressure (BP) among individuals engaged in ART programmes in urban Zambia to provide a foundation for future public health strategies.

**Materials and methods**: We analyzed recorded data about cardiovascular (CV) risk factors (elevated BP, overweight (body mass index ≥25 kg/m^2^), smoking, hazardous drinking) from all HIV-infected adults enroled in a prospective cohort in Lusaka, Zambia. We used Chi-squared and Mann-Whitney tests to evaluate associations between individual patient characteristics and elevated BP, defined as one or more values of systolic BP ≥140 mmHg or diastolic BP ≥90 mmHg. In patients with elevated BP, we explored patient awareness and management of elevated BP as well as history of CV events (diabetes, stroke and heart condition) and family history of hypertension using mobile phone follow-ups.

**Results**: Among 895 individuals, 92 (10.3%) individuals had elevated BP and 57 (6.4%) had at least two elevated measurements. Patients with elevated BP were older (median age 37 vs. 34 years, p<0.001), more likely to be men (61% vs. 46%, p=0.01) and to be overweight (26% vs. 12%, p<0.001) compared to other participants. Pre-ART CD4 cell count as well as proportion of patients with hepatitis B infection and alcohol/tobacco consumption were similar in both groups. Among the group with elevated BP, 66 (72%) were contactable telephonically and 35 (53%) of them were aware of their condition (Figure 1). For those aware of their condition, the information about elevated BP had been communicated primarily by a nurse (60%) and at ART clinics at scheduled study visits (63%). Fourteen (21%) reported having ever taken BP-lowering drugs; however, only one (3%) was currently taking a BP-lowering drug prescribed following a CV event in a university teaching hospital. Of the 66 patients contacted, 24 (36%) had ≥2 related CV risk factors, and nine (14%) reported prior history of CV events.

**Conclusions**: Despite routine screening for arterial hypertension, awareness of elevated BP is low and prescription of BP-lowering drugs is rare even in individuals with reported CV events, and regular follow-ups. This suggests that integrated NCD screening and management in a population linked to care through ART programmes has not yet been realized.

**Abstract P177–Figure 1:** Level of awareness and use of blood pressure-lowering drugs among contacted patients with elevated blood pressure (N = 66).

# Sub article: P178 Reduction of immune activation in HIV-infected patients after introducing pitavastatin

**Published:** 2016-10-23

**Authors:** Enrique Bernal Morell, Monica Martinez, Joaquin Bravo, Francisco Vera, Onofre Martinez, Carlos Galera, Angeles Muñoz, Irene Marín, Antonia Alcaraz, Alfredo Cano, Salvador Valero, Jose Miguel Gomez Verdu, Ana Torres, Alfredo Minguela

**Affiliations:** 1 Infectious Disease Unit, Reina Sofia Hospital, Murcia, Spain; 2 Infectious Disease Unit, Morales Meseguer Hospital, Murcia, Spain; 3 Infectious Disease Unit, Santa Lucia Hospital, Murcia, Spain; 4 Infectious Disease Unit, Virgen de la Arrixaca Hospital, Murcia, Spain; 5 Cardiology Unit, Reina Sofia Hospital, Murcia, Spain; 6 Immunology Service, Virgen de la Arrixaca Hospital, Murcia, Spain

## Abstract

**Introduction**: Pitavastatin is a new statin highly effective in lowering cholesterol, but its effect on the immune system in HIV-infected patients is unknown. The aim of this study was to evaluate the effects of pitavastatin on immune activation in HIV patients receiving antiretroviral treatment.

**Materials and methods**: Clinical trial was carried out to compare in HIV-infected patients with hypercholesterolemia on antiretroviral treatment, diet+pitavastatin versus diet at 24 weeks. Student's t-test for paired data was used to compare changes of the parameters analyzed.

**Results**: Fifty-one patients were included, 30 started with pitavastatin and 21 with diet. Three patients were withdrawn in the pitavastatin group and five in the diet group. There were 21 males, 51±11 years receiving treatment with NNRTIs (14 patients), PI (11 patients) and integrase inhibitors (one patient) in pitavastatin group. There were 14 males, 47±9 years receiving treatment with NNRTIs (seven patients), PI (seven patients) and integrase inhibitors (two patients). After 24 weeks, there was a significant decrease in total cholesterol (237.6±43.24 mg/dL to 191.6±26.7 mg/dL; p<0.001), cholesterol-LDL (158.5±35.8 mg/dL to 114.79±27.4 mg/dL; p<0.001), triglycerides (205.89±103.9 mg/dL to 169.05±88.7 mg/dL; p=0.027), CD38-mean fluorescence intensity (MFI) in NKs (9007.05±3692.2 to 7640.53±4720.66; p=0.034) and CD38-MFI in CD4+CD28+ T lymphocytes (1415.67±509.87 to 1253.39±592.39; p=0.049) and increased of Apo A1 (127.33±6.42 mg/dL to 150.67±9.71 mg/dL; p=0.007) in pitavastatin group. However the levels of total cholesterol, cholesterol-LDL and Apo A1 were not affected in the diet group. There was a significant increase of CD38-MFI in CD4+CD28- T lymphocytes (4230±1021.2 to 3106.71±1171.3; p=0.012) in diet group. Pitavastatin was well tolerated and there were no serious side effects.

**Conclusions**: Pitavastatin is safe and significantly reduces the levels of total cholesterol and LDL in HIV-infec ted patients on antiretroviral therapy. Additionally, pitavastatin decreases immune activation and therefore might reduce non-AIDS events.

# Sub article: P179 Extensive vascular phenotyping – for the detection of subclinical atheromatosis, arteriosclerosis and arterial hypertrophy – and its association with international cardiovascular prediction scores in HIV-infected individuals: a single-centre study

**Published:** 2016-10-23

**Authors:** Mina Psichogiou, Katerina Arida, Martha Papadopoulou, Theodoros Papaioannou, Argyro Lazarini, George Konstantonis, Panagiotis Petrikkos, George Daikos, George Kitas, Petros Sfikakis, Athanasios Protogerou

**Affiliations:** 1 1st Department of Internal Medicine, Laikon Hospital, Medical School, National and Kapodistrian University of Athens, Athens, Greece; 2 1st Department of Propaedeutic Internal Medicine, Laikon Hospital, Medical School, National and Kapodistrian University of Athens, Athens, Greece; 3 Cardiology, Ippokration Hospital, Athens, Greece; 4 Internal Medicine, 251 Hellenic Airforce VA General Hospital, Athens, Greece; 5 Rheumatology, University of Manchester, Dudley Group NHS Foundation Trust, Manchester, UK; 6 Cardiovascular Prevention and Research Unit, Laikon Hospital, Medical School, National and Kapodistrian University of Athens, Athens, Greece

## Abstract

**Introduction**: Accelerated subclinical arterial damage has been widely identified in HIV-infected individuals and has arterial site-specific predilection [1]. The increased incidence of cardiovascular disease (CVD) in HIV-infected individuals is mediated [2] and predicted [3] by the presence of subclinical arterial damage. Various CVD risk prediction scores – derived mostly from general populations – are used for CVD prevention in HIV-infected individuals. All scores underestimate the presence of subclinical arterial damage in these populations and exhibit low agreement [4]. No mortality-based comparison studies and no consensus exist regarding the best available CVD risk prediction score for HIV-infected individuals. We performed (i) extensive vascular phenotyping to detect subclinical arterial damage of all types of arterial pathology at different arterial beds and (ii) tested/compared the association of the four most widely applied CVD risk prediction scores with the presence of subclinical arterial pathology.

**Materials and methods**: Consecutive HIV-infected individuals, free of CVD, underwent by the same technician vascular tests for the detection of (i) atheromatosis (common/internal carotid and femoral bed plaque, ankle-brachial index), (ii) arteriosclerosis (common carotid elasticity, aortic pulse wave velocity) and (iii) arterial hypertrophy (common carotid intimal-medial thickness). The European Society of Cardiology (ESC), Framingham (FR), American Heart Association/American College of Cardiology (AHA/ACC) and the Data collection on Adverse effects of Anti-HIV Drugs study (DAD) scores were assessed. Logistic regression and ROC analysis and c-statistics were applied.

**Results**: Out of 134 participants (92.5% males; age 40.8±1.5 years), 76.1% had at least one type of subclinical arterial pathology, 60% arteriosclerosis, 35.3% arterial hypertrophy, 31.6% atheromatosis. The ESC, FR and DAD scores presented statistically significant and consistent association with combined as well as with almost each type of arterial pathology in separate. On the contrary, the AHA/ACC score failed to associate with any type of arterial pathology. The ESC, FR and DAD scores, but not the AHA/ACC score, detected the presence of combined arterial pathology. The FR-10 year-CHD score had higher area under the curve than all other scores (AUC: 0.756, p<0.001; c-statistics p<0.05 versus all other scores).

**Conclusions**: This single-center, single-operator vascular phenotyping study in HIV-infected individuals suggests that: (i) extensive subclinical arterial pathology of all types is present in this population; (ii) the ESC, DAD and FR scores, but not the AHA/ACC score, associate with and detect all types of subclinical arterial pathology.

## References

[1] http://dx.doi.org/10.1371/journal.pone.0132307.

[2] http://dx.doi.org/10.7326/M14-0378.

[3] http://dx.doi.org/10.1016/j.atherosclerosis.2010.11.013.

[4] http://dx.doi.org/10.1177/2047487312452964.

# Sub article: P180 Initiation of antiretroviral therapy restores endothelial cell function in HIV-infected individuals

**Published:** 2016-10-23

**Authors:** Patricia Echeverría, Elisabet Gómez-Mora, Santiago Roura, Anna Bonjoch, Jordi Puig, Nuria Pérez-Alvarez, Antonio Bayés-Genis, Bonaventura Clotet Sala, Juliá Blanco, Eugenia Negredo

**Affiliations:** 1 HIV Unit, Foundation Lluita contra la SIDA, Hospital Universitari Germans Trias i Pujol, Badalona, Spain; 2 HIV, AIDS Research Institute-IRSICAIXA, Hospital Universitari Germans Trias i Pujol, Badalona, Spain; 3 Cardiology, ICREC Group, Institut Germans Trias i Pujol (IGTP), Badalona, Spain; 4 Centro de Medicina Regenerativa de Barcelona, Barcelona, Spain; 5 Statistics and Operations Research, Technical University of Catalunya, Barcelona, Spain; 6 Servei de Cardiologia, Hospital Universitari Germans Trias i Pujol, Badalona, Spain; 7 Departament de Medicina, Universitat Autònoma de Barcelona, Barcelona, Spain; 8 Universitat de Vic – Universidad Central de Catalunya UVIC-UCC, Vic, Spain

## Abstract

**Introduction**: Initiation of antiretroviral therapy reduces hyperactivity of the immune system; however, the effect on endothelial cell function seems to be contradictory [1].

**Design**: We performed a 24-week prospective, case-control and comparative pilot study of antiretroviral-naïve HIV-infected patients who started a darunavir (DRV)- or rilpivirine (RPV)-based regimen and age/sex-matched non-HIV-infected volunteers to compare changes at week 24 from baseline in levels of circulating endothelial cells (CECs), endothelial progenitor cells (EPCs) and circulating angiogenic cells (CACs), as well as changes in immune activation markers and their association with virologic, immunologic and clinical parameters.

**Results**: The study population comprised 48 participants (24 HIV-infected patients and 24 non-infected volunteers). Both HIV groups completely suppressed viremia and had significantly increased CD4 T-cell counts after 24 weeks of treatment. HIV-infected patients had higher levels of activation markers than the control group in CD8 T-cell populations at baseline; these decreased after 24 weeks of treatment but without reaching the levels of the control group. No statistical differences in immune activation were seen between the DRV and RPV groups (Figure 1.). Levels of CECs were higher and levels of EPCs and CACs were lower in HIV-infected patients than in the control group, although all these parameters were similar between the DRV group and the control group but not the RPV group at week 24 (Figure 2). An unfavourable association was observed between RPV, age and increased number of CECs.

**Conclusions**: Restoration of circulating levels of EPCs and CECs in DRV-treated patients was greater than in those treated with RPV, suggesting ongoing endothelial repair mechanisms.

**Abstract P180–Figure 1:** Immunologic responses to initiation of cART. **Immune activation levels measured by the frequency of CD38 + HLA-DR+ CD8 T (left panel). Levels of the exhaustion marker PD-1 in the CD8 T (right panel)**.

**Abstract P180–Figure 2:** Endothelial response to cART initiation. **Frequencies of CEC (right), EPC (middle) and CAC (right) are shown at baseline and the end of the study (week 24). Data uninfected volunteers represented by empty dots. Light grey dots correspond to RPV and dark grey to DRV group**.

## References

[1] Torriani FJ, Komarow L, Parker RA, Cotter BR, Currier JS, Dubé MP (2008). Endothelial function in human immunodeficiency virus-infected antiretroviral-naïve subjects before and after starting potent antiretroviral therapy: the ACTG (AIDS Clinical Trials Group) Study 5152s. J Am Coll Cardiol.

# Sub article: P181 Cardiovascular risk factors and use of lipid-lowering therapy in a cohort of HIV-positive patients with high cardiovascular risk

**Published:** 2016-10-23

**Authors:** Johanna Denman, Kaveh Manavi

**Affiliations:** 1 Genitourinary Medicine, University Hospital Birmingham NHS Trust, Birmingham, UK

## Abstract

**Introduction**: Patients with HIV have high cardiovascular risk [1]. Cardiovascular disease (CVD) is a common cause of death amongst people with HIV [2]. We aimed to review contributors to cardiovascular risk in patients with HIV at high risk of CVD and the management of lipids with reference to NICE guidelines 2008 [3].

**Materials and methods**: Patients with estimated 10-year CVD risk greater than 20% (QRISK2 score) in 2014 were selected from the clinic database. Data recorded included demographic details, CD4 count, viral load, antiretroviral treatment, contributors to QRISK2 score and lipid management.

**Results**: We identified 39 patients with QRISK2 greater than 20%. The viral load was undetectable (<40 copies/mL) in 89% of these patients indicating well-controlled HIV. Median CD4 count was 612 cells/mm^3^, four patients had CD4 less than 350 cells/mm^3^. Modifiable risks were identified in 86.5% of patients; 40.5% were current smokers, 43.2% had a systolic blood pressure >140 mmHg or diastolic >90 mmHg, 54.1% had non-HDL cholesterol greater than 3 mmol/L, 32.4% had a BMI >30 kg/m^2^. NICE guidelines 2008 (CG67) recommended statin be offered if 10-year risk >20% [3]. Only 26 out of 37 patients (70.3%) were on statin prior to risk assessment (included two patients on fenofibrate due to intolerance of statin). A statin was subsequently commenced in 4 of the remaining 11 patients (36.4%). None achieved the recently adopted target of 40% reduction in non-HDL cholesterol after 8 months [4].

**Conclusions**: Modifiable CVD factors (smoking, hypertension, lipids and weight) contributed to a significant number of patients with high CVD in our cohort. Lifestyle changes should be promoted and supported better in HIV clinics. This would include better communication with the GPs. Our clinic is developing a standard letter for GPs requesting assistance with addressing modifiable risk factors, to be sent when a patient with high cardiovascular risk is identified. Once statin is commenced lipids should be monitored and reviewed to ensure target reduction in non-HDL cholesterol is achieved.

## References

[1] Paisible A, Chang C, So-Armah K, Butt AA, Leaf DA, Budoff M (2015). HIV infection, cardiovascular disease risk factor profile, and risk for acute myocardial infarction. J Acquir Immune Defic Syndr.

[2] British HIV Association (BHIVA) National HIV mortality audit.

[3] National Institute for Health and Care Excellence (NICE) Lipid modification: cardiovascular risk assessment and the modification of blood lipids for the primary and secondary prevention of cardiovascular disease.

[4] National Institute for Health and Care Excellence (NICE) Cardiovascular disease: risk assessment and reduction, including lipid modification.

# Sub article: P182 Prevalence of cardiovascular diseases in West African HIV-infected adults receiving HAART

**Published:** 2016-10-23

**Authors:** Nogbou Frédéric Ello, Chrysosthome Mossou, Patrick Coffie, Affoué Gisèle Kouakou, Coulibaly Iklo, Jean-Baptiste Anzoua-Kacou, Serge Paul Eholie

**Affiliations:** 1 Dermatology-Infectious Diseases, Treichville University Teaching Hospital, Abidjan, Ivory Coast; 2 Thoracic and Vascular Treichville University Teaching Hospital, Abidjan, Ivory Coast

## Abstract

**Introduction**: Non-communicable diseases (NCDs) are emerging as an important concern related to the improvement of life expectancy of HIV-infected patients, to antiretroviral drug toxicity and also to the chronic inflammation associated with persistent viral replication [1]. Few studies have been conducted in low-income countries, particularly in West Africa [2,3]. We are interested in severe morbidity of cardiovascular diseases (CVD) in HIV-positive patients on antiretroviral therapy. Therefore, we assessed the prevalence of severe CVD in HIV-infected patients followed up in the Tropical and Infectious Diseases Unit (TIDU) and looked for factors associated with them.

**Materials and methods**: A cross-sectional study was conducted at the TIDU in Abidjan, from April to July 2015, in patients aged over 18 years, HIV positive and on antiretroviral therapy for at least 12 months. Data were collected using a structured questionnaire. Clinical assessment, laboratory tests, transthoracic echocardiography and electrocardiogram were performed for all the patients. All the subjects underwent ultrasonography of the carotid and femoral vessels to evaluate intima-media thickness. The primary endpoint was proportion of patients with severe CVD. Analysis of factors associated was conducted by logistic regression.

**Results**: Two hundred and seventy eight patients (mean age 46 years, female 74.5%) were included. The proportion of patients with clinical stage C of the CDC classification was 119 (42.8%) and 229 (82.4%) were with virologic suppression (undetectable viral load). The prevalence of severe CVD was 7.6% [95% CI 4.74–11.32]; the majority was represented by pulmonary arterial hypertension (5%). In multivariate analysis, running time below 30 min, high blood pressure, high ALT rate, high glycaemia rate and low nadir CD4 count were significantly associated with the prevalence of CVD.

**Conclusion**: The prevalence of life-threatening CVD was significant. Therefore, standardized screening and risk reduction interventions should be routinely undertaken among HIV-infected patients on HAART [4] and discussion of the current approach to primary prevention of CVD in HIV-positive patients is crucial.

## References

[1] Dubé MP, Lipshultz SE, Fichtenbaum CJ, Greenberg R, Schecter AD, Fisher SD (2008). Effects of HIV infection and antiretroviral therapy on the heart and vasculature. Circulation.

[2] Eholié S, Lacombe K, Krain A, Diallo Z, Ouiminga M, Campa P (2015). Metabolic disorders and cardiovascular risk in treatment-naïve HIV-infected patients of sub-Saharan origin starting antiretrovirals: impact of westernized lifestyle. AIDS Res Hum Retroviruses.

[3] Olusegun-Joseph D, Ajuluchukwu JN, Okany C, Mbakwem A, Oke D, Okubadejo N (2012). Echocardiographic patterns in treatment-naïve HIV-positive patients in Lagos, south-west Nigeria. Cardiovasc J Afr.

[4] TEMPRANO ANRS 12136 Study Group A trial of early antiretrovirals and isoniazid preventive therapy in Africa. N Engl J Med.

# Sub article: P183 Premature cerebral atherosclerosis in HIV-infected individuals in Lisbon: a carotid ultrasound and transcranial Doppler study

**Published:** 2016-10-23

**Authors:** António Pais de Lacerda, Marta Melo, Pedro Coelho, Paulo Baptista, Fátima Soares, Luis Caldeira, Emília Valadas, Vitor Oliveira

**Affiliations:** 1 Hospital de Santa Maria, Centro Hospitalar Lisboa Norte / Faculdade de Medicina da Universidade de Lisboa, Serviço de Medicina Intensiva, Lisboa, Portugal; 2 Faculdade de Medicina da Universidade de Lisboa, Clínica Univ de D. Infecciosas e Parasitárias, Lisboa, Portugal; 3 Nova Information Management School, Universidade Nova de Lisboa, Lisboa, Portugal; 4 Laboratório de Hemodinâmica Cerebral, Hospital de Santa Maria, Centro Hospitalar Lisboa Norte, Lisboa, Portugal; 5 Clínica Univ de D. Infecciosas e Parasitárias, Hospital de Santa Maria, Centro Hospitalar Lisboa Norte, Lisboa, Portugal

## Abstract

**Introduction**: Premature atherosclerosis has been linked to HIV infection and/or to antiretroviral treatment [1]. Carotid intima-media thickness (CIMT) and pulsatility index (PI) accessed by carotid duplex ultrasonography (CDU) and transcranial Doppler (TCD) may be useful markers [2–4].

**Materials and methods**: Carotid and cerebral circulation were evaluated by CDU and TCD in 40 HIV-infected Caucasian men (mean age 49.4±5.9 years). CD4+ T-cell current and nadir counts, current and zenith viral load and HIV drug classes with duration of ART were registered; cardiovascular risk scores were also assessed. Multivariate regression analysis and Pearson's correlation coefficient were used.

**Results**: All men received ART and presented mean CD4+ count of 817±369 cells/mm^3^ (mean nadir 242.8±158.2 cells/mm^3^) at the time of the study, 95% had non-detectable viral load (mean zenith 381,416±858,881 copies/mL), 35% had history of high blood pressure, 35% dyslipidaemia, 7.5% diabetes and 80% tobacco consumption. Cardiovascular risk by Framingham Risk Score, SCORE and ASCVD score were low at 10 years and lifetime. More than half (67.5%) had increased CIMT (mean 0.92±0.13 mm), but none presented increased PI. No correlation was found between duration of infection, ART classes or cardiovascular risk scores with CDU or TCD data. However, a significantly positive association between a CD4+ nadir count <400 cells/mm^3^ and an increase of 0.12 in PI was confirmed by regression analysis where CD4 categories showed significant effect over PI (p=0.04).

**Conclusions**: In this series, HIV infection showed an association with premature cerebral atherosclerosis, even at low cardiovascular risk scores, and independently of therapies employed and treatment time. PI may be an early marker of atherosclerosis in HIV-infected people with CD4+ nadir <400 cells/mm^3^. There is a need to evaluate these parameters in a larger number of HIV-infected people, including elite controllers.

## References

[1] Calza L, Manfredi R, Pocaterra D, Chiodo F (2008). Risk of premature atherosclerosis and ischemic heart disease associated with HIV infection and antiretroviral therapy. J Infect.

[2] Hsue P, Lo J, Franklin A, Bolger A, Martin J, Deeks S (2004). Progression of atherosclerosis as assessed by carotid intima-media thickness in patients with HIV infection. Circulation.

[3] Longenecker C, Hoit B (2012). Imaging atherosclerosis in HIV: carotid intima-media thickness and beyond. Transl Res.

[4] Stein J, Currier J, Hsue P (2014). Arterial disease in patients with human immunodeficiency virus infection: what has imaging taught us?. JACC Cardiovasc Imaging.

# Sub article: P184 CVD risk assessment using various tools in an HIV cohort in Greece

**Published:** 2016-10-23

**Authors:** Efmorfili Zouzia, Eleni Kakalou, Vasileios Papastamopoulos, Theodoros Kakkavas, Dionysios Dimas, Theophilos Rosenberg, Athanasios Skoutelis

**Affiliations:** 1 Medical School, MSc International Medicine-Health Crises Management, School of Medicine, National and Kapodistrian University of Athens, Greece; 2 Infectious Diseases Department, Evangelismos General Hospital, Athens, Greece

## Abstract

**Introduction**: ART has led to improvements in life expectancy but chronic diseases, including cardiovascular disease (CVD), have emerged as a major factor of morbidity and mortality among the HIV infected. Traditional CVD risk prediction tools have questionable accuracy in this population. Only the D:A:D algorithm has been specifically developed for HIV patients. This study aims: a) to describe the prevalence of CVD risk factors in an HIV-infected population using various CVD risk prediction tools; b) to compare the results calculated by standard CVD risk assessment tools with those of the D:A:D risk equation.

**Materials and methods**: A cross-sectional study was conducted in Evangelismos General Hospital in Athens, Greece. Patients attending the outpatient HIV clinic during the period of 1 to 31 March 2016 were included. A total of 120 patients were included and their data were analyzed. Electronic medical records were used to collect data. Seven cardiovascular risk assessment tools were used (Framingham CVD, Framingham Hard CHD, SCORE, PROCAM Health Check, CUORE, QRISK2 and D:A:D Risk Score). Agreement among results was assessed using Cohen's weighted kappa coefficient.

**Results**: 81.5% (95% CI 73.6–87.5) of participants were male and 76.3% (95% CI 67.8–83.0) were born in Greece. The mean age was 41.9 (SD 10.47) and transmission mode was sexual in 62.2% (95% CI 53.2–70.4) and intravenous drug use in 30.3% (95% CI 22.7–39) of cases; 67.8% were current smokers. D:A:D risk equation classified 8.9% as of low (L), 83% as of medium (M) and 8% as of high risk (H) for CVD. Respectively, other equations’ estimated results were: Framingham CVD (L:72% – M:20.6% – H:7.5%), Framingham Hard CHD (L:73.7% – M:18.4% – H:7.9%), CUORE (L:61.5% – M:37.4% – H:1.1%), SCORE (L:65.8% – M:30.8% – H:3.4%), PROCAM (L:90.4% – M:6.1% – H:3.5%) and QRISK2 (L:90.4% – M:4.4% – H:5.3%). Calculating weighted Cohen's kappa using three categories, “low-risk,” “medium-risk” and “high-risk,” coefficient values were very low (<0.2), indicating poor agreement between different tools. However, when categories “low-risk” and “medium-risk” were merged into one (“non-high risk”), Cohen's kappa resulted in significantly better agreement between the results of the various algorithms (in some cases κ~0.7) (Table 1).

**Conclusions**: General population CVD risk assessment tools underestimate CVD risk of HIV patients, especially in the “medium-risk” strata. D:A:D risk equation might be the tool of choice for this population [1,2]. Smoking prevalence is high in the present cohort and efforts should focus on assisting patients to quit smoking.

**Abstract P184–Table 1:** Agreement between D:A:D and other CVD calculation tools (high, non-high risk)

	Framingham CVD	Framingham Hard CHD	Score	PROCAM	QRISK2	CUORE
D:A:D						
K	0.729	0.191	0.434	0.595	0.266	0.206
95% CI	0.475, 0.982	−0.104, 0.485	0.092, 0.775	0.278, 0.912	−0.070, 0.602	−0.137, 0.548
p-value	<0.001	0.047	<0.001	<0.001	0.004	0.002

## References

[1] Nery MW, Martelli CMT, Aparecida Silveira E, Sousa CA, de, Falco M, de O, Castro A, de C Cardiovascular risk assessment: a comparison of the Framingham, PROCAM, and DAD equations in HIV-infected persons. Sci World J.

[2] Pirš M, Jug B, Eržen B, Šabović M, Karner P, Poljak M (2014). Cardiovascular risk assessment in HIV-infected male patients: a comparison of Framingham, SCORE, PROCAM and DAD risk equations. Acta Dermatovenerol Alp Pannonica Adriat.

# Sub article: P185 Myocardial inflammatory changes before and after ART in HIV-infected patients with advanced disease

**Published:** 2016-10-23

**Authors:** Alicia Piñeirua Menendez, Rommel Flores Miranda, Dulce Adoración Sánchez Nava, Raúl Ortega Pérez, Francisco Belaunzaran Zamudio, Santiago Pérez Patrigeon, Aylin Cardenas Ochoa, Jorge Oseguera Moguel, Jaime Galindo Uribe, Consuelo Orihuela Sandoval, Zuilma Yurith Vásquez Ortiz, Jorge Vásquez Lamadrid, Martha Morelos Guzmán, Sandra Rosales Uvera, Brenda Crabtree Ramírez, Juan Sierra Madero

**Affiliations:** 1 Infectious Diseases Department, Clínica Especializada Condesa/Iztapalapa, Mexico City, Mexico; 2 Infectious Diseases Department, Instituto Nacional de Ciencias Médicas y Nutrición Salvador Zubirán, Mexico City, Mexico; 3 Cardiology Department, Instituto Nacional de Ciencias Médicas y Nutrición Salvador Zubirán, Mexico City, Mexico; 4 Radiology Department, Instituto Nacional de Ciencias Médicas y Nutrición Salvador Zubirán, Mexico City, Mexico

## Abstract

**Introduction**: The effect of immune restoration with ART on HIV-related cardiac inflammation is unknown. We investigated the presence of myocarditis before and after ART initiation in patients with HIV advanced disease.

**Materials and methods**: Myocardial inflammatory changes were studied with MRI, using Lake Louise Consensus Criteria [1] in ART-naïve, HIV-infected adults with CD4+ T cell counts <200 cells/µL, at ART initiation and 6 weeks later. Myocardial function was assessed with transthoracic echocardiogram. Troponin I, proBNP (heart-injury biomarkers) and serum antibodies and plasma PCR for cardiotropic pathogens were measured. Immune activation and lymphocyte differentiation were analyzed by flow cytometry.

**Results**: Seventeen patients were enroled, 15 (88%) were men. At baseline, median age was 34 years and CD4 count 46 cells/µL. No patients had cardiovascular-related symptoms at enrolment. We summarized in Table 1 the frequency of myocardial inflammation, myocardial dysfunction and pulmonary hypertension, and the presence of HHV-6, HHV-8 and parvovirus B19 at baseline and 6 weeks after ART in all subjects. Among those with baseline myocardial inflammation (n=6), three (50%) had systolic dysfunction and one had diastolic dysfunction. None had cardiovascular-related symptoms. Among the five (29%) patients with myocardial inflammation at week 6, two (40%) had systolic dysfunction, two (40%) diastolic and one more had both. One patient progressed to symptomatic heart failure after ART initiation. He had the most severe baseline systolic dysfunction (LVEF 41%), which resolved with medical treatment after 1 year of follow-up (LVEF 61%). No myocardial inflammation at baseline and at 6 weeks was observed in eight (47%) subjects; four (23%) had baseline inflammation that spontaneously resolved after 6 weeks; inflammation persisted after 6 weeks of ART in 2/6 patients, and three more developed new inflammation after ART. Baseline and 6-week IgG for *T. gondii*, CMV and EBV were frequent and not associated with myocardial inflammation. No evidence of past or present *T. cruzi* or Coxsackie virus was found. No association was found between myocardial inflammation and HPVB19, HHV-6 or -8, or with immune activation markers.

**Conclusions**: Subclinical myocarditis was common in this group of patients with HIV-associated advanced disease; and resolved spontaneously after ART initiation in most patients. Three patients developed myocarditis after ART initiation with no apparent associated infectious cause, suggesting a possible role of immune restoration disease. In one of them, myocardial inflammation caused heart failure requiring clinical management for 1 year. Awareness of this condition may improve management of those patients.

**Abstract P185–Table 1:** Frequency of abnormal findings in heart assessment, cardiotropic pathogens and CD4+ counts and HIV RNA measurements at baseline and 6 weeks after ART initiation in 17 patients starting treatment with advanced HIV disease (<200 CD4+ cells/mm^**3**^)

Variable	Basal n (%)	6 weeks n (%)
Myocardial inflammation	6 (35)	5 (29)
Systolic dysfunction (LVEF <60%)	5	3
Diastolic dysfunction (slow relaxation pattern)	2	3
Systolic + diastolic dysfunction	1	0
Pulmonary hypertension (TTE)	1	1
Plasma PCR for HHV6	0	1
Plasma PCR for HHV8	1	2
Plasma PCR for parvovirus B19	2	0
CD4+ cells/mm^3^	46 (18–81)	208 (90.5–205)
CD4 + /CD8+ ratio	0.063 (0.048–0.092)	0.25 (0.131–0.326)
HIV RNA copies/mm^3^	449,967 (227,367–740,959)	143 (87–502)

## References

[1] Friedrich MG, Sechtem U, Schulz-Menger J, Holmvang G, Alakija P, Cooper LT (2009). Cardiovascular magnetic resonance in myocarditis: a JACC White Paper. J Am Coll Cardiol.

# Sub article: P186 TDF/FTC/RPV + atorvastatin as comorbidity-driven cART

**Published:** 2016-10-23

**Authors:** Franco Maggiolo, Elisa Di Filippo, Laura Comi, Annapaola Callegaro, Daniela Valenti, Giampietro Gregis

**Affiliations:** 1 Infectious Diseases, ASST Papa Giovanni XXIII, Bergamo, Italy; 2 Laboratory of Microbiology and Virology, ASST Papa Giovanni XXIII, Bergamo, Italy

## Abstract

**Introduction**: Comorbidities are relevant in the management of HIV infection; however, few studies have considered the choice of ARV regimen based on non-HIV-dependent comorbidities.

**Materials and methods**: In this uncontrolled pilot study, we enroled patients with cardiovascular disease or diabetes. All were on an effective cART (HIV RNA <50 copies/mL for >6 months). Patients were switched to TDF/FTC/RPV STR and all received a 40 mg dose of atorvastatin. According to the American Heart Association indications [1], the reduction of LDL-cholesterol levels at 3 and 6 months were used as primary goal of the study.

**Results**: Twenty patients, half diabetics and half with a previous cardiovascular accident (e.g. stroke, MI, stent positioning), were enroled. Nineteen were males, with a mean age of 55 years (range 40–69). One-third were smokers. They had been on cART for a mean of 11 years (range 2–22) and on current cART for 4.8 years (range 0.6–13). At enrolment, all had HIV RNA <50 copies/mL with a mean CD4 count of 693 cells/mcL. Their copharmacy included aspirin and beta-blockers (40% each), antidiabetics, statins (35% each) ramipril, anti-lipid drugs (30% each) and a sartan (20%). Other medications were taken by 35% of subjects. All patients maintained viral suppression over time, a single virologic blip (60 copies/mL) was observed in one patient at 6 months. CD4 counts increased by 57 cells/mcL. Total cholesterol decreased from 206 (SD 33) to 144 mg/dL (SD 35), HDL from 46 (SD 19) to 39 mg/dL (SD 14) and LDL from 123 (SD 19) to 79 mg/dL (SD 24) (for all p<0.001); HDL/LDL ratio was normalized in all patients. D-dimer levels were studied to explore the anti-inflammatory, non-lipidic lowering effects of atorvastatin. They varied from 391 ng/mL (SD 263) at baseline to 311 ng/mL (SD 260) (p=0.010) at 3 months, to 319 ng/mL (SD 251) after 6 months (p=0.012). Therapy was well tolerated and CPK levels did not modify.

**Conclusions**: The management of comorbidities is paramount in HIV patients. Cardiovascular diseases are recognized as a major contributor to morbidity and mortality in HIV-infected subjects. TDF/FTC/RPV has a neutral lipid effect and no interactions with statins allowing for the use of these drugs at full dose. We demonstrated that the concomitant use of TDF/FTC/RPV and atorvastatin reduces the cardiovascular risk of HIV patients by significantly lowering both LDL and d-dimer blood levels while maintaining virologic suppression.

## References

[1] Stone NJ, Robinson JG, Lichtenstein AH, Bairey Merz CN, Blum CB, Eckel RH, American College of Cardiology/American Heart Association Task Force on Practice Guidelines 2013 ACC/AHA guideline on the treatment of blood cholesterol to reduce atherosclerotic heart association task force on practice guidelines cardiovascular risk in adults: a report of the American College of Cardiology/American Heart Association Task Force on Practice Guidelines. Circulation.

# Sub article: P187 Lipid profile in HIV patients with long-term ART: darunavir versus raltegravir versus rilpivirine

**Published:** 2016-10-23

**Authors:** Bianca Branco Ascenção, Isabel Casella, Catarina Gonçalves, Nuno Luís, Ana Teresa Goes, Joana Sá, Ana Paula Brito, José Poças

**Affiliations:** 1 Infectious Diseases, Centro Hospitalar de Setubal, Setubal, Portugal

## Abstract

**Introduction**: The long-standing use of ART in association with dyslipidaemia and cardiovascular events has previously been thoroughly studied. Protease inhibitors (PIs) have been linked to an increased risk of dyslipidaemia when compared with other ART groups such as integrase inhibitors (INIs) or non-nucleoside reverse transcriptase inhibitors (NNRTIs), namely, rilpivirine. Our aim was to describe the lipid profile outcome of three different drugs (darunavir, raltegravir and rilpivirine) in a real-life situation.

**Materials and methods**: We conducted an observational study in our Infectious Diseases Department. Eligible subjects included HIV-1 infected adults, with virologic suppression, under an ART regimen consisting of a backbone of two nucleoside reverse transcriptase inhibitors (NRTIs) plus a third agent: darunavir (group 1), raltegravir (group 2) and rilpivirine (group 3), for at least 1 year (2015). We evaluated the changes in the lipid profile in these groups, comparing the differences in total cholesterol (TC), high-density lipoprotein (HDL), low-density lipoprotein (LDL), triglycerides (TG), at baseline (without ART, under PIs, NNRTIs or INIs) and at current time.

**Results**: A total of 192 patients were included, 72.4% males with a mean age of 47.2 years. In group 1 (N=101), we observed a medium increase of 4 mg/dL in TC, with a major increase in LDL (17 mg/dL). However, medium TG value decreased 11 mg/dL. In subgroup analysis, 22 patients naïve at baseline had an increase of 34 mg/dL in TC (LDL: 25 mg/dL), with decrease in TG. Sixty-three patients initially under other PIs showed the same pattern. In group 2 (N=37), TC and TG decreased, but LDL increased 2 mg/dL. Six patients were naïve, and showed a TC increase of 25 mg/dL, and TG decrease 1 mg/dL; 21 patients on PIs had TC and LDL decreased (7 mg/dL, 5 mg/dL), with 62 mg/dL decline in TG. However, the 10 patients with NNRTIs at baseline presented with TC increase: 5 mg/dL; HDL decrease: 7 mg/dL, LDL increase: 11 mg/dL; and TG decrease: 5 mg/dL. In group 3 (N=54), all parameters of lipid profile showed a substantial decrease. In patients initially under PIs (N=13) TC decreased: 18 mg/dL; LDL: 11 mg/dL; TG: 28 mg/dL. Seventeen patients under NNRTIs showed 19 mg/dL decline in TC, 12 mg/dL in LDL and 25 mg/dL in TG. Even the naïve subgroup (N=22) showed a TC decrease 2 mg/dL and TG decrease 13 mg/dL. Adversely two patients under INIs showed an increase in all parameters.

**Conclusions**: Rilpivirine showed a better evolution in lipid profile, both in naïve and experienced patients, in comparison to darunavir and raltegravir. As this study considered real-life data, the information could be very useful in clinical practice and future ART decision making.

# Sub article: P188 Cardiovascular disease risk scores comparison in Serbian HIV-infected patients

**Published:** 2016-10-23

**Authors:** Bozana Dimitrijevic, Djordje Jevtovic, Ivan Soldatovic, Jovan Ranin, Dubravka Salemovic, Jovana Kusic, Gordana Dragovic

**Affiliations:** 1 Clinical Pharmacology and Toxicology, School of Medicine, University of Belgrade, Pharmacology, Belgrade, Serbia and Montenegro; 2 Infectious and Tropical Diseases Hospital, HIV/AIDS Center, Belgrade, Serbia and Montenegro; 3 School of Medicine, Institute for Biomedical Statistics, Belgrade, Serbia and Montenegro

## Abstract

**Introduction**: Increased rates of non-AIDS mortality, including cardiovascular diseases (CVD), emerged as an important issue in HIV-infected patients [1,2]. Thus, we aimed to estimate cardiovascular risk in HIV-infected patients using four cardiovascular risk scores recommended by different international guidelines: Framingham Risk Score (FRS), Systematic Coronary Risk Evaluation (SCORE), American Heart Association Atherosclerotic Cardiovascular Disease risk score (ASCVD) and one designed particularly for HIV-infected patients, Data Collection on Adverse Events of Anti-HIV Drugs (D:A:D) model. Furthermore, we also aimed to analyze the agreement of the high D:A:D CVD score with other high CVD scores and to calculate discriminative power for each of used scores in Serbian patient population.

**Materials and methods**: We included 202 patients in cross-sectional study conducted at HIV/AIDS Center at Clinic for Infectious and Tropical Diseases, Belgrade, Serbia from January 2014 to January 2015. We collected data on risk factors for CVD including age, gender, race, total cholesterol, systolic blood pressure, smoking status and also HIV-specific parameters such as duration and current use of lopinavir or abacavir, as well as family history. Inclusion criteria were at least 12 months on antiretroviral therapy and age range of 40 to 79 years. We calculated agreement between D:A:D score and three other scores using Cohen's kappa coefficient (κ). We also described discriminative power of each of the scores using receiver operating characteristic (ROC curves).

**Results**: All patients were Caucasians with median age of 49 years, 151 (74.8%) were males. As for traditional risk factors, 100 (49.5%) patients are current smokers, 64 (31%) had hypertension, while hypercholesterolemia was found in 72 (35.4%). Fifty-one (25.2%) persons were overweight (BMI >25), 15 (7.4%) were obese (BMI >30), 45 (22.3%) had metabolic syndrome and seven diabetes (3.5%). The prevalence of high CVD scores were 8%, 13%, 35% and 40% for SCORE, FRS, D:A:D and ASCVD score, respectively. The agreement between high D:A:D score and high ASCVD score was higher (k=0.73) than between the D:A:D score and FRS (k=0.59) or SCORE (k=0.60) algorithms. We also found that D:A:D score and ASCVD score had a highly significant predictive value for outcome in comparison with two other scores (Figure 1).

Among four estimated CVD risks, D:A:D score and ASCVD score had a highly significant predictive value for outcome. D:A:D score had the area under the receiver operator, ROC curve, AUC of 0.691 (p=0.004), while the ASCVD had the area under the ROC curve of 0.624 (p=0.05).

**Conclusion**: We found a high number of HIV-infected patients in our population who are in need of CVD risk reduction. We also found substantial agreement of D:A:D and ASCVD risk score in order to estimate CVD risk in Serbian patient population.

**Abstract P188–Figure 1:** Evaluation of discriminative power (D:A:D ROC = 0.691, ASCVD ROC 0.624).

## References

[1] Smith C (2010). Factors associated with specific causes of death amongst HIV-positive individuals in the D:A:D Study. AIDS.

[2] Mocroft A, Reiss P, Gasiorowski J, Ledergerber B, Kowalska J, Chiesi A (2010). Serious fatal and nonfatal non-AIDS-defining illnesses in Europe. J Acquir Immune Defic Syndr.

# Sub article: P189 The extent of B-cell activation and dysfunction preceding lymphoma development

**Published:** 2016-10-23

**Authors:** Leah Shepherd, Alvaro Borges, Richard Harvey, Mark Bower, Andrew Grulich, Michael Silverberg, Jonathan Weber, Matti Ristola, Jean-Paul Viard, Johannes Bogner, Panagiotis Gargalianos-Kakolyri, Cristina Mussini, Kamal Mansinho, Israel Yust, Alexandra Calmy, Dzmitry Paduta, David Jilich, Tomasz Smiatacz, Roxana Radoi, Janez Tomazic, Peter Plomgaard, Ruth Frikke-Schmidt, Jens Lundgren, Amanda Mocroft, EuroSIDA in Eurocoord

**Affiliations:** 1 Research Department of Infection and Population Health, University College London, London, UK; 2 Centre for Health & Infectious Disease Research (CHIP), Department of Infectious Diseases, Rigshospitalet, Copenhagen, Denmark; 3 Charing Cross Oncology Laboratory and Trophoblast Disease Centre, Charing Cross Hospital Campus of Imperial College Healthcare National Health Service Trust, London, UK; 4 National Centre for HIV Malignancy, Chelsea and Westminster Hospital NHS Foundation Trust, London, UK; 5 Kirby Institute, The University of New South Wales, Sydney, Australia; 6 Department of Research, Kaiser Permanente, North California, CA, USA; 7 Department of Medicine, Imperial College London, London, UK; 8 Division of Infectious Diseases, University of Helsinki and Helsinki University Hospital, Helsinki, Finland; 9 Centre de Diagnostic et de Thérapeutique, Hôpital de l'Hôtel-Dieu, AP-HP, Paris, France; 10 Department of Internal Medicine IV, University of Munich, Munich, Germany; 1 First Department of Internal Medicine and Infectious Diseases Unit, General Hospital of Athens, Athens, Greece; 2 Clinic of Infectious and Tropical Diseases, University of Modena and Reggio Emilia, Azienda Policlinico, Modena, Italy; 3 Department of Infectious Diseases, Hospital Egas Moniz – Centro Hospitalar Lisboa Ocidental (CHLO), EPE, Lisboa, Portugal; 4 Internal Medicine, Ichilov Hospital, Tel-Aviv, Israel; 5 HIV Unit, Division of Infectious Diseases, Geneva University Hospitals, Geneva, Switzerland; 6 Epidemiology and Healthcare, GOCGEIOZ (Gomel Regional Centre for Hygiene), Burcharest, Belarus; 7 Department of Infectious and Tropical Diseases, First Faculty of Medicine, Charles University, Prague, Czech Republic; 8 Infectious Diseases Department, Medical University of Gdansk, Gdansk, Poland; 9 HIV, Clinical Hospital of Infectious and Tropical Diseases, Victor Babes, Bucharest, Romania; 10 Department of Infectious Diseases, University Medical Center Ljubljana, Ljubljana, Slovenia; 1 Department of Clinical Biochemistry, Rigshospitalet, Copenhagen, Denmark; 2 Department of Clinical Biochemistry, Rigshospitalet, Copenhagen, Denmark

## Abstract

**Introduction**: B-cell dysfunction and activation is thought to contribute to lymphoma development in HIV+ people; however, the mechanisms are complex and not well understood. We investigated markers of B-cell dysfunction prior to lymphoma diagnosis.

**Materials and methods**: A nested case control study of 73 HIV+ people with lymphoma (52 non-Hodgkin lymphoma and 21 Hodgkin lymphoma) and 142 matched controls within EuroSIDA was conducted. Cases and controls were matched on date of first and last sample preceding lymphoma, age and CD4 cell count at first sample, gender and region of Europe. Prospectively stored plasma samples before lymphoma (or matched date in controls) were measured for markers of B-cell dysfunction and activation: free light chain [FLC]-kappa, FLC-lambda, immunoglobulin [Ig]G, IgA, IgM and IgD. Conditional logistic regression investigated associations between markers and lymphoma <2 and >2 years prior to diagnosis.

**Results**: A total of 215 HIV+ people were included with a median of 2.0 (IQR 0.4–4.3) years between first sample and end of follow-up. Considering cases and controls together, all markers were correlated with lower CD4 level (FLC-lambda: Spearman's ρ=−0.27, p<0.01; FLC-kappa: ρ=−0.24, p<0.01; IgG: ρ=−0.27, p<0.01; IgA: ρ=−0.13, p<0.01; IgM: ρ=−0.17, p<0.01; IgD: ρ=−0.09, p<0.01). FLC-lambda (ρ=0.32, p<0.01), FLC-kappa (ρ=0.28, p<0.01), IgG (ρ=0.40, p<0.01) and IgM (ρ=0.40, p<0.01) were also positively correlated with HIV-VL. In the years prior to diagnosis, levels of FLC-kappa were stable in cases but increasing in controls by 4% (95% CI 1–8%) per year (p-value for difference =0.10). Levels of IgG and IgM were declining in cases by 4% (95% CI 1–6%) and 9% (95% CI 4–14%) per year, respectively, but stable in controls (p: for different in slopes in cases and controls: 0.10 and p<0.01 respectively). Levels of FLC-lambda, IgA and IgD were similar in cases and controls over time (all p>0.05). Elevated FLC-lambda (OR 3.28, 95% CI 1.47–7.7), FLC-kappa (OR 3.40, 95% CI 1.05–14.50) and IgG (OR 2.67, 95% CI 1.20–6.28) were associated with higher odds of lymphoma >2 years prior to diagnosis; however, levels were not predictive within 2 years prior to diagnosis (Figure 1). A similar trend was observed for IgM; however, significance was borderline with high uncertainty (>2 years OR 9.10, 95% CI 1.00–433.52; <2 years OR 7.12, 95% CI 0.69–354.54). IgA and IgD were not associated with lymphoma.

139 (cases=46, controls=93) people had samples <2 years prior to diagnosis and 156 (cases=53, controls=103) people had samples >2 years prior to diagnosis.

**Conclusions**: FLC-lambda, FLC-kappa and IgG were higher than 2 years before lymphoma diagnosis, but the difference diminished nearer diagnosis. B-cell dysfunction, as demonstrated by polyclonal hyperglobulinemia, occurs many years prior to lymphoma development.

**Abstract P189–Figure 1:** Odds ratios of lymphoma in those with a marker level of greater than the upper limit of the normal (ULN) relative to a level in the normal range, <2 and >2 years prior to diagnosis.

# Sub article: P190 Survival in HIV-1 infected individuals with diagnosis of lymphoma compared to general population: data from ICONA Foundation cohort study

**Published:** 2016-10-23

**Authors:** Antonella Cingolani, Alessandro Cozzi Lepri, Luciana Teofili, Laura Galli, Valentina Mazzotta, Gian Maria Baldin, Stefan Hohaus, Alessandra Bandera, Lucia Alba, Nadia Galizzi, Antonella Castagna, Antonella d'Arminio Monforte, Andrea Antinori, Study Group on behalf of Icona Foundation

**Affiliations:** 1 Infectious Diseases, Catholic University, Roma, Italy; 2 Department of Infection & Population Health, University College London, London, UK; 3 Hematology, Catholic University, Roma, Italy; 4 Infectious Diseases, San Raffaele Hospital, Milano, Italy; 5 Clinical Department, National Institute for Infectious Diseases, Roma, Italy; 6 Infectious Diseases, San Gerardo Hospital, Milano, Italy; 7 Infectious Diseases, San Paolo Hospital, University of Milano, Milano, Italy; 8 Health Sciences, San Paolo, Milano, Italy

## Abstract

**Introduction**: There are limited data on comparison of clinical outcome of lymphoma in HIV-positive (HIV-L) versus HIV-negative individuals (nHIV-L). Objectives of our analysis were to estimate overall survival (OS) after a diagnosis of lymphoma, comparing HIV-L versus nHIV-L and to identify predictors of death.

**Materials and methods**: All HIV-infected patients with a diagnosis of HIV-L (non-Hodgkin lymphoma, NHL; Hodgkin disease, HD) between 1 January 2000 and 31 December 2013 in ICONA or in three collaborating hospital databases were included. As controls, patients with nHIV-L seen for care in one of these centres over the same time period were included. Survival estimates by kaplan meier (KM) and predictors of OS by multivariable Cox regression after adjusting for main potential confounders (calendar year, age, gender, international prognostic index (IPI), treatment) were performed.

**Results**: A total of 1355 patients were included: 488 HIV-L (343 NHL and 145 HD) and 867 nHIV-L (589 NHL and 278 HD). Median age 49 years (IQR 38–64), 522 female (38%) and 423 (32%) had HD; of NHL, 765 (84%) were diffuse large B-cell lymphoma (DLBCL); among HIV-L, 91 (22%) were intravenous drug user (IVDU), median CD4+ count at lymphoma diagnosis 235 cells/mm3 (IQR 134–428) and 443 (91%) were on cART. HIV-L was more aggressive than nHIV-L (worse IPI score). The 3-year cumulative probability of death was 34% for HIV-L (95% CI 30–38) and 18% (15.5–20.8) for nHIV-L (log rank p=0.001). In univariable analysis, a significantly increased 3-year cumulative probability of death for HIV-L compared to nHIV-L was reported for all NHL (38.9% vs. 22.1%; p<0.001), for DLBCL (36.9% vs. 22.5%; p=0.008), for HD (22.3% vs. 10.1%; p<0.001). Unadjusted and adjusted hazard ratio (HR) of death according to HIV status in all NHL, in DLBCL and in HD from fitting separate Cox regression models are shown in Table 1. Results were mostly consistent when we performed a matched-cohort analysis using propensity scores, and restricting analysis, among HIV-L, to cART-treated only. Considering only NHL in HIV, older age (HR 1.45 (1.26–1.66)) and higher IPI (1.39 (0.87–2.23)) were independently associated with increased risk of death, whereas female gender (0.64 (0.45–0.90)) were associated with a decreased risk.

**Conclusions**: Comparing a large population of HIV-L and nHIV-L, we found an increased risk of death associated with HIV. The excess of risk independently attributable to HIV status ranged between 37% for DLBCL and >2-fold higher for HD, after controlling for unbalanced aggressive presentation and advanced stage at diagnosis. We cannot rule out bias due to other unmeasured confounding.

**Abstract P190–Table 1:** Unadjusted and adjusted HR of death in all NHL, in DLBCL and in HD from fitting three separate multivariable Cox regression hazard models

	Unadjusted HR (95% CI)	p	Adjusted^a^ HR (95% CI)	p	Adjusted^b^ HR (95% CI)	p	Adjusted^c^ HR (95% CI)	p
All NHL								
HIV-	1.00		1.00		1.00		1.00	
HIV +	1.63 (1.29–2.06)	<0.001	2.08 (1.56–2.76)	<0.001	1.35 (1.03–1.78)	0.03	1.52 (1.09–2.12)	0.01
DLBCL								
HIV-	1.00		1.00		1.00		1.00	
HIV +	1.44 (1.09–1.91)	0.01	1.83 (1.33–2.53)	<0.001	1.32 (0.96–1.81)	0.08	1.37 (0.95–1.99)	0.09
			Adjusted^a^ RH (95% CI)		Adjusted^d^ RH (95% CI)		Adjusted^e^ RH (95% CI)	
HD								
HIV-	1.00		1.00		1.00		1.00	
HIV +	2.36 (1.50–3.70)	<0.001	2.26 (1.37–3.74)	0.001	2.15 (1.13–4.09)	0.006	2.10 (1.08–4.06)	0.007

a Adjusted for age gender and calendar year

b adjusted for rituximab and IPI

c adjusted for factors in a and b

d adjusted for ABVD regimen and staging

e adjusted for factors in a and d.

# Sub article: P191 Cervical and breast cancer screening practices among women living with HIV

**Published:** 2016-10-23

**Authors:** Kate Salters, Monica Ye, Ann Burchell, Neora Pick, Danielle Smith, Shahab Jabbari, Mark Hull, Jeannie Shoveller, Julio Montaner, Robert Hogg

**Affiliations:** 1 Faculty of Health Sciences, Simon Fraser University, Burnaby, Canada; 2 Epidemiology & Population Health, British Columbia Centre for Excellence in HIV/AIDS, Vancouver, Canada; 3 Dalla Lana School of Public Health, University of Toronto, Toronto, Canada; 4 Oak Tree Clinic, BC Women's Hospital, Vancouver, Canada; 5 AIDS Research Program, British Columbia Centre for Excellence in HIV/AIDS, Vancouver, Canada

## Abstract

**Introduction**: Research suggests significant gaps exist in cancer screening practices for women living with HIV (WLWH); however, there is limited research from regions where screening is universally available. Annual Pap tests are considered best practice for WLWH, in response to the higher rates of cervical cancer. Current mammography recommendations suggest once every 24 months for women aged 40 to 74, with no specification for WLWH or immune-suppressed women.

**Materials and methods**: The comparison of outcomes and service utilization trends (COAST) study provides a retrospective, population-based cohort of HIV-positive individuals between 1996 and 2013 in British Columbia, Canada. The primary outcome variables (mammography for breast cancer and pelvic exams and/or Pap tests for cervical cancer screening) were identified by physician billing codes. Screening was identified between HIV diagnosis date and December 2013 as well as within the previous 12 and 24 months for cervical and breast cancer, respectively. Multivariate logistic model identified factors associated with receipt of breast and cervical cancer screening since HIV diagnosis.

**Results**: Of the 1070 WLWH between ages 40 and 74 in our study, 198 (18.5%) received at least one mammogram since being diagnosed with HIV, and only 61 (5.7%) in the previous 24 months. Additionally, among 1683 WLWH between ages 25 and 69, 628 (37.3%) received at least one Pap test since being diagnosed with HIV and only 97 (5.8%) in the previous 12 months. Receipt of Pap test since known HIV diagnosis date was less likely for individuals who have used injection drugs (AOR 0.62, 95% CI 0.50–0.77), are of Indigenous ancestry (AOR 0.66, 95% CI 0.50–0.87) and urban dwellers (AOR 0.50, 95% CI 0.34–0.74), but more likely for older individuals (AOR 1.16, 95% CI 1.05–1.28) and those with higher baseline CD4 cell count (AOR 1.11, 95% CI 1.05–1.16). Receiving a mammogram since HIV diagnosis was less likely for WLWH who were diagnosed with HIV after the year 2000 (compared to 1996 to 2000) (AOR 0.59, 95% CI 0.43–0.81) and those of Indigenous ancestry (AOR 0.59, 95% CI 0.40–0.87), but more likely for older individuals (AOR 1.96, 95% CI 1.70–2.26).

**Conclusions**: An alarmingly small proportion of WLWH in our sample received a mammogram and Pap test since being diagnosed with HIV despite current recommendations. This is notably low, even when accounting for completeness of administrative data such as physician billing codes. Of note, there may be significant barriers to screening for Indigenous WLWH and those with advanced HIV infection.

# Sub article: P192 Immune suppression at cART initiation is associated with cancer development in women living with HIV/AIDS

**Published:** 2016-10-23

**Authors:** Kate Salters, Monica Ye, Neora Pick, Danielle Smith, Sam Wiseman, Shahab Jabbari, Mark Hull, Jeannie Shoveller, Julio Montaner, Robert Hogg

**Affiliations:** 1 Faculty of Health Sciences, Simon Fraser University, Vancouver, Canada; 2 Epidemiology, British Columbia Centre for Excellence in HIV/AIDS, Vancouver, Canada; 3 Oak Tree Clinic, BC Women's Hospital, Vancouver, Canada; 4 Department of Surgery, St Paul's Hospital, Vancouver, Canada; 5 AIDS Research Program, British Columbia Centre for Excellence in HIV/AIDS, Vancouver, Canada

## Abstract

**Introduction**: The elevated risk of AIDS-defining malignancies (ADM) among people living with HIV has been largely and directly attributed to cell-mediated immune suppression, characterized by low CD4 counts. Since the advent of modern cART, rates of ADM have subsequently declined; however, the potential protective effect of early cART therapy initiation on cancer incidence, particularly non-ADM, remains largely unknown.

**Materials and methods**: The comparison of outcomes and service utilization trends (COAST) study provides a retrospective, population-based cohort of HIV-positive individuals between 1996 and 2013 in British Columbia, Canada. For this study, we included women with a confirmed HIV diagnosis. Incident cancer cases were identified by International Classification of Diseases for Oncology (ICD-O)codes. We conducted a Poisson regression to determine correlates of all-type cancer, ADM and non-ADM, and an adjusted model to determine incidence rate (per 1000 PY) by baseline CD4 cell count (<200, 200–350 and >350 cells/mm^3^) among women living with HIV (WLWH). We also calculated the attributable fraction (AF) of malignancies associated with CD4 count at cART initiation.

**Results**: Among 1660 WLWH included in this study, 50 WLWH were diagnosed with cancer between 1996 and 2013 (31 ADM and 19 non-ADM). Compared to WLWH without cancer, WLWH with a cancer diagnosis were more likely to have lower baseline CD4 (median 135 (IQR 60–260) cells/mm^3^ vs. 260 (IQR 140–390) cells/mm^3^), nadir CD4 (median 45.0 (10–101) cells/mm^3^ vs. 133 (43–250) cells/mm^3^) and a higher proportion of AIDS-defining illness at baseline (26.0% vs. 10.3%). Initiating cART with higher baseline CD4 cell count (>350 cells/mm^3^) is associated with lower all-type cancer diagnosis (RR 0.33 (95% CI 0.16–0.70)) and non-ADM diagnosis (RR 0.15 (95% CI 0.03–0.64)) compared to those to initiate cART with CD4 of <200 cells/mm^3^. No significant association was found between baseline CD4 and incidence of ADM diagnosis. After adjusting for age at HIV diagnosis, the incidence rate of all-type cancer is 5.55 (95% CI 3.89–7.91) cases per 1000 PY with AF of 63.66% and non-ADM incidence is 2.50 (95% CI 1.47–4.27) cases per 1000 PY with AF of 82.06% for those with a CD4 of <200 cells/mm^3^, compared to those with CD4 of >350 cells/mm^3^ at cART baseline.

**Conclusions**: Early initiation of cART may be protective against all-type cancer and non-ADM diagnosis. In the context of “Treatment as Prevention,” this study suggests there may be significant oncologic health benefits of early treatment initiation for some WLWH.

# Sub article: P193 High rate of the progression of low squamous intraepithelial lesion (LSIL) to high squamous intraepithelial lesion (HSIL) in a cohort of HIV MSM in “the modern antiretroviral era”

**Published:** 2016-10-23

**Authors:** Carmen Hidalgo-Tenorio, Concepcion Gil Anguita, Jessica Ramirez Taboada, Miguel Angel Lopez Ruz, Mohamed Omar Momamed Balghata, Rosario Javier, Javier Esquivias, Marina Gutierrez, Juan Pasquau

**Affiliations:** 1 Infectious Disease Service, Complejo Hospitalario Universitario, University Hospital Virgen de las Nieves, Granada, Spain; 2 Infectious Disease Unit, Complejo Universitario Hospitalario de Jaen, Jaen, Spain; 3 Pathology Service, Complejo Hospitalario Universitario, University Hospital Virgen de las Nieves, Granada, Spain

## Abstract

**Introduction**: Anal squamous cell carcinoma (ASCC) is one of the most frequent non-AIDS-defining malignancies in HIV-infected MSM [1]. A protocol of early diagnosis of ASCC has been considered cost-effective.

**Materials and methods**: This is a single-centre study conducted between May 2010 and June 2015. The patients were included in a screening, therapeutic and prophylaxis (implement use of condom, and qHPV vaccine (n=64 patients)) programme of HPV and ASCC. Baseline visit (V0) enclosed HPV PCR genotyping (GeneAmp PCR System 9700, Applied Biosystems), cytology and high-resolution anoscopy (HRA). In V0 and each visit, we collected medical history, sexual habits, CD4 and HIV viral load. Patients diagnosed with LSIL were subjected to an annual check-up that included HPV testing and HRA; patients diagnosed with HSIL were sent to the general surgery service where they underwent a mucosectomy; or they received intra-anal imiquimod three times/week for 16 weeks. When ASCC was diagnosed, the patient was sent to the Oncology Service; patients with normal HRA were evaluated every year with anal cytology and HPV PCR, in cases of anal squamous intraepithelial lesions and/or oncogenic HPV, a HRA was carried out. The cytologic and histologic classification was Bethesda's and LASTS Project for HPV-Associated Lesions, respectively.

**Results**: Two hundred and seventy-seven patients were included, with an average age of 36.8 years, and follow-up during 18.1 months/patient (IQR 0–34). In V0, 277 HRA were carried out: 40.8% were normal, 44.4% LSIL, 14.4% HSIL and 0.4% ASCC. IR of HSIL was 78.4×1000 person-years, and IR of ACSS 242×100,000 person-years. 16.1% and 1.6% of patients with normal HRA progressed to HSIL and ASCC, respectively. 19.1% of patients with LSIL progressed to HSIL. In the multiple logistic regression analysis, we observed, as a predictive factor of a new case of HSIL, previous LSIL in HRA, OR 5 (95% CI 1.6–15.9). The rest of variables analyzed (history of AIDS-defining illnesses, median time of HIV duration, antiretroviral therapy, education, employment, smoking, alcohol, STDs, genotypes or number of HPV, viral load, CD4 cells/uL, qHPV vaccine, imiquimod and mucosectomy) were not related.

**Conclusions**: One in every five of patients with LSIL progressed to premalignant lesions in 18 months. The only risk factor associated with the high IR of HSIL was preliminary diagnosis of low squamous intraepithelial lesions.

## References

[1] Shiels MS, Cole SR, Kirk GD, Poole C (2009). A meta-analysis of the incidence of non-AIDS cancers in HIV-infected individuals. J Acquir Immune Defic Syndr.

# Sub article: P194 Relapse of HIV-associated multicentric Castleman's disease following rituximab-based immunotherapy

**Published:** 2016-10-23

**Authors:** Alessia Dalla Pria, Jennifer Roe, Margherita Bracchi, Nicole Pagani, Katie McFaul, John Forni, Shairoz Merchant, Amit Samani, Ruth Byrne, Mark Nelson, Mark Bower

**Affiliations:** 1 National Centre for HIV Malignancy, Chelsea and Westminster Hospital, London, UK; 2 HIV-GUM, Chelsea and Westminster Hospital, London, UK

## Abstract

**Introduction**: The management of HIV-associated multicentric Castleman's disease (MCD) was revolutionized by the introduction of rituximab-based immunochemotherapy in 2003. However, relapses may occur following treatment and the clinicopathological features and outcomes after relapse of MCD have not been described.

**Materials and methods**: A retrospective review of prospectively collected data on 83 patients treated with rituximab-based therapy at the National Centre for HIV Malignancy.

**Results**: Eighty-four patients (72 male, mean age 42 years) were treated with rituximab-based immunotherapy for MCD (median plasma HHV8 viral load at MCD diagnosis 375,000 copies/mL). Four died from refractory or progressive MCD within the first month of treatment and 80 achieved clinical remission. The median follow-up for these 80 patients is 4.2 years, the 5-year overall survival is 92% (95% CI 84–99%) (Figure 1) and seven patients have died (three from HHV8-related lymphomas, one pulmonary Kaposi Sarcoma (KS), two suicides and one MCD (at fourth relapse).

Fifteen of 80 who achieved remission have relapsed at least once with biopsy-confirmed relapsed MCD (including three patients with concurrent lymphoma). The median time to first relapse is 22 months (range 8–94). At first relapse all 15 had symptoms of median duration 2 months (compared to 4 months at first diagnosis) and detectable plasma HHV8 viraemia. The median CD19 (B-cell) count at relapse was 474/mL (16%) suggesting that this had recovered following rituximab first-line therapy. The 5-year relapse-free survival for patients achieving remission is 78% (95% CI 67–89%) (Figure 2).

The risk of relapse was not influenced by gender (p=0.7), age (p=0.1), time since HIV diagnosis (p=0.3), prior AIDS diagnosis (p=0.2), plasma HIV viraemia (p=0.9), use of antiretroviral therapy (p=0.1), CD4 (p=0.9), CD8 (p=0.1), CD19 (B cell) (p=0.4) and CD16/56 (Natural Killer (NK) cell) (p=0.5) counts. The plasma HHV8 at MCD diagnosis (p=0.4) and the addition of chemotherapy to rituximab for high-risk patients (p=0.2) similarly did not affect the relapse risk. All 12 patients with no lymphoma at relapse were retreated with rituximab-based immunotherapy and all achieved a second clinical remission. Five have had second relapses also successfully treated and three have had third relapses including one patient who died from progressive MCD at fourth relapse 9.4 years after first MCD diagnosis.

**Conclusions**: Relapse following rituximab-based treatment for MCD is not infrequent and may occur after recovery of CD19 (B cell) counts. Clinical, virological and immunological predictors of relapse have not been identified. Relapses are usually sensitive to rechallenging with rituximab-based immunochemotherapy.

**Abstract P194–Figure 1:** Five-year overall survival of 84 patients treated with rituximab-based immunotherapy for HIV-associated MCD: 92% (95% CI 84–99%).

**Abstract P194–Figure 2:** Five-year relapse-free survival for patients achieving remission from HIV-associated MCD following rituximab first-line therapy: 78% (95% CI 67–89%).

# Sub article: P195 Incidence of cancer in a cohort of HIV-positive patients on virologically suppressive antiretroviral therapy in western India: a resource-limited setting perspective

**Published:** 2016-10-23

**Authors:** Ameet Dravid, Padmaj Kulkarni, Chinmay Saraf, Milind Kulkarni, Sachin Kore, Niranjan Rathod

**Affiliations:** 1 Department of HIV Medicine, Ruby Hall Clinic, Pune, India; 2 Oncology, Deenanath Mangeshkar Hospital, Pune, India; 3 Pathology, Precision Diagnostics and Biosciences, Pune, India; 4 Department of HIV Medicine, Ashwini Sahakari Rugnalaya, Solapur, India; 5 Department of HIV Medicine, Apex Hospital, Kolhapur, India

## Abstract

**Introduction**: With advent of antiretroviral therapy, non-communicable diseases, including malignancies, are increasingly contributing to morbidity and mortality among HIV-infected patients. Data on incidence of cancer (AIDS-defining and non-AIDS-defining malignancies) in patients on virologically suppressive ART from resource-limited settings like India are rare.

**Materials and methods**: HIV-infected patients following up at a private HIV clinic from February 2009 to 2016 and on virologically suppressive ART (plasma viral load <1000 copies/mL) were included. Patients presenting with incident cancer were recorded. Histopathology examination, immunohistochemistry testing, bone marrow and cerebrospinal fluid examination and positron emission tomography scan were done for diagnosis, staging and prognostication of cancer. Cox proportional hazard model was developed to assess relationship between time to cancer and covariates namely age, gender, baseline CD4, hepatitis B co-infection, baseline addictions and duration of virologically suppressive ART.

**Results**: A total of 1431 HIV-infected individuals (36% females) with median follow-up on suppressive ART of 40 months were included. Median age was 40 years and median baseline CD4 count 161 cells/mm^3^. Of these, 39 patients had diagnosis of incident cancer with an incidence of 7.29 (95% CI 5.32–9.97) episodes per 1000 person-years. Non-Hodgkin's lymphoma (15/39), Hodgkin's lymphoma (4/39) and hepatocellular carcinoma (3/39) were the commonly diagnosed incident cancers in our cohort. Overall 18/39 (46.15%) patients had AIDS-defining cancers while 21/39 (53.85%) had non-AIDS-defining cancers. Eight of 21 (38.1%) patients had infection-related non-AIDS-defining cancers (i.e. hepatocellular carcinoma, anal cancer and Hodgkin's lymphoma) and 13/21 (61.9%) patients had infection-unrelated non-AIDS-defining cancer (i.e. lung cancer, ovarian cancer, cancer of oral cavity). Median time to development of cancer was 24 months. Male patients (p=0.039) and those with HIV/hepatitis B co-infection (p=0.018) were significantly associated with incident cancer (Table 1). Forty-one percent of patients died during treatment of incident cancer.

**Conclusion**: Spectrum of incident cancers in our cohort of virologically suppressed HIV patients is evenly distributed between AIDS-defining and non-AIDS-defining malignancies. Regular screening for cancer amongst elderly HIV-infected males and HIV/hepatitis B co-infected patients is warranted. Emphasis on tobacco de-addiction can further reduce cancer incidence.

**Abstract P195–Table 1:** Risk factors associated with incident cancer in Pune cohort

Factors	Hazard rates ratio (RR)	p value	95% CI for hazard rates ratio
Age >40 years	1.886	0.056	0.983–3.620
Male:female	2.515	0.039	1.046–6.046
Baseline hepatitis B	3.835	0.005	1.490–8.974
Baseline CD4 count ≤50	1.881	0.086	0.914–3.868

# Sub article: P196 Self-administered treatment with imiquimod 5% cream for intra-anal HSIL (AIN2/3) in HIV-positive patients: efficacy, safety and a comfortable option

**Published:** 2016-10-23

**Authors:** Carmen Hidalgo-Tenorio, Concepcion Gil Anguita, Jessica Ramirez Taboada, Samantha Elisabeth De Jesus, Javier Esquivias, Mercedes Alvarez, Marina Gutierrez, Miguel Angel Lopez Ruz, Rosario Javier, Juan Pasquau

**Affiliations:** 1 Infectious Disease Service, Complejo Hospitalario Universitario Granada, University Hospital Virgen de las Nieves, Granada, Spain; 2 Pathology Service, Complejo Hospitalario Universitario Granada, University Hospital Virgen de las Nieves, Granada, Spain

## Abstract

**Introduction**: Anal squamous cell carcinoma (ASCC) is one of the most frequent non-AIDS-defining malignancies in HIV-infected patients; it is closely related to the infection by oncogenic HPV genotypes (HR-HPV). Currently there are diverse treatments for premalignant lesions with disappointing results. Imiquimod is a drug with anticarcinogenic and antiviral activity, and it is recommended for treatment of genital/perianal condylomas. It is not suggested for the treatment of anal HSIL; nevertheless, the results of two research studies about imiquimod in premalignant anal lesions in HIV-positive patients have been published with a cure rate of 45 to 61% [1,2]. The aim of this prospective study was to analyze the efficacy of 3 consecutive days a week for 4 months of self-administered treatment with imiquimod 5% cream for anal HSIL, and to assess safety, and antiviral activity against HPV, in seropositive patients.

**Methods**: Between May 2013 and May 2016, 20 HIV-positive patients with HSIL were enroled to self-apply imiquimod cream into the anal canal. Baseline visit (V0) and each visit enclosed cytology, HPV PCR genotyping (GeneAmp PCR System 9700, Applied Biosystems) and high-resolution anoscopy (HRA) (Zeiss 150 fc©), medical history, sexual habits, CD4 and HIV viral load. Response was assessed by cytology, HRA and biopsy 1 month after therapy, and annually. If HSIL persisted, the treatment with imiquimod was extended 6 weeks more. The cytologic and histologic classification was Bethesda's, and LASTS Project for HPV-Associated Lesions, respectively.

**Results**: The average age was 36.5 years, 95% MSM and 5% WSM, 50% had history of AIDS-defining illnesses, CD4 nadir 295 cells/mL and current CD4 577 cells/mL. Ninety percent antiretroviral therapy, and 94.7% viral load <50 copies/mL. Fifty percent were smokers, 20% had syphilis and 35% genital/perianal warts. Sixty-three percent of them used condoms in 100% of intimate relationships. Ninety-five percent had HR-HPV genotypes, and the most frequent was HPV16 (65%). The median of quadrants of anal canal affected with HSIL was 2 (IQR 1–2). Complete response was observed 90% of the patients (88% (16/18) received imiquimod 4 months, and 11.1% (2/18) required prolonged the treatment), the median disease-free time was 21 months (IQR 11.3–27.5). Ten percent dropped out due to intolerance. After imiquimod, the clearance rate of HR-HPV was higher than the acquisition rate, 28.6% versus 21.4%, respectively, p=0.014.

**Conclusions**: Imiquimod could be an effective, safe and comfortable option for the treatment of anal HSIL in HIV-positive patients; and it could exercise control over the infection of HR-HPV in anal mucosa.

## References

[1] Van der Snoeck EM, Den Hollander JC, van der Ende ME (2015). Imiquimod 5% cream for five consecutive days a week in an HIV-infected observational cohort up to 32 weeks in the treatment of high-grade squamous intraepithelial lesions. Sex Transm Infect.

[2] Fox PA, Nathan M, Francis N, Singh N, Weir J, Dixon G (2010). A double-blind, randomized controlled trial of the use of imiquimod cream for the treatment of anal canal high-grade anal intraepithelial neoplasia in HIV-positive MSM on HAART, with long-term follow-up data including the use of open-label imiquimod. AIDS.

# Sub article: P197 Investigating Barriers In HIV-Testing Oncology Patients: the IBITOP study phase II

**Published:** 2016-10-23

**Authors:** Tu Nguyen-Ngoc, Matthias Cavassini, Laurent Merz, Stefan Zimmermann, Solange Peters, Katharine Darling

**Affiliations:** 1 Department of Oncology, Oncology Centre, Lausanne University Hospital, Lausanne, Switzerland; 2 Infectious Diseases Service, Lausanne University Hospital, Lausanne, Switzerland

## Abstract

**Introduction**: The prevalence of non-AIDS-defining cancers (non-ADCs) is increasing among people living with HIV. Conversely, some non-ADCs are associated with HIV prevalence figures higher than those of the general population. After observing HIV testing rates below 5% in our oncology centre, we performed a study Investigating Barriers In HIV-Testing Oncology Patients (IBITOP I) among oncology physicians and patients at Lausanne University Hospital (LUH) [1]. We found that 18% of cancer patients were offered HIV testing although patient acceptance of testing was high (91%). After this study, the Swiss Federal Office of Public Health HIV testing recommendations were updated to include cancer patients undergoing chemotherapy in the lists of HIV testing indications. The study presented here, IBITOP II, examined HIV testing practices and physician barriers to testing following these recommendations.

**Methods**: Between 1 January and 31 October 2015, patients of unknown HIV status newly diagnosed with solid-organ non-ADCs referred to LUH Oncology Service, Lausanne, Switzerland, were offered free HIV testing as part of their oncology work-up. The primary endpoints were 1) physician proposition rates for HIV testing and 2) physician reasons for not offering testing.

**Results**: Of 438 patients of unknown HIV status with a new non-ADC diagnosis, 255 (58%) were offered HIV testing, of whom 42 declined (acceptance rate 213/255, 84%). Excluding 37 patients tested prior to their oncology consultation, 146 patients (of 438, 33%) were not offered testing. The most frequent physician reasons for not testing were: forgetting (35 patients, 24%); patient follow-up elsewhere (25 patients, 17%); no planned chemotherapy (25 patients, 17%); excessive burden of information for the patient (23 patients, 16%) and no time (21 patients, 14%).

**Conclusion**: This is the first study exploring physician reasons for not HIV-testing cancer patients despite current national HIV testing recommendations. Given the physician barriers we observe, testing will not be practised universally among cancer patients. Further, it is possible the testing rate of 58% will be lower outside the context of a study on testing. As HIV-positive status impacts on the medical management of cancer patients, knowledge of HIV status is important. We conclude that opt-out testing in this setting, conducted as part of the baseline oncology work-up, would circumvent physician barriers and optimize testing rates.

## References

[1] http://dx.doi.org/10.7448/IAS.17.4.19622.

# Sub article: P198 A descriptive study of cancer incidence in a cohort of HIV-infected patients followed since 1986

**Published:** 2016-10-23

**Authors:** Rocio Montejano, J Ramon Arribas, J Ignacio Bernardino, Luz Martin-Carbonero, M Luisa Montes, Victoria Moreno, Ignacio Perez-Valero, Juan Gonzalez-Garcia, Eulalia Valencia

**Affiliations:** 1 Infectious Diseases Unit, Hospital Universitario La Paz, Madrid, Spain

## Abstract

**Introduction**: HIV-infected patients are at increased risk of developing certain cancers, especially those related to viral infections [1,2].

**Patients and methods**: We conducted a retrospective cohort study on 4994 HIV-infected patients that were followed up in our hospital between 1986 and 2016. We evaluated the incidence of types of cancer occurring in this cohort.

**Results**: We detected 416 patients with at least one malignancy. Epidemiological data appears in Table 1 and Table 2.

HIV infection and cancer were simultaneously diagnosed in 111 patients (26.6%) and in the other 304 patients after a median of 7 years (IQR 1–15) of follow-up. The malignancy was diagnosed as clinically advanced in 35 patients (8.4%). The most frequent cancers were Kaposi's sarcoma (110, 26.4%; disseminated 40/110, 36.7%), cervix carcinoma (85, 20.4%), lymphoma (78, 18.7%; non-Hodgkin lymphoma 43/78, 55.84%), anal carcinoma (26, 6.25%), hepatocellular carcinoma (20, 4.8%), lung carcinoma (13, 3.1%) and head and neck tumours (11, 2.6%). Hundred and fifteen (27%) cancers were related to human papillomavirus (HPV) and 20 hepatocellular carcinoma (100%) had chronic HBV and/or HCV infection, 19/20 chronic HCV co-infection. After 15 years (IQR 8–21) of follow-up, 73 patients developed a second malignancy (mainly lymphoma and cervix carcinoma) and afterwards, 8 patients developed a third cancer. Non-AIDS-defining cancers (lung carcinoma, hepatocellular carcinoma and head and neck cancers) were significantly more frequent in late ART period (p<0.001 for all). No differences were found in the incidence of anal carcinoma. On the other hand, AIDS-defining cancers trended to decrease with the ART improvement. During follow-up, 62 patients died mainly due to progression of their malignancies (87.1%). Higher mortality was observed in patients with lung carcinoma (100%) and hepatocellular carcinoma (65%).

**Conclusions**: A malignancy was, in a substantial number of cases, the first manifestation of HIV infection. As expected, a significant proportion of cancers were related to other viral infections; especially HPV and hepatitis virus. Non-AIDS-defining cancers were more frequent during the late ART period and have high mortality.

**Table 1:** Baseline characteristics

	N (%)
Total	416
Male	279 (67%)
Median age at cancer diagnosis	42 (IQR 35–50)
Caucasian	367 (89.2%)
Risk behaviour	
- Sexual	239 (57.4%)
- Parenteral	130 (31.2%)
- Unknown	47 (11.3%)
Chronic HBV	17 (4%)
Chronic HCV	87 (20.9%)
AIDS	280 (67.3%)
CD4+ nadir (cells/mL)	132 (IQR 47–224)
Receiving ART on diagnosis	277 (69%)

**Abstract P198–Table 2:** Type of malignancy related to ART use and diagnosis time

	Pre-ART (before 1996)	Early ART (1996–2006)	Late ART (after 2006)	p
Total malignancies	46	148	222	
Lymphoma	3 (6.5%)	35 (23.6%)	40 (18%)	0.031
Kaposi's sarcoma	18 (39.1%)	56 (37.8%)	36 (16.2%)	<0.001
Cervix cancer	17 (36.9%)	38 (25.6%)	30 (13.5%)	<0.001
Anal cancer	4 (8.7%)	4 (2.7%)	18 (8.1%)	0.084
Hepatocellular carcinoma	0	2 (1.35%)	18 (8.1%)	<0.001
Lung carcinoma	0	0	13 (5.8%)	<0.001
Head and neck cancer	0	0	11 (4.9%)	<0.001

## References

[1] Rubinstein PG, Aboulafia DM, Zloza A (2014). Malignancies in HIV/AIDS. AIDS.

[2] Deeken JF, Tjen-A-Looi A, Rudek MA, Okuliar C, Young M, Little RF (2012). The rising challenge of non-AIDS-defining cancers in HIV-infected patients. Clin Infect Dis.

# Sub article: P199 Human papilloma virus and HIV

**Published:** 2016-10-23

**Authors:** Anna Popova, Alexey Kravtchenko, Marina Deulina, Olga Shipulina

**Affiliations:** 1 Russian Federal AIDS Center, Federal Budget Institution of Science Central Research Institute of Epidemiology, Moscow, Russian Federation; 2 Federal Budget Institution of Science Central Research Institute of Epidemiology, CMD, Moscow, Russian Federation

## Abstract

**Introduction**: In recent years in Russia, the number of cases of HIV infection transmitted heterosexually increased. This led to an increase in the number of women of reproductive age among patients with HIV infection. HIV-infected women have a higher risk of papillomavirus infection than HIV-negative women, as well as a higher risk of malignancy and persistence.

**Objective**: To study the prevalence of human papilloma virus (HPV) in HIV-infected women.

**Materials and methods**: We examined 561 people (155 (27.6%) HIV-positive patients and 406 (72.4%) HIV-negative women) from January 2014 to March 2016. For all women, HPV PAP test was performed.

**Results**: In the study group, young women up to 40 years (62.4%) were predominant. For 60 (38.7%) patients, HIV infection was diagnosed with HPV. In 92% of these patients, HPV concentration was greater than log & circ;3. In the control group, HPV was found to be 14.8%. HPV concentration in the control group was observed log  circ;3 more in 75% of cases. Analyzing the distribution of ASC-US and CIN, we discovered in the group with HIV infection that CIN1 and CIN2 substantially prevailed. The study showed a difference in the frequency of detection of certain genotypes in the study groups (Table 1).

In both groups, we found no combination of 16 and 18 genotypes. The most common changes in the results of cytogram PAP test were correlated with genotype 16. The group of HIV-infected women shared the leading position 16, 31 and 18 genotypes. The high oncogenic HPV types were more often detected in HIV-infected women.

**Conclusions**: In the group of HIV-infected women significant abnormalities associated with HPV were observed more than the control group. We must look for ways to solve this problem by advising HIV-positive women and their partners, conducting educational seminars, providing information on the need for screening for HPV among women living with HIV and the general population.

**Abstract P199–Table 1:** The distribution of HPV genotypes in the studied groups

Groups	HPV (%)	HPV concentration >log ^3	Mixed HPV genotypes	Leading genotypes
HIV infection	38.7%	92%	48.3%	18, 16, 1, 45
Control group	14.8%	75%	23.3%	16, 33, 45

# Sub article: P200 Glucose metabolism disorders, HIV and antiretroviral therapy among Tanzanian adults

**Published:** 2016-10-23

**Authors:** Emmanuel Maganga, Luke Smart, Samuel Kalluvya, Johannes Kataraih, Ahmed Saleh, Lama Obeid, Jennifer Downs, Daniel Fitzgerald, Robert Peck

**Affiliations:** 1 Internal Medicine, Weill Bugando Medical Center, Mwanza, Tanzania; 2 Internal Medicine, Weill Cornell Medicine, New York, USA; 3 Medical Education, Weill Cornell Medicine, Doha, Qatar

## Abstract

**Introduction**: Millions of HIV-infected Africans are living longer due to long-term antiretroviral therapy (ART), yet little is known about glucose metabolism disorders in this group. We aimed to compare the prevalence of glucose metabolism disorders among HIV-infected adults on long-term ART to ART-naïve adults and HIV-negative controls, hypothesizing that the odds of glucose metabolism disorders would be two-fold greater even after adjusting for possible confounders.

**Methods**: In this cross-sectional study conducted between October 2012 and April 2013, consecutive adults (>18 years) attending an HIV clinic in Tanzania were enroled in three groups: 153 HIV-negative controls, 151 HIV-infected, ART-naïve and 150 HIV-infected on ART for ≥2 years. The primary outcome was the prevalence of glucose metabolism disorders as determined by oral glucose tolerance testing. We compared glucose metabolism disorder prevalence between each HIV group versus the control group by Fisher's exact test and used multivariable logistic regression to determine factors associated with glucose metabolism disorders.

**Results**: HIV-infected adults on ART had a higher prevalence of glucose metabolism disorders (49/150 (32.7%) vs. 11/153 (7.2%), p<0.001) and frank diabetes mellitus (27/150 (18.0%) vs. 8/153 (5.2%), p=0.001) than HIV-negative adults, which remained highly significant even after adjusting for age, gender, adiposity and socioeconomic status (OR 5.72 (2.78–11.77), p<0.001). Glucose metabolism disorders were significantly associated with higher CD4+ T-cell counts. Awareness of diabetes mellitus was <25%.

**Conclusions**: HIV-infected adults on long-term ART had five-fold greater odds of glucose metabolism disorders than HIV-negative controls but were rarely aware of their diagnosis. Intensive glucose metabolism disorder screening and education are needed in HIV clinics in sub-Saharan Africa. Further research should determine how glucose metabolism disorders might be related to immune reconstitution.

# Sub article: P201 Decreasing incidence of diabetes mellitus in HIV-positive Taiwanese patients on combination antiretroviral therapy from 2004 to 2013

**Published:** 2016-10-23

**Authors:** Pei-Ying Wu, Shang-Ping Yang, Yu-Zhen Lou, Jun-Yu Zhang, Hsi-Yen Chang, Hsin-Yun Sun, Wang-Huei Sheng, Szu-Min Hsieh, Chien-Ching Hung

**Affiliations:** 1 Center of Infection Control, National Taiwan University Hospital, Taipei, Taiwan; 2 Department of Internal Medicine, National Taiwan University Hospital, Taipei, Taiwan; 3 Department of Internal Medicine, Center for Infection Control, National Taiwan University Hospital, Taipei, Taiwan

## Abstract

**Introduction**: The widespread use of combination antiretroviral treatment (cART) has led to a decrease of mortality and morbidity and improvement of survival in HIV-positive patients. However, increasing trends of metabolic complications including type 2 diabetes mellitus (DM) have threatened the long-term successful management of HIV infection. Our study aimed to evaluate the incidence of DM in ART-naïve HIV-positive Taiwanese adults who initiated ART in 2004 to 2013.

**Materials and methods**: Between 2004 and 2013, 1432 ART-naïve HIV-positive patients without DM initiated cART at the National Taiwan University Hospital. All patients were followed until the date when DM was diagnosed, 31 December 2015, loss to follow-up or death, whichever occurred first. Incident DM was defined as fasting glucose ≥126 mg/dL or HbA1C ≥6.5%. The trends of DM were compared between patients initiating cART in 2004 to 2008 (n=564 patients) and those in 2009 to 2013 (n=886).

**Results**: Over a total observation of 7632 person-years of follow-up (PYFU), DM was diagnosed in 28 patients, with an overall incidence rate of 3.7 per 1000 PYFU. While the rate increased with cumulative exposure to cART, from 0 per 1000 PYFU in patients with cumulative exposure to cART of <12 months to 3.9 per 1000 PYFU in those with cumulative exposure of ≥36 months, the overall rate decreased from 4.8 per 1000 PYFU in 2004–2008 to 1.2 per 1000 PYFU (p=0.02). The occurrence of DM was associated with an older age (adjusted hazard ratio (aHR) 1.049; 95% CI 1.013–1.085), exposure to boosted darunavir (aHR 3.287; 95% CI 1.168–9.254) and exposure to tenofovir/emtricitabine (aHR 0.194; 95% CI 0.072–0.522). The incident rate of DM increased with cumulative exposure to zidovudine/lamivudine duration: <12 months, 2.6 per 1000 PYFU; 12 to 24 months, 2.0 per 1000 PYFU; 24 to 36 months, 4.3 per 1000 PYFU; and ≥36 months, 4.8 per 1000 PYFU. In contrast, the rate remained stable with cumulative exposure to tenofovir/emtricitabine: <12 months, 0.9 per 1000 PYFU; 12 to 24 months, 1.2 per 1000 PYFU; 24 to 36 months, 1.3 per 1000 PYFU; and ≥36 months, 1.2 per 1000 PYFU. The rates were higher in patients with exposure to stavudine and/or didanosine, ranging from 7.7 to 10 per 1000 PYFU.

**Conclusions**: The incidence of DM in HIV-positive Taiwanese patients initiating cART decreased from 4.8 per 1000 PYFU in 2004–2008 to 1.2 per 1000 PYFU in 2009–2013. The trends of DM incidence varied with the cumulative exposure to different combination of nucleos(t)ide reverse transcriptase inhibitors.

# Sub article: P202 Comparison of risk tools to estimate type 2 diabetes risk in an urban HIV cohort

**Published:** 2016-10-23

**Authors:** Jonathan Mok, Louise Goff, Barry Peters, Alastair Duncan

**Affiliations:** 1 School of Medical Education, King's College London, London, UK; 2 Division of Diabetes and Nutritional Sciences, King's College London, London, UK; 3 Harrison Wing, Guy's and St Thomas’ Hospital NHS Foundation Trust, London, UK

## Abstract

**Introduction**: Type 2 diabetes (T2D) is more common in people living with HIV (PLHIV) than general populations, thought to be driven by HIV-specific and general factors. Early detection of risk is key to limit disease progression. Several clinical risk tools are available but do not account for the consequences of HIV infection. We aimed to compare the sensitivity and specificity of diabetes risk tools in PLHIV.

**Materials and methods**: A wide range of clinical factors was measured and recorded in a representative HIV-positive patient sample attending three London outpatient clinics. Glycaemic status was classified as: normal, prediabetes or T2D by fasting glucose (<6.0, 6.0–6.9 and ≥7.0 mmol/L respectively) or by previous diagnosis. T2D risk was calculated using three risk tools: the Finnish (FINDRISC), Q-Diabetes and the Data Collection on Adverse Events of Anti-HIV Drugs (D:A:D) risk scores. Percentage calculations were used to evaluate dysglycaemia prevalence against published national averages. Receiver operator characteristic (ROC) curves were used to calculate tool sensitivity and specificity.

**Results**: Of 338 patients sampled, 17.2% had prediabetes and 15.1% had T2D. In the general London population, the rates of prediabetes and T2D are 10.8% and 7.6% respectively (data collected elsewhere). The Q-Diabetes tool calculates T2D relative risk; the mean for this cohort was 2.4. ROC analysis suggested that D:A:D is the most sensitive and specific of the three tools for prediabetes, correctly identifying 100% of those affected (area under curve [AUC] 0.879; 95% CI 0.843–0.914; p<0.001), compared to FINDRISC and QDiabetes with 0.804 (95% CI 0.751–0.858; p<0.001) and 0.611 (95% CI 0.533–0.688; p<0.001) respectively. For T2D detection, D:A:D had the greatest specificity, followed by the FINDRISC score, identifying 96% and 90%, respectively (AUC 0.888; 95% CI 0.851–0.924; p<0.001 for D:A:D; for FINDRISC, AUC 0.825; 95% CI 0.775–0.875; p<0.001). Q-Diabetes had a comparable specificity of 84% for T2D (AUC 0.676; 95% CI 0.605–0.747; p<0.001) but the poorest sensitivity of the three tools tested (42%, 65% and 68% for Q-Diabetes, FINDRISC and D:A:D, respectively).

**Conclusion**: The D:A:D tool appears to be the most statistically sensitive and specific method for predicting both prediabetes and T2D in this HIV-positive cohort, with the FINDRISC tool also performing well. The Q-Diabetes tool, developed for use in the UK, has the lowest sensitivity of three tools. Pending development of an HIV-specific diabetes risk tool, the D:A:D tool should be used to estimate risk of T2D in PLHIV.

# Sub article: P203 Lipodystrophy as cause of metabolic syndrome: a contribution of antiretroviral drugs to increased cardiovascular risk

**Published:** 2016-10-23

**Authors:** Vanessa Muñoz-Mendoza, Maria Fontecha-Ortega, Cristina Gómez Ayerbe, Maria Jesús Vivancos Gallego, Matilde Sánchez Conde, Jose Luis Casado Osorio

**Affiliations:** 1 Internal Medicine, Hospital Obispo Polanco, Teruel, Spain; 2 Internal Medicine, Hospital de Getafe, Madrid, Spain; 3 Infectious Diseases, Hospital Ramón y Cajal, Madrid, Spain

## Abstract

**Introduction**: Lipodystrophy syndrome (LS) is characterized by abnormal fat distribution with visceral fat accumulation and peripheral lipoatrophy. The aim of this study was to evaluate the development of cardiovascular risk factors and the progression to metabolic syndrome in patients with previous lipodystrophy according to the severity of fat accumulation.

**Materials and methods**: Cross-sectional study of 276 HIV patients previously evaluated for the presence of lipodystrophy and its severity (absence, mild, moderate or severe) through HOPS questionnaire during 2004–2011. Patients were evaluated for fat accumulation by dual X-ray absorptiometry (DXA) and the presence of hypertension (HTA), diabetes mellitus (DM), waist circumference, insulin resistance and lipid alterations. Metabolic syndrome (MS) was defined according to ATP-III, IDF and AHA criteria.

**Results**: Mean age was 45.1 years (20–80), 80% were males, and prior fat accumulation was classified as absent in 37%, mild in 21%, moderate in 19% and severe in 23%. Mean BMI was 24.2 (16.1–34.5) and 6% had a BMI >30. The median time of HIV infection was 15 years (7–21). All patients with lipodystrophy had a history of prior therapy with thymidine analogues, and at the inclusion, 47% were receiving a PI and 53% an NNRTI. Median time from questionnaire to evaluation was 9.5 years. DXA scan showed a close correlation with severity of lipodystrophy by questionnaire. A systolic blood pressure >140 mmgHG was observed in 30%, serum glucose >110 mg/dL in 13%, insulin resistance in 23%, total cholesterol >200 mg/dL in 30%, LDL cholesterol >130 mg/dL in 31%, HDL cholesterol <35 mg/dL in 29% and triglycerides (TG) >200 mg/dL in 22%. Patients having moderate or severe fat accumulation showed increased values of these parameters. Thus, overall, 40% fulfilled the ATP III criteria for metabolic syndrome (ranging from 23% in absence of LD, 32% mild, 46% moderate to 71% in case of previous severe LD), a similar presentation to that observed with the IDF definition (36% of MS; ranging from 20% in absence to 69% in severe) and higher than that of AHA (overall, 19%; ranging from 8%, 5%, 21% to 45%).

**Conclusions**: The presence of fat accumulation and its severity is associated with increased incidence of different cardiovascular risk factors and progressive appearance of “iatrogenic” secondary metabolic syndrome.

# Sub article: P204 Normalisation of undernutrition following initiation of HAART is not associated with future diabetes risk

**Published:** 2016-10-23

**Authors:** Jonathan Mok, Louise Goff, Barry Peters, Alastair Duncan

**Affiliations:** 1 School of Medical Education, King's College London, London, UK; 2 Division of Diabetes and Nutritional Sciences, King's College London, London, UK; 3 Harrison Wing, Guy's and St Thomas’ Hospital NHS Foundation Trust, London, UK

## Abstract

**Introduction**: HAART and HIV infection have been implicated in impairing glucose and lipid metabolism in people living with HIV. Increases in body mass index (BMI) following HAART initiation have been well documented in the literature. We aimed to investigate the association between BMI status at HAART initiation and future risk of developing type 2 diabetes.

**Methods and materials**: A wide range of clinical factors were measured and recorded in a representative HIV-positive patient sample attending three London outpatient clinics. BMI was calculated prior to commencing HAART and 1 year after initiating therapy and were classified according to World Health Organization international criteria (underweight ≤18.5 kg/m^2^, normal 18.5–24.9 and overweight 25.0–29.9, obese ≥30.0). Glycaemic status was classified by fasting glucose as normal or dysglycaemia (<6.0 and ≥6.0 mmol/L respectively). Univariate statistical analysis and binary logistic regression were used to estimate contributions to risk of dysglycaemia.

**Results**: Binary logistic regression suggests that BMI percentage change in the first year post-HAART was a significant predictor of dysglycaemia with relative risk (RR) of 6.6% for each percentage increase in BMI (RR 1.066; 95% CI 1.031–1.101; p<0.001) (Table 1). Dysglycaemia risk increased by 13% for each percentage increase in BMI (RR 1.131; 95% CI 1.068–1.198; p<0.001) for normal weight subjects and by 41% for obese patients (RR 1.408; 95% CI 1.069–1.853; p=0.015). Weight gain in those patients with a BMI below 18.5 kg/m^2^ was not associated with future diabetes risk. The type of HAART used in the first year of treatment was not significantly associated with future dysglycaemia.

**Conclusion**: Normalisation of undernutrition in the year following initiation of HAART is not associated with future diabetes risk. Prevention of excessive weight gain following initiation of HAART should be a priority in those with a BMI greater than 18.5 kg/m^2^.

**Table 1:** Percentage change in BMI after 1 year of HAART, stratified by pre-HAART BMI status

BMI status pre-HAART	n (cohort%)	BMI change 1 year post-HAART initiation (%)	95% CI	p
Underweight	19 (6)	19.2	9.3, 31.3	<0.001
Normal weight	171 (56)	4.7	3.6, 5.8	<0.001
Overweight	80 (26)	3.3	1.5, 5.1	<0.001
Obese	36 (12)	1.6	−1.2, 4.1	0.221

# Sub article: P205 Hepatic steatosis is highly prevalent in HIV and significantly associated with diabetes risk

**Published:** 2016-10-23

**Authors:** Jonathan Mok, Louise Goff, Barry Peters, Alastair Duncan

**Affiliations:** 1 School of Medical Education, King's College London, London, UK; 2 Division of Diabetes and Nutritional Sciences, King's College London, London, UK; 3 Harrison Wing, Guy's and St Thomas’ Hospital NHS Foundation Trust, London, UK

## Abstract

**Introduction**: HIV infection and antiretroviral therapy have been implicated in mediating metabolic derangement. Hepatic steatosis has been associated with the development of dysglycaemia in HIV-negative cohorts; however, the impact of HIV and concordant factors has been relatively understudied. We aimed to investigate the prevalence of hepatic steatosis in an urban HIV cohort and assess its association with other factors.

**Methods and materials**: As part of the NIHR-funded STOP Diabetes in HIV cross-sectional study, a range of clinical information was collected from a sample structured to statistically represent HIV-positive patients attending large South London clinics. Hepatic steatosis was diagnosed by biopsy or FibroScan. Glycaemic status was classified by fasting glucose as normal or dysglycaemia (<6.0 and ≥6.0 mmol/L, respectively). Univariate statistical analysis and binary logistic regression were used to estimate risk factors for hepatic steatosis, and contributions to risk of dysglycaemia.

**Results**: Hepatic steatosis was present in 21% (n=71) of the total cohort (n=339). There was a significant correlation between hepatic steatosis and dysglycaemia (Pearson's Chi-squared p<0.001). For those with hepatic steatosis, the odds ratio (OR) of developing the condition is 10.08 (95% CI 5.53–18.40; p<0.001). Significant ORs were found for nucleoside reverse transcriptase inhibitors traditionally associated with metabolic dysfunction: zidovudine, stavudine, didanosine and zalcitabine (OR 20.339; 95% CI 1.144–361.611; p=0.040). Other significant factors included statin therapy (OR 3.313; 95% CI 1.767–6.211; p<0.001), overweight (OR 0.320; 95% CI 0.134–0.767; p=0.011) and obesity (OR 0.073; 95% CI 0.031–0.174; p<0.001).

**Conclusion**: Hepatic steatosis is significantly correlated with diabetes risk in people living with HIV. Factors that are implicated in mediating this metabolic derangement include the use of nucleoside reverse transcriptase inhibitors and statin therapies, and BMI. The diagnosis and subsequent treatment of hepatic steatosis in HIV patients is key.

# Sub article: P206 HIV-positive inflammatory activity monitoring correlated to insulin resistance: HIRE study

**Published:** 2016-10-23

**Authors:** Melissa Soares Medeiros, Henrique Pires Moreira, Debora Veras Da Ponte, Ana Carolina dos Santos Araujo, Andre Pereira de Brito Neves, Rebecca Santos Souza, Huylmer Lucena Chaves, Morgana Feitosa de Queiroga, Vinicius Ximenes Paula, Erico Antonio Gomes Arruda

**Affiliations:** 1 Infectious Diseases, Unichristus University/Hospital Sao Jose, Fortaleza, Brazil; 2 Infectious Diseases, Unichristus University, Fortaleza, Brazil; 3 Infectious Diseases, UECE University/Hospital Sao Jose, Fortaleza, Brazil

## Abstract

**Introduction**: Insulin resistance and diabetes mellitus are important metabolic complications of HIV-infected patients’ therapy, since an increased survival occurred after HAART [1,2]. HIV-infected patients have an increased risk of hyperglycaemia associated with inflammatory activity and medications, and this can implicate directly in survival and life quality [3]. Inflammatory status related to these patients can also be responsible for increases risk of hospitalization and bad prognosis [4].

**Materials and methods**: This study was a retrospective analysis of a multicentre cohort proposed to evaluate impact and risk factors for insulin resistance in HIV outpatients of Unichristus Center University and Hospital Geral de Fortaleza, including sociodemographic issues, hospitalization data, comorbidities and laboratory data.

**Results**: A total of 218 patients were included, 73.9% male, median age of 37 years, median HIV diagnosis of 24 months and median follow-up period of 21 months. CD4/CD8 rate before ART 0.38±0.29 and final 0.62±0.4, initial CD4 count mean 400 cells/mm^3^ and final 570 cells/mm^3^, 97.3% had suppressed viral load in final visit. Only 2.8% of patients had diabetes mellitus before HIV diagnosis. There was a significant increase in glucose levels after HAART initiation (18.5% vs. 36.7%, p=0.0025). Fasting glucose elevation was detected as a risk factor to develop symptoms during follow-up (RR 1.35; 95% CI 1.01–1.80; p=0.002). A higher monocyte/lymphocyte ratio was associated with hospitalization during the follow-up before (p=0.011) and after (p=0.033) introduction of ART. After introduction of HAART, there was an increase in Castelli index for hyperglycaemic patients, but significant difference did not remain during follow-up. Castelli index was 4.5±1.2 before ART, 4.8±1.4 after 12 months, 5.4±1.8 after 24 months and 5.3±1.8 after 36 months.

**Conclusion**: Antiretroviral therapy is an important factor associated with higher glucose levels, and causes insulin resistance associated with uncontrolled lipid levels. Perhaps, HIV treatment is essential to control chronic inflammation and its consequences. Monocyte/lymphocyte ratio can be an easy marker for inflammation activation monitoring and could be associated with higher risk for hospitalization.

## References

[1] Galescu O, Bhangoo A, Ten S (2013). Insulin resistance, lipodystrophy and cardiometabolic syndrome in HIV/AIDS. Rev Endocr Metab Disord.

[2] Kagaruki GB, Kimaro GD, Mweya CN, Kilale AM, Mrisho RM, Shao AF (2015). Prevalence and risk factors of metabolic syndrome among individuals living with HIV and receiving antiretroviral treatment in Tanzania. Br J Med Med Res.

[3] Brown TT, Tassiopoulos K, Bosch RJ, Shikuma C, McComsey GA (2010). Association between systemic inflammation and incident diabetes in HIV-infected patients after initiation of antiretroviral therapy. Diabetes Care.

[4] Shen Y, Wang Z, Liu L, Zhang R, Zheng Y, Lu H (2013). Prevalence of hyperglycaemia among adults with newly diagnosed HIV/AIDS in China. BMC Infect Dis.

# Sub article: P207 A cross-sectional study of comorbidities in HIV-infected patients receiving cART in Taiwan: a nationwide surveillance

**Published:** 2016-10-23

**Authors:** Chia-Jui Yang, Hsiu-Yin Wang, Tse-Chih Chou, Chee-Jen Chang

**Affiliations:** 1 Department of Internal Medicine, Far Eastern Memorial Hospital, New Taipei City, Taiwan; 2 Janssen, Medical Affair, Taipei, Taiwan; 3 Clinical Informatics and Medical Statistics, Chang Gung University, Taoyuan, Taiwan

## Abstract

**Introduction**: HIV-infected individuals may be at increased risks of age-associated non-communicable comorbidities during use of cART. We conducted a nationwide surveillance for the prevalence of non-communicable comorbidities among HIV-infected patients receiving cART.

**Materials and methods**: Comorbidity data from 2010 to 2013 were obtained from the Taiwan National Health Insurance Research Database while 20,726 HIV-infected patients were identified. Non-communicable comorbidities are defined as type II diabetes mellitus (DM), hypertension, dyslipidaemia, acute coronary syndrome (ACS) and cholelithiasis or nephrolithiasis.

**Results**: Among 20,726 HIV-infected patients in Taiwan, 13,142 of them receiving antiretroviral therapy were included in the analysis. Mean age of the 13,142 patients was 36.6 while 34.1% of them are older than 40 years and most are male (93.6%). The annual number of subjects newly on cART increased from 1819 to 3418 during study period. In the newly on cART population, around 70% were aged between 20 and 39 years and the majority were male (93%). The prevalences of comorbidities in the total study population were type II DM 7.3%, hypertension 33.6%, dyslipidaemia 24.0%, major depressive disorder 21.2%, use of sedative drug 39.5%, ACS 0.5% and cholelithiasis or nephrolithiasis 5.5%. In addition, the prevalence increased sharply after age 40, especially for metabolic comorbidities (type II DM: 15.0% vs. 3.3%; hypertension: 46.7% vs. 26.8%; dyslipidaemia: 34.9% vs. 18.4%; ACS: 1.2% vs. 0.2%; cholelithiasis or nephrolithiasis: 7.3% vs. 4.6%). In the study population, 13.2% of patients had more than two concomitant comorbidities and that prevalence also increased sharply after 40 years old (24.8% vs. 7.2%).

**Conclusion**: According to our nation-wide surveillance between 2010 and 2013, comorbidities among HIV-infected patients receiving cART in Taiwan demonstrated high prevalence in patients aged 40 years or older.

# Sub article: P208 Tryptophan metabolism and its relationship with central nervous system toxicity in subjects switching from efavirenz to dolutegravir

**Published:** 2016-10-23

**Authors:** Michael Keegan, Alan Winston, Chris Higgs, Dietmar Fuchs, Adriano Boasso, Mark Nelson

**Affiliations:** 1 HIV Research Unit, Clinical Trials Centre, Imperial College London/ViiV Healthcare Ltd., London, UK; 2 HIV Research Unit, Clinical Trials Centre, Imperial College London, London, UK; 3 Chelsea & Westminster Hospital, St Stephen's AIDS Trust, London, UK; 4 Centre for Chemistry and Biochemistry, Innsbruck, Austria; 5 Centre for Immunology & Vaccinology, Chelsea & Westminster Hospital, London, UK

## Abstract

**Introduction**: The pathogenesis of central nervous system (CNS) toxicities observed in antiretroviral treated (ART) persons living with HIV (PLWH) remains elusive. We investigated the associations between indoleamine 2,3-dioxygenase-1 (IDO-1) activity, via kynurenine/tryptophan (KYN/TRP) ratios, and measurements of CNS toxicity in PLWH switching from efavirenz (EFV) to dolutegravir (DTG).

**Materials and Methods**: In a prospective, randomised, open-label, multi-centre study, virologically-suppressed PLWH receiving an EFV-containing regimen for >12 weeks with ongoing CNS toxicity were switched to DTG and followed-up for 12 weeks. Plasma neopterin, TRP and KYN concentrations were measured and the KYN/TRP ratio calculated. Rates of CNS toxicities were measured using a questionnaire based on the EFV label and graded according to the ACTG adverse events scale. They included dizziness, depression, insomnia, anxiety, confusion, impaired concentration, headache, somnolence, aggression and abnormal dreams. Scores ranged from 0 (none) to 3 (severe) and were summed, giving a total score ranging from 0 to 30. CNS toxicity measurements also included assessment using the Instrumental Activities of Daily Living (IADL) and Hospital Anxiety & Depression (HAD) scales. Univariate (paired-samples t-tests) and linear mixed model analyses were conducted.

**Results**: The majority of subjects were male (95%) and White (95%). Mean age was 47.8 years. The mean plasma concentration of KYN significantly increased from baseline to week 12 (2.12 to 2.49 µmol/L, p=0.002). A non-significant increase was observed for the KYN/TRP ratio (39.7 to 44.8 µmol/mmol, p=0.012). Significant reductions in mean CNS toxicity score (10.1 to 4.5, p<0.001) and HAD score (14.1 to 8.4, p<0.001) were observed from baseline to week 12. Mean IADL scores did not change significantly (7.8 to 7.90, p<0.570). In the linear mixed model analyses, plasma KYN concentrations and KYN/TRP ratios were found to be statistically, significantly, negatively correlated with CNS toxicity scores. For every 1 μmol/L increase observed in KYN concentration, a 1.7 point decrease was observed in the CNS toxicity score (Table 1). Likewise, for every 1 μmol/mmol increase observed in the KYN/TRP ratio, a 0.1 point decrease was observed in the CNS toxicity score. No significant relationship was observed for KYN or KYN/TRP ratios and HAD scores.

**Conclusions**: Switching from EFV to DTG was associated with improvements in CNS toxicity and HAD scores, and increases in plasma KYN concentrations. Increases in plasma KYN concentrations and the KYN/TRP ratio correlated with decreases in CNS toxicity. Underlying mechanisms need to be established and may include EFV-induced changes in concentrations of hepatic reactive oxygen species and CNS inflammatory processes.

**Abstract P208–Table 1:** Linear mixed model results for KYN and KYN/TRP ratio and CNS toxicity from baseline to week 12

Parameter	Estimate (95% CI)	t(df)	P	−2 Log Likelihood for Model
Model 1: CNS Toxicity and KYN				
Mean CNS Toxicity Score	10.4 (7.0 to 13.9)	6.0 (104)	<0.001	685.2
KYN, µmol/L	−1.7 (−3.1 to −0.3)	−2.4 (111)	0.019	
				
Model 2: CNS Toxicity and KYN/TRP Ratio				
Mean CNS Toxicity Score	10.4 (6.8 to 14.1)	5.7 (83)	<0.001	690.4
KYN/TRP Ratio, µmol/mmol	−0.1 (−0.2 to −0.0)	−2.3 (89)	0.027	

# Sub article: P209 Multicentre open-label pilot study of switching from efavirenz to dolutegravir for central nervous system (CNS) toxicity

**Published:** 2016-10-23

**Authors:** Margherita Bracchi, Nicole Pagani, Amanda Clarke, Tanya Adams, Laura Waters, Matthew Bolton, Alan Winston, Borja Mora-Peris, Laura Dickinson, Marta Boffito, Mark Nelson

**Affiliations:** 1 Chelsea and Westminster Hospital, St Stephen's AIDS Trust, London, UK; 2 HIV/GUM, Royal Sussex County Hospital, Brighton, UK; 3 HIV/GUM, Mortimer Market Centre, London, UK; 4 HIV/GUM, St Mary's Hospital, London, UK; 5 Molecular & Clinical Pharmacology, University of Liverpool, Liverpool, UK; 6 HIV/GUM, Chelsea and Westminster Hospital, London, UK

## Abstract

**Introduction**: Efavirenz (EFV) remains a widely used third agent with increasing numbers of patients switching due to CNS toxicities. Data on improvement of CNS toxicities after switch to dolutegravir (DTG) are scarce. We investigated substitution of EFV for DTG, in combination with two NRTIs, in patients with ongoing EFV-associated CNS side effects.

**Methods**: A randomized open-label multicentre study of virologically suppressed patients receiving an EFV-containing regimen for at least 12 weeks. Randomization was to immediate (IS) versus delayed switch (DS) (after 4 weeks) to DTG, without backbone change. Primary endpoint was rate of CNS toxicity (CNS score) at 4 weeks in the IS versus DS arms, measured by a questionnaire based on EFV SPC and graded according to ACTG adverse event scale. Secondary endpoints were: rate of CNS toxicity at 12 weeks post-switch, change in sleep, quality of life, neurocognitive function, CD4 count, fasting lipids, maintenance of virological suppression post-switch and DTG PK post-switch.

**Results**: Forty patients (38 males), mean age 48 years (range 27–67) were enrolled: 19 IS and 21 DS arms. Median CD4 was 544/601 cells/µ in IS/DS, respectively. Baseline CNS score was similar in both arms (IS 33, DS 40). There was a significant improvement in CNS score at week 4 in the IS arm versus DS arm (p<0.001). Combined (both arms) improvement in total CNS score, 4 weeks post-switch was significant (p<0.001) and maintained during the study period (p<0.001). Reduction in abnormal dreams (p<0.001), dizziness and depression (p=0.008) at weeks 4 and 12 post-switch were observed (Table 1). Significant improvement in hospital anxiety and depression scale (HADS), quality of life (EQ-5D) and quality of sleep (Pittsburgh Sleep Score) was found at weeks 4 and 12 post-switch. All subjects completed the study and maintained virological suppression without changes in CD4 cell count/% or adherence. Better lipid profile with changes (mmol/L) in total cholesterol, LDL and triglycerides at weeks 4 and 12 post-switch was observed (mean change at week 12: cholesterol −0.8, p<0.001; LDL −0.38, p<0.001; triglycerides −0.24, p<0.001). Geometric mean (95% CI) of DTG plasma concentrations 24 hours post-dose (predicted by population PK modelling) was 862.8 ng/mL (848.9–962.2 ng/mL). Concentrations increased over the 4 weeks post-switch and all remained above DTG IC90 for WT virus (64 ng/mL).

**Conclusions**: Switching EFV to DTG was associated with significant improvement in CNS toxicity, with a reduction in overall CNS score and improvement in depression, dizziness and quality of sleep, without affecting antiretroviral efficacy.

**Abstract P209–Table 1:** Overall CNS score at week 4 (IS vs. DS) and CNS grade 3/4 side effects (S/E) at 4 and 12 weeks post-switch in IS and DS arms

	IS (N19)	DS (N21)	IS (N19)	DS (N21)		IS (N21)	DS (N19)	IS (N21)	DS (N19)
					
	BL		W4		p	W4 post-switch		W12 post-switch	
Overall CNS score (0–100), median (IQR)	33 (20–53)	40 (27–53)	10 (7–20)	33 (20–43)	<0.001	10 (7–20)	10 (3–23)	10 (3–17)	10 (3–23)
						% of improvement:			
						16% (4–36)	13% (10–26)	17% (4–33)	16% (10–33)
						p < 0.001	p < 0.001	p < 0.001	p < 0.001
Proportion (%) of patients with:						Proportion (%) of patients with grade 3/4 S/E:			
Overall grade 3/4 toxicity	100	95.2	26.3	95.2	<0.001	26.3 (p < 0.001)	47.6 (p < 0.001)	26.3 (p < 0.001)	42.9 (p < 0.001)
Insomnia	47.4	61.9	21.1	42.9	0.258	21.1 (p = 0.025)	28.6 (p = 0.317)	15.8 (p = 0.014)	19.1 (p = 0.317)
Abnormal dreams	84.2	85.7	5.3	81	<0.001	5.3 (p < 0.001)	14.3 (p < 0.001)	0 (p < 0.001)	19.1 (p < 0.001)
Dizziness	21.1	28.6	0	28.6	0.037	0 (–)	0 (–)	0 (–)	0 (–)
Depression	42.1	38.1	5.3	38.1	0.035	5.3 (p = 0.008)	4.8 (p = 0.008)	5.3 (p = 0.008)	19.1 (p = 0.046)
Anxiety	36.8	52.4	10.5	19.1	0.756	10.5 (p = 0.059)	4.8 (p = 0.180)	5.3 (p = 0.014)	14.3 (p = 0.564)
Confusion	15.8	0	0	0	–	0 (–)	0 (–)	5.3 (p = 0.157)	4.8 (–)
Impaired concentration	26.3	28.6	10.5	19.1	0.756	10.5 (p = 0.180)	14.3 (p = 0.564)	5.3 (p = 0.046)	9.5 (p = 0.317)
Headache	0	19.1	0	19.1	0.140	0 (–)	0 (–)	0 (–)	4.8 (p = 0.083)
Somnolence	21.1	38.1	5.3	28.6	0.128	5.3 (p = 0.180)	14.3 (p = 0.083)	10.5 (p = 0.317)	14.3 (p = 0.083)
Aggressive behaviour	10.5	23.8	5.3	19.1	0.402	5.3 (p = 0.564)	4.8 (p = 0.083)	5.3 (p = 0.317)	4.8 (p = 0.083)

BL, baseline; DS, delayed switch; IQR, inter-quartile range; IS, immediate switch; S/E, side effect; W4, week 4; W12, week 12.

# Sub article: P210 Psychiatric adverse events from the DTG ART-naïve phase 3 clinical trials

**Published:** 2016-10-23

**Authors:** Romina Quercia, Jeremy Roberts, Andrew Murungi, Lloyd Curtis, Nassrin Payvandy, Justin Koteff, Michael Aboud

**Affiliations:** 1 Research and Development, ViiV Healthcare, Brentford, UK; 2 Statistics, ViiV Healthcare, Mississauga, Canada; 3 Medical Affairs, ViiV Healthcare, Brentford, UK; 4 Pharmacovigilance, GlaxoSmithKline, Stockley Park, UK; 5 Pharmacovigilance, ViiV Healthcare, Brentford, UK; 6 Medical Affairs, ViiV Healthcare, Research Triangle Park, North Carolina, USA

## Abstract

**Introduction**: To provide characterization of select psychiatric adverse events (pAEs), including anxiety, depression (including: depression, bipolar, suicidal ideations and hypomania), insomnia and nightmares/abnormal dreams reported in the dolutegravir (DTG) phase 3/3b treatment-naïve clinical trials.

**Materials and Methods**: Safety data of pAEs from phase 3/3b trials in ART-naïve adults were analysed. Data at 96 weeks for SPRING-2, SINGLE, FLAMINGO and at 48 weeks for the all-women study, ARIA, were analysed. Frequencies of pAEs were summarized for DTG and the comparator drug by study.

**Results**: There were 2634 subjects analysed in the four clinical studies including 1315 patients treated with DTG. Safety summaries showed a low number of pAEs across all study treatment arms, with the majority of these being low grade (1–2). The rates of pAEs leading to withdrawals were low across all trials (<5% for each individual analysis). Anxiety led to four discontinuations with EFV in SINGLE. Depression led to one discontinuation with DTG in SINGLE, two with RAL in SPRING-2 and seven patients on EFV in SINGLE. Insomnia led to two DTG discontinuations, one each in SINGLE and ARIA respectively, and three EFV patients in SINGLE. Additionally in SINGLE, two DTG and seven EFV patients discontinued because of nightmares/abnormal dreams. There was higher pAE reporting within SINGLE that was inconsistent with the other studies. The rates of anxiety, insomnia, depression and nightmares/abnormal dreams, in the DTG and comparators arms across the four phase 3/3b clinical trials, are outlined in Figure 1.

**Conclusions**: In the four treatment-naïve clinical trials, DTG once daily was well tolerated with a low rate of pAEs. The inconsistency seen in the SINGLE study may be partially explained by study design bias; a double-blind study versus efavirenz and the use of the HIV Symptom Index Distress Module. The majority of all pAE cases were low grade, and few led to discontinuations.

**Abstract P210–Figure 1:** Psychiatric Adverse Events rates from the DTG ART-Naïve Phase III Clinical Trials.

# Sub article: P211 Prevalence of undiagnosed neurocognitive impairment in HIV-infected adults taking efavirenz on a long-term basis with undetectable or low HIV RNA compared with protease inhibitors

**Published:** 2016-10-23

**Authors:** Nuno Marques, Catarina Fernandes, Sandra Tejo, Ana Cristina Rosa, Maria Emília Nogueira, Sónia Rocha, João Trêpa, José Saraiva da Cunha

**Affiliations:** 1 Infectious Diseases, Hospital Garcia de Orta, EPE, Almada, Portugal; 2 Infectious Diseases, Centro Hospitalar e Universitário de Coimbra, EPE, Coimbra, Portugal; 3 Department of Mathematics, Faculty of Sciences and Technology, University of Coimbra, Coimbra, Portugal

## Abstract

**Introduction**: Neurocognitive impairment remains a major issue in HIV infection. The main objective was to assess the prevalence of HIV-associated neurocognitive disorders (HAND) according to the Frascati criteria in asymptomatic HIV-infected adults on stable and long-term cART regimen containing EFV versus a protease inhibitor (PI) regimen.

**Materials and methods**: Cross-sectional comparative study of HIV-infected adults on cART containing EFV or a PI (DRV/r; ATV/r or LPV/r) with undetectable or low (<400 copies/mL) viral load for over 6 months and on the same regimen for at least the past 12 months. Exclusion criteria were pregnancy; previous diagnosis of: dementia of any cause; major opportunistic infection of the brain in the past 3 years before study entry; history of ischemic or haemorrhagic stroke; history of untreated and/or symptomatic syphilis; current major psychiatric/neurologic disorders according to the opinion of the investigators; current illicit drug-use disorder and/or alcohol abuse. Patients were clinical evaluated with neurologic exam, CD4+ count, HIV viral load, urine drug screen and syphilis serology. Psychological testing included International HIV Dementia Scale (IHDS), Beck Depression Inventory, The Lawton Instrumental Activities of Daily Living and an adaption of the questionnaire to assess adherence to antiretroviral treatment – HIV (CEAT-VIH). Additional testing was performed in case of IHDS score <10. Statistical analysis was performed using SPSS version 22 and SAS-JMP version 12 for Firth bias-adjustment in logistic regression.

**Results**: A total of 314 patients (sample size with power of 80% and level of significance of 5%), 157 on EFV and 157 on PI were included. HAND was not associated with EFV or PI regimens (p=0.359). Its prevalence was 18.5% (n=29) on EFV and 14% (n=22) on PI group. Baseline characteristics are shown in Table 1. In the univariate analysis, the variables associated with HAND were: mental status abnormalities such as memory (p<0.001), abstraction, judgment and mood (p<0.001) and calculation abilities (p=0.015); abnormalities in the casual gait (p=0.032), heel-to-toe gait (p<0.001) and toes walking (p=0.007); dysdiadochokinesis (p=0.002); diabetes (p=0.025); dyslipidaemia (p=0.043); hypertension (p=0.048); anaemia (p=0.028), report of adverse events on CEAT-VIH (p=0.05); educational level, such as illiteracy (p<0.001) and primary education (p<0.01) and older age (p<0.001).

**Conclusions**: Abnormalities in the neurologic exam, metabolic comorbidities, anaemia, older age and education level were associated with HAND.

**Acknowledgements**: This work has been supported by an unrestricted grant from Gilead Sciences Europe Ltd.

**Abstract P211–Table 1:** Baseline characteristics

Baseline characteristics	EFV group (n = 157)	PI group (n = 157) [DRV/r: 66; ATV/r: 63; LPV/r: 28]	p
Age (mean±SD)	48.82 (±12.12)	48.74 (±11.35)	0.95
Gender			0.892
Male	121 (77.1%)	123 (78.3%)	
Female	36 (22.9%)	34 (21.7%)	
Body mass index (mean±SD)	24.47 (±4.27)	25.51 (±4.33)	0.112
Educational level			0.446
Illiteracy	4 (2.5%)	4 (2.5%)	
Basic 1st cycle	33 (21%)	49 (31.2%)	
Basic 2nd cycle	24 (15.3%)	19 (12.1%)	
Basic 3rd cycle	45 (28.7%)	42 (26.8%)	
Secondary	36 (22.9%)	28 (17.8%)	
Higher education	15 (9.6%)	15 (9.6%)	
HIV transmission categories			>0.999
Heterosexual	89 (56.7%)	88 (56%)	
Men who have sex with men	33 (21%)	34 (21.7%)	
Blood transfusion	1 (0.6%)	0 (0%)	
Injection drug use	34 (21.7%)	35 (22.3%)	
Years of HIV infection (mean±SD)	10.73 (±5.37)	9.95 (±6.01)	0.224
Years on current cART (mean±SD)	5.36 (±2.4)	4.27 (±2.34)	<0.001
Years on antiretroviral therapy (mean±SD)	7.98 (±4.59)	7.18 (±4.75)	0.098
Years of HIV viral suppression (mean±SD)	7.21 (±4.21)	5.34 (±3.56)	<0.001
AIDS			<0.001
History of AIDS	21 (13.4%)	49 (31.2%)	
No history of AIDS	136 (86.6%)	108 (68.8%)	
Nadir CD4 +	243.85 (±136.56)	177.24 (±150.58)	<0.001
CD4+ count at the time of study entry	690.27 (±285.89)	589.77 (±287.32)	0.002
CPE, antiretroviral CSF penetration-effectiveness			<0.001
CPE of 6	0 (0%)	47 (29.9%)	
CPE of 7	135 (86%)	84 (53.5%)	
CPE of 8	21 (13.4%)	23 (14.7%)	
CPE of 9	1 (0.6%)	3 (1.9%)	
Syphilis			0.019
History of syphilis	37 (23.6%)	57 (36.3%)	
No history of syphilis	120 (76.4%)	100 (63.7%)	
Erectile dysfunction			0.036
Presence of erectile dysfunction	1 (0.6%)	8 (5.1%)	
No erectile dysfunction	156 (99.4%)	149 (94.9%)	
Antidepressant use			0.1
No antidepressant use	141 (89.8%)	130 (82.8%)	
Antidepressant use	16 (10.2%)	27 (17.2%)	
HCV co-infection			0.599
No HCV co-infection	112 (73.2%)	115 (76.2%)	
HCV co-infection	41 (26.8%)	36 (23.8%)	
ABC/3TC backbone	21 (13.4%)	25 (15.9%)	
AZT/3TC backbone	1 (0.6%)	9 (5.7%)	
FTC/TDF backbone	135 (86%)	121 (77.1%)	
AZT + TDF backbone	0 (0%)	2 (1.3%)	

# Sub article: P212 Adverse events and discontinuation of dolutegravir-based therapy in naïve and experienced HIV patients: tertiary HIV centre experience

**Published:** 2016-10-23

**Authors:** Cristina Fernandez, Kirsten Michie, Rebecca Thomson-Glover, Mas Chaponda, Libby Ratcliffe

**Affiliations:** 1 Infectious Diseases Department, Royal Liverpool Hospital, Liverpool, UK; 2 GUM Department, Royal Liverpool Hospital, Liverpool, UK

## Abstract

**Introduction:** Dolutegravir was first available for use on a compassionate basis in the UK in 2013 for the treatment of HIV and was then licensed in 2014. It is now recommended as one of the preferred third agents in the national British HIV Association guidelines [1]. Despite dolutegravir's perception as a well-tolerated antiretroviral, its SPC describes psychiatric side effects including insomnia, abnormal dreams and depression as common (incidence 1–10%) and headaches as very common (incidence >10%) [2]. This study looked at real-world data of dolutegravir tolerability and switch in our UK tertiary centre that manages over 1500 HIV-positive patients.

**Materials and methods**: A retrospective case review of all patients who received dolutegravir-containing antiretroviral regimens was conducted, both as fixed-dose combination (Triumeq) and dolutegravir single tablet. Data were collected from when dolutegravir was first prescribed in our centre (June 2013) until June 2016. Information regarding patient demographics, previous experience to ART, documented side effects and switch was collected from HIV patient records.

**Results**: Hundred and seventy-eight patients have received dolutegravir-containing regimens in our centre, 126/178 (71%) were treatment-experienced patients and 52/178 (29%) naïve patients. Table 1 shows patient demographics; they are predominantly Caucasian males, with ART-naïve patients being on average 10 years younger than their ART-experienced counterparts. More ART-naïve patients commenced a fixed-dose combination tablet containing dolutegravir (Triumeq) compared to ART-experienced patients, 90% (47/52) compared to 69% (87/126).

In total, 59/178 (33%) patients starting a regimen containing dolutegravir experienced adverse events. Of these, 68% (40/59) were experienced patients. Table 2 shows the adverse events experienced. Despite 35 (20%) of all patients suffering severe CNS side effects (anxiety, depression, paranoia and personality change) only one patient suffered severe CNS disturbance with new suicidal ideation and self-harm. Only 10 patients (6%) had to stop their dolutegravir-containing regimen with eight (4%) of these stopping due to side effects.

**Conclusion**: In our cohort, the majority of patients starting dolutegravir-containing regimens were treatment experienced. ART-experienced patients also suffered the most adverse events, with CNS problems being the most common. Whilst up to one-third of patients experienced adverse events from their dolutegravir-containing regimen, very few patients have needed to stop their treatment. From our real-world data the incidence of CNS side effects is significantly greater than in its licensing studies and has an implication on the use of dolutegravir in the clinical setting.

**Table 1:** Patient demographics

Demographics	Naïve	Experienced	Total
Gender			
Male	47/52 (90%)	81/126 (64%)	128/178 (72%)
Female	5/52 (10%)	45/126 (36%)	50/178 (28%)
Ethnicity			
Caucasian	46/52 (88%)	92/126 (73%)	138/178 (78%)
African/Afro Caribbean	4/52 (8%)	32/126 (25%)	36/178 (20%)
Asian/other	2/52 (4%)	2/126 (2%)	4/178 (2%)
Age			
Median	34 years	43 years	40 years
Range	20–64 years	18–76 years	18–76 years

**Table 2:** Incidence of adverse events of patients in dolutegravir-containing regimens

Adverse event	Naïve (/52)	Experienced (/126)	Total (/178)
Central nervous system (CNS)	10 (19%)	25 (20%)	35 (20%)
Gastrointestinal	6 (12%)	11 (9%)	17 (10%)
Neurological	7 (13%)	5 (4%)	12 (7%)
Musculoskeletal	0 (0%)	6 (5%)	6 (3%)
Lethargy	1 (2%)	5 (5%)	6 (3%)
Dermatological	1 (2%)	3 (2%)	4 (2%)
Urogenital	0 (0%)	2 (2%)	2 (1%)

## References

[1] Waters L, Ahmed N, Angus B, Boffito M, Bower M, Churchill D British HIV Association guidelines for the treatment of HIV-1-positive adults with antiretroviral therapy 2015. 2016 interim update [Internet].

[2] ViiV Healthcare UK Ltd Summary of product characteristics Tivicay 50 mg film-coated tablets [Internet].

# Sub article: P213 Cerebrospinal fluid and plasma biomarkers in patients with HIV-associated neurocognitive disorders

**Published:** 2016-10-23

**Authors:** Mattia Trunfio, Daniela Vai, Alessandra Romito, Cristiana Atzori, Daniele Imperiale, Sabrina Audagnotto, Chiara Montrucchio, Silvia Scabini, Chiara Cardellino, Veronica Pirriatore, Giovanni Di Perri, Stefano Bonora, Andrea Calcagno

**Affiliations:** 1 Department of Medical Science, Amedeo di Savoia Hospital, University of Turin, Torino, Italy; 2 Unit of Neurology, Maria Vittoria Hospital, ASL TO2, Torino, Italy; 3 Laboratory of Immunology, Maria Vittoria Hospital, ASL TO2, Torino, Italy

## Abstract

**Introduction**: Biomarkers able to differentiate patients with HIV-associated neurocognitive disorders (HAND) are urgently needed for diagnosing and managing HIV-positive patients. Specifically infected astrocytes may be involved in the process since they are key elements in the neurovascular unit and blood brain barrier (BBB).

**Materials and methods**: Naïve and treated patients complaining of cognitive disturbances and undergoing complete cognitive tests (eight areas, diagnosis according to the Frascati criteria) and lumbar puncture (less than 6 months apart) were included; patients with opportunistic infections or neoplasms affecting the central nervous system were excluded. Immunovirological and therapeutic data as well as plasma S100Beta and CSF (tau, ptau, BAmil, neopterin, S100Beta, CSAR) biomarkers were recorded. Variables are described as medians (interquartile ranges) and analysed through non-parametric tests.

**Results**: Seventy-nine patients were included: 58 (73.4%) were male and 38 (48.1%) were on treatment. Median age, plasma and CSF HIV RNA and CD4+ T cell count were 47 years (43–56), 74,912 copies/mL (55–342,216), 1614 copies/mL (68–12,662) and 102 cells/mm3 (48–408). Thirty-six patients (45.5%) were diagnosed with HAND: 25 asymptomatic (31.6%), eight mild neurocognitive impairment (10.1%) and three dementias (3.8%). CSF tau, p-tau, BAmil, neopterin, S100Beta, plasma S100Beta and CSAR were 105 pg/mL (38–251), 33 pg/mL (21–44), 839.8 pg/mL (651–1156), 1.6 ng/mL (1–5.2), 150.5 pg/mL (88.6–275), 34 pg/mL (34–52.5) and 6 (4.1–7.68). CSF tau (234 pg/mL (87.9–357) vs. 62 pg/mL (37.5–206.4), p<0.001) and CSF S100Beta (227.3 pg/mL (111.7–299.6) vs. 129 pg/mL (76.8–220), p=0.025) were significantly higher in patients with HAND; CSF neopterin was borderline higher in patients with HAND (2.9 ng/mL (1.25–8.95) vs. 1.45 ng/mL (0.75–3.18), p=0.051). Plasma S100Beta was found to be mildly associated with CSAR (p=0.042, r=0.272). Furthermore, we observed significant correlations between CSF S100Beta, CSF neopterin and tau and specific neurocognitive tests (mostly in the areas of attention, verbal fluency, concentration and short-term memory), as shown in Table 1 underneath.

**Conclusions**: CSF tau and S100beta were significantly higher in patients with HAND. The former represents a marker of neuronal damage while the latter is produced by activated astrocytes, thus highlighting the potential role of these cells in the pathogenesis of HIV-associated neurological damage. Higher CSF markers were associated with worse performances in selected test in memory, attention and verbal fluency domains. Plasma S100Beta was associated with CSAR but this observation needs to be further confirmed in order to validate a peripheral marker of BBB impairment.

**Abstract P213–Table 1:** Correlations between CSF biomarkers and specific neurocognitive tests among HIV-positive patients

	Stroop test	Trail Making test AB	Corsi test	Serial Repetition of Disyllabic Words	Verbal Fluency	Free and Cue Selective Reminding test
CSF tau	p 0.05 rho 0.39	NS	p <0.01 rho –0.47	p 0.01 rho –0.35	p 0.03 rho –0.30	NS
CSF S100 β	NS	p 0.03 rho –0.29	p <0.01 rho –0.37	p 0.04 rho –0.24	NS	Delayed: p 0.04 rho –0.40 Immediate: p 0.03 rho –0.41
CSF neopterin	p 0.03 rho 0.36	NS	p 0.01 rho –0.31	NS	p 0.05 rho –0.26	NS

NS, non-significant. Spearman correlation test was used to make statistical analysis.

# Sub article: P214 Incidence of CSF HIV escape in patients on virologically suppressive second-line protease inhibitor-based ART in Pune, Western India

**Published:** 2016-10-23

**Authors:** Ameet Dravid, Chinmay Saraf, Milind Kulkarni, Sachin Kore, Niranjan Rathod, Uma Mahajan

**Affiliations:** 1 Department of HIV Medicine, Ruby Hall Clinic, Pune, India; 2 Pathology, Precision Diagnostics and Biosciences, Pune, India; 3 Department of HIV Medicine, Ashwini Sahakari Rugnalaya, Solapur, India; 4 Department of HIV Medicine, Apex Hospital, Kolhapur, India; 5 Biostatistics, Precision Diagnostics and Biosciences, Pune, India

## Abstract

**Introduction**: Incidence of neurosymptomatic CSF HIV escape in patients on suppressive protease inhibitor (PI)-based ART is inadequately studied in resource-limited settings (RLS) like India. Data on emergence of multidrug-resistant HIV in CNS are also rare.

**Methods**: HIV patients enrolled in cohort from February 2009 to 2016 and currently taking PI-based ART (two nucleoside reverse transcriptase inhibitors (NRTI) plus boosted PI or raltegravir plus boosted PI) for minimum 6 months with plasma viral load <1000 copies/mL were included. Those presenting with incident neurodeterioration were recorded as cases. Magnetic resonance imaging (MRI) and CSF study was done to establish diagnosis. Paired plasma and CSF viral load was done to diagnose CSF HIV escape. CSF escape was defined as CSF viral load >50 copies/mL while plasma load <50 copies/mL or CSF viral load 1 log higher than plasma viral load. CSF genotypic resistance testing (GRT) was performed in a subset of patients.

**Results**: Out of 1427 ART-experienced individuals (36% females), 322 were on PI-based suppressive ART. Median age was 40 years, median baseline CD4 count 161 cells/mm^3^ and median duration of suppressive ART 39 months. Seventeen patients developed CSF HIV escape and incident encephalopathy (incidence rate: 14.73 (95% CI 8.16–26.59) episodes per 1000 person-years). Median plasma and CSF viral load in patients were 170 and 2300 copies/mL. Median time to development of CSF escape was 33 months. CSF GRT was performed in 7/17 patients. Resistance mutations to lamivudine (M184V) and NNRTI were seen in all seven patients. Thymidine analogue mutations (TAMs) conferring NRTI cross resistance and major PI mutations (I50L, V82A) were seen in 5/7 patients each. In 10/17 therapy was changed only on basis of cerebral penetration effectiveness score (CPE) of ART. Eleven of 17 patients with CSF escape had plasma and CSF HIV viral load <50 copies/mL after change to neuroactive ART. There was one death due to CSF HIV escape. Use of tenofovir and atazanavir/ritonavir was associated with CSF escape while zidovudine protected against it. History of smoking (p=0.034) and CPE score <6 (p=0.031) were strongly associated with CSF escape.

**Conclusions**: Association of CSF escape with CPE score <6 further strengthens the case for using ART with better CNS penetration. CSF GRT shows emergence of multidrug-resistant CNS HIV requiring use of newer ARVs like darunavir, raltegravir and maraviroc which are sparsely available in RLS like India.

# Sub article: P215 Monitoring of 8-hydroxy-efavirenz concentrations for management of mood changes

**Published:** 2016-10-23

**Authors:** Nádia Grilo, Maria Correia, Catarina Sequeira, Shrika Harjivan, Umbelina Caixas, Lucília Diogo, Matilde Marques, Emília Monteiro, Alexandra Antunes, Sofia Pereira

**Affiliations:** 1 Centro de Estudos de Doenças Crónicas (CEDOC), NOVA Medical School/Faculdade de Ciências Médicas, Universidade Nova de Lisboa, Translational Pharmacology, Lisboa, Portugal; 2 Instituto Superior Técnico, Universidade de Lisboa, Centro de Química Estrutural (CQE), Lisboa, Portugal; 3 Centro de Estudos de Doenças Crónicas (CEDOC)/Centro Hospitalar de Lisboa Central, Translational Pharmacology, Lisboa, Portugal

## Abstract

**Introduction**: The major efavirenz metabolite, 8-hydroxy-efavirenz, has been reported as plausibly responsible for efavirenz-induced neurotoxic effects [1–3]. Notably, up to 35% of patients on efavirenz suffer from mood changes [4]. This work aimed to investigate 8-hydroxy-efavirenz as a determinant of mood changes and to evaluate the suitability of 8-hydroxy-efavirenz biomonitoring for the management of these manifestations.

**Materials and methods**: A case control study comparing the plasma concentrations of efavirenz, 8-hydroxy-efavirenz and 8-hydroxy-efavirenz-glucuronide was performed in two age-matched groups of HIV-infected male patients, one without adverse central nervous system complaints (control group, 28 patients) and the other presenting mood changes (study group, 14 patients). The following anthropometric and clinical data were gathered for each patient: age, time on efavirenz, antiretroviral comedication, time between blood sampling and last efavirenz dose intake, viral load, CD4+ cell count, alanine aminotransferase levels and self-reported symptoms of mood changes (anxiety, agitation, euphoria, mental confusion, paranoia, hallucinations and depression). The study protocol received prior approval from the Ethics Committee of Centro Hospitalar de Lisboa Central, EPE (115/2013). Patients gave their written informed consent in accordance with the Declaration of Helsinki and compliance was controlled by the clinician.

**Results**: There were no differences between the two groups regarding the recorded clinical and anthropometric parameters. The most noticed mood change was anxiety, in 71% of the patients. Non-conjugated 8-hydroxy-efavirenz plasma levels were higher in the study group, when compared to the control group (p=0.020). No differences were found for efavirenz or 8-hydroxy-efavirenz-glucuronide levels among groups. Efavirenz was directly associated with 8-hydroxy-efavirenz-glucuronide (Spearman r=0.414, p<0.010) within therapeutic efavirenz concentrations. However, for toxic plasma concentrations of the parent drug (>4 mg/L), this correlation was lost.

**Conclusion**: The biotransformation of efavirenz into 8-hydroxy-efavirenz has a role in efavirenz-related mood changes. The plasma concentration of this metabolite is a suitable parameter for therapeutic drug monitoring and mood changes management. Moreover, these data suggest that 8-hydroxy-efavirenz crosses the blood-brain barrier and that toxic concentrations of efavirenz might inhibit peripheral detoxification of 8-hydroxy-efavirenz via glucuronidation.

**Financial support**: Fundação para a Ciência e a Tecnologia (FCT, Portugal), through research grants UID/QUI/00100/2013, RECI/QEQ-MED/0330/2012, SFRH/BD/80690/2011; SFRH/BD/86791/2012; IF/01091/2013, and the LRI Innovative Science Award.

## References

[1] Aouri M, Barcelo C, Ternon B, Cavassini M, Anagnostopoulos A, Yerly S (2016). In vivo profiling and distribution of known and novel phase I and phase II metabolites of efavirenz in plasma, urine, and cerebrospinal fluid. Drug Metab Dispos.

[2] Brandmann M, Nehls U, Dringen R (2013). 8-Hydroxy-efavirenz, the primary metabolite of the antiretroviral drug efavirenz, stimulates the glycolytic flux in cultured rat astrocytes. Neurochem Res.

[3] Tovar-y-Romo LB, Bumpus NN, Pomerantz D, Avery LB, Sacktor N, McArthur JC (2012). Dendritic spine injury induced by the 8-hydroxy metabolite of efavirenz. J Pharmacol Exp Ther.

[4] Fumaz CR, Munoz-Moreno JA, Molto J, Negredo E, Ferrer MJ, Sirera G (2005). Long-term neuropsychiatric disorders on efavirenz-based approaches: quality of life, psychologic issues, and adherence. J Acquir Immune Defic Syndr.

# Sub article: P216 The predictive role of cerebrospinal fluid (CSF)/plasma immune activation and neuronal injury biomarkers for HIV-associated neurocognitive disorders (HAND) diagnosis: a cross-sectional study

**Published:** 2016-10-23

**Authors:** Carmela Pinnetti, Valentina Fedele, Pietro Balestra, Stefania Carta, Patrizia Lorenzini, Adriana Ammassari, Veronica Bordoni, Lucia Alba, Valentina Mazzotta, Susanna Grisetti, Federico Martini, Francesca Ceccherini-Silberstein, Carlo Federico Perno, Andrea Antinori

**Affiliations:** 1 Clinical Department, National Institute for Infectious Disease Lazzaro Spallanzani IRCCS, Rome, Italy; 2 Laboratory of Antiretroviral Therapy Monitoring, National Institute for Infectious Disease Lazzaro Spallanzani IRCCS, Rome, Italy; 3 Cellular Immunology and Pharmacology Laboratory, National Institute for Infectious Disease Lazzaro Spallanzani IRCCS, Rome, Italy; 4 Department of Experimental Medicine and Surgery, University of Rome Tor Vergata, Rome, Italy

## Abstract

**Introduction**: The diagnosis of HAND relies on neuropsychological assessment (NPA), often confounded by other conditions affecting cognitive performance. Moreover, these methods do not distinguish static and residual impairment from active and ongoing brain injury. For a more accurate clinical diagnosis, plasma and CSF biomarkers analysis was performed.

**Materials and methods**: Single-centre, cross-sectional analysis of immune activation (neopterin, sCD14) or neuronal injury (neurofilament light-chain protein, NFL) biomarkers by ELISA assay in CSF/plasma paired samples from HIV-positive patients well characterized for neurocognitive impairment (NCI) and HAND classification. All patients underwent lumbar puncture (LP) and received NPA in a period of 6 months before or after LP. A comprehensive tests battery (14 tests/five domains) was used to diagnose NCI. HAND were classified according to Frascati's criteria. Wilcoxon matched-pairs test was used.

**Results**: Fifty-four CSF/plasma pairs from as many patients included: 74% male, median age 47 years (IQR 40–51), heterosexual 31%, MSM 9%, IVDU 33%; 79% CDC C. Neurological signs/symptoms in 76%. Median current and nadir CD4 cells/mm^3^ was 251 (IQR 105–384) and 68 (IQR 24–118), respectively; 41 patients (76%) were on ARV, median plasma log10 HIV RNA was 2.5 (IQR 1.6–4.5) and CSF 2.2 (IQR 1.6–3.8). Undetectable HIV RNA in 30% of plasma and 32% of CSF sample. According to Frascati's criteria, eight patients (14.8%) resulted unimpaired, 30 patients had NCI (nine ANI 30%; 13 MND 43.3%; eight HAD 26.7%), and 16 patients (29.6%) showed a major confounder and were excluded from the analysis. CSF neopterin concentration increased with NCI worsening (unimpaired 164 ng/mL, ANI 124, MND 191, HAD 350, p for linear trend test=0.002) (Figure 1). Both NFL concentration in CSF (median in unimpaired 677 pg/mL, ANI 762, MND 814, HAD 1164, p at test for linear trend=0.025) and in plasma (unimpaired 1676 pg/mL, ANI 1719, MND 1830, HAD 2795, p for linear trend=0.033) increased by HAND occurrence and severity, and a significant difference was observed both for NFL concentration in CSF (p=0.036) and plasma (p=0.027) in pairwise comparison between unimpaired and HAD (Figure 1). No differences were found in plasma and CSF sCD14 by HAND.

**Conclusions**: NFL concentration both in CSF and in plasma seems to better discriminate patients with active neuronal injury with a good correlation with HAD stage. Instead, CSF neopterin was less sensitive to predict HAND. Mild NCI, remarkably ANI, was not sufficiently characterized by all biomarkers, due to presumably sub-clinical active CNS disease even in cognitively unimpaired individuals.

**Abstract P216–Figure 1:** Concentration of CSF NFL (log pg/mL), CSF neopterin (log ng/mL) and plasma NFL (log pg/mL) according to unimpaired results by neuropsychological assessment and HAND classification. ANI, asymptomatic neurocognitive impairment; HAD, HIV-associated dementia; MND, mild neurocognitive disorders.

# Sub article: P217 Neuro+3 study: cognitive evolution in HAND after 96 weeks of treatment intensification with higher CNS penetration score

**Published:** 2016-10-23

**Authors:** Gilles Force, Pierre De Truchis, Dhiba Marigot-Outtandy, Damien Le Du, Didier Troisvallets, Laurent Blum, Hocine Ait-Mohand, Idir Ghout, Valérie Hahn, Hélène Defferrière, Natacha Darchy, Claire Lecornu, Sandrine Brefort, Maud Larroze, Myriam Sauvage, Constance Delaugerre, Marie-Laure Néré, Nadia Mahjoub, Gilles Peytavin

**Affiliations:** 1 Internal Medicine, Institut Hospitalier Franco-Britannique, Levallois, France; 2 Infectious Disease, Raymond Poincare Hospital, Garches, France; 3 Bligny Hospital, Infectious Disease, Bligny, France; 4 Internal Medicine, Gonesse Hospital, Gonesse, France; 5 Internal Medicine, Pontoise Hospital, Pontoise, France; 6 Clinical Research Unit, Raymond Poincare Hospital, Garches, France; 7 Neurology, Sainte-Anne Hospital, Paris, France; 8 Neurology, Pontoise Hospital, Pontoise, France; 9 Virology, Saint-Louis Hospital, Paris, France; 10 Pharmacology, Bichat Hospital, Paris, France

## Abstract

**Introduction:** Neuro+3 is a pilot open-label study of ARV intensification in virologically controlled patients presenting HAND (HIV-associated neurocognitive disorders): ARV was changed for a new combination with CNS penetration effectiveness (CPE or CHARTER) score improved ≥+3 points and total CPE ≥9. The major endpoint is the evaluation of neurocognitive disorders after 48 and 96 weeks.

**Materials and methods:** Sixty-three patients were screened with BREF ≤15/18 or mHIVDS ≤10/12 in eight investigational centres. Thirty-one patients were included with at least two ability domains altered (>1 SD) for the following tests, after Beck Depression Inventory BDI II: Grooved Pegboard (d and nd), Verbal Fluency, CVLT, Digit span, PASAT, Digit symbol, Wisconsin Card Sorting Test (six domains). Raw test scores were converted to obtain a global deficit score (GDS) and each patient was classified into HAND levels (ANI, MND, HAD) using Cognitive Complaint Questionnaire (CCQ) score. Ultrasensitive HIV RNA and ARV drugs concentrations were performed at baseline and follow-up in plasma and CSF. Exclusion criteria were drug or alcohol abuse, positivity for HBsAg or HCV, hypothyroidism, vitamin B deficiency and psychiatric troubles. For CPE score, we considered only drugs without genotypic resistance.

**Results:** Median range characteristics of the 31 enrolled patients were: 26 men, 54 years (33–64), educational level of 11 years (5–17), HIV duration 20 years (2–29), undetectable plasma HIV RNA duration 7 years, baseline plasma HIV RNA 2 copies/mL (2 patients >20:26 and 40 copies/mL), baseline CSF HIV RNA <1 copy/mL (nine patients between 7 and 78 copies/mL), 29% of undetectable drugs in CSF, baseline CPE of 6 (3–8) with current ARV therapy, CPE after new combination of 10 (all score ≥9 except two patients). Treatment intensification was obtained with INSTI (64.5%), CCR5 inhibitor (32.3%) or NNRTI (19.4%). Median GDS was significatively reduced from 1.4 at baseline to 0.8 at week 48 and 1.0 at week 96, number of altered domains from four to three at week 48 and week 96, CCQ score from 4 to 2 at week 48 and 1 at week 96, BDI score from 14 to 8 at week 48 and 10 at week 96. At baseline, there were seven ANI, eight MND, and 16 HAD. At weeks 48 and 96, 16/31 patients (52%) and 19/31 (61%) were classified in an improving category of HAND. The evolution of CSF HIV RNA and drug concentrations are consistent with the drug combination used.

**Conclusions:** Treatment intensification by NNRTI, INSTI and/or R5 inhibitor was associated with a statistically significant improvement in cognitive tests at week 48 and week 96.

# Sub article: P218 Improved kidney function in patients who switch their protease inhibitor from atazanavir or lopinavir to darunavir

**Published:** 2016-10-23

**Authors:** Sophie Jose, Mark Nelson, Andrew Phillips, David Chadwick, Roy Trevelion, Rachael Jones, Debbie Williams, Lisa Hamzah, Caroline Sabin, Frank Post

**Affiliations:** 1 Department of Infection and Population Health, University College London, London, UK; 2 HIV Medicine, Chelsea & Westminster Hospital NHS Foundation Trust, London, UK; 3 Center for Clinical Infection, South Tees Hospitals NHS Foundation Trust, Middlesbrough, UK; 4 i-Base Treatment Information, HIV i-Base, London, UK; 5 Department of HIV/GUM, Chelsea & Westminster Hospital NHS Foundation Trust, London, UK; 6 HIV/GUM, Brighton and Sussex University Hospitals, Brighton, UK; 7 HIV and Sexual Health, Kings College Hospital NHS Foundation Trust, London, UK

## Abstract

**Introduction:** Atazanavir (ATV) and lopinavir (LPV) have been associated with kidney disease progression in HIV-positive individuals, with no data reported for darunavir (DRV). We examined kidney function in patients who switched their protease inhibitor from ATV or LPV to DRV.

**Materials and methods:** The UK CHIC study is an ongoing cohort of HIV-positive individuals accessing HIV care in the UK since 1996. Individuals who switched from either ATV or LPV to DRV with at least 6 months exposure and two estimated glomerular filtration rate (eGFR) measurements both pre- and post-switch were included in this study. Mixed effects linear regression models were used to compare pre- and post-switch eGFR slopes in all switchers, those with rapid eGFR decline (>5 mL/min/1.73 m^2^/year) on ATV or LPV, those with eGFR <60 mL/min/1.73 m^2^ prior to switch and according to tenofovir (TDF) use. Models were adjusted for age, gender, ethnicity and time-updated CD4 cell count, HIV RNA and TDF use. Mean (95% CI) eGFR slopes are reported in mL/min/1.73 m^2^/year.

**Results:** Data from 1691 patients were included. At the time of switching, median age was 45 years, 79% were male, 77% had an undetectable viral load, and the median eGFR was 93 mL/min/1.73 m^2^. Mean (95% CI) pre- and post-switch eGFR slopes were −0.97 (−1.35, −0.59) and 1.06 (0.69, 1.44) for ATV, and −0.51 (−0.90, −0.12) and 0.43 (0.14, 0.71) for LPV, showing a significant increase in eGFR after switching to DRV. Amongst those with rapid eGFR decline on ATV or LPV, stable or improved kidney function was observed following the switch to DRV (Table 1). Improved kidney function after switching was also observed in those with an eGFR <60 mL/min/1.73 m^2^ prior to switch (Table 1). When split by TDF use prior to switch, we observed steeper eGFR declines pre-switch and more rapid eGFR increases post-switch amongst those exposed to TDF, compared to those unexposed. Further, there was no significant difference in pre- and post-switch eGFR slopes amongst those not receiving TDF. Significant changes in eGFR slopes were still observed following switch to DRV in those who did not also discontinue TDF at the time of the switch (Table 1).

**Conclusions:** Improved kidney function was observed in patients who switched from ATV or LPV to DRV, particularly amongst those with renal dysfunction and those exposed to TDF prior to switching, suggesting that DRV may have a more favourable renal safety profile.

**Abstract P218–Table 1:** Pre- and post-switch eGFR slopes amongst individuals who switch from either atazanavir or lopinavir to darunavir

		Mean change in eGFR per year (95% CI)		
			
	N	Pre-switch	Post-switch	p
All switchers				
Atazanavir	676	−0.97 (−1.35, −0.59)	1.06 (0.69, 1.44)	<0.001
Lopinavir	1015	−0.51 (−0.90, −0.12)	0.43 (0.14, 0.71)	<0.001
Rapid eGFR decline (>5 mL/min/1.73 m2)				
Atazanavir	49	−14.74 (−18.79, −10.69)	2.55 (0.50, 4.61)	<0.001
Lopinavir	42	−12.99 (−15.68, −12.30)	0.63 (−0.85, 2.11)	<0.001
eGFR <60 mL/min/1.73 m^2^				
Atazanavir	87	−6.59 (−8.69, −4.48)	2.68 (1.23, 4.13)	<0.001
Lopinavir	66	−2.77 (−4.08, −1.46)	2.13 (0.28, 3.99)	<0.001
Received TDF prior to switch				
Atazanavir	478	−1.08 (−1.52, −0.64)	1.47 (1.01, 1.93)	<0.001
Lopinavir	605	−0.90 (−1.09, −0.52)	0.48 (0.13, 0.82)	<0.001
Did not receive TDF prior to switch				
Atazanavir	198	−0.27 (−0.93, 0.40)	0.69 (0.04, 1.34)	0.051
Lopinavir	410	0.35 (−0.87, 1.57)	0.55 (0.05, 1.05)	0.777
Did not discontinue TDF at the time of switch				
Atazanavir	530	−0.42 (−0.86, 0.02)	0.38 (0.07, 0.69)	0.006
Lopinavir	901	−0.44 (−0.74, −0.14)	0.52 (0.27, 0.77)	<0.001

# Sub article: P219 Renal health after long-term exposure to tenofovir disoproxil fumarate (TDF) in HIV/HBV co-infected individuals in Sub-Saharan Africa: results from the HEPIK cohort

**Published:** 2016-10-23

**Authors:** Giovanni Villa, Richard Odame Phillips, Colette Smith, Alexander Stockdale, Apostolos Beloukas, Lambert Tetteh Appiah, David Chadwick, Alessandra Ruggiero, Fred Stephen Sarfo, Frank Post, Anna Maria Geretti

**Affiliations:** 1 Institute of Infection and Global Health, University of Liverpool, Liverpool, UK; 2 Department of Medicine, Kwame Nkrumah University of Science and Technology and Komfo Anokye Teaching Hospital, Kumasi, Ghana; 3 Infection and Population Health, University College London, London, UK; 4 Centre for Clinical Infection, James Cook University Hospital, Middlesbrough, UK; 5 King's Centre for Global Health, King's College London, London, UK

## Abstract

**Introduction**: Tenofovir is recommended for the antiretroviral treatment of HIV-positive adults in Sub-Saharan Africa, including individuals co-infected with HBV. Use of TDF is gradually expanding in the region, where evidence indicates a high burden of pre-existing renal disease. This cross-sectional analysis evaluated the renal profile of HIV/HBV co-infected subjects receiving long-term TDF as part of ART in Kumasi, Ghana.

**Methods**: Patients underwent a comprehensive clinical and laboratory assessment, including serum biochemistry with creatinine and eGFR (CKD-EPI), urinary protein-to-creatinine ratio (uPCR), albumin-to-protein ratio (uAPR; if uPCR ≤20 mg/mmol), glycated haemoglobin (HbA1c), urinary schistosoma antigen, full blood count and CD4 cell count, and HIV-1 RNA and HBV DNA load. Tubular proteinuria (TP) was defined as a uPCR >20 mg/mmol in the absence of significance albuminuria (uAPR <0.4 mg/mmol).

**Results**: The study comprised 101 subjects (66% women; mean age 45 years) that had received ART for median 7.9 years (IQR 6.0–9.2) and TDF for median 4.1 years (3.9–4.3), 90% were on efavirenz (n=87) or nevirapine (n=4) and 10% were on lopinavir/ritonavir (LPV/r). CD4 counts were median 572 (383–716) cells/mm^3^. Overall 21% had detectable HIV-1 RNA (>40 copies/mL), with median levels of 4.2 (2.1–5.1) log10 copies/mL; 17% had detectable HBV DNA (>15 IU/mL), with median levels of 2.4 (1.7–3.4) log10 IU/mL. Blood pressure was raised in 35% of subjects and 10% had grade 3 elevations; 6% had diabetes (HbA1c ≥48 mmol/mol and/or specific treatment); 17% had a positive schistosoma test. Median uPCR was 13 (13–20) mg/mmol; 28% had uPCR ≥20 and 13% >50 mg/mmol. TP was detected in 16% of participants and was independently predicted by female gender (adjOR 10.5; 95% CI 1.3–88; p=0.03) and hypertension (adjOR 2.1 per grade increment; 95% CI 1.3–3.5; p<0.01). Five of 13 patients with uPCR >50 mg/mmol had uAPR <0.4, and this was associated with diabetes (OR 27; 95% CI 2.81–265; p<0.01). Median eGFR was 103 (91–115) and <60 mL/min/1.73 m^2^ in 4%. When comparing the eGFR measured after 1 year of TDF with the current one, the mean eGFR change was −2.6 mL/min/1.73 m^2^/year (SD±4.3), and independently predicted by LPV/r use (p=0.05) and a suppressed HBV DNA load (p=0.01).

**Conclusions**: Subjects on stable ART in Ghana have a substantial prevalence of comorbidities that can impact on renal function. The findings point to an urgent need to define ascertainment and management strategies for renal health in these populations.

# Sub article: P220 Factors associated with decreased estimated glomerular filtration rate (eGFR) among HIV-1 positive persons in methadone programme: data from Warsaw HIV Outpatient Clinic

**Published:** 2016-10-23

**Authors:** Bartłomiej Matłosz, Ewa Pietraszkiewicz, Ewa Firlag-Burkacka, Ewa Grycner, Andrzej Horban, Justyna Kowalska

**Affiliations:** 1 HIV Outpatient Clinic, Hospital for Infectious Diseases, Warsaw, Poland; 2 Department for Adults Infectious Diseases, Medical University of Warsaw, Warsaw, Poland

## Abstract

**Introduction:** Intravenous drug use is listed as one of the risk factors for impaired renal function; however, this group is rarely assessed for specific renal-related risks. Here we analyze the group of patients in methadone programme due to opiate and/or mixed addiction.

**Materials and methods:** Patients attending methadone programme from 1994 to 2015 were included in the study. Electronic medical records (available since 1994) included demographic data, laboratory tests, antiretroviral treatment history, methadone dosing and drug abstinence. Methadone was provided in oral solution (0.1% concentration before and 0.5% after 2 January 2014). Patients’ drug abstinence was routinely checked monthly on personnel demand (BioMaxima urine tests) for amphetamine, opiates, benzodiazepines and THC. We have evaluated two study outcomes: (i) having at least one (1eGFR) or (ii) three (3eGFR) eGFR <60 mL/min (MDRD formula). Logistic regression models investigated factors related to study outcomes (multivariate included all p<0.1 in univariate).

**Results:** In total, 267 persons with 2593 person-years of follow-up were included into analyses, 83 (31.1%) women, 218 (81.6%) infected through injecting drugs. Median age at entering HIV care was 30.2 (IQR 25.9–35.5) years, weight 69 (61–77) kg, HIV RNA 4.2 (3.2–4.8) log copies/mL, CD4 count 440 (255–619) cells/µL, serum creatinine 71 (56–88) mmol/L. At the time of analyses 251 (94%) were on ARV, 204 (81%) on PIs, 123 (46%) were anti-HBc total+ and 97 (36%) anti-HCV+. Median methadone dose was 80 (60–90) mg. Fifty-two (19.5%) patients had 1eGFR and 20 (7.5%) 3eGFR <60. In total, 57 (21%) patients broke abstinence with no effect on study outcomes (univariate OR 1.09 (0.35–3.41; p=0.88) for 1eGFR; OR 1.62 [0.72–3.68; p=0.25] for 3eGFR <60). Multivariate model results are presented in Table 1.

**Conclusions:** We have demonstrated high rate of kidney function impairment among HIV-1 positive patients in methadone programme. All risk factors for decreased eGFR in this subpopulation of patients were similar to those described for general HIV population [1]. Breaking abstinence and methadone dose had no effect on study outcomes. These findings imply the need for frequent but standard kidney function monitoring in this subgroup of patients.

**Abstract P220–Table 1:** Logistic regression odds ratios for having one or three eGFR measurements <60

	One eGFR <60				Three eGFRs <60			
		
	Univariate		Multivariate		Univariate		Multivariate	
				
Risk Factors	OR (95% CI)	p	OR (95% CI)	p	OR (95% CI)	p	OR (95% CI)	p
Gender (female vs. male)	2.77 (1.49–5.16)	0.001	4.58 (2.03–10.3)	0.0001	4.70 (1.80–12.26)	0.002	5.58 (1.84–16.9)	0.002
Entering care in 1999–2006 (vs. before 1999)	0.60 (0.27–1.36)	0.856	0.37 (0.13–1.01)	0.875	1.31 (0.47–3.63)	0.086	0.97 (0.25–3.68)	0.100
Entering care in 2007-2016 (vs. before 1999)	0.31 (0.12–0.78)	0.056	0.16 (0.05–0.55)	0.026	0.17 (0.2–1.30)	0.064	0.08 (0.01–0.89)	0.030
Age at registration (per 5 years older)	1.37 (1.10–1.72)	0.005	1.69 (1.27–2.23)	0.0003	1.24 (0.90–1.72)	0.179	1.48 (1.03–2.13)	0.036
Nadir CD4 count (per 1 cell higher)	1.00 (0.99–1.00)	0.084	1.00 (0.99–1.00)	0.196	1.00 (0.99–1.00)	0.110	–	–
Baseline eGFR (per 5 units higher)	0.88 (0.79–0.98)	0.019	0.85 (0.76–0.96)	0.010	0.71 (0.58–0.87)	0.001	0.73 (0.60–0.90)	0.003
Time on ARV (per 1 year longer)	1.11 (1.05–1.17)	0.0004	0.99 (0.91–1.08)	0.882	1.11 (1.03–1.21)	0.010	1.04 (0.93–1.17)	0.462
Undetectable on cART (yes vs. no)	3.90 (0.90–16.9)	0.069	1.13 (0.17–7.31)	0.899	2.63 (0.34–20.3)	0.349	–	–
Detectable on cART (yes vs. no)	2.34 (1.14–4.81)	0.02	1.64 (0.60–4.49)	0.338	2.29 (0.74–7.07)	0.148	–	–

HIV risk group, baseline HIV RNA, anti-HBc total and anti-HCV status, ARV group, methadone dose, breaking abstinence tested in univariate as non-significant (p < 0.1).

## References

[1] Mocroft A, Lundgren JD, Ross M, Law M, Reiss P, Kirk O (2015). Development and validation of a risk score for chronic kidney disease in HIV infection using prospective cohort data from the D:A:D study. PLoS Med.

# Sub article: P221 Kidney tubular dysfunction and decline in renal function in HIV-infected Chinese receiving tenofovir disoproxil fumarate

**Published:** 2016-10-23

**Authors:** Chung Yan Grace Lui, Pui Chung Denise Chan, CY Simon Cheung, Man Po Lee, Claire Naftalin, Ka Lun Chan, Ngai Sze Wong, Shui Shan Lee

**Affiliations:** 1 Department of Medicine and Therapeutics, Prince of Wales Hospital, Hong Kong, Hong Kong; 2 Stanley Ho Centre for Emerging Infectious Diseases, The Chinese University of Hong Kong, Hong Kong, Hong Kong; 3 Department of Medicine, Queen Elizabeth Hospital, Hong Kong, Hong Kong

## Abstract

**Introduction:** The prevalence of kidney tubular dysfunction (KTD) in Chinese HIV-infected individuals taking tenofovir disoproxil fumarate (TDF) and its impact on renal function over time are not known.

**Materials and methods:** A cross-sectional study was performed in a cohort of Chinese HIV-infected individuals in Hong Kong who had received ≥3 months of TDF. Blood and urine tests were taken to measure creatinine clearance (by Cockcroft Gault equation), and markers of KTD (fractional tubular resorption of phosphate and excretion of uric acid, β2-microglobulin, α1-microglobulin, N-acetyl-β-D-glucosaminidase and retinol-binding-protein). KTD was defined as the presence of at least three abnormal markers. Serial creatinine clearance from prior to initiation of TDF until up to 96 months post-treatment were collected from patients’ records. Variables associated with KTD were evaluated using binary logistic regression. Association between KTD and serial creatinine clearance was evaluated by generalized estimating equations (GEE).

**Results:** Hundred and forty-one HIV-infected individuals were recruited from June 2014 to January 2015: mean (±SD) age 46±10 years, 88% male, median (IQR) duration of HIV diagnosis 84 (40–155) months, 51% with history of AIDS, 8% with diabetes, 15% with hypertension, median duration of TDF 40 (17–61) months, 55% on protease inhibitors (PI). KTD was present in 21% of individuals, and was associated with older age, lower body weight, higher prevalence of diabetes, history of AIDS, lower nadir CD4 count, duration of TDF, use of PI and lower baseline creatinine clearance prior to initiation of TDF (all p<0.05). Multivariable analysis showed that KTD was independently associated with diabetes (adjusted odds ratio (OR) 11.5, 95% CI 2.1–61.8, p=0.005), current use of PI (OR 3.1, 95% CI 1.0–9.6, p=0.048), duration of TDF (OR 1.02, 95% CI 1.00–1.03, p=0.048) and baseline creatinine clearance (OR 0.97, 95% CI 0.95–1.00, p=0.022), after adjustment for the above variables. KTD was a significant variable for creatinine clearance across time in GEE model, and remained significant after adjustment for comorbidities, class of antiretroviral drugs, duration of HIV diagnosis, duration of TDF therapy and baseline creatinine clearance in GEE models. Creatinine clearance decreased over time after initiation of TDF (B=−0.14, p=0.01), and negatively correlated with KTD (B=−16.9, p=0.002). Annual rate of change of creatinine clearance was −2.14 mL/min and −1.57 mL/min in those with and without KTD (Figure 1).

**Conclusions:** KTD was present in 21% of HIV-infected Chinese individuals taking TDF, and was associated with more rapid decline in creatinine clearance over time.

**Abstract P221–Figure 1:** Change in creatinine clearance over time in those with (red line) and without (blue line) kidney tubular dysfunction.

# Sub article: P222 TDF, ATV/r and other ARV: renal safety in a resource-limiting country

**Published:** 2016-10-23

**Authors:** Francisco Beraldi de Magalhaes, Suelem Silva Penteado, Mariana Collodetto Soares, Daniel Claro Amaral, Melissa Mello Mazepa, Paulo Kato, Monica Gomes da Silva

**Affiliations:** 1 Infectious Diseases, Federal University of Parana, Curitiba, Brazil; 2 Pharmacy, Federal University of Parana, Curitiba, Brazil; 3 School of Medicine, Federal University of Parana, Curitiba, Brazil

## Abstract

**Introduction**: With increased options of ARV and the need for long-term intake, tolerance and safety are important characteristics of ARV combinations. In resource-limited settings, economic restrains may guide ARV choices [1]. In Brazil, a frequent combination is the association of TDF/3TC with ATV/r, mostly because its low pill burden compared to other local options. Concerning the nephrotoxicity of these ARVs [2,3], alone or in combination, we design this study to evaluate the eGFR in HIV patients taking ARVs, and compare the effect of different ARV combinations.

**Materials and methods**: This is a retrospective cohort to evaluate renal impairment in HIV-infected patients followed at the infectious disease out-clinic in a Brazilian hospital. During the study period, 777 patients were seen at the clinic and withdrawn their ARV at hospital's pharmacy. Patients were analyzed in four groups: group 1: patients taking TDF with any ARV except ATV/r; group 2: patients taking TDF associated with ATV/r; group 3: patients taking ATV/r with any ARV except TDF; group 4: patients taking any ARV but never ATV/r and TDF. All patients had their eGFR by using the CKD-EPI formula, calculated on their 6-month visits, until 4 years [4]. Proteinuria, crystalluria, diabetes, hepatitis B and C and viral load suppression were also evaluated.

**Results**: A total of 639 patients were enrolled. Comparing groups 1, 2 and 3 with group 4, we observed different decline in eGFR. In up to 4 years of follow-up, group 3 presents a reduction of 4.82 mL/min/1.73 m^2^ (p=0.0003), group 1 reduces in 4.25 mL/min/1.73 m^2^ (p<0.00001). However, group 2 exhibited a more pronounced reduction compared with other groups, declining 7.51 mL/min/1.73 m^2^ (p<0.00001) of eGFR after 4 years of use, compared to other strategies. Group 2 eGFR decline was 76% higher than in patients who took TDF without ATV/r and 56% higher than ATV/r without TDF. Group 4 expressed a lower eGFR reduction during study period. The presence of HCV co-infection was also associated with eGFR reduction of 13.89 mL/min/1.73 m^2^ (p=0.00026) as proteinuria and diabetes, eGFR decline of 6.01 mL/min/1.73 m^2^ (p<0.00001) and 3.40 mL/min/1.73 m^2^ (p=0.0628), respectively. Interestingly, eGFR was higher when patients maintain partially suppressed VL compared to those with undetectable VL.

**Conclusion**: In summary, combinations including TDF, ATV/r or both lead to a significant reduction in eGFR compared to strategies without these medications. This reduction is more pronounced with the association of TDF and boosted ATV. More convenient ARV options with safer kidney profile are needed in resource-limiting countries.

## References

[1] Santiago P, Grinsztejn B, Friedman RK, Cunha CB, Coelho LE, Luz PM (2014). Screening for decreased glomerular filtration rate and associated risk factors in a cohort of HIV-infected patients in a middle-income country. PLoS One.

[2] Ryom L, Mocroft A, Kirk O, Worm SW, Kamara DA, Reiss P (2013). Association between antiretroviral exposure and renal impairment among IV-positive persons with normal baseline renal function: the D:A:D study. J Infect Dis.

[3] Young J, Schäfer J, Fux CA, Furrer H, Bernasconi E, Vernazza P (2012). Renal function in patients with HIV starting therapy with tenofovir and either efavirenz, lopinavir or atazanavir. AIDS.

[4] Kidney Disease Improving Global Outcomes (KDIGO) KDIGO 2012 Clinical practice guideline for the evaluation and management of chronic kidney disease.

# Sub article: P223 Kidney transplant in HIV-positive population: outcomes and therapeutic perspectives in a 10-year experience

**Published:** 2016-10-23

**Authors:** Claudia Chirico, Ilaria Izzo, Salvatore Casari, Nicola Bossini, Nigritella Brianese, Maria Antonia Forleo, Emanuele Focà, Silvio Sandrini, Francesco Castelli

**Affiliations:** 1 Infectious Diseases, ASST Spedali Civili, Brescia, Italy; 2 Kidney Transplantation Unit, ASST Spedali Civili, Brescia, Italy

## Abstract

**Introduction:** The introduction of HAART made solid organ transplantation a concrete option for HIV-infected patients with end-stage organ disease, due to a prolonged life expectancy. Data about patients and grafts survival are encouraging, but there are still uncertainties about the prognosis in this category of patients and about PK interactions between HAART and immunosuppressive therapy, often with necessity of HAART modification. The aim of our study was to investigate the outcome of HIV-infected patients after kidney transplantation, focusing on the grafts survival, on the control of HIV disease and on HAART options.

**Materials and methods:** We performed a retrospective, single-centre study on kidney transplantation in HIV-positive patients, evaluated between 2005 and February 2016 in the Department of Infectious Diseases of Brescia, Northern Italy. We included HIV-positive patients with end-stage renal disease, sustained virologic suppression (if appropriate) and CD4+ T-cell count >200 cells/mm^3^.

**Results:** We evaluated 60 patients; 32 (53%) met the eligibility criteria (all in HAART except one patient) and entered the waiting list for kidney transplantation; 24 (40%) patients underwent transplantation, while 22 (37%) were excluded (three died, nine lost to follow-up, three transplanted in other centres, seven for personal reasons). In a median follow-up time of 51 months, we observed a cumulative number of 19 rejections in 15 patients (62.5%) and a general graft survival proportion of 67% (N=16 patients). Three patients (12.5%) experienced AIDS-defining events (one oesophageal candidiasis, two cutaneous Kaposi's sarcoma). We observed a mortality of 21% (five patients), for: invasive sinusal mucormycosis (one), sepsis in pancolitis (one), West Nile virus encephalitis (one), acute myocardial infarction (one) and colorectal cancer (one). To avoid PK interactions, we changed the regimen from a PI/NRTI-based to a INI-based regimen in 12 patients (50%).

**Conclusions:** Our study confirms the safety and effectiveness of kidney transplantation in HIV-infected patients. In our experience, we observed a high incidence of acute rejection, as reported by other studies. We expect that the recent implementation of the immunosuppressive protocol at transplant will allow a better immunologic control. The recent introduction of INI allows a better strategy of HAART, with lower incidence of PK interactions with immunosuppressive drugs.

# Sub article: P224 Renal function and chronic kidney disease prognosis through the lens of comorbidities in people living with HIV infection: an association hard to escape

**Published:** 2016-10-23

**Authors:** Ioanna Kalkounou, Theodoros Retsas, Sofia Kourkounti, Ioannis Katsarolis, Vasileios Paparizos

**Affiliations:** 1 HIV Unit, Andreas Syggros’ Hospital of Cutaneous & Venereal Diseases, Athens, Greece; 2 Medical Affairs, Gilead Sciences Hellas, Hellinikon, Greece

## Abstract

**Introduction:** Chronic kidney disease (CKD) is prevalent among people living with HIV infection (PLHIV). HIV care guidelines stress the importance of preserving renal function [1]. The aim of this study was to describe the parameters that affect renal function status in a cohort of PLHIV and the risk of progression to CKD.

**Materials and methods:** Cross-sectional, single-centre, retrospective study in a random sample of clinically stable PLHIV on routine follow-up during the last quarter of 2015. Creatinine clearance (eGFR) was calculated by Cockroft-Gault equation with the most recent serum creatinine and height/weight available within 6 months (grouped by ≥90, 70–89, 50–69, <50 mL/min). D:A:D algorithm for CKD risk progression was applied (low-medium-high risk) [2]. Demographics, HIV-relevant data and comorbidities were collected from file. Multimorbidity was defined as ≥2 prevalent chronic conditions per individual.

**Results:** Four hundred and forty-nine PLHIV were included in the analysis sample: 84.2% men, stage C 16.3%, age 47 years, time of HIV diagnosis 11 years, current CD4 607 cells/µL, CD4/CD8 0.69 (median values). Approximately 94% were on ART (median ART duration 4.7 years, VL <50 90%). The distribution of eGFR levels was as follows: ≥90 mL/min 39.9%, 70–89 mL/min 42%, 50–69 mL/min 16.1%, and <50 mL/min 2%. Classification in D:A:D risk groups was available for 438/449: low 31.7% – medium 27.2% – high 41.1%. For 282/449 (62.8%) a comorbidity was prevalent (associated with age p<0.001, time of HIV diagnosis p<0.001, eGFR p<0.001). Lipid disorders were the most prevalent (30.7%) followed by neuropsychiatric conditions (16.5%), viral hepatitis (8.9%), hypertension (6.9%), diabetes (5.1%) and cardiovascular disease (4.7%). In approximately 18%, ≥2 comorbidities were prevalent. Comorbidities’ prevalence differed per eGFR group: ≥90 mL/min 54%, 70–89 mL/min 62.2%, 50–69 mL/min 84.5% and <50 mL/min 88.9% (p<0.001). A similar pattern was identified for multimorbidity prevalence (p<0.001): ≥70 mL/min 14.1%, 50–69 mL/min 32.4% and <50 mL/min 44.4%. Multimorbidity followed the same pattern per D:A:D risk group: low 5% – medium 17.6% – high 27.8%. Of interest, 46/173 (26.6%) with eGFR ≥90 mL/min were at medium and high risk for CKD progression (26% and 0.6% respectively). For those 46 with normal eGFR and medium or high D:A:D risk score, 28 had at least one comorbidity (p<0.001).

**Conclusions:** Approximately 40% of the cohort displayed normal eGFR levels (≥90 mL/min), while 41% were at high risk for CKD progression. eGRF and D:A:D risk group were closely associated with the co-existence of comorbidities. Special consideration should be paid to PLHIV with normal eGFR levels and their treatment management, given the identified medium/high risk for CKD progression. Given the rising prevalence of ageing HIV+ population, implementation of the D:A:D CKD prediction algorithm, along with eGFR, may optimize HIV treatment decisions.

## References

[1] European AIDS Clinical Society (EACS) EACS guidelines version 8 [Internet]. http://www.eacsociety.org/guidelines/eacs-guidelines/eacs-guidelines.html.

[2] Achhra AC, Nugent M, Mocroft A, Ryom L, Wyatt CM (2016). Chronic kidney disease and antiretroviral therapy in HIV-positive individuals: recent developments. Curr HIV/AIDS Rep.

# Sub article: P225 Different classifications of chronic kidney disease (CKD) in HIV-infected patients result in large discrepancies in CKD prevalence in this population

**Published:** 2016-10-23

**Authors:** Amandine Gagneux-Brunon, Anne Frésard, Claire Guglielminotti, Céline Cazorla, Frédéric Lucht, Elisabeth Botelho-Nevers

**Affiliations:** 1 Infectious and Tropical Diseases, University Hospital of Saint-Etienne, Saint-Etienne, France

## Abstract

**Introduction**: Chronic kidney disease (CKD) is more prevalent in HIV-infected patients than in general population. EACS [1], IDSA [2] published guidelines for CKD diagnosis with classifications integrating estimated glomerular filtration rate (GFR) and urinary protein to creatinine ratio (uPCR) or urinary albumin creatinine ratio (uACR). These guidelines differ and may have an impact for the estimation of the prevalence of CKD in HIV-infected patients.

**Materials and methods**: We compared in a single centre population of HIV-infected patients the prevalence of CKD using French [3], EACS and IDSA guidelines for CKD diagnosis. GFR was estimated with MDRD (for French guidelines) and CKD-EPI (for EACS and IDSA guidelines). uACR and uPCR were measured in spot urine at the same time of estimates of GFR. EACS and IDSA classifications combined uPCR and/or uACR to eGFR. French classification uses only eGFR (except in patients with eGFR greater than 90 mL/min/1.73 m^2^, CKD is defined if uPCR >200 mg/g). We also estimated in this population the prevalence of eGFR under 60 and 70 mL/min/1.73 m^2^.

**Results**: We included 236 participants (mean age 48.9±10 years, sex ratio 4.64/1), 219 (92.4%) received combined antiretroviral therapies, and 201 (91.7%) of them had an undetectable viral load. Median of CD4 positive cells count was 552/mm^3^ (55–1840). Median uPCR and uACR respectively were 116 mg/g (0–8934) and 11 mg/g (0–5914). uPCR exceeded 150 mg/g in 86 (36.3%) patients and uACR exceeded 30 mg/g in 51 (21.5%) patients. Mean MDRD and CKD-EPI respectively were 93.4±21.2 and 97.7±17.3 mL/min/1.73 m^2^. EACS, IDSA with uACR, IDSA with uPCR, and French classifications respectively identify 21 (8.9%), 54 (22.9%), 87 (36.9%) and 126 (47.4%) patients at risk for poorer kidney outcomes (p<0.001 for EACS vs. IDSA, IDSA uACR vs. IDSA uPCR, French vs. EACS and IDSA guidelines). Eighteen and 26 patients were respectively identified with a GFR under 70 mL/min/1.73 m^2^ using CKD-EPI or MDRD (p<0.001). Nine and 18 patients were respectively identified with a GFR under 60 mL/min/1.73 m^2^ using respectively CKD-EPI and MDRD (p<0.001).

**Conclusion**: A standardized definition of CKD in HIV-infected patients based on both markers is needed. EACS and IDSA guidelines for CKD diagnosis should be more evaluated in HIV-infected patients.

## References

[1] European AIDS Clinical Society (2016). EACS Guidelines 8.0. [Internet]. http://www.eacsociety.org/guidelines/eacs-guidelines/eacs-guidelines.html.

[2] Gupta SK, Eustace JA, Winston JA, Boydstun II, Ahuja TS, Rodriguez RA (2005). Guidelines for the management of chronic kidney disease in HIV-infected patients: recommendations of the HIV Medicine Association of the Infectious Diseases Society of America. Clin Infect Dis.

[3] http://www.sante.gouv.fr/IMG/pdf/Rapport_Morlat_2013_Mise_en_ligne.pdf.

# Sub article: P226 Urinary products of N-acetyltransferase 8 as indicators of kidney disease progression in HIV infection

**Published:** 2016-10-23

**Authors:** Clara Dias, Lucília Diogo, Diva Trigo, Pedro Campos, Ana Lemos, Judit Morello, Patrícia Pacheco, Emília Monteiro, Karina Soto, Sofia Pereira

**Affiliations:** 1 Translational Pharmacology, CEDOC – NOVA Medical School/Faculdade de Ciências Médicas (NMS/FCM), Universidade Nova de Lisboa, Lisboa, Portugal; 2 Infectious Diseases, Hospital Fernando Fonseca, Lisboa, Portugal; 3 Nephrology, Hospital Fernando Fonseca, Lisboa, Portugal; 4 CEDOC – NOVA Medical School/Faculdade de Ciências Médicas (NMS/FCM), Hospital Fernando Fonseca, Translational Pharmacology/Nephrology, Lisboa, Portugal

## Abstract

**Introduction**: The continuous exposition to antiretrovirals has been pointed out as important risk factor for earlier kidney dysfunction in HIV+ individuals [1]. This nephrotoxic effect is mainly focused at kidney tubular level. Screening for newly selective and non-invasive markers reflecting a pathophysiological mechanism is paramount for early diagnosis in order to prevent kidney disease progression. The mercapturic acid pathway is a metabolic route for processing drugs and toxins. The last step of this pathway is catalysed by N-acetyltransferase enzyme type 8 (NAT8) allowing the transfer of an acetyl group from acetyl-CoA to the cysteine amino group, producing a mercapturic acid, which is excreted in the urine [2]. This proximal tubular enzyme has recently been pointed out as a regulator of kidney function and nephrotoxic response [3]. The aim of the present work was to evaluate N-acetylated cysteine-disulphides conjugates, namely N-acetyl-cysteine (uNAC) and coenzyme A (ucoA) on kidney disease progression.

**Methods**: A 1-year prospective nested case-control analysis was performed in a cohort of HIV patients under cART, with visits at time 0 of study admission (T0), 6 (T6) and 12 (T12) months. Glomerular filtration rate (eGFR) was estimated by CKD-EPI equation, expressed in mL/min/1.73 m^2^. Patients were stratified according their eGFR evolution: group A – no decline in eGFR; group B – declined eGFR ≥10% at T12. uNAC and ucoA were quantified by HPLC-FD. Data are presented as percentage relative to T0.

**Results**: A total of 23 HIV-infected patients were included (70% men, 30% Black, 53 [IQR 46–63] years old at month 0, 88% with undetectable viral load at month 0; cART scheme: 96% NRTI; 83% NNRTI; 29% PI; 8% II). The percentage of patients on tenofovir and the time of exposure to antiretrovirals between group A (69%, 8±4 years) and group B (70%; 9±6 years) was similar. The eGFR and analytes levels remained unchanged in group A through study time (n=13). Patients of group B (n=10) at month 12 showed decreased eGFR (83±7% of T0 paired t-test, p=0.010), corresponding to significant decreased uNAC (60±40% Wilcoxon signed rank test, p=0.006) and ucoA (44±SD27% paired t-test, p=0.032).

**Conclusions**: Kidney disease progression was associated with a significant decline in both acetylated cysteine-disulphides conjugates and coenzyme A. The present results might suggest the NAT8 role in the pathophysiological mechanism of underlying kidney dysfunction. This functional tool seems to be suitable to study kidney disease progression in HIV patients and to assess the contribution of antiretroviral drugs in this context.

**Financial support**: EXPL/DTP-FTO/1792/2013; PD/BD/105892/2014 (CGD).

## References

[1] Izzedine H, Harris M, Perazella MA (2009). The nephrotoxic effects of HAART. Nat Rev Nephrol.

[2] Veiga-da-Cunha M, Tyteca D, Stroobant V, Courtoy PJ, Opperdoes FR, Van Schaftingen E (2010). Molecular identification of NAT8 as the enzyme that acetylates cysteine S-conjugates to mercapturic acids. J Biol Chem.

[3] Juhanson P, Kepp K, Org E, Veldre G, Kelgo P, Rosenberg M (2008). N-acetyltransferase 8, a positional candidate for blood pressure and renal regulation: resequencing, association and in silico study. BMC Med Genet.

# Sub article: P227 Nephrotic range proteinuria in a patient with HIV infection and a history of intravenous drug abuse: case report and review of the literature

**Published:** 2016-10-23

**Authors:** Maria Tsilika, Dimitra Kavatha, Konstantinos Protopapas, Georgios Siakallis, Antonios Papadopoulos, Anastasia Antoniadou

**Affiliations:** 1 4th Internal Medicine Department, Attikon University Hospital, Athens, Greece

## Abstract

**Introduction**: Nephrotic range proteinuria in patients infected with HIV and a history of intravenous drug abuse may occur in the context of several underlying conditions such as HIV nephropathy, HCV or HBV nephropathy in co-infected cases, heroin-associated nephropathy or AA amyloidosis complicating chronic infections or lymphomas. We describe the case of nephrotic range proteinuria in a patient with HIV infection and active intravenous heroin abuse.

**Material and methods**: A 37-year-old Caucasian male with HIV and HCV co-infection was admitted to our department for epistaxis and anaemia. He had been successfully suppressed with HAART, with a CD4 cell count of 308/L. His medical history was also remarkable for active intravenous heroin abuse, chronic skin and soft tissue infection of the lower extremities and deep venous thrombosis for which he received acenocoumarol. Initial laboratory work-up revealed acute renal failure and significant hypoalbuminemia (creatinine: 3.2 mg/dL, urea: 68.9 mg/dL, ALB: 1.6 g/dL). Urine analysis was positive for excessive protein loss (16.489 g/24h), whereas his ultrasound imaging revealed enlarged kidneys with increased echogenicity. Further diagnostic work-up included renal biopsy, which was positive for AA amyloidosis.

**Results**: Recurrent chronic infections associated with intravenous drug abuse in HIV patients may be complicated with renal AA amyloidosis. Overt proteinuria with normal sized or enlarged kidneys may help diagnostic guidance; however, clinical features between HIVAN and AA amyloidosis are often indistinguishable thus making diagnostic accuracy quite daunting. Unless patients remain abstinent from drug abuse, prognosis is poor with rapid progression to end stage renal disease.

**Conclusions**: Treating physicians should be aware of the association between renal AA amyloidosis and intravenous drug abuse in HIV-infected patients, and its indistinguishable clinical and laboratory findings when compared to HIVAN. High-risk groups with chronic skin and soft tissue infections in the context of intravenous substance abuse should be periodically assessed for proteinuria. In suspicious cases, renal biopsy remains the gold standard for establishing accurate diagnosis and guiding therapeutic approach.

# Sub article: P228 Tolerability of integrase inhibitors in a real-life setting

**Published:** 2016-10-23

**Authors:** Mireia Padilla, Jhon Rojas, Ana Gonzalez-Cordon, Jose Blanco, Jordi Blanch, Montserrat Lonca, Berta Torres, Maria Martinez-Rebollar, Montserrat Laguno, Amparo Tricas, Ana Rodriguez, Josep Mallolas, Jose Gatell, Elisa de Lazzari, Judit Peñafiel, Esteban Martinez

**Affiliations:** 1 Infectious Diseases Unit, Hospital Clínic-IDIBAPS, University of Barcelona, Barcelona, Spain

## Abstract

**Introduction**: Integrase inhibitors are preferentially recommended in guidelines because they have shown better tolerability than other drugs in clinical trials. We aimed to compare the rates and reasons for discontinuation of raltegravir (RAL), elvitegravir (EVG) and dolutegravir (DTG) in a large cohort of HIV-infected patients.

**Methods**: Retrospective study of all antiretroviral-naïve and antiretroviral-experienced with undetectable plasma HIV RNA who were prescribed a first regimen containing RAL, EVG or DTG and had at least one follow-up visit. We predefined the following major outcomes: early (≤1 year) discontinuation, and early discontinuation due to toxicity. Specific toxicities were grouped by organs/systems according to the description in the clinical history database. We also planned sensitivity analyses regarding any discontinuation irrespective of follow-up, and discontinuation restricted to the period 2014–2015 (when all three integrase inhibitors were available). Incidence was calculated as the number of episodes per 1000 person-years. Risk factors for discontinuation were assessed by multivariate Cox models.

**Results**: Patients on EVG were younger, more commonly men who had sex with men, and with higher baseline CD4 cell count, and patients on RAL were less frequently males. Incidence of early discontinuation was 271 (n=71, 12.7% of the patients on RAL, n=557), 168 (n=26, 8.1% of the patients on EVG, n=322) and 264 (n=26, 12.3% of the patients on DTG, n=322) per 1000 patient-years (p=0.0821). Early discontinuations due to toxicity were more common with EVG (n=16, 5.0%) (61.5% of EVG discontinuations) than with RAL (n=20, 3.6%) (28.2% of RAL discontinuations) or DTG (n=8, 3.8%) (38.8% of DTG discontinuations) (p=0.0083). Specific reasons for early discontinuations due to toxicity were digestive (n=7, 35%), neuropsychiatric (n=7, 35%), skin/mucoses (n=4, 20%), muscular (n=3, 15%), respiratory (n=1, 5%) and systemic (n=2, 10%) for RAL; muscular (n=6, 38%), digestive (n=4, 25%), neuropsychiatric (n=3, 19%), skin/mucoses (n=2, 13%), and kidney (n=1, 6%) for EVG; and neuropsychiatric (n=7, 88%), muscular (n=3, 38%), and systemic (n=3, 38%) for DTG. Some specific toxicities such as neuropsychiatric (p=0.0046) or systemic (p=0.0224) were more common with dolutegravir. Age (HR 1.04, 95% CI 1.02–1.07, p=0.0007) was the only independent risk factor for early discontinuation due to toxicity. Planned sensitivity analyses confirmed previous results.

**Conclusions**: EVG tended to be less discontinued in general, but discontinuations due to toxicity were more common with EVG than with RAL or DTG. Neuropsychiatric toxicity leading to drug discontinuation was more frequently associated with DTG than with RAL or EVG.

# Sub article: P229 Erythrocyte inosine triphosphatase activity: a potential biomarker for adverse events during combination antiretroviral treatment for HIV

**Published:** 2016-10-23

**Authors:** Chantal Peltenburg, Jörgen Bierau, Jaap Bakker, Jolanda Schippers, Selwyn Lowe, Aimée Paulussen, Bianca van den Bosch, Mathie Leers, Bettina Hansen, Annelies Verbon

**Affiliations:** 1 Internal Medicine, Division Infectious Diseases, Erasmus Medical Center, Rotterdam, Netherlands; 2 Clinical Genetics, Maastricht University Medical Center, Maastricht, Netherlands; 3 Clinical Chemistry and Laboratory Medicine, Leiden University Medical Center, Leiden, Netherlands; 4 Integrated Care, Maastricht University Medical Center, Maastricht, Netherlands; 5 Internal Medicine, Division Infectious Diseases, Maastricht University Medical Center, Maastricht, Netherlands; 6 Clinical Chemistry and Hematology, Zuyderland Medical Center, Heerlen, Netherlands; 7 Gastroenterology and Hepatology, Erasmus Medical Center, Rotterdam, Netherlands; 8 Micobiology and Infectious Diseases, Erasmus Medical Center, Rotterdam, Netherlands

## Abstract

**Introduction**: Predicting whether adverse events (AEs) will occur in combination antiretroviral therapy (cART) for patients infected with HIV would be a valuable tool in the choice of cART regimens. A biomarker predicting AEs in other diseases is the enzyme inosine 5’-triphosphate pyrophosphohydrolase (ITPase). A decreased ITPase activity is associated with a reduced risk of anaemia in patients treated for hepatitis C, but with an increased risk of AEs in patients treated with thiopurines. The purine analogues abacavir, tenofovir and didanosine that are part of the backbone in most cART regimens are a potential substrate for ITPase. Here, we determined whether ITPase activity may be used as biomarker for occurrence of AEs during tenofovir, abacavir or didanosine use.

**Materials and methods**: In 393 adult HIV-seropositive patients (1464 cART regimens), AEs were defined as events that led to stop or change of cART regimen. Clinical and demographic data were retrieved from the Dutch HIV monitoring foundation and the medical records. ITPase activity in erythrocytes was measured. ITPase activity ≥4 mmol IMP/mmol Hb/hour was considered as normal. Logistic regression analysis with repeated statement and weighted by total duration of cART therapy and cumulative duration of purine analogue therapy was used to determine odds ratios (ORs) for developing AEs.

**Results**: Two hundred and five patients (52.2%) had an ITPase activity <4 mmol IMP/mmol Hb/hour. In cART regimens containing tenofovir a decreased ITPase activity was associated with a reduction in AEs (p=0.01; OR 0.65), a longer mean regimen duration (p=0.001) and significantly less often switching of medication secondary to AEs (p=0.02) compared to normal ITPase activity. Moreover, of all the renal AEs that occurred in patients using tenofovir 63.6% occurred in the patients with normal ITPase activity (p=0.04). In contrast, in cART regimens containing abacavir, a decreased ITPase activity was associated with increased switching of medication due to AEs (p=0.03) and significantly more AEs occurred compared to regimens prescribed in normal ITPase activity (crude p=0.02; after logistic regression p=0.08; OR 1.69). No association was found for ITPase activity and occurrence of AEs in didanosine-containing regimens.

**Conclusions**: Here, we show that ITPase activity is a potential biomarker for AEs in patients using tenofovir and abacavir in their cART regimen. ITPase enzyme activity <4 mmol IMP/mmol Hb/hour seems to be protective against occurrence of AEs in cART regimens containing tenofovir, while it leads to an increase in AEs in cART regimens containing abacavir.

# Sub article: P230 Dolutegravir tolerability in clinical practice: results from the SCOLTA cohort

**Published:** 2016-10-23

**Authors:** Giordano Madeddu, Elena Ricci, Roberto Gulminetti, Bagella Paola, Nicola Squillace, Giuseppe De Socio, Barbara Menzaghi, Orofino Giancarlo, Chiara Molteni, Stefano Rusconi, Benedetto Maurizio Celesia, Chiara Bellacosa, Antonio Di Biagio, Giovanni Pellicanò, Canio Martinelli, Laura Carenzi, Laura Valsecchi, Leonardo Calza, Katia Falasca, Francesca Vichi, Giovanni Penco, Paolo Bonfanti

**Affiliations:** 1 Department of Clinical and Experimental Medicine, Unit of Infectious Diseases, University of Sassari, Sassari, Italy; 2 Department of Infectious Diseases, ASST Fatebenefratelli Sacco, Milan, Italy; 3 Unit of Infectious Diseases, San Matteo Polyclinic Foundation – IRCCS, Pavia, Italy; 4 Infectious Diseases Unit, San Gerardo Hospital – University of Milano-Bicocca, Monza, Italy; 5 Infectious Diseases Unit, Santa Maria Hospital, Perugia, Italy; 6 Unit of Infectious Diseases, ASST della Valle Olona, Busto Arsizio, Italy; 7 Department of Infectious Diseases, Amedeo di Savoia Hospital, Turin, Italy; 8 Unit of Infectious Diseases, A. Manzoni Hospital, Lecco, Italy; 9 Infectious Diseases Unit, University of Milan, DIBIC Luigi Sacco, Milan, Italy; 10 Unit of Infectious Diseases, Garibaldi Hospital, Catania, Italy; 11 Infectious Diseases Clinic, University of Bari, Bari, Italy; 12 Unit of Infectious Diseases, IRCCS San Martino Hospital-IST, Genoa, Italy; 13 Infectious Diseases Unit, University of Messina, Messina, Italy; 14 Unit of Infectious and Tropical Diseases, Careggi Hospital, Florence, Italy; 15 Department of Infectious Diseases, S. Orsola Malpighi Hospital, University of Bologna, Bologna, Italy; 16 Infectious Diseases Unit, University of Chieti, Chieti, Italy; 17 Infectious Diseases Unit, Santa Maria Annunziata Hospital, Florence, Italy; 18 Infectious Diseases Unit, Galliera Hospital, Genoa, Italy

## Abstract

**Introduction**: In clinical trials dolutegravir (DTG) proved efficacious and safe in naïve and experienced patients. However, a recent study in a real-life setting reported an unexpectedly high rate of discontinuation mainly due to central nervous system (CNS) events.

**Materials and methods**: The SCOLTA project is a prospective, observational, multicentre study created to assess the incidence of adverse events in patients receiving new antiretroviral drugs. We aimed to further investigate the tolerability of DTG in a cohort of HIV-infected patients in clinical practice.

**Results**: A total of 358 HIV-infected patients were included, 266 (74.3%) males and 113 (31.6%) were heterosexuals. CDC stage was A in 156 (43.6%) patients. Mean age at enrolment was 46.9±11.4 years, mean CD4 cell count 520±383 cells/µL and mean HIV RNA 2.0±1.9 log10 copies/mL. Eighty-three (23.2%) patients were HCV Ab+ and 60 (16.7%) were naïve. After a median follow-up of 7 (IQR 6–11) months, 20 (4.5%) therapy interruptions were reported. These were caused by virologic failure in four (1.1%), death in three (0.8%), therapy simplification in two (0.5%), adverse events in eight (2.2%), lost to follow-up and other reason in one case each. Among adverse events-related interruptions two were grade ≥3 reactions, one acute renal failure and one rash, and six grade 1–2, one creatinine increase, one myalgia+rhabdomyolysis, one transaminase increase, two CNS events (one somnolence and one headache) and one gastrointestinal (vomiting). Among patients with available follow-up data at week 24 and 48, we found a significant reduction in eGFR at both follow-up times (−11.7 mL/min/1.73 m^2^, p<0.0001 and −9.1 mL/min/1.73 m^2^, p=0.001, respectively). Regarding lipid profile, we observed a non-significant reduction in total cholesterol at week 24 and 48 and a slight increase in HDL cholesterol. Triglycerides level showed a significant reduction at week 24 (−22.0 mg/dL, p=0.015) and a further decrease at 48 (−9.4 mg/dL, p=n.s.). Finally, both AST and ALT levels decreased during follow-up.

**Conclusions**: Dolutegravir was well tolerated during follow-up as confirmed by the low rate of both total DTG-based regimen discontinuations (4.5%) and adverse events-related interruptions (2.2%). eGFR showed an initial reduction but a stabilization during follow-up as already shown in clinical trials, possibly attributable to the inhibition of the OCT-2 creatinine transporter in the proximal tubular cells. Dolutegravir was also associated with an improvement of the lipid profile with significant reduction of triglycerides.

# Sub article: P231 Prevalence, spectrum, predictors and screening of clinically significant chronic liver disease associated with didanosine use in HIV-infected individuals

**Published:** 2016-10-23

**Authors:** James Millard, Yvonne Gilleece, Sumita Verma

**Affiliations:** 1 Infectious Disease, St. Mary's Hospital, London, UK; 2 Lawson Unit, Royal Sussex County Hospital, Brighton, UK; 3 Department of Medicine, Brighton and Sussex Medical School, Brighton, UK

## Abstract

**Introduction**: Chronic liver disease (CLD) is a leading cause of morbidity amongst HIV-infected individuals. An increasing burden is due to non-viral causes, including non-alcoholic fatty liver disease (NAFLD) and potentially hepatotoxic ARVs [1]. Exposure to the antiretroviral didanosine (DDI) can result in non-cirrhotic portal hypertension (NCPH) [2,3]. Our aim was to assess the spectrum of CLD associated with DDI use.

**Methods**: This prospective study (December 2014–April 2016) included HIV-infected individuals exposed to DDI for ≥6 months. Those without liver imaging (ultrasound Scan (USS), computed tomography (CT) or magnetic resonance imaging (MRI)) within 1 year underwent liver USS. Hepatic fibrosis was determined by assessment of liver stiffness measurement (LSM) using FibroScan^®^. Prior liver biopsy, laboratory and endoscopy results were reviewed and likely aetiology identified. Clinically significant CLD was defined by one or more of the following: portal hypertension (PHT), ≥F2 fibrosis (liver biopsy/FibroScan^®^), LSM ≥9.5 kPa (in HIV mono-infected without NALFD or alcohol excess), moderate–severe steatohepatitis on liver biopsy.

**Results**: Amongst our cohort of 2300 patients, 271 (11.8%) had ≥6 months DDI exposure. Complete data were available in 162. Individuals were a mean of 55 years old (range 27–83), predominately male (92.6%) and Caucasian (93.8%), HIV infected (mean 267, range 33–381 months) and taking ARVs (mean 237, range 21–544 months) for a prolonged period and most were virologically suppressed (85.2%). Current hepatitis C and B infection was present in 5.5% and 9.3%, respectively. PHT was present in 9.1%, with overall NCPH prevalence 3.1%. All individuals with NCPH had been previously identified by biopsy. Amongst individuals with NCPH, with LSM, 50% were abnormal, median 8.2 kPa (IQR 6.7–13.2). Individuals with NCPH had almost three times the median exposure to DDI (92 months vs. 34 months, p=0.067) and significantly lower current mean CD4 count (421 cells/mm^3^ vs. 676 cells/mm^3^, p=0.03), despite no difference in CD4 nadir (187 cells/mm^3^ vs. 193 cells/mm^3^, p=0.54) or virological suppression (<40 copies/mL; 80% vs. 85%, p=0.75). The prevalence of clinically significant CLD was 29.6%, with over half due to NAFLD.

**Conclusions**: Approximately 30% of HIV-infected individuals with DDI exposure have clinically significant CLD related to NAFLD (16.7%) and NCPH (3.1%). Fifty percent of those with NCPH had abnormal LSM and hence FibroScan^®^ lacked utility in either predicting NCPH or excluding fibrosis/cirrhosis in individuals with NCPH. Our preliminary results support screening for CLD in DDI-exposed individuals and emphasize the under-recognized burden from NAFLD.

## References

[1] Joshi D, O'Grady J, Dieterich D, Gazzard B, Agarwal K (2011). Increasing burden of liver disease in patients with HIV infection. Lancet.

[2] Khanna R, Sarin SK (2014). Non-cirrhotic portal hypertension – diagnosis and management. J Hepatol.

[3] Maida I, Garcia-Gasco P, Sotgiu G, Rios MJ, Vispo ME, Martin-Carbonero L (2008). Antiretroviral-associated portal hypertension: a new clinical condition? Prevalence, predictors and outcome. Antivir Ther.

# Sub article: P232 Neurocognitive performance and psychological symptoms improve in HIV-positive patients switching from an efavirenz (EFV)- to a rilpivirine (RPV)-based cART

**Published:** 2016-10-23

**Authors:** Carmela Pinnetti, Martina Ricottini, Pietro Balestra, Patrizia Lorenzini, Raffaella Libertone, Valentina Mazzotta, Maria Maddalena Plazzi, Rita Bellagamba, Mauro Zaccarelli, Andrea Antinori, Adriana Ammassari

**Affiliations:** 1 Clinical Department, National Institute for Infectious Diseases, Rome, Italy

## Abstract

**Introduction**: Neurocognitive impairment (NCI) is an important issue in the HIV setting, even though cART has reduced prevalence in recent years. Treatment with EFV may cause well-recognized neuropsychiatric side effects, but association with NCI remains controversial. Aim was to assess neurocognitive performance and psychological symptoms in patients switching from EFV to RPV.

**Materials and methods**: Single-centre prospective evaluation of patients switching from EFV to RPV in 2015. All patients underwent neuropsychological assessment (NPA), before (T1) and after (T2) the switch. NPA was carried out through a standardized and comprehensive battery of 14 tests (five different domains). Furthermore, the Beck Anxiety Inventory (BAI), Beck Depression Inventory (BDI) and Sleep Disorders Questionnaire were administered. Patients were classified as having NCI if they scored >1 standard deviation (SD) below the normal mean in at least two tests, or >2 SD in one test. HIV-associated neurocognitive disorders (HAND) were classified according to Frascati's criteria. Paired Wilcoxon and McNeamar tests were used for statistical comparisons.

**Results**: Forty-two patients were evaluated: 83.3% male; median age 46 years; 52.4% MSM; median education 13 years; 14% HCV-Ab positive; CD4/mm^3^ nadir was <200 in 35.7%; median CD4 were 555 and 621 cells/mm^3^ at T1 and T2, respectively; HIV RNA was <40 copies/mL in 95.2% and 97.6% of patients at T1 and T2. At T1, all patients were receiving an EFV-based cART (92.8% with FTC+TDF and 7.2% with ABC+3TC). After switch, all patients received coformulated TDF+FTC+RPV. Median time between the two tests was 6.6 months (IQR 4.2–10.9). At T1, 11 patients (26.2%) had NCI (mild neurocognitive disorder (MND) 2.4%; asymptomatic neurocognitive impairment (ANI) 16.7%; not HIV-related cognitive disorder 7.1%), whereas at T2, only seven patients (16.7%) presented NCI (ANI 11.9%; cognitive disorder not HIV-related 4.8%). NPA improved in five patients (11.9%), worsened in one (2.4%) and remained stable in 36 (85.7%). In particular, NPZ8 score significantly increased after switch (−0.05 IQR −0.33–0.42 vs. 0.20 IQR −0.08–0.51; p=0.002) (Figure 1). Also self-reported questionnaires (BAI and BDI-II) revealed an improvement at T2: the patients proportion with symptoms related to anxiety were 42.1% at T1 and 18.4% at T2 (p=0.013); depression was found in 37.8% at T1 and 18.9% at T2 (p=0.020).

**Conclusions**: Our results suggest that switch from EFV- to RPV-based cART may improve NPA as well as psychological symptoms in a relatively short period of time. EFV-treated patients, despite long-term therapy, should undergo systematic neurocognitive screening in order to identify subjects that may benefit from change of treatment.

**Figure 1:** Comparison of NPZ8 score in patients on EFV-based cART and after switch to RPV-based regimen.

# Sub article: P233 First-line ARV treatment characteristics among HIV-infected patients with and without comorbidities

**Published:** 2016-10-23

**Authors:** Girish Prajapati, Dongmu Zhang, Bridgett Goodwin

**Affiliations:** 1 Center for Observational and Real-World Evidence, Merck & Co., Inc., Rahway, NJ, USA; 2 Center for Observational and Real-World Evidence, Merck & Co., Inc., Upper Gwynedd, PA, USA

## Abstract

**Introduction**: The aim of this study was to describe ARV treatment patterns in previously naïve US-based HIV patients with and without comorbidities.

**Materials and methods**: A retrospective study was conducted using Truven Health MarketScan Commercial Claims and Encounters, and Medicare Supplemental and Coordination of Benefits database. Index date was the earliest date of any ARV medications between 1 July 2011 and 30 June 2014. HIV patients ≥18 years old on index date, and with continuous health plan enrolment of at least 12 months prior to and 15 days after index date, were included. Patients who received ARV drugs during 12 months prior to index date or had only one class of ARV drugs during observation period were excluded. Comorbidities in 12 months prior to index date were identified using ICD-9 diagnosis codes. ARV regimen was defined based on class of third agent used in combination with two NRTIs.

**Results**: A total of 9960 HIV patients were analyzed. Average age was 40 years (SD 12 years). Majority were men (79%), resided in South (46%) and had PPO/EPO health plan (58%). Lipid disorder (18%) was the most common comorbidity detected followed by hypertension (20%) and depression (12%). Patients with comorbidities were noted to be older except for patients with depression and tuberculosis (no difference in age), and anxiety and bipolar disorder (patients with comorbidity were younger). Presence of comorbidity was observed to be more common in men with exception of osteoporosis. A total of 9319 (94%) patients received ARV regimen containing two NRTIs with a NNRTI (44%), INSTI (27%) or a PI (23%). A total of 641 (6%) received other combinations of ARV drugs. NNRTI-based regimens were observed to be more commonly utilized when compared to INSTI- and PI-based regimens, respectively, among patients with lipid disorder (47% vs. 25% vs. 21%), cardiovascular disease (48% vs. 22% vs. 20%), cerebrovascular disease (36% vs. 24% vs. 31%), renal disease (39% vs. 21% vs. 26%), hepatic impairment (44% vs. 28% vs. 19%), diabetes mellitus/abnormal glucose control (50% vs. 23% vs. 19%), depression (39% vs. 31% vs. 25%), anxiety (40% vs. 35% vs. 21%) and bipolar disorder (37% vs. 36% vs. 23%).

**Conclusion**: DHHS guidelines recommend consideration of individual comorbidities when selecting initial ARV regimen. The study findings suggest the need for clinicians to consider comorbidities when selecting ARV therapy in order to minimize drug–drug interactions, adverse events and thereby optimize treatment outcomes.

# Sub article: P234 Adverse events occurring after introduction of EVG/COBI/FTC/TDF: data from the Surveillance COhort Long-term Toxicity Antiretrovirals/antivirals (SCOLTA) cohort

**Published:** 2016-10-23

**Authors:** Nicola Squillace, Paolo Bonfanti, Elena Ricci, Leonardo Calza, Benedetto Maurizio Celesia, Canio Martinelli, Francesca Vichi, Laura Cordier, Laura Carenzi, Marco Franzetti, Giuseppe De Socio, Francesca Peruzzu, Giancarlo Orofino, Chiara Bellacosa, Antonio Di Biagio, Andrea Gori, Tiziana Quirino

**Affiliations:** 1 Infectious Diseases Unit, San Gerardo Hospital, University of Milano-Bicocca, Monza, Italy; 2 Unit of Infectious Diseases, A. Manzoni Hospital, Lecco, Italy; 3 Department of Infectious Diseases, ASST Fatebenefratelli Sacco, Milan, Italy; 4 Department of Infectious Diseases, S. Orsola Malpighi Hospital, Bologna, Italy; 5 Unit of Infectious Diseases, Garibaldi Hospital, Catania, Italy; 6 Unit of Infectious Diseases, Careggi Hospital, Firenze, Italy; 7 Unit of Infectious Diseases, Santa Maria Annunziata Hospital, Firenze, Italy; 8 Infectious Diseases Clinic, ASST Fatebenefratelli Sacco, Milan, Italy; 9 Unit of Infectious Diseases, Santa Maria Hospital, Perugia, Italy; 10 Department of Clinical and Experimental Medicine, University of Sassari, Sassari, Italy; 11 Unit of Infectious Diseases, Amedeo di Savoia Hospital, Torino, Italy; 12 Infectious Disease Clinic, University of Bari, Bari, Italy; 13 Infectious Diseases Clinic, San Martino Hospital Genoa, University of Genoa, Genoa, Italy; 14 Unit of Infectious Diseases, ASST della Valle Olona – Busto Arsizio, Busto Arsizio, Italy

## Abstract

**Introduction**: Most studies evaluating safety of EVG/COBI/FTC/TDF described a significant increase of creatinine in the first 2 weeks of treatment and only few changes through 48 weeks and no significant elevation in ALT and AST [1,2]. Our aim was to evaluate the impact of this regimen on patients experienced (E) or naïve (N) to cART on liver and kidney toxicity.

**Materials and methods**: Patients initiating EVG/COBI/FTC/TDF were enrolled in SCOLTA project, a multicentre observational study reporting all adverse events (AEs). Patients were evaluated at T0 (baseline), T1 (6 months) and T2 (12 months). Groups were compared using chi-square for categorical variables and univariate and multivariate analysis of variance for continuous variables. Repeated measures were analyzed as change from baseline.

**Results**: Three hundred and twenty-nine patients were enrolled and 280 (85.1%) had at least one follow-up visit. Patients’ characteristics are depicted in Table 1: 202 (72.1%) were E and 78 (27.9%) were N.

ALT, alanine aminotransferase; AST, aspartate aminotransferase; BMI, body mass index; eGFR, estimated glomerular filtration rate; HDL, high-density lipoprotein-cholesterol; IQR, interquartile range; IVDU, intravenous drug user; SD, standard deviation.

The median observation time was 11 months (IQR 7–15.5). Fifty-four (19.3%) patients withdrew treatment: 11 were virologic failures, six switched for significant drug interactions, nine were lost to follow-up, 11 chose to interrupt. One patient died for hepatic cancer and one for accidental drug overdose. Fifteen patients (4.5%) interrupted their treatment because of AE (nine grade 1–2, six grade 3–4). At T1, we observed a significant decline in eGFR both in E and N patients (mean change from T0: E −7.0±14.1 mL/min, N −14.7±20.2 mL/min, p<0.001) that was confirmed at T2 (mean change from T0: E −7.1±17.7 mL/min, N −16.0±22.9 mL/min, p<0.001). After adjusting for HCV co-infection, CDC stage, BMI, CD4+ and eGFR at T0, change from baseline was statistically significant both in N and E patients at T1 and T2 versus T0. Both for naïve and experienced subjects, change from T1 to T2 was negligible (respectively, −1.2±12.2 and 0.9±15.7 mL/min). No significant differences were observed in AST and ALT (grade 1–2 AE) during the observation between N and E and between HCVAb-positive and HCVAb-negative patients. Four patients (two E, one of which HCV co-infected and two N) interrupted because of kidney-related events (impaired creatinine clearance), two for liver-related events (one liver decompensation in a N HCV co-infected and one transaminase increase in a N HCV negative).

**Conclusions**: A close monitoring of renal function is required in patients initiating EVG/COBI/FTC/TDF especially in first 6 months. No significant liver toxicity was observed.

**Abstract P234–Table 1:** Patients’ characteristics

	Experienced	Naive	
Variables	N = 202 (72.1%)	N = 78 (27.9%)	p
Males	147 (72.8)	65 (83.3)	0.06
Risk factor (IVDU)	50 (24.8)	2 (2.6)	<0.0001
CDC stage C	52 (25.7)	14 (18.0)	0.05
HCV co-infection (14 missing)	58 (30.0)	2 (2.7)	<0.0001
eGFR BL <90.0	99 (50.8)	23 (30.3)	0.002
	**Mean (SD) or median (IQR)**	**Mean (SD) or median (IQR)**	
Age (years)	45.6 (9.9)	39.2 (12.0)	<0.0001
BMI (kg/m^2^)	24.1 (3.4)	22.9 (2.7)	0.0009
CD4+ (cells/mL)	579 (356)	382 (278)	<0.0001
HIV RNA (copies/mL)	24 (19–67)	50,042 (8040–117,171)	<0.0001
ART duration (years)	8.1 (3.4–16.8)	0	
Total cholesterol (mg/dL)	191.0 (45.2)	159.5 (37.2)	<0.0001
HDL cholesterol (mg/dL)	43.3 (12.8)	40.1 (13.7)	0.09
Triglycerides (mg/dL)	140 (91.5–194.5)	92.5 (71–121)	<0.0001
Glucose (mg/dL)	94.2 (29.6)	89.9 (17.2)	0.25
AST (IU/L)	24.5 (20–35)	24.0 (19.5–32.0)	0.75
ALT (IU/L)	28 (18–46)	27 (16.5–37.5)	0.27
Creatinine (mg/dL)	0.94 (0.47)	0.99 (1.16)	0.61
Alcaline phosphatase (IU/L)	119.1 (77.4)	88.2 (42.4)	0.005
Phosphate (mg/dL)	3.19 (0.68)	3.10 (0.68)	0.46
eGFR	94.7 (25.3)	106.9 (28.4)	0.0007

## References

[1] Sax PE, DeJesus E, Mills A, Zolopa A, Cohen C, Wohl D (2012). Co-formulated elvitegravir, cobicistat, emtricitabine, and tenofovir versus co-formulated efavirenz, emtricitabine, and tenofovir for initial treatment of HIV-1 infection: a randomised, double-blind, phase 3 trial, analysis of results after 48 weeks. Lancet.

[2] DeJesus E, Rockstroh JK, Henry K, Molina JM, Gathe J, Ramanathan S (2012). Co-formulated elvitegravir, cobicistat, emtricitabine, and tenofovir disoproxil fumarate versus ritonavir boosted atazanavir plus co-formulated emtricitabine and tenofovir disoproxil fumarate for initial treatment of HIV-1 infection: a randomised, double-blind, phase 3, non-inferiority trial. Lancet.

# Sub article: P235 Prevalence of smoking and nicotine dependence in HIV patients, the project STOPS HIV from Italy

**Published:** 2016-10-23

**Authors:** Giuseppe Vittorio De Socio, Elena Ricci, Marco Dell'Omo, Paolo Maggi, Elisabetta Schiaroli, Carmen Rita Santoro, Nicola Squillace, Giorgia Angeli, Aria Patacca, Beatrice Tiri, Clara Abeli, Giordano Madeddu, Federica Magnè, Chiara Molteni, Francesca Vichi, Daniela Francisci, Franco Baldelli

**Affiliations:** 1 Malattie Infettive, Azienda Ospedaliero-Universitaria di Perugia, Perugia, Italy; 2 Malattie Infettive, Università di Milano, Milan, Italy; 3 Medicina del Lavoro, Azienda Ospedaliero-Universitaria di Perugia, Perugia, Italy; 4 Malattie Infettive, Università di Bari, Bari, Italy; 5 Division of Infectious Diseases, Department of Internal Medicine, San Gerardo Hospital, University Milano-Bicocca, Monza, Italy; 6 Malattie Infettive, Ospedale Terni, Terni, Italy; 7 Divisione di Malattie Infettive, Ospedale di Circolo, Busto Arsizio (VA), Italy; 8 Malattie Infettive, Università di Sassari, Sassari, Italy; 9 Malattie Infettive, Università di Genova, Genoa, Italy; 10 Unit of Infectious Diseases, Manzoni Hospital, Lecco, Italy; 11 Department of Infectious Diseases, Santa Maria Annunziata Hospital, Firenze, Italy

## Abstract

**Introduction**: Tobacco use is a leading cause of preventable illness and death for all individuals, but it is even more of a concern for people living with HIV, who tend to smoke more than the general population. Well-treated HIV-infected individuals may lose more life years through smoking than through HIV [1].

**Objective**: We aimed to investigate in HIV patients, prevalence of smoking, the nicotine dependence and the propensity to stop according to the stages of change by a standardized questionnaire.

**Methods**: Multicentre nationwide Italian study, consecutive HIV patients were included. We evaluated the nicotine dependence by Fagerström Test for Nicotine Dependence (FTND), and the propensity to stop according to the stages of change by a standardized questionnaire. Smokers and not smokers were compared using chi-square for categorical variables and univariate and multivariate analysis of variance for continuous variables.

**Results**: A total of 899 patients (age 48±11, male 71%, Caucasian ethnicity 88%) were included. Prevalence of current smokers was 52.6%, ex-smokers 16.9% and never smokers 30.4%. Among current smokers, the mean pack years was 23.9±19.6. According the stages of change, 65.2% of the smokers were in the precontemplation, 14.8% in contemplation, 15.8% in preparation and 4.2% in the action. The median of FTND was 4 (IQR 2–6). The dependence degree was low (point 0–4), moderate–high (point 5–6), very high (point 7–10) in 55.5%, 22.2% and 22.4%, respectively. The main study population characteristics are reported in Table 1.

In multivariable regression model including age, gender, risk factor for HIV acquisition and ethnicity, CD4+ cells count and atherosclerotic cardiovascular diseases risk prediction were confirmed as associated with current but not former smoking. Similarly, in a logistic multivariate model, current smoking remained associated with psychiatric comorbidity and alcohol use.

**Conclusion**: Prevalence rates for smoking in HIV+ subjects (around 50%) is higher than expected in the Italian general population (approximately 20%) [2], smoking screening and cessation support should be offered at HIV clinics. Our findings underscore the value of smoking cessation strategies targeting HIV+ persons.

**Abstract P235–Table 1:** Characteristics of 899 HIV patients at enrolment: 474 (52.7%) current smokers; 273 (30.4%) never smokers; 152 (16.9%) ex-smokers

	All HIV patients N = 899	Current smokers N = 474 (52.7%)	Ex-smokers N = 152 (16.9%)	Never smokers N = 273 (30.4)	p
Age (years)	48.0±11	46.9±10	53.5±12	46.5±12	<0.0001
Men (%)	71.2	73.2	80.3	62.3	0.002
Body mass index (kg/m^2^)	24.8±4	24.1±4	26.0±4	25.3±4	<0.0001
Caucasian ethnicity (%)	87.8	93.5	93.4	74.7	<0.0001
CDC C3 (%)	27.1	26.0	31.6	26.6	0.95
CD4 lymphocytes (mm^3^)	649±327	696±365	631±272	576±265	<0.0001
Psychiatric comorbidity (%)	9.5	13.5	5.9	4.4	<0.0001
ASCVD risk (%), median (IQR)	5.5 (2.6–10.1)	6.9 (4.2–12.0)	5.5 (2.6–10.9)	2.6 (1.1–6.1)	

Values are mean±SD.

ASCVD, atherosclerotic cardiovascular diseases risk prediction; p, unadjusted.

## References

[1] Helleberg M, May MT, Ingle SM, Dabis F, Reiss P, Fätkenheuer G (2015). Smoking and life expectancy among HIV-infected individuals on antiretroviral therapy in Europe and North America. AIDS.

[2] Gallus S, Lugo A, Colombo P, Pacifici R, La Vecchia C (2013). Smoking prevalence in Italy 2011 and 2012, with a focus on hand-rolled cigarettes. Prev Med.

# Sub article: P236 Soft modelling of health-related quality of life specific to HIV in relation to anxiety, depression, personality traits and precariousness

**Published:** 2016-10-23

**Authors:** Martin Duracinsky, Christophe Lalanne, Lionel Piroth, Mélanie Aboromman, Laurence Weiss, Agathe Rami, Olivier Chassany

**Affiliations:** 1 AP-HP DRCD EA REMES 7334, Hôpital Fernand Widal, Paris, France; 2 Internal Medicine, CHU Dijon Bourgogne, Dijon, France; 3 Clinical Immunology, Hôpital Européen Georges-Pompidou, Paris, France; 4 Internal Medicine, Hôpital Lariboisière, Paris, France

## Abstract

**Introduction**: Depression is a well-known predictor of health-related quality of life (HRQL), specifically in HIV and ageing populations. Little is known, however, on how precariousness is related to both depression and HRQL status. A structural equation model relying on partial least squares (SEM-PLS) was used to analyze the relation between anxiety/depression, harm avoidance and quality of life among HIV patients in an online survey.

**Methods**: Data were collected on 517 HIV+ patients (70% males, mean age 48 years) using validated self-reported measures. A structural model was posited a priori to link various domains: HRQL (PROOQL-HIV, three dimensions: physical, cognitive and social health, on a 0–100-points scale), personality traits (TCI-56, two dimensions: harm avoidance and novelty seeking) and anxiety/depression (HADS, two dimensions). Participants were classified into a precarious (52%) and a non-precarious (48%) group based on the French EPICES score. Two-group comparisons were performed using two-tailed Student's t and Pearson chi-squared tests. The links between the various dimensions were assessed using SEM-PLS on the whole sample and on the two subgroups separately.

**Results**: Adherence was high (97%) and few patients reported using drug or having excessive drinking habits. HRQL was higher in men (p<0.001), non-smokers and soft users of alcohol or drugs (p<0.01). Univariate analysis suggest that average scores were lower (p<0.001) in the precarious group for all dimensions of PROQOL-HIV (−14% up to −20%) and HADS (−10% and −14%) but not for harm avoidance. The SEM-PLS analysis indicates that all prespecified path coefficients were positive and significant at the 5% level. Depression was strongly associated with the cognitive (p<0.001) and the physical (p<0.001) dimensions of PROQOL-HIV while anxiety was strongly related to harm avoidance (p<0.001) as expected. However, no significant differences were observed between the two groups at the level of the structural models despite interesting variations in regression weights between the two subgroups (lower impact of depression of social and sexual relationships in the precarious group).

**Conclusion**: Depression is a strong predictor of lower physical, cognitive and social health but anxiety and personality traits are also potential moderators of HRQL, independent of the level of precariousness.

# Sub article: P237 Prevalence and predictors of HPV infection at oral cavity and anal site: findings in an Italian anal cancer screening programme for HIV-positive males

**Published:** 2016-10-23

**Authors:** Anna Rosa Garbuglia, Pierluca Piselli, Marco Gentile, Franca Del Nonno, Catia Sias, Daniele Lapa, Raffaella Libertone, Federico Lupi, Andrea Baiocchini, Maria Rosaria Capobianchi, Adriana Ammassari

**Affiliations:** 1 Laboratory of Virology, Istituto Nazionale per le Malattie Infettive “Lazzaro Spallanzani” IRCCS, Rome, Italy; 2 Department of Epidemiology, Istituto Nazionale per le Malattie Infettive “Lazzaro Spallanzani” IRCCS, Rome, Italy; 3 Clinical Department, Istituto Nazionale per le Malattie Infettive “Lazzaro Spallanzani” IRCCS, Rome, Italy; 4 Pathology Service, Istituto Nazionale per le Malattie Infettive “Lazzaro Spallanzani” IRCCS, Rome, Italy

## Abstract

**Introduction**: HIV-infected men carry an increased risk of HPV-associated infection, and information for appropriate allocation of cancer screening as well as vaccination programmes is needed. Purpose was to describe prevalence of HPV infection at the oral cavity of HIV-positive men, to identify predictors and to assess concordance with anal site.

**Methods**: Paired oral rinse and anal samples were collected from HIV-positive males within a large cross-sectional programme for anal cancer screening. Samples were tested with two sets of primers (MY09/MY11; FAP59/64) and HPV-positive samples typed by CLART2 HPV assay (Genomica) or direct sequencing. Cytologic examination and immunohistochemical analysis by using a monoclonal antibody against p16 (INK4a) were done in HPV-positive cases.

**Results**: Two hundred and forty-two HIV-positive males through homo-/bisexual contact in 85.5% and heterosexual in 10.3%. Median age 44.7 years. At testing, cART was prescribed in 93.9%, HIV RNA <40 copies/mL in 88% and median CD4 699/mm^3^. Prevalence of HPV in the oral cavity was significantly lower compared with anal site: 22.7% (n=55) versus 88.8% (n=207) (p<0.001). Multiple HPV types were found in 13 (23.6%) oral and in 157 (75.8%) anal samples (p<0.001). Risk factors for oral infection were: CD4 <200/mm^3^ (p=0.009), more than 10 partners in previous 12 months (p=0.01), more than 100 lifetime sexual partners (p=0.04). Infection at both sites was found in 51 (21.7%) cases and oral infection was more frequent in patients with HPV at anal site in respect to those without, but association was only slightly significant (24.6% vs. 11.4%; p=0.08). In Figure 1, frequency of HPV types by anatomic site, HR/LR groups and multiplicity are shown. Among the 51 patients with typed HPV infection at both sites, 44 (86.3%) had completely different HPV types at oral rinses from those found in anal swabs. Patients with theoretical benefit from 9-valent vaccination were: 17 (30.9%) with oral and 153 (73.9%) with anal infection. Cytologic examination of oral rinses showed ASCUS in 37 cases (49%) and HG-SIL in one.

**Conclusions**: In HIV-positive patients, prevalence of HPV infection in the oral cavity was significantly lower than that observed at anal site. Severe immune depression and sexual history, but not anal infection, are crucial to identify persons at highest risk of oral HPV infection and in need of screening. The absence of significant concordance between oral and anal sites may suggest different infection routes or timing.

**Abstract P237–Figure 1:** Frequency and multiplicity of HPV infection at the oral (a) and at the anal site (b).

# Sub article: P238 Prevalence and presentation of syphilitic hepatitis in HIV-infected patients

**Published:** 2016-10-23

**Authors:** Tyler Raycraft, Arshia Alimohammadi, Arpreet Singh, Ghazaleh Kiani, Rajvir Shahi, Syune Hakobyan, Brian Conway

**Affiliations:** 1 Clinical Research, Vancouver ID Research and Care Centre Society, Vancouver, Canada

## Abstract

**Introduction**: Rates of syphilis have been increasing in recent years, particularly in HIV-infected populations [1]. The reciprocal interaction between *Treponema pallidum* and HIV has been well established, but progression to liver inflammation, termed syphilitic hepatitis (present in up to 38% of cases in some studies [2]), is understudied in this setting. Liver enzyme abnormalities are well documented in HIV patients, often attributed to co-infection with viral hepatitis, alcohol use or direct hepatotoxicity of HAART [3]. However, previous studies have failed to sufficiently examine syphilis as a potential cause of hepatic inflammation. The aim of this analysis is to determine the prevalence of syphilitic hepatitis among HIV-infected individuals diagnosed with acute syphilis.

**Methods**: We performed a retrospective analysis of all HIV-infected individuals regularly attending a tertiary clinic in Vancouver, Canada. We identified cases of acute syphilis resulting in syphilitic hepatitis according to the following criteria: (1) RPR-confirmed *T. pallidum* infection occurring after HIV infection; (2) elevated liver enzyme laboratory tests, including alkaline phosphatase (ALP), alanine aminotransferase (ALT) and aspartate aminotransferase (AST) that normalized after penicillin treatment; and (3) no clearly identifiable cause of liver inflammation beyond syphilis (such as viral hepatitis or alcohol use). Any patient exhibiting ongoing risk factors for *T. pallidum* acquisition received routine syphilis screening every 6 months. In addition to laboratory data, demographic and clinical information were collected for each patient. The cases included in this study occurred between April 2011 and December 2015.

**Results**: Among 567 HIV-infected patients, 35 (6.2%) were diagnosed with acute syphilis based on RPR results. According to our definition, 3/35 (8.6%) cases of early syphilis resulted in syphilitic hepatitis. Within the cohort demonstrating syphilitic hepatitis, the age range was 28–63, the median RPR titre was 1:256 and all three self-identified as men who have sex with men (MSM). The common presenting symptoms are listed in Table 1. However, as demonstrated in Table 2, no symptoms demonstrated a statistically significant difference in prevalence between the syphilitic hepatitis and syphilis without hepatitis groups.

**Conclusions**: Syphilitic hepatitis is not uncommon in HIV-infected patients and should be considered as an etiologic agent in this setting. Active case finding and prompt initiation of treatment may contribute to the lower prevalence observed in our cohort as compared to previous reports in the literature.

**Abstract P238–Table 1:** Attributes of patients with syphilic hepatitis

Case Number	Age	Race	Duration of HIV (years)	HIV viral load (copies/mL)	CD4 cell count (cells/µL)	ARV regimen	Syphilis stage	RPR titre	Symptoms	ALT (IU/L)	AST (IU/L)	ALP (IU/L)	Treatment
1	28	AA	5	<40	670	EVG/c/TDF/FTC	Secondary	1:128	Abdominal pain, pharyngitis, lesion	605	427	541	Benzathine penicillin G (2.4 MU) IM×1
2	40	C	0	176,995	150	TVD/DRV	Secondary	1:256	Fever, arthralgia, rash	64	ND	307	Benzathine penicillin G (2.4 MU) IM×3
3	63	C	26	<40	450	TVD/RGV	Primary	1:256	Lesion, rash	76	50	382	Benzathine penicillin G (2.4 MU) IM×1

AA, African American; ALP, alkaline phosphatase; ALT, alanine aminotransferase; AST, aspartate aminotransferase; C, Caucasian; c, cobicistat; DRV, darunavir; EVG, elvitegravir; FTC, emtricitabine; ND, not done; RGV, raltegravir; TDF, tenofovir disproxil fumarate; TVD, Truvada.

**Abstract P238–Table 2:** Comparison of patients with acute/early syphilis with and without syphilitic hepatitis

Characteristic	Syphilitic hepatitis (N = 3)	Syphilis without hepatitis (N = 32)
Abdominal pain	1	1
Pharyngitis	1	0
Rash	2	17
Fever	1	1
Arthralgia	1	6
Skin lesion	2	7
Median RPR titre	1:256	1:10
Median CD4 count (cells/µL)	423	754
Median HIV viral load (copies/mL)	<40	<40
Receiving ARVs	3	28
Virologic suppression (VL <40 copies/mL)	2	24
Male	3	30
Mean age (range)	44 (28–63)	46 (25–63)
African American	1	1
Caucasian	2	27
Hispanic	0	1
Asian	0	3

VL, HIV viral load.

## References

[1] Chesson HW, Heffelfinger JD, Voigt RF, Collins D (2005). Estimates of primary and secondary syphilis rates in persons with HIV in the United States, 2002. Sex Transm Dis.

[2] Crum-Cianflone N, Weekes J, Bavaro M (2009). Syphilitic hepatitis among HIV-infected patients. Int J STD AIDS.

[3] Pol S, Lebra P, Vallet-Pichard A (2004). HIV infection and hepatic enzyme abnormalities: intricacies of the pathogenic mechanisms. Clin Infect Dis.

# Sub article: P239 The impact of engagement in care on the life expectancy of people living with HIV

**Published:** 2016-10-23

**Authors:** Sophie Jose, Margaret May, Caroline Sabin

**Affiliations:** 1 Department of Infection and Population Health, University College London, London, UK; 2 School of Social and Community Medicine, University of Bristol, Bristol, UK

## Abstract

**Introduction**: Poor retention in care is associated with higher rates of mortality. However, the impact of poor engagement in care (EIC) on life expectancy is not known.

**Materials and methods**: The UK CHIC study is a cohort of HIV-positive individuals who have accessed HIV care in the UK since 1996. Individuals who initiated ART aged ≥20 years between 2000 and 2011 with ≥1 year of follow-up were included. Pregnant women and injecting drug users were excluded. EIC rates at 1, 2, 3, 4 and 5 years on ART were calculated as the proportion of months since ART start that an individual was considered to be in care based on the REACH algorithm [1] and classified as high (≥80%) or low (<80%). Age-specific mortality rates from each time point on ART (1, 2, 3, 4 and 5 years) for those with high and low EIC were used to construct abridged life tables for the estimation of life expectancy. Life expectancy is the average number of additional years an individual can expect to live at a given age. Expected age at death at ages 35 and 50 are reported by adding the corresponding life expectancy.

**Results**: Amongst 18,046 individuals, median age at ART initiation was 38 years, 74.5% were male, 54.1% white, 53.5% acquired HIV through sex between men. Median (IQR) CD4 count was 230 (135–333) cells/mm^3^ and 66.5% started a non-nucleoside based regimen. EIC was good at 1, 2, 3, 4 and 5 years of ART duration, with 83.9%, 81.4%, 82.7%, 81.1% and 81.7% having high EIC, respectively. Expected age at death was lower for those with low EIC (Figure 1). Expected age at death increased with longer duration of high EIC on ART. At 1 year on ART, expected age at death (standard error) for a 35- and 50-year-old with high EIC was 72 (0.3) and 74 (0.2), and after 5 years on ART was 74 (0.4) and 76 (0.4). Life expectancy decreased with a longer duration of low EIC on ART. Expected age at death for those aged 35 and 50 years with low EIC was 68 (0.6) and 71 (0.6) at 1 year on ART, but decreased to 65 (0.8) and 68 (0.8) at 5 years on ART.

**Conclusions**: Poor EIC is associated with decreased life expectancy, especially if maintained over a long duration on ART. Whilst ART adherence is vital, high EIC may also contribute to good outcomes in people living with HIV.

**Abstract P239–Figure 1:** Life expectancy at exact ages 35 and 50 according to engagement in care rates at 1, 2, 3, 4 and 5 years on ART.

## References

[1] Howarth AR, Burns FM, Apea V, Jose S, Hill T, Delpech VC (2016). Development and application of a new measure of engagement in outpatient HIV care. HIV Med.

# Sub article: P240 Long sleep and longer naps are associated with severity of HIV disease

**Published:** 2016-10-23

**Authors:** Brice Faraut, Alexandre Malmartel, Jade Ghosn, Martin Duracinsky, Damien Léger, Sophie Grabar, Jean-Paul Viard

**Affiliations:** 1 Centre du Sommeil et de la Vigilance, Hotel Dieu Hospital, Université Paris-Descartes, Paris, France; 2 Family Medicine, Biostatistics and Epidemiology, Hotel Dieu Hospital, Université Paris-Descartes, Paris, France; 3 Immuno-infectiology Unit, Hotel Dieu Hospital, Université Paris-Descartes, Paris, France; 4 Department of Clinical Research (PRO Unit), Hôpital Fernand-Widal, Paris, France; 5 Biostatistics and Epidemiology Unit, Hotel Dieu Hospital, Paris, France; 6 Immuno-infectiology Unit, Hotel Dieu Hospital, Paris, France

## Abstract

**Introduction**: Total sleep time is usually linked to health status with short sleep <6 hours and long sleep >8 hours both associated with inflammation and a higher morbidity risk. In HIV infection, little is known on the association between sleep duration and quality, and disease severity.

**Methods**: Self-administered questionnaires were systematically proposed to HIV-infected patients in a single-centre study to assess insomnia (ICSD-3 criteria), poor sleep quality (PSQI >5) and total sleep time. Actigraphy over a 10-day period was also performed in a sub-sample of voluntary patients. SF-12 (38) and PROQOL-HIV (39) evaluated quality of life.

**Results**: Six hundred and forty patients were enrolled, including 97 with actigraphy recordings. PSQI >5 (68%) and insomnia (50%) reached high prevalence. A CD4 count <500 cells/mm^3^ was inversely associated with both insomnia (OR 0.73; p<0.01) and short sleep (OR 0.73; p<0.01) but positively associated with long sleep (OR 1.49; p<0.01). Long sleep according to actigraphy was also associated with a low CD4 nadir (OR 0.2; p=0.05) and AIDS status (OR 3.99; p=0.04). Seventy-six percent of long sleepers took long naps (≥1 hour) during weekdays, and napping ≥1 hour was associated with lower CD4 nadir and AIDS status when compared to napping less than <1 hour (OR 0.52; p=0.02 and OR 17.26; p=0.03, respectively). Self-reported napping ≥1 hour was also associated with a CD4/CD8 ratio <1 (OR 2.08; p=0.03 vs. non-napper status). The prevalence of PSQI >5 and insomnia were associated with the physical component of PROQOL-HIV (respectively: 64.5 vs. 85.5; p<0.01 and 66.3 vs. 81.5; p<0.01), SF-12 MCS (39.6 vs. 48.2; p<0.01 and 39.6 vs. 47.9; p<0.01) and PCS (48.0 vs. 53.7; p=0.03 and 48.8 vs. 52.3; p<0.01).

**Conclusions**: A high prevalence of insomnia and impaired sleep quality was found in HIV patients. Severity of the infection was not associated with short sleep but with long sleep and long naps, possibly in relation with T-cell activation.

# Sub article: P241 High rates of alcohol and illicit drug consumption among HIV-infected patients attended in Spain

**Published:** 2016-10-23

**Authors:** Alicia Gonzalez-Baeza, Victor Hontañon-Antoñana, Gema Caceres, Jesus Montes, Ignacio Perez-Valero

**Affiliations:** 1 HIV Unit, Hospital Universitario La Paz (IdiPAZ), Madrid, Spain; 2 Medicina Interna, Hospital Universitario La Paz (IdiPAZ), Madrid, Spain

## Abstract

**Introduction**: The prevalence of risky alcohol and illicit drug consumption and its associated factors is not well established among HIV patients attended in Spain.

**Materials and methods**: All the participants completed self-administered questionnaires to screen for risky alcohol consumption (AUDIT >8) and drug use (DUDIT >5 in men; DUDIT >1 in women), and emotional distress (HADS >12). Type of illicit drugs used and adherence rates were also reported. We calculated the association between clinical/demographic and consumption variables. Multiple logistic regressions were conducted including risky alcohol intake, risky drug use, use of drugs and polydrug use in the last year as dependent variables. Covariates included those clinical/demographic that showed association (p<0.10) with each dependent variable in the univariate analysis. Moreover, rates of consumption were compared among participants with different sexual orientation.

**Results**: Two hundred and forty-six participants were included. The majority were middle age (mean: 46.4 years), male (82%), high school or college educated (71.6%) and Spanish born (75.2%). The 96.7% received ART. Of all the participants, 32% reported consumption of any illicit drug during the last year: marijuana/cannabis (21.6%), cocaine (11.4%), poppers (6.5%), amphetamine derivatives (6.1%), opioids (2.9%), ketamine (2%), GHB (1.6%) and pentadone (1.5%). The prevalence of risky alcohol intake was 14% and 15.6% of the participants had risky drug consumption. Of the total of drug consumers, 21.5% received boosted ART agents. Patients out of ART (OR (95% CI) 5.22 (1.16–23.49), p=0.031) and those with poorer ART adherence (4.97 (1.88–13.30), p=0.001) had higher rates of risky alcohol intake. The use of any drug in the last year was independently associated with lower age (0.97 (0.94–0.99), p=0.016) and viral rebound in the last year (5.27 (2.11–13.14), p=0.001), whereas risky drug use was only associated with some viral rebound in the last year (3.09 (1.15–8.27), p=0.025). Moreover, homo- and bisexual participants (2.50 (0.98–6.38), p=0.055) and those younger (0.5 (0.91–0.97), p=0.008) had higher rates of polydrug use during the last year. Comparison between homo-/bisexual and heterosexual patients are included in Table 1.

Homo- and bisexual participants had higher rates of polydrug use, and amphetamine derivates, mephedrone, cocaine and popper use in the last year. Heterosexual participants had a trend towards higher cannabis and opiates use (Table 1).

**Conclusion**: We found a substantially high prevalence of risky alcohol intake and drug use. Measures of consumption were associated with poorer ART adherence, viral rebounds, age or sexual orientation. Homo- and bisexual participants had higher polydrug use and higher intakes of particular drugs – amphetamine derivates, cocaine, popper – that can be used as sexual enhancer. We show updated data of drugs and alcohol consumption in a HIV sample routinely attended in Spain.

**Table 1:** Comparison between homo/bisexual and heterosexual patients

	Homosexual/bisexual patients	Heterosexual patients	p
Age, mean (SD)	43.3 (10.9)	49.9 (7.8)	0.001
Years since HIV diagnosis, mean (SD)	10.6 (8.1)	20.3 (8.5)	0.001
Months on ART, mean (SD)	106.8 (86.2)	187.8 (90.2)	0.001
Non-Spanish born, N (%)	45 (34.4)	16 (13.9)	0.002
Risky alcohol intake, N (%)	14 (10.9)	18 (18.2)	0.127
Risky drug use, N (%)	19 (17.4)	18 (14.1)	0.724
Polydrug use, N (%)	25 (19.2)	7 (6.1)	0.002
Cannabis/marijuana, N (%)	22 (16.9)	31 (27)	0.063
Cocaine use, N (%)	22 (16.9)	6 (5.2)	0.004
Amphetamine derivates use, N (%)	15 (11.5)	0 (0)	0.001
Mephedrone use, N (%)	6 (4.6)	0 (0)	0.031
Poppers, N (%)	16 (12.3)	0 (0)	0.001
Opiates, N (%)	1 (0.77)	6 (5.2)	0.053

# Sub article: P242 Common comorbidities found in a population of clinically stable HIV-infected patients on chronic antiretroviral therapy in five ambulatory clinics in Lima-Callao, Peru

**Published:** 2016-10-23

**Authors:** Jose Hidalgo, Alberto Florez, Cecilia Agurto, Yvett Pinedo, Raul Gutierrez, Lourdes Rodriguez, Rosemarie Ayarza

**Affiliations:** 1 Infectious Diseases/HIV Clinic, Almenara Hospital/Via Libre, Lima, Peru; 2 Infectious Diseases, Grau Hospital, Lima, Peru; 3 Infectious Diseases, Sabogal Hospital, Lima, Peru; 4 Infectious Diseases, Loayza Hospital, Lima, Peru; 5 Medical Affairs, Merck Sharp & Dohme Peru, Lima, Peru; 6 HIV Clinic, Via Libre, Lima, Peru

## Abstract

**Introduction**: As earlier and more regular access to HAART increases globally, the proportion of chronically treated, clinically stable HIV patients will increase. The incidence of non-AIDS-defining comorbidities in these patients is not well defined in Peru. The aim of this study is to characterize this population and to describe most commonly found comorbidities to help design future policies of HIV care in the country.

**Materials and methods**: Review of HIV patients’ records selected from five HIV clinics in Lima-Callao attending regular appointments for follow-up visits in January–February 2016. Patients were adults (>21 years), ambulatory, on HIV therapy for >6 months and with no current or recent AIDS-defining condition (>6 months). Records were reviewed to collect information regarding epidemiologic, clinical and laboratory characteristics. Data obtained were processed statistically to describe frequencies observed.

**Results**: Three hundred and three patients were found eligible for review. A majority of patients were male (73.3%, n=222), with a median age of 46.1 years (range 21–79 years). Older individuals (≥60 years) were 15.2% (n=46) of the group. Patients had a diagnosis of HIV infection for an average time of 9.41 years, and were on HAART for an average of 7.78 years. Most patients were on an NNRTI-based first-line regimen (76.2%, n=231), followed by rescue regimens (12.2%, n=37), PI-based first-line regimens (9.3%, n=28) and other combinations for first-line therapy (2.3%, n=7). Median CD4 count was 614.2 cells/µL and proportion of patients with undetectable viral load (<40 copies/mL) was 91.1% (n=276). Seventeen patients (5.6%) had viral loads between 41 and 400, and only 10 patients (3.3%) higher than that. The most frequently observed metabolic diagnoses were dyslipidaemia, found in 40.6% patients (n=123), and obesity (BMI ≥30) in 11.9% (n=36). Diabetes mellitus was diagnosed in 21 patients (6.9%). Hypertension has been diagnosed in 23 patients (7.6%). Other recorded diagnoses of cardiovascular disease (coronary disease, CHF, cerebrovascular disease) were nine (3.0%), one myocardial infarction was reported in the group. Record of regular medical treatment for dyslipidaemia was 27.6%. Diabetes and hypertension were regularly treated in 91.3%.

**Conclusions**: A population of stable, ambulatory HIV patients on long-term HAART showed a high proportion of metabolic comorbidities, with dyslipidaemia the most frequent condition, followed by obesity. Prevalence of diabetes was similar to reported elsewhere. Relatively infrequent was cardiovascular disease. Medical treatment of dyslipidaemia was low. Care needs to consider proper treatment of chronic comorbidities in HIV.

# Sub article: P243 Quality of life in an Italian cohort of women living with HIV: preliminary results from IANUA study (Investigation on Antiretroviral Therapy)

**Published:** 2016-10-23

**Authors:** Alberto Venturini, Giovanni Cenderello, Barbara Giannini, Marcello Montefiori, Antonio Di Biagio, Giovanni Penco, Giovanni Mazzarello, Patrizia Orcamo, Caterina Merlano, Maurizio Setti, Mauro Giacomini, Claudio Viscoli, Giovanni Cassola

**Affiliations:** 1 Infectious Diseases, EO Ospedali Galliera, Genoa, Italy; 2 Informatica, Bioingegneria, Robotica e Ingegneria, Università di Genova, Genoa, Italy; 3 Economia, Università di Genova, Genoa, Italy; 4 Infectious Diseases, Università di Genova, Genoa, Italy; 5 Health Department, Regione Liguria, Genoa, Italy; 6 Clinical Immunology Unit, Università di Genova, Genoa, Italy

## Abstract

**Introduction**: The introduction of cART has reduced HIV-associated morbidity and mortality and changed the patients’ perspective of life. As a result, health-related quality of life (HRQoL) has become a crucial clinical issue. It has been suggested that factors influencing HRQoL in women may differ from those in men. Main objectives are as follows: assessment of HRQoL in a sample of Italian women from IANUA study; investigate correlation primarily between CD4+ cells count, viral load, co-infection HIV–HCV and changes in HRQoL.

**Materials and methods**: EQ-5D-3L self-reported questionnaire has been used in the evaluation of HRQoL. It assesses five dimensions: “mobility,” “self care,” “usual activities,” “pain/discomfort” and “anxiety/depression.” Each dimension has three levels: no problems, some problems and extreme problems. In addition, it includes a visual analogue scale (VAS) where one's own health “today” is rated from 0 “worst imaginable health” to 100 “best imaginable health.” The respondents provide information on marital status, education, employment/unemployment, other treatments used in addition to HAART (one, two, three, four, five or more) and number of hospitalizations due to HIV/AIDS.

**Results**: Three hundred and twenty patients completed the questionnaire. The mean age of the sample was 49 years (range 21–86). The mean VAS score was 74.1. It was lower than the mean VAS score of the sample of men (n=620) of 76.1 (p=0.01). Table 1 provides information about five dimensions of the EQ-5D-3L questionnaire.

Positive correlations were found between HRQoL and CD4+ cells count at last available visit (R 0.14, p=0.05) and nadir CD4+ cells count (R 0.17, p=0.01). Negative correlations were found between HRQoL and co-infection HIV–HCV (R −0.16, p=0.01) and anxiety/depression (R −0.49, p=0.01).

**Conclusions**: The analysis of self-reported questionnaires indicates that HRQoL in our sample group is not deeply affected by HIV/AIDS. The dimensions that are affected in the least are “mobility” and “self care” while the major problem is “anxiety/depression” with more than half of the sample reporting moderate or high level.

**Table 1:** Subjects frequencies in the EQ-5D dimensions

	No problems (%)	Some problems (%)	Extreme problems (%)
Mobility	257 (79.8)	61(18.9)	2 (0.6)
Self-care	298 (92.5)	22 (6.8)	1 (0.3)
Usual activities	271 (84.2)	45 (14)	4 (1.2)
Pain/discomfort	186 (57.8)	112 (34.8)	22 (6.8)
Anxiety/depression	127 (39.4)	158 (49.1)	35 (10.9)

# Sub article: P244 Seasonal variations in vitamin D levels in HIV-infected patients: when to test for hypovitaminosis?

**Published:** 2016-10-23

**Authors:** Sebastian Noe, Silke Heldwein, Rita Pascucci, Celia Oldenbuettel, Ariane von Krosigk, Carmen Wiese, Eva Jaegel-Guedes, Hans Jäger, Wolfgang Mayer, Eva Wolf, Christoph Spinner

**Affiliations:** 1 Private Practice, MVZ Karlsplatz, Munich, Germany; 2 Private Laboratory, MVZ Bavariahaus, Munich, Germany; 3 MUC Research, Munich, Germany; 4 Department of Medicine II, University Hospital Klinikum rechts der Isar, Munich, Germany

## Abstract

**Introduction**: The best time for accurate testing for vitamin D deficiency in HIV-infected patients remains unclear. We aimed to study the seasonal changes in serum 25OH-cholecalciferol (vitamin D), serum calcium and other markers of bone metabolism in an unselected European population of HIV-infected patients, most of whom were treated with antiretroviral drugs.

**Methods**: Retrospective single-centre study. Patients’ medical records were screened for serum vitamin D levels, β-crosslaps and surrogate values of bone turnover (serum calcium, phosphate and alkaline phosphatase (AP)).

**Results**: A total of 1011 data sets (625 patients) were evaluated. Overall, the median vitamin D level was 19.6 µg/L (95% confidence interval 18.8–20.6). In 207 (16.4%) data sets, patients were receiving oral cholecalciferol supplementation. Seasonal changes in serum vitamin D levels were reflected by minimum levels (median 13.5 µg/L) in March and maximum levels (median 23.7 µg/L) in July (p<0.001). Seasonal changes in vitamin D levels are shown in Figure 1. In contrast, serum calcium levels were lowest in September and October (2.23 mmol/L) and highest in May (2.32 mmol/L).

The dotted line shows a polynomic trend line, the x-axis indicates serum vitamin D levels (µg/L) and the y-axis indicates months (1=January, 12=December).

**Conclusions**: Significant variation in seasonal serum vitamin D levels was found in an unselected population of HIV-infected patients. This finding is in line with results from HIV-negative populations. Accordingly, the time point of vitamin D testing might be crucial for appropriate diagnosis of hypovitaminosis. We recommend vitamin D testing between December and May. However, given the seasonal variation, varying thresholds for vitamin D insufficiency and deficiency may be needed. As serum calcium levels did not demonstrate the same pattern, the meaning of this finding is unclear and warrants further investigation.

**Figure 1:** Monthly distribution of serum vitamin D levels showing seasonal changes in non-African patients not taking cholecalciferol (p < 0.001).

# Sub article: P245 Utilizing US prescription and claims data to better understand treatment dynamics of HIV/HCV co-infection patients in the second generation DAA era

**Published:** 2016-10-23

**Authors:** Michel Denarie, Steven Gubernick, Nirmala Panchatcharam, Shiwei Gao, Sean O'Neil, Jacob Arluck

**Affiliations:** 1 Real World Evidence Solutions, IMS Health, Plymouth Meeting, PA, USA; 2 IMS Consulting Group, IMS Health, Plymouth Meeting, PA, USA; 3 Advanced Analytics, IMS Health, Plymouth Meeting, PA, USA; 4 Managed Services, IMS Health, Plymouth Meeting, PA, USA

## Abstract

**Introduction and aims**: Since the introduction of the second generation DAAs, many HIV/HCV co-infection patients previously warehoused for HCV, but in treatment for HIV, started undergoing HCV therapy. The intent of this study is to better understand the treatment dynamics of HIV/HCV co-infection patients in the second generation DAA era. Key parameters include gender and age distribution across HIV third agents, proportion of patients receiving DAA therapies, proportion of HIV third agents pre- and post-HCV diagnosis and dynamics of patients who switched HIV third agents post-HCV diagnosis.

**Design and methods**: This retrospective study utilized IMS longitudinal prescription (LRx) and medical claims data (Dx). LRx covers 88% of all retail scripts. The linked Dx data cover 1.1 billion office claims annually. Patients were selected if they received an HIV diagnosis or HIV treatment between May 2014 and October 2015. Patients were identified as HCV if they received either a diagnosis or a treatment for HCV during the same period. Stability and eligibility rules were applied to the LRx to minimize the risk of anomalous results due to variability. We then observed the HIV treatment regimens pre- and post-HCV co-infection diagnosis.

**Results**: This retrospective study assesses IMS APLD and claims data from 30,061 patients having both HIV and HCV diagnoses. Sixty-eight percent of patients are male and 72% over the age of 50. Thirteen thousand three hundred and fifty-nine patients have both HIV and HCV diagnoses and an HIV index regimen. Of these patients, 69% are male and 73% are over the age of 50 with both age and gender similar across HIV third agents. Twelve percent received a DAA regimen for HCV. Greater than 88% of these patients were placed on a sofosbuvir-based regimen (Harvoni or Sovaldi), and this was similar across HIV third agents. One thousand five hundred and thirty-three patients have both HIV and HCV diagnoses and an identifiable HIV regimen pre- and post-HCV diagnosis. In these patients (which include those on standard triple therapy and nuc-sparing regimens), integrase inhibitors are the most frequently used HIV third agent pre- (51%) and post- (53%) HCV diagnosis followed by NNRTIs (33% pre and 32% post) and protease inhibitors (32% pre and 26% post). Hundred and seventy-three patients switched their HIV third agent post-HCV diagnosis with most (87%) switching to include an integrase inhibitor as part of their regimen. Of these 149 patients, 50% switched to Tivicay/Triumeq, 45% to Isentress and 5% to Stribild.

**Conclusions**: Only 11% of patients having both HIV and HCV diagnoses and an identifiable HIV regimen pre and post-HCV diagnosis switched their HIV regimens prior to initiating DAA therapy. Of those who switched their HIV regimens, 87% switched to include an integrase inhibitor. In addition, 95% of all switches to integrase inhibitors were to integrases not requiring a booster (Tivicay/Triumeq and Isentress).

# Sub article: P246 Comparison of early serologic response of early syphilis to treatment with a single-dose benzathine penicillin G between HIV-positive and HIV-negative patients: a cohort study

**Published:** 2016-10-23

**Authors:** Yi-Ching Su, Lan-Hsin Chang, Wen-Chun Liu, Cheng-Hsin Wu, Chia-Jui Yang, Chien-Ching Hung

**Affiliations:** 1 Internal Medicine, National Taiwan University Hospital, Taipei, Taiwan; 2 Internal Medicine, Far Eastern Memorial Hospital, New Taipei City, Taiwan

## Abstract

**Introduction**: Serologic response of early syphilis to treatment has been reportedly poorer in HIV-positive patients compared with HIV-negative patients; however, the interpretation of the published data is limited by the differences in study design, subjects with different stages of syphilis included, definition used for serologic response, treatment administered and follow-up frequency and duration. We aimed to compare the early serologic response of early syphilis to benzathine penicillin G (BPG) during the monthly follow-up for 3 consecutive months between HIV-positive and HIV-negative patients.

**Materials and methods**: Since January 2015, adult patients aged 20 years or older who presented with early syphilis (primary, secondary and early latent syphilis) with baseline rapid plasma regain (RPR) titres of 4 or greater were included in this prospective observational study after the patients received a single dose of BPG for early syphilis according to the STD Treatment Guidelines 2015 of US CDC [1]. RPR titres were determined at baseline and thereafter every 4 weeks for 12 weeks, followed by every 12 weeks. Serologic response was defined as decline of RPR titre by fourfold or greater at each time point compared with baseline. Serologic failure was defined as an increase of RPR titre by fourfold or greater during follow-up after ever achieving a decline of the titre.

**Results**: Between January 2015 and May 2016, 111 HIV-positive and 24 HIV-negative patients were included; all were men who have sex with men. Compared with HIV-positive patients, HIV-uninfected patients had more cases of secondary syphilis (66.7% vs. 30.6%, p=0.002), less early latent syphilis (25.0% vs. 60.4%, p=0.003), less prior syphilis (8.3% vs. 70.3%, p<0.001). HIV-negative patients had faster serologic response than HIV-positive patients: 58.3% versus 31.8% (p=0.02), 100% versus 60.2%, (p<0.001), 100% versus 77.7% (p=0.02) and 93.8% versus 80.4% (p=0.3) at week 4, week 8, week 12 and week 24, respectively. In multivariate analysis to examine the factors associated with 12-week serologic response, we found that the response was associated with early latent syphilis (adjusted odds ratio (AOR) 0.17; 95% confidence interval (CI) 0.05–0.61) and per 1−log_2_ increase of RPR titre at baseline (AOR 1.01; 95% CI 1.00–1.01, p=0.029).

**Conclusions**: HIV-negative patients had better early serologic response of early syphilis to BPG than HIV-positive patients during the first 12 weeks of follow-up. Early latent syphilis was associated with a poorer response while a higher RPR titre was associated with a better response to BPG.

# Sub article: P247 Cognitive and emotional functioning in HIV-infected MSM treated with effective antiretroviral therapy

**Published:** 2016-10-23

**Authors:** Bogna Szymanska, Emilia Łojek, Natalia Gawron, Grazyna Cholewinska, Ewa Firlag-Burkacka, Andrzej Horban, Robert Borstein

**Affiliations:** 1 Outpatients Clinic, Hospital for Infectious Diseases in Warsaw, Warsaw, Poland; 2 Faculty of Psychology, University of Warsaw, Warsaw, Poland; 3 HIV/AIDS, Hospital for Infectious Diseases in Warsaw, Warsaw, Poland; 4 HIV/AIDS, Medical University of Warsaw, Warsaw, Poland; 5 Department of Psychiatry and Behavioral Health, Ohio State University, Columbus, OH, USA

## Abstract

**Introduction**: There is growing evidence that despite effective cART, cognitive and emotional impairments have been still observed in HIV-infected individuals, which depends on multiple factors. One of the most important predictors of neurocognitive changes is the nadir CD4 cells count. The aim of the current report is to determine the neurocognitive and emotional differences between HIV-infected MSM with undetectable viral load and non-infected controls and to examine the significance of ARV regimens and nadir CD4 count in HIV(+) group.

**Materials and methods**: In this study, there were 95 HIV(+) MSM and 95 HIV-uninfected controls matched on socio-demographic variables. The characteristics of HIV(+) group were as follows: duration of HIV infection M=6.5 years (SD=5.8); CD4+ nadir M=265.2 cells/mL (SD=147.5); current CD4+ M=586.1 cells/mL (SD=217); duration of cART M=5.1 years (SD=4.9); 56% were treated with 2NRTI+PI/r, 23% 2NRTI+NNRTI, 21% other regimen. HIV(+) subjects were divided into two groups with nadir CD4 count <350 cells/mL (n=69) and >350 cells/mL (n=26). The participants performed a battery of standard neuropsychological tests and psychological questionnaires. In the analyses were used Student's, non-parametric tests (Kolmogrow–Smirnov) and correlations.

**Results**: HIV(+) and HIV(−) groups did not differ in terms of age and years of education. HIV(+) individuals achieved lower outcomes in attention (p<0.05), executive function (p<0.05) and language (p<0.01) tasks when compared to HIV(−) controls. Moreover, the HIV(+) group demonstrated higher levels of anxiety (p<0.05) and depression (p<0.05). There were no differences between cART regimens as well as cognitive and emotional domains. HIV-infected participants with CD4 nadir <350 cells/mL obtained lower results in attention (p<0.05) than HIV-infected participants with CD4 nadir >350 cells/mL.

**Conclusions**: Despite effective cART, the HIV(+) MSM showed lower functioning in neurocognitive domains and frail emotional condition as compared to the control group. We found differences in neurocognitive functioning in relation to nadir CD4 count. However, ARV regimen was not an important factor of cognitive decline in patients with undetectable viral load.

# Sub article: P248 TURQUOISE-I part 2: safety and efficacy of ombitasvir+paritaprevir/r±dasabuvir with or without RBV in patients with HIV-1 and HCV GT1 or GT4 co-infection

**Published:** 2016-10-23

**Authors:** Jürgen Rockstroh, Chloe Orkin, Rolando Viani, David Wyles, Anne Luetkemeyer, Adriano Lazzarin, Ruth Soto-Malave, Mark Nelson, Sanjay Bhagani, Hartwig Klinker, Giuliano Rizzardini, Pierre-Marie Girard, Nancy Shulman, Yiran Hu, Linda Fredrick, Roger Trinh, Edward Gane

**Affiliations:** 1 Medicine, Universitätsklinikum Bonn, Bonn, Germany; 2 Emergency Services, The Royal London Hospital, London, UK; 3 Infectious Disease Development, AbbVie Inc., North Chicago, IL, USA; 4 Medicine, University of California San Diego, La Jolla, CA, USA; 5 Infectious Disease, San Francisco General Hospital, San Francisco, CA, USA; 6 Infectious Disease, Fondazione Centro San Raffaele del Monte Tabor, Milan, Italy; 7 Infectious Disease, Innovative Care PSC, Bayamon, Puerto Rico; 8 Infectious Disease, Chelsea and Westminster Hospital, London, UK; 9 Infectious Disease, Royal Free London Foundation Trust, London, UK; 10 Infectious Disease, Universitätsklinikum Wuerzburg, Wuerzburg, Germany; 11 Infectious Disease, ASST Fatebenefratelli Sacco, Milan, Italy; 12 Infectious and Tropical Disease, Hopital Saint Antoine, Paris, France; 13 Statistics, AbbVie Inc., North Chicago, IL, USA; 14 Liver Unit, Medicine, Auckland City Hospital, Auckland, New Zealand

## Abstract

**Introduction**: Ombitasvir, paritaprevir co-administered with ritonavir, and dasabuvir (OBV/PTV/r+DSV) comprise the 3 direct-acting antiviral (DAA; 3D) regimen±ribavirin (RBV) approved for HCV genotype (GT) 1 infection. Here we investigate the safety and efficacy of 3D±RBV for GT1, and the 2 DAA (2D) regimen of OBV+PTV/r+RBV approved for GT4, in HIV-1 co-infected patients with or without compensated cirrhosis.

**Methods**: TURQUOISE-I, part 2 is a phase 3 multicentre study. Eligible patients were HCV treatment-naïve or RBV/interferon-experienced, on an HIV-1 antiretroviral regimen containing atazanavir, raltegravir, dolutegravir or darunavir (for GT4 only) and had plasma HIV-1 RNA <40 copies/mL at screening. Patients received OBV/PTV/r (25/150/100 mg)±DSV (250 mg)±weight-based RBV for 12 or 24 weeks per label guidelines. Interim safety and efficacy data are presented.

**Results**: Table 1 presents baseline demographics on 227 treated patients as of 21 April 2016. Of the 194 GT1- and 26 GT4-infected patients with available data, 98% and 100% achieved sustained virologic response (SVR) at post-treatment week (PTW) 4 (SVR4), respectively. Three patients experienced virologic failure: one GT1a, treatment-naïve patient without cirrhosis relapsed at PTW4, a second GT1a, treatment-naïve patient without cirrhosis relapsed at PTW12 and one GT1b, treatment-experienced patient with cirrhosis experienced breakthrough at week 10. No patients discontinued treatment due to adverse events (AEs). Most AEs were mild to moderate in severity, and key lab abnormalities were rare (Table 2).

**Conclusions**: The 2D and 3D regimens were well tolerated and yielded high SVR4 rates in patients with HCV GT1 or GT4/HIV-1 co-infection. OBV+PTV/r±DSV±RBV is a potent HCV treatment option for patients with HIV-1 co-infection, regardless of treatment experience or presence of compensated cirrhosis.

**Table 1:** Baseline demographics and disease characteristics

	GT1 N=199	GT4 N=28
Male, n (%)	156 (78)	26 (93)
White race, n (%)	172 (86)	25 (89)
Age, median (range), years	50 (26–69)	47 (30–63)
BMI, median (range), kg/m^2^	25 (17–41)*	24 (15–38)
HCV genotype 1a, n (%)	147 (74)	–
Cirrhosis, n (%)	22 (11)	0
Treatment-experienced, n (%)	64 (33)†	11 (39)
HCV RNA, median (range), log_10_ IU/mL	6.5 (1.8–7.6)	6.0 (4.7–7.0)
CD4+ cell count, median (range), /µL‡	612 (133–2351)	731 (262–1533)

BMI, body mass index.

* N=198

† N=193

‡ N=197 GT1, N=27 GT4.

**Table 2:** Safety and post-baseline laboratory abnormalities

Event, n (%)	GT1 N=199	GT4 N=28
Any AE	167 (84)	24 (86)
Serious AEs	9 (5)	1 (4)
RBV dose modifications due to haemoglobin decline	25 (13)	3 (11)
ALT grade ≥3 (>5×ULN)	1 (1)	0
Total bilirubin grade ≥3 (>3×ULN)	26 (13)	2 (7)
Patients on ATV-containing ART, n/N (%)	23/26 (88)	2/2 (100)
Haemoglobin grade 2 (<10 g/dL)	15 (8)	0
Haemoglobin grade 3 (<8 g/dL)	0	0

AE, adverse event; ALT, alanine aminotransferase; ATV, atazanavir; RBV, ribavirin; ULN, upper limit of normal.

# Sub article: P249 New findings in HCV genotype distribution in selected West European, Russian and Israeli regions

**Published:** 2016-10-23

**Authors:** Vladimir Kartashev, Matthias Doering, Leonardo Nieto, Eleda Coletta, Eva Heger, Maria Neumann-Fraune, Rolf Kaiser, Saleta Sierra

**Affiliations:** 1 Infectious Diseases, Rostov State Medical University, Rostov on the Don, Russian Federation; 2 Computational Biology and Applied Algorithmics, Max Planck Institute for Informatics, Saarbruecken, Germany; 3 Biochemistry and Microbiology, University Hospital Vall d'Hebron, Universitat Autònoma de Barcelona, Barcelona, Spain; 4 Microbiology and Immunology, Valladolid University Hospital, Valladolid, Spain; 5 Institute of Virology, University of Cologne, Cologne, Germany

## Abstract

**Introduction**: HCV affects 185 million people worldwide and leads to death and morbidities. HCV has a high genetic diversity and is classified into seven genotypes and 67 subtypes. Novel anti-HCV drugs (direct-acting antivirals) eligibility, resistance and cure rates depend on HCV geno/subtype (GT).

**Materials and methods**: Anonymized GT and epidemiological information gained in 2011–2015 was analyzed retrospectively. Data were obtained from 52 centres in Austria, Belgium, Germany, Israel, Italy, Luxembourg, Portugal, Russia, Spain and the UK.

**Results**: Thirty-seven thousand eight hundred and thirty-nine samples were included in the study. The most prevalent was GT1 (64.9%), followed by GT3 (20.9%) and GT4 (9.0%). Three samples classified as the recombinant genotype-P were identified in Munich (Germany). We show that the GT distribution is similar throughout Western European countries, with some local differences. Here, GTs 1b and 2 prevalences are lower and of GT 1a and 4 higher than in all previous reports. Israel has a unique GT pattern with only GT1b, 1a and 4 (78.6%, 20.2% and 1.2%, respectively). In South Russia, the GT proportions are more similar to Asia, with prevalent GTs 1, 3 and 2 (50.8%, 37.9% and 11.2%, respectively). GTs 5 and 6 were detected in very low proportions. Three cases of the recombinant genotype P were reported in Munich (Germany). In addition, we observed that GT proportion was dependant on patients’ gender, age and transmission route (Figure 1): GTs 1b and 2 were significantly more common in female, older, nosocomially-infected patients, while GTs 1a, 3 and 4 were more frequent in male, younger patients infected by tattooing, drug consume and/or sexual practises. In infections acquired by drug consume, GTs 1a (35.0%) and 3 (28.1%) prevailed. In infections related to sexual practises, lower proportion of GT3 (14.0%) and higher of GT4 (20.2%) were detected. GT4 was mostly abundant in MSM (29.6%). HIV co-infection was significantly associated with higher proportions GTs 1a and 4 (42.5% and 19.3%, respectively).

**Conclusions**: Genotype prevalence evolves and correlates to epidemiological factors. Continuous surveillance is necessary to better assess hepatitis C infection in Europe and to take appropriate health actions.

**Abstract P249–Figure 1:** HCV GT distribution patterns.

# Sub article: P250 Progression of liver fibrosis among HIV-infected patients under suppressive antiretroviral therapy: role of untreated HCV and other unrelated factors in the ICONA Foundation study cohort

**Published:** 2016-10-23

**Authors:** Giuseppe Lapadula, Alessandro Soria, Patrizia Lorenzini, Andrea De Luca, Massimo Puoti, Andrea Antinori, Claudio Mastroianni, Giovanni Cassola, Stefano Bonora, Mirella Santoro, Andrea Gori, Antonella d'Arminio Monforte

**Affiliations:** 1 Division of Infectious Diseases, San Gerardo Hospital, University of Milano-Bicocca, Monza, Italy; 2 Clinical Department, National Institute for Infectious Diseases, IRCCS, Lazzaro Spallanzani, Rome, Italy; 3 University Infectious Diseases Unit, Azienda Ospedaliera Universitaria Senese, Siena, Italy; 4 Department of Infectious Diseases, Ospedale Niguarda Ca’ Granda, Milano, Italy; 5 Infectious Diseases Unit, Sapienza University, Polo Pontino, Latina, Italy; 6 Division of Infectious Diseases, Galliera Hospital, Genova, Italy; 7 Unit of Infectious Diseases, Department of Medical Sciences, University of Torino, Turin, Italy; 8 Experimental Medicine and Surgery, University of Rome Tor Vergata, Rome, Italy; 9 Clinic of Infectious and Tropical Diseases, Department of Health Sciences, University of Milan, Milan, Italy

## Abstract

**Introduction**: Liver fibrosis progression is faster in HIV/HCV co-infected than in HCV mono-infected patients. We aimed at assessing the rate of progression to advanced liver fibrosis among HIV-infected patients on suppressive ART, with or without HCV co-infection, and identifying its predictors.

**Methods**: Patients from the ICONA cohort with known HCV-antibody (HCVAb) status and a FIB-4 ≤3.25 were studied from baseline (the first of two consecutive HIV RNA <50 copies/mL under ART), up to the last available FIB-4, HIV RNA rebound or anti-HCV treatment introduction, whichever occurred first. Time to development of advanced fibrosis (the first of two consecutive FIB-4 >3.25) was assessed using multivariable Cox analyses, separately conducted among HCVAb-positive and HCVAb-negative patients. The tested covariates were as follows: gender, country of birth, injecting drug use as HIV risk factor, CD4 nadir, baseline CD4, HDL cholesterol, diabetes, duration of HIV infection, HCV RNA, HCV genotype, baseline FIB-4 and first-line ART drugs.

**Results**: Five thousand seven hundred and seventeen patients with a median follow-up of 4 (IQR 2.2–7.4) years, contributing to 30,299 patient-years of follow-up (PYFU) were included. The median number of FIB-4 measurements was 7 per patient (IQR 4–14). Patients were predominantly males (75%), their median age was 40 years (IQR 34–46); 20% were HCVAb-positive. Median baseline FIB-4 was 1.09 (IQR 0.81–1.58) and 0.81 (IQR 0.59–1.12) among HCVAb-positive and HCVAb-negative patients, respectively (Table 1). During follow-up, 272 patients progressed to advanced fibrosis. Incidence (0.9 per 100 PYFU (95% CI 0.8–1.0)) was higher among HCVAb-positive patients with positive or unknown HCV RNA (2.94 (95% CI 2.43–3.55) or 3.10 (95% CI 2.51–3.83) per 100 PYFU) than among HCVAb-negative or HCVAb-positive HCV RNA-negative patients (0.33 (95% CI 0.26–0.41) and 0.49 (95% CI 0.19–1.32) per 100 PYFU, respectively). At multivariable analysis, in HCVAb-negative patients, higher baseline FIB-4 (per unit increase, HR 3.88, 95% CI 2.86–5.26, p<0.001) and first-line ART containing didanosine or stavudine (HR 1.65, 95% CI 0.99–2.75, p=0.054) were associated with higher risk of progression. In HCVAb-positive patients, adjusting for HCV RNA status, baseline FIB-4 (per unit increase, HR 3.81, 95% CI 3.15–4.60, p<0.001) and first-line didanosine or stavudine (HR 1.38, 95% CI 1.01–1.89, p=0.040) were associated with higher risk of progression to advanced fibrosis, while HDL cholesterol (>35 mg/dL vs. ≤35 mg/dL, HR 0.65, 95% CI 0.47–0.89, p=0.008) was protective.

**Conclusions**: In our cohort, progression to more advanced liver fibrosis was associated with HCV co-infection and, independently of HCV, to having received d-drugs-containing ART. Although these regimens are now abandoned, past exposure independently contributed to faster fibrosis progression, suggesting irreversible iatrogenic damage whose mechanism warrants further investigation. In HCV co-infected patients, HDL cholesterol had a protective role on fibrosis progression.

**Abstract P250–Table 1:** Baseline characteristics of HIV-infected patients under suppressive antiretroviral therapy, with or without HCV co-infection

Characteristics at baseline	HCVAb-negative	HCVAb-positive	p
Male gender, n (%)	3449 (75.4)	832 (72.7)	0.060
Age (years), median (IQR)	39 (33–47)	40 (36–45)	0.005
Migrants, n (%)	674 (14.7)	53 (4.6)	<0.001
IDU as mode of HIV transmission	108 (2.4%)	806 (70.4%)	<0.001
Time since HIV infection, sum (IQR)	1.9 (0.7–4.9)	9.0 (3.7–14.8)	<0.001
CD4 at baseline cell/mm^3^, n (%)	276 (152–378)	243 (120–343)	0.016
Nadir CD4, cell/mm^3^, n (%)	463 (321–619)	442 (285–630)	<0.001
Calendar year of baseline, median (IQR)	2010 (2004–2012)	2004 (2001–2008)	<0.001
Diabetes at baseline	176 (3.8%)	53 (4.6%)	0.226
HDL cholesterol, mg/dL	44 (37–53)	44 (36–55)	0.502
NVP-based first line	217 (4.7%)	79 (6.9%)	0.030
PI/r-based first line	1831 (40.0%)	267 (23.3%)	<0.001
ddI/d4t-containing first line	509 (11.1%)	261 (22.8%)	<0.001

# Sub article: P251 Hepatitis C can be cured for less than $100 per person: analysis of drug exports from India

**Published:** 2016-10-23

**Authors:** Andrew Hill, Dzintars Gotham, Melissa Barber, Anton Pozniak, Joseph Fortunak

**Affiliations:** 1 St Stephen's AIDS Trust, Chelsea and Westminster Hospital, London, UK; 2 Faculty of Medicine, Imperial College London, London, UK; 3 Centre of Development Studies, Cambridge University, Cambridge, UK; 4 Chemistry and Pharmaceutical Sciences, Howard University, Washington, DC, USA

## Abstract

**Introduction**: Novel direct-acting antivirals (DAAs) for chronic hepatitis C virus (HCV) achieve sustained virologic response (SVR) rates of >90%. Current high prices limit global access to DAAs. While Gilead (originator company of sofosbuvir) offers voluntary licences to generic producers in a limited number of countries, these countries represent only 50% of the worldwide epidemic. Costs of production can be estimated by tracking the average cost-per-kilogram of the active pharmaceutical ingredient (API) exported internationally from India, and combining the estimated per-pill API expenditure with other components of production cost (e.g. formulation, packaging). The per-kilogram price of API and steady, high demand volumes are key determinants of the total production cost of any medicine.

**Materials and methods**: Data were extracted from an online database of Indian export ledgers for per-kilogram prices and volumes of DAA APIs exported from India over January to June 2016. Average API costs were calculated for June 1, using linear regression models, weighted by individual export size. For velpatasvir, which is the newest DAA in this study and lacks export data, per-kilogram API cost was estimated from analysis of chemical synthesis processes described in originator patents. Costs of per-pill API requirements were combined with estimated costs for formulation and excipients ($0.04/pill), packaging ($0.35/month). Finally, a profit margin of 50% was added to estimate a price at which generic producers could profitably enter the market. Current US and Indian prices were collected, for comparison, from multiple databases.

**Results**: Export volumes from India in January–June 2016 were as follows: sofosbuvir 10,200 kg, (equivalent to 303,000 12-week treatment courses), daclatasvir 5443 kg (1,080,000 courses), ledipasvir 240 kg (32,000 courses). API prices decreased throughout the time frame. Mean API prices on 1 June 2016 were: sofosbuvir $1094/kg, daclatasvir $998/kg, ledipasvir $2441/kg. API cost for velpatasvir was estimated at $8900–11,700/kg. US prices were 1355 times higher than the target price for sofosbuvir, 4500 times higher for daclatasvir, 984 times higher for sofosbuvir+ledipasvir and 346–413 times higher for sofosbuvir+velpatasvir (Table 1).

**Conclusions**: HCV DAAs production costs are falling rapidly. Twelve-week treatments of sofosbuvir can be manufactured for $62, sofosbuvir+ledipasvir $96, daclatasvir $14, sofosbuvir+velpatasvir $181–216. These target prices all include a 50% profit margin for generic suppliers. These estimated generic prices for DAAs are comparable to those that have allowed massive treatment scale-up in HIV/AIDS.

**Abstract P251–Table 1:** Calculated target prices and current prices for 12-week DAA treatment courses

Drug	June 2016 API cost/kg (USD)	Target price per 12-week treatment	Current global lowest price per 12-week treatment	Current US price per 12-week treatment
Sofosbuvir (SOF)	$1094	$62	$324	$49,860–84,000
Daclatasvir	$998	$14	$153	$50,653–63,000
Ledipasvir (LDV)	$2441	$34	unknown	unknown
SOF+LDV	N/A	$96	$507	$56,700–94,500
Velpatasvir (VEL)	$8900–11,700	$119–154	unknown	unknown
SOF+VEL	N/A	$181–216	unknown	$74,760

# Sub article: P252 Hepatitis C virus screening project of patients on current anti-HCV therapy

**Published:** 2016-10-23

**Authors:** Elena Knops, Prabhav Kalaghatgi, Maria Neumann-Fraune, Eva Heger, Eugen Schülter, Joerg Timm, Andreas Walker, Thomas Lengauer, Verena Keitel, Tobias Goeser, Stefan Esser, Thomas von Hahn, Iris Pauser, Nazifa Qurishi, Katja Roemer, Stefan Scholten, Martin Daeumer, Martin Obermeier, Rolf Kaiser, Saleta Sierra

**Affiliations:** 1 Institute of Virology, University of Cologne, Cologne, Germany; 2 Computational Biology and Applied Algorithmics, Max Planck Institute for Informatics, Saarbruecken, Germany; 3 Institute for Virology, Duesseldorf University Hospital, Duesseldorf, Germany; 4 Gastroenterology and Hepatology, Duesseldorf University Hospital, Duesseldorf, Germany; 5 Gastrointestinal Center, Cologne University Hospital, Cologne, Germany; 6 Clinic for Dermatology, Duisburg-Essen University Hospital, Essen, Germany; 7 Department of Gastroenterology, Hepatology and Endocrinology, Medizinische Hochschule Hannover, Hannover, Germany; 8 Märkische Radioonkologische Versorgungszentren GmbH, Luedenscheid, Germany; 9 Private Practice Gotenring, Cologne, Germany; 10 Private Practice Hohenstaufenring, Cologne, Germany; 11 Institut für Immunologie und Genetik, Kaiserslautern, Germany; 12 Medizinisches Infektiologiezentrum Berlin, Berlin, Germany

## Abstract

**Introduction**: Clinical outcome of HCV therapy of direct-acting antivirals (DAAs) depends on host and viral factors. This observational, retrospective and non-interventional study collects data from viral geno/subtypes (GTs), DAA-resistance-associated mutations (RAMs) in the NS3/protease, NS5A and NS5B genes, to predict clinical outcome using the geno2pheno (HCV) tool. The current geno2pheno version interprets resistance according to viral GT background.

**Materials and methods**: Baseline NS3/protease, NS5A and NS5B sequences were obtained. Subtyping and presence of RAMs against asunaprevir (ASV), boceprevir (BOC), grazoprevir (GZV), paritaprevir (PTV), simeprevir (SMV), telaprevir (TVR), daclatasvir (DCV), elbasvir (EBR), ledipasvir (LDV), ombitasvir (OBV), dasabuvir (DSV) and sofosbuvir (SOF) were determined by sequencing (either Sanger or NGS) and subsequent interpretation with geno2pheno (HCV) (www.hcv.bioinf.mpi-inf.mpg.de/).

**Results**: One thousand five hundred and seventy HCV-infected patients from the PEPSI project have been enrolled until June 2016. We obtained 1024 NS5B sequences, which were used for genotyping. The most prevalent GTs were as follows: GT1a=39.1%; GT1b=34.2%; GT3a=16.6%; GT4d=4.6%. Baseline treatment susceptibility was analysed. 595 NS3/protease sequences were obtained and used for protease-inhibitors resistance prediction (Figure 1). Baseline resistance was found: ASV=2.8%; BOC=3.4%; GZV=16.5%; PTV=2.0%; SMV=21.8%; TVR=3.4%. For ASV, 22.0% of the samples were predicted as possibly resistant. NS5A: the susceptibility of 402 sequences was analyzed. The percentage of resistant samples was similar for all four NS5A inhibitors, 10.2–12.0%. NS5B: the sequence sets used for the analysis of each of the NS5B inhibitors varied, since the described RAM patterns for each drug comprise different amino acid residues. While 14.2% of the 502 sequences used for DSV screening were reported as resistant, none of the 912 samples screened for SOF were predicted as resistant. Baseline RAM analysis: the prevalence of the mutations NS3 80K and 170I (18.2% and 14.8%, respectively) leads to baseline resistance to SMV and GZV (21.8% and 16.5%, respectively). In addition, substitutions on other five amino acid positions were found in lower proportions. In the NS5A, amino acids exchanges at positions 28, 30, 31 and 93 were found, leading to NS5A baseline resistance of 10%. In the NS5B, the mutations 556GNR were found in 13.0% of the cases.

**Conclusions**: DAA susceptibility may be compromised at baseline. Baseline sequencing of target genes is cost-effective procedure to assess viral genotype in the DAA target genes and to determine resistance. These two parameters can help the physician to determine the best treatment and avoid misclassification of viral recombinant GTs.

**Abstract P252–Figure 1:** Proportion of samples resistant, partially resistant and susceptible to the licenced DAAs as predicted by geno2pheno(HCV).

# Sub article: P253 Are HIV/HCV co-infected patients more likely to experience multiple lines of ART than HIV mono-infected patients? Results from the Icona Foundation study

**Published:** 2016-10-23

**Authors:** Giulia Marchetti, Raffaele Bruno, Milensu Shanyinde, Cristina Mussini, Pietro Caramello, Mauro Zaccarelli, Massimo Andreoni, Antonella d'Arminio Monforte, Alessandro Cozzi-Lepri

**Affiliations:** 1 Clinic of Infectious Disease, Department of Health Sciences, ASST Santi Paolo e Carlo Hospital, University of Milan, Milan, Italy; 2 Department of Infectious Disease, Fondazione IRCCS Policlinico San Matteo Pavia, Pavia, Italy; 3 Department of Infection & Population Health, Division of Population Health, University College London, London, UK; 4 Clinic of Infectious Diseases, University of Modena, Modena, Italy; 5 Malattie Infettive I, Ospedale Amedeo di Savoia, Torino, Italy; 6 Department of Infectious Diseases, Lazzaro Spallanzani, Rome, Italy; 7 Infectious Diseases, University of Tor Vergata, Rome, Italy

## Abstract

**Introduction**: Despite a common perception that ART leads to more drug discontinuations in HIV/HCV co-infected patients, especially for certain compounds, as compared to HIV mono-infected, it remains unclear whether co-infection leads to higher frequency of treatment changes for a given time in care.

**Methods**: We performed a cross-sectional analysis within the Icona Foundation study cohort including all patients who started cART and were tested for HCVAb at least once over follow-up. People were defined HCVAb+ if they were ever tested positive. Individuals who seroconverted for HCV, spontaneously reverted to HCV negative, cured or HBV co-infected patients were excluded. Exposure factors were calculated at the date of starting cART apart from the number of ART lines ever used which was calculated at the date of last visit. Total ART lines used were calculated first counting all switches and, in three other analyses, counting only switches that occurred for a specific reason as reported by the physician: (1) treatment failure (virological/immunological failure); (2) change due to toxicity/intolerance; and (3) change due to ART simplification. Univariable and multivariable analyses logistic regression models were performed. Potential confounders used in the multivariable model are listed in the footnote of Table 1.

**Results**: We enrolled 8188 patients: 1626 HCVAb+, 6562 HCVAb−. At the date of starting cART, HCVAb+ patients were younger (median (IQR) 37 (33–42) vs. 38 (31–46); p<0.001); more frequently Italian (95.5% vs. 83%; p<0.001), IDU (79.4% vs. 2.4%; p<0.001), smokers (32% vs. 29%; p<0.001) and alcohol abusers (7% vs. 6%; p<0.001); they had lower CD4+ nadir (HCVAb+ with <350 CD4+/mL, 69% vs. 62%; p<0.001). Overall, in HCVAb+ subjects, a significantly higher proportion of HCVAb+ patients had a history of >3 ART lines (31% vs. 19%; p<0.001). Results were similar when counting only changes due to failure (4% vs. 3%, p<0.001) and toxicity/intolerance (8% vs. 5%, p<0.001). After controlling for potential confounders, especially after adding lifestyle factors, these differences were attenuated and, for the analysis counting switches due to simplification, there was an inverted trend (Table 1).

**Conclusions**: Overall, HCVAb+ individuals were more likely to be exposed to multiple lines than HIV mono-infected while in care, especially when comparing people who used at least three ART lines. This difference was most likely driven by toxicity and treatment failure. In contrast, HCVAb+ showed a cumulative lower risk to change ART because of simplification, reflecting the tendency of clinicians to maintain an already suppressive regimen in this population.

**Abstract P253–Table 1:** Odds ratios from fitting a logistic regression model

	n = 1626	n = 6562	All switches (n = 8188)			
No. of ARV lines previously used	HCVAb+ n (%)	HCVAb− n (%)	Unadjusted OR (95% CI)	p	Adjusted^a^ OR (95% CI)	p
Per additional			1.07 (1.03–1.12)	<0.001	1.01 (0.93–1.10)	0.82
0–1	566 (35%)	2745 (42%)	1.00		1.0	
2–3	560 (34%)	2541 (39%)	1.07 (0.94–1.21)	0.31	1.23 (0.96–1.69)	0.09
>3	500 (31%)	1276 (19%)	1.90 (1.65–2.18)	<0.001	1.72 (1.10–2.68)	0.02
			**Only switches due to treatment failure (n = 755)**			
Per additional	204	551	1.07 (1.03–1.12)	<0.001	0.95 (0.87–1.04)	0.27
None	1422 (87%)	6011 (92%)	1.00		1.0	
1 ARV	59 (4%)	141 (2%)	1.77 (1.30–2.41)	<0.001	0.94 (0.53–1.66)	0.82
2–3 ARV	75 (5%)	228 (3%)	1.39 (1.06–1.82)	0.02	0.79 (0.47–1.33)	0.38
>3 ARV	70 (4%)	182 (3%)	1.62 (1.23–2.16)	<0.001	0.81 (0.46–1.45)	0.49
			**Only switches due to toxicity/intolerance (n = 1471)**			
Per additional	391	1080	1.10 (1.06–1.13)	<0.001	1.01 (0.95–1.08)	0.80
None	1235 (76%)	5482 (84%)	1.00		1.0	
1 ARV	91 (6%)	213 (3%)	1.90 (1.47–2.44)	<0.001	1.28 (0.78–2.08)	0.33
2–3 ARV	165 (10%)	550 (8%)	1.33 (1.11–1.60)	0.002	1.38 (0.99–1.93)	0.06
>3 ARV	135 (8%)	317 (5%)	1.89 (1.53–2.33)	<0.001	0.98 (0.62–1.57)	0.96
			**Only switches due to simplification (n = 1292)**			
Per additional			0.89 (0.84–0.93)	<0.001	0.86 (0.80–0.94)	0.001
None	1474 (90%)	5422 (83%)	1.0		1.0	
1 ARV	9 (0.5%)	126 (2%)	0.26 (0.13–0.52)	<0.001	0.21 (0.07–0.59)	0.003
2–3 ARV	84 (5%)	728 (11%)	0.42 (0.34–0.54)	<0.001	0.74 (0.49–1.12)	0.16
>3 ARV	59 (4%)	286 (4%)	0.76 (0.57–1.01)	0.06	0.48 (0.27–0.82)	0.007

a Adjusted for age, CD4+, HIV RNA, mode of HIV transmission, gender, nationality, smoking, alcohol consumption, calendar year, follow-up duration and previous use of individual drugs.

# Sub article: P254 Rapid decline of anti-HCV antibodies following treatment of incident HCV infection in HIV-infected MSM in the Swiss HIV Cohort Study (SHCS)

**Published:** 2016-10-23

**Authors:** Karoline Aebi-Popp, Gilles Wandeler, Luisa Salazar-Vizcaya, Jan Fehr, Marcel Stoeckle, Matthias Hoffmann, Franziska Suter-Riniker, Alexander Luethi, Alexandra Calmy, Matthias Cavassini, Enos Bernasconi, Andri Rauch

**Affiliations:** 1 Infectious Diseases, University Hospital Bern, Bern, Switzerland; 2 Infectious Diseases, University Hospital Zuerich, Zuerich, Switzerland; 3 Infectious Diseases, University Hospital Basel, Basel, Switzerland; 4 Infectious Diseases, Cantonal Hospital St. Gallen, St. Gallen, Switzerland; 5 Institute for Infectious Diseases, University of Bern, Bern, Switzerland; 6 Infectious Diseases, University Hospital Geneva, Geneva, Switzerland; 7 Infectious Diseases, University Hospital Lausanne, Lausanne, Switzerland; 8 Infectious Diseases, Regional Hospital Lugano, Lugano, Switzerland

## Abstract

**Introduction**: Following clearance of hepatitis C virus (HCV) infection, HCV antibody titres (anti-HCV) may decline resulting in seroreversion. However, it is unclear whether changes in antibody levels differ between patients with spontaneous HCV clearance and those treated during early or chronic HCV infection.

**Material and methods**: We compared anti-HCV dynamics following an incident HCV infection after HIV diagnosis in 67 HIV-seropositive men who have sex with men (MSM) grouped by different clinical outcomes: 22 patients not treated for HCV infection (untreated), 12 with spontaneous HCV clearance and 33 with treatment-induced sustained virological response (SVR) (median time from diagnosis to treatment 3.2 months). Anti-HCV antibody levels were measured at baseline and annually for 3 years thereafter using a commercial ELISA kit (ARCHITEKT, Abbott Laboratories). Results were compared to 12 SHCS participants with chronic HCV infection acquired before HIV diagnosis and subsequent SVR (chronic HCV infection). We compared the relative change (%) in antibody levels between patient groups by estimating: (1) the maximum drop over the study period and (2) rates of decline per year over time. Re-infections were assessed by repeated HCV RNA measurements in all participants.

**Results**: MSM with SVR following treatment of incident HCV infections showed a more pronounced decrease in anti-HCV levels within the first 3 years after treatment (median decline 71%) compared to patients with spontaneous clearance (median decline 37.6%, p<0.001, Figure 1a). Antibody titres remained stable in untreated patients and in those treated during chronic HCV infection. There was no association between antibody decline and HCV genotype, IL28B, CD4+ T cell count and HIV viral load. Five of 33 (15%) with SVR and 1/12 (8%) subjects with spontaneous clearance seroreverted during follow-up. Nine (20%) subjects experienced a re-infection during follow-up; anti-HCV levels increased above the level of primary infection at time of re-infection. Figure 1b shows the estimated trajectories for the relative change in antibody levels. Patients with an SVR following an incident HCV infection experienced the fastest decline (rate (IQR)=−0.47 (−0.18 to −0.85)), followed by spontaneous clearers (−0.15 (−0.06 to −0.27)) and patients with an SVR due to HCV treatment during chronic HCV infection (+0.05 (−0.05 to 0.13)).

The origin was diagnosis of incident HCV infection for patients in the “untreated” and “spontaneous clearance” categories and HCV treatment start for patients in the “treated with SVR” and “treated during chronic infection” categories. The light grey dots indicate seroreversions

**Conclusions**: Treatment-induced HCV clearance of incident infection was associated with the greatest decline in anti-HCV antibody levels and with highest rates of seroreversions among HIV-seropositive MSM. These results suggest that fast clearance of HCV RNA following treatment of incident HCV infection might lead to a limited viral antigen stimulation required for a persistent antibody response.

**Figure 1:** Maximum and relative change in antibody titre (%) by clinical outcome category.

# Sub article: P255 Effectiveness of hepatitis A vaccination in HIV-positive men who have sex with men during an ongoing hepatitis A outbreak in Taiwan

**Published:** 2016-10-23

**Authors:** Kuan-Yin Lin, Pei-Ying Wu, Wen-Chun Liu, Hsin-Yun Sun, Yi-Chun Lo, Szu-Min Hsieh, Wang-Huei Sheng, Yu-Chung Chuang, Guan-Jhou Chen, Chien-Ching Hung, Shan-Chwen Chang

**Affiliations:** 1 Department of Internal Medicine, National Taiwan University Hospital, Taipei, Taiwan; 2 Center of Infection Control, National Taiwan University Hospital, Taipei, Taiwan

## Abstract

**Introduction**: An ongoing outbreak of acute hepatitis A virus (HAV) infection has been occurring among men who have sex with men (MSM) in Taiwan since June 2015, with more than 400 cases reported to the Taiwan CDC as of June 2016. This study aimed to evaluate the effectiveness of HAV vaccination in HIV-positive patients in an outbreak setting.

**Materials and methods**: In light of an ongoing outbreak of acute HAV infection among MSM, we prospectively performed a seroepidemiologic survey of HAV in HIV-positive patients during June 2015 to June 2016. The HAV-seronegative patients were offered HAV vaccine. The serologic outcomes were assessed after the first and last doses of HAV vaccine. The clinical outcome was acute HAV infection.

**Results**: During the 1-year study period, 1237 HAV-seronegative patients with 94.7% being MSM and a median CD4 count of 567 cells/mm^3^ (range 4–2342 cells/mm^3^) were included for analysis. Before 30 June 2016, 728 patients (58.9%) had received at least one dose of HAV vaccine, and 100 (8.1%) had completed the two-dose vaccine series. Compared with non-vaccinated patients, the vaccinated patients were older (mean age, 35.5 years vs. 34.0 years), less likely to be anti-hepatitis C-positive (5.5% vs. 10.8%) and more likely to have received antiretroviral therapy (97.0% vs. 93.9%) and have had HAV vaccination previously (8.9% vs. 4.5%). The overall seroconversion rate before the administration of the second dose of HAV vaccine was 27.8%. The seroconversion rates within 4 weeks, at weeks 4–8, weeks 8–16 and weeks 16–24 were 18.1%, 20.6%, 48.4% and 51.9%, respectively. One month after the last dose, the seroconversion rate increased to 95.5%. The factors associated with seroconversion between the first and last doses of HAV vaccination were time to anti-HAV IgG testing (adjusted odds ratio (AOR), per 1-week increase, 1.16; 95% CI 1.09–1.22) and previous HAV vaccination (AOR 47.72; 95% CI 7.16–317.97). The incidence rate of acute HAV infection in patients with and without HAV vaccination was 0.13 and 6.45 per 100 person-years, respectively, resulting in a vaccine effectiveness of 98.0%. The predicting factors of acute HAV infection included having had not receiving HAV vaccine (adjusted hazard ratio (AHR) 45.45; 95% CI 5.95–333.33) and recent syphilis (AHR 6.11; 95% CI 3.02–12.39).

**Conclusions**: Despite the impaired immunity from HIV infection and delayed serologic response to HAV vaccination in HIV-positive MSM, the risk of acute HAV infection was significantly reduced among the vaccinated patients during the HAV outbreak setting.

# Sub article: P256 High sustained virological response rates using imported generic direct-acting antiviral treatment for hepatitis C, imported into Australia, UK, Europe and North America

**Published:** 2016-10-23

**Authors:** Andrew Hill, Anna Savage, Greg Jeffreys, Richard Sallie, Adam Kennedy, Pham Thi Ngoc Nieu, John Freeman, James Freeman

**Affiliations:** 1 St. Stephen's AIDS Trust, Chelsea and Westminster Hospital, London, UK; 2 Faculty of Medicine, Imperial College London, London, UK; 3 University of Tasmania, Hobart, Australia; 4 Hepatology, Nedlands Medical Centre, Perth, Australia; 5 Kingswood, Pharmacy, Kingswood, Australia; 6 FixHepC, GP2U Telehealth, Hobart, Australia; 7 Nephrology, Sandy Bay Medical, Sandy Bay, Australia

## Abstract

**Introduction**: High prices of direct-acting antivirals (DAAs) can prevent access to treatment. Generic versions of sofosbuvir (SOF) are being mass produced for prices under 1% of the current U.S. retail price. Under UK and Australian law, individual patients have the legal right to import 3 months of treatment for hepatitis C virus (HCV), for their personal use. This analysis assessed the efficacy and safety of generic DAAs legally imported into countries where treatment access is limited.

**Methods**: SOF, ledipasvir (LDV) and daclatasvir (DCV) were imported from generic companies into Europe, Australia and North America. Selection of DAAs and treatment duration depended on baseline HCV genotype and fibrosis stage. Initial generic supplies were evaluated using high precision liquid chromatography (HPLC) and nuclear magnetic resonance (NMR) to evaluate presence of active drug. Patients taking generic DAAs were evaluated pretreatment, and at weeks 2, 4, 12 and then for SVR4 and 12. Adverse events were recorded. This analysis includes data from 448 patients, whose imported treatment was organized from the FixHepC website.

**Results**: Of the 448 patients treated, 237 received SOF/LDV, 208 SOF/DCV and 3 SOF/RBV. By HPLC and NMR, all imported drugs passed quality control standards for active DAA drugs. Overall, the patients were 57% male with a mean age of 55 years; 66% were genotype 1, 25% genotype 3 and mean baseline HCV RNA was 6.5 log_10_ IU/mL. Based on currently available data, the percentage with HCV RNA <LLOQ was 196/223 (98.9%) at end of treatment (EOT), 192/211 (91%) at SVR4 and 130/144 (90%) at SVR12. Summary baseline and outcome data from the patients given SOF/LDV or SOF/DCV are shown in Table 1.

**Conclusions**: In this analysis, treatment with legally imported generic DAAs achieved SVR4 rates of 93% on SOF/LDV and 89% on SOF/DCV. These SVR rates are comparable to those seen in phase III trials of the same, but more expensive, branded treatments. Mass treatment with legally imported generic DAAs is a feasible, low-cost option where high prices prevent access to branded treatment.

**Table 1:** HCV RNA undetectability rates for generic DAAs

	SOF/LDV	SOF/DCV
HCV RNA	N=237	N=208
Treatment naïve (%)	123/237 (52)	130/208 (63)
Cirrhosis (%)	52/237 (23)	79/208 (39]
Genotype 1 (%)	215/237 (91)	80/208 (39)
**HCV RNA <25 IU/mL**		
Week 12/EOT (%)	180/182 (99)	171/173 (99)
SVR4 (%)	104/112 (93)	85/96 (89)

# Sub article: P257 Long-term virologic and serologic response of chronic hepatitis B virus infection to tenofovir disoproxil fumarate-containing regimens in HIV-positive patients

**Published:** 2016-10-23

**Authors:** Yu-Shan Huang, Yi-Ching Su, Wen-Chun Liu, Sui-Yuan Chang, Wang-Huei Sheng, Hsin-Yun Sun, Szu-Min Hsieh, Yu-Chung Chuang, Chien-Ching Hung, Shan-Chwen Chang

**Affiliations:** 1 Internal Medicine, National Taiwan University Hospital Hsin-Chu Branch, Hsin-Chu, Taiwan; 2 Internal Medicine, National Taiwan University Hospital, Taipei, Taiwan; 3 Laboratory Medicine, National Taiwan University Hospital, Taipei, Taiwan

## Abstract

**Introduction**: Tenofovir disoproxil fumarate (TDF) combined with lamivudine (LAM) or emtricitabine are the recommended nucleos(*t*)ide reverse-transcriptase inhibitors backbone for patients co-infected with HIV and hepatitis B virus (HBV). TDF leads to rapid decline of HBV DNA. However, data regarding the durability of HBV suppression of TDF-containing cART in HIV/HBV co-infected patients are scarce in hyperendemic area of chronic HBV infection. This study aimed to assess the long-term virologic response of HBV to TDF-containing cART in HIV-positive patients in Taiwan where the prevalence of chronic HBV infection was estimated 15–20% in persons born before nationwide neonatal HBV vaccination programme was implemented in 1986.

**Methods**: Between 2004 and 2016, 186 HIV/HBV co-infected patients with baseline HBV DNA >1000 copies/mL were included and followed for 5 years or longer. Serial blood samples were collected for determinations of plasma HBV DNA load, HBV serologic markers (HBsAg, anti-HBs, HBeAg, and anti-HBe) and liver and renal functions after initiation of cART with or without TDF. Factors associated with undetectable HBV DNA at 5 years of treatment were explored by logistic regression.

**Results**: Of 186 HIV/HBV co-infected patients included, 53 received cART which contained LAM as the only therapy for HBV, 58 switched to TDF-containing cART after detection of resistance-associated mutations of HBV to LAM (n=40) or an elevation of HBV DNA load (n=18) and 75 received TDF-containing cART as initial anti-HBV therapy. The percentages of HBV viral suppression at year 1 and year 5 were 64% and 73.7%, respectively, in patients receiving LAM monotherapy for HBV, 76.4% and 89.7%, respectively, in patients switching to TDF-containing cART, and 86.1% and 100%, respectively, in patients receiving TDF-containing regimens as their first cART. In multivariate analysis, the only factor associated with failure to achieve viral suppression at 5 years was higher HBV DNA load at baseline (adjusted odds ratio (AOR), per 1−log_10_ copies/mL increase, 1.722; 95% CI 1.094–2.711, p=0.019). TDF exposure was of borderline statistical significance (AOR 0.183; 95% CI 0.028–1.193, p=0.076) in the analysis. During study period, 10 of 46 patients (21.7%) with baseline HBeAg positivity had HBeAg seroconversion and loss of HBsAg was observed in four patients (2.2%).

**Conclusions**: TDF-containing cART achieved durable HBV viral suppression in HIV/HBV co-infected patients. A higher HBV DNA load at baseline was associated with failure to achieve HBV viral suppression after long-term TDF-containing cART.

# Sub article: P258 The emergence of hepatitis C virus genotype 4d infection in men who have sex with men

**Published:** 2016-10-23

**Authors:** Liam Sutcliffe, Emma Thomson

**Affiliations:** 1 School of Medicine, University of Glasgow, Glasgow, UK; 2 MRC Centre for Virus Research, University of Glasgow, Glasgow, UK

## Abstract

**Introduction**: Since 2000, there have been numerous reported cases of acute hepatitis C virus (HCV) in HIV-positive men who have sex with men (MSM) in developed countries [1]. The majority of these men deny any history of injecting drug use (IDU), making sexual transmission the most likely route of transmission. HCV genotype 4 is increasing in prevalence amongst MSM populations [2], but is subject to less research than other HCV genotypes. This study aims to use a variety of phylogenetic approaches and evolutionary analysis to determine the origins, spread and changing epidemiology of HCV genotype 4d (HCV-4d).

**Methods**: NS5B region sequences were obtained for patients with HCV-4d using the Los Alamos HCV Sequence Database and NCBI GenBank. Most of these patients were also co-infected with HIV. For each sequence, the sample collection date, country and most likely route of HCV infection were recorded. Sequences were aligned and maximum likelihood phylogenetic trees created with 1000 bootstrap replicates. Molecular clock analysis was performed using the Bayesian Markov chain Monte Carlo (MCMC) approach.

**Results**: The records of 193 individuals (n=193) were reviewed (See Figure 1 on page 259). In the maximum likelihood tree, a distinct cluster of 70 sequences from MSM from four European countries was noted. An MSM cluster was noted containing 12 sequences from the UK, with a 4.8% pairwise distance from the nearest other sequence. A strongly supported homologous pair of genetically similar Dutch sequences was also noted, one from an injecting drug user (IDU) and one from an MSM who reports IDU. Molecular clock analysis identified six HCV-4d clusters comprising mostly IDU sequences, and two MSM-specific clusters. The estimated year of origin of the IDU clusters ranged from 1982 to 1994, whilst the MSM clusters were estimated to have originated in 1995 and 1999. In the IDU clusters, 65% of lineage splits occurred before 1996. In the MSM clusters, 83% of lineage splits occurred after 2000. The UK-specific MSM cluster appears to originate following a lineage split from a strain of HCV-4d circulating in IDUs in Southern Europe in the late 1980s to early 1990s.

**Conclusion**: Incidental transfer of HCV-4d strains from IDUs and rising viral transmission in highly connected MSM populations has led to the formation of international MSM-specific transmission networks. Increased investment in public health interventions is required to slow this expanding epidemic.

**Abstract P258–Figure 1:** Molecular clock analysis of the 193 sequences from the Group 1 maximum likelihood phylogenetic tree. Produced using a Bayesian Markov Chain Monte Carlo (MCMC) approach. Branches are coloured by location; AT – Austria, CN – China, DE – Germany, ES – Spain, FR – France, GB – United Kingdom, IT – Italy, NL – Netherlands, PT – Portugal, US – United States of America. The MSM and IDU clusters identified from this analysis are labelled on the figure. The sequences obtained from a Dutch MSM who also reports injecting drug use is indicated by a red arrow.

## References

[1] van de Laar TJ, Pybus O, Danta M (2009). Evidence of a large, international network of international hepatitis C virus transmission in HIV-positive men who have sex with men. Gastroenterology.

[2] de Bruijne J, Schinkel J, Prins M, Koekkoek SM, Aronson SJ, van Ballegooijen MW (2009). Emergence of hepatitis C virus genotype 4: phylogenetic analysis reveals three distinct epidemiological profiles. J Clin Microbiol.

# Sub article: P259 SOF/VEL single-tablet regimen in HCV mono-infected and HIV/HCV co-infected patients: comparison of efficacy and safety data from phase 3 clinical trials

**Published:** 2016-10-23

**Authors:** David Wyles, Norbert Brau, Susanna Naggie, Mark Sulkowski, Kosh Agarwal, Keyur Patel, Nezam Afdhal, Lin Liu, Xiao Ding, Kuan-Chieh Huang, Anu Osinusi, John McNally, Diana Brainard, Michael Mertens, John McHutchison

**Affiliations:** 1 Division of Infectious Diseases, University of California, San Diego, CA, USA; 2 Department of Medicine, Icahn School of Medicine, James J Peters VA Medical Center, New York, NY, USA; 3 Duke Clinical Research Institute, Duke University Medical Center, Durham, NC, USA; 4 Viral Hepatitis Center, Johns Hopkins University School of Medicine, Baltimore, MD, USA; 5 Institute of Liver Studies, King's College Hospital, London, UK; 6 Division of Gastroenterology, Duke University Medical Center, Durham, NC, USA; 7 Hepatology, Beth Israel Deaconess Medical Center, Boston, MA, USA; 8 Biostatistics, Gilead Sciences, Foster City, CA, USA; 9 Clinical Research, Gilead Sciences, Foster City, CA, USA; 10 Medical Affairs, Gilead Sciences, London, UK

## Abstract

**Introduction**: Twelve-week therapy with the single-tablet regimen (STR) of sofosbuvir/velpatasvir (SOF/VEL) has demonstrated high efficacy in genotypes 1 to 6 HCV-infected patients. Astral-5 clinical trial completed the phase 3 program with the analysis of 12-week SOF/VEL regimen in HIV/HCV co-infected patients. Previous SOF-based combinations showed a comparable safety and efficacy profile in both HCV mono-infected and HCV/HIV co-infected individuals. In order to confirm these data also for SOF/VEL, we compared data obtained in Astral-5 trial with safety and efficacy results of HCV mono-infected individuals produced by Astral 1, 2 and 3.

**Methods**: Astral-5 study enrolled treatment-naïve and -experienced HCV/HIV co-infected patients of all HCV genotypes, with or without cirrhosis. Patients who were on stable ARV regimens with fully suppressed HIV RNA received SOF/VEL (400 mg/100 mg daily) for 12 weeks. Patients were on a wide range of ARV regimens including emtricitabine/tenofovir disoproxil fumarate or abacavir/lamivudine with a backbone of raltegravir, cobicistat/elvitegravir, rilpivirine, ritonavir-boosted atazanavir, darunavir or lopinavir. Astral 1, 2 and 3 phase 3 trials enrolled treatment-naïve and treatment-experienced genotype 1 to 6 HCV-infected patients, with and without cirrhosis, no limit of BMI and no limit of age. Patients received SOF/VEL for 12 weeks.

**Results**: A total of 106 HIV/HCV co-infected patients were enrolled and treated with SOF/VEL for 12 weeks. Overall 86% were male, 45% were black, 77% had IL28B non-CC genotypes, 29% had prior treatment failure (primarily pegIFN/RBV) and 16% had compensated cirrhosis. The genotype distribution in HIV/HCV patients was 62% GT1a, 11% GT1b, 10% GT2, 11% GT3 and 5% GT4. The median baseline CD4 count was 548 cells/µL (range 183–1513 cells/µL) with a median estimated glomerular filtration rate of 97 mL/min (range 57–198 mL/min). Boosted protease inhibitor regimens were the most commonly used regimen (47%). No patient experienced confirmed HIV virologic rebound (HIV-1 RNA ≥400 copies/mL). A total of 1035 HCV mono-infected individuals were treated with a 12-week SOF/VEL regimen in Astral 1, 2 and 3 trials. Cirrhotic patients represented the 21% (n=220) of the total population, and 291 patients (28%) failed a previous anti-HCV treatment. The genotype distribution was 20% GT1a, 12% GT1b, 23% GT2, 27% GT3, 11% GT4, 3% GT5 and 4% GT6. IL28B non-CC genotype was present in 77% of the patients. Efficacy and safety outcomes of mono- and co-infected patients, including complete SVR12, HIV parameters and the impact of HCV resistance variants on outcome will be presented.

# Sub article: P260 HIV/hepatitis C co-infected patients are significantly more complex to manage than HIV mono-infected patients in a large cohort of treatment-naïve, HIV-positive individuals

**Published:** 2016-10-23

**Authors:** Douglas Dieterich, Jennifer Fusco, Cassidy Henegar, Ronald D'Amico, Kathy Schulman, Susan Zelt, Philip Lackey

**Affiliations:** 1 Division of Liver Diseases, Mount Sinai Health System, New York, NY, USA; 2 Epidemiology, Epividian, Inc., Durham, NC, USA; 3 US Medical Affairs, ViiV Healthcare, Inc., Research Triangle Park, Durham, NC, USA; 4 Health Outcomes, Outcomes Research Solutions, Inc., Waltham, MA, USA; 5 US Health Outcomes, ViiV Healthcare, Inc., Research Triangle Park, Durham, NC, USA; 6 ID Consultants & Infusion Care Specialists, Carolinas Healthcare System, Charlotte, NC, USA

## Abstract

**Introduction**: HCV co-infection is common in HIV populations due to shared modes of transmission. Evaluating differences between HIV only and HIV/HCV populations may lead to earlier identification, more effective management and better clinical outcomes.

**Materials and methods**: Using the OPERA database, a collaboration of caregivers at 79 clinics in 15 states, HIV+ individuals initiating HIV antiretroviral therapy for the first time between 1 January 2007 and 31 March 2015 were identified. Patients were followed from HIV treatment initiation to discontinuation of regimen, loss to follow-up, death or study end (31 March 2016). Demographics and clinical characteristics were compared between HIV/HCV co-infected and HIV-only patients using Pearson chi-square or Wilcoxon rank-sum tests. Differences in time to HIV viral load (VL) suppression (<50 copies/mL) with and without HCV were assessed using multivariable Cox proportional hazards regression.

**Results**: Of 9190 HIV+ treatment-naïve patients, 7837 (85.3%) were HCV Ab- and 472 (5.1%) were HCV+ by diagnosis, VL or treatment. Additionally, 881 (9.6%) had a history of HCV clearing or no testing prior to baseline and were excluded. Comparing HCV/HIV co-infected patients with HIV-only patients, co-infected patients were significantly (p<0.0001) older (median age: 46.7 vs. 34.0 years), had a lower proportion of males (76.9% vs. 86.6%) and were less likely to be Hispanic (16.9% vs. 25.5%) or men who have sex with men (39.8% vs. 59.8%). HIV/HCV patients had more comorbidities (CVD, cancer, endocrine, renal, neuropathy and hypertension) and higher pill burden for their HIV and non-HIV medications. Co-infected patients were twice as likely to present with mental illness (25.6% vs. 13.4%), and a third had documented substance addiction (32.0% vs. 13.7%). Baseline CD4 counts were lower for HIV/HCV patients (279 vs. 330 cells/µL) with no difference between groups in baseline HIV VL. HIV treatment was less successful in the HIV/HCV patients, both in achieving suppression (HIV/HCV: 55.5% vs. HIV: 65.4%, p<0.0001) and avoiding rebound (HIV/HCV: 12.3% vs. HIV: 9.4%, p=0.0376) during initial treatment. In crude and adjusted models, those with HCV co-infection were significantly less likely to suppress their HIV VL to undetectable during their first regimen (Chr 0.784 (95% CI 0.693–0.888), Ahr 0.825 (0.726–0.938)).

**Conclusions**: HIV/HCV co-infected patients differ significantly from HIV-only patients. They tend to be older, have more comorbidities and have more complex lifestyles. Strategies to simplify their HIV care (lower pill burden) and avoid complications (reduce drug–drug interactions) in order to incorporate HCV treatment and continue treatment of comorbid conditions will be especially important in this population.

# Sub article: P261 Efficacy of generic direct-acting antiviral treatment for hepatitis C, imported into Russia and Eastern Europe

**Published:** 2016-10-23

**Authors:** Andrew Hill, Julia Dragunova, Roxanna Korologou-Linden, Sergey Golovin

**Affiliations:** 1 St Stephen's AIDS Trust, Chelsea and Westminster Hospital, London, UK; 2 International Treatment Preparedness Coalition, St Petersburg, Russian Federation; 3 Faculty of Medicine, Imperial College London, London, UK

## Abstract

**Introduction**: Russia and Eastern Europe are not included in voluntary licence agreements, and prices of direct-acting antivirals (DAAs) in Eastern Europe are very high. An increasing number of individuals in Russia/Eastern Europe are treating their hepatitis C virus (HCV) infection with generic drugs produced in India or Egypt. It is legal to import DAAs for personal use. This analysis assessed the efficacy of generic DAAs legally imported into Russia/Eastern Europe.

**Methods**: Generic versions of sofosbuvir (SOF), ledipasvir (LDV) and daclatasvir (DCV) were sourced from generic suppliers in India and Egypt. The choice of DAAs and the length of treatment were determined based on baseline HCV genotype and stage of fibrosis. Patients taking generic DAAs were evaluated pretreatment, and at week 4 (rapid virological response – RVR), Week 12 or end of treatment (EOT), and then for sustained virologic response (SVR) 4, 12 and 24. This analysis includes available data from 179 patients being monitored in infectious disease and state university hospitals throughout Russia, Belarus, Ukraine, Spain, Colombia, Israel and Estonia. Patients are monitored by state university hospitals, private doctors, infectious disease hospitals, local AIDS centres and online patient Facebook groups such as the Gepatitka group.

**Results**: Of the 179 patients treated, 42 received SOF/LDV, 136 SOF/DCV and 1 patient received SOF/RBV. The backbone of their generic DAA treatment, SOF, was mainly from Indian generic companies: Hetero (54%), Natco (13%), Mylan (8%) and Zydus (7%). Overall, the patients were 57% male with a mean age of 36.5 years; 40% were genotype 1, and mean baseline HCV RNA was 6.34 log_10_ IU/mL. A RVR was observed in 70% (26/37) of the patients treated with SOF/DCV, 82% (9/11) of the patients treated with SOF/LDV. Based on currently available data, the percentage with HCV RNA <LLOQ was 93% (37/40) at EOT and 16/16 (100%) at SVR 4. EOT responses were similar for patients treated with SOF/LDV (92%) and SOF/DCV (93%).

**Conclusions**: In this analysis, treatment with legally imported generic DAAs achieved high rates of HCV RNA undetectability at EOT, and SVR in all 16 patients evaluated so far. Mass treatment with the current generic DAAs is a feasible and economical alternative route of accessing curative DAAs, where the high prices for branded DAAs prevent access to treatment.

# Sub article: P262 High sustained virologic response rates using generic direct-acting antiviral treatment for hepatitis C, imported into Southeast Asia

**Published:** 2016-10-23

**Authors:** Roxanna Korologou-Linden, Andrew Hill, Aditya Wardhana, Giten Khwairakpam

**Affiliations:** 1 Faculty of Medicine, Imperial College London, London, UK; 2 St Stephen's AIDS Trust, Chelsea and Westminster Hospital, London, UK; 3 Indonesian, AIDS Coalition, Jakarta, Indonesia; 4 amfAR, The Foundation for AIDS Research, Treat Asia, Bangkok, Thailand

## Abstract

**Introduction**: Global elimination of hepatitis C virus (HCV) would be feasible only if prices of direct-acting antivirals (DAAs) are affordable for mass treatment programs. Several countries in Southeast Asia are not included in voluntary license agreements, and prices of DAAs in Southeast Asia are high. An increasing number of individuals in Southeast Asia are treating their HCV infection with generic drugs produced in India. This analysis assessed the efficacy of generic DAAs imported into Southeast Asia.

**Methods**: Generic versions of sofosbuvir (SOF), ledipasvir (LDV) and daclatasvir (DCV) were sourced from generic suppliers in India. The choice of DAAs and the length of treatment were determined based on baseline HCV genotype and stage of fibrosis. Patients taking generic DAAs were evaluated pre-treatment, and at weeks 4 and 12 during treatment and then for sustained virologic response (SVR) 4, 12 and 24. This analysis includes available data from 62 patients being monitored in regional medical institutes and national university hospitals throughout Singapore, Vietnam, Thailand, Indonesia and India.

**Results**: Of the 62 patients treated, 22 received SOF/LDV, 15 SOF/DCV and 25 SOF/RBV. The backbone of combination DAA therapy, SOF, was predominantly from Indian generic companies: Cipla (40%), Zydus (21%), Hetero (13%), Natco (8%) and Mylan (8%). Overall, the patients were 87% male with a mean age of 46 years; 59% were genotype 1, and mean baseline HCV RNA was 6.6 log_10_ IU/mL. Based on currently available data, the percentage with HCV RNA <LLOQ was 100% (33/33) at the end of treatment (EOT) and 100% (23/23) at SVR 4 (Table 1). All of the patients with data at EOT, irrespective of their type of treatment, achieved undetectable HCV RNA levels.

**Conclusions**: In this analysis, treatment with legally imported generic DAAs achieved excellent rates of HCV RNA undetectability at the EOT, and SVR in all 23 patients evaluated so far. The efficacy observed is similar to phase 3 trials of the branded medicines. Mass treatment with the current generic DAAs is a feasible and economical alternative route of accessing curative DAAs, where the high prices for branded DAAs prevent access to treatment.

**Table 1:** Summary baseline and outcome data for patients treated with SOF/LDV, SOF/DCV or SOF/RBV

HCV RNA	SOF/LDV (N = 22)	SOF/DCV (N = 15)	SOF/RBV (N = 25)
% cirrhosis	33% (6/18)	33% (4/12)	32% (7/22)
% genotype 1	91% (20/22)	14% (2/14)	56% (14/25)
% genotype 2	–	7% (1/14)	–
% genotype 3	4.5% (1/22)	79% (11/14)	28% (7/25)
% genotype 4	–	–	8% (2/25)
% genotype 6	4.5% (1/22)	–	–
% genotype 2,6	–	–	4% (1/25)
% genotype 3,4	–	–	4% (1/25)
HCV RNA <25 IU/mL			
RVR	86% (6/7)	100% (5/5)	75% (3/4)
Week 12/EOT	100% (12/12)	100% (8/8)	100% (13/13)
SVR	100% (9/9)	100% (4/4)	100% (10/10)

SOF, sofosbuvir; LDV, ledipasvir; DCV, daclatasvir; RBV, ribavirin; RVR, rapid virological response; SVR, sustained virologic response; EOT, end of treatment.

# Sub article: P263 Is ART use associated with increased risk of ALT elevation in HIV/HCV co-infected patients over and above what is expected in HIV mono-infected? A nested case-control analysis

**Published:** 2016-10-23

**Authors:** Milensu Shanyinde, Giulia Marchetti, Raffaele Bruno, Francesca Ceccherini-Silberstein, Andrea Gori, Andrea De Luca, Jacopo Vecchiet, Miriam Lichtner, Giordano Madeddu, Paolo Bonfanti, Antonella D'Arminio Monforte, Alessandro Cozzi-Lepri, Study Group on behalf of Icona Foundation

**Affiliations:** 1 Infection and Population Health, University College London, London, UK; 2 Health Sciences, Clinic of Infectious Diseases, Milan, Italy; 3 Infectious Diseases, University of Pavia Fondazione, Pavia, Italy; 4 Experimental Medicine and Surgery, University of Rome Tor Vergata, Rome, Italy; 5 Infectious Diseases Clinic, San Gerardo Hospital/University of Milano-Bicocca, Monza, Italy; 6 Medical Biotechnologies, Siena University Hospital, Siena, Italy; 7 Clinic of Infectious Diseases, University of Chieti, Chieti, Italy; 8 Clinic of Infectious Diseases, La Sapienza University, Rome, Italy; 9 Unit of Infectious Diseases, Clinical and Experimental Medicine, University of Sassari, Sassari, Italy; 10 Infectious Diseases, Azienda Ospedaliera Lecco, Lecco, Italy; 11 Health Sciences, San Paolo Hospital, Milan, Italy

## Abstract

**Introduction**: ART-induced toxicity has been frequently reported in HIV/HCV co-infected individuals. However, there is conflicting evidence on whether HCV co-infection has a synergistic effect on ART-induced toxicity. One way to evaluate this hypothesis is to compare the risk of alanine aminotransferase (ALT) elevation associated with the use of ART in HIV mono-infected versus HIV/HCV co-infected populations.

**Materials and methods**: We selected individuals in the ICONA Foundation study cohort with at least one ALT measurement and known current HCV status. We designed a case-control analysis nested in the cohort. Cases were defined as individuals who showed liver enzyme elevation (LEE) >5 x upper limit normal at their last clinical observation; controls were participants who showed normal liver enzyme levels over the same calendar time after enrolment in the cohort. Controls were matched by a predefined set of potential confounders: age (<20, 21–25 and 26–30 to >65), CD4 count cells/mm^3^ (<350, 351–500 and >501), HIV RNA viral load copies/mL (<1000 and 1001–5000 to >100,000) and mode of HIV transmission. A conditional logistic regression model was used to evaluate the associations between ART exposure and risk of LEE in a univariable model adjusted for matching factors and after further controlling for gender, nationality, alcohol use, smoking status and calendar year of enrolment. Interaction between HIV/HCV co-infection status and ART exposure were also formally assessed.

**Results**: We included 2061 (n=687 cases) individuals of whom 70% were males with median calendar year of last clinical visit in 2014 (IQR 2007–2015). Median age was 35 (IQR 31–40) and CD4 count was 386 (IQR 188–586), matched in cases and controls. Proportion of HIV/HCV co-infected individuals was higher in cases than controls 39 and 29%, respectively (p<0.001). Proportion of ART use was higher in cases than controls 79 and 72%, respectively (p<0.001). In the model without interaction, ART use was associated with an increased risk of LEE (adjusted odds ratio 1.87 (95% CI 1.38–2.52; p<0.001)) independently of all factors shown in footnote of Table 1. In the multivariable model, the association between ART use and risk of LEE was 2.37 (95% CI 1.36–4.12) in HIV/HCV co-infected individuals and 1.84 (95% CI 1.24–2.73) in HIV mono-infected (p=0.60).

**Conclusions**: Using a nested case-control study approach, we found no evidence that ART use has a synergistic effect with HIV/HCV co-infection on the risk of ALT elevation. These results are consistent with those obtained in other studies including those of a previous analysis of this cohort.

**Abstract P263–Table 1:** Multivariable conditional logistic regression models for ALT elevation

	Elevated ALT N = 687 (%)	No elevated ALT N = 1374 (%)	Total N = 2061 (%)	Unadjusted OR (95% CI); p	aAdjusted OR (95% CI); p
HCV status (time-dependent)					
HCV negative	418 (60.8)	971 (70.7)	1389 (67.4)	1.00	1.00
HCV positive	269 (39.2)	403 (29.3)	672 (32.6)	2.89 (2.10–3.97); <0.001	2.95 (2.09–4.16); <0.001
ART status (time-dependent)					
ART naïve	144 (21.0)	389 (28.3)	533 (25.9)	1.00	1.00
On ART	543 (79.0)	985 (71.7)	1528 (74.1)	1.82 (1.39–2.37); <0.001	1.87 (1.38–2.52); <0.001

a Besides HCV status and ART use, the model was further adjusted for age, CD4 cell count, HIV RNA viral load, mode of HIV transmission, gender, nationality, alcohol use, smoking status and calendar year of enrolment.

# Sub article: P264 Long-term trends in HCV treatment uptake, efficacy and liver disease in the SHCS

**Published:** 2016-10-23

**Authors:** Charles Béguelin, Annatina Sutter, Enos Bernasconi, Dominique Braun, Helen Kovari, Heiner Bucher, Marcel Stoeckle, Mathias Cavassini, Mathieu Rougemont, Patrick Schmid, Andri Rauch

**Affiliations:** 1 Department of Infectious Diseases, Bern University Hospital and University of Bern, Bern, Switzerland; 2 Division of Infectious Diseases, Regional Hospital Lugano, Lugano, Switzerland; 3 Division of Infectious Diseases, University Hospital Zurich, Zürich, Switzerland; 4 Institute for Clinical Epidemiology & Biostatistics, University Hospital Basel, Basel, Switzerland; 5 Department of Infectious Diseases, University Hospital Basel, Basel, Switzerland; 6 Division of Infectious Diseases, University Hospital Lausanne, Lausanne, Switzerland; 7 Division of Infectious Diseases, University Hospital Geneva, Geneva, Switzerland; 8 Division of Infectious Diseases, Cantonal Hospital St. Gallen, St. Gallen, Switzerland

## Abstract

**Introduction**: Interferon-free hepatitis C virus (HCV) therapies with second-generation direct-acting antiviral agents (DAAs) are highly effective and well tolerated. For that reason they have the potential to substantially increase treatment eligibility and efficacy in HIV-infected patients. We assessed the impact of DAAs on treatment uptake, efficacy as well as its impact on liver disease burden in the Swiss HIV Cohort Study (SHCS).

**Materials and methods**: We prospectively collected data on all SHCS participants who started HCV therapy since January 2009. HCV treatment uptake and efficacy as well as the stage of liver fibrosis was compared between three different time periods: period 1, January 2009 to August 2011 (prior to the availability of DAAs); period 2, September 2011 to March 2014 (first-generation DAAs); period 3, April 2014 to December 2015 (second-generation DAAs).

**Results**: Treatment uptake (4.5/100 patient years (py), 5.7/100 py and 22.4/100 py) and efficacy ((SVR 12): 54%, 70% and 90%) continuously increased through the different periods (Figure 1). Treatment uptake increased across all HCV genotypes in period 3. At the beginning of the third period, 876 SHCS participants had a chronic HCV infection and of those, 186 started HCV therapy with a second-generation DAA. Eighty-eight of 98 patients who reached the end of follow-up, and from whom complete data were already available, achieved an SVR: three patients died (two of liver decompensation and one of sepsis), four had viral relapses (three SOF/RBV and one SOF/LDV), one had a virologic breakthrough (SOF/RBV) and two were lost to follow-up. The majority of treated patients were Caucasian (95%) male (77%) PWIDs (59%) on ART (96%) with advanced liver fibrosis (69% with F4). Patients treated for HCV-genotype 1 during period 3 had significantly higher liver fibrosis stages (44/61, 72% with F4) than those treated during period 2 (21/58, 36% with F4). The proportion of SHCS participants remaining to be treated with liver cirrhosis declined during the last two periods from 18% to 10%.

**Conclusions**: The introduction of interferon-free second-generation DAA treatments in the SHCS increased treatment uptake and efficacy across all HCV genotypes. Because of treatment priorities and limitations in reimbursement, most patients treated with second-generation DAAs had advanced fibrosis or cirrhosis. The treatment of these patients was made possible because of the favourable safety profile of new drugs, and resulted in a significant reduction of the number of cirrhotic patients with replicating HCV infection in the SHCS.

**Abstract P264–Figure 1:** Treatment uptake and efficacy over the 3 periods.

# Sub article: P265 Effectiveness of all-oral DAAs for HCV genotype 4 in HIV/HCV co-infected subjects with compensated liver disease: real-world experience from the MADRID-CoRe study

**Published:** 2016-10-23

**Authors:** Juan Gonzalez-Garcia, Teresa Aldámiz-Echevarría, Carmen Quereda, Victor Hontañon, Lourdes Dominiguez-Dominguez, Angela Gil-Martin, Encarnacion Cruz-Martos, Maria Tellez, Ignacio Santos, Guillermo Cuevas-Tascon, Gabriel Gaspar, Beatriz Alvarez-Alvarez, Juan Losa, Miguel Cervero, Carlos Barros, Raquel Mosalvo, M Antonia Menendez, Luis Belda, Maria Calvo, Marta Alcaraz, Inmaculada Jarrin, Juan Berenguer

**Affiliations:** 1 Internal Medicine/Infectious Diseases, Hospital Universitario La Paz/IdiPAZ, Madrid, Spain; 2 Infectious Diseases, Hospital General Unversitario Gregorio Marañon, Madrid, Spain; 3 Infectious Diseases, Hospital Ramon y Cajal, Madrid, Spain; 4 Internal Medicine/Infectious Diseases, Hospital Doce de Octubre, Madrid, Spain; 5 Pharmacy, Servicio Madrileño de Salud, Madrid, Spain; 6 Internal Medicine/Infectious Diseases, Hospital Clinico San Carlos, Madrid, Spain; 7 Internal Medicine/Infectious Diseases, Hospital de la Princesa, Madrid, Spain; 8 Internal Medicine, Hospital Infanta Leonor, Madrid, Spain; 9 Internal Medicine, Hospital de Getafe, Madrid, Spain; 10 Internal Medicine/Infectious Diseases, Fundacion Jimenez Diaz, Madrid, Spain; 11 Internal Medicine/Infectious Diseases, Fundacion Hospital de Alcorcon, Madrid, Spain; 12 Internal Medicine/Infectious Diseases, Hospital Severo Ochoa, Madrid, Spain; 13 Internal Medicine/Infectious Diseases, Hospital Universitario de Mostoles, Madrid, Spain; 14 Internal Medicine, Hospital del Tajo, Madrid, Spain; 15 Internal Medicine/Infectious Diseases, Hospital Gomez Ulla, Madrid, Spain; 16 Internal Medicine, Hospital del Escorial, Madrid, Spain; 17 Public Health, Instituto de Salud Carlos III, Madrid, Spain

## Abstract

**Introduction**: We evaluated therapeutic outcomes of all-oral direct-acting antivirals (DAAs) for HCV genotype 4 (GT4) in HIV/HCV co-infected patients with compensated liver disease.

**Methods**: The Madrid co-infection registry (MADRID-CoRe) is a prospective registry of all co-infected adults (≥18 years) undergoing DAA therapy (Rx) for HCV in hospitals from the Madrid Regional Health Service (SERMAS). We assessed SVR12, viral relapse and failure due to Rx discontinuation. Between November 2014 and May 2016, 2402 co-infected individuals have initiated DAA Rx within MADRID-CoRe. Herein, we present data of co-infected patients with GT4 and compensated liver disease with programmed Rx finalization censored to 31 December 2015.

**Results**: We evaluated 243 co-infected individuals who met the inclusion criteria. DAA regimens used included (1) sofosbuvir/ledipasvir (SOF/LDV) 190 patients (181 without ribavirin (RBV) (8 weeks 2, 12 weeks 123 and 24 weeks 56) and nine with RBV (12 weeks 8 and 24 weeks 1)); (2) ombitasvir/paritaprevir/ritonavir (OBV/PTV/r) 33 patients (one without RBV for 12 weeks and 32 with RBV (12 weeks 24 and 24 weeks 8)); (3) simeprevir/sofosbuvir (SMV/SOF) 10 patients (eight without RBV (12 weeks six and 24 weeks eight) and two with RBV (12 weeks one and 24 weeks one)); (4) daclatasvir/sofosbuvir (DCV/SOF) 10 patients (seven without RBV (12 weeks one and 24 weeks six) and three with RBV (12 weeks two and 24 weeks one)). Patients’ characteristics and treatment outcomes categorized by DAA regimens are shown in Table 1.

**Conclusions**: High effectiveness was found with LDV/SOF and OBV/PTV/r for GT4 in co-infected patients with compensated liver disease. Small sample size and very high liver stiffness preclude any conclusion about the effectiveness of SMV/SOF and DCV/SOF.

**Abstract P265–Table 1:** Baseline characteristics and outcome in patients coinfected with HIV and HCV genotype 4 treated with all-oral direct-acting antivirals for HCV

	LDV/SOF	OBV/PTV/r	SMV/SOF	DCV/SOF
Baseline variables	N = 190	N = 33	N = 10	N = 10
Age, median (IQR)	51 (47–54)	51 (46–53)	52 (47–54)	50 (48–53)
Male, n (%)	146 (76.8)	24 (72.7)	8 (80.0)	6 (60.0)
Cart, n (%)	183 (96.3)	30 (90.9)	8 (80.0)	8 (80.0)
Log HCV RNA, median (IQR)	6.3 (5.9–6.7)	6.0 (5.8–6.6)	6.3 (6.0–6.6)	6.0 (5.5–6.5)
Cirrhosis, n (%)	76 (40.0)	8 (24.2)	9 (90.0)	9 (90.0)
Liver stiffness, median (IQR)	11.0 (8.1–18.6)	9.5 (8.4–12.3)	48.0 (25.7–66.4)	31.2 (20.9–48.0)
HCV-naïve, n (%)	108 (56.8)	11 (33.3)	4 (40.0)	6 (60.0)
Outcomes				
SVR12, n (%)	177 (93.2)	32 (97.0)	5 (50.0)	6 (60.0)
% SVR, 95% CI	88.6–96.3	84.2–99.9	18.7–81.3	26.2–87.8
Relapse, n (%)	10 (5.3)	1 (3.0)	5 (50.0)	2 (20.0)
Discontinuation, n (%)	3 (1.5)	0	0	2 (20.0)

SOF, sofosbuvir; LDV, ledipasvir; DCV, daclatasvir; SVR, sustained virologic response; OBV, ombitasvir; PTV/r, paritaprevir/ritonavir; SMV, simeprevir.

# Sub article: P266 Continued increase of recent hepatitis C virus infections amongst HIV-positive patients in Taiwan

**Published:** 2016-10-23

**Authors:** Wen-Chun Liu, Li-Hsin Su, Cheng-Hsin Wu, Pei-Ying Wu, Shang-Pin Yang, Jun-Yu Zhang, Hsi-Yen Chang, Sui-Yuan Chang, Cheng-Hua Liu, Hsin-Yun Sun, Chien-Ching Hung, Shan-Chwen Chang

**Affiliations:** 1 Internal Medicine, National Taiwan University Hospital, Taipei, Taiwan; 2 Center of Infection Control, National Taiwan University Hospital, Taipei, Taiwan; 3 Laboratory Medicine, National Taiwan University Hospital, Taipei, Taiwan

## Abstract

**Introduction**: We previously have shown that the rate of recent hepatitis C virus (HCV) infection in HIV-positive patients seeking HIV care at the National Taiwan University Hospital (NTUH), Taipei, had increased from 0 in 1994–2000 and 2.29 in 2001–2005 to 10.13 per 1000 person-years of follow-up (PYFU) in 2006–2010. This study aimed to investigate whether the increasing trend of recent HCV infection continued between 2011 and 2015.

**Materials and methods**: Between January 2011 and December 2015, HIV-positive patients seeking care at the NTUH were prospectively observed, and serologic tests for HCV were provided at baseline during their first visit and subsequently on an annual basis or to those who acquired syphilis or had elevated aminotransferases according to the national HIV treatment guidelines. Antibodies to HCV were determined with a third-generation enzyme immunoassay (Ax SYM HCV III; Abbott Laboratories, North Chicago, IL). HCV RNA load was determined, and HCV was genotyped. Recent HCV seroconversion was defined as the first positive anti-HCV detected within 1 year after the last negative anti-HCV. The date of seroconversion was assigned as the midpoint between the date of the last negative and that of the first positive anti-HCV result. All patients were followed until 30 April 2016.

**Results**: During the 5-year study period, 3483 HIV-positive patients aged 15 years or older sought HIV care at NTUH. After excluding 29 patients without anti-HCV data at baseline and 371 testing positive for HCV (prevalent HCV infections), 3083 were included for prospective follow-up. A total of 140 (4.5%) had recent HCV infection (incident HCV infections) during a total observation duration of 9900.51 PYFU, giving an overall incidence rate of 14.14 per 1000 PYFU. The rate was 12.64, 13.81, 13.27, 11.77 and 18.55 per 1000 PYFU in 2011, 2012, 2013, 2014 and 2015, respectively. Compared with 2695 patients without HCV seroconversion, patients with recent HCV seroconversion were more likely to be male (100.0% vs. 96.1%, p=0.009), younger (mean age, 32.4 vs. 35.9 years, p=0.0004) and men who have sex with men (85.7% vs. 78.4%, p=0.007) and to have recent syphilis (37.1% vs. 11.1%, p<0.0001). The mean plasma HCV RNA load was 6.07 log_10_ copies/mL. Of the 76 HCV strains submitted for genotyping, genotype 1 accounted for 40.8%.

**Conclusions**: The increasing trend of recent HCV infection continued in HIV-positive patients seeking HIV care at the university hospital in Taiwan from 2011 to 2015.

# Sub article: P267 Treatment failure with DAAs in chronic hepatitis C: a Portuguese multicentre report

**Published:** 2016-10-23

**Authors:** Cristina Valente, M Isabel Casella, Domitilia Faria, M João Aleixo, Inês Vaz Pinto, Margarida Mota, Amparo Mingo, Alexandre Carvalho, Fausto Roxo, Rita Faísca, Paula Proença, José Poças, Margarida Prata, Nuno Marques, José Saraiva Cunha

**Affiliations:** 1 Infectious Diseases, Centro Hospitalar Universitário Coimbra, Coimbra, Portugal; 2 Infectious Diseases, Hospital de São Bernardo, Setúbal, Portugal; 3 Immunodeficiency Unit, Centro Hospitalar Algarve, Portimão, Portugal; 4 Infectious Diseases, Hospital Garcia de Orta, Almada, Portugal; 5 HIV Unit, Hospital Cascais, Cascais, Portugal; 6 Immunodeficiency Unit, Centro Hospitalar Gaia, Gaia, Portugal; 7 Infectious Diseases, Hospital Faro, Faro, Portugal; 8 Immunodeficiency Unit, Hospital Braga, Braga, Portugal; 9 Immunodeficiency Unit, Hospital Santarém, Santarém, Portugal; 10 Infectious Diseases, Hospital Cova Beira, Covilhâ, Portugal

## Abstract

**Introduction**: Either in clinical trials or in real-life studies, cure rates with oral direct-acting antivirals (DAAs) in the treatment of chronic hepatitis C virus (HCV) are greater than 90%. However, as these drugs become more widely used, clinicians are beginning to report occasional cases of treatment failure.

**Materials and methods**: GEPCOI is a multicentre group that involves several sites in Portugal. For this study, 10 centres participated, and all cases of DAA failure were collected. Treatment failure was considered in case of relapse, virologic failure during treatment, discontinuation or death. All co-infected HIV/HCV patients that started oral DAA drugs were included in this analysis.

**Results**: A total of 573 chronic hepatitis co-infected patients started a course of oral DAAs since the beginning of 2015. All but 23 patients (4%) achieved sustained virologic response: 16 (2.8%) had a relapse or failure during treatment, 2 discontinued treatment and 5 died. All except one were male (95.6%) with an average age of 47 years. They were naïve (65.2%) or null responders (21.7%) and relapsers (4%) in previous treatment with pegIFN+ribavirin (RBV). All patients were under ART, and only two had detectable viremia. Only three patients changed ART schedule due to HCV treatment (pIs to IIs and TDF/FTC to ABC/3TC). Regarding HCV, genotype 1 was the predominant (60.8%) and the degree of fibrosis was respectively: F0–F2, 21.7%; F3; 21.7%; and F4, 56.5%. Those who have relapse or virologic failure were G1 10, G2 two, G3 three and G4 one. All HCV G1/4 were treated with SOF/LDV+RBV 12 weeks or 24 weeks without RBV. In all, G2/3 SOF+RBV was used, except in one patient classified initially as G1, who completed 12 weeks with asunaprevir+daclatasvir+RBV (G3). Deaths (five) occurred during or after the end of treatment, due to hepatic decompensation or related complications: they were all F4, with a MELD score ranging from 9 to 27. All of these patients had both albumin level <3.5 gr/dL and platelets <100,000 mcL.

**Conclusions**: Failures in this real-life study occurred in 4%, with relapses or virologic failures accounting for 70% of the total. They were mainly patients with advanced fibrosis (F3/F4 74.9%). Those who died were cirrhotic, and all of them had both low level of albumin and platelets. HCV drugs did not have any impact on HIV outcome at the end of treatment. Treatment in co-infected patients with advanced disease should have a very close monitoring.

# Sub article: P268 Seroepidemiology of hepatitis A virus among HIV-positive patients in Taiwan in the setting of acute hepatitis A outbreak from 2015 to 2016

**Published:** 2016-10-23

**Authors:** Guan-Jhou Chen, Kuan-Yin Lin, Hsin-Yun Sun, Wang-Huei Sheng, Szu-Min Hsieh, Yu-Chung Chuang, Chien-Ching Hung, Shan-Chwen Chang

**Affiliations:** 1 Department of Internal Medicine, National Taiwan University Hospital, Taipei, Taiwan

## Abstract

**Introduction**: Since June 2015, an ongoing outbreak of acute hepatitis A virus (HAV) infection is occurring amongst men who have sex with men (MSM) in Taiwan, with more than 440 cases reported to the Taiwan Centres for Disease Control as of 30 June 2016. This study aimed to describe the seroepidemiology of HAV infection among HIV-positive patients in northern Taiwan, where three-fourths of the patients in the outbreak reside.

**Materials and methods**: We reviewed the medical records of HIV-positive patients seeking HIV care at the National Taiwan University Hospital, Taipei. Information on demographics and clinical characteristics was collected, which included age, risk group for HIV transmission, plasma HIV RNA load, CD4 count and serologies of hepatitis viruses and syphilis at baseline and during follow-up, and dates of HAV vaccination administered. A case-control study was conducted to identify the associated factors with acute HAV infection. Case patients were those who received a diagnosis of acute HAV infection between June 2015 and 2016, and four controls were identified that were matched with case patients by age (±5 years), HIV risk factor and similar observation duration.

**Results**: During the study period, 2029 HIV-positive patients, with a mean age of 38.4 years and 83.3% being MSM, had baseline HAV serologic data and 33.6% (n=682) tested seropositive for HAV. The HAV seroprevalence was 15.4% in those aged <35 years. As of June 2016, 52.9% (n=713) of HAV-seronegative patients were vaccinated with HAV vaccine by following the current recommendations of vaccination for adults, and most of the patients (681; 95.5%) received HAV vaccines only after the outbreak was taking place. Among the unvaccinated individuals, a total of 37 patients, all being MSM, developed acute HAV infection during the follow-up, giving an incidence rate of 4.76 cases per 100 person-years of follow-up as of 30 June 2016. In the case-control study, acute HAV infection was significantly associated with recent syphilis that had occurred within 3 months of acute HAV infection, with an adjusted odds ratio of 2.3 (95% CI 1.3–3.2).

**Conclusions**: With a low HAV seroprevalence among HIV-positive MSM aged <35 years in Taiwan, the adherence to HAV vaccination in this at-risk group was low before this HAV outbreak. In the outbreak, patients with acute HAV infection in our cohort were significantly associated with recent syphilis, suggesting risky sexual behaviour contributing to this acute HAV outbreak in the at-risk group in Taiwan.

# Sub article: P269 Different impact of DAA on innate and adaptive cellular immunity in HIV/HCV co-infected and HCV mono-infected patients

**Published:** 2016-10-23

**Authors:** Paola Zuccalà, Miriam Lichtner, Serena Vita, Raffaella Marocco, Claudia Mascia, Stefano Savinelli, Michela Campagna, Fabio Mengoni, Tiziana Tieghi, Irene Pozzetto, Gabriella D'Ettorre, Francesca Paoletti, Claudio Maria Mastroianni, Vincenzo Vullo

**Affiliations:** 1 Department of Public Health and Infectious Diseases, Sapienza University of Rome, Rome, Italy

## Abstract

**Introduction**: HIV/HCV co-infected patients have higher HCV loads and generally more rapid progression to fibrosis, end-stage liver disease and death. HIV and HCV viral infections are both characterized by systemic immune activation that plays an important role in disease progression. In the direct-acting antiviral (DAA) era, little is known about the immune-pathological response in HCV mono-infected and in HCV/HIV co-infected patients. The aim of the study was to analyze activation of T lymphocytes, DCs and Mo subsets in HCV and HCV/HIV patients under effective ART that receiving anti-HCV therapy.

**Material and methods**: In our study, we assessed 75 samples from 26 patients (13 HCV/HIV patients under effective ART and active HCV replication and 13 HCV patients) undergoing IFN-free regimens DAA based. Samples were collected before starting anti-HCV therapy (TO) and 12 weeks after the end of treatment when they obtained a sustained virologic response (SVR 12). Fourteen healthy donors (HD) were used as controls. We analyzed whole blood samples evaluating mDC, pDC, slanDC and typical, atypical and intermediate monocytes with a cytofluorimetric method based on seven fluorochromes. HLA-DR/CD38 CD4 and CD8 lymphocytes were also evaluated. Liver fibrosis was measured using FibroScan and FIB-4 score. ANOVA with Dunn's test, Mann–Whitney test, Wilcoxon test and Spearman correlation test were used for statistical analysis.

**Results**: All patients in both groups obtained SVR12. Activation of CD8 T cells was significantly higher in HIV/HCV and HCV patients than control (p=0.0002 and p=0.0041, respectively). Interestingly, a decrease in both groups was found (comparing SVR12 in HIV/HCV and HCV patients to HD, p=0.0186 and p=0.0479, respectively) up to a normalization after anti-HCV therapy. HLA-DR/CD38 CD4 levels were elevated only in co-infected patients (p=0.0003) without modification during therapy (comparing SVR to control p=0.0385). Intermediate Mo were increased in patients with HCV infection compared to HD (p=0.0654) and normalized after therapy. Considering the sub-population of DCs, Mdc and pDC were reduced only in HIV/HCV patients (p<0.001 and p<0.01, respectively), and normalize after therapy, while MDC8 were decreased both in HIV/HCV and HCV patients compared to HD (p<0.001 and p<0.01, respectively); this decreases persist after therapy.

**Conclusions**: A different pattern of immune dysfunction was found in HIV/HCV co-infected and HCV mono-infected subjects. IFN-free treatments seem to reverse some of these alterations that should be monitored with a longer follow-up.

# Sub article: P270 Real-life renal impact of ledipasvir/sofosbuvir on a cohort of HIV-infected patients treated with tenofovir combined with a boosted protease inhibitor

**Published:** 2016-10-23

**Authors:** João Cabo, Ana Raquel Garrote, Pedro Sim[otilde]es, Diana Póvoas, Rita Gonçalves, Freddy Ramirez, Stepanka Betkova, Diana Seixas, Liliana Alves, Maria José Manata, Nidia Garrido, Orlando Cardoso, Sara Cardoso, Sara Lino, Teresa Martins, Fernando Maltez

**Affiliations:** 1 Infectious Diseases Department, Hospital Curry Cabral – Centro Hospitalar Lisboa Central, Lisboa, Portugal

## Abstract

**Introduction:** Regarding the treatment of HIV/HCV co-infected patients, we still have some concerns about the renal safety of the interaction between ledipasvir (LDV)/sofosbuvir (SOF) and an ARV regimen including tenofovir and a boosted protease inhibitor (PI). Data are lacking from clinical trials to support this co-administration, since these patients were excluded from the main studies in co-infected patients. Increased levels of tenofovir and risk of renal impairment might prompt preventive ARV therapy switch recommendations, not possible in all patients due to their history of previous ARV regimens.

**Methods**: An observational study was conducted among the co-infected on an ARV regimen including tenofovir and boosted PI, who started HCV treatment with DAAs, between 1 January 2015 and 20 May 2016. Data on demographic, clinical and virological features were collected by analysis of clinical files.

**Results**: A total of 149 patients were treated with tenofovir/emtricitabine, from which 68 with SOF/LDV and an antiretroviral regimen that included a boosted PI: 30 on DRV/r, 22 on LPV/r, 14 on ATV/r, 1 on SQV/r and 1 on FPV/r. Mean age was 47 years, 72% males. Regarding HIV infection, 85% of the patients had undetectable viral load (<20 copies/mL), ranging below 100 copies/mL in the remaining, with a median of 582 T CD4+ cells/µL (141–1570). Treatment was planned for 24 weeks in 35% (24) of patients, according to liver fibrosis stage. At baseline, four patients had CKD stage IIi (Mean EgFR 54 mL/min) and by week 8 three of them had their FTC/TDF regimen switched (Mean EgFR 44 mL/min). By the end of treatment, these three patients had egfR >60 mL/min. Only one of the remaining 64 patients presented with an egfR <60 mL/min during treatment, requiring no switch on ARV regimen. From the 48 patients with available data at week 12 post-treatment, all but one had sustained virological response.

**Conclusion**: In our study, SOF/LDV did not have a major negative impact in patients on TDF and a boosted PI. In this population, renal function must be carefully monitored, mainly in patients with other risk factors for renal impairment.

# Sub article: P271 Changes in lipid profile during and after hepatitis C virus (HCV) treatment with direct-acting antiviral (DAA), interferon-free regimens in patients co-infected with HIV

**Published:** 2016-10-23

**Authors:** Lourdes Domínguez-Domínguez, Otilia Bisbal, María Lagarde, Mariano Matarranz, Asunción Hernando, Rafael Rubio, Federico Pulido

**Affiliations:** 1 HIV Unit, Instituto de Investigación Hospital 12 de Octubre, Madrid, Spain; 2 Facultad de Ciencias Biomédicas, Universidad Europea de Madrid, Villaviciosa de Odón, Spain

## Abstract

**Introduction:** HCV infection is associated with lower lipid levels in HIV co-infected patients treated or not with ART [1]. This has been related both to the HCV infection and to the impairment of the liver function. Some studies have reported increased lipid values after sustained virological response (SVR) with therapy based on interferon (INF) [2]. We aim to evaluate lipid changes in HIV/HCV co-infected patients receiving all-oral HCV therapy.

**Methods**: Retrospective longitudinal study in a cohort of HIV/HCV co-infected patients treated with direct-acting antiviral (DAA) and INF-free therapy. All patients whose treatment finished on 30 December 2015 or before, and whose 24-week post-treatment evaluation was on or before 15 June 2016, were included. The values of triglycerides (TG), total cholesterol (TC), high-density lipoprotein (HDL) cholesterol (HDL-c) and low-density lipoprotein (LDL) cholesterol (LDL-c) were collected at three time points: (1) 12 weeks before starting DAAs (pre-treatment); (2) between week 4 and the final day of treatment (on-treatment); (3) weeks 12 to 24 after the end of HCV therapy (post-treatment). Means were compared with the repeated measures ANOVA test. Results have been adjusted by a general linear regression which includes the following variables: age, gender, basal HCV RNA, presence of a protease inhibitor (PI) drug in the DAA regimen, presence of a PI drug in the ART regimen and estimated liver fibrosis (cirrhosis has been considered when liver stiffness ≥14.6 kPa).

**Results**: Two hundred and fifty patients had reached week 24 post-treatment on 15 June 2016. Only 130 patients had available lipid data before, during and after HCV therapy. Table 1 shows baseline characteristics. SVR was achieved in 127 patients (97.7%). TC and LDL-c values statistically increased on and after treatment (p<0.001) versus pre-treatment. There were no significant changes when comparing TC and LDL-c values on versus after-treatment, nor between TG and HDL-c values pre-treatment versus on-treatment or post-treatment (Table 2). Changes in TC and LDL-c values are not influenced by gender (p=0.55 and p=0.86, respectively), age (p=0.07 and p=0.06), basal HCV RNA (p=0.21 and p=0.1), presence of PI in the ART regimen (p=0.50 and p=0.46) nor cirrhosis (p=0.41 and p=0.19). Moreover, changes between LDL-c values are not influenced by the presence of PI in the DAA regimen (p=0.18), but DAA regimens including a PI were associated with increased TC values (p=0.005).

**Conclusion**: TC and LDL-c values increase during the HCV treatment using DAA, INF-free regimens, and remain increased after stopping the HCV therapy.

**Table 1:** Baseline characteristics

Age, mean (SD)	50.9 (5.5)
Gender: male/female, n (%)	93 (71.5)/37 (28.5)
Risk group: PWID/heterosexual/MSM, n (%)	113 (86.9)/13 (10.0)/4 (3.1)
CDC (93) classification system's C stage, n (%)	48 (36.9)
Basal HCV RNA, medIAn (UI/mL) (IQR)	2,047,181.5 (3,879,143.0)
Prior INF-based therapy: none/failure/relapse/intolerance, n (%)	79 (60.8)/35 (26.9)/8 (6.2)/8 (6.2)
Liver fibrosis assessed by transient elastography: F0–1, F2, F3, F4, n (%)	1 (0.8)/54 (41.5)/14 (10.8)/61 (46.9)

MSM, men who have sex with men; PWID, people who inject drugs.

**Abstract P271–Table 2:** Means of the values in mg/dL with standard deviation

	Pre-treatment	On-treatment	After-treatment	p*	p†	p‡
TG	156.4 (79.7)	142.7 (68.1)	161.5 (85.2)	0.073	1.000	0.011
TC	176.0 (37.9)	199.5 (55.0)	196.1 (51.9)	<0.001	<0.001	1.000
HDL-c	49.3 (20.5)	52.0 (23.4)	49.9 (18.4)	0.100	1.000	0.751
LDL-c	97.6 (37.1)	121.1 (46.8)	114.2 (44.7)	<0.001	<0.001	0.107

Statistical significance p-value established in 0.05. Bonferroni correction has been done.

* compares values pre-therapy versus on-treatment

† compares pre-therapy versus after-treatment

‡ compares on-treatment versus after-treatment.

TG, triglycerides; TC, total cholesterol; HDL-c, HDL cholesterol; LDL-c, LDL cholesterol.

## References

[1] Cooper CL, Mills E, Angel JB (2007). Mitigation of antiretroviral-induced hyperlipidemia by hepatitis C virus co-infection. AIDS.

[2] Lange CM, von-Wagner M, Bojunga J, Berg T, Farnik H, Hassler A (2010). Serum lipids in European chronic HCV genotype 1 patients during and after treatment with pegylated interferon-a-2a and ribavirin. Eur J Gastroenterol Hepatol.

# Sub article: P272 HIV/hepatitis C co-infection: successfully treating hepatitis C with direct-acting antivirals and managing those who do not access traditional care

**Published:** 2016-10-23

**Authors:** Rebecca Metcalfe, Naomi Bulteel, Joe Schofield, Patricia McGinness, Claire Glover, Lorna McLean, Fiona Marra, Aliceann Murphy, Erica Peters, David Bell, Ray Fox, Emma Thomson

**Affiliations:** 1 Brownlee Centre, NHS Greater Glasgow and Clyde, Glasgow, UK; 2 Public Health, NHS Greater Glasgow and Clyde, Glasgow, UK

## Abstract

**Introduction**: In our HIV/hepatitis C virus (HCV) co-infected cohort, we are successfully treating HCV with direct-acting antivirals (DAAs) regardless of genotype, regimen, disease stage or prior treatment exposure. However, we recognize a proportion of patients for whom we are unable to provide treatment, because they do not engage in the traditional care setting. We report on the efficacy and safety of DAA therapy in our cohort of HIV/HCV co-infected individuals, the demographics of those not engaging in care and the strategies employed to tackle this population.

**Methods**: All patients co-infected with HIV and HCV in our cohort were included, and case notes were reviewed. Those who spontaneously cleared HCV infection, transferred care or died were excluded.

**Results**: At May 2016, the HIV/HCV co-infected cohort comprised 181 patients, of whom 89 (49%) had commenced HCV treatment. Thirty-three of these patients were treated successfully with interferon and ribavirin. Fifty-seven patients received ≥1 dose second-generation DAA, including 20 patients with cirrhosis, six in clinical trials. The majority were male (46/57) with a history of injecting drug use (35/57). The majority were HCV genotype 1 infected (48/57). Most were treatment naïve (43/57); six prior null responders; four relapsers after previous IFN/RBV; none were DAA experienced. Fifty-five of 57 were on a suppressive HIV antiretroviral regimen. At the time of writing, 52/57 patients had reached end of treatment. Forty-two had achieved SVR12 (42/42, 100%). Despite high success rates with those engaged in care, 92 (51%) patients remain untreated, of whom the majority are not attending scheduled hospital appointments, and many are currently struggling with addictions. Some are recently diagnosed as part of an ongoing outbreak of HIV and HCV amongst people who inject drugs. To target this population, we are implementing service change. New strategies will include local pharmacy “directly observed therapy” dispensing, specialist nurse-led service in the community and in addiction services. We show the area of residence of those who have over 50% non-attendance rates, in relation to the hospital where care is traditionally delivered to highlight the need for local services.

**Conclusions**: In those who access care, we observe excellent SVR rates in HIV-infected patients receiving DAAs for HCV. Serious adverse events with DAAs are rare and delivering treatment in the community to difficult-to-treat populations will increase engagement in HIV care and HCV cure rates. Poor engagement in care should be tackled by service redesign to reach out to these populations.

# Sub article: P273 Improving of glycaemic control associated with DAAs HCV treatment persists at SVR12

**Published:** 2016-10-23

**Authors:** Paolo Pavone, Gabriella d'Ettorre, Miriam Lichtner, Tiziana Tieghi, Raffaella Marocco, Ivano Mezzaroma, Giulia Passavanti, Claudio Maria Mastroianni, Vincenzo Vullo

**Affiliations:** 1 Public Health and Infectious Diseases, Sapienza University, Rome, Italy; 2 Infectious Diseases, Sapienza University, Latina, Italy; 3 Statistics, Sapienza University, Rome, Italy

## Abstract

**Introduction**: Association between HCV infection and insulin resistance and type 2 diabetes has been widely postulated. Our group already reported a rapid improving of glycaemic control associated with DAAs [1]. Aim of our study was to evaluate if improving of glycaemic control persists after the end of DAAs treatment.

**Materials and methods**: We retrospectively evaluated 39 HCV-infected patients (10 HIV+) with type 2 diabetes who were treated with different IFN-free regimens, including sofosbuvir, simeprevir, ledipasvir, daclatasvir, dasabuvir and ombitasvir/paritaprevir/ritonavir. To evaluate general improving of glycaemic control, we investigated for reduction of fasting glucose (FG) or glycated haemoglobin (A1C) or modification of insulin/metformin dosing during and after anti-HCV treatment. Statistical analysis was performed with the paired t-test, Kruskal-Wallis test and Welch one-way ANOVA procedure (R software).

**Results**: The mean age of the patients was 60 years (32 M, 7 F). The HCV genotypes were different but with type 1 prevalence (n=24). All the patients had HCV RNA undetectable at end of treatment (<15 UI/mL, if still on treatment). CD4 were above 14%, and HIV RNA was undetectable in all patients. Pretreatment FG was reported in 38 patients, mean value 168 mg/dL (min. 81, max. 455), pretreatment A1C in 22 patients, mean value 7% (min. 5.1%, max. 11.8%). Nine patients (24%) needed to reduce or stop (n=2) hypoglycaemic drugs. One patient with basal A1C value of 11.8% needed to stop insulin treatment and is off-therapy with A1C <5% at SVR 12. Three patients needed to increase insulin dosing, one of these patients died after HCV relapse. Their FG and A1C values were excluded from analysis. FG values during treatment were available for 35 patients, and analysis showed a statistically significant reduction (p=0.01), reduction mean value (mv) was −27 mg/dL; at SVR12, FG values were available in 18 patients, with a reduction mv of 44.5 mg/dL (p=0.0031). A1C during treatment was available for 17 patients, and analysis showed a statistically significant reduction (p=0.01), reduction mv was −1.14%; at SVR12, A1C values were present in 12 patients, reduction mv was 0.5% (p=0.09). FG and A1C reductions were not correlated to the drug regimen, HCV genotype, BMI, ALTt0/ALTt1 and HIV status. No cases of symptomatic hypoglycaemia were found.

**Conclusions**: HCV suppression following DAA treatment is often associated with rapid and persisting improving of glycaemic control. Patients undergoing DAAs should be closely monitored for eventual modifications of hypoglycaemic drugs. Eradication of HCV might result in diabetes cure in some patients.

## References

[1] Pavone P, Tieghi T, d'Ettorre G, Lichtner M, Marocco R, Mezzaroma I Rapid improving of glycemic control in HCV patients treated with IFN-free regimens. [Poster 610.] Conference on Retroviruses and Opportunistic Infections (CROI); 2016 Feb 22–25; Boston, MA, USA.

# Sub article: P274 Ledipasvir/sofosbuvir for 12 or 24 weeks in HCV genotype 1 in HIV/HCV co-infected subjects with compensated liver disease: real-world experience from the MADRID-CoRe study

**Published:** 2016-10-23

**Authors:** Juan Berenguer, Ana Moreno, Luz Martín-Carbonero, Lourdes Domínguez, Teresa Aldámiz-Echevarría, Ángela Gil-Martín, Encarnación Cruz-Martos, Jorge Vergas, Ignacio Santos, Laura Benítez, Julio De Miguel, Jesús Troya, Beatriz Álvarez-Álvarez, Rafael Torres, Eduardo Canalejo, Sari Arponen, María Teresa de Guzmán, Luis Gotuzzo, María José Calvo, Marta Alcaraz, Inmaculada Jarrin, Juan González-García

**Affiliations:** 1 Infectious Diseases, Hospital General Universitario Gregorio Marañón, Madrid, Spain; 2 Infectious Diseases, Hospital Ramón y Cajal, Madrid, Spain; 3 Internal Medicine, Hospital Universitario La Paz, Madrid, Spain; 4 Internal Medicine, Hospital Doce de Octubre, Madrid, Spain; 5 Pharmacy, Servicio Madrileño de Salud, Madrid, Spain; 6 Infectious Diseases, Hospital Clínico de San Carlos, Madrid, Spain; 7 Infectious Diseases, Hospital Universitario de La Princesa, Madrid, Spain; 8 Internal Medicine, Hospital Universitario Puerta de Hierro, Madrid, Spain; 9 Infectious Diseases, Hospital Principe de Asturias, Alcalá de Henares, Spain; 10 Internal Medicine, Hospital Infanta Leonor, Madrid, Spain; 11 Infectious Diseases, Fundación Jiménez Días, Madrid, Spain; 12 Internal Medicine, Hospital Severo Ochoa, Madrid, Spain; 13 Internal Medicine, Hospital de Fuenlabrada, Madrid, Spain; 14 Internal Medicine, Hospital de Torrejón, Madrid, Spain; 15 Internal Medicine, Hospital Infanta Cristina, Madrid, Spain; 16 Internal Medicine, Hospital Rey Juan Carlos, Madrid, Spain; 17 Public Health, Instituto de Salud Carlos III, Madrid, Spain

## Abstract

**Introduction**: We evaluated therapeutic outcomes LDV/SOF for HCV genotype 1 (GT1) in HIV/HCV co-infected patients with compensated liver disease.

**Materials and methods**: The Madrid co-infection registry (MADRID-CoRe) is a prospective registry of all co-infected adults (≥18 years) undergoing DAA therapy (Rx) for HCV in hospitals from the Madrid Regional Health Service (SERMAS). We assessed SVR12, viral relapse and failure due to Rx discontinuation. Between November 2014 and May 2016, 2402 co-infected individuals have initiated DAA Rx within MADRID-CoRe. Herein, we present data of co-infected patients with GT1a or GT1b and compensated liver disease that were treated with LDV/SOF with or without ribavirin (RBV) for 12 or 24 weeks, and with programmed Rx finalization censored to 31 December 2015.

**Results**: We evaluated 377 co-infected individuals who met the inclusion criteria: 284 infected with GT1a and 93 infected with GT1b. Patients characteristics and treatment outcomes categorized by subtypes, treatment duration and use of RBV are shown in Table 1.

**Conclusions**: High effectiveness and safety were found with LDV/SOF for 12 or 24 weeks in GT1 co-infected patients with compensated liver disease.

**Abstract P274–Table 1:** Patients’ characteristics and treatment outcomes categorized by subtypes, treatment duration and use of ribavirin

	G1a	G1a	G1a	G1a	G1b	G1b	G1b
	
	No-RBV	No-RBV	RBV	RBV	No-RBV	No-RBV	RBV
	
	12 weeks	24 weeks	12 weeks	24 weeks	12 weeks	24 weeks	12 weeks
	
Baseline variables	N = 197	N = 51	N = 33	N = 3	N = 57	N = 22	N = 14
Age years, median	51	51	50	51	50	52	54
Male, %	82.2	78.4	87.9	100	77.2	68.2	64.3
cART, %	97.5	98.0	93.4	100	100	90.9	92.9
Log HCV RNA, median	6.4	6.3	6.3	6.7	6.4	6.0	6.5
Cirrhosis, %	25.4	90.2	90.9	66.7	45.6	100	92.9
Liver stiffness kPa, median	10.0	18.3	20.0	24.0	11.8	20.6	19.8
HCV-naïve, %	67.0	54.9	36.4	0	50.9	10.9	64.3
Outcomes							
SVR12, n (%)	183 (92.9)	50 (98.0)	30 (90.9)	3 (100)	56 (98.2)	20 (90.9)	13 (92.9)
% SVR, 95% CI	88.4–96.1	89.6–99.9	75.7–98.1	–	90.6–99.9	70.8–98.9	66.1–99.8
Relapse, n (%)	7 (3.5)	0	2 (6.1)	0	0	1 (4.5)	0
Breakthrough, n (%)	0	0	0	0	0	0	0
D/C due to AEs, n (%)	1 (0.5)	0	0	0	0	0	0
D/C other reasons, n (%)	6 (3.0)	1 (2.0)	1 (3.0)	0	1 (1.8)	1 (4.5)	1 (7.1)

RBV, ribavirin; SVR, sustained virologic response; D/C, discontinuation; AEs, adverse events.

# Sub article: P275 Seroepidemiology of hepatitis B virus (HBV) infection among HIV-positive men who have sex with men born in the era of nationwide neonatal HBV vaccination in Taiwan

**Published:** 2016-10-23

**Authors:** Yi-Chia Huang, Shang-Ping Yang, Wen-Chun Liu, Pei-Ying Wu, Hsin-Yun Sun, Wang-Huei Sheng, Szu-Min Hsieh, Chien-Ching Hung, Shan-Chwen Chang

**Affiliations:** 1 Department of Internal Medicine, National Taiwan University Hospital, Taipei City, Taiwan; 2 Center of Infection Control, National Taiwan University Hospital, Taipei City, Taiwan

## Abstract

**Introduction**: Previous studies have demonstrated a waning immunity against hepatitis B virus (HBV) 15 years after vaccination in individuals born after 1986 when nationwide HBV vaccination program was implemented in Taiwan [1], where the prevalence of chronic HBV infection was 15 to 20% among those born before 1984. We aimed to assess the HBV seroepidemiology and serologic response to booster vaccination for HBV among HIV-positive men who have sex with men (MSM) born after 1986.

**Materials and methods**: Medical records of HIV-positive MSM who were born after 1986 and sought HIV care at the NTUH between 2000 and 2016 were reviewed, and information on clinical characteristics and antiretroviral therapy containing tenofovir disoproxil fumarate (TDF) and emtricitabine (FTC) or lamivudine (3TC) was collected.

**Results**: During the 16-year study period, 632 HIV-positive MSM were included, with a mean age of 23.0 years. About 83% were receiving cART containing TDF plus FTC or 3TC. Twenty patients (3.2%) had anti-HCV antibody and 197 (31.2%) had syphilis at baseline. Eighty-two patients (13%) were excluded from analysis due to lack of the result of anti-HBc antibody at baseline. Among 550 patients, 18 (3.3%) had chronic HBV infection and 271 (49.3%) had lost HBV seroprotection (anti-hbS <10 mIU/mL). During the follow-up, 135 patients received at least one dose of HBV vaccine and 75 patients (56%) with follow-up of serologic response after vaccination achieved anti-HBs titERS >10 mIU/mL. Among the vaccine-responders, 47 had repeat testing at week 52 of booster vaccination and 22 (47%) had a sustained serologic response. For those who had lost HBV seroprotection at the beginning of this study, three incident cases of HBV infection occurred after 754.7 person-years of follow-up (PYFU), accounting for an incidence rate of 4.0 per 1000 PYFU. Two cases of anti-HBc antibody seroconversion occurred in patients with an anti-HBs titer ≥10 mIU/mL at baseline (incidence rate, 3.0 per 1000 PYFU). During the same period of observation, the incidence of HCV infection and syphilis was 13.4 per 1000 PYFU and 96.4 per 1000 PYFU, respectively.

**Conclusions**: Despite impaired immunity from HIV infection and poor response to HBV booster vaccination, the seroprevalence of chronic HBV infection has significantly declined in the HIV-positive MSM born after 1986. In this high-risk group for acquisition of sexually transmitted infections and with waning immunity against HBV, the risk of incident HBV infection remains low.

## References

[1] Sun HY, Cheng CY, Lee NY, Yang CJ, Liang SH, Tsai MS (2014). Seroprevalence of hepatitis B virus among adults at high risk for HIV transmission two decades after implementation of nationwide hepatitis B virus vaccination program in Taiwan. PLoS One.

# Sub article: P276 Liver fibrosis regression in HIV–HCV co-infected individuals after sustained virologic response with HCV direct-acting antivirals

**Published:** 2016-10-23

**Authors:** Ana Arias, Gemma Lledó, Ana Treviño, Laura Benitez-Gutierrez, Itziar Carrasco, Valentín Cuervas-Mons, Carmen De Mendoza

**Affiliations:** 1 Internal Medicine, Puerta de Hierro Hospital, Madrid, Spain

## Abstract

**Introduction:** The hepatitis C virus (HCV) field has been revolutionized following the introduction of new direct-acting antivirals (DAA). HCV eradication with peginterferon-ribavirin already was shown to be associated with significant improvements in liver function and hepatic fibrosis. Information on the extent of liver fibrosis regression is lacking in HIV-HCV co-infected indivudals, in whom hepatic fibrosis more rapidly develops.

**Methods**: All HIV-HCV co-infected patients treated with DAA at our clinic until March 2016 were examined. Transient elastometry (FibroScan) was performed at baseline and after 12 weeks of completion of therapy, at the time of sustained virologic response (SVR12). Liver fibrosis regression was defined as a shift from advanced fibrosis (METAVIR F3–F4 [>9.5 kPa]) to null-mild fibrosis (F0–F2) and/or >30% reduction on kPa from any baseline value.

**Results**: A total of 50 HIV-HCV co-infected individuals with SVR12 were identified. Overall, 79.2% were male and median age 52 years. Baseline serum HCV RNA was 6.2 Log IU/mL. Distribution of HCV genotypes: G1a (25), G1b (nine), G3 (eight) and G4 (eight). A total of 52.8% of patients had unfavourable IL28B genotypes (CT/TT). Elevated AST/ALT at baseline was seen in 77.4% of patients. Patients were treated with SOF-LED (31), 3D (eight), SOF-DCV (eight) and SOF-SMV (eight). Median length of therapy was 12 weeks. Up to 54% received RBV. At baseline, 65.4% had F3 to F4 being significantly higher in those with IL28B-CC compared with IL28B CT/TT (80% vs. 52%; p=0.044). Mean FibroScan value was 20.1 kPa. At the time of SVR12, a significant regression in liver fibrosis was found in 38.5%. The mean reduction was of 4.81 kPa (p<0.001). A trend towards higher improvement was seen in patients with baseline F3 to F4 compared with F0 to F2 (47% vs. 22%; p=0.08, respectively). No relationship was found between significant improvement in liver fibrosis and IL28B-CC, baseline serum HCV RNA, HCV genotype, DAA treatment modality and ribavirin use.

**Conclusion**: Cure of hepatitis C with DAA in HIV-HCV co-infected patients is associated with significant and rapid improvement in hepatic fibrosis measured by FibroScan. At least 38% of patients experience significant liver fibrosis regression after SVR12. Thus, DAA therapy should be prioritized in the HIV-HCV co-infected population.

# Sub article: P277 Treatment rate for HCV in the DAAs era in HIV co-infected patients: data from an Italian cohort

**Published:** 2016-10-23

**Authors:** Gianluca Cuomo, Cinzia Puzzolante, Cristina Mussini, Vanni Borghi

**Affiliations:** 1 Infectious Diseases, AOU Policlinico di Modena, Modena, Italy; 2 Infectious Diseases, AOU Policlinico di Modena, Università di Modena e Reggio Emilia, Modena, Italy

## Abstract

**Introduction**: New drugs (DAAs) are now effective in HCV infection, and no difference in sustained virological response (SVR) was observed between HCV mono-infected and HIV co-infected patients. Despite major advances in HCV therapy, persons living with HIV (PLHIV) were undertreated. The aim of this study was to describe the management of HCV treatment in an HIV/HCV cohort during a 15-year period.

**Methods**: An electronic chart review of all HIV patients with >1 observation at our department from the year 2000 was made. Demographic, virological and treatment data were collected.

**Results**: From 2000 to 2015, 2353 PLHIV were enrolled; 67.8% were males, median age was 48 years and 19.2% were not Italian. HIV transmission due to intravenous drug use (IDU) was reported in 681 people (28.9%), in 573 (24.4%) was in men who had sex with men (MSM), heterosexual transmission in 1007 (42.8%) and other risk in 92 (3.9%). Seven hundred and ninety-five patients’ (33.8%) result was HCVAb positive. HCV+ patients were mostly Italian (94.3%), IDU in 72.0% and MSM in 6.5%. Only 596 HCV+ patients had detectable HCV RNA in the blood (75%); this mostly related to patients that obtained an SVR before the year 2000. HCV genotypes were tested in 516 patients; 55.8% were genotype 1, 31.8% were 3 subtype; 12.4% were genotype 2 or 4. The percentage of HCVAb patients amongst the total of HIV population significantly decreased from the 49.2% of 2000 to the 30.7% of 2015 (p<0.001). Year-per-year analysis showed the stability of treatment rate for HCV, with a significant increase in 2015 (20.6% vs. 8.5% of 2014, p>0.001). SVR rate was significantly improved in 2015 (79.9% vs. 50% of 2014, p=0.02). The number of re-treatments significantly increased in 2014 and 2015 (58.8% and 61.2% of the total of treatments respectively, p>0.001 vs. 2013). The number of HCVAb+ patients with HCV RNA not relievable (cured) significantly increased during years. At 2015, 50.4% of patients resulted to be HCV RNA negative (18.1% in 2000, p<0.001) (Figure 1).

**Conclusions**: In the DAAs era, a significant increase in the number of treatments was observed. SVR rate was significantly higher. Treatment rate remains quite low maybe related to the availability of DAAs only for patients with advanced liver disease (in Italy). More than half of our population of HCV co-infected patients’ results to be cured for HCV.

**Abstract P277–Figure 1:** Number of HCV treatments (naïve, re-treatment) during the years (from 2000 to 2015), and percentage of HCV-RNA negative patients amongt the HCVAb positive population during the same period.

# Sub article: P278 The elderly and direct antiviral agents (DAAs): constraint or challenge?

**Published:** 2016-10-23

**Authors:** Claudia Fabrizio, Giuseppe Bruno, Eugenio Milano, Raffaele Dell'Acqua, Laura Monno, Sergio Lo Caputo, Michele Milella, Annalisa Saracino, Gioacchino Angarano

**Affiliations:** 1 Clinic of Infectious Diseases, University of Bari, Bari, Italy

## Abstract

**Introduction:** New IFN-free regimens based on direct antiviral agents (DAAs) in patients with HCV-related chronic hepatitis showed high rates of sustained virological response (SVR) and good safety profile. So far, few data are available about the impact of these therapies on elderly patients, who are often not included in clinical trials. Aim of this study was to evaluate the efficacy and safety profile of DAAs in elderly patients.

**Materials and methods:** In this prospective, single-centre observational study, all patients aged ≥65 years, who initiated a DAA-based regimen, were enrolled from February 2015 to May 2016, then divided according to age (group A: 65–74 years; group B: ≥75 years). Baseline clinical, anamnestic and laboratory data were collected (Table 1).

**Results:** In the study period, 289 patients started a DAA-based regimen, including 167 patients (males: 87, 52.1%) aged ≥65 years (group A: 99 patients, 59.3%; group B: 68 patients, 40.7%). The following regimens were administered: sofosbuvir-based: 38 patients (22.7%), simeprevir-based: 25 (15%), ledipasvir-based: 33 (19.8%), daclatasvir-based: three (1.8%), paritaprevir, ombitasvir/ritonavir ± dasabuvir-based: 68 (40.7%). Ribavirin was used in 49 patients (29.3%). In 38 patients (22.8%), an adjustment of comedications was necessary due to drug interactions. Safety was assessed for 134 patients who reached end of treatment (EOT) during the study period (Table 2).

At least one AE occurred in 93 patients (69.4%), of whom seven (5.2%) had serious AEs (World Health Organization grade 3/4). Treatment discontinuation because of AEs occurred in six patients (4.5%), including one death due to oesophageal varices bleeding. The following AEs were observed: neurological/psychiatric symptoms (headache, dizziness, insomnia and mood disorders) (23.9%); skin reactions (23.1%); anaemia with Hb <10 g/dL (12.7%), requiring hospitalization in four patients; gastrointestinal toxicity (12.7%); other AEs (13.4%). The presence of two or more comorbidities and a pill burden of comedications ≥4 were observed in subjects with concurrent AEs, in particular in older patients (group B). A 12-week follow-up after EOT was available for 88 patients, and SVR12 was obtained in all subjects.

**Conclusions:** The heavy burden of comorbidities and comedications in elderly patients complicates the management of DAA-based therapies. However, despite a considerable amount of AEs, drug-related toxicity did not significantly impair the completion and the effectiveness of treatment. Further studies, based on larger populations and prolonged follow-up, are warranted to assess the optimal real-life management of these peculiar patients.

**Abstract P278–Table 1:** Clinical and laboratory baseline features of the 167 enrolled patients

	Tot patients (N=167)	A (65–74 years, N=99)	B (≥75 years, N=68)
Genotype, n° (%)			
1a	2 (1.2)	0 (0.0)	2 (2.9)
1b	95 (56.9)	45 (45.5)	50 (73.5)
2	37 (22.2)	23 (23.2)	14 (20.6)
3	1 (0.6)	1 (1.0)	0 (0.0)
4	6 (3.6)	5 (5.1)	1 (1.5)
Cirrhosis, n° (%)	112 (67.1)	62 (62.6)	50 (73.5)
Previous failure of anti HCV therapy, n° (%)	87 (52.1)	57 (57.6)	30 (44.1)
HCV RNA IU/mL, median (range)	1.296.000 (4.501–27.590.025)	1.302.000 (4.501–17.140.000)	1.150.000 (13.000–27.590.025)
ALT UI/L, median (range)	72 (18–434)	77 (18–368)	63 (15–434)
Total bilirubin mg/dL, median (range)	0.8 (0.2–2.9)	0.8 (0.2–2.9)	0.8 (0.4–2.9)
Serum creatine mg/dL, median (range)	0.83 (0.40–1.66)	0.81 (0.46–1.30)	0.88 (0.40–1.66)
Platelets (×10^9^/L), median (range)	136 (38–347)	154 (50–347)	123 (38–342)
Body mass index, median (range)	25.9 (17.8–38.8)	25.3 (19.3–38.8)	26.5 (17.8–31.2)
Patients with at least 1 comorbidity, n° (%)	138 (82.6)	81 (81.8)	57 (83.8)
Hypertension, n° (%)	101 (60.5)	60 (60.6)	41 (60.3)
Diabetes, n° (%)	38 (22.8)	24 (24.2)	14 (20.6)
Oesophageal varices/portal hypertension, n° (%)	16 (9.6)	5 (5.1)	11 (16.2)
Other comorbidities, n° (%)			
Thyreopathies	13 (7.8)	14 (14.1)	9 (13.2)
Dyslipidemia	18 (10.8)	16 (16.1)	2 (2.9)
Psychiatric disorders	16 (9.6)	10 (10.1)	6 (8.8)
Neoplasms	10 (6.0)	6 (6.1)	4 (5.9)
Cardiopathies	24 (14.4)	12 (12.1)	12 (17.6)
Prostatic hypertrophy	19 (11.4)	8 (8.1)	11 (16.2)
Others	32 (19.2)	18 (18.2)	14 (20.6)
Pill burden of comedications, median (range)	3 (0–14)	3 (0–14)	3 (0–13)

**Abstract P278–Table 2:** Safety profile of the 134 patients who reached the end of treatment

		Tot pts, N:134	A (65–74 years), N:78	B (≥75 years), N:56
Tot pts with any AE, n° (%)		93 (69.4)	56 (71.8)	37 (66.1)
	Tot pts with ≥2 AEs	32 (23.9)	21 (26.9)	11 (19.6)
	Tot pts with serious AE (WHO grade 3/4)	7 (5.2)	2 (2.6)	5 (8.9)
	Tot pts with serious AE leading to therapy discontinuation^a^	6 (4.5)	2 (2.6)	4 (7.1)
	Deaths before EOT	1 (0.7)	0 (0.0)	1 (1.8)
AEs according to sex				
	Male sex	43/65 (66.2)	26/36 (72.2)	17/29 (58.6)
	Female sex	50/69 (72.5)	30/42 (71.4)	20/27 (74.1)
AEs according to comorbidities				
	Pts without comorbidities	19/29 (65.5)	12/17 (70.6)	7/12 (58.3)
	Pts any comorbidity	74/105 (70.5)	44/61 (72.1)	30/44 (68.2)
	Pts with ≥2 comorbidities	52/72 (72.2)	32/41 (78.0)	20/31 (64.5)
	Pts with cirrhosis	65/93 (69.9)	36/51 (70.6)	29/42 (69.0)
	Pts with obesity (BMI ≥30)	15/21 (71.4)	12/18 (66.7)	3/3 (100.0)
AEs according to comedications				
	Pts with no comedications	20/33 (60.6)	15/22 (68.2)	5/11 (45.5)
	Pts with pill burden between 1 and 3	29/43 (67.4)	17/25 (68.0)	12/18 (66.7)
	Pts with pill burden ≥4	44/57 (77.2)	24/30 (80.0)	20/27 (74.1)
Pts with changes in comedications due to drug interactions		19/27 (70.4)	9/14 (64.3)	10/13 (76.9)
AEs according to type of DAA therapy				
	with RBV	65/89 (73.0)	41/54 (75.9)	24/35 (68.6)
	without RBV	28/45 (62.2)	15/24 (62.5)	13/21 (61.9)
of which:				
	SMV based ± RBV	18/25 (72.0)	14/17 (82.4)	4/8 (50.0)
	SOF based + RBV	21/29 (72.4)	13/17 (76.5)	8/12 (66.7)
	LDV based ± RBV	19/25 (76.0)	10/13 (76.9)	9/12 (75.0)
	DAC based + RBV	1/1 (100.0)	1/1 (100.0)	0/0 (0.0)
	3ABT/2ABT based ± RBV	34/54 (63.0)	18/30 (60.0)	16/24 (66.7)

3ABT/2ABT, paritaprevir, ombitasvir/ritonavir ± dasabuvir; AE, adverse event; DAA, direct antiviral agents; DAC, daclatasvir; LDV, ledipasvir; RBV, ribavirin; SMV, simeprevir; SOF, sofosbuvir; EOT, end of treatment.

a discontinuations were due to: skin reaction (one patient), cardiological adverse events (AEs) (one patient), diarrhoea (one patient), oesophageal varices bleeding (one patient) and severe anaemia (two patients).

# Sub article: P279 Treating HCV-positive Italian inmates with direct-acting antivirals: the clinical experience in three major correctional houses of Milan

**Published:** 2016-10-23

**Authors:** Roberto Ranieri, Antonella Foschi, Maddalena Casana, Anna Radice, Cinzia D'Angelo, Anna De Bona, Francesca Bai, Cesare Lari, Antonella d'Arminio Monforte

**Affiliations:** 1 Milano Opera Correctional House, ASST Santi Paolo e Carlo, Milano, Italy; 2 Pharmacy Unit, ASST Santi Paolo e Carlo, Milano, Italy; 3 Clinic of Infectious Diseases, ASST Santi Paolo e Carlo, Milano, Italy; 4 Health Department, ASST Santi Paolo e Carlo, Milano, Italy

## Abstract

Epidemiologic studies report high HCV positivity rates in prisons (3–40%) with high unawareness rates, resulting in a substantial risk of HCV transmission. For this, treating HCV-positive prisoners could impact on both individual and public health. Though international guidelines have emphasized that prisoners must be treated as well as general population, HCV treatment eligibility in prisons has always been suboptimal due to poor adherence, psychiatric comorbidities, side effects and legal issues. The availability of highly effective, short-course DAAs could increase inmates’ treatment opportunities [1,2]. Our study was performed in 2015 in three prisons of Milan (Opera, San Vittore and Bollate) harbouring yearly 3400 prisoners overall. Every new inmate was proposed HIV, HCV, HBV, PPD and syphilis testing. HCV RNA+ prisoners were submitted to an infectious disease visit, HCV genotyping, ultrasonography and fibroscan. Legal issues and patient's motivation were considered. All motivated prisoners with advanced liver fibrosis (F3/F4) and at least 3 months end of sentence were selected for DAA treatment according to Italian guidelines. Three thousand three hundred and fifty-four tests were performed: HCV-Ab positivity was found in 10% (n=314) of inmates. Seventy percent of HCV-Ab+ inmates were IDUs. HCV RNA was detected in 60% of HCVAb+ inmates. Thirty percent of HCV RNA+ patients had advanced liver fibrosis (F3/F4). Sixty percent of them were treated with first- and second-generation DAAs. The main reasons for treatment deferral were transfer to other prison or release (33%), low compliance (19%) and end of sentence <3 months (15%); seven patients (33%) are under evaluation. We treated 25 patients with second-generation DAAs; the higher the fibrosis the higher the rate of treated patients. In Table 1, we report the characteristics of the treated patients. Eighty percent of them were naïve to previous treatments; 16% were HIV co-infected; the main genotypes were 1 (48%) and 3 (44%), child-turcotte-pugh (CTP) score was between A5 and B7. No treatment discontinuations were observed. SVR12 was reached in 83.3% of the treated patients. Two patients relapsed. The relapsers were genotype 3 cirrhotic patients who underwent, in early 2015, a 24-week sofosbuvir + ribavirin regimen that nowadays is considered suboptimal for this category of patients. Our data show high rates of F3/F4 treated patients as compared to previous studies. Inmates showed a strict adherence and satisfaction and none of them discontinued treatment. In conclusion, short-course, highly effective and well-tolerated DAAs are a feasible and strongly recommended HCV treatment strategy in prison settings that could improve both individual and public health.

**Abstract P279–Table 1:** Characteristics of the treated patients and treatment schedule

Opera/San Vittore/Bollate correctional houses	Patients, N (%)
Treated inmates, N	25
Age, years, median (range)	50 (45–56)
History of drug addiction, N (%)	15 (60)
Co-infection with HIV, N (%)	4 (16)
HCV genotype, N (%)	
1a+1b	12 (48)
3	11 (44)
4	2 (8)
CTP classification for cirrhotic patients, N (%)	
A5	14 (56)
A6	3 (12)
B7	4 (16)
MELD, median (range)	8.5 (7–10)
METAVIR fibrosis score, N (%)	
F4	21 (84)
F3	3 (12)
F2	1 (4)
Fibrosis 4 score, N (%)	
< 1.45	1 (4)
1.45–3.25	5 (20)
> 3.25	19 (76)
HCC, N (%)	1 (4)
Previous HCV treatment history, N (%)	
No	20 (80)
Yes	5 (20)
Current HCV treatment duration, N (%)	
12 weeks	11 (44)
24 weeks	14 (56)
HCV treatment schedule, N (%)	
SOF+RBV	6 (24)
SOF+DCV	1 (4)
SOF+LDV	2 (8)
SOF+P/R	1 (4)
SOF+SIM+RBV	4 (16)
SOF+LDV+RBV	5 (20)
SOF+DCV+RBV	4 (16)
3D+RBV	1 (4)
2D+RBV	1 (4)
EOTR, N (%)	21 (87)
SVR12, N (%)	10 (83)
Relapse, N (%)	2 (8)

2D, ombitasvir+paritaprevir/ritonavir; 3D, ombitasvir+paritaprevir+dasabuvir/ritonavir; DCV, daclatasvir; LDV, ledipasvir; P/R, peg-interferon plus ribavirin; RBV, ribavirin; SIM, simeprevir; SOF, sofosbuvir, EOTR, end-of-treatment response; HCC, hepatocellular carcinoma; CTP, child-turcotte-pugh.

## References

[1] Zampino R, Coppola N, Sagnelli C, Di Caprio G, Sagnelli E (2015). Hepatitis C virus infection and prisoners: epidemiology, outcome and treatment. World J Hepatol.

[2] Fazel S, Baillargeon J (2011). The health of prisoners. Lancet.

# Sub article: P280 Effectiveness of dasabuvir, ombitasvir/paritaprevir/ritonavir (DSV+OBV/PTV/r) for HCV genotype 1 in HIV/HCV co-infected subjects with compensated liver disease: real-world experience from the MADRID-CoRe study

**Published:** 2016-10-23

**Authors:** Juan Gonzalez-Garcia, Maria Luisa Montes-Ramírez, Lourdes Dominguez-Dominguez, Teresa Aldámiz-Echevarría, M Jesus Vivancos, Angela Gil-Martin, Encarnacion Cruz-Martos, Vicente Estrada, Ana Arias, Jose Sanz, Gabriel Gaspar, Juan Losa, Carlos Barros, Jose Ruiz-Giardin, Alejandra Gimeno-Garcia, Ana Vegas, M Teresa Garcia-Benayas, Regino Serrano, M Jose Calvo, Marta Alcaraz, Inmaculada Jarrin, Juan Berenguer

**Affiliations:** 1 Internal Medicine/Infectious Diseases, Hospital Universitario La Paz/IdiPAZ, Madrid, Spain; 2 Internal Medicine/Infectious Disease, Hospital Doce de Octubre, Madrid, Spain; 3 Infectious Diseases, Hospital General Universitario Gregorio Marañon, Madrid, Spain; 4 Infectious Diseases, Hospital Ramon y Cajal, Madrid, Spain; 5 Pharmacy, Servicio Madrileño de Salud, Madrid, Spain; 6 Internal Medicine/Infectious Disease, Hospital Clinico Universitario, Madrid, Spain; 7 Internal Medicine/Infectious Disease, Clinica Puerta de Hierro, Madrid, Spain; 8 Internal Medicine/Infectious Disease, Hospital Principe de Asturias, Alcalá de Henares (Madrid), Spain; 9 Internal Medicine, Hospital de Getafe, Madrid, Spain; 10 Internal Medicine/Infectious Disease, Fundacion Hospital de Alcorcon, Madrid, Spain; 11 Internal Medicine/Infectious Disease, Hospital de Mostoles, Madrid, Spain; 12 Internal Medicine, Hospital de Fuenlabrada, Madrid, Spain; 13 Internal Medicine, Hospital de Torrejon, Madrid, Spain; 14 Internal Medicine, Hospital Infanta Elena, Madrid, Spain; 15 Internal Medicine, Hospital del Sureste, Madrid, Spain; 16 Internal Medicine, Hospital del Henares, Madrid, Spain; 17 Public Health, Instituto Salud Carlos III, Madrid, Spain

## Abstract

**Introduction**: We evaluated therapeutic outcomes of DSV+OBV/PTV/r for HCV genotype 1 (GT1) in HIV/HCV co-infected patients with compensated liver disease.

**Methods**: The Madrid co-infection registry (MADRID-CoRe) is a prospective registry of all co-infected adults (≥18 years) undergoing DAA therapy (Rx) for HCV in hospitals from the Madrid Regional Health Service (SERMAS). We assessed SVR12, viral relapse and failure due to Rx discontinuation. Between November 2014 and May 2016, 2402 co-infected individuals have initiated DAA Rx within MADRID-CoRe. Herein, we present data of co-infected patients with GT1 and compensated liver disease with programmed Rx finalization censored to 31 December 2015, who were treated with DSV+OBV/PTV/r with or without ribavirin (RBV).

**Results**: We evaluated 132 co-infected individuals who met the inclusion criteria; 72 infected with GT1a and 60 infected with GT1b. Patient characteristics and treatment outcomes categorized by subtypes, treatment duration and use of RBV are shown in Table 1.

**Conclusions**: High effectiveness and safety were found with DSV+OBV/PTV/r with or without RBV for GT1 in co-infected patients with compensated liver disease.

**Abstract P280–Table 1:** Baseline characteristics and outcome in patients coinfected with HIV and HCV genotype 1 treated with dasabuvir and ombitasvir/paritaprevir/ritonavir+ribavirin

	Genotype 1a	Genotype 1a	Genotype 1a	Genotype 1a	Genotype 1a	Genotype 1b	Genotype 1b	Genotype 1b	Genotype 1b
	
	No RBV	No RBV	RBV	RBV		No RBV	No RBV	RBV	
	
	12 weeks	24 weeks	12 weeks	24 weeks	Total	12 weeks	24 weeks	12 weeks	Total
	
Baseline variables	N=3	N=1	N=42	N=26	N=72	N=37	N=1	N=22	N=60
Age, years, median	51	47	50	51	50	50	50	49	50
Male, %	66.7	100	76.2	92.3	81.9	67.6	0	72.7	68.3
cART, %	100	100	100	100	100	100	100	100	100
Log HCV RNA, median	6.2	5.5	6.5	6.6	6.5	6.5	5.2	6.1	6.3
Cirrhosis, %	66.7	100	4.8	96.2	41.7	13.5	100	86.4	41.7
Liver stiffness kPa, median	14.4	64.0	8.6	18.0	10.4	9.3	17.5	20.4	11.2
HCV naïve, %	66.7	100	52.4	65.4	58.3	67.6	0	45.4	58.3
Outcomes									
SVR12, n (%)	3 (100)	0	40 (95.2)	24 (92.3)	67 (93.1)	34 (91.9)	1 (100)	21 (95.4)	56 (96.3)
% SVR, 95% CI	–	–	83.8–99.4	74.9–99.1	84.5–99.7	78.1–98.3	–	77.2–99.9	83.8–98.2
Relapse, n (%)	0	1 (100)	1 (2.4)	0	2 (2.8)	1 (2.7)	0	1 (4.5)	2 (3.3)
Breakthrough, n (%)	0	0	0	0	0	1 (2.7)	0	0	1 (1.7)
D/C due to AEs, n (%)	0	0	0	1 (3.8)	1 (1.4)	1 (2.7)	0	0	1 (1.7)
D/C other reasons, n (%)	0	0	1 (2.4)	1 (3.8)	2 (2.8)	0	0	0	0

RBV, ribavirin; SVR, sustained virologic response; D/C, discontinuation; AEs, adverse events.

# Sub article: P281 Response to DAA-based regimens in genotype 4 chronic HCV/HIV co-infected patients in real life: COINFECOVA-2-SEICV study

**Published:** 2016-10-23

**Authors:** Carlos Minguez, Miguel García-Deltoro, Juan Flores, Marta Montero, Jorge Carmena, Maria-José Galindo, Sergio Reus, Jose-Emilio Ballester, Jorge Usó, Enrique Ortega, for the Coinfecova-2 Seicv Study Group

**Affiliations:** 1 Internal Medicine/Infectious Diseases Unit, Hospital General Universitario de Castellón, Castellón, Spain; 2 Infectious Diseases Unit, Consorcio Hospital General Universitario de Valencia, Valencia, Spain; 3 Internal Medicine/Infectious Diseases Unit, Hospital Arnau de Vilanova, Valencia, Spain; 4 IInfectious Diseases Unit, Hospital Universitari i Politecnic La Fe, Valencia, Spain; 5 Internal Medicine/Infectious Diseases Unit, Hospital Universitario Dr Peset, Valencia, Spain; 6 Infectious Diseases Unit, Hospital Clinico Universitario, Valencia, Spain; 7 Infectious Diseases Unit, Hospital General Universitario de Alicante, Alicante, Spain

## Abstract

**Introduction and aims:** Because genotype 4 (GT-4) HCV/HIV co-infected patients with advanced liver fibrosis are generally under-represented in clinical trials, and their prevalence in developed countries is low, little data are currently available on use of DAAs in this setting. The aim of this study was to assess in the clinical practice, the efficacy and safety of interferon-free DAA therapy in GT-4 HCV co-infected patients.

**Methods:** COINFECOVA-2 is an observational, multicentre study accomplished in hospitals of a region of eastern Spain, including co-infected patients treated with all oral DAA on routine practice. GT-4 HCV/HIV co-infected patients were included in this analysis, if interferon-free DAA therapy was initiated before 1 September 2015 (24-week regimen) or before 1st December 2015 (12-week regimen), allowing a sufficient follow-up to evaluate efficacy. Epidemiologic and clinical data were retrospectively collected by their responsible physicians and investigators.

**Results:** We included 102 patients in 14 outpatient clinics with a median age of 50 years (IQR 46–54), 74% men and 94% on antiretroviral therapy. HIV viral load was <50 copies/mL in 82%, median CD4+ nadir was 184 cells/mm^3^ (IQR 99–299) and mean CD4+ at baseline 682 cells/mm^3^ (95% CI 603–761). Fifty-six percent of the patients were naïves to HCV therapy, 47% were cirrhotic and 22% had an F3 degree of fibrosis (METAVIR). Fifty percent of cirrhotic had a liver stiffness (LS) measured by elastometry ≥21 kPa. The employed combinations of DAA with or without ribavirin (RBV) included: sofosbuvir (SOF)/ledipasvir (LDV) (59%); ombitasvir (OMB)/paritaprevir/ritonavir (PTV/r) (20%); SOF+simeprevir (SMV) (14%); and SOF+daclatasvir (DCV) (7%). RBV was employed in 32% of the patients, and therapy was scheduled to 24 weeks in 5%. Efficacy results are displayed in Table 1. There were five patients with treatment failure (four relapses and one breakthrough), and two losses of follow-up. Adverse events (AEs) were reported in 29 patients (28%), three of which were serious (3%), and mainly related to anaemia in patients treated with RBV (13/33 patients treated with RBV had anaemia). No patient stopped therapy for AEs.

**Conclusions:** In a real-world setting, this cohort of GT-4 co-infected patients, mostly with an advanced liver fibrosis, presented a rate of SVR over 90%, excepted for cirrhotic patients with high LS (≥21 kPa) who tended to have a worse response, especially if treated with SOF/LDV. Results in cirrhotic patients with LS <21 kPa are similar to patients with F3 degree of fibrosis.

**Abstract P281–Table 1:** SVR stratified by treatment regimens and degree of fibrosis

	SOF/LDV±RBV (N=60)	OMB/PTV/r+RBV (N=20)	SOF+SMV±RBV (N=14)	SOF+DCV±RBV (N=7)	Overall (N=102)^a^
	
	N (%)	N (%)	N (%)	N (%)	N (%)
All degrees	56/60 (93)	20/20 (100)	13/14 (93)	5/7 (71)	95/102 (93)
Fibrosis 3	14/14 (100)	9/9 (100)	3/3 (100)	1/2 (50)	27/28 (96)
Cirrhosis	26/30 (87)	6/6 (100)	7/8 (87)	2/3 (67)	41/47 (87)
Cirrhosis LS<21 kPa	15/15 (100)	2/2 (100)	0/1^b^ (0)	2/2 (100)	19/20 (95)
Cirrhosis LS≥21 kPa	11/15 (73)	4/4 (100)	6/6 (100)	0/1 (0)	21/26 (81)

LDV, ledipasvir; RBV, ribavirin; SMV, simeprevir; SOF, sofosbuvir; OMB, ombitasvir; PTV/r, paritaprevir/ritonavir; LS, liver stiffness.

a One patient was treated with SOF+RBV and achieved SVR

b one patient (not included) had a diagnostic of cirrhosis by clinical criteria and achieved SVR.

# Sub article: P282 The role of two noninvasive tests to evaluate the liver fibrosis (FIB-4 and transient elastography) and its implications in the different HCV genotypes in a cohort of HIV/HCV co-infected patients

**Published:** 2016-10-23

**Authors:** Laura Perez Martinez, Carlos Lapresta, Maria Gracia, Javier Moreno, Alvaro Cecilio, Teresa Rubio, Carmen Irigoyen, Gloria Samperiz, Isabel Sanjoaquin, Jose Ramon Blanco

**Affiliations:** 1 Infectious Diseases Department, Hospital San Pedro-Center for Biomedical Research of La Rioja (CIBIR), Logroño (La Rioja), Spain; 2 Infectious Diseases, Hospital Universitario Miguel Servet, Zaragoza, Spain; 3 Infectious Diseases, Complejo Hospitalario de Navarra, Pamplona (Navarra), Spain; 4 Infectious Diseases, Hospital Clínico Universitario Lozano Blesa, Zaragoza, Spain; 5 Infectious Diseases, Hospital Reina Sofia de Tudela, Tudela (Navarra), Spain

## Abstract

**Introduction**: End-stage liver disease caused by chronic HCV infection is the leading cause of morbidity and mortality in HIV patients. Several factors such as duration of infection, age, male gender, consumption of alcohol and transmission path have been associated with a faster fibrosis progression rate. The aim of this study was to evaluate the FIB-4 score, a noninvasive test for the assessment of liver fibrosis, and to compare it with transient elastography (TE) in order to predict the fibrosis degree, and its implications in the different genotypes (GTs).

**Materials and methods**: Observational and multicentre study was conducted in five hospitals of the northern of Spain (2014–2015). HIV/HCV patients ≥18 years on stable cART (≥6 months) were selected to analyze their liver fibrosis using two noninvasive biomarkers: TE and FIB-4 index calculation.

**Results**: A total of 584 HIV/HCV patients were included (median age 49.5 years; male 71.2%; 86.9% people who inject drugs). Median CD4 was 620 cells/mL; 82% of them had a VL <50 copies/mL. HCV GT distribution was as follows: GT1 59.2% (72.3% of them GT1a), GT2 2.1%, GT3 22.1% and GT4 16.5%. Median liver fibrosis was 7.8 kPa; it was F0–F1 in 46.1%, F2 in 15.6%, F3 in 18.1% and F4 in 20.1%. Median FIB-4 was 1.7: <1.45, 1.45–3.25 and >3.25 in 36.5%, 43.4% and 20.1%, respectively. There was a significative correlation between fibrosis degree and FIB-4 score (p<0.0001). FIB-4 score was significantly lower in GT2 (median 1.3 vs. 1.8; p=0.04) but higher in GT3 (1.9 vs. 1.7; p=0.04). Comparing GT1a and GT1b, GT1a had a faster fibrosis degree (p=0.037).

**Conclusions**: The prevalence of severe fibrosis is high in these patients (20.1%). Compared with TE, FIB-4 has a good correlation. GT3 has a faster fibrosis grade, so an early therapeutic intervention in these patients is necessary. In the same way, GT1a has a higher fibrosis degree.

# Sub article: P283 Diversity of hepatitis C virus envelope associated with fibrosis in treatment-naïve patients

**Published:** 2016-10-23

**Authors:** Leontina Banica, Simona Paraschiv, Emil Neaga, Valeriu Gheorghita, Ionelia Batan, Ovidiu Vlaicu, Costin Ioan Popescu, Dan Otelea

**Affiliations:** 1 Molecular Diagnostic Laboratory, Prof. Dr. Matei Bals National Institute for Infectious Diseases, Bucharest, Romania; 2 Clinical Department, Prof. Dr. Matei Bals National Institute for Infectious Diseases, Bucharest, Romania; 3 Viral Glycoproteins, Institute of Biochemistry of the Romanian Academy, Bucharest, Romania

## Abstract

**Introduction**: Hepatitis C virus (HCV) continues to be a serious global health problem despite the introduction of direct acting antiretroviral drugs. The limitations in surveillance that contribute to high transmission rates as well as the emergence of drug resistance argue for vaccine development both for prophylactic and therapeutic purposes. High evolution rate of HCV due to high error rates of viral polymerase combined with constant immune pressure from the host generates major challenges for vaccine development. Herein, we evaluated inter-/intra-host diversity of HCV in the E1E2 region in relationship with fibrosis in treatment-naïve patients.

**Materials and methods**: Blood samples from nine treatment-naïve HCV genotype 1b infected patients, five with no/low fibrosis (F0, F1) and four with high fibrosis (F4) were collected. HCV full length E1E2 region was reverse transcribed and amplified in two rounds of PCR. The amplicon was cloned in the mammalian expression vector pcDNA™3.1/V5-His TOPO TA and 10 clones were sequenced using 3500 ABI instrument. BioEdit, Mega 7 and FastTree software tools were used to analyze the genetic evolution and the intra- and inter-host viral diversity.

**Results**: Intra-host variability was relatively low in patients with high fibrosis (F4), while for those with no/low fibrosis (F0, F1) the viral diversity varied, being high in those with older infection and low in acute infection. The impact of positive (immune-mediated) or negative (virus adaptation) selection on viral diversity was evaluated based on non-synonymous to synonymous substitution rates per site (dN/dS ratio). dN/dS ratio showed a reduced immune pressure on E1E2 glycoprotein of HCV-infected patients with high fibrosis, while those with chronic infection and low fibrosis showed a strong positive selection pressure in hypervariable region 1 of E2 glycoprotein.

**Conclusion**: Advanced fibrosis was associated with low intra-host viral diversity and reduced selection pressure; apparently viral populations that are structurally conserved and tolerated by the immune system are being stably selected during late stages of disease.

**Acknowledgments**: This work was supported by ANCSI; grant EEA-JRP-RO-NO-2013-1-0022, GreenVac Project.

# Sub article: P284 Ledipasvir/sofosbuvir for 8 or 12 weeks in GT1 HCV-infected, treatment-naïve and non-cirrhotic patients with HIV infection: real-world experience from the MADRID-CoRe study

**Published:** 2016-10-23

**Authors:** Juan Berenguer, Ángela Gil-Martin, Ana Moreno, Francisco Moreno, Lourdes Dominguez, Teresa Aldámiz-Echevarría, Encarnación Cruz-Martos, Vicente Estrada, Ignacio Santos, Laura Benítez, José Sanz, Pablo Ryan, Gabriel Gaspar, Beatriz Álvarez-Álvarez, Juan Emilio Losa, Juan José Jusdado, Carlos Barros, Eduardo Malmierca, María José Calvo, Marta Alcaraz, Inmaculada Jarrin, Juan González-García

**Affiliations:** 1 Infectious Diseases, Hospital General Universitario Gregorio Marañón, Madrid, Spain; 2 Pharmacy, Servicio Madrileño de Salud, Madrid, Spain; 3 Infectious Diseases, Hospital Ramón y Cajal, Madrid, Spain; 4 Internal Medicine, Hospital Universitario La Paz, Madrid, Spain; 5 Internal Medicine, Hospital Doce de Octubre, Madrid, Spain; 6 Infectious Diseases, Hospital Clínico de San Carlos, Madrid, Spain; 7 Infectious Diseases, Hospital Universitario de La Princesa, Madrid, Spain; 8 Internal Medicine, Hospital Universitario Puerta de Hierro, Madrid, Spain; 9 Internal Medicine, Hospital Principe de Asturias, Alcalá de Henares, Spain; 10 Internal Medicine, Hospital Infanta Leonor, Madrid, Spain; 11 Internal Medicine, Hospital Universitario de Getafe, Madrid, Spain; 12 Infectious Diseases, Fundación Jiménez Días, Madrid, Spain; 13 Infectious Diseases, Fundación Hospital de Alcorcón, Madrid, Spain; 14 Internal Medicine, Hospital Severo Ochoa, Madrid, Spain; 15 Infectious Diseases, Hospital de Móstoles, Madrid, Spain; 16 Internal Medicine, Hospital Infanta Sofía, Madrid, Spain; 17 Public Health, Instituto de Salud Carlos III, Madrid, Spain

## Abstract

**Introduction**: We evaluated therapeutic outcomes of LDV/SOF for 8 or 12 weeks in HCV genotype 1 (GT1) HCV-infected, treatment-naïve (TN) and non-cirrhotic patients with HIV infection.

**Materials and methods**: The Madrid co-infection registry (MADRID-CoRe) is a prospective registry of all co-infected adults (≥18 years) undergoing DAA therapy (Rx) for HCV in hospitals from the Madrid Regional Health Service (SERMAS). We assessed SVR12, viral relapse and failure due to Rx discontinuation. Between November 2014 and May 2016, 2402 co-infected individuals have initiated DAA Rx within MADRID-CoRe. Herein, we present data of co-infected patients with GT1, TN and non-cirrhotic that were treated with LDV/SOF for 8 or 12 weeks without ribavirin, and with programmed Rx finalization censored to 31 December 2015.

**Results**: We evaluated 192 co-infected individuals who met the inclusion criteria: 134 treated with LDV/SOF for 12 weeks and 58 treated for LDV/SOF for 8 weeks. Patients’ characteristics and treatment outcomes are shown in Table 1.

**Conclusions**: In real-life clinical practice, no significant differences were found in effectiveness and safety with LDV/SOF for 8 or 12 weeks for GT1 in TN, non-cirrhotic co-infected patients.

**Abstract P284–Table 1:** Patients’ characteristics and treatment outcomes

	LDV/SOF 8 weeks (N=58)	LDV/SOF 12 weeks (N=134)	P
Baseline variables	G1a=40, G1b=12, non-subtyped=6	G1a=95, G1b=36, non-subtyped=3	
Age, years, median	49 (45–53)	51 (47–54)	0.030
Male, %	43 (74.1)	105 (78.4)	0.520
cART, %	55 (94.8)	132 (98.5)	0.140
Log HCV RNA, median	6.1 (5.7–6.6)	6.4 (6.0–6.8)	0.004
Liver stiffness kPa, median	7.7 (7.2–9.0)	8.8 (7.7–10.3)	0.002
Outcomes			
SVR12, n (%)	52 (89.7)	122 (91.0)	0.760
% SVR12, 95% CI	78.8–96.1	84.9–95.3	
Relapse, n (%)	4 (6.9)	7 (5.2)	0.650
Breakthrough, n (%)	0	0	–
D/C due to AEs, n (%)	0	1 (0.7)	0.510
D/C other reasons, n (%)	2 (3.4)	4 (3.0)	0.870

SOF, sofosbuvir; LDV, ledipasvir; SVR, sustained virologic response; D/C, discontinuation; AEs, adverse events.

# Sub article: P285 Effect of mono/dual antiretroviral therapy on HCV and HIV suppression during HCV treatment in HIV/HCV co-infected patients

**Published:** 2016-10-23

**Authors:** Luz Martín-Carbonero, Lourdes Dominguez, Lucía Bailón, Rafael Torres, Rafael Rubio, Raquel Ron, Mikel Rico, Inmaculada Jiménez-Nacher, Francisco Moreno, Juan González, Federico Pulido, Marisa Montes

**Affiliations:** 1 HIV Unit, Internal Medicine, Hospital Universitario La Paz, IdiPAZ, Madrid, Spain; 2 HIV Unit, Internal Medicine, Hospital Universitario Doce de Octubre, IMAS12, Madrid, Spain; 3 HIV Unit, Internal Medicine, Hospital Universitario Severo Ochoa, Leganés, Spain; 4 Pharmacy, Hospital Universitario La Paz, IdiPAZ, Madrid, Spain

## Abstract

**Introduction**: New HCV treatment combinations have been studied only with few HIV drugs and always in triple therapy. In the clinical setting, many times we have to use non-conventional combinations, such as mono- or dual therapy, due to resistance or toxicity. The effect of non-conventional ART combinations on both HCV and HIV suppression needs to be assessed.

**Material and methods**: Retrospective review of HIV/HCV co-infected patients has initiated DAA-based HCV treatment from November 2014 to 2015 in three different hospitals in Madrid. HIV viral suppression was assessed at the beginning and at the end of HCV treatment and compared between groups that have received triple therapy versus mono- or dual antiretroviral treatment. Values are given as percentage and median (interquartile range) for qualitative and quantitative variables, respectively. Chi-square and non-parametric (U de Mann-Whitney) test were used for comparisons.

**Results**: Overall, 596 patients initiated HCV treatment. Of them, 393 were receiving a triple antiretroviral combination, 66 PI/r monotherapy, 51 PI/r+3TC, 32 other dual therapies and 40 other combinations. Mono-/dual therapy groups were older than patients on triple therapy. However, the rest of baseline characteristics were similar between groups (Table 1).

HCV sustained virologic response (SVR) 12 weeks after the end of therapy were 93.2% (520/560) and 94.6% (522/552) in intention-to-treat (ITT) and on-treatment analysis, respectively. No differences in SVR were seen in patients on triple therapy or mono-/dual therapy: ITT (92.9% vs. 95.3%; Δ=−2.4; 95% CI −6.3 to 1.5; p=0.3), OTT (93.6% vs. 96.1%; Δ=−2.5; 95% CI −6.6 to 1.66; p=0.2). HIV viral load <50 copies/mL rate at the end of HCV treatment was 96% and 97.6% with conventional and non-conventional HAART (Δ=1.6; 95% CI −1.4 to 5.36; p=0.4). Neither were differences seen 12 weeks after (94.5% vs. 95%; Δ=−0.1; 95% CI −4.49 to 5.51; p=0.9). CD4 cell count did not significantly change during HCV treatment: 16 (95% CI −68 to 104) versus −18 (95% CI −138 to 100) in triple versus mono-/dual groups, respectively; p=0.1.

**Conclusion**: Mono- and dual ART maintain HIV suppression during DAA-based HCV treatment. HCV SVR at week 12 is very high in HIV/HCV co-infected patients independently of using triple ART or non-conventional combinations.

**Abstract P285–Table 1:** Baseline characteristics of patients with triple versus mono/dual ATR

	All	Triple Therapy	Mono-Dual therapy	
		
	N=596	N=393	N=149	p
Male gender	450 (75.4)	300 (76.3)	109 (73.2)	0.2
Age	51 (48–54.2)	50.5 (47.4–54.3)	52 (49.3–54.3)	0.03
Genotype				0.1
1a	240 (44.1)	167 (46.9)	47 (35.1)	
1b	90 (16.5)	56 (15.7)	30 (22.4)	
3	78 (14.3)	48 (13.4)	23 (17.2)	
4	133 (24.4)	84 (23.5)	33 (24.6)	
RNA-HCV	6.2 (5.8–6.6)	6.2 (5.8–6.6)	6.3 (5.9–6.7)	0.1
Liver Stiffness (kPa)				0.6
<7	62 (10.4)	43 (11)	12 (8.1)	
7.1–9.5	181 (30.4)	118 (30.2)	42 (28.2)	
9.6–14	124 (20.8)	77 (19.7)	35 (23.5)	
>14	228 (38.3)	153 (39.1)	60 (40.3)	
Previous HCV treatment	248 (41.5)	174 (44.3)	54 (36.2)	0.09
HCV treatment				0.2
Sofosbuvir/Ledipasvir	410 (68.8)	261 (66.4)	105 (70.5)	
Ombitasvir/Paritaprevir/r +/- DasabuvirAbbvie	87 (14.6)	64 (16.3)	21 (14.1)	
Sofosbuvir/Daclatasvir	76 (12.7)	48 (12.3)	21 (14.1)	
Ribavirin	168 (28.2)	38 (25.5)	121 (30.8)	0.2
Treatment duration				0.1
8 weeks	22 (3.7)	10 (2.5)	9 (6)	
12 weeks	367 (61.6)	240 (61.1)	92 (61.7)	
24 weeks	207 (34.7)	143 (36.4)	48 (32.2)	
Baseline HIV-RNA <50 copies/mL	554 (94.5)	367 (94.6)	146 (98)	0.08
Baseline CD4 cell count	593 (399–832)	582 (379–829)	613 (433–895)	0.3

# Sub article: P286 Health resources use in patients with HIV: evidence from Italian administrative databases

**Published:** 2016-10-23

**Authors:** Valentina Perrone, Diego Sangiorgi, Elisa Crovato, Stefano Buda, Luca Degli Esposti

**Affiliations:** 1 CliCon S.r.l. Health, Economics and Outcomes Research, Ravenna, Italy

## Abstract

**Introduction**: The success of ART has changed HIV from a life-threatening disease to a manageable lifelong disease. Today HIV+ patients have a life expectancy approaching that of the general population, but are exposed to a higher risk of developing comorbidities as a consequence of ageing and exposure to highly active ART. Currently, scarce evidence is available on comorbidities in HIV+ patients and the related burden on the healthcare systems. This study aimed to evaluate the prevalence of comorbidities among HIV patients and estimate the associated healthcare costs.

**Materials and methods**: An observational retrospective cohort analysis, using administrative and laboratory test outcomes databases from seven local health units (LHUs) in Italy, was designed. Currently, data from one LHU are presented in this analysis. Records of patients diagnosed with HIV (identified through hospitalizations, specific treatments or blood test results) between 1 January 2013 and 31 December 2015 were extracted. The date of the first HIV-related healthcare consumption was used as the index date. Clinical characteristics of patients were investigated in the year before the index date. All patients were followed up for one year after the index date (only patients with one year follow-up were included).

**Results**: The preliminary analyses from one LHU included 366 HIV+ patients. Mean age was 53.6 years, 66% were male and 5% had AIDS. Twenty-five percent of patients had one comorbidity, 11% had two and 6% had three or more. Thirty-two percent of patients had rheumatologic diseases (anti-inflammatory/anti-rheumatic drugs prescribed), 7% had chronic kidney disease (CKD; defined as GFR <60 mL/min, from laboratory outcomes database). Seventeen percent of patients were prescribed statins and 2% of patients were prescribed osteoporosis drugs. Three percent were hospitalized for cardiovascular disease (heart failure, myocardial infarction, cerebrovascular event) and 1% experienced a hospitalization for fracture. On average, the annual healthcare cost of a patient without comorbidities was 8400€, the cost of a patient with one comorbidity 9400€, and the cost of a patient with two comorbidities or three or more were 9700€ and 10,600€, respectively.

**Conclusions**: In this cohort, 42% of HIV+ patients were shown to have at least one comorbidity. In addition, healthcare costs were also shown to increase with the number of comorbidities. Evidence from this study suggests that a multidisciplinary approach to HIV+ patients is required to optimize care and healthcare consumption.

# Sub article: P287 HCV+ and HIV/HCV+ patients treated with direct antiviral agents (DAAs): to what extent do they differ?

**Published:** 2016-10-23

**Authors:** Giuseppe Bruno, Claudia Fabrizio, Raffaele Dell'Acqua, Eugenio Milano, Michele Milella, Annalisa Saracino, Laura Monno, Gioacchino Angarano

**Affiliations:** 1 Clinic of Infectious Diseases, University of Bari, Bari, Italy

## Abstract

**Introduction**: DAA treatment has been associated with high rates of sustained virological response (SVR) and a good safety profile both in HCV+ and HIV/HCV+ individuals. We aimed to assess the efficacy and safety of DAAs in HIV/HCV+ compared with HCV+ patients in a large single-centre.

**Methods**: All HCV-infected patients, with or without HIV infection, who received an IFN-free regimen with DAAs from February 2015 throughout June 2016, were enrolled. Clinical, virological and laboratory data were collected.

**Results**: A total of 449 patients received DAAs, and 339 (54 HIV/HCV+ and 285 HCV+) completed their treatment. A follow-up ≥3 months after the end of treatment (EOT) was available for 184 HCV+ and 40 HIV/HCV+ subjects. HIV/HCV+ individuals were younger (median age 52 vs. 68 years), mostly male (90.9% vs. 57.2%) and more commonly infected with genotypes 1a (52.7% vs. 6.4%), 3 (25.5% vs. 6.8%) and 4 (14.5% vs. 3.1%). Two hundred and twenty-four (63.4%) patients were cirrhotic and 188 (53.7%) had a previous therapy failure. HCV+ patients were more likely to have ≥2 comorbidities (27.8% vs. 9%; p=0.003). Type of therapy, effectiveness and safety were analyzed for 350 patients, including 11 subjects who discontinued treatment (Table 1).

Most common adverse events were rash (16.9%), fatigue (12.6%), anaemia (13.1%) and headache (8.6%). Severe adverse events (SAE) occurred in eight HCV+ (2.3%) and in one HIV/HCV+ patient (1.8%). HCV+ patients experienced more frequently ≥2 adverse events (23.1% vs. 9.1%, p=0.02) and underwent a larger ribavirin use (69.2% vs. 54.5%, p=0.03). Therapy was discontinued due to liver transplantation (one patient), SAE (nine patients, including one death because of oesophageal varices bleeding), breakthrough (one HIV/HCV+ genotype 4 patient). SVR12 was achieved in 92.4% patients overall, 92.9% HCV+ versus 90% HIV/HCV+ (p=0.52). Relapse was observed in five genotype 3 patients (three HCV+ and two HIV/HCV+) treated with sofosbuvir and ribavirin and one genotype 4 HIV/HCV+ patient treated with sofosbuvir and simeprevir.

**Conclusion**: A worse safety profile pertained HCV+ patients with a higher burden of comorbidities. However, an overall high rate of SVR12 was obtained independent of HIV infection.

**Table 1:** Type of DAAs therapy, effectiveness data and safety profile of the 350 patients

	Total		HCV		HIV-HCV		
				
Total number of patients evaluated	350	%	295	%	55	%	p*
Type of therapy							
SOF+RBV	78	22.3	66	22.4	12	21.8	0.93
SOF+SIM±RBV	56	16.0	39	13.2	17	30.9	0.001
SOF+LDV±RBV	56	16.0	46	15.6	10	18.2	0.63
SOF+DCV±RBV	25	7.1	21	7.1	4	7.3	0.96
OMB+PTV/R+DAS±RBV	129	36.9	118	40.0	11	20.0	0.005
OMB+PTV/R +RBV	6	1.7	5	1.7	1	1.8	0.94
Use of ribavirin	234	66.8	204	69.2	30	54.5	0.03
Effectiveness							
Patients with EOT	339	96.8	285	96.6	54	98.1	0.54
Drop-out	11	3.1	10	3.3	1	1.8	0.54
Patients with at least 3 m of follow-up post EOT	224	49.8	184	62.3	40	72.7	0.14
SVR12	207	92.4	171	92.9	36	90	0.52
Most common side effects							
Headache	30	8.6	26	8.8	4	7.3	0.71
Insomnia	13	3.7	11	3.7	2	3.6	0.97
Fatigue	44	12.6	34	11.5	10	18.2	0.17
Dizziness	14	4.0	12	4.1	2	3.6	0.88
Mood disorders	13	3.7	11	3.7	2	3.6	0.97
Gastrointestinal disorders	22	6.3	20	6.8	2	3.6	0.38
Jaundice	8	2.3	4	1.4	4	7.3	0.007
Rash, pruritus or photosensitivity	59	16.9	53	18.0	6	10.9	0.19
Haematological abnormalities							
Anaemia	46	13.1	40	13.6	6	10.9	0.59
Haemoglobin <10 g/dl	29	8.3	23	7.8	6	10.9	0.44
Haemoglobin <8 g/dl	5	1.4	5	1.7	0	0.0	0.33
Blood transfusion	5	1.4	5	1.7	0	0.0	0.33
Reduction RBV	27	7.7	26	8.8	1	1.8	0.07
EPO use	17	4.9	14	4.7	3	5.5	0.82
At least two adverse events	73	20.9	68	23.1	5	9.1	0.02
Severe adverse events	9	2.6	8	2.7	1	1.8	0.7
Gastrointestinal haemorrhage	5	1.4	4	1.4	1	1.8	0.79
Severe anaemia	1	0.3	1	0.3	0	0.0	0.66
Encephalopathy	2	0.6	2	0.7	0	0.0	0.54
Ascites	1	0.3	1	0.3	0	0.0	0.66

Results are presented as frequencies (%) for qualitative variables. DAS, dasabuvir; DCV, daclatasvir; EOT, end of treatment; LDV, ledipasvir; OMB, ombitasvir; PTV/r, paritaprevir/r; RBV, ribavirin; SIM, simeprevir; SOF, sofosbuvir; SVR, sustained virological response.

* p<0.05.

# Sub article: P288 Renal safety of boosted PI in HIV/HCV patients on SOF/LDV

**Published:** 2016-10-23

**Authors:** Maria J Vivancos-Gallego, Ana Moreno, Maria Perez Elias, Carmen Quereda, Jose Luis Casado, Matilde Sanchez-Conde, Enrique Navas, Santos Del Campo, Santiago Moreno

**Affiliations:** 1 Infectious Diseases, Hospital Ramon y Cajal, Instituto Ramon y Cajal de Investigación Sanitaria, Madrid, Spain

## Abstract

**Objectives**: To better describe safety of PI-boosted (ritonavir/cobicistat) plus ledipasvir/sofosbuvir (LDV/SOF) in routine clinical care.

**Methods**: A retrospective HIV/HCV cohort study of patients from a tertiary centre in Madrid, Spain, starting LDV/SOF with PI-boosted with complete renal function and overall safety follow-up data during therapy. Paired baseline and at the end of treatment eGFR (CKD-EPI) comparisons were made regarding cobicistat or ritonavir boosted PI use (plus TDF or not).

**Results**: From a DAA cohort of 424 co-infected patients, 83 were on protease inhibitor-based regimen for HIV. Of these, 47 patients on LDV/SOF (57%) were included (83% men, age 51 years (47–51)). HCV genotypes: 1 (68.1%); 3 (10.6%); 4 (21.3%). DAA duration was: 8 weeks (five patients); 12 weeks (33 patients); 24 weeks (nine patients). Twenty-eight patients without cirrhosis (59.6%), 19 with cirrhosis (40.4%). PI distribution was: darunavir/ritonavir (25 patients); darunavir/cobicistat (11 patients); lopinavir/ritonavir (six patients); and atazanavir/ritonavir (five patients). Antiretrovirals associated with PI were: Kivexa (six patients) and Truvada (nine patients); lamivudine (16 patients) and PI monotherapy (16 patients). Median time between the baseline and the last eGFR was 24 weeks (22–26). Mean baseline eGFR (CKD-EPI) in darunavir/cobicistat group was 94.4 mL/min meanwhile in the others boosted PI was 91.2 mL/min (p=0.6). After the end of LDV/SOF mean eGFR was 94.2 mL/min versus 83.4 mL/min, respectively (p=0.2). In the boosted TDF group mean baseline eGFR was 95.4±11.5 mL/min versus 74.5±32.2 mL/min in the last observation (p=0.15). The observed changes in eGFR were not statistically significant, of small magnitude and non-clinically relevant. No adverse events were reported during treatment.

**Conclusion**: In a population of HCV/HIV co-infected patients no impact on kidney function or safety considerations was observed during the short 8 to 24 weeks DAA treatment duration with cobicistat/ritonavir boosted PI and LDV/SOF therapy.

# Sub article: P289 Entecavir plus lamivudine as an alternative treatment for co-infected HBV/HIV patients with toxicity on tenofovir therapy

**Published:** 2016-10-23

**Authors:** María Fontecha-Ortega, Vanesa Muñoz-Mendoza, Marta Monsalvo, Marisa Mateos, Miguel Angel Rodríguez-Sagrado, Cristina Gómez, Jose Luis Casado

**Affiliations:** 1 Infectious Diseases, Hospital Ramón y Cajal, Madrid, Spain; 2 Microbiology, Hospital Ramón y Cajal, Madrid, Spain; 3 Pharmacology, Hospital Ramón y Cajal, Madrid, Spain

## Abstract

**Introduction**: Combination of lamivudine (LAM) or emtricitabine with tenofovir disoproxil fumarate (TDF) is the recommended first-line regime for treatment in chronic hepatitis B virus (HBV)/HIV co-infection. However, little is known about the best strategy in patients who developed tenofovir toxicity. We report the outcome of HBV co-infected patients who switched from TDF to entecavir (ETV) due to renal or bone toxicity with maintenance of LAM.

**Materials and methods**: Retrospective case series (2009–2015) of HBV/HIV co-infected patients who developed TDF toxicity and switched to ETV together with LAM. HBV suppression (HBV DNA) and renal and bone toxicity were evaluated during follow-up.

**Results**: Overall, 12 patients switched to ETV+LAM because TDF toxicity. Mean age was 54.9 years, and 83% male. Patients showed chronic replicative HBV co-infection (Ag-HBe positive in four cases, Ac-HBe in six cases; median HBV DNA level at baseline was 104.146 UI/mL) suppressed while receiving HBV therapy with LAM for a median of 122 months (49.6–170.3), along with TDF for 67 months (34–136.2). Thus, at the time of change, HBV DNA was undetectable in all the cases except one patient with lack of adherence (1,700,000 UI/L). Patients showed additional cases of renal or bone toxicity (two HTA, two DM, two liver transplantation, one renal transplant, one patient on dialysis, four patients receiving antineoplastic chemotherapy and one patient with Fanconi syndrome suspicion). At the time of switch, mean estimated glomerular filtration rate (eGFR) was 87.3 mL/min (49–103.3) and six patients were below 60 mL/min. Urine analysis showed a protein-creatinine ratio (PCR) of 112 mg/gr (40–189) and fractional excretion of phosphate was 35.4% (13.5–79.9%). Also, two patients showed severe osteoporosis and three patients had vertebral fractures. During a median follow-up of 29.4 months on ETV (3.9–81; 7.3 patient-years), HBV remained suppressed in all the patients, with normalization of transaminases. In addition, there was improvement in eGFR (from 75.6 to 90.6 mL/min; 0.16), serum phosphate (from 2.7 to 3.11; p=0.06) and FE of phosphate in urine (from 59.2 to 74.3; p=0.21).

**Conclusions**: The switch to entecavir together with lamivudine could be an alternative to TDF in HBV/HIV co-infected patients in case of toxicity or intolerance.

# Sub article: P290 Direct-acting antiviral drugs (DAAs) for the treatment of chronic HCV infection in HIV co-infected patients: a monocentric experience in real life

**Published:** 2016-10-23

**Authors:** Ilaria Izzo, Salvatore Casari, Andrea Vavassori, Paola Nasta, Silvia Odolini, Filippo Castelnuovo, Alberto Bergamasco, Issa El Hamad, Giuseppe Paraninfo, Angiola Spinetti, Serena Zaltron, Lucia Urbinati, Luciano Biasi, Emanuele Focà, Elena Festa, Graziella Cristini, Francesco Castelli

**Affiliations:** 1 Department of Infectious and Tropical Diseases, University of Brescia and Spedali Civili General Hospital, Brescia, Italy; 2 Department of Infectious Diseases, Spedali Civili General Hospital, Brescia, Italy

## Abstract

**Introduction**: There are few data on the real-world experience of hepatitis C virus (HCV) direct-acting antivirals (DAAs) in HIV/HCV co-infected patients. The aim of the study was to evaluate the efficacy of DAA therapies in a cohort of HIV/HCV patients in the Infectious Diseases Department in Brescia, Northern Italy.

**Materials and methods**: We retrospectively analyzed data of all HIV/HCV patients who started treatment with DAAs from February 2015 to March 2016. The primary outcome was to evaluate sustained virologic response at 12 weeks after DAAs completion (SVR12).

**Results**: Hundred and fifty-three HCV/HIV co-infected patients started treatment in the study period. Most patients were male (121, 79.1%) and HCV genotypes 1a, 3 and 4 were the most represented (55, 36.0%; 37, 24.2% and 33, 21.6%, respectively). cART was modified before starting DAA in 35/153 patients (22.9%), to avoid PK interactions. Sixty-four (41.8%) patients had received prior treatment with an IFN-containing regimen; 126 (82.4%) of patients presented with cirrhosis, 25 (16.3%) presented with moderate fibrosis at transient elastography (F3) and one (0.65%) was treated according to Agenzia Italiana del Farmaco (AIFA) inclusion criteria no. 3 (extra-hepatic HCV manifestations). Ribavirin was included in the vast majority of regimens (109 patients, 71.2%). The following regimens were prescribed: sofosbuvir/ledipasvir (±ribavirin) 68 (44.5%), sofosbuvir/daclatasvir (±ribavirin) 52 (34%), sofosbuvir/ribavirin nine (5.9%), sofosbuvir/simeprevir (±ribavirin) eight (5.2%), ombitasvir/paritepravir/ritonavir/dasabuvir (±ribavirin) eight (5.2%) and ombitasvir/paritepravir/ritonavir (±ribavirin) eight (5.2%). Among the 72 patients who completed therapy and had a 12-week follow-up by the end of the study, the overall SVR12 rate was 95.8% and 3/72 (4.2%) patients faced virologic failure. No significant differences in SVR12 rate were observed according to gender, age, fibrosis grade and baseline HCV viral load. Anyway the three failed patients were males with cirrhosis.

**Conclusions**: Treatment with DAAs was highly effective (cure rate of 95.8%) in our cohort of co-infected patients, the majority of whom (82.4%) presented with cirrhosis. Neither HIV co-infection nor advanced liver disease should be considered as a barrier to HCV treatment. However, DAAs-cART interactions are a real challenge during therapy and in some cases (22.9% in our experience) cART needs to be modified before DAA treatment.

# Sub article: P291 Daclatasvir (DCV) pharmacokinetics in HCV/HIV co-infected patients co-administered with rilpivirine and other antiretroviral drugs

**Published:** 2016-10-23

**Authors:** Ambra Barco, Letizia Marinaro, Marco Merli, Chiara Alcantarini, Chiara Montrucchio, Giulia Vendemiati, Maurizio Milesi, Francesca Patti, Fabio Favata, Alessandra Ariaudo, Hamid Hasson, Antonio D'Avolio, Caterina Uberti-Foppa, Giovanni Di Perri, Stefano Bonora

**Affiliations:** 1 Unit of Infectious Diseases, Ospedale Amedeo di Savoia, Torino, Italy; 2 Infectious Diseases Clinic, San Raffaele Hospital, Milano, Italy

## Abstract

**Introduction**: Due to potential drug-drug interaction, DCV dose reduction to 30 mg is required when co-administered with atazanavir/ritonavir (ATV/r), while no data are available for unboosted atazanavir (ATV). Moreover, no data on rilpivirine (RPV) and DCV interaction are currently available. The aim of our study was to describe DCV pharmacokinetics when co-administered with different protease inhibitors (PIs) and RPV in a real-life cohort of HIV/HCV-positive patients.

**Materials and methods**: HIV/HCV co-infected patients treated with DCV plus sofosbuvir (SOF) for at least 4 weeks and receiving ART were enrolled. Assuming a comparable effect of ATV and ATV/r, patients treated with ATV received a reduced DCV dose of 30 mg. DCV plasmatic levels (DCVpl) (22±2 hours after last intake) were evaluated using UPLC-MS/MS validated method and reported as ng/mL. Data are expressed as numbers (percentage) and median (IQR).

**Results**: Twenty-nine patients were enrolled: 86.2% males, age 52 (IQR 49–54), BMI 25 kg/m^2^ (22.5–27.7). Metavir score was 4, 3 and 1 in 22 (76%), 6 (21%) and 1 (3%), respectively. Child-Pugh score was A and B in 93% and 7%, respectively. Twenty-four patients had HCV genotype 3 and five had genotype 1. ART was PI-based in 13 (48%) patients (six ATV/r, two ATV, five DRV/r), RPV-containing in eight (26%) and INI-containing in eight (26%). Twenty-seven DCV determinations were obtained at week 4. Median DCVpl in study population was 212 ng/mL (103–299). In patients co-administered with RPV, DCVpl was 216 ng/mL (61–383) and no significant statistical difference was found in DCVpl of patients receiving other ARVs (p=1). Patients receiving PI showed a DCVpl of 280 ng/mL (224.5–369.75), with no statistical difference compared with DCVpl of RPV group (p=0.36). DCVpl of subjects in ATV/r (280 ng/mL, 212–283) and DRV/r (315 ng/mL, 277–315) showed no significant difference (p=0.29), as well as DCVpl of those in ATV/r or ATV (237 ng/mL, 212–237) (p=0.37).

**Conclusions**: DCV plasmatic concentrations in our cohort of patients administered with different ARV regimens resulted comparable to values reported from literature. This is the first report on DCV exposures in HIV/HCV patients co-administered with RPV. Results show that standard DCV dose of 60 mg provides adequate DCV levels, comparable to those reported with other regimens. Moreover our findings confirm the appropriateness of reduced DCV dose of 30 mg both in individuals treated with ATV/r and in patients receiving unboosted ATV.

# Sub article: P292 Chronic HCV genotype 4 infection in a Portuguese cohort of co-infected HIV patients: treatment with sofosbuvir-based regimens

**Published:** 2016-10-23

**Authors:** Pedro Brogueira, Virginia Moneti, Ana Miranda, Fernando Ventura, Teresa Baptista, Fernando Borges, Jaime Nina, Susana Peres, Isabel Aldir, Maria Campos, Isabel Antunes, João Pereira, Kamal Mansinho

**Affiliations:** 1 Infectious Diseases, Hospital Egas Moniz, Lisbon, Portugal

## Abstract

**Introduction**: HCV genotype 4 infection has an estimated prevalence of 10 to 20% worldwide. In Europe the number of infections has been rising due to several epidemiologic variations. In Portugal its incidence reaches 7% [1]. The high rates of cure seen with oral-based regimens brought the need to well characterize genotype 4 infection in terms of its epidemiology, natural history and response to treatment in a real-life experience basis.

**Materials and methods**: At our Infectious Disease Center, since 1 January 2015 until 31 March 2016, 416 patients were eligible to engage HCV treatment with DAA regimens.

**Inclusion criteria**: Included all chronically HCV-infected patients, with or without HIV infection, followed at our service, and proposed to DAA treatment. Epidemiologic, demographic, clinical, laboratory and therapeutic data were collected.

**Data analysis**: This was performed by using Microsoft Excel and SPSS version 15.0.

**Results**: Of the 416 patients proposed for DAA treatment, 281 patients were HIV co-infected (68%) and 135 were HCV mono-infected (32%). The global prevalence of HCV genotype 4 was 17% (n=72), 20% in co-infected (HIV/HCV) versus 11% in HCV mono-infected patients. The 72 patients with genotype 4 infection included 57 (79%) patients co-infected with HIV, 77% with TCD4 cell count >500 cells/µL, all of them under cART with viral suppression. The demographic analysis of co-infected versus HCV mono-infected patients revealed: male gender 80% versus 82%, mean age 48 years versus 56 years, injectable drug use being the more frequent route of transmission 78% versus 45%, mean time since diagnosis 15 years versus 11 years. Chronic liver disease stage was Child-Pugh A in 91% versus 100%; MELD <9 in 87% versus 100%. Serum markers of fibrosis: FIB-4 >3.25 in 10% versus 36% and APRI >0.7 in 20% versus 36%. Real-time elastography was performed and revealed fibrosis stage ≤F2 in 39% versus 20%. Stage F4 was detected in four patients (7%) versus one (7%). The proposed treatment was SOF/LED in 97% of patients (n=70); SOF/LED+RBV (n=1); SOF+PEG+RBV (n=1). At the present date, 64 patients started treatment, 80% (n=51) HIV/HCV versus 20% (n=13) HCV mono-infected. The preliminary sustained virologic response rate was 95%. There was one death due to hepatocellular carcinoma.

**Conclusions**: Our cohort reveals a genotype 4 prevalence above that estimated for general population, especially in the HIV co-infected patients reaching 20%. This analysis suggests that co-infected patients have an earlier diagnosis of HCV infection, as they were younger and presented earlier in time.

## References

[1] Anjo J, Café A, Carvalho A, Doroana M, Fraga J, Gíria J (2014). The burden of hepatitis C in Portugal. GE Jornal Português de Gastrenterologia.

# Sub article: P293 Mortality during direct-acting antiviral therapy in HCV/HIV patients with cirrhosis

**Published:** 2016-10-23

**Authors:** Maria J Vivancos-Gallego, Ana Moreno, Maria J Perez Elias, Carmen Quereda, Jose Luis Casado, Cristina Gomez Ayerbe, Matilde Sanchez-Conde, Santos Del Campo, Santiago Moreno

**Affiliations:** 1 Hospital Ramon y Cajal, Instituto Ramon y Cajal de Investigación Sanitaria, Infectious Diseases, Madrid, Spain

## Abstract

**Introduction**: Direct antiviral agents (DAAs)-based therapy has dramatically changed outcomes among patients with cirrhosis, but the benefits in advanced liver disease are unclear.

**Materials and methods**: From April 2013, we consecutively included 181 HIV/HCV cirrhotics patients treated with DAAs. Baseline characteristics, sustained virologic response (SVR12) and discontinuations for any reason were recorded.

**Results**: The baseline characteristics are in Table 1. Most were GT1 (61%) and treatment experienced (58%). The rate of SVR12 obtained for our cohort was 85% (149/175). There was a statistically significant difference in the SVR12 rate in non-GT4-infected patients compared with GT4-infected patients (88.9% vs. 67.7%, p=0.009). Simeprevir (SMV)-including DAA therapy was associated with treatment failure p=0.009. The premature discontinuation rate was 4.4% (8/181) in our study, of whom five patients (62.5%) died. Reasons for discontinuation in the remaining three patients included intracranial haemorrhage (n=1), upper gastrointestinal bleeding (n=1) and liver transplantation (n=1). On-treatment mortality rate was 2.76% and the baseline demographics and HCV characteristics of these five patients are described. The median age was 52 years and received treatment with DCV/SOF in three patients and LDV/SOF in two patients. Genotypes distribution was 1a (two patients), 1b (one patient) and 3 (two patients). The mean model for end-stage liver disease (MELD) score, platelet counts, bilirubin and albumin levels were not statistically different from those patients who survived treatment. The causes of death for the five patients were hepatocarcinoma (n=3), upper gastrointestinal bleeding (n=1) and sepsis (n=1). Two of three patients with hepatocarcinoma developed a multicentric HCC “de novo” with rapid progression (Table 2). Of note, while there were five on-treatment deaths there was no mortality in the 12 weeks post-treatment. Three patients with premature discontinuation reached SVR12.

**Conclusions**: We did not find a high mortality rate although close monitoring on direct-acting antiviral therapy is essential. DAAs effectively cured HCV in patients with advanced liver disease. The longer-term impact of HCV treatment in patients with cirrhosis remains to be determined.

**Table 1:** Baseline characteristics

Baseline demographics	Total n=181	Died on treatment n=5
Median age (range), years	52 (36–73)	52.6 (44–57)
Male, n (%)	149 (82%)	5 (100%)
History of decompensation, n (%)	14 (8%)	3 (60%)
MELD score (range)		
0–9, n (%)	94 (57%)	2 (40%)
10–15, n (%)	60 (37%)	2 (40%)
16–21, n (%)	9 (5%)	0 (0%)
21+, n (%)	1 (1%)	1 (20%)
HCV genotype		
1a, n (%)	73 (41%)	2 (40%)
1b, n (%)	37 (20%)	1 (20%)
1 (nosubtype), n (%)	51 (39%)	2 (40%)
2, n (%)	1 (0.6%)	0 (0%)
3, n (%)	37 (20.4%)	2 (40%)
4, n (%)	33 (18%)	0 (0%)
Treatment experienced		
Dual therapy, n (%)	21 (11.6%)	1 (20%)
PI/PR, n (%)	105 (58%)	4 (80%)
Child-Pugh score		
A, n (%)	124 (68.5%)	1 (20%)
B, n (%)	43 (25.3%)	3 (60%)
C, n (%)	3 (1.7%)	1 (20%)
Median baseline HCV RNA IU/mL	6.18 MUI	1.5 MUI
Treatment duration (weeks)		
12	93 (51.4%)	0 (0%)
24	82 (45%)	0 (0%)
48	1 (0.6%)	0 (0%)
Others	5 (3%)	5 (100%)
RBV use, n (%)	61 (34%)	2 (40%)

**Abstract P293–Table 2:** Cases of multicentric and fatal HCC

	HCV	FBS (kPa)	Compensated cirrhosis	Previous Tx	DAAs	AFP	Clinical presentation	Exitus
Case 1	GT 3a ILB28 CT	36	Yes	PI/PR (1)	04/22/2015 LDV/SOF + RBV	6,48	Multicentric HCC. Portal vein thrombosis and ascites	27 days after admission/4 m DAA
Case 2	GT 1b ILB28 CT	45	Yes	PI/PR (2) Dual therapy	01/27/15 SOF/DCV	63751	Multicentric HCC. Portal vein thrombosis and ascites	6 days after admission/4 m DAA

# Sub article: P294 Characteristics and effectiveness in HCV mono-infected and HIV–HCV co-infected cirrhotic patients receiving oral direct-acting antiviral (DAA) regimens interferon-free therapies in southern Spain

**Published:** 2016-10-23

**Authors:** Begoña Alcaraz, Francisco Vera, Paloma Escribano, Rocio Rojano, Amaya Jimeno, Elena Ruiz, Ana Garcia, Javier Trujillo, Nazaret Cobos, Enrique Bernal, Mar Alcalde, Josefina Garcia, Onofre Martinez, Maria Carolina Capozzi, Lorena Martinez

**Affiliations:** 1 Medicina Interna, Hospital General Universitario Santa Lucia, Cartagena, Spain; 2 Medicina Interna, Hospital General Universitario Reina Sofia, Murcia, Spain

## Abstract

**Introduction**: Patients with liver cirrhosis due to hepatitis C virus (HCV) are a priority treatment group due to the risk of developing clinical decompensation and hepatocellular carcinoma. Oral direct-acting antiviral (DAA) interferon (IFN)-free therapies have simplified regimens and reduced adverse effects (AE), not being exempt from drug interactions. Cirrhotic patients, with more comorbidities and polymedication, could have higher risk of AE during the treatment with DAA, with compromised effectiveness.

**Objectives**: 1) To describe and analyze clinical, virologic and elastographic characteristics in cirrhotic HCV mono-infected and HIV–HCV co-infected patients who receive treatment with DAA, and 2) to describe and analyze the effectiveness and safety of DAA in the patients included.

**Methods**: Retrospective descriptive observational study of a cohort of cirrhotic HCV mono-infected and HIV–HCV co-infected patients on DAA IFN-free therapies in the Infectious Diseases Unit of the General Hospital Universitario Santa Lucia (Cartagena), from 1 January 2015 to 30 April 2016. The variables analyzed were: clinical and elastographic characteristics, comorbidities, routine treatment, efficacy rate, AE and therapeutic failures.

**Results**: From 116 patients with HCV chronic infection who initiated treatment with DAA, 31 (26.7%) were cirrhotic. Twenty-three patients were male (74%), and the mean age was 53.8±8 years. Nineteen patients (61.3%) were co-infected with HIV. The most frequent HCV genotype was 1a. The elastography median result was 20.4 kPa (p25–p75: 17–33 kPa). The mean HCV viral load (log) was 6.18±0.73 (3.75–7.19). Eighty-four percent had at least three comorbidities. Twenty-two patients consumed three or more drugs (71%), and most frequently: benzodiazepines (61%), antihypertensives (50%), proton pump inhibitors (50%), antidepressants (32%) and antipsychotics (29%). Thirteen percent of patients were on methadone program. Effectiveness analysis: 23 patients (74.2%) reached week 12 post-treatment; intention-to-treat analysis: 21 patients (91%) achieved SVR; observed data: 17 patients (74%) with SVR. Six patients presented AE (19%), of which two (33%) resulted in failure due to toxicity/intolerance. One patient had severe hepatic decompensation and deceased. Four patients failed treatment, due to virologic failure (two) and lack of adherence (two).

**Conclusions**: Patients with liver cirrhosis receiving DAA IFN-free therapies present high comorbidity and use of polypharmacy. A higher rate of severe AE was observed without a significant reduction in the virologic efficacy.

# Sub article: P295 Chronic hepatitis C treatment with direct antiviral drugs in mono- and HIV co-infected patients: a real-life experience in an infectious diseases department at a hospital in Portugal

**Published:** 2016-10-23

**Authors:** João Cabo, Teresa Martins, Nidia Garrido, Stepanka Betkova, Maria José Manata, Liliana Alves, Sara Cardoso, Orlando Cardoso, Rita Gonçalves, Ana Raquel Garrote, Pedro Sim[otilde]es, Freddy Ramirez, Diana Seixas, Sara Lino, Fernando Maltez, Diana Póvoas

**Affiliations:** 1 Infectious Diseases Department, Hospital Curry Cabral – Centro Hospitalar Lisboa Central, Lisboa, Portugal

## Abstract

**Background and aims**: Hepatitis C (HCV) treatment with direct antiviral agents (DAAs) showed high rates of sustained virological response in clinical trials. The aim of this study is to evaluate the efficacy and safety of DAAs in a real-world setting, and compare the results in HCV mono-infected and HCV/HIV co-infected patients.

**Methods**: Observational study including all HCV-infected patients starting treatment with DAAs between January 2015 and May 2016. Data were collected by review of clinical files. Liver fibrosis was evaluated by indirect methods (real time elastography, ultrasonography and biochemical markers).

**Results**: Three hundred and eighty-five patients were included, 295 (78%) HIV co-infected, 76% male and 69% aged 40 to 59 years. Mode of transmission was intravenous drugs use in 76%. Genotype (Gt) distribution was: Gt1 244 (63%); Gt2 seven (2%); Gt3a 66 (17%); and Gt4 68 (18%). Liver cirrhosis was present in 10% (38/385). Regimens used were: SOF/LDV in 298 (77%); SOF+RBV in 50 (13%); SOF/LDV+RBV in 26 (7%); other in 11 (3%). Duration of treatment was 8, 12, 16 or 24 weeks, depending on genotype, fibrosis stage and history of previous treatment, according to EASL guidelines. At the time of this analysis, 295 (76%) patients had completed treatment and data for SVR12 were available for 248 (64%). Overall SVR12 was 96%. SVR12 with SOF-based regimens was: Gt1 99% (164/166), Gt2 86% (6/7), Gt3 85% (23/27) and Gt4 91% (40/44). Regimens without SOF, used in haemodialysis patients, had 100% (4/4) SVR12. Cirrhotic patients (32/248) had 94% SVR12; average variation on MELD score for Child-Pugh A and B/C patients with SVR12 was, respectively, −0.9 and −1.4. Eleven patients experienced treatment failure: six treatment experienced; one F4; no relation with HIV infection status – 4% (8/189) in HIV versus 5% (3/59) non-HIV patients. Treatment was well tolerated. Thirty-six percent reported at least one adverse event (AE), the most common being asthaenia (12%) and headache (9%). None discontinued. ART was changed in 10 patients before or during HCV treatment, in seven due to renal AEs. Tenofovir-based regimens were used in 149 HIV-infected patients starting SOF or SOF/LED therapy, prompting changes on ART in six (4%).

**Conclusions**: HCV treatment with DAAs showed high efficacy and good tolerability. SVR12 rate (96%) was consistent with data from clinical trials. High rate of SVR12 and improvement in liver function was observed in cirrhotic patients. Failure was not related with HIV co-infection. Regular monitoring of renal function is needed in patients on tenofovir starting SOF or SOF/LDV.

# Sub article: P296 Chronic HCV treatment with DAA: what changes when treating cirrhotic patients – a Portuguese cohort real-life experience

**Published:** 2016-10-23

**Authors:** Ana Claudia Miranda, Telma Azevedo, Inês Cruz, Fernando Ventura, Teresa Baptista, Fernando Borges, Susana Peres, Isabel Aldir, Jaime Nina, Isabel Antunes, Maria José Campos, João Pereira, Kamal Mansinho

**Affiliations:** 1 Serviço de Infecciologia e Medicina Tropical, Hospital Egas Moniz – Centro Hospitalar de Lisboa Ocidental, Lisbon, Portugal; 2 Serviço de Medicina Interna 2, Hospital Egas Moniz – Centro Hospitalar de Lisboa Ocidental, Lisbon, Portugal; 3 Serviço de Medicina Interna 1, Hospital Egas Moniz – Centro Hospitalar de Lisboa Ocidental, Lisbon, Portugal

## Abstract

**Introduction**: Cirrhosis and hepatic carcinoma represents the ultimate stage of HCV infection implying a negative impact on patients’ quality of life, increase morbidity and mortality. Hepatic cirrhosis present as a wide histopathologic findings and early HCV infection diagnosis and treatment are major objectives in order to treat infection before hepatic severe fibrosis establishment. Since early 2015, Portugal is living a favourable scenario that allows the prescription of reimbursed DAA regimens, including sofosbuvir (SOF), sofosbuvir/ledipasvir (SOF/LDP) coformulation, and most recently, ombitasvir/pariteprevir/ritonavir plus dasabuvir combination (3D), with or without ribavirin (RBV) association.

**Material and methods**: Demographic, epidemiologic, clinical, virologic and treatment response data of HCV chronic infected cirrhotic patients, who were eligible to start treatment with DAA regimens, were collected, during the period between 1 January 2015 and 30 June 2016. Hepatic fibrosis was assessed by real-time elastography together with APRI and FIB-4 serum biomarkers determination. Cirrhosis was considered for those with METAVIR F4 and for those with F3 plus high APRI or FIB-4 scores.

**Results**: During the inclusion period, 153 chronically HCV-infected cirrhotic patients started treatment with DAAs: 100 (65%) HCV/HIV co-infected and 53 (35%) HCV mono-infected. Demographic and epidemiologic characterization of both groups (HCV/HIV vs. HCV) revealed: male predominance in 80% versus 64%, mean age of 49 years versus 55 years, parenteral HCV transmission in 74% versus 56% and mean time since HCV diagnosis of 15 years versus 9.8 years. HCV infection staging evidenced (HCV/HIV vs. HCV): genotype 1 was the most frequent (68% vs. 60%); CC IL28B gene polymorphism in 39% vs. 32% and mean HCV plasma RNA of 6.48 log_10_ versus 6.4 log_10_. Clinical scores Child-Pugh and MELD evidenced compensated hepatic disease in the vast majority of patients: 83% versus 96% Child-Pugh A and 77% versus 89% MELD <10. Co-infected patients presented a mean TCD4 count of 601 cells/mm^3^ and 88% had undetectable HIV plasma RNA. At the present time, 72% versus 42% patients completed treatment and 53% versus 34% performed HCV RNA determination at 12 weeks after treatment ending, revealing a SVR12 of 93% versus 95%. The reported rate of adverse events was 36% versus 44%, with one death considered not related with pharmacologic reasons, documented in a HCV/HIV patient.

**Conclusion**: Our cohort of chronically HCV-infected patients, with advanced liver disease, achieved high rates of SVR12. HCV/HIV co-infected patients presented with a younger age and with a longer time since HCV diagnosis, eventually corresponding to an earlier refer to healthcare.

# Sub article: P297 Chronic hepatitis C in HIV-infected patients: treatment in real-life with new generation direct-acting antivirals in a Portuguese centre

**Published:** 2016-10-23

**Authors:** André Silva-Pinto, Rogério Ruas, Isabel Abreu, Filipa Ceia, Cristóvão Figueiredo, Ana Claúdia Carvalho, Ana Sofia Santos, Jorge Soares, Carmela Piñeiro, Rosário Serrão, António Sarmento

**Affiliations:** 1 Infectious Diseases, Centro Hospitalar São João, Porto, Portugal

## Abstract

**Introduction**: The direct-acting antivirals (DAA) revolutionized the hepatitis C virus (HCV) infection treatment. Since 2015, DAA became available in Portugal due to a universal access policy. Its use in HIV-infected patients has some peculiarities, especially due to drug interactions. Our aim is to assess the DAA efficacy in real life and to monitor fibrosis and alpha-fetoprotein (AFP) changes with treatment.

**Methods**: We performed a retrospective study of HCV-HIV patients treated with DAA between February 2015 and June 2016. The primary outcome was sustained virological response (SVR) at week 12 after treatment and the secondary outcomes were changes in fibrosis, transaminases and AFP. We used the most appropriate measures of central tendency/distribution and statistical test.

**Results**: We included 236 patients that completed 12 weeks after treatment evaluation (from 402 patients with chronic HCV-HIV, 275 started the DAA treatment). Ninety percent (n=212) were men and the mean age was 47.4 (SD 6.6). The majority acquired the infection through drug abuse (91.5%) and 64% were HCV treatment naïve. The most frequent HCV genotype was 1 (77.1%), followed by 3 (10.2%). The cirrhotic rate was 30%. Concerning the HIV treatment, 99% of patients were on treatment. In 68 patients (28.8%) the HIV treatment had to be changed due to drug interactions: efavirenz (38.3%), boosted atazanavir (27.9%), boosted lopinavir (23.5%), nevirapine (8.8%) and boosted elvitegravir (1.5%). The patients changed the HIV treatment to raltegravir, rilpivirine, boosted darunavir or dolutegravir. Regarding HCV treatment, the majority did sofosbuvir/ledipasvir (83.9%), followed by sofosbuvir/ribavirin (9.3%), sofosbuvir/ledipasvir/ribavirin (5.9%) and sofosbuvir/daclatasvir/ribavirin (0.8%). The duration of treatment was 12 weeks in 49.6% and 24 weeks in 50.4%. Our SVR rate was 97% (229 patients cured the infection): four patients did not cure the infection owing to lack of adherence, in two patients the treatment was stopped (acute renal failure and alveolar haemorrhage, respectively) and one patient had a relapse. There were no statistically significant differences in the HIV viral load or CD4 count during the treatment (evaluated at 0, 4 and 12 weeks, end of treatment and 12 weeks after treatment) (Friedman test). The elastography stiffness, AFP and transaminases decreased in a statistically significant manner comparing before and after treatment (Wilcoxon test).

**Conclusion**: Our results confirm in real life the efficacy of these DAA with improvement of fibrosis, hepatic cytolysis and AFP.

# Sub article: P298 Impact of HCV RNA kinetics in SVR12 in a cohort of HIV/HCV co-infected patients treated with DAAs in a “real-life” setting

**Published:** 2016-10-23

**Authors:** Silvia Cavinato, Silvia Zuin, Giulia Marini, Francesco Barbaro, Saverio Parisi, Annamaria Cattelan

**Affiliations:** 1 Infectious and Tropical Diseases Unit, Azienda Ospedaliero-Universitaria di Padova, Padua, Italy; 2 Department of Molecular Medicine, University of Padua, Padua, Italy

## Abstract

**Introduction**: Direct-acting antiviral (DAA) drugs created a major paradigm shift in the treatment of chronic hepatitis C in HIV/HCV co-infected patients. The aim of this study was to evaluate the effectiveness and safety of DAA therapy and the role of on-treatment HCV RNA kinetics on virological outcome in a “real-life” setting.

**Materials and methods**: All consecutive HIV/HCV co-infected patients starting DAAs from May 2015 to March 2016 at our HIV Outpatient Clinic were evaluated. Baseline characteristics, safety data, sustained virological response at 12 weeks after end of treatment (SVR12) and HCV RNA at 8 hours/48 hours/week 1/week 2/week 4/week 8 and monthly thereafter were assessed.

**Results**: Forty-nine patients were treated. Demographic, clinical/virological characteristics at baseline and DAA regimens are shown in Table 1.

To date, 31 patients completed 12 weeks of follow-up after end of treatment. Thirty patients (97%) achieved SVR12 and one patient (3%) failed to obtain HCV RNA undetectability at 12 weeks after end of treatment. Thirteen patients achieved HCV RNA <12 UI/mL at the end of treatment and are still waiting for SVR12. Three patients died during treatment; one patient died for recurrence of hepatocellular carcinoma after end of treatment, one patient discontinued therapy at week 3 for a severe adverse event (psychotic syndrome). The other most common side effects were asthaenia 28%, insomnia 26% and anaemia 10%. HCV RNA declined rapidly after the initiation of DAAs with significant decay at 8 and 48 hours from first dose of therapy; mean HCV RNA at 8 hours was 370.039 UI/mL (−80.6% from baseline), at 48 hours was 2.592 UI/mL (−99.6% from baseline; p<0.05). No significant correlation was found between viral load decay during first hours of therapy and time of first HCV RNA undetectability (r: −0.095 at 8 hours, −0.18 at 48 hours). Different patterns of viral response were observed: (a) rapid response: HCV RNA <12 UI/mL at week 4: 10 patients (21%), (b) HCV RNA >12 UI/mL at week 4 and HCV RNA <12 UI/mL at week 8: 7 patients (15%), (c) slow response: HCV RNA >12 UI/mL at week 8 and HCV RNA <12 UI/mL at week 12 or end of treatment: 27 patients (60%).

**Conclusions**: In our HIV–HCV co-infected patients, DAAs confirmed to be highly effective and well tolerated both in treatment-naïve and treatment-experienced, including those with cirrhosis. A significant decay of HCV RNA during first hours of therapy did not correlate with time of HCV RNA undetectability as well as different patterns of HCV RNA kinetics did not show a relation with subsequent achievement of SVR12.

**Abstract P298–Table 1:** Demographic, clinical and virological characteristics of patients at baseline and DAAs regimens

Demographic characteristics		Total number of 49 patients
Male gender		35 (72%)
Age (years)		52 (range 26–64)
HIV/HCV risk factor		
	PWIDs	39 (80%)
	Transfusion	2 (5%)
	MSM	2 (5%)
	Vertical transmission	1 (2%)
	Unknown	4 (8%)
HIV characteristics		
CDC stage		
	A	9 (19%)
	B	17 (35%)
	C	23 (46%)
Under antiretroviral treatment		49 (100%)
HIV RNA <20 copies/mL		46 (93%)
CD4 count (mean, cells/µL)		607 (±103)
Change of antiretroviral treatment before start of DAAs		29 (60%)
Antiretroviral regimens during DAAs		
	Raltegravir based	28 (58%)
	PI based	11 (21%)
	Tenofovir/emtricitabine/rilpivirine	7 (14%)
	Dolutegravir based	3 (7%)
HCV characteristics		
HCV treatment-experienced		24 (49%)
Liver fibrosis (FibroScan)		
	F0–F1	0 (0%)
	F2	10 (21%)
	F3	10 (21%)
	F4	29 (58%)
HCV genotype		
	GT1a	15 (30%)
	GT1b	9 (19%)
	GT1	1 (2%)
	GT2	1 (2%)
	GT3	9 (19%)
	GT4	14 (28%)
HCV RNA (mean, UI/mL)		234,1817 (±754,312)
DAA regimens		
	Ombitasvir/paritaprevir/ritonavir + dasabuvir (3D) + ribavirin	21 (42%)
	Ledipasvir/sofosbuvir (±ribavirin)	15 (30%)
	Daclatasvir + sofosbuvir + ribavirin	9 (19%)
	Ombitasvir/paritaprevir/ritonavir (2D) + ribavirin	3 (7%)
	Sofosbuvir + ribavirin	1 (2%)
Use of ribavirin in DAA regimens		41 (84%)
Duration of DAA therapy		
	12 weeks	22 (44%)
	24 weeks	27 (56%)

# Sub article: P299 CD4 cell count before chronic hepatitis C (HCV) treatment with direct-acting antivirals (DAAs) and at the end of follow-up in HCV/HIV co-infected patients

**Published:** 2016-10-23

**Authors:** Josefina Mendez, Cristina Soeiro, Marta Marques, Celina Gonçalves, Ana Paula Tavares, Ana Horta, Olga Vasconcelos, João Seabra, Miguel Abreu, Rui Sarmento e Castro

**Affiliations:** 1 Infectious Diseases, Centro Hospitalar do Porto, Porto, Portugal

## Abstract

**Introduction**: Treatment of chronic hepatitis C (HCV) with interferon-based regimens was associated with a decrease in the CD4 cell count in HCV–HIV co-infected patients, which returned to baseline after the end of treatment. HCV cure with those regimens was associated with a progressive increase in CD4 cell count.

**Aim**: To assess the impact of HCV treatment with DAAs on CD4 cell count.

**Methods**: Prospective study of HCV/HIV co-infected patients treated with DAAs for chronic hepatitis C. CD4 cell counts at baseline, at the end of treatment and 12 weeks after treatment were compared. The patients were randomized based on CD4 count at baseline lower or equal and higher than 350 CD4/mm^3^.

**Results**: We included 133 patients: 88.7% were male, the mean age was 46 years old and the acquisition of HCV was by intravenous drug use in 94%. The most frequent genotype was G1 (79.7%), followed by G4 (11.3%), G3 (8.3%) and G2 (0.8%). Overall, 41.4% were treatment experienced. Mean value of fibrosis was 18.4 kPa and 52.6% of patients were cirrhotic. All patients were receiving antiretroviral treatment and all had undetectable HIV RNA. The mean baseline CD4 count was 603/mm^3^. Of the patients with <350 CD4/mm^3^, 78% were cirrhotic. Sofosbuvir and ledipasvir ± ribavirin was prescribed in 88.7% of patients. Other regimens were sofosbuvir + ribavirin (6.8%), ombitasvir/paritaprevir/ritonavir + dasabuvir (2.3%), sofosbuvir + daclatasvir + ribavirin (1.5%) and sofosbuvir + pegylated interferon (0.8%). SVR12 rate was 97% (four patients relapsed). The evolution of the CD4 count during treatment is shown in Table 1.

The evolution of the CD4 count at the end of follow-up is shown in Table 2.

**Conclusion:** There is a global increase in the mean CD4 cell count during and after HCV treatment. The increase of CD4 cell count after treatment is greater in patients with less than 350 CD4 cells/mm^3^ possibly due to the higher proportion of cirrhotic patients (78%) in this group.

**Table 1:** CD4 cell count evolution during HCV treatment with DAAs

	Mean baseline CD4 cell count (mm^3^)	Mean end of treatment CD4 cell count (mm^3^)	p
Total (n=87)	629	646	0.408
≤350 CD4/mm^3^ (n=13)	253	285	0.196
>350 CD4/mm^3^ (n=74)	695	710	0.547

**Table 2:** CD4 cell count evolution at the end of follow-up

	Mean baseline CD4 cell count (mm^3^)	Mean end of follow-up CD4 cell count (mm^3^)	p
Total (n = 126)	597	638	0.408
≤350 CD4/mm^3^ (n = 27)	242	290	<0.05
>350 CD4/mm^3^ (n = 99)	694	732	0.063

# Sub article: P300 Testing for HCV and HIV at baseline visit due to post-exposure prophylaxis

**Published:** 2016-10-23

**Authors:** Justyna Kowalska, Ewa Pietraszkiewicz, Ewa Firlag-Burkacka, Karolina Pyziak-Kowalska, Andrzej Horban

**Affiliations:** 1 Hospital for Infectious Diseases in Warsaw, Medical University of Warsaw, Warszawa, Poland; 2 HIV Outpatient Clinic, Hospital for Infectious Diseases in Warsaw, Warszawa, Poland

## Abstract

**Introduction**: People consulted for post-exposure prophylaxis (PEP) can be at higher risk for HCV and/or HIV infection due to earlier exposures, lifestyle or occupation. Here, we evaluate the positivity rate of anti-HCV and anti-HIV/p24 in this population, as well as factors related to it.

**Methods**: We performed retrospective analyzes of consultations due to PEP in HIV Outpatient Clinic in Warsaw. Data were obtained from electronic database, which collects all medical information since 2007. Logistic regression models were used to identify factors related to positive anti-HCV test (all with p<0.01 in univariate included in final model). Due to low positivity rate, HIV was not included in the model.

**Results**: In total, 3928 persons were tested for both HIV and HCV in 2008 to 2016, 2231 (56.8%) women, median age 33.4 (26.3–43.4) years. Hundred and fifty (4.9%) persons had elevated ALT level (of 3032 measured at baseline), 161 (5.4%) had elevated AST (of 2961 measured), median PLT was 248 (213–296) 10^3^/µL (for 484 measured). Eighty-one (2.1%) persons were anti-HCV positive, 34 (1.0%) HBsAg positive and 4 (0.1%) anti-HIV/p24 positive. Two persons were both HCV and HBsAg positive, one both anti-HIV/p24 and HCV positive. The final multivariate model included 3912 patients (80 HCV positive) and is presented in Table 1.

**Conclusions**: The rate of positive anti-HCV tests at baseline PEP visit was comparable with the one observed in general Polish population, despite higher baseline risks in this group of patients. Positive HCV test was associated with traditional factors, namely age and elevated liver enzymes. The next step in the project is to review possible risk behaviours described at baseline visit in order to optimize HCV testing patterns.

**Abstract P300–Table 1:** Logistic regression odds ratios for having anti-HCV positive test at baseline screening

	Univariate		Multivariate	
		
Effect	OR (95% CI)	p	OR (95% CI)	p
Female gender	0.44 (0.28–0.69)	0.0004	0.63 (0.34–1.18)	0.148
AST > ULL	9.18 (4.85–17.4)	<0.0001	5.15 (2.22–11.9)	0.0001
ALT > ULL	8.62 (4.52–16.4)	<0.0001	3.01 (1.25–7.28)	0.014
Age of first visit per each 10 years older	1.19 (1.01–1.42)	0.039	1.36 (1.08–1.71)	0.009
Year 2011–2013 versus 2008–2010	0.93 (0.46–1.90)	0.131	0.56 (0.19–1.65)	0.068
Year of test 2014–2016 versus 2008–2010	1.98 (1.04–3.76)	0.002	1.31 (0.49–3.49)	0.092
HBsAg positive versus negative	3.17 (0.74–13.5)	0.173	–	–
HBsAg unknown versus negative	1.31 (0.75–2.29)	0.497	–	–

All with p < 0.01 in univariate included in final model. HIV test not included due to low number of events.

# Sub article: P301 Interactions between HIV and HCV therapies: how common and who wins?

**Published:** 2016-10-23

**Authors:** Adele Torkington, Justine Barnes, Sarah Cripps, Karen Lee, Neal Marshall, Helen Morgan, Anthony Pratt, Ian Sayers, Jackie Swabe, Leonie Swaden, Paul Selby, Tina Vaghjiani, Matthew Zimmer, Sanja Bhagani, Jane Collier, Douglas Macdonald, Mark Nelson, Martin Prince, Mark Wright, Andrew Ustianowski

**Affiliations:** 1 Pharmacy, North West ID Unit, North Manchester General Hospital, Manchester, UK; 2 Pharmacy, Heartlands Hospital, Birmingham, UK; 3 Pharmacy, Oxford University Hospitals NHS Foundation Trust, Oxford, UK; 4 Pharmacy, Central Manchester University Hospitals, Manchester, UK; 5 Ian Charleson Centre for HIV Medicines, Royal Free London, London, UK; 6 Pharmacy, Chelsea and Westminster Hospital, London, UK; 7 Pharmacy, York Teaching Hospitals NHS Foundation Trust, York, UK; 8 Pharmacy, University Hospital Southampton NHS Foundation Trust, Southampton, UK; 9 Pharmacy, Cambridge University Hospital, Cambridge, UK; 10 Pharmacy, Royal Free London, London, UK; 11 Gastroenterology, Oxford University Hospitals NHS Foundation Trust, Oxford, UK; 12 Hepatology, Royal Free London, London, UK; 13 HIV and Sexual Health Kobler Clinic, Chelsea and Westminster Hospital, London, UK; 14 Hepatology, Central Manchester University Hospitals, Manchester, UK; 15 Hepatology, University Hospital Southampton NHS Foundation Trust, Southampton, UK; 16 Infectious Diseases, North West ID Unit, North Manchester General Hospital, Manchester, UK

## Abstract

**Introduction**: The current era of HCV direct-acting antivirals (DAAs) has allowed HIV–HCV co-infected patients to achieve similar rates of response to HCV mono-infected patients [1]. Managing HIV–HCV therapy is complex, often involving drug–drug interactions (DDIs) between the DAAs, ARVs and other medicines. We evaluated the incidence of DDIs in co-infected patients and its impact on choice of preferred HCV therapy as recommended by NHS England.

**Material and methods**: Retrospective evaluation of all HIV–HCV co-infected patients receiving DAAs seen across nine UK centres from June 2015 till May 2016. Data were collected on demographics, HCV genotype, choice of DAA and ARVs and any changes made to these or additional monitoring required. The Liverpool hep-druginteractions.org website [2] was used to evaluate the presence and severity of potential drug interactions.

**Results**: Hundred and eighty-three patients were identified of which 163/183 (89%) were male and median 49 years old. Hundred and forty-eight of 183 (81%) were HCV genotype 1, 17 (9%) genotype 4, 15 (8%) genotype 3 and two with genotype 2, with 78/183 (43%) reporting cirrhosis. Eighty-eight of 183 (48%) were non-responders or relapses to prior HCV therapy. The HIV and HCV regimens are listed in Tables 1 and 2, respectively. Twenty-nine of 183 (16%) required alteration to their HIV regimen prior to DAA therapy (Table 3). Twenty-two of 29 (76%) of which received Abbvie 2D/3D (ritonavir)-based DAA. Six of 183 (3%) required adaptation of HCV regimen due to current ART regimen. Six hundred and ninety-four comedicines were identified in 146/183 (80%) patients (median 3.5/patient). Hundred and seventy-two of 694 (25%) amber DDIs (close monitor/dosage adjustment required) were identified in 56/183 (31%) of patients, with 17/694 (2%) red (do not co-administer) observed for 14/183 (8%) of patients. The need for additional monitoring was reported for 61/183 (33%) of patients due to potential DDIs with the DAA chosen, including renal monitoring for tenofovir/ledipasvir co-administration, monitoring of INR for warfarin, blood pressure and lipids.

**Conclusion**: Managing HIV–HCV co-infected patients is clearly complex requiring review and modification of both HIV and HCV therapy with additional monitoring. The majority received what was deemed first-line HCV therapy as per national prescribing guidelines at the time of initiation of HCV treatment. The role of the specialist pharmacy team is key to managing this cohort.

**Table 1:** HIV regimens

HIV regimen	Number (%) patients
Integrase inhibitor	72 (39)
Protease inhibitor (PI)	55 (30)
Non-nucleoside reverse transcriptase inhibitor (NNRTI)	39 (21)
Complex regimens	15 (8)
Not on treatment	2 (1)

**Table 2:** HCV regimens

HCV regimen	Number (%) of patients
Harvoni±ribavirin	102 (56)
Abbvie 3D±ribavirin	32 (17)
Sofosbuvir/pegIFN/ribavirin	27 (15)
Sofosbuvir/daclatasvir±ribavirin	9 (5)
Abbvie 2D±ribavirin	8 (4)
Other	5 (3)

**Table 3:** ARV changes

ARV change	Number (%) of patients
Omit ritonavir on Abbvie 2D/3D	12 (7)
NNRTI to unboosted integrase	10 (5)
PI to unboosted integrase	2 (1)
Boosted integrase converted to unboosted integrase	1 (1)
PI changed	1 (1)
Other	3 (2)

## References

[1] Arends JE, Lieveld FI, Boeijen LL, de Kanter CT, van Erpecum KJ, Salmon D (2015). Natural history and treatment of HCV/HIV coinfection: is it time to change paradigms?. J Hepatol.

[2] http://www.hep-druginteractions.org/.

# Sub article: P302 Efavirenz significantly decreases etonogestrel exposure: results of a bidirectional pharmacokinetic evaluation of efavirenz- and nevirapine-based antiretroviral therapy plus etonogestrel contraceptive implants

**Published:** 2016-10-23

**Authors:** Catherine Chappell, Kimberly Scarsi, Shadia Nakalema, Beatrice Chen, Sharon Riddler, Susan Cohn, Kristin Darin, Sharon Achilles, Mohammed Lamorde

**Affiliations:** 1 Obstetrics, Gynecology and Reproductive Sciences, University of Pittsburgh, Pittsburgh, PA, USA; 2 Pharmacy Practice, University of Nebraska Medical Center, Omaha, NE, USA; 3 Infectious Diseases Institute, Makerere University College of Health Sciences, Kampala, Uganda; 4 Medicine, University of Pittsburgh, Pittsburgh, PA, USA; 5 Feinberg School of Medicine, Northwestern University, Chicago, IL, USA

## Abstract

**Introduction**: Increasingly, women in Sub-Saharan Africa are choosing etonogestrel (ENG) contraceptive implants because they are highly effective and well tolerated. However, implantable contraceptive failures are reported in HIV-positive women due to drug-contraceptive interactions with efavirenz (EFV)-based ART [1–3]. We characterized ENG pharmacokinetics in HIV-positive Ugandan women receiving ENG implants plus EFV- or nevirapine (NVP)-based ART compared with ART-naïve women. To explore the potential for a bidirectional interaction, we compared EFV and NVP concentrations before and after ENG implant insertion.

**Material and methods**: This non-randomized, parallel-group study included three arms: ART-naïve, EFV- or NVP-based ART (N=20 per group). Participants in the ART groups were on stable ART with an undetectable HIV RNA at entry. Sparse pharmacokinetic sampling of ENG, EFV and NVP were performed at screening, entry and then 1, 4, 12 and 24 weeks post-implant insertion. In the ART groups, plasma was collected 12 to 14 hours post-EFV or 11 to 13 hours post-NVP dose. Participants on EFV-based ART had copper intrauterine devices in place at entry. ART and ENG concentrations were quantitated using validated HPLC and HPLC-MS/MS methods, respectively. Data are compared as geometric mean ratio (GMR), with 90% CI.

**Results**: At entry, study groups were similar in age, weight and CD4 count. Data from 58 participants are included; one participant each was excluded from the EFV (ART non-adherence) and NVP (processing error) groups. Geometric mean (GM) ENG area under the curve (AUC) from 0 to 24 weeks (AUC_0–24_) were 11.12, 1.80 and 10.47ng*wk/mL in the ART-naïve, EFV and NVP groups, respectively (AUC_0–24_ GMR: EFV: ART-naïve 0.162 (0.160–0.163); NVP: ART-naïve 0.941 (0.938–0.944)). EFV and NVP concentrations were lower at week 24 compared with pre-implant (EFV: GM 3.6 vs. 4.7 mg/L, respectively, GMR 0.76 (0.71–0.79), p=0.009; NVP: GM 5.7 vs. 6.9 mg/L, respectively, GMR 0.83 (0.78–0.88), p=0.227). No participant in the EFV group and one participant in the NVP group had concentrations below the suggested threshold for virologic suppression at week 24 (EFV <1 mg/L and NVP <3 mg/L).

**Conclusions**: Over 24 weeks of combined use, ENG exposure was 84% lower in women using EFV-based ART compared with ART-naïve women. In contrast, NVP did not significantly impact ENG exposure. Similar to findings with the levonorgestrel implant [1], decreased ENG exposure in combination with EFV raises concerns about reduced implantable contraceptive effectiveness for women on EFV-based ART. Although statistically lower EFV concentrations were observed after ENG insertion, all participants in the EFV group had therapeutic concentrations at week 24.

## References

[1] Scarsi KK, Darin KM, Nakalema S, Back DJ, Byakika-Kibwika P, Else LJ (2016). Unintended pregnancies observed with combined use of the levonorgestrel contraceptive implant and efavirenz-based antiretroviral therapy: a three-arm pharmacokinetic evaluation over 48 weeks. Clin Infect Dis.

[2] Patel RC, Onono M, Gandhi M, Blat C, Hagey J, Shade SB (2015). Pregnancy rates in HIV-positive women using contraceptives and efavirenz-based or nevirapine-based antiretroviral therapy in Kenya: a retrospective cohort study. Lancet HIV.

[3] Vieira CS, Bahamondes MV, de Souza RM, Brito MB, Rocha Prandini TR, Amaral E (2014). Effect of antiretroviral therapy including lopinavir/ritonavir or efavirenz on etonogestrel-releasing implant pharmacokinetics in HIV-positive women. J Acquir Immune Defic Syndr.

# Sub article: P303 Dolutegravir plasma concentrations according to companion antiretroviral drug: unwanted drug interaction or desirable boosting effect?

**Published:** 2016-10-23

**Authors:** Dario Cattaneo, Davide Minisci, Valeria Cozzi, Agostino Riva, Paola Meraviglia, Emilio Clementi, Giuliano Rizzardini, Cristina Gervsasoni

**Affiliations:** 1 Unit of Clinical Pharmacology, Luigi Sacco University Hospital, Milano, Italy; 2 Infectious Diseases, Luigi Sacco University Hospital, Milano, Italy

## Abstract

**Introduction**: Main pharmacologic advantages of dolutegravir compared with other integrase inhibitors are its suitability for once-daily administration, no need for pharmacokinetic boosting, a high barrier to resistance and its modest drug-to-drug interaction profile. It should be pointed out, however, that most of these features derive from studies in healthy volunteers. Here we aimed to characterize the pharmacokinetics of dolutegravir in a real-life setting.

**Materials and methods**: Consecutive HIV-infected patients treated with dolutegravir for at least one month with a request for monitoring of dolutegravir plasma concentrations were considered. Drug concentrations were assessed by a validated HPLC-UV method. Dolutegravir trough concentrations were estimated using the interval between last dose intake and blood sampling and the drugs elimination half-life and clustered according to the companion antiretroviral drug.

**Results**: Sixty HIV-infected adult patients were included in the study. All patients had good immunologic status (CD4 cell count 660±325 cells/mL) with no clinical signs of liver or kidney dysfunction. Patients were given dolutegravir at 50 mg once daily, with mean sparse and trough drug concentrations of 2553±1932 and 1507±1283 ng/mL, respectively. Dolutegravir was given in combination with atazanavir (n=25), darunavir (n=13), a non-nucleoside reverse transcriptase inhibitor (n=12, rilpivirine or etravirine) or abacavir/emtricitabine (n=10). By multivariate analyzes, only companion antiretroviral drug resulted significantly associated with dolutegravir plasma trough concentrations (p=0.012). Indeed, patients given dolutegravir with atazanavir had three- to four-fold higher drug concentrations compared with those given darunavir, a non-nucleoside reverse transcriptase inhibitor or abacavir/emtricitabine (2918±1411 vs. 617±286, 932±965 or 1168±822 ng/mL, p<0.001 for all comparisons, Figure 1).

**Conclusions:** Here we have documented that co-administration of atazanavir resulted in highly significant increased dolutegravir concentrations compared with other antiretroviral drugs. These results partially challenge previous findings in healthy volunteers which showed that concomitant atazanavir/ritonavir intake produced only modest, non-clinically significant increase in dolutegravir exposure. This drug-to-drug interaction (related to the atazanavir-mediated inhibition of UDP-glucuronosyltransferase 1A1, the main enzyme involved in the metabolism of dolutegravir) could become relevant in all clinical conditions which require higher than conventional dolutegravir exposure. Moreover, the administration of dolutegravir with atazanavir/ritonavir might also improve the exposure of poorly compliant patients to antiretroviral therapy. No association between high dolutegravir concentrations and the development of drug-related adverse events or toxicity has been reported to date. Therefore, it remains to be established whether the increased dolutegravir exposure observed in HIV-infected patients might eventually translate in late tolerability to treatment.

**Figure 1:** Dolutegravir plasma trough concentrations clustered according to the companion antiretroviral. Boxplots and whiskers represent the 5th, 25th, 50th, 75th and 95th percentiles, respectively. **p < 0.001 versus other groups. Dashed lines depict the protein-adjusted 90% inhibitory concentration for wild-type and resistant viruses (64 and 640 ng/mL, respectively).

# Sub article: P304 Determinants of dolutegravir plasma concentrations in the clinical setting

**Published:** 2016-10-23

**Authors:** Chiara Alcantarini, Andrea Calcagno, Letizia Marinaro, Micol Ferrara, Maurizio Milesi, Alice Trentalange, Ambra Barco, Chiara Montrucchio, Amedeo De Nicolò, Alessandra Ariaudo, Fabio Favata, Antonio D'Avolio, Giovanni Di Perri, Stefano Bonora

**Affiliations:** 1 Department of Medical Sciences, Unit of Infectious Diseases, University of Turin, Torino, Italy

## Abstract

**Introduction**: Dolutegravir (DTG) is the latest available integrase strand transfer inhibitor. It is primarily metabolized via UGT1A1 with CYP 3A4 as a minor pathway and it is substrate of p-glycoprotein. Few drug-to-drug interactions have been observed but data on DTG pharmacokinetics (PK) in the clinical setting are limited.

**Materials and methods**: The Torino Therapeutic Drug Monitoring (TDM) registry was used and patients on DTG, with fully available data (demographic, time after dose and concomitant medications), were included; patients on rifampin were excluded. Data are described as medians (interquartile ranges) and analyzed through non-parametric tests. A multivariate linear regression analysis was performed including variables with p-values below 0.10 at univariate tests.

**Results**: Three hundred and sixty-three samples were available from 149 patients (median 1, range 1–19 per patient). Median age and body mass index were 49.3 years (46.4–54.5) and 24.2 kg/m^2^ (20.8–27.7); 102 patients (68.4%) were male and 50 (33.5%) were HCV positive. Samples were withdrawn 22.5 hours (10.8–24.2) after drug intake (198 (54.5%) were trough values) and DTG median concentrations were 1107 ng/mL (399–2549). Three hundred and twenty-four (91.3%) and 31 (8.7%) samples were from patients on once-daily or twice-daily DTG: respective trough values were 660 ng/mL (255–1237) and 2674 ng/mL (1000–3474). Inter-patient variability was high (102%) and lower in patients on twice-daily DTG (56.9% vs. 108%); intra-patient variability (calculated in 10 patients with >4 trough samples, all on once-daily DTG) was 64.7%. A moderate significant correlation was observed between DTG concentrations and age (rho 0.21 and p<0.001, rho 0.58 and p<0.001 considering C_max_). Higher concentrations were observed in patients on atazanavir (2321 vs. 922 ng/mL, p<0.001) while borderline lower in those on valproic acid (n=7, 829 vs. 1132 ng/mL, p=0.08). At multivariate linear regression analysis age, post-dose time, atazanavir use (p<0.001) and, borderline, valproic acid use (p=0.06) were independent predictors of DTG concentrations explaining approximately 46% of its variability.

**Conclusions**: DTG PK in the clinical setting showed significant variability although resulted in the range of efficacy. Significant higher exposure was confirmed with atazanavir, while unexpected higher drug exposure in older patients and lower concentrations in valproic acid intakers need to be confirmed in further studies.

# Sub article: P305 Pharmacokinetics (PK) of darunavir/ritonavir (DRV/RTV) with tenofovir DF/emtricitabine (TDF/FTC) or raltegravir (RAL) in HIV-infected adults enrolled in the NEAT001/ANRS143 study and relationship with virological response

**Published:** 2016-10-23

**Authors:** Stefano Bonora, Wolfgang Stohr, Antonio D'Avolio, Adam Cursley, Jean-Michel Molina, Gerd Faetkenheuer, Linos Vandekerckhove, Giovanni Di Perri, Anton Pozniak, Laura Richert, Francois Raffi, Marta Boffito

**Affiliations:** 1 Unit of Infectious Diseases, University of Turin, Turin, Italy; 2 Medical Research Council, University College London, London, UK; 3 Infectious Diseases, University of Paris Diderot, Paris, France; 4 Unit of Internal Medicine, University Köln, Köln, Germany; 5 HIV Translational Research Unit, Ghent University and Ghent University Hospital, Ghent, Belgium; 6 St Stephen's Centre, Chelsea and Westminster NHS Trust, London, UK; 7 INSERIM ISPED, University of Bordeaux, Bordeaux, France; 8 Infectious and Tropical Diseases, Nantes University Hospital, Nantes, France

## Abstract

**Introduction**: Limited prospective PK data are available on DRV/RTV once daily (OD) and RAL twice daily (BD) in ARV-naïve HIV-infected individuals. We here present the PK analysis performed in the NEAT001/ANRS143 study.

**Materials and methods**: NEAT 001/ANRS143 96-week randomized study demonstrated non-inferiority of first-line ART with DRV/RTV (800/100 mg OD) plus RAL (400 mg BD) compared with DRV/RTV plus TDF/FTC (245/200 mg OD). However, higher failure rates in the RAL arm were seen in those with low CD4 counts and high viral load (VL) at baseline. Blood for PK analysis of study drugs (TFV, FTC, DRV, RTV, and RAL) was collected 4 and 24 weeks after ARV initiation. Only samples drawn between 5 and 30 hours post-dose were included in this analysis. Drug concentrations were log-transformed, and linear regression analysis was used to examine possible determinants of DRV concentrations (age, gender, weight, ethnicity, RTV, RAL), adjusted for time post-dose. We also examined if DRV concentration was lower in patients with CD4 <200 cells/µL or VL ≥100,000 copies/mL at baseline. Cox regression was used to associate week 4 drug levels with virological failure at or after week 32 (defined as confirmed VL ≥50 copies/mL), adjusting for baseline VL.

**Results**: Six hundred and sixty participants provided 1146 plasma samples with DRV concentrations (317 on RAL plus DRV/RTV, 343 on TDF/FTC plus DRV/RTV). Eighty-nine percent were males, 83% white; median (IQR) age and weight were 37 (31–46) years and 72 (65–80) kg, respectively. Two hundred and ninety-nine participants provided 483 RAL measurements, 658 provided 1138 RTV measurements. No associations were observed between DRV concentration and sex, age, ethnicity or weight. We did not see lower DRV concentrations in the subgroups with baseline CD4 <200 cells/µL or VL ≥100,000 copies/mL. Higher DRV concentrations were associated with both higher RTV and (in RAL arm) higher RAL concentration (p<0.001). No association was found between DRV concentration at week 4 and virological failure at or after week 32 in analyses of both arms together or separately for each arm. RAL concentration at week 4 was also not associated with VL failure.

**Conclusion**: DRV exposure was not affected by age, gender, weight or ethnicity but showed a positive association with RTV and RAL concentrations. There was no evidence of an association between DRV concentrations and virological failure at or after week 32.

# Sub article: P306 Evaluation of the drug-drug interaction (DDI) potential between elvitegravir/cobicistat/emtricitabine/tenofovir alafenamide and atorvastatin

**Published:** 2016-10-23

**Authors:** Joseph Custodio, Steve West, Devi Sengupta, Arian Zari, Rita Humeniuk, Kah Hiing Ling, Marshall Fordyce, Brian Kearney

**Affiliations:** 1 Clinical Pharmacology, Gilead Sciences, Foster City, CA, USA; 2 Biometrics, Gilead Sciences, Foster City, CA, USA; 3 Clinical Research, Gilead Sciences, Foster City, CA, USA; 4 Clinical Operations, Gilead Sciences, Foster City, CA, USA

## Abstract

**Introduction**: Elvitegravir/cobicistat/emtricitabine/tenofovir alafenamide (EVG/COBI/FTC/TAF (150/150/200/10 mg); E/C/F/TAF; Genvoya) is a single-tablet regimen approved for HIV treatment. Atorvastatin (AVA; Lipitor), a HMG-CoA reductase inhibitor, is a commonly prescribed medication for lipid lowering in HIV-infected individuals. AVA is metabolized by CYP3A and is a substrate of Pgp and OATP1B1/1B3. COBI, a pharmacokinetic (PK) enhancer in E/C/F/TAF, is an inhibitor of CYP3A, P-gp and OATP1B1/1B3. This study evaluated the DDI potential between E/C/F/TAF and AVA.

**Materials and methods**: This was a randomized, three-period and open-label study. Healthy subjects (n=16) received the following treatments in a fixed sequence: AVA 10 mg on day 1; E/C/F/TAF on days 4 to 13; E/C/F/TAF+AVA 10 mg on day 14. PK assessments were performed on the last day of each period (days 1, 13 and 14). Statistical comparisons of EVG, COBI, TAF, TFV and AVA exposures were made using geometric mean ratios (GMRs) and associated 90% CI bounds of 70 to 143% (EVG, COBI, TAF, TFV AUC, C_max_ and C_tau_; AVA AUC) and 50 to 200% (AVA C_max_), with E/C/F/TAF+AVA serving as the test (day 14) and E/C/F/TAF or AVA alone serving as the reference (day 13 or 1, respectively).

**Results**: All subjects completed the study and treatments were generally well tolerated. The majority of adverse events (AEs) were mild in severity and no grade 3 or 4 AEs were observed. Following co-administration of E/C/F/TAF+AVA, relative to AVA alone, the GMRs of the PK parameters of AVA were 2.3- to 2.9-fold higher (Table 1) and its active ortho- and parahydroxylated metabolites were undetectable, indicating a DDI attributed the COBI-mediated effect of E/C/F/TAF on AVA. In contrast, and as expected due to the limited liability of AVA as a perpetrator of DDI, following co-administration of E/C/F/TAF+AVA, relative to E/C/F/TAF alone, the 90% CIs about the GMRs of the PK parameters of EVG, COBI, TAF and TFV were within the prespecified bounds and consistent with historical data.

**Conclusions**: All treatments were generally well tolerated. An increase in AVA exposures was observed following co-administration with E/C/F/TAF, consistent with intestinal Pgp inhibition and the inhibition of the CYP3A-mediated metabolism of AVA by COBI. AVA did not result in changes in the PK of the components of E/C/F/TAF. These findings are aligned with the prescribing information of E/C/F/TAF regarding AVA, which recommends initiating AVA treatment with the lowest starting dose and titrate with clinical monitoring.

**Table 1:** Summary of AVA PK parameters and statistical comparisons

AVA PK Parameter	E/C/F/TAF + AVA (test) N = 16 mean (% CV)	AVA (reference) N = 16 mean (% CV)	GMR (90% CI) test/reference
AUC_inf_ (ng*h/mL)	38.4 (32)	14.6 (27)	260 (231–293)
AUC_last_ (ng*h/mL)	34.9 (33)	12.0 (30)	291 (263–322)
C_max_ (ng/mL)	3.0 (51)	1.3 (56)	232 (191–282)

# Sub article: P307 Steady-state pharmacokinetics (PK) of atazanavir/cobicistat and darunavir/cobicistat once daily over 72 hours in healthy volunteers: the importance of PK forgiveness in clinical practice

**Published:** 2016-10-23

**Authors:** Emilie Elliot, Alieu Amara, Nicole Pagani, Laura Else, Graham Moyle, Alexandra Schoolmeesters, Chris Higgs, Saye Khoo, Marta Boffito

**Affiliations:** 1 St Stephens AIDS Trust, Chelsea and Westminster Hospital, London, UK; 2 Liverpool Bioanalytical Facility, Royal Liverpool University Hospital, Liverpool, UK; 3 HIV/GUM Directorate, Chelsea and Westminster Hospital, London, UK; 4 Institute of Translational Medicine, Royal Liverpool University Hospital, Liverpool, UK

## Abstract

**Introduction**: Data on protease inhibitors (PI) persistence in plasma are limited to ritonavir (RTV)-boosting, and data with cobicistat-boosting are unavailable. The object of this study was to investigate the PK of darunavir (DRV)–cobicistat and atazanavir (ATV)–cobicistat once-daily dosing over 72 hours following drug intake cessation.

**Materials and methods**: Healthy volunteers received a fixed-dose combination of atazanavir 300 mg + cobicistat 150 mg once daily for 10 days, followed by a 10-day washout period and then a fixed-dose combination of darunavir 800 mg + cobicistat 150 mg once daily for 10 days. Full PK profiles were assessed for each phase for the 72 hours following day 10. PK parameters were determined over 24 hours in plasma and to the last measurable concentration in plasma and urine (24–72 hours post-dose) by non-compartmental methods.

**Results**: Sixteen subjects completed the study. Geometric mean (GM) terminal elimination half-life to 72 hours of darunavir was 6.35 hours, lower than the 0 to 24 hours half-life (10.41 hours). The terminal elimination half-life of atazanavir was 6.77 hours, lower than the 0 to 24 hours half-life (9.69 hours). These values did not remarkably differ from those measured with RTV [1]. Thirteen of 16 subjects had darunavir concentrations higher than the target of 550 ng/mL for protease-resistant HIV isolates (equivalent to 10 times the protein binding corrected minimum inhibitory concentration (IC50) for wild-type virus) [2] at 24 hours post-dose, and 5/16 subjects had concentrations higher than the target at 30 hours post-dose (GM of 1033 and 381 ng/mL). All subjects had atazanavir concentrations above the suggested minimum effective concentration of 150 ng/mL (equivalent to 10 times the protein binding corrected IC50 for wild-type virus) [2] 24 hours post-dose and 14/16 subjects had concentrations higher than the target at 30 hours post-dose (GM of 759 and 407 ng/mL). At 48 hours post-dose, no subject had concentrations above targets with darunavir-cobicistat whilst 3/16 subjects did for atazanavir-cobicistat. Cobicistat half-life to 72 hours was 3.62 hours with darunavir and 4.21 hours with atazanavir (both were shorter than RTV's) [1]. GM urine C24 darunavir and atazanavir concentrations were 11,878 ng/mL and 24,857 ng/mL respectively. Urine concentrations decay mirrored plasma concentration decay for both drugs.

**Conclusions**: This study investigated the PK forgiveness of two cobicistat-boosted protease inhibitors in plasma. Different concentration decay patterns and relationships with cut-offs used to define therapeutic concentrations were seen for DRV and ATV, which may be partially explained by cobicistat half-life (shorter with DRV than ATV). For the first time, we also measured drug PK forgiveness in urine, which can be an easier marker of adherence. Further clinical data are warranted to inform on whether drug doses can be delayed or missed.

## References

[1] Boffito M, Jackson A, Amara A, Back D, Khoo S, Higgs C (2011). Pharmacokinetics of once-daily darunavir-ritonavir and atazanavir-ritonavir over 72 hours following drug cessation. Antimicrob Agents Chemother.

[2] La Porte CJL, Back DJ, Blaschke T, Boucher CAB, Fletcher CV, Flexner C (2006). Updated guideline to perform therapeutic drug monitoring for antiretroviral agents. Rev Antiviral Ther.

# Sub article: P308 Association of SLCO1B1 521T>C (rs4149056) with darunavir/ritonavir (DRV/r) plasma concentrations in HIV-infected individuals enrolled in the NEAT001/ANRS143 study

**Published:** 2016-10-23

**Authors:** Rohan Gurjar, Marta Boffito, Antonio D'Avolio, Christine Schwimmer, Yazdan Yazdanpanah, Manuela Doroana, Giovanni Di Perri, Stefano Bonora, Anton Pozniak, Laura Richert, Francois Raffi, Andrew Owen

**Affiliations:** 1 Molecular and Clinical Pharmacology, University of Liverpool, Liverpool, UK; 2 Clinical Pharmacology, Imperial College London, London, UK; 3 Unit of Infectious Diseases, University of Torino, Turin, Italy; 4 Inserm U1219 Epidemiologie-Biostatistique, University of Bordeaux, Bordeaux, France; 5 Infectious Diseases Department, Bichat Hospital, Paris, France; 6 Servico de Doencas Infecciosas, Hospital de Santa Maria, Lisbon, Portugal; 7 Department of Medical Sciences, University of Torino, Turin, Italy; 8 Infectious Diseases Department, Chelsea and Westminster NHS Foundation Trust, London, UK; 9 Infectious and Tropical Diseases Department, Nantes University Hospital, Nantes, France

## Abstract

**Introduction**: Darunavir (DRV) is metabolized by cytochrome P450 3A4 (CYP3A4) and CYP3A5 and is a substrate for the hepatic influx transporter, OATP1B1 (encoded by SLCO1B1). The pregnane X receptor (PXR; NR1I2) and constitutive androstane receptor (CAR; NR1I3) play important roles in transcriptional regulation of enzymes and transporters. The effect of polymorphisms within these genes on the pharmacokinetics of DRV was investigated in participants from the NEAT 001/ANRS 143 study.

**Methods**: NEAT 001/ANRS 143 was a randomized study that demonstrated non-inferiority of first-line ART with DRV/ritonavir (DRV/r; 800/100 mg once daily) plus raltegravir (RAL; 400 mg twice daily) compared with DRV/r plus tenofovir/emtricitabine (TDF/FTC; 245/200 mg once daily). Blood samples were collected at week 4 post-therapy initiation at any time >5 hours post-dose. DNA was extracted from whole blood and genotyping for CYP3A4 (rs35599367), CYP3A5 (rs776746), SLCO1B1 (rs4149056; 521T>C), CAR (rs2307424) and PXR (rs2472677) polymorphisms was conducted. Plasma drug concentrations were log transformed and genetic associations were assessed using linear regression. Analysis was conducted in the entire cohort (pooled), as well as independently in each arm.

**Results**: A total of 1278 plasma concentrations were available from 653 participants. In week 4 pooled analysis, SLCO1B1 rs4149056 was associated with DRV plasma concentrations (p=0.025). SLCO1B1 rs4149056 was significantly associated with DRV plasma concentrations in the TDF/FTC arm (p=0.036), but not the RAL arm (p=0.38). In the TDF/FTC arm, plasma DRV concentrations were 2936±5256 ng/mL, 3121±2160 ng/mL and 2520±1296 ng/mL, in TT, TC and CC genotype groups, respectively. NR1I3 rs2307424 was significantly associated with ritonavir plasma concentrations in TDF/FTC arm (p=0.001) but not the RAL arm (p=0.57). Plasma concentrations for ritonavir in the TDF/FTC arm at week 4 were 207±256 ng/mL, 192±333 ng/mL and 111±117 ng/mL for GG, AG and AA, respectively. No other associations were observed.

**Conclusions**: Lower DRV plasma concentrations were observed in C homozygotes for SLCO1B1 521T>C in patients receiving a TDF/FTC backbone but not those receiving RAL. This association is different to that previously reported for lopinavir and statins, where concentrations were higher in C homozygotes. This may indicate an indirect effect of this polymorphism on DRV concentrations (e.g. mediated by an interacting drug) but confirmatory studies are required and the underlying mechanism needs to be elucidated.

# Sub article: P309 When food can make the difference: the case of elvitegravir-based coformulation

**Published:** 2016-10-23

**Authors:** Cristina Gervsasoni, Sara Baldelli, Davide Minisci, Paola Meraviglia, Emilio Clementi, Massimo Galli, Dario Cattaneo

**Affiliations:** 1 Infectious Diseases, Luigi Sacco University Hospital, Milano, Italy; 2 Unit of Clinical Pharmacology, Luigi Sacco University Hospital, Milano, Italy

## Abstract

**Introduction**: In the product monograph of Stribild, it is recommended that the formulation should be administered under fed conditions to optimize drugs exposure. Here we assessed to what extent this advice is applied in the real-life scenario by measuring drugs plasma trough concentrations in HIV-infected patients given Stribild alone or as part of antiretroviral therapy as per their daily routine practice.

**Materials and methods**: Consecutive HIV-infected patients treated with Stribild for at least one month, with no clinical evidence of gastrointestinal impairment and not given drugs known to affect elvitegravir or tenofovir pharmacokinetics, were considered. Drug concentrations were assessed by a validated LC-MS/MS method (lower limit of quantification (LOQ): 25, 10, 5 and 100 ng/mL for elvitegravir, tenofovir, cobicistat and darunavir, respectively).

**Results**: Thirty-six percent of our patients (n=65) took Stribild in the evening with food, and the remaining were distributed as follows: 23% in the morning with breakfast, 9% middle in the morning, 17% at lunchtime and 15% late in the evening. Nine out of the 65 patients had elvitegravir concentrations below the LOQ of the method, whereas in the remaining the levels were largely distributed (Figure 1). All patients with suboptimal elvitegravir exposure took Stribild under fasting conditions. Wide inter-individual variability in the tenofovir and cobicistat levels was also observed, with 13 out of the 65 patients having cobicistat concentrations <LOQ. Of these, patients given Stribild with darunavir had drug concentration significantly lower compared with values measured in patients with quantifiable cobicistat levels (402±547 vs. 3215±2435; p<0.001).

**Conclusions**: In a real-life context a significant proportion of patients took Stribild in fasting conditions. This resulted in a wide inter-individual variability of elvitegravir and tenofovir plasma trough concentrations. It is likely that suboptimal tablet disintegration and poor drug absorption may have taken place in these patients. Conversely, the intake of Stribild with food increases the resident time of the drug in the stomach, the disintegration of the pharmaceutical formulation, the increased dissolution of the components and their higher systemic absorption. Food-related altered absorption of cobicistat may become particularly relevant when darunavir is co-administered in order to reinforce the antiretroviral therapy, because the cobicistat-related boosting effect is lost, eventually resulting in sub-therapeutic darunavir concentrations. To avoid the risk for patients to experience suboptimal drug exposure, it is important that physicians more convincingly advise their patients to take Stribild in fed conditions.

**Figure 1:** Distribution of elvitegravir (right panel), tenofovir (middle panel) and cobicistat (left panel) trough concentrations in 65 HIV-infected patients given Stribild as maintenance antiretroviral therapy.

# Sub article: P310 Impact of food on the bioavailability of darunavir, cobicistat, emtricitabine and tenofovir alafenamide (DCFTAF), the first protease inhibitor-based complete HIV-1 regimen

**Published:** 2016-10-23

**Authors:** Herta Crauwels, Bryan Baugh, Erika Van Landuyt, Simon Vanveggel, Anja Hijzen, Magda Opsomer

**Affiliations:** 1 Janssen Infectious Diseases BVBA, Beerse, Belgium; 2 Janssen Research & Development LLC, Raritan, NJ, USA

## Abstract

**Introduction**: DCFTAF is the first single-tablet, once-daily protease inhibitor-based complete HIV-1 regimen containing darunavir (DRV, D 800 mg), cobicistat (COBI, C 150 mg), emtricitabine (FTC, F 200 mg) and the novel prodrug of tenofovir, tenofovir alafenamide (TAF, 10 mg). TAF has an improved renal and bone safety profile compared with tenofovir disproxyl fumarate. The efficacy and safety of DCFTAF is under investigation in two international, fully randomized phase 3 studies, AMBER (NCT02431247) and EMERALD (NCT02269917). This study evaluated the impact of food on the pharmacokinetics of the DCFTAF components.

**Methods**: This was a phase 1, open-label, randomized, two-period, single-centre, crossover study in 24 HIV-negative, healthy volunteers (NCT02475135). In two treatment sessions, participants received a single oral dose of DCFTAF under fasted conditions or 30 minutes after a standardized high-fat breakfast, with a ≥7-day washout period in between. Pharmacokinetic profiles were determined over 72 hours for DRV and COBI, 48 hours for FTC and 12 hours for TAF. Plasma concentrations of DRV, COBI, FTC and TAF were determined using validated LC-MS/MS assays. Pharmacokinetic parameters were determined using non-compartmental analysis (WinNonlin) and evaluated using least square (LS) means ratios and 90% CIs. Safety and tolerability were assessed throughout the study.

**Results**: When administered as DCFTAF, DRV exposure was 30 to 45% lower and COBI exposure was 16 to 30% lower, in fasted (test) compared with fed conditions (reference) (Table 1). For FTC, C_max_ was 26% higher in fasted compared with fed conditions, while AUC_last_ was comparable under both conditions (Table 1). For TAF, the C_max_ was 82% higher, while AUC_inf_ was 20% lower, in fasted than in fed conditions. The TAF AUC_last_ was comparable under both conditions (90% CI of the LS mean ratio was within the 80 to 125% boundaries of no effect) (Table 1). Administration of DCFTAF was generally well tolerated under fed and fasted conditions. No new safety issues, grade 3/4 or serious adverse events or deaths occurred. There were no discontinuations due to adverse events.

**Conclusions**: When administered as the DCFTAF tablet, DRV exposure was decreased in fasted conditions versus fed conditions, similar to other (co)formulations of DRV. Differences in the exposures to COBI, FTC and TAF in fasted conditions versus fed conditions are not considered to be clinically relevant. Consistent with other DRV formulations, it is recommended the DCFTAF tablet be taken with food, which is also the recommendation in the ongoing phase 3 AMBER and EMERALD trials in HIV-1-infected adults.

SD, standard deviation; LS, least square; CI,confidence interval; C_max_, maximum plasma concentration; t_max_, time to Cmax; AUC_last_, area under the plasma concentration-time curve (AUC, calculated by linear-linear trapezoidal summation) from time of administration up to the last timepoint with a measurable concentration post-dose; AUC_inf_, AUC from time of administration to infinity; t1/2term, terminal elimination half-life; ND, not determined.

**Table 1:** DRV, COBI, FTC and TAF pharmacokinetic parameters and statistical analysis

Parameter, mean (SD)^a^	DCFTAF fasted (test) n=24	DCFTAF fed (high-fat breakfast) (reference) n=24	LS means ratio,%	90% CI,%
DRV				
C_max_, ng/mL^b^	4089 (1846)	6629 (1543)	54.99	46.73–64.71
T_max_, hours^b^	3.00 (1.00–8.02)	5.00 (1.50–8.00)	ND	ND
AUC_last_, ng.h/mL^b^	67,504 (35,642)	93,541 (39,730)	65.65	56.76–75.92
AUC_inf_, ng.h/mL^c^	72,147 (36,009)	94,686 (40,882)	70.25	59.49–82.95
t1/2term, hours^c^	7.0 (2.3)	7.8 (3.5)	ND	ND
COBI				
C_max_, ng/mL^b^	704 (368)	711 (164)	76.96	55.70–106.33
tmax, hours^b^	3.00 (1.00–6.00)	5.00 (2.00–6.10)	ND	ND
AUC_last_, ng.h/mL^b^	5771 (3206)	6168 (2260)	70.90	51.13–98.30
AUC_inf_, ng.h/mL^d^	6136 (3064)	6258 (2268)	84.39	68.52–103.95
t1/2term, hours^d^	4.1 (0.9)	3.9 (0.6)	ND	ND
FTC				
C_max_, ng/mL	2247 (573)	1785 (486)	125.99	112.85–140.65
t_max_, hours	1.00 (0.50–2.00)	2.00 (0.75–5.00)	ND	ND
AUC_last_, ng.h/mL	11,593 (2573)	11,499 (2055)	100.12	96.29–104.10
AUC_inf_, ng.h/mL^e^	12,286 (2729)	10,029 (1079)^g^	ND	ND
t1/2term, hours^e^	10.8 (1.2)	10.7 (1.2)^g^	ND	ND
TAF				
C_max_, ng/mL	180 (90.6)	107 (65.2)	182.29	140.50–236.50
t_max_, hours	0.50 (0.25–0.75)	0.88 (0.25–5.00)	ND	ND
AUC_last_, ng.h/mL	106 (44.7)	117 (51.5)	89.54	81.20–98.72
AUC_inf_, ng.h/mL^f^	109 (47.7)	125 (57.3)	80.38	73.04–88.45
t1/2term, hours^f^	0.3 (0.2)	0.5 (0.1)	ND	ND

a Except t_max_=median (range)

b n=23 for test and n=24 for reference

c n=20 for test and reference

d n=22 for test and n=24 for reference

e n=16 for test and n=7 for reference

f n=21 for test and n=16 for reference

g Accurate determination not possible for more than 50% of participants; interpret with caution.

# Sub article: P311 Prevalence of drug-drug interactions (DDI) and its impact on durability among patients receiving elvitegravir/cobicistat/emtricitabine/tenofovir (EVG/C/F/T) and concomitant medication

**Published:** 2016-10-23

**Authors:** Cristina Gomez-Ayerbe, Santos Del Campo, Maria Jesus Vivancos, Gabriel Gaspar, Ana Moreno, Andres Martin, Alberto Romero, Patricia Jimenez, Miguel Cervero, Miguel Angel Rodriguez-Sagrado, Regino Serrano, Alfonso Muriel, María Jesús Peréz Elías

**Affiliations:** 1 Infectious Diseases, Instituto Ramon y Cajal de Investigación Sanitaria, Hospital Ramon y Cajal, Madrid, Spain; 2 Internal Medicine, Hospital de Getafe, Getafe, Spain; 3 Internal Medicine, Hospital Universitario Puerta del Mar, Cádiz, Spain; 4 Internal Medicine, Hospital Universitario de Puerto Real, Cádiz, Spain; 5 Internal Medicine, Hospital Severo Ochoa, Leganés, Spain; 6 Pharmacy Department, Instituto Ramon y Cajal de Investigación Sanitaria, Hospital Ramon y Cajal, Madrid, Spain; 7 Internal Medicine, Hospital del Henares, Coslada, Spain; 8 Statistics Department, Instituto Ramon y Cajal de Investigación Sanitaria, Hospital Ramon y Cajal, Madrid, Spain

## Abstract

**Introduction**: Cobicistat, a component of single-treatment regimen EVG/C/F/T, is a potent cytochrome P450 inhibitor [1], so many DDI are expected. From the real practice perspective, we evaluated the clinical impact of DDI associated with the use of EVG/C/F/T and concomitant medication (CM).

**Methods**: From July 2014 to January 2016, we retrospectively reviewed all patients starting a new EVG/C/F/T regimen, both in naïve and switching scenarios, in six hospitals of Spain. CM was recorded since EVG/C/F/T was started, and categorized, according to the Liverpool HIV Drug Interactions (LHDI) tool (no interactions expected, potential interactions/use with caution and formally contraindicated). The highest degree of DDI found was applied. Time and reasons to change EVG/C/F/T were compared according to CM status (no DDI, if patient has no CM or no interactions were expected, and DDI present, if CM has been identified by LHDI tool with potential interactions or contraindicated) [2].

**Results**: Overall 243 patients, followed for a median time of 13.7 years before starting EVG/C/F/T, were included, 75% men, with a median age of 47 years, route of HIV transmission (IDU 35%, MSM 34%), AIDS stage 28%, baseline CD4 count 521 and HIV RNA fully suppressed in 73%. Among 198 (82%) pre-treated patients, main reasons for starting EVG/C/F/T were simplification 57% and toxicity 18%. Thirty-seven percent of patients did not received CM, while 152 (63%) received a median of 2 (2–4) CM that were classified as no DDI 26%, potential DDI 73% or contraindicated 2% (domperidone and ivadravina). After a median exposure of 278 days to EVG/C/F/T, 13% of patients changed its therapy due to toxicity 6.2%, DDI 2.1%, failure 0.8%, simplification 0.8% and other reasons 3.3%. No differences were observed in median time to change EVG/C/F/T according to DDI status, neither in the univariate log rank test p (0.69) nor in the Cox regression analysis p (0.64).

**Conclusions**: In clinical practice, despite a large number of drug-drug interactions are predicted for EVG/C/F/T and concomitant medication, these did not influence a shorter durability.

## References

[1] http://www.hiv-druginteractions.org.

[2] Xu L, Liu H, Murray BP, Callebaut C, Lee MS, Hong A (2010). Cobicistat (GS-9350): a potent and selective inhibitor of human CYP3A as a novel pharmacoenhancer. ACS Med Chem Lett.

# Sub article: P312 Relationships between dolutegravir plasma-trough concentrations, UGT1A1 genetic polymorphisms and side-effects of central nervous system in Japanese HIV-1-infected patients

**Published:** 2016-10-23

**Authors:** Hiroki Yagura, Dai Watanabe, Misa Ashida, Takao Nakauchi, Kosuke Tomishima, Hiroaki Togami, Atushi Hirano, Rumi Sako, Toshiyuki Doi, Munehiro Yoshino, Masaaki Takahashi, Kunio Yamazaki, Tomoko Uehira, Takuma Shirasaka

**Affiliations:** 1 Department of Pharmacy, Osaka National Hospital, Osaka, Japan; 2 AIDS Medical Center, Osaka National Hospital, Osaka, Japan; 3 Department of Pharmacy, Nagoya Medical Center, Osaka, Japan; 4 Department of Pharmacy, Osaka-Minami Medical Center, Osaka, Japan; 5 Department of Pharmacy, Suzuka Hospital, Mie, Japan

## Abstract

**Introduction**: Dolutegravir (DTG) is a second-generation integrase inhibitor used for the treatment of HIV-1-infected patients. DTG has shown anti-HIV effects non-inferior to those of other drugs in phase 3 trials and can be conveniently taken once daily. The characteristic side effects of DTG include central nervous system side-effects (CNSSEs) leading to drug discontinuation in cases. Furthermore, DTG is primarily metabolized by UGT1A1, and there is a weak correlation between DTG plasma-trough concentrations and UGT1A1 genetic polymorphisms. The principal aim of the study was to explore DTG plasma-trough concentrations association with CNSSEs. Moreover, we considered whether UGT1A1 genetic polymorphisms could predict of DTG CNSSEs.

**Materials and methods**: We included 101 Japanese HIV-1-infected patients given DTG at Osaka National Hospital from June 2014 to March 2016. Their DTG trough levels were measured by liquid chromatography-mass spectrometry. UGT1A1 was genotyped using the sequence method. We compared the frequency of CNSSEs among three groups: A (with homozygous mutations in UGT1A1*6/*28 or compound heterozygous mutations in *6/*28); B (patients with heterozygous mutations in *6/*28); and C (wild-type).

**Results**: Side-effects developed in 37 of 101 patients (37%), and of these, CNSSEs were evident in 21 patients (21%): headache in eight (38%); insomnia in six (29%); irritability in three (14%); light-headedness in two (9%); depression in one (1%); and dizziness in one (1%). The median DTG plasma-trough concentration was significantly higher in patients with CNSSEs (1.34 µg/mL) than in those without CNSSEs (1.06 µg/mL) (p<0.05).The frequencies of CNSSEs in the three groups were: A, 23%; B, 25% and C, 18%. No significant difference in the frequency of CNSSEs was evident in terms of UGT1A1 genetic polymorphisms.

**Conclusion**: Although UGT1A1 genetic polymorphisms are not predictive of DTG CNSSEs, the data suggest that a relationship may exist between DTG plasma-trough concentrations and CNSSEs.

# Sub article: P313 S-protein thiol-omics to assess the redox-modulation effects of antiretroviral drugs

**Published:** 2016-10-23

**Authors:** Maria Correia, Clara Dias, Umbelina Caixas, Nádia Grilo, Ana Lemos, Diva Trigo, Patricia Pacheco, Emilia Monteiro, Karina Soto, Lucília Diogo, Sofia Pereira

**Affiliations:** 1 Translational Pharmacology, CEDOC-NOVA Medical School/Faculdade de Ciências Médicas (NMS/FCM), Lisboa, Portugal; 2 Translational Pharmacology, Centro Hospitalar de Lisboa Central, CEDOC-NOVA Medical School/Faculdade de Ciências Médicas (NMS/FCM), Lisboa, Portugal; 3 Infectious Diseases, Hospital Fernando Fonseca, Lisboa, Portugal; 4 Translational Pharmacology/Nephrology, Hospital Fernando Fonseca, CEDOC-NOVA Medical School/Faculdade de Ciências Médicas (NMS/FCM), Lisboa, Portugal

## Abstract

**Introduction**: There is expanding evidence that redox-imbalance plays a role in viral, inflammatory and immunological response of HIV infection [1]. Albumin circulates as S-thiolated form (RSSP) in near of 25%, and S-thiolation by disulfide bond with low molecular weight thiols, such as glutathione (GSH) generating glutathionylated proteins (GSSP), cysteine (cysSH; CysSSP), homocysteine (HCysSH; HCysSSP) and cysteinylglycine (CysGlySH; CysGlySSP) is a common reversible oxidative modification. This process protects protein thiols from irreversible oxidation, is a relevant redox-buffer in blood and has a regulatory function [2]. The RSSP-profile might represent a pharmacodynamics biomarker for assessing the redox-modulating effects of drugs. The antiretrovirals, as efavirenz (EFV) and nevirapine (NVP), were implicated with protein adducts formation and oxidative stress induction [3]. This work is aimed to investigate the RSSP-profiling in a cohort of HIV-infected patients and additionally the redox-modulating response to EFV and NVP.

**Methods**: The study protocol received prior approval from hospitals ethics committee. Patients gave their written informed consent. A cross-sectional analysis was performed. Patients were stratified according to cART use: naïve, on NVP-cART and EFV-cART. Anthropometric and clinical data (age, CD4 cell count, viral load, kidney and liver function parameters, hepatitis B and C co-infection) were recorded for each patient. Exclusion criteria included kidney and hepatic dysfunction. Patients with detectable viral load in cART groups were also excluded. Thiols were quantified by an HPLC-FD method and the RSSP-profiles were obtained.

**Results**: A total of 135 patients were included (70% male, 44±11 years old; CD4 cell count 606±22 cells/µL). Patients’ characteristics and data obtained from thiol-omic analyses are summarized in Table 1. Among naïve patients, there was no association between viral load and any type of RSSP. Conversely, CD4 cell count was associated with CysSSP (r=0.5390, p=0.014), GSSP (r=0.4930, p=0.044) and CysGlySSP (r=0.4508, p=0.046). Multivariable analysis of the entire cohort showed that GSSP levels were associated with age (B: −0.04; 95% CI −0.06–−0.02; p=0.001) and cART (B: 1.7; 95% CI 1.1–2.2; p<0.001). CysSSP levels were only influenced by age (B: 1.3; 95% CI 0.8–1.9; p<0.001). When compared with naïve patients, EFV-cART increases GSSP and decreased CysSSP, whereas NVP-cART influenced GSSP and CysSSP in opposite way (Table 1).

**Conclusions**: Immunological status of patients is related to their RSSP-profile, which is influenced by age and cART. EFV-cART and NVP-cART showed an inverted GSSP and CysSSP profile. This data support the use of RSSP-profile for the assessment of redox-modulating effects of antiretroviral drugs.

**Financial support**: EXPL/DTP-FTO/1792/2013; PD/BD/105892/2014(CGD); SFRH/BD/86791/2012(NMG).

**Table 1:** Patient characteristics and data attended from thiol-omics analysis

Variable	Healthy volunteers^a^	Naïve	NVP	EFV	p
N	63	22	30	83	–
Age (years)	NA	41.6±9.9	47.9±10.1	43.30±11.1	ns
CD4 cell count (cell/µL)	NA	478.6±192.2	600.2±300.5	641.7±239.0	0.025^b^
Viral load (copies/mL)	–	43,096±55,670	BQL	BQL	–
Total Hcys (µM)	11.3±4.4	10.5±3.7	11.4±3.6	12.6±4.9	ns
HcysSSP (µM)	9.3±3.6	8.2±3.4	9.9±3.4	10.5±4.5	0.039^c^
Total Cys (µM)	237.1±36.6	215.7±38.7	223.9±35.0	193.3±38.4	<0.001^b^
CysSSP (µM)	156.8±28.4	166.9±29.2	180.6±31.6	145.9±36.3	<0.001^b^
Total GSH (µM)	5.8±1.8	2.4±1.2	1.7±0.5	3.9±1.9	<0.001^c^
GSSP (µM)	2.3±1.2	1.5±0.8	0.9±0.4	2.7±1.5	<0.001^c^
Total CysGly (µM)	19.7±3.9	24.6±5.6	27.4±5.9	32.9±6.3	<0.001^b^
CysGlySSP (µM)	11.9±3.0	17.8±4.9	21.4±5.0	24.7±5.0	<0.001^b^

Data are presented as mean±SD. BQL, below quantification limit; NA, not available; ns, not significant.

a Data from Rossi et al., 2009 [4]

b one-way ANOVA

c Kruskal–Wallis test.

## References

[1] Aquaro S, Scopelliti F, Pollicita M, Perno CF (2008). Oxidative stress and HIV infection: target pathways for novel therapies?. Future HIV Ther.

[2] Dalle-Donne I, Milzani A, Gagliano N, Colombo R, Giustarini D, Rossi R (2008). Molecular mechanisms and potential clinical significance of S-glutathionylation. Antioxid Redox Signal.

[3] Caixas U, Antunes AM, Marinho AT, Godinho AL, Grilo NM, Marques MM (2012). Evidence for nevirapine bioactivation in man: searching for the first step in the mechanism of nevirapine toxicity. Toxicology.

[4] Rossi R, Giustarini D, Milzani A, Dalle-Donne I (2009). Cysteinylation and homocysteinylation of plasma protein thiols during ageing of healthy human beings. J Cell Mol Med.

# Sub article: P314 Utilizing phase 3 clinical trial data to assess adverse event (AE) frequency of a potentially interacting medication (PIM) amlodipine with elvitegravir/cobicistat (EVG/COBI)

**Published:** 2016-10-23

**Authors:** Daniel Podzamczer, Karen Tashima, Eric Daar, Joseph McGowan, Thomas Campbell, Jihad Slim, Melanie Thompson, Susan Guo, Peter Borg, Richard Haubrich, Moupali Das, Ian McNicholl, Scott McCallister

**Affiliations:** 1 Servicio de Enfermedades Infecciousas, Hospital Universitari de Bellvitge, Barcelona, Spain; 2 Infectious Diseases, Miriam Hospital, Providence, RI, USA; 3 Internal Medicine, Los Angeles Biomedical Research Institute at Harbor-UCLA Medical Center, Torrance, CA, USA; 4 Internal Medicine, North Shore University Hospital, Manhasset, NY, USA; 5 Internal Medicine, University of Colorado, Denver, CO, USA; 6 Internal Medicine, Saint Michael's Medical Center, Newark, NJ, USA; 7 Internal Medicine, AIDS Research Consortium of Atlanta, Atlanta, GA, USA; 8 Biometrics, Gilead Sciences, Foster City, CA, USA; 9 Medical Affairs, Gilead Sciences, Stockley Park, UK; 10 Medical Affairs, Gilead Sciences, Foster City, CA, USA; 11 Clinical Research, Gilead Sciences, Foster City, CA, USA

## Abstract

**Introduction**: EVG/COBI has shown high rates of efficacy and when combined in the single-tablet regimen (STR) with emtricitabine/tenofovir-alafenamide (F/TAF) improved bone and renal safety in treatment-naïve and treatment-experienced participants compared with F/tenofovir disoproxil fumarate (F/TDF). We evaluated the clinical consequences of use of the PIM amlodipine, a medication which has a caution and recommendation for clinical monitoring when administered with EVG/COBI/FTC with either TDF or TAF, in nine large phase 3 clinical trials.

**Materials and methods**: We retrospectively pooled data from five treatment-naïve studies (GS-US-292-0104, GS-US-292-0111, GS-US-236-0102, GS-US-236-0103, GS-US-236-0128) and four treatment-experienced studies (GS-US-292-0109, GS-US-292-0112, GS-US-236-0115, GS-US-236-0121) to assess AEs associated with concomitant use of amlodipine. All participants received EVG/COBI/emtricitabine combined in an STR with either TDF or TAF. Drug-specific AEs were obtained from Micromedex and Lexi-Comp. We evaluated the following: (1) AEs occurring in >10% of participants, (2) AEs leading to premature discontinuation and (3) drug-specific grade 2–4 AEs. Statistical comparisons between users and non-users of the PIM were conducted using two-sided Fisher exact tests.

**Results**: Of the 4667 participants, there were 153 who received amlodipine (mean age 50 years, 75% male and 46% Caucasian). See Table 1.

Although there was no difference in all-grade adverse events between amlodipine users and non-users, amlodipine users had higher rates of drug-specific AEs: (1) peripheral edema (4.6% with and 0.4% without amlodipine, p<0.001) and (2) nervous system disorders (2.6% vs. 0.8% with and without amlodipine, p=0.035). Although participants on amlodipine had a higher overall STR discontinuation rate than non-users, only one discontinuation event could be considered due to an amlodipine-specific AE (local swelling).

**Conclusions**: Overall AEs and discontinuations due to drug-specific AEs were similar in participants who did or did not use concomitant amlodipine. Amlodipine-specific AEs were higher for participants using amlodipine, but only one participant discontinued EVG/COBI/F/TDF or EVG/COBI/F/TAF due to an amlodipine AE. Because EVG/COBI/F/TDF or EVG/COBI/F/TAF can increase the level of amlodipine when co-administered, clinical monitoring is recommended.

**Table 1:** Frequency of adverse events between amlodipine users and non-users

	All-grade AEs occurring in >10% of participants	Drug-specific grade 2–4 treatment-emergent AEs	Treatment-emergent AEs leading to premature DC
Amlodipine (n=153)	49%	14% (22)	5.2% (8)
No amlodipine (n=4514)	47%	5.3% (237)	2.3% (105)
p	NS	<0.001	0.031

# Sub article: P315 Real-world antiretroviral plasma levels in HIV-positive patients treated with sofosbuvir-containing DAA for hepatitis C infection

**Published:** 2016-10-23

**Authors:** Massimo Tempestilli, Gabriele Fabbri, Laura Timelli, Mauro Zaccarelli, Rita Bellagamba, Stefania Cicalini, Federico Lupi, Anna Gallo, Raffaella Libertone, Simone Fazio, Andrea Antinori, Adriana Ammassari

**Affiliations:** 1 Clinical Immunology and Pharmacology Laboratory, National Institute for Infectious Diseases, Rome, Italy; 2 Clinical Department, National Institute for Infectious Diseases, Rome, Italy

## Abstract

**Introduction**: Drug–drug interactions (DDI) between hepatitis C direct-acting antiviral agents (DAA) and HIV ARVs are frequent. To date, most information has been obtained from phase 1 DDI studies in healthy volunteers and drug combinations permitted in phase 2 and 3 HIV/HCV co-infection trials [1,2]. Aim of this study was to investigate ARV plasma trough levels before and during sofosbuvir (SOF)-based treatment in HCV/HIV co-infected patients treated in the real-world setting.

**Material and methods**: This study is a monocentre, prospective, open-label, observational cohort study. HIV/HCV co-infected persons undergoing HCV treatment with standard dose of DAA and antiretrovirals are enrolled. Patients also need to receive the same ARVs for at least 2 weeks before starting DAA treatment and to have HIV RNA <40 copies/mL at baseline. Antiretroviral regimen is prescribed by clinical care providers based on antiretroviral treatment history, previous HIV genotypic resistance testing, tolerability and recommendations for management of HCV/HIV co-infected persons in need of HCV treatment. The C_trough_ of ARVs is measured using a validated high-performance liquid chromatography (HPLC). Blood samples are collected before and after 2 months of DAA treatment. For the purpose of this analysis, estimated change of C_trough_ from before to during DAA was obtained by using a random effect linear regression. A minimum of seven C_trough_ coupled values for each ARV were required for final statistical analysis.

**Results**: To date, 66 out of 91 enrolled patients were analyzed: 27 received SOF+LDV (40.9%), 16 SOF+dactatasvir (24.4%), 6 SOF+simeprevir (9.1%) and 17 SOF+ribavirin (25.8%). Concurrent ARVs included atazanavir (n=8), darunavir (n=19), raltegravir (n=19), efavirenz (n=7), etravirine (n=8) and rilpivirine (n=8). No statistically significant difference in C_trough_ of considered ARVs was found in samples obtained before and during SOF-based treatment (Figure 1). In 2/66 patients (3.0%), at least one HIV RNA detectable >40 copies/mL during SOF-based treatment was observed. Consequences of loss of virological suppression, such as resistance development or treatment change, are still under observation.

**Conclusions**: In this large HIV/HCV co-infected patient population observed in the real-world setting, no significant modifications in ARV concentrations during SOF-based DAA treatment were observed for the most commonly used antiretrovirals. Nonetheless, loss of virological suppression does occur during DAA treatment and thus monitoring of plasma drug levels and viral load is advisable.

**Figure 1:** Median C_trough_ plasma concentration of atazanavir, darunavir, efavirenz, rilpivirine, etravirine and raltegravir before and during SOF-based treatment.

## References

[1] Molina JM, Orkin C, Iser DM, Zamora FX, Nelson M, Stephan C (2015). Sofosbuvir plus ribavirin for treatment of hepatitis C virus in patients co-infected with HIV (PHOTON-2): a multicentre, open-label, non-randomised, phase 3 study. Lancet.

[2] Saeed S, Strumpf EC, Walmsley SL, Rollet-Kurhajec K, Pick N, Martel-Laferrière V (2016). How generalizable are the results from trials of direct antiviral agents to people coinfected with HIV/HCV in the real world?. Clin Infect Dis.

# Sub article: P316 Detection and analysis of antiretroviral medication errors by a clinical pharmacist in hospitalized HIV patients

**Published:** 2016-10-23

**Authors:** Siria Pablos Bravo, Carmen García Muñoz, Federico Pulido, Andrea Lázaro Cebas, Jose Miguel Ferrari Piquero

**Affiliations:** 1 Pharmacy Department, Hospital Universitario 12 de Octubre, Madrid, Spain; 2 HIV Unit, Internal Medicine Department, Hospital Universitario 12 de Octubre, Madrid, Spain

## Abstract

**Introduction**: Regarding the published data, the overall medication error rate in HIV patients admitted to a hospital varies between 5.8% and 86% [1], depending on the methodology and study duration. Admission of an HIV-infected patient by a physician not specialized in infectious diseases could be a risk factor for drug-related problems. Most of the described errors are prescribing errors [2],^3^, highlighting the need for a detailed and accurate medication reconciliation on admission.

**Materials and methods**: Descriptive observational study. HIV-infected patients with any ART admitted to a hospital ward were included. The study lasted 5 months (March–July 2015).The primary outcome was the ART error rate. Secondary outcomes included the following: type of ART error; omission of treatment, wrong schedule, wrong dose, wrong drug, pharmacologic interaction (classified as potential interaction or forbidden co-administration according to the University of Liverpool classification of interactions); error rate in each type of ward (medical or surgical); number of times error reach to patient; time until correction of medication errors. A clinical pharmacist reviewed prescriptions of all hospitalized HIV patients with ART on a daily basis. Medication reconciliation was made comparing outpatient medication records with the treatment prescribed to the patient at admission. The pharmaceutical intervention was carried out through a text message associated with the electronic prescription and a phone call to the physician in charge of the patient. Subsequently, the degree of acceptance of interventions was evaluated.

**Results**: In total, 105 HIV patients were admitted to our hospital during follow-up period, with a total of 124 admissions. Patients had a mean (SD) age of 49 years (±8.48) and 73.4% were male. A total of 32 patients (30.5%) had at least one error. We detected a total of 41 errors (Table 1).

A total of 13 forbidden co-administration and 505 potential interactions were detected. However, pharmacist intervention was necessary only in two patients with contraindicated combinations (protease inhibitor+statins), the rest was controlled by HIV physicians. Error rate was similar in both surgical and medical wards (34% and 33%, respectively). A total of 29% of errors reached to patients. Patients were exposed to errors a median time of 54 hours. In total, 46.3% of pharmaceutical interventions were accepted.

**Conclusions**: Error rate was as high as in other studies. Medication reconciliation on admission by a pharmacist helps to correct these errors. Collaboration between hospital pharmacists and HIV unit with physicians not specialized in infectious diseases, and the development of strategies are needed to prevent medication errors in HIV patients at admission.

**Abstract P316–Table 1:** Medication errors detected during the study

Type of errors (%)	Description	Example of drugs affected (ratio)
Wrong dose (34)	It was prescribed to be taken one tablet instead of two. Or it was prescribed to be taken 300 mg/24h in patients with CrCl <50 mL/min	LPV/r (4/14) or 3TC (3/14)
Wrong schedule (29)	It was prescribed to be taken once daily instead of twice daily, or vice versa	ETR (5/12); RAL (2/12)
Omission (22)	The rest had partial omission of treatment	All treatment (4/9)
Wrong drug (15)	It was prescribed TDF + FTC	ABC + 3TC (4/6)

3TC, lamivudine; ABC, abacavir; CrCl, creatinine clearance; ETR, etravirine; FTC, emtricitabine; LPV, lopinavir; r, ritonavir; RAL, raltegravir; TDF, tenofovir.

## References

[1] Li EH, Foisy MM (2014). Antiretroviral and medication errors in hospitalized HIV-positive patients. Ann Pharmacother.

[2] Liedtke MD, Tomlin CR, Skrepnek GH, Farmer KC, Johnson PN, Rathbun RC (2016). HIV pharmacist's impact on inpatient antiretroviral errors. HIV Med.

[3] Zucker J, Mittal J, Jen SP, Cheng L, Cennimo D (2016). Impact of stewardship interventions on antiretroviral medication errors in an urban medical center: a 3-year, multiphase study. Pharmacotherapy.

# Sub article: P317 Clinical and genetic factors associated with kidney tubular dysfunction in a real-life single-centre cohort of HIV-positive patients

**Published:** 2016-10-23

**Authors:** Sara Emanuela Salvaggio, Andrea Giacomelli, Felicia Stefania Falvella, Letizia Oreni, Paola Meraviglia, Chiara Atzori, Emilio Clementi, Massimo Galli, Stefano Rusconi

**Affiliations:** 1 DIBIC Luigi Sacco Malattie infettive III Div, ASST Fatebenefratelli Sacco, Milan, Italy; 2 Dipartimento di Farmacologia Clinica Luigi Sacco, ASST Fatebenefratelli Sacco, Milan, Italy; 3 DIBIC Luigi Sacco Malattie infettive I Div, ASST Fatebenefratelli Sacco, Milan, Italy

## Abstract

**Introduction**: Tenofovir (TDF) is one of the most widely used antiretroviral drugs. Despite the high degree of tolerability, a small percentage of patients experienced alteration in tubular function during TDF use. Intracellular TDF disposition is regulated by ATP-binding cassette (ABC) drug efflux transporters, and a reduced transport activity may be implicated in accumulation of TDF into the cells. The aim of our study was to assess the major determinant of TDF-associated tubular dysfunction in a real-life setting including the usefulness of single-nucleotide polymorphisms (SNPs) mapping into ABCC2, ABCC4 and ABCC10 genes.

**Materials and methods**: We retrospectively analyzed all HIV-positive patients who were followed at the Infectious Diseases Unit, DIBIC Luigi Sacco, University of Milan from April 2013 to June 2015. All patients treated with TDF during their antiretroviral history were evaluated for the functional variants mapping in ABCC2 (−24 C>T/rs717620 and 4544 G>A/rs8187710), ABCC4 (3463 A>G/rs1751034) and ABCC10 (rs2125739 T>C). Kidney tubular dysfunction (KTD) was defined as the presence of urine phosphate wasting and/or proteinuria at 24-hour urine analysis.

**Results**: During the period of observation, 158 patients were selected and genotyped; 42 (26.6%) patients experienced a KTD and 116 (73.4%) had a normal tubular function. No statistically significant differences were observed among these two groups of patients regarding age, gender and ethnicity; non-Caucasian patients were two (4.8%) and three (2.6%), respectively. No differences were also observed in the distribution of hypertension and diabetes among groups. Patients who experienced KTD had a longer median duration of therapy in TDF than patients without KTD (6.3 years (interquartile range (IQR) 2.5–9.2) vs. 4.9 (IQR 1.9–7.9); p=0.09) and a higher prevalence of bone disease (23 (54.8%) vs. 32 (27.6%); p=0.002). The percentage of patients with KTD was higher among those with “GG” genotype at rs1751034 of ABCC4 compared to patients with normal tubular function (6 (14.3%) vs. 4 (3.5%), p=0.01) (Figure 1).

No statistically significant differences were observed regarding the distribution of ABCC2 and ABCC10 SNPs. Carriers of “G” allele in homozygous status at rs1751034 of ABCC4 showed a significant association with KTD (odds ratio 4.67, 95% CI 1.25–17.46, p=0.02) in bivariate analysis, but this association was lost in multivariable analysis.

**Conclusions**: In our real-life cohort, 26% of patients treated with TDF manifested signs related to kidney tubular dysfunction. According to our results, ABCC4 rs1751034 should be a genetic determinant of KTD; however, further validation studies are needed for therapy personalization.

**Figure 1:** Distribution of different genotypes at rs1751034 of ABCC4 in patients with or without KTD. *χ square.

# Sub article: P318 Prevalence of drug–drug interactions involving antiretroviral treatment: impact of the integrase inhibitor class

**Published:** 2016-10-23

**Authors:** Peter Messiaen, Charlotte Baecke, Jeroen van der Hilst

**Affiliations:** 1 Department of Infectious Diseases & Immunity, Jessa Hospital, Hasselt, Belgium

## Abstract

**Introduction**: ARV agents pose a high risk for drug–drug interactions (DDIs) with other ARV and non-ARV drugs. Induction or inhibition of different cytochrome P450 enzymes by protease inhibitors (PI) or non-nucleoside reverse transcriptase inhibitors (NNRTI) is one of the major but not exclusive metabolic pathways potentially leading to an increased risk of toxicity or loss of efficacy of ARV and non-ARV drugs. Partly metabolized by other pathways, the integrase inhibitor (INI) class might show a more favourable profile. The aim of this study was to investigate the prevalence of potential DDIs in patients who recently started ARV and to evaluate the effect of INI introduction.

**Materials and methods**: The study population comprised all patients starting ART in our centre from 2009 till April 2016. All prescribed comedications since start of ARV were recorded retrospectively from the medical files. The complete treatment was screened for DDIs using the most recent version of the University of Liverpool HIV Drug Interactions database (www.hiv-druginteractions.org). DDIs were scored as absent, potential, contra-indicated or not assessable due to lack of data.

**Results**: Of the 145 patients included, 28% (n=41) were treated on an NNRTI-based regimen, 30% (n=44) on a PI-based regimen and 42% (n=61) on an INI-based regimen. Dolutegravir (69%, n=42) and elvitegravir (30%, n=18) were the most prescribed INIs. A total of 78% (n=113) of the patients took comedication. Polypharmacy (≥5 comedications) was seen in 26% of patients, significantly correlated with age (p=0.024). Potential DDI was seen in 63% (n=71) of the patients with comedication and in 32% (160/503) of all non-ARV prescriptions. These involved mainly antimicrobial drugs (33%), cardiovascular drugs (19%) and central nervous system drugs (19%). Contra-indicated prescriptions were detected in 1% (n=6), disproportionally more involving gastro-intestinal drugs (66%). PI-based ART was an independent risk factor for potential or contra-indicated DDI (odds ratio (OR) 2.36; 95% CI 1.14–4.90; p=0.030). There was no higher risk associated with NNRTI-based ART (OR 0.66; 95% CI 0.32–1.36) as well as for INI-based regimens (OR 0.64; 95% CI 0.33–1.25). A significantly lower risk for drug interaction was seen with dolutegravir-based treatment (OR 0.47; 95% CI 0.22–0.98; p=0.046), though not for elvitegravir-based ART (OR 1.76; 95% CI 0.64–4.82).

**Conclusions**: Integrase inhibitor use did not result in lower or higher risk for drug–drug interactions in our patient cohort. However, dolutegravir-based treatment showed a significantly lower risk for DDIs, which was not the case for elvitegravir.

# Sub article: P319 Temporal trend of the plasma level of efavirenz: comparison between CYP2B6-516 GG and GT genotype

**Published:** 2016-10-23

**Authors:** Chun Kwan Bonnie Wong, Pui Chung Denise Chan, Shui Shan Lee

**Affiliations:** 1 Special Preventive Programme, Department of Health, Hong Kong Special Administrative Region, Hong Kong; 2 Stanley Ho Centre for Emerging Infectious Diseases, The Chinese University of Hong Kong, Shatin, Hong Kong

## Abstract

**Introduction**: Efavirenz (EFV) is primarily metabolized by hepatic cytochrome P450 (CYP) 2B6, which is genetically polymorphic. Genotype 516TT is associated with decreased plasma clearance of EFV and a higher incidence of neurologic complications. The pharmacokinetic difference between 516GG and 516GT after long-term use of EFV has however received less attention.

**Methods**: Therapeutic drug monitoring (TDM) is available as a supplemental clinical service to HIV patients receiving HAART in Hong Kong. A high-performance liquid chromatography system has been in operation for about 10 years. Mid-dose plasma level of EFV of patients started on an EFV-based treatment regimen at year 1 (defined as more than 2 months and less than 2 years) were evaluated. As a sub-study, EFV-treated patients of Integrated Treatment Centre with blood tests done at two or more time points were analyzed.

**Results**: TDM results of 95 patients were examined in the first part of the study. The mean age at diagnosis of these patients was 40.0 years (SD 11.7), of which, 93 (97.9%) were male. Their CYP2B6-516 genotypes were as follows: GG 48 (50.5%), GT 37 (38.9%) and TT 10 (10.5%), the distribution of which was in Hardy–Weinberg equilibrium. At year 1, the mean EFV level of GT was high at 8.78 mg/L (SD 2.66). The difference between GG and GT was statistically significant (2.89±1.26 mg/L vs. 3.65±1.26 mg/L, t test p<0.01). No significant difference in EFV level between GG and GT could be seen over time when exploring data from 62 patients in the sub-study.

**Conclusion**: EFV level in patients with GT genotype of CYP2B6-516 is generally higher than those with GG genotype in the first 2 years after initiation of EFV regimen. Nevertheless, the difference of EFV level between two genotypes is not significant when temporal changes were evaluated. Differential pattern of auto-induction may explain the results elicited in this study. Extrapolation of the results is however cautioned in view of the small number of patient samples tested, which may also be compounded by the high inter- and intra-individual variation of plasma EFV levels.

# Sub article: P320 European survey on doctor–patient relationship

**Published:** 2016-10-23

**Authors:** Damian Kelly, Kristjan Jachnowitsch, Yuliya Raskevich, Nenad Petkovic, Brian West, Deniz Uyanik, Ian Hodgson, Viken Darakjian, Jorge Garrido Fuentes, Jackie Morton, Nyambe Mukelabai, Sanja Bleak, Stephan Dessler, Ninoslav Mladenovic, Christina Antoniadi

**Affiliations:** 1 Training and Capacity Building Working Group, European AIDS Treatment Group, Brussels, Belgium; 2 Training and Capacity Building Working Group, European AIDS Treatment Group, Kiev, Ukraine; 3 Training and Capacity Building Working Group, European AIDS Treatment Group, Belgrade, Serbia and Montenegro; 4 Training and Capacity Building Working Group, European AIDS Treatment Group, Edinburgh, UK; 5 Training and Capacity Building Working Group, European AIDS Treatment Group, Istanbul, Turkey; 6 Training and Capacity Building Working Group, European AIDS Treatment Group, Bradford, UK; 7 Training and Capacity Building Working Group, European AIDS Treatment Group, Yerevan, Armenia; 8 Training and Capacity Building Working Group, European AIDS Treatment Group, Madrid, Spain; 9 Training and Capacity Building Working Group, European AIDS Treatment Group, York, UK; 10 Training and Capacity Building Working Group, European AIDS Treatment Group, Leeds, UK; 11 Training and Capacity Building Working Group, European AIDS Treatment Group, Zagreb, Croatia; 12 Training and Capacity Building Working Group, European AIDS Treatment Group, Berlin, Germany; 13 Training and Capacity Building Working Group, European AIDS Treatment Group, Athens, Greece

## Abstract

**Introduction**: The European AIDS Treatment Group (EATG) conducted a survey in February 2016 to provide a snapshot on patient experience with their HIV clinicians. During 1 week, 357 responses from 34 countries were received (74% male, 23% female, 1% trans, 2% N/A, all age groups 18–80 were represented in the responses).

**Method**: A pan-European online survey consisting of 37 questions was undertaken using existing networks from across Europe to promote the survey in order to better understand the current care continuum from across the region.

**Results**: While there generally is a high level of trust – 88% of the survey participants have answered “yes” to the question “do you trust your HIV doctor?” – the survey results reveal areas where further efforts on the European level are necessary. Figure 1 shows what topics are being addressed during patients’ appointments with their doctors. In this light, “Treatment Options” (73%), “Side Effects” (65%) and “Diagnostics” (62%) are the most frequently addressed topics.

Given the high importance of the topics of “Adherence” and “Sexual Health,” it is particularly surprising that only 35% and 39% of the participants, respectively, indicated that they discuss these topics with their doctors. Even much less frequently social or psychological issues such as “Recreational Drug Use,” “Legal Rights,” “Reproductive Choices,” “Social Concerns” or “Mental Health” are being discussed. Almost all the topics are addressed less when patients do not trust their HIV doctor. When asked if test results are being explained to them, 57% of participants replied that test results are explained to them very well, 30% replied that this is only sometimes the case and 13% either never receive an explanation or do not understand it. With regard to treatment options, only 56% of the survey participants indicated that they are given a choice between treatment options while this was not the case with 44%.

**Conclusion**: Understanding test results and having a voice in the choice of treatment and care options are central elements for an empowered patient. Whilst the outcome shows that in general patients trust their doctors, there appears to be missed opportunities to identify what really matters to patients and the long-term management of good health. Whilst limited time is a pressure, a broader conversation is needed by both doctors and patients to address the medical, social and psychological aspects of living with HIV.

**Figure 1:** What topics do patients discuss with their HIV doctors?

# Sub article: P321 HIV testing in the community: responding to the Glasgow outbreak

**Published:** 2016-10-23

**Authors:** Lee Middleton

**Affiliations:** 1 NHS Greater Glasgow and Clyde Addiction Service, South West Community Addiction Team, Glasgow, UK

## Abstract

**Introduction**: In 2015, an outbreak of HIV was noted in the population of people who inject drugs (PWID) in Glasgow [1]. By August 2015, over 40 individuals had been identified, where previous years had an annual average of 10 [2]. Dry blood spot testing (DBST) is a non-invasive way to diagnose HIV which can be quickly completed in a community setting.

**Aims**: Increase access to HIV testing for opiate replacement therapy (ORT) patients linked to the South West Community Addiction Team (SWCAT), via DBST, improve acceptability and uptake of HIV testing among target group; increase awareness amongst staff and patients of the outbreak and importance of prevention and testing.

**Materials and methods**: This project used a two-pronged approach. Awareness campaign. Briefing delivered to SWCAT integrated staff team (NHS Greater Glasgow and Clyde and Glasgow City Council); this encompassed both raising awareness of the outbreak and the importance of increasing testing rates. Poster campaign in clinic premises in conjunction with European HIV-Hepatitis Testing Week; a dialogue began with patients regarding the benefits of screening and to begin to move away from focusing on risk-taking behaviour and any sense of stigma. DBST. Testing period identified (initially 1 week – extended to 4 due to high uptake rate). Targeted approach by staff to promote testing to patients. Identified medical officer available throughout testing period at each ORT clinic. Instant access to DBST for those accepting the test.

**Results**: A total of 148 ORT patients were identified attending clinics at SWCAT in November/December 2015. All 148 were offered HIV tests at their clinic appointments. Of this group, 146 (98.6%) accepted the tests, which were completed at the same appointment.

**Conclusions**: The uptake of HIV tests in SWCAT significantly exceeded all expectations. The results show that HIV testing in a community setting, when offered instantly with a “no-wait” approach during an awareness campaign, can be made both highly accessible and acceptable to a population engaged with an ORT programme. By switching the focus of the test away from risk-taking behaviour towards health promotion, it allowed patients to actively make decisions about their health and fully engage with the process.

## References

[1] Health Protection Scotland HPS weekly report. 2015. http://www.hps.scot.nhs.uk/documents/ewr/pdf2015/1526.pdf.

[2] Greater Glasgow and Clyde Rise in HIV cases 2015/16. http://www.nhsggc.org.uk/your-health/public-health/public-health-protection-unit-phpu/current-outbreaks-rise-in-hiv-cases-2015/.

# Sub article: P322 Factors influencing and associated with the decision to join in Thailand's first online supervised HIV self-testing and counselling initiative

**Published:** 2016-10-23

**Authors:** Tarandeep Anand, Chattiya Nitpolprasert, Stephen Kerr, Tanakorn Apornpong, Jintanat Ananworanich, Praphan Phanuphak, Nittaya Phanuphak

**Affiliations:** 1 Adam's Love, Thai Red Cross AIDS Research Centre, Bangkok, Thailand; 2 Biostatistics, HIV Netherlands Australia Thailand Research Collaboration, Thai Red Cross AIDS Research Centre, Bangkok, Thailand; 3 Therapeutics Research, US Military HIV Research Program, Walter Reed Army Institute of Research, Silver Spring, MD, USA; 4 Thai Red Cross AIDS Research Centre, Bangkok, Thailand; 5 HIV Prevention, Thai Red Cross AIDS Research Centre, Bangkok, Thailand

## Abstract

**Introduction**: HIV testing rates are low among men who have sex with men (MSM) and transgender (TG) individuals who contribute >50% of new HIV infections in Thailand [1,2]. Online supervised, finger-prick, HIV self-testing and counselling (eHTC) is an innovative strategy to expand early testing among Thai MSM and TG. We studied acceptability and uptake of this strategy.

**Methods**: In December 2015, the Thai Red Cross AIDS Research Centre (TRCARC) launched an Online Test and Treat implementation research project to explore approaches to engage and retain “hard-to-reach” MSM and TG, in HIV testing and care. Participants recruited and enrolled online via TRCARC's Adam's Love (www.adamslove.org) could choose between (1) eHTC with real-time guidance from counsellors or (2) online counselling followed by private clinic-based testing. Questionnaires assessed reasons for choosing eHTC over clinic-based testing, and feelings post-eHTC utilization.

**Results**: Between December 2015 and May 2016, 99,110 MSM and TG were reached via online study promotions, 264 were screened, 153 (58%) passed the eligibility criteria and 97 (36.7%) were successfully enrolled. Among 97 individuals, 25 (25.8%) selected eHTC while 72 (74.2%) opted for online counselling followed by clinic-based testing. Younger MSM/TG, (median age 25 vs. 29 years, p=0.006), less frequent (previous test >1 year) and first time testers (47.37% vs. 17.74%, p=0.01) and those having previous STIs (20% vs. 11.11%, p=0.015) were more likely to prefer eHTC than clinic-based testing. High-risk behaviours were similar in both groups, with high social media sex-seeking >80% and consistent condom use in the past 6 months <28%. HIV prevalence was significantly higher among eHTC than clinic-based testing participants (16% vs. 1.4%, p=0.02). Preference for eHTC was guided by logistic/time convenience (79%), scheduling flexibility (7%), confidentiality (7%) and altruism (7%). Reasons for declining eHTC included stigma of receiving self-testing kit at home (40%), fear of one's own lack of understanding of self-testing and receiving results alone (28%), fear of finger-prick (24%) and fear of internet glitches while video chatting with counsellors during guidance (8%). Positive perceptions (“it's good and convenient,” “it's amazing,” “HIV testing is normal now”) increased pre- and post-HIV self-testing from 38% to 85% and negative perceptions (“I feel anxious,” “I am scared”) decreased from 38% to 8%.

**Conclusions**: eHTC is feasible to reach high-risk Thai MSM and TG who never or infrequently previously tested for HIV. eHTC has high potential to be scaled up to reach harder-to-reach populations for the first target of UNAIDS 90–90–90 targets [3,4].

## References

[1] Thai Working Group on HIV/AIDS Projections (2010) AIDS Epidemic Model: projections for HIV/AIDS in Thailand 2010–2030 [Internet]. http://www.gfaidsboe.com/Downloads/book/2557/Summary_result_book_1Aug2014.pdf.

[2] National AIDS Committee (NAC) (2012). 2015 Thailand AIDS response progress report, reporting period: fiscal year of 2014 [Internet]. http://www.unaids.org/sites/default/files/country/documents/THA_narrative_report_2015.pdf.

[3] Joint United Nations Programme on HIV/AIDS (UNAIDS) 90–90–90: an ambitious treatment target to help end the AIDS epidemic. http://www.unaids.org/sites/default/files/media_asset/90-90-90_en_0.pdf.

[4] Joint United Nations Programme on HIV/AIDS (UNAIDS) Smart investments. http://www.unaids.org/sites/default/files/media_asset/20131130_smart-investments_en_1.pdf.

# Sub article: P323 Ways in which legal and regulatory barriers hinder the HIV care continuum and 90/90/90 target across Europe

**Published:** 2016-10-23

**Authors:** Lisa Power, Julian Hows, Dorthe Raben, Stine Finne Jakobsen, Sara Croxford

**Affiliations:** 1 Optimising Testing and Linkage to Care for HIV across Europe (OptTEST), Cardiff, UK; 2 OptTEST, Global Network of People Living with HIV, Amsterdam, Netherlands; 3 OptTEST, CHIP, University of Copenhagen, Copenhagen, Denmark; 4 OptTEST, Centre for Research in Infectious Diseases (CHIP), University of Copenhagen, Copenhagen, Denmark; 5 OptTEST, Public Health England, London, UK

## Abstract

**Introduction**: Recent HIV continuum of care data from the European Centre for Disease Prevention and Control [1] shows 78% of responding countries had significant break points relating to diagnosis, 41% in linkage to care and 48% in subsequently accessing treatment. Break points involving care/treatment are far greater in non-EEA countries but exist across Europe. Two-thirds of countries identified legal or policy issues contributory to such gaps.

**Methods**: A literature review done between January and October 2015 for Optimizing Testing and Linkage to Care for HIV across Europe (OptTEST) [2] included 54 documents, including academic and grey literature, identifying a wide range of legal and regulatory barriers.

**Results**: Legal barriers identified included criminalization of HIV transmission and perceived exposure; criminalization of key populations, for example, drug users and sex workers; failure to provide legal protections for these and others, for example, MSM and transgender people. These acted to deter access to HIV services and to impede disclosure of risk activities which might otherwise trigger TasP. Immigration law deterred official access to healthcare for many undocumented migrants and denial of/poor access to ART existed in a number of prison and immigration detention systems. Drug laws in particular were shown to act to increase HIV and decrease access to care, while their reform can directly act to reduce HIV transmission (e.g. in Portugal) [3]. Regulatory barriers were less well documented but there was extensive coverage of testing. Outdated guidelines alongside restrictive practices and regulations acted to hinder proven new testing technologies and settings, including restrictions on who can administer tests; requirement of extensive pre-/post-test counselling; refusal to accept referrals from community testing into care; limited testing sites and restricted types of test. Wider barriers to improving the continuum of care included separation of healthcare into vertical specialities (e.g. drugs care separate from HIV and from TB); lack of case management systems; failure to integrate healthcare and social support; disruption of care between civil and detention authorities. Complex entitlement regulations and charging systems deterred migrants, including even those entitled to healthcare sometimes.

**Conclusion**: Findings suggest a need for consistent, updated evidence-based guidelines for testing and care across Europe and guideline implementation; for reform of laws based on stigma rather than evidence and practices based on custom rather than current knowledge; for better dialogue between policymakers, clinicians, NGOs and people with HIV/in key populations about the actual legal and regulatory barriers which hinder achievement of 90/90/90.

## References

[1] European Centre for Disease Prevention and Control (ECDC) Thematic report on continuum of care; monitoring implementation of the Dublin Declaration. http://ecdc.europa.eu/en/publications/publications/dublin-declaration-continuum-of-care-2014.pdf.

[2] Forthcoming online publication from Optimising Testing and Linkage to Care for HIV across Europe (OptTEST) Health Information Exchange/Centre for Research in Infectious Diseases (CHIP). Copenhagen, Denmark; 2017.

[3] The Drug Policy Alliance (DPA) Drug decriminalization in Portugal: a health-centered approach. http://www.drugpolicy.org/sites/default/files/DPA_Fact_Sheet_Portugal_Decriminalization_Feb2015.pdf.

# Sub article: P324 Integrated HIV, hepatitis B and hepatitis C testing during the 2015 European Testing Week

**Published:** 2016-10-23

**Authors:** Ida Sperle, Brian West, Valerie Delpech, Teymur Noori, Anna Zakowicz, Ann-Isabelle Von Lingen, Nikos Dedes, Jason Farrel, Caroline Daamen, Josip Begovac, Lisa Power, Cary James, Ben Collins, Tudor Kovacs, Ricardo Fernandes, Zoran Dominković, Dorthe Raben

**Affiliations:** 1 Department of Infectious Diseases, Rigshospitalet, University of Copenhagen CHIP, Copenhagen, Denmark; 2 Governance, AIDS Action Europe, Berlin, Germany; 3 HIV and STI Department, Public Health England, London, UK; 4 Unit of Surveillance and Response Support (SRS), European Centre for Disease Prevention and Control, Stockholm, Sweden; 5 Europe Bureau, AIDS Healthcare Foundation, Amsterdam, Netherlands; 6 Policy Working Group, European AIDS Treatment Group, Brussels, Belgium; 7 OptTEST, Positive Voice, Athens, Greece; 8 European HCV and Drug Use Initiative, Correlation Network, Amsterdam, Netherlands; 9 Public Health Capacity and Communication, European Centre for Disease Prevention and Control, Stockholm, Sweden; 10 Department of Infectious Diseases, University of Zagreb School of Medicine, Brussels, Belgium; 11 OptTEST, Individual Consultant, London, UK; 12 Health Improvement, Terrence Higgins Trust, London, UK; 13 Programmes Department, International Lesbian, Gay, Bisexual, Transgender and Queer Youth and Student Organisation (IGLYO), Brussels, Belgium; 14 Portuguese Community Advisory Board, Grupo de Ativistas em Tratamentos (GAT), Lisbon, Portugal; 15 Iskorak, LGBT Health, Zagreb, Croatia

## Abstract

**Introduction**: In the World Health Organization European Region, it is estimated that approximately 2.5 million people are living with HIV (PLHIV) [1] and around 13 and 15 million are living with hepatitis B (HBV) and C viruses (HCV), respectively [2]. Around one in three is unaware that they are living with HIV [3,4] and one in three people has been exposed to either HBV or HCV [2]. European HIV-Hepatitis Testing Week (ETW) is a partnership between civil society, healthcare professionals, governmental and other policy organizations. A dedicated website (www.testingweek.eu) provides a hub for interested organizations to sign up and download materials to support planned activities.

**Materials and methods**: ETW 2015 took place from 20 to 27 November 2015. All participating organizations were invited to complete an online evaluation survey with questions about their carried out ETW activities. Data were entered into the Research Electronic Data Capture system (REDCap) hosted at CHIP, Rigshospitalet, University of Copenhagen. Five electronic survey reminders were sent prior to the survey deadline, 15 January 2016. Data were extracted in Excel format from REDCap and descriptive statistics were produced as frequencies and respective proportions in Excel.

**Results**: Of the 417 organizations that signed up, 194 from 39 countries submitted the evaluation survey (46.5%). The majority of respondents were NGOs (65.5%) followed by healthcare professionals/hospitals/clinics (18.0%) and governmental and other policy organizations (9.3%). The majority of respondents carried out testing activities (Figure 1) and awareness-raising activities.

Several respondents reported testing for more than one of the three conditions during ETW (Table 1).

The percentage of respondents reporting increase in testing during ETW compared to an average week was 78% for HIV, 74% for HBV and 70% for HCV. ETW has brought forward many innovative best practice examples from all over Europe of how testing and awareness raising can be done. Examples include designing of coffee cup sleeves promoting HIV testing distributed to coffee shops throughout Dublin, dissemination sessions in the streets, use of rapid tests, for example, via a mobile clinic in Kiev doing outreach testing to MSM and collaboration across sectors and between organizations and institutions.

**Conclusions**: Several organizations tested for HIV, HBV and HCV (35) and reported significant increases in testing during ETW. ETW has proven to be an efficient initiative in uniting Europe in promoting testing and in increasing testing for HIV, HBV and HCV through innovative activities carried out by ETW participants.

**Figure 1:** Activities during ETW (N = 194).

**Table 1:** Organizations carrying out testing activities during ETW (N=194)

	HIV testing	HIV & HBV testing	HIV & HCV testing	HBV & HCV testing	HIV, HBV & HCV testing
Organizations, N (%)	158 (81)	37 (19)	59 (30)	36 (19)	35 (18)

## References

[1] Joint United Nations Programme on HIV/AIDS (UNAIDS) Fact sheet. http://www.unaids.org/en/resources/campaigns/HowAIDSchangedeverything/factsheet.

[2] World Health Organization (WHO) Data and statistics. http://www.euro.who.int/en/health-topics/communicable-diseases/hepatitis/data-and-statistics.

[3] Hamers FF, Phillips AN (2008). Diagnosed and undiagnosed HIV-infected populations in Europe. HIV Med.

[4] Coenen T, Lundgren J, Lazarus JV, Matic S (2008). Optimal HIV testing and earlier care: the way forward in Europe. HIV Med.

# Sub article: P327 Modelling the cost effectiveness of HIV care in Poland shows clear benefit while transmission risk is considered in the calculations

**Published:** 2016-10-23

**Authors:** Justyna Kowalska, Grzegorz Wójcik, Jakub Rutkowski, Magdalena Ankiersztejn-Bartczak, Ewa Siewaszewicz

**Affiliations:** 1 Hospital for Infectious Diseases, Medical University of Warsaw, Warsaw, Poland; 2 HTA Consulting, Krakow, Poland; 3 FES, Foundation of Social Education, Warsaw, Poland; 4 Gilead Sciences, Poland, Warsaw, Poland

## Abstract

**Introduction**: The HIV epidemic remains a major global health issue. Data from cost-effectiveness analyses are usually based on CD4+ counts and morbidity. Here, we evaluate the impact of sexual HIV transmission due to delayed cART on the cost effectiveness of care.

**Methods**: A lifetime Markov model (1-month cycle) was developed to estimate lifetime costs, clinical outcomes and cost per quality adjusted life years (QALY) gained for 1- and 3- year delay in starting cART (as compared to staring immediately at linkage to care). Health states included into the model were asymptomatic HIV, AIDS defining condition (mild, moderate, severe) [1], Hodgkin's Lymphoma and non-AIDS defining condition (>20 illnesses/events in total). Mortality rates and utility values were obtained from published literature. The number of new infected persons was estimated based on sexual orientation, number of sexual partners per year, number of sex acts per month, frequency of condom use and use of cART [2]. We assumed that patients had HIV RNA <50 copies/ml immediately since starting cART and for a lifetime. Transmission risk was presented for three scenarios: low, medium and high (Table 1).

For the input data Test and Keep in Care (TAK) project, cohort pre-clinical and clinical information was used [3]. Costs of care, cART and potential life-years lost were based on estimated total costs and the difference in expected QALY gained between HIV positive and average person in Polish population. Analysis was performed from the public payer perspective therefore costs were based on real expenditures of Ministry of Health (MoH), National Health Fund, available studies [16–18] and expert's opinion. Costs and effects were discounted at rates of 5% and 3.5%, respectively. Cost-effectiveness threshold for incremental cost-utility ratio (ICUR) was set to 125 955 PLN (29 312 EUR) according to MoH requirements.

**Results**: Input data were available for 141 patients from TAK cohort. Estimated number of new HIV infections for low, medium and high-risk scenarios were 0.28, 0.61, 2.07 and 0.82, 1.80, 6.11 with 1-year and 3-year delay, respectively. This reflected QALY loss due to cART delay of 0.52, 1.13, 3.84 and 2.02, 4.43, 15.03 for 1 and 3-year delay, respectively. If additional costs of treatment and potential life-years lost due to new HIV infections were not taken into account, initiating cART immediately at linkage to care was not cost effective irrespective of cART delay. When additional costs and QALY lost were included, immediate cART initiation was dominant (cheaper and more effective) regardless of the chosen scenarios (Table 1).

**Conclusions**: Accounting for HIV transmission in cost-effectiveness analysis provides further evidence supporting immediate-initiation of HIV treatment from a public payer perspective.

**Abstract P327–Table 1:** Lifetime Markov model results for the cost per quality adjusted life years (QALY) for 1- and 3-year delay in starting cART

			Without transmission			Low risk			Medium risk			High risk		
Number of sexual partners per year			–			3			10			50		
Number of monthly sex acts			–			10			20			20		
Condom use per acts pct			–			90%			50%			0%		
Delay treatment	Category		Immediate cART	Delayed cART	Difference	Immediate cART	Delayed cART	Difference	Immediate cART	Delayed cART	Difference	Immediate cART	Delayed cART	Difference
1 year	Sexual HIV transmission	Infected person	0.00	0.00	0.00	0.01	0.28	−0.27	0.03	0.61	−0.59	0.09	2.07	−1.99
	Total treatment costs	PLN	516,333	473,560	42,773	516,333	473,560	42,773	516,333	473,560	42,773	516,333	473,560	42,773
	Cost of treatment new infections	PLN	0	0	0	5,895	125,975	−120,080	12,646	277,067	−264,421	42,181	939,075	−896,894
	Total costs (total treatment costs + cost of new infections)	PLN	516,333	473,560	42,773	522,228	599,536	−77,307	528,979	750,627	−221,648	558,515	1,412,636	−854,121
	QALY	QALY	11.29	11.15	0.14	11.29	11.15	0.14	11.29	11.15	0.14	11.29	11.15	0.14
	QALY lost	QALY	0.00	0.00	0.00	0.02	0.52	−0.50	0.04	1.13	−1.09	0.15	3.84	−3.70
	QALY (adj)	QALY	11.29	11.15	0.14	11.27	10.64	0.63	11.25	10.02	1.23	11.14	7.31	3.83
	ICUR	PLN per QALY			313,484			dominates			dominates			dominates
3 year	Sexual HIV transmission	infected person	0.00	0.00	0.00	0.04	0.82	−0.78	0.08	1.80	−1.73	0.25	6.11	−5.86
	Total treatment costs	PLN	516,333	369,129	147,204	516,3333	369,129	147,204	516,333	369,129	147,204	516,333	369,129	147,204
	Cost of treatment new infections	PLN	0	0	0	5,947	262,526	−246,580	34,208	577,394	−543,186	114,103	1,956,986	−1,842,882
	Total costs (total treatment costs + cost of new infections)	PLN	516,333	369,129	147,204	532,280	631,656	−99,376	550,541	946,523	−395,982	630,437	2,326,115	−1,695,678
	QALY	QALY	11.29	10.35	0.94	11.29	10.35	0.94	11.29	10.35	0.94	11.29	10.35	0.94
	QALY lost	QALY	0.00	0.00	0.00	0.06	2.02	−1.96	0.12	4.43	−4.31	0.41	15.03	−14.62
	QALY (adj)	QALY	11.29	10.35	0.94	11.23	8.33	2.90	11.17	5.91	5.25	10.88	−4.68	15.56
	ICUR	PLN per QALY			156,335			dominates			dominates			dominates

*Dominates = cheaper and more effective*.

## References

[1] Mocroft A, Sterne JA, Egger M, May M, Grabar S, Furrer H (2009). Variable impact on mortality of AIDS-defining events diagnosed during combination antiretroviral therapy: not all AIDS-defining conditions are created equal. Clin Infect Dis.

[2] Lasry A, Sansom SL, Wolitski RJ, Green TA, Borkowf CB, Patel P (2014). HIV sexual transmission risk among serodiscordant couples: assessing the effects of combining prevention strategies. AIDS.

[3] Ankiersztejn-Bartczak M, Firlag-Burkacka E, Czeszko-Paprocka H, Kubicka J, Cybula A, Horban A (2015). Factors responsible for incomplete linkage to care after HIV diagnosis: preliminary results from the Test and Keep in Care (TAK). HIV Med.

# Sub article: P328 Targeting HIV testing at a population level: cost-effectiveness of three approaches

**Published:** 2016-10-23

**Authors:** Cristina Gomez Ayerbe, Alfonso Muriel, Carlos Reverte, Pilar Perez Elias, Santos Del Campo, Maria Jesus Vivancos, Cristina Santos, Maria Eugenia Calonge, Almudena Uranga, Ana Moreno, Santiago Moreno, Jose Luis Casado, Maria Jesus Perez Elias

**Affiliations:** 1 Infectious Diseases, Hospital Ramon y Cajal, Madrid, Spain; 2 Statistics, Hospital Ramon y Cajal, Madrid, Spain; 3 Primary Care Centre, Centro de Salud Garcia Noblejas, Madrid, Spain

## Abstract

**Introduction:** Targeted HIV testing has been proposed as the most efficient strategy for HIV diagnosis in low prevalence populations. We aimed to compare cost effectiveness of three HIV testing targeted approaches, previously validated, to predict HIV infection.

**Methods:** All participants in DRIVE study (non-targeted HIV testing program in emergency department and primary care centre (PCC)) were tested for HIV and filled out a self-administered HIV Risk Exposure and Clinical Conditions Questionnaire (RE&CI-Q). The RE&CI-Q included six questions to evaluate risk of exposure to HIV and 14 HIV-associated clinical indicators (from HIV Indicator Diseases across Europe Study (HIDES) list [1]). One affirmative answer defined the person as being at risk. The other two tools evaluated were as follows: Denver HIV Risk Score (DHRS) with a cut-off >30 and HIDES using only 14 clinical indicators. Using DRIVE study database, we calculated sensitivity (Sn), specificity (Sp), positive predictive value (PPV) and negative predictive value (NPV) of the three tools, considering the gold standard confirmed cases of HIV infection with EIA/WB. Number of missed HIV infections (NMHI), tests avoided and incremental costs/effectiveness ratio (ICER) were also calculated.

**Results:** A total of 5329 participants of age 18–60 years completed RE&CI-Q and rapid HIV test in the DRIVE study (69.3% in PCC). In total, 50.4% women and median age 37 years (28–47). Confirmed new HIV infection was detected in 22 (0.41%) with this non-targeted strategy. The percentages of population identified at risk for HIV infection by a positive RE&CI-Q, DHRS >30 and at least one item of 14 HIDES list selection were 51.2%, 39.7% and 26.9%, respectively. Sn, Sp, PPV, NPV, NMHI and test avoided were 100%, 49%, 0.80%, 100%, 0 and 2601, respectively in RE&CI-Q approach, 72.7%, 60.41%, 0.76%, 99.8%, 6 and 3212 in DHRS, and 91%, 74.4%, 1.4%, 99.9%, 2 and 3948 in 14 items of HIDES list. The cost per new HIV diagnosis was 552€ (HIDES), 992€ (RE&CI-Q) and 1058€ (DHRS). ICER per new HIV diagnosis -66€ for RE&CI-Q and -506€ for HIDES respect DHRS strategy.

**Conclusions:** All approaches avoided HIV tests compared with routine strategy, but only RE&CI-Q captured all HIV-infected subjects detected by the non-targeted strategy. Cost of each NHIVD obtained using RE&CI-Q compared to HIDES list is low with respect to the benefit obtained.

## References

[1] Sullivan AK, Raben D, Reekie J (2013). Feasibility and effectiveness of indicator condition-guided testing for HIV: results from HIDES I (HIV Indicator Diseases across Europe Study). PLoS One.

# Sub article: P329 HIV linkage to care: impact of a proactive intervention in a health area of Spain

**Published:** 2016-10-23

**Authors:** Santos del Campo, Cristina Gómez Ayerbe, María Jesus Vivancos Gallego, Ana María Moreno Zamora, Jose Luis Casado Osorio, Elena Loza de Bobadilla, Juan Carlos Galán Montemayor, Mario Rodriguez Dominguez, Fernando Dronda Nuñez, Matilde Sanchez Conde, Santiago Moreno Guillén, María Jesús Pérez Elías

**Affiliations:** 1 Infectious Diseases IRYCIS, Hospital Ramón y Cajal, Madrid, Spain; 2 Microbiology, Hospital Ramón y Cajal, Madrid, Spain

## Abstract

**Introduction:** Linkage to care is one of the essential steps in HIV cascade of care. To evaluate the impact of an active intervention aimed to shorten time from first HIV EIA result to first HIV outpatient clinic visit.

**Methods:** From 1 January to 30 June 2015 (first period) and 2016 (second period), we identified all first positive HIV EIA (HIV) results obtained in the Microbiology Laboratory Department of Ramón y Cajal Hospital (RyC). All samples came from two main settings: hospital departments (HD) or primary health area (PHA). In 2015 period, HIV+ results were electronically informed and when possible prescriber physician was alerted by phone, that a second sample needs to be sent to confirm serology. In the 2016 period addition to the above mentioned, all HIV+ results were weekly identified, and we phoned the requesting physician informing the HIV+ result and recommending that the confirmation and the first HIV visit should be done as soon as possible at the HIV RyC outpatient clinic. Number and result of HIV tests, linkage to care at RyC HIV clinic or other clinic (rate and time to first visit) were compared between the two periods.

**Results:** Overall 21,049 first tests were requested (9969 and 11,072 in first and second period). Absolute number and rate of new HIV diagnoses (NHIVD) were 111 (0.53%) (60 (0.61%) and 51 (0.46%); p=0.16). No differences were observed in NHIVD in first and second period according to sex (women 20% vs. 12%; p=0.3), age (38 vs. 37 mean years; p=0.6) or setting (HA 35% vs. 35%; p=1) between first and second periods. From patients with at least 1 month of follow-up (108), unadjusted rate of linkage to care was 50/60 (83.3%) versus 46/48 (95.8%); p=0.04, for first and second period. Mean time to linkage to care was (82±157 and 27±31 days; p=0.019). In an unadjusted analysis, age and setting (HD or PHA) presented same rate of linkage to care, while women had lower rates. In an estimative model, only a trend towards a higher probability to linkage to care was observed for second period (OR 4.6 (09;22.3); p=0.07), when it was adjusted by sex.

**Conclusions:** A higher rate of linkage to care was observed in the intervention period, but the effect was attenuated by sex.

# Sub article: P331 Retention of mother–baby pairs in care and treatment through mother–baby care point initiative in Eastern Uganda

**Published:** 2016-10-23

**Authors:** Mary Abwola Olwedo, Noah Lukoda, Bud Crandall

**Affiliations:** 1 STAR-E, Management Sciences for Health, Mbale, Uganda

## Abstract

**Introduction:** The Ugandan Ministry of Health adopted Option B+ in 2013 for the elimination of mother-to-child transmission of HIV. This required the mother to attend multiple services delivery points for eMTCT program and ART clinic for her care and treatment as well as the baby's EID services after birth. The mothers didn't want to visit the ART clinic after birth because of stigma. The MoH became concerned over high loss-to-follow-up rates and introduced mother–baby care point within the MCH department.

**Methods:** In 2014, the MoH recommended the establishment of mother–baby care points within MCH departments, where midwives provide daily one-stop services to mothers receiving Option B+ with their HIV-exposed infants until child is 18 months old, when mother and child, if HIV positive, are transferred to the ART clinic. Strengthening TB and HIV & AIDS Responses in Eastern Uganda (STAR-E), a USAID project funded by PEPFAR and implemented by Management Sciences for Health, supported the MoH to establish mother–baby care points at MCH departments in 154 health facilities in Eastern Uganda from October 2014 to February 2015 and also did an operational research to understand the impact of MBCP as compared to the old model of implementation.

**Lessons learned:** STAR-E assessed for retention among 496 mother–baby pairs enrolled into Option B+ program during when the MBCP had not been established in 34 health facilities and found the following: 52% were retained at 3 months and 44% at 6 months. And at 3 months, post-delivery period when they were transferred and receiving services at the ART clinic, only 20% were retained. After MBCP were established, the project reviewed records in the same facilities from October 2014 to March 2016 and found higher retention after delivery than earlier research. Of the 277 mother–baby pairs enrolled, 72% were retained at 3 months post-partum, 64% at 6 months, 55% at 9 months and 41% by 12 months, as most of the babies are weaned by then, second DNA PCR done to determine if baby has been protected.

**Conclusion:** Mother–baby care points, where HIV and MCH services are integrated, improved retention of mother–baby pairs in Option B+ during the post-delivery period. This enabled more of the HIV-exposed infants to access DNA PCR twice, nutritional assessment, cotrimoxazole prophylaxis and nevirapine prophylaxis. More HIV-positive infants into care and treatment since they were in contact with the health system more often and mothers were retained on ART.

# Sub article: P332 Efficiency of antiretroviral therapy in Russia

**Published:** 2016-10-23

**Authors:** Anastasia Pokrovskaya, Nadezhda Kozyrina, Uliya Guschina, Zoya Suvorova, Oleg Yurin, Vadim Pokrovskiy

**Affiliations:** 1 Russian Federal AIDS Centre, Central Research Institute of Epidemiology, Moscow, Russian Federation; 2 Deparment of General and Clinical Pharmacology, Peoples’ Friendship University of Russia, Moscow, Russian Federation

## Abstract

**Introduction:** Free ART is available in Russia for PLH, who visit special AIDS centres. According to current guidelines, ART is eligible for patients with CD4 <350 cells/mL. At the beginning of the year 2015, more than 200,000 PLH were on ART. The aim of this study was to characterize the basic aspects of antiretroviral therapy among PLH who visited AIDS centres in Russia in order to develop recommendations for new treatment guidelines.

**Methods:** Multicentre, open-label study with the inclusion of a retrospective model. We analyzed medical records and questionnaires of 7000 adult patients, who visited AIDS centres and signed an informed consent form in 27 regions of Russia in April to July 2014. Funding support for this study was provided by Bristol-Myers Squibb.

**Results:** A total of 3406 (49%) of all recruited participants were females, one was transgender. A total of 4445 (60%) of all participants were on ART which was initiated at mean CD4 – 224.6±138.9; median – 216 (1–1400) cells/mL. Termination of therapy in the time of the study was recorded in 10.1% of patients. Brand name ART drugs were mainly used in the period of the study. The most commonly prescribed ART combinations for naïve patients were as follows: ZDV+3TC+EFV (26.6%), ZDV+3TC+LPV/r (21.7%), ZDV+3TC+ATV/r (8.9%). The average duration of ART was 34 months (max. – 16 years), 18.7% of study participants were on ART over 5 years. A total of 52.3% of patients received the first ART combination, 29.1% – the second (max. – nine ART regimens in patient). The main causes of treatment regimens change were adverse events – 43.3%, simplification (reducing the number of pills and multiplicity) – 27%, pregnancy – 11.2%. Virologic failure was the cause of ART change only in 3% of patients. A total of 83.9% of patients in the study reached HIV RNA <1000 copies/mL and 69.1% – less 400 copies/mL at the end of the first year of treatment. Among patients, who have a serodiscordant regular sexual partner, only 66.7% were on ART.

**Conclusions:** The majority of patients receiving ART in Russia have not yet a very long treatment experience. CD4 level at the moment of ART initiation was low. Though old-fashioned ART combinations were effective and tolerable in a part of the patients, the number of adverse effects were significant. Measures are needed to encourage earlier ART initiation and use drugs with lower toxicity.

# Sub article: P333 Closing the gap of perinatal HIV infection in Hong Kong

**Published:** 2016-10-23

**Authors:** Chun Kwan Bonnie Wong, Wai Chi Ada Lin, Ka Hing Wong

**Affiliations:** 1 Special Preventive Programme, Department of Health, HKSAR, Hong Kong

## Abstract

**Introduction:** To achieve the ultimate goal of eliminating perinatal transmission, we reviewed and identified gaps of the current public health programme for the prevention of mother-to-child transmission (PMTCT) of HIV in Hong Kong, a region with low HIV seroprevalence of <0.01% in the antenatal population. The Universal Antenatal HIV Testing Programme (UATP) was introduced in 2001, with an aim to interrupt MTCT through timely diagnosis and management of infected expectant mothers. The programme was strengthened with implementation of rapid HIV testing component in 2008 to offer rapid HIV test in labour wards for women who did not receive testing in early antenatal period.

**Materials and methods:** We reviewed the programme performance, and matched with perinatal infections reported.

**Results:** UATP has high coverage rate of >98% in recent years. From 2001 to 2014, 3 perinatal infections were identified out of 72 infants born to HIV-infected mothers. All were detected before 2007, two of which were due to late presentation to antenatal care without participation in UATP. The other was due to failure of intra-partum and post-partum intervention when the mother presented 6 days prior to her preterm delivery. The incorporation of rapid HIV testing in 2008 had filled the gap for late-presenting pregnant women so that interventions could be offered to HIV-infected women not identified by UATP. Since 2008, the percentage of women with HIV test results known prior to delivery remained above 98.6%; and 97% of HIV-positive mothers and their babies had received either three-part or two-part ART. However, five cases of HIV-infected children born to their infected mother who were tested negative by UATP in the early antenatal period were reported in 2009–2015. Unprotected sex during pregnancy was the common risk factor. All five mothers and all but one of the spouses/partners were either non-Hong Kong residents or originated from Asian or African countries where the HIV prevalence was higher than Hong Kong, highlighting its unique epidemiological pattern.

**Conclusions:** The gap in PMTCT in Hong Kong lies in the HIV-infected women who seroconverted after they were tested negative in the early antenatal period. Partner counselling and testing, enhancement of safer sex, targeted HIV retesting at third trimester for pregnant women based on their epidemiological and behavioural risks are options to close the gap.

# Sub article: P334 “The first 90”: how close can we get with home-based HIV testing? First results from recruitment for the CASCADE trial in rural Lesotho

**Published:** 2016-10-23

**Authors:** Niklaus Daniel Labhardt, Isaac Ringera, Thabo Ishmael Lejone, Josephine Muhairwe, Christiane Fritz, Thomas Klimkait, Tracy Renee Glass

**Affiliations:** 1 Medical Services and Diagnostic, Swiss Tropical and Public Health Institute, Basel, Switzerland; 2 Towards 90–90–90 Project, SolidarMed, Swiss Organization for Health in Africa, Butha-Buthe, Lesotho; 3 Towards 90–90–90 Project, SolidarMed, Swiss Organization for Health in Africa, Maseru, Lesotho; 4 SolidarMed, Swiss Organization for Health in Africa, SolidarMed Headquarters, Lucerne, Switzerland; 5 Molecular Virology, Department of Biomedicine, University of Basel, Basel, Switzerland; 6 Epidemiology and Public Health, Swiss Tropical and Public Health Institute, Basel, Switzerland

## Abstract

**Introduction**: The first of the UNAIDS 90–90–90 targets aims at 90% coverage of HIV testing and counselling (HTC) [1]. Studies on HTC at the homes of individuals report HTC uptake (individuals tested/individuals encountered at home) of >90% [2]. However, HTC coverage (individuals knowing their status/individuals living in targeted area) remains below 90% because of persons absent during home-based HTC [3]. This study assesses the HTC coverage achieved in Lesotho after two consecutive home visits in order to achieve maximal coverage and to cover presence during the week and on weekends.

**Materials and methods**: The study was conducted in Lesotho, Southern Africa. Data were derived from home-based HTC campaigns serving to recruit HIV-infected individuals for the CASCADE trial (NCT02692027). Assessment of HTC coverage after two home visits is a nested study in the published CASCADE trial protocol [4]. The primary outcome of interest was the HTC coverage in targeted areas after two visits. Counsellors visited randomly selected villages or urban areas moving door to door and offering HTC to all household members. Each area was visited twice, once during the week and once over the weekend. Household members were defined as spending at least one night at least twice a month in that household. The duration of the HTC campaigns was from 22 February to 3 July 2016. Data were captured on tablet computers and synchronised daily [5].

**Results**: Counsellors visited 6429 occupied households with 17,887 household members in 60 rural villages and 17 urban areas; 1988 (30.9%) households were revisited because of members absent at first visit. Among individuals encountered at home, 1381 (9.5%) were already known to be HIV infected. Among the 13,193 with unknown HIV status, 11,268 (85.4%) accepted HTC. HTC coverage in visited areas increased from 62.7%, after the first visit, to 70.5%, after the second visit (Figure 1). Table 1 shows HTC uptake and HTC coverage after two visits, stratified by age and gender. HTC uptake was similar among men and women, but coverage was lower among men due to a lower proportion encountered at home.

**Conclusions**: A second catch-up visit on a weekend increased the proportion of persons knowing their HIV status by 8%, but home-based HTC still fell short of the targeted 90% coverage. Future strategies need to combine home-based HTC with approaches specifically tailored to frequently absent household members, such as testing at the workplace or school-based HTC or self-testing.

**Figure 1:** HTC coverage after first (weekday) and second visit (weekend). Individuals with missing data on HIV status were excluded (n=157).

**Table 1:** Proportion of individuals encountered at home, HTC uptake and HTC coverage stratified by age and gender. Individuals with missing data on age, gender were excluded (n=170)

	Total	n (%)	Odds ratio (95% CI)	p
**Encountered at home**				
Women ≥15 years	8115	7211 (88.9)	1	
Men ≥15 years	5209	3668 (70.4)	0.30 (0.27–0.33)	<0.001
Children <15 years	4393	3534 (80.5)	0.52 (0.47–0.57)	<0.001
**HTC uptake**				
Women ≥15 years	7211	6204 (86.0)	1	
Men ≥15 years	3668	3116 (85.0)	0.92 (0.82–1.03)	0.13
Children <15 years	3534	3048 (86.3)	1.02 (0.91–1.14)	0.77
**HTC coverage**				
Women ≥15 years	8115	6204 (76.5)	1	
Men ≥15 years	5209	3116 (59.8)	0.46 (0.43–0.49)	<0.001
Children <15 years	4393	3048 (69.4)	0.70 (0.64–0.76)	<0.001

## References

[1] Joint United Nations Programme on HIV/AIDS (UNAIDS) 90–90–90: an ambitious treatment target to help end the AIDS epidemic.

[2] Labhardt ND, Motlomelo M, Cerutti B, Pfeiffer K, Kamele M, Hobbins MA (2014). Home-based versus mobile clinic HIV testing and counseling in rural Lesotho: a cluster-randomized trial. PLoS Med.

[3] Sharma M, Ying R, Tarr G, Barnabas R (2015). Systematic review and meta-analysis of community and facility-based HIV testing to address linkage to care gaps in sub-Saharan Africa. Nature.

[4] Labhardt ND, Ringera I, Lejone TI, Masethothi P, Thaanyane T, Kamele M (2016). Same day ART initiation versus clinic-based pre-ART assessment and counselling for individuals newly tested HIV positive during community-based HIV testing in rural Lesotho – a randomized controlled trial (CASCADE trial). BMC Pub. Health.

[5] Visible Impact Cascade trial. https://www.visibleimpact.org/projects/1197-cascade-trial.

# Sub article: P335 Characterization of an inmate population followed in an infectious diseases department in the centre of Portugal

**Published:** 2016-10-23

**Authors:** Maria Isabel Casella, Bianca Ascenção, Ana Teresa Goes, Nuno Pinto Luís, Ana Catarina Messias, Ana Paula Brito, Sá Joana, José Poças

**Affiliations:** 1 Infectious Diseases Department, Hospital de São Bernardo, Centro Hospitalar de Setúbal EPE, Setúbal, Portugal; 2 Internal Medicine Department, Hospital do Litoral Alentejano, Santiago do Cacém, Portugal

## Abstract

**Introduction:** HIV and HCV monitoring among prison inmates is essential in countries with a high HIV/HCV prevalence. Recent studies on this topic are sparse in Portugal and other European countries. The monitoring of HIV and HCV prevalence among inmates, and the characterization of this population, might be a useful measure to study the epidemiological situation.

**Materials and methods:** The HIV and HCV prevalence was estimated among inmates of two male prisons in the centre of Portugal (Pinheiro da Cruz and Setúbal), followed in a medical appointment in an infectious diseases department between 2014 and 2016. Data were obtained from the hospital medical records. Collected information included variables such as age, country of birth, transmission risk, serological status and adherence to consultation and therapy. Patients were considered as refractory to consultation if they had fewer than two consultations per year.

**Results:** Approximately 1000 men were incarcerated in those prisons at the time of data collection. In total, 82 (8%) were under follow-up in our hospital. A total of 36 (44%) of the inmates were co-infected with both HIV and HCV, 31 (38%) had solely HCV infection and only 15 (18%) had HIV monoinfection. In total, 91% had history of intravenous drug abuse, although only 10 were on methadone maintenance treatment. In total, 43% of the 67 HCV-positive inmates were being or had been treated for HCV infection, and 18 (62%) had already obtained sustained virological response. Out of a total of 51, only 6 of the HIV-positive patients were not under HAART at the moment. In total, 38 (84%) of the patients undergoing treatment had undetectable viral load. In total, 62 (79%) were adhering to both HIV and HCV medication as prescribed. A total of 13 patients were considered as refractory to the consultation. At the time of data collection, only three of these refractory patients were still incarcerated.

**Conclusion:** HIV and HCV prevalence in the inmate population was 5–13 times higher than the general population in Portugal, which is a major public health issue. Adhesion to HAART is higher than in the general population, probably due to controlled medication distribution in prison facilities. The prevalence of patients undergoing treatment for HCV is approximately the same as in general population. National data on HIV and HCV prevalence in prison facilities are essential to implement prevention interventions and to improve screening and treatment for these two chronic conditions, as well as to implement measures to increase adherence to follow-up.

# Sub article: P336 Rationales for indicator conditions-based HIV testing data from the Emergency Department in the Hospital for Infectious Diseases in Warsaw

**Published:** 2016-10-23

**Authors:** Karolina Pyziak-Kowalska, Justyna Kowalska, Andrzej Horban

**Affiliations:** 1 Emergency Department, Hospital for Infectious Diseases, Warsaw, Poland; 2 Department for Adults Infectious Diseases, Medical University of Warsaw, Warsaw, Poland

## Abstract

**Introduction:** HIDES study has shown that indicator conditions-based HIV testing may offer better results than standard approach. It has also been proven that populations with the HIV prevalence of >0.1% should be routinely screened for HIV. Currently, routine HIV testing for indicator conditions is not covered by public healthcare in Poland, which may delay or miss the opportunity for HIV diagnosis. The aim of this study was to evaluate HIV testing patterns among patients presenting with specific indicator condition, that is, ongoing mononucleosis-like illness in the Emergency Department (ED) and referred for further diagnostics to hospital.

**Materials and methods:** We conducted retrospective analysis of medical records of patients referred from the ED with ongoing mononucleosis-like illness to the Hospital for Infectious Diseases in Warsaw for further diagnosis within past 12 months (1 May 2014–30 April 2015). Patients were eligible if being 18 years up.

**Results:** In total, 173 patients were consulted at the ED with a mononucleosis-like illness, 94 men and 79 women, with a median age of 26 years; 72 (41.6%) were admitted to hospital; 54 (75%) were offered HIV test and all expressed consent; 4 (5.5%) patients were diagnosed with HIV infection, referred to HIV clinic and linkaged to care. The continuum of care for mononucleosis-like illness is presented in Figure 1.

**Conclusion:** The rate of HIV diagnosis among patients hospitalized due to mononucleosis-like illness was high (5.5%), confirming clear benefit in routine testing of this group of patients. According to our analyses, 58% patients missed the opportunity for HIV testing in ED due to the lack of such healthcare program. With presented rate, this translated to six HIV patients who may still remain unaware of HIV infection. Standards of care for mononucleosis-like illness should include routine HIV testing, which needs additional financing and attention from public healthcare representatives and other stakeholders.

**Figure 1:** Continuum of care for mononucleosis-like illness.

# Sub article: P337 The changing epidemiology of newly diagnosed HIV-infected adults in New York City

**Published:** 2016-10-23

**Authors:** Benjamin Patterson, Jason Zucker, Tanya Ellman, Ellen Morrison, Magdalena Sobiesczcyk

**Affiliations:** 1 Division of Infectious Diseases, Columbia University Medical Center, New York, NY, USA

## Abstract

**Introduction:** In keeping with the US National HIV/AIDS strategy, New York City has attained a >90% rate of persons living with HIV who know their serostatus over the years 2008 to 2012. To maintain this high standard, it is imperative to target HIV testing and linkage to care to the highest risk groups. We investigated a cohort of newly diagnosed individuals at an academic hospital in Northern Manhattan to evaluate changes in the epidemiology and clinical characteristics with a focus on men who have sex with men (MSM) and those over the age of 50 years.

**Materials and methods:** This was a retrospective review of all new HIV diagnoses between 1 January 2006 and 1 August 2015. Eligible patients were >18 years old, had a new positive HIV test and a CD4 cell count within 90 days of diagnosis. Univariate and multivariate analyses were performed to compare clinical and demographic characteristics of individuals diagnosed in 2006 to 2010 (early) and 2011 to 2015 (late) periods.

**Results:** There were a total of 578 new HIV diagnoses: 294 in 2006 to 2010 with mean age 38 years and mean CD4 254 cells/µL; 284 in 2011 to 2015 with mean age 37 years, mean CD4 286 cells/µL. The proportion of HIV diagnoses made in the ED increased from 9% to 34% in the early compared to late period with a concomitant decrease in inpatient diagnoses from 44% to 19% (p<0.0005). There were more diagnoses among individuals self-reporting as MSM in 2011 to 2015 compared to earlier (46.5% vs. 29.6%, p<0.0005); overall MSM tended to be younger with higher mean CD4 cell count than non-MSM (384 vs. 284 cells/µL, p=0.0025). Individuals ≥50 years were more likely to have CD4 <200 cells/µL compared to their younger counterparts and this persisted in 2011 to 2015 (45.3% vs. 29.0%, p=0.009).

**Conclusion:** We noticed a shift in the epidemiology and setting of new HIV diagnoses in an academic medical centre. Increasing new diagnoses among MSM may reflect local and city-wide public health campaigns focused on HIV testing and pre-exposure prophylaxis awareness. Also notable are individuals aged ≥50 years who continue to be diagnosed with advanced disease. This group needs to be a focus of HIV prevention and testing campaigns.

# Sub article: P338 Reasons for transferring HIV care in London

**Published:** 2016-10-23

**Authors:** Nadia Ahmed, Duncan Scott, Laura Waters, Nashaba Matin, Gary Whitlock

**Affiliations:** 1 HIV/GU, Mortimer Market Centre, London, UK; 2 HIV, Barts Health NHS Trust, London, UK; 3 HIV/GU Medicine, 56 Dean Street, London, UK

## Abstract

**Introduction:** In England, people living with HIV (PLHIV) can access care at any centre, regardless of geographical location. Non-UK born and individuals without residency are also entitled to free HIV care at any service. There are no data currently available on reasons patients transfer their HIV management and care from one service to another. We aimed to investigate reasons for transfer amongst PLHIV transferring their care to one of three London HIV units in London, UK.

**Materials and methods:** Patients transferring their HIV care to one of three London clinics between December 2015 and June 2016 were asked to fill in a questionnaire. The questionnaire was designed to explore reasons for leaving their previous centre and reasons for choosing the new service.

**Results:** A total of 111 patients completed the questionnaire; 47% (n=52) transferred from services abroad, 37% (n=41) within London and 16% (n=18) transferred from outside of London. Reasons for leaving the previous HIV clinic included location (75%, n=83), problems at the clinic (10%, n=11) and confidentiality (5%, n=5). Other reasons (n=12) included services offered (e.g. specialist services for HCV treatment), finance and employment. Reasons for choosing the service patients transferred to included location (31%, n=34), good reputation (20%, n=22), friend/partner attending the service (14%, n=6). A total of 21% (n=22) gave a combination of these reasons and 15% (n=17) gave other reasons including previously attending the service, recommendation by a doctor. Only one patient mentioned using the internet to find their clinic. Current BHIVA guidelines recommend a medical summary should be received within 2 weeks of transferring to a new service. And 27% (26 of 95) of patients were aware of the summary being received at the time of their first appointment of whom 11/26 had transferred their care within the United Kingdom; 36 stated it had not been received and 33 did not know.

**Conclusions:** Most patients transferred their care to another HIV service for geographical reasons. Reasons for choosing their new clinic included a combination of location, reputation or a friend/partner already attending the service. Reassuringly, a minority cited problems at their old clinic as a reason to transfer care. However, this could have been due to sampling bias, patients with problems may have been less likely to complete the questionnaire. In the age of digital media, it is also interesting that only one patient found their chosen clinic via the internet. Patients seem to base their choice on recommendation.

# Sub article: P339 Atmosphere of risk or family-like support? Alternative patient experiences of decentralized care in North Central Nigeria

**Published:** 2016-10-23

**Authors:** Grace Kolawole, Hannah Gilbert, Nancin Dadem, Patricia Agaba, Becky Genberg, Oche Agbaji, Prosper Okonkwo, Norma Ware

**Affiliations:** 1 AIDS Prevention Initiative in Nigeria, Jos University Teaching Hospital, Jos, Nigeria; 2 Global Health and Social Medicine, Harvard Medical School, Boston, MA, USA; 3 Family Medicine, Jos University Teaching Hospital, Jos, Nigeria; 4 Health Services, Brown University, Providence, RI, USA; 5 Internal Medicine, Jos University Teaching Hospital, Jos, Nigeria; 6 AIDS Prevention Initiative in Nigeria, Abuja, Nigeria

## Abstract

**Introduction:** Decentralization of HIV care and treatment has played a critical role in scaling up services across sub-Saharan Africa [1,2]. However, little is understood about the implications for people living with HIV (PLHIV) in having care closer to their communities. This qualitative study examined patient experiences of challenges and advantages of receiving care at decentralized clinics.

**Materials and methods:** Four decentralized clinics in small community hospitals in Plateau State, North Central Nigeria, served as study sites. In total, 39 patients took part in individual open-ended interviews; 23 participated in four focus groups. All participants had transferred from a large, urban HIV clinic. Interview topics addressed access to and preferences for care, services received, perceived impact of decentralization and experiences of decentralization. Resulting data were analyzed to identify recurrent themes and develop descriptive categories.

**Results:** Receiving care at clinics located in local communities shapes the experience of care for patients. Because decentralized sites have fewer HIV patients, HIV clinics take place on specific days of the week. This creates a situation of predictable clinic attendance for PLHIV that can alternately lead to unwanted disclosure of HIV status or promote a “family-like” atmosphere of support within the clinic. Underlying factors determine whether a decentralized HIV clinic creates an atmosphere of risk or family-like support. These include the following: physical layout of the clinic, whether “ground rules” for confidentiality are established and enforced by staff and whether staff foster social interaction among patients by offering patient-centred care and organizing activities such as group meetings and positive living discussions.

**Conclusion**: Decentralized clinics embedded within communities can pose the risk of unwanted disclosure. However, with patient-specific provider management, clinics can use local positioning to promote family-like relationships. These relationships may positively impact patient interpretations of quality of care, thereby improving retention rates in decentralized clinics.

**Funding:** US National Institute of Mental Health (K24MH090894, NC Ware, PI)

## References

[1] Boyer S, Eboko F, Camara M, Abe C, Nguini ME, Koulla-Shiro S (2010). Scaling up access to antiretroviral treatment for HIV infection: the impact of decentralization of healthcare delivery in Cameroon. AIDS.

[2] Chan AK, Mateyu G, Jahn A, Schouten E, Arora P, Mlotha W (2010). Outcome assessment of decentralization of antiretroviral therapy provision in a rural district of Malawi using an integrated primary care model. Trop Med Int Health.

# Sub article: P340 High mortality attributable to late presentation and delayed ART initiation in HIV-infected adults receiving care in Latin America

**Published:** 2016-10-23

**Authors:** Pablo Belaunzarán-Zamudio, Bryan Shepherd, Yanink Caro-Vega, Claudia Cortés, Brenda Crabtree-Ramírez, Beatriz Grinsztejn, Eduardo Gotuzzo, Fernando Mejía, Denis Padgett, Jean Pape, Peter Rebeiro, Vanessa Rouzier, Valdilea Veloso, Sandra Wagner-Cardoso, Catherine McGowan, Juan Sierra-Madero

**Affiliations:** 1 Departamento de Infectología, Instituto Nacional de Ciencias Médicas y Nutrición Salvador Zubirán, Mexico; 2 Department of Biostatistics, Vanderbilt University, Nashville, TN, USA; 3 Fundación Arriarán, Universidad de Chile, Santiago, Chile; 4 Fundacão Oswaldo Cruz, Instituto de Pesquisa Clínica Evandro Chagas, Rio de Janeiro, Brazil; 5 Instituto de Medicina Tropical Alexander von Humboldt, Lima, Peru; 6 Instituto Hondureño de Seguridad Social and Hospital Escuela, Tegucigalpa, Honduras; 7 Le Groupe Haitien d'Etude du Sarcome de Kaposi et des Infections Opportunistes, Port-au-Prince, Haiti; 8 Department of Medicine, Vanderbilt University, Nashville, TN, USA

## Abstract

**Introduction**: Late presentation and late ART initiation for HIV infection is common in Latin America. Here, we estimated the proportion of deaths among HIV-infected adults receiving care at CCASAnet sites that could be attributed to late presentation (LP) to care, late ART initiation (LI) and no-ART initiation (NI) to highlight the potential impact of implementing strategies that reduce the time between HIV infection and diagnosis, linkage to care, and ART initiation.

**Methods**: In this observational, multicentre, cohort study including adults enrolled at six centres in Latin America from 2001 to 2014, we estimated the population attributable fraction (PAF) for mortality due to LP, LI and NI. LP was defined as CD4 <200 cells/µL or AIDS at clinic enrolment. LI was defined as failing to start ART before CD4 <200 cells/µL or AIDS. The primary endpoint was all-cause mortality. We compared mortality in LP versus non-LP; LI versus non-LI among non-LP; and ART initiation versus NI among LP. We used weighted Cox regression and marginal structural models to estimate survival probabilities used in calculating the PAF.

**Results**: Of 9,229 patients, 5,162 (56%) were LP (Figure 1). Median CD4 at enrolment was 198 cells/µL (IQR, 68–381); 32% had an AIDS defining illness. Survival probability at 10 years was 84% (95% CI=82%, 86%) for LP and 93% (95% CI=92–94%) for non-LP. The PAF of mortality for LP was 78% (95% CI=70–86%), 58% (95% CI=49%, 67%), and 43% (95%CI=33%, 54%) at 1, 5 and 10 years after enrolment, respectively, meaning 78% of deaths during the first year after enrolment would have been prevented if individuals had been non-LP (Figure 2). Of 4,067 patients who were non-LP, 77% started ART after a median of 8 months. The proportion of deaths among these non-LPs that would have been prevented by initiating ART before CD4 <200 or AIDS was 46% (95% CI=0–77%), 55% (95% CI=0–86%), and 47% (95% CI=10–80%) at 1, 5 and 10 years after enrolment (Figure 3). Among LP, starting ART decreased the hazard of death by 63% (95% CI=43–72%). However, 93% of LP started ART, so universal and immediate initiation of ART among LP would only result in an estimated 12% (95% CI=6–26%) decrease in mortality after 1 year.

**Conclusion**: Earlier presentation to care and earlier initiation of ART would substantially reduce mortality among HIV-infected subjects in Latin America, mainly during the first year after enrolment. Interventions to improve early diagnosis and linkage to care are particularly needed.

**Figure 1:** Progression of all HIV-infected patients included in the analysis (N = 9,229) from status at enrolment (late presentation *vs*. non-late presentation) to early (non-LI) versus late antiretroviral therapy initiation (LI) among late presenters, and ART initiation (non-NI) versus no ART initiation (NI) among late presenters and total deaths by treatment group.

**Figure 2:** Attributable risk of death (left) and population attributable fraction (right) of death due to late presentation (CD4 < 200 or AIDS) over time (and 95% confidence intervals).

**Figure 3:** Estimated survival probabilities and 95% confidence intervals for patients entering care in not advanced stages of disease (non-LP) and starting ART before progression to AIDS in comparison to those who started after advancing to late stages of disease.

# Sub article: P341 Insights into missed opportunities for earlier testing in newly diagnosed patients referred for HIV care to a Swiss teaching hospital between 2010 and 2015

**Published:** 2016-10-23

**Authors:** Loïc Lhopitallier, Estelle Moulin, Matthias Cavassini, Katharine Darling

**Affiliations:** 1 Infectious Diseases, Centre Hospitalier Vaudois, Lausanne, Switzerland

## Abstract

**Introduction:** Missed opportunities (MOs) for HIV testing occur when a patient with undiagnosed HIV infection presents to a healthcare provider and is not offered an HIV test. Some late presenters (LPs), defined as patients first presenting for care with a CD4 count below 350 cells/mm^3^, have presented several MOs before an HIV test is performed. The aim of this study was to examine the characteristics of newly diagnosed patients presenting for care to our clinic and the extent to which MOs for HIV testing occur in our hospital.

**Materials and methods:** Medical records of all patients newly presenting to our infectious diseases outpatient clinic for HIV care between 2010 and 2015 were examined. Demographic characteristics, HIV stage at diagnosis and reasons for HIV testing were recorded. For each patient, inpatient and outpatient visits to our teaching hospital during the 5 years preceding the HIV diagnosis were reviewed to determine whether HIV testing had been indicated according to the 2015 Swiss HIV testing recommendations. MOs were defined as visits at which HIV testing was indicated but not performed.

**Results:** In total, 201 patients were included. Patient characteristics are summarized in Table 1. A total of 106 patients (53%) were late presenters, and94 patients (47%) had presented at least one MO (range 1–17) at our teaching hospital during the 5 years preceding diagnosis. Figure 1 shows the distribution of the type of MOs. Multivariate analysis revealed that MOs occurred more frequently among patients from sub-Saharan Africa (SSA) (aOR 3.5, 95% CI 1.4–8.6), men who have sex with men (MSM) (aOR 3.3, 95% CI 1.2–9.4) and patients with chronic disease (aOR 4.5, 95% CI 1.8–11.1). In multivariate analysis, MOs were not associated with increased risk of late presentation (aOR 0.6, 95% CI 0.3–1.4). Median CD4 count (cells/mm^3^) at HIV diagnosis was significantly higher among patients presenting at least one MO (351 vs. 244, p<0.01).

**Conclusions:** Almost half our patients presented at least one MO before HIV diagnosis. The increased MO frequency among patients from SSA and MSM suggests that rates of HIV testing should be improved in key groups at higher risk of HIV acquisition. LPs had fewer MOs than non-LPs. As our data on MOs relate to hospital visits, it remains to be determined whether LPs in our population have reduced access to healthcare if they present MOs in the primary care sector.

**Figure 1:** Distribution of the type of MOs in our study population.

**Table 1:** Patient demographic characteristics and mode of HIV acquisition

	Number of patients (%) without MOs	Number of patients (%) with MOs
**Age (years)**		
18–29	23 (41)	33 (59)
30–49	59 (53)	53 (47)
>50	25 (76)	8 (24)
**Sex**		
Female	41 (54)	34 (46)
Male	66 (52)	60 (48)
**Geographical origin**		
Europe, North America, Australasia	58 (55)	48 (45)
Sub-Saharan Africa	32 (48)	34 (52)
Other	17 (59)	12 (41)
**Mode of HIV acquisition**		
Heterosexual	67 (59)	47 (41)
Men who have sex with men (MSM)	29 (43)	39 (57)
Injecting drug users (IVDU)	3 (33)	6 (67)
Other	8 (80)	2 (20)
**Time since previous HIV test**		
None	72 (61)	47 (39)
≤1 year	12 (43)	16 (57)
>1 year ago	23 (43)	31 (57)

# Sub article: P342 Is it acceptable to ignore national testing guidelines? Current testing practice in Lothian 8 years after BHIVA national testing guidelines were published

**Published:** 2016-10-23

**Authors:** Muge Cevik, Durba Raha, Callum Mutch, Clifford Leen

**Affiliations:** 1 Infectious Diseases, Western General Hospital, Edinburgh, UK; 2 GUM, Chalmers Sexual Health Centre, Edinburgh, UK

## Abstract

**Introduction**: In the United Kingdom, almost 90% of patients diagnosed with HIV had initiated ART, with 93% of those on ART having a suppressed viral load. However, approximately one-third of all HIV infections in adults still remain undiagnosed and one-fourth of newly diagnosed individuals are late presenters. We aim to assess the newly diagnosed individuals in Lothian where prevalence is over 0.2% with no agreed policy to screen for HIV in order to understand current testing practice.

**Methods**: Using our dedicated HIV database, we included all new presenters to our services between April 2015 and April 2016. Data were retrospectively collated through electronic patient records. Descriptive statistics were performed to examine demographics, baseline characteristics and treatments.

**Results**: We identified 51 individuals (3 females) who were newly diagnosed with HIV. Median age was 35 years (20–73) with three individuals >65 years. A total of 17 individuals were diagnosed in GUM clinic, 14 in GP practice, 10 in secondary care, 6 through outreach services and 3 using self-testing kit. A total of 21 individuals diagnosed through routine screening, 5 contact tracing and 24 individuals presented with clinical symptoms. Median nadir CD4 cell count was 326 cells/mL. However, we observed significant differences in those diagnosed in various settings (Table 1).

Individuals diagnosed in secondary care and those who used home testing kit had significantly lower median CD4 counts, 81 and 114 cells/mL, respectively. More than half of our cohort (28) had CD4 count <350 cells/mL, 10 (36%) of those presented with AIDS-defining illness and 11 individuals (39%) presented to a healthcare facility in the past year before the diagnosis with a clinical indicator of HIV infection. Overall 13 (25%) individuals had CD4 <200 cells/mL, 4 (8%) out of those died within 3 months after diagnosis due to an AIDS-defining illness.

**Conclusion**: Early diagnosis is critical to improve morbidity and mortality, and also for the prevention of onward transmission. Late diagnosis has been associated with longer hospital stay and increased cost to healthcare services. In this cohort, one-fourth of patients had CD4 count <200 cells/mL and more than half had CD4 <350 cells/mL. In total, 39% of late presenters had contact with healthcare prior to HIV diagnosis representing missed opportunities for earlier diagnosis. We believe routine testing in all relevant settings should be offered as per national guidelines in order to reduce undiagnosed HIV infection at late diagnosis.

**Abstract P342–Table 1:** Demographics and baseline characteristics of individuals diagnosed with HIV in various settings

	Outreach	GUM	GP	Home testing	Secondary care	Total
Median age (years)	35 (27–47)	28 (22–50)	36 (25–73)	31 (26–46)	45 (20–64)	35 (20–73)
MSM:HSM	5:1	17:0	9:2	3:0	11:0	45:3
Female	0	0	3	0	0	3
Median CD4 count (cells/mL)	490 (275–659)	386 (234–1166)	385 (40–885)	114 (68–659)	81 (6–570)	326 (6–1166)
CD4 count <350 (n)	2	8	7	3	8	28 (55%)
CD4 count <200 (n)	0	0	2	3	8	13 (25%)
Survival at 1 year	100%	100%	100%	100%	63%	92%
Number/percentage	6 (12%)	17 (33%)	14 (27%)	3 (6%)	11 (22%)	51 (100%)

# Sub article: P343 HIV late presenters: a retrospective cohort study on an outpatient clinic in Lisbon, 2010–2014

**Published:** 2016-10-23

**Authors:** Fábio Cota-Medeiros, Cláudia Afonso, Alexandra Zagalo, Luís Caldeira

**Affiliations:** 1 Infectious Diseases, Centro Hospitalar Lisboa Norte – Hospital Santa Maria, Lisbon, Portugal

## Abstract

**Introduction:** The diagnosis of HIV late presenter is associated with a worse clinical condition, increased rate of HIV transmission and higher healthcare cost burden. Recognition of this population could change this outcome. Our aim was to analyze the characteristics, factors and risk predictors associated with being late presenter among newly HIV-infected patients in an outpatient clinic in Lisbon during a 5-year period.

**Materials and methods:** Retrospective analysis of the clinical records of patients older than 18 years, newly diagnosed with HIV infection admitted to our outpatient clinic between January 2010 and December 2014. Epidemiologic, immunologic, clinical and laboratory data were recorded. European Late Presenter Consensus definition criteria for late presentation [1] were used.

**Results:** A total of 347 newly HIV-infected patients were admitted to our outpatient clinic, 149 (42.9%) meet the criteria for late presenters (LP) and 88 (25.4%) were late presenters with advanced disease (LPWAD). The majority of LP were male (68.5%), with a median age of 41 (62.5% were aged between 31 and 50 years), Caucasians (71.1%) and 66.4% were Portuguese. Heterosexual transmission was the main route of infection (57.7%). The main trigger of HIV infection diagnosis on the LP group was clinical investigation due to symptoms (55%) followed in 18.1% by screening due to behaviour risk mainly in the MSM and IVDU groups. A total of 52 (34.9%) patients were asymptomatic and 95 (63.8%) fulfilled AIDS criteria according to the CDC classification. The median CD4 count was 195 cells/mm^3^ (4.2–724 cells/mm^3^); the median viral load was 159,200 copies/mL (303–7,578,000 copies/mL) and 55.7% patients had viral load higher than 5 log_10_. Almost half of the new HIV diagnosed patients were foreign (48.5%) and 53.2% of the patients of African origin were LP. There was a higher number of heterosexual males who were LP than men who have sex with men (49.1% vs. 30.3%). Although only 4.6% of newly diagnosed patients were IVDU, 75% of these were LP.

**Conclusions:** There was a high percentage of late diagnosis of HIV infection in our cohort. These results emphasize the need to promote a better access to care not only to the classic behavioural risk groups like MSM and IVDU but mainly to the foreign population and heterosexual males.

## References

[1] Antinori A, Coenen T, Costagiola D, Dedes N, Ellefson M, Gatell J (2011). Late presentation of HIV infection: a consensus definition. HIV Med.

# Sub article: P344 Clinical characteristics of newly diagnosed HIV-infected patients and risk factors for late presentation: a Portuguese cohort

**Published:** 2016-10-23

**Authors:** Rita Veiga Ferraz, Francisco Almeida, Raquel Duro, Nuno Pereira, Carmela Pinero, Cátia Caldas, Rosário Serrão, António Sarmento

**Affiliations:** 1 Infectious Diseases, Centro Hospitalar de São João, Porto, Portugal

## Abstract

**Introduction**: The epidemiology of HIV infection changed dramatically in the last few years; however, late diagnosis, associated with increased disease burden and risk of transmission as well as a reduction of the benefits of antiretroviral therapy, continues to be a major issue. The main objectives of this study were to identify risk factors related to late presentation and describe the evolution of clinical characteristics of newly diagnosed HIV-infected patients.

**Methods**: Retrospective observational cohort study of all newly diagnosed HIV-infected patients admitted to our Infectious Diseases Department between 2006 and 2015. Data were collected after chart review. For a temporal trend analysis patients were categorized into five time periods: 2006 to 2007; 2008 to 2009; 2010 to 2011; 2012 to 2013; 2014 to 2015. Continuous variables were expressed as median and standard deviation, and categorical variables were expressed as number (percentage). Patient characteristics were compared using chi-square test, independent samples t-test or ANOVA, as appropriate. Risk factors for immunity depression were initially investigated through univariate analysis; with the factors identified, a multiple logistic regression model was performed. The level of significance considered was p<0.05. Analyses were carried out using SPSS version 22.0.

**Results**: We identified 972 newly diagnosed HIV-infected people between 2006 and 2015. The majority of the patients were male (73.6%) and the mean age was 41±13 years. The main mode of transmission was unprotected heterosexual sex (62.0%). Late presenters represented more than half of the patients (58.3%) and 41.8% already had an opportunistic infection when first observed in HIV clinic. Clinical characteristics and its temporal analysis are summarized in Table 1.

In univariate analysis, the risk factors for late presentation identified were older age, risk of acquisition, site of referral to HIV clinic and previous opportunistic infection. In multivariate analysis, the risk factors identified were age (OR 1.021; 95% CI 1.009–1.034) and referral from infectious diseases ward/emergency room/other hospital wards/other hospitals.

**Conclusion**: This study reveals that despite the changing epidemiology of HIV infection late presentation is still a major issue in HIV patients. These data put in question the efficacy of campaigns targeting specific groups to improve early diagnosis and raise the question of universal testing of HIV infection.

**Table 1:** Clinical characteristics of newly diagnosed HIV patients: temporal trend analysis

Clinical characteristics	Total	2006–2007 (n = 226)	2008–2009 (n = 239)	2010–2011 (n = 182)	2012–2013 (n = 179)	2014–2015 (n = 146)	p
Gender							
Male	715 (73.6%)	164 (72.6%)	158 (66.1%)	138 (75.8%)	133 (74.3%)	122 (83.6%)	0.005
Female	257 (26.4%)	62 (27.4%)	81 (33.9%)	44 (24.2%)	46 (25.7%)	24 (16.4%)	
Age (mean±SD)	40.78 (13.4)	40.90 (13.3)	42.48 (14.3)	40.74 (13.9)	39.56 (12.3)	39.19 (12.6)	0.101
Risk for HIV acquisition (MSM, heterosexual, IDU, other)	284 (29.2%) 603 (62%) 70 (7.2%) 15 (1.5%)	41 (18.1%) 146 (64.6%) 39 (17.3) 0	51 (21.3%) 173 (72.4%) 9 (3.8%) 6 (2.5%)	53 (29.1%) 118 (64.8%) 11 (6.0%) 0	76 (42.5%) 95 (53.1%) 7 (3.9%) 1 (0.6%)	63 (43.2%) 71 (48.6%) 4 (2.7%) 8 (5.5%)	<0.001
Site of referral	374 (38.5%)	88 (38.9%)	89 (37.2%)	72 (39.6%)	65 (36.3%)	60 (41.1%)	<0.001
ID ward/ER/other Ambulatory Serodiscordant couple/drug centre/jails Voluntary testing Blood donation/pregnancy Unknown	344 (35.4%) 90 (9.3%) 110 (11.3%) 39 (4.0%) 15 (1.5%)	72 (31.9%) 41 (18.1%) 13 (5.8%) 5 (2.2%) 7 (3.1%)	87 (36.4%) 27 (11.3%) 24 (10%) 12 (5%) 0	74 (40.7%) 13 (7.1%) 19 (10.4%) 4 (2.2%) 0	65 (36.3) 5 (2.8%) 34 (19%) 9 (5%) 1 (0.6%)	46 (31.5%) 4 (2.7%) 20 (13.7%) 9 (6.2%) 7 (4.8%)	
CD4 cell count (mean±SD)	322 (252.0)	342.33 (305.1)	306.82 (269.8)	308.33 (225.2)	306.10 (199.7)	355.18 (258.5)	0.208
CD4 cell count categorized <350 >350	567 (58.3%) 405 (41.7%)	132 (58.4%) 94 (41.6%)	150 (62.8%) 89 (37.2%)	102 (56%) 80 (44%)	109 (60.9%) 70 (39.1%)	74 (50.7%) 72 (49.3%)	0.177
Clinical stageAIDSNon-AIDS	406 (41.8%) 566 (58.2%)	100 (44.2%) 126 (55.8%)	118 (49.4%) 121 (50.6%)	72 (39.6%) 110 (60.4%)	64 (35.8%) 115 (64.2%)	52 (35.2%) 94 (64.4%)	0.021

# Sub article: P345 Immune recovery in acute and chronic HIV infection and the impact of thymic stromal lymphopoietin

**Published:** 2016-10-23

**Authors:** Marco Gelpi, Hans Jakob Hartling, Kristina Thorsteinsson, Jan Gerstoft, Henrik Ullum, Susanne Dam Nielsen

**Affiliations:** 1 Infectious Diseases, Rigshospitalet, Copenhagen, Denmark; 2 Infectious Diseases, Hvidovre Hospital, Hvidovre, Denmark; 3 Clinical Immunology, Rigshospitalet, Copenhagen, Denmark

## Abstract

**Introduction**: Symptomatic primary HIV infection is associated with faster decline in CD4+ T cells count and progression to AIDS, and immediate initiation of combination antiretroviral therapy (cART) is recommended. However, little is known about immunological predictors of immune recovery. Thymic stromal lymphopoietin (TSLP) is a cytokine that promotes homeostatic polyclonal proliferation of CD4+ T cells and participates in regulating Th17/regulatory T-cell balance, immunological functions known to be affected during primary HIV infection. The aim of this study was to describe immune recovery in primary and chronic HIV infection and possible impact of TSLP.

**Materials and methods**: Prospective study including 100 HIV-infected individuals (primary HIV infection (N=14), early presenters (>350 CD4+ T cells/µL, N=42), late presenters without advanced disease (200–350 CD4+ T cells/µL, N=24) and late presenters with advanced disease (<200 CD4+ T cells/µL, N=20). Plasma TSLP was determined using ELISA and CD4+ T cell subpopulations (recent thymic emigrants, naïve and memory cells) were measured using flow cytometry at baseline and after 6, 12 and 24 months of cART.

**Results**: Immune recovery was comparable in all groups, and no differences in immune homeostasis were found between primary HIV infection and early presenters (Figure 1b). In primary HIV infection group, lower thymic output compared to late presenters without advanced disease was found. However, lower proportion of effector memory and higher proportion of late differentiated CD4+ T cell were found in primary HIV infection compared to late presenters. TSLP was elevated in primary HIV infection at baseline and after 24 months of cART (Figure 1c and Table 1). Interestingly, TSLP was negatively associated with proportion of recent thymic emigrants (correlation coefficient −0.60, p=0.030). However, TSLP was not associated with immune recovery in primary HIV infection. Finally, higher plasma TSLP was associated with lower CD4+ T cell recovery in the late presenters non-advanced disease group (correlation coefficient −0.50, p=0.034).

**Conclusions**: Immune recovery was comparable in primary and chronic HIV infection whereas differences in absolute counts and proportions of CD4+ T cell subpopulations were found between primary HIV infection and late presenters supporting early initiation of cART. Higher plasma TSLP was found in primary HIV infection. Association between TSLP and a lower thymic output, but not with immune recovery was found in primary HIV infection. These findings indicate a possible role of TSLP in immune homeostasis in HIV infection but do not support TSLP to affect immune recovery in primary HIV infection.

**Figure 1:** CD4 count (a), immune recovery (b) and plasma TSLP (c) before cART and during 24 months of follow-up. Mann–Whitney was used to compare PHI group with the chronic groups. Significant differences are marked: ^a^PHI versus late presenters [s1]with advanced disease; ^b^PHI versus late presenters without advanced disease; ^c^PHI versus early presenters.

**Abstract P345–Table 1:** Proportion of CD4+ T cell subsets in HIV-infected individuals with either primary HIV infection or chronic HIV infection according to CD4+ T cell count before initiation of cART

	PHI, N = 14	LP-AD <200 cells/µL, N = 20	LP-nonAD 200-350 cells/µL, N = 24	EP >350 cells/µL, N = 42
Gender, males/females (% males)	13/1 (92.9)	18/2 (90.0)	21/3 (87.5)	39/3 (92.9)
Age, years, median (IQR)	47 (12)	42 (16)	38 (16)	44.5 (12)
Time since diagnosis, days, median (IQR)	2 (3)	3 (9)	18 (269)	24 (983)
CD4+ nadir, cells/µL, median (IQR)	540 (335)	45 (113)	290 (95)	480 (170)
CD4+ at baseline, cells/µL, median (IQR)	550 (327)	55 (110)	290 (97)	510 (172)
CD4+ at 24 months of cART, cells/µL, median (IQR)	680 (240)^a^	269 (160)^a^	695 (290)	820 (317)
RTE at baseline, %, median (IQR)	14 (11)	11 (16)	20 (15)	18 (14)
RTE at 24 months of cART, %, median (IQR)	18 (9)^b^	17 (10)	28 (11)^b^	17 (16)
Naive at baseline, %, median (IQR)	43 (20)^a^	23 (30)^a^	40 (26)	44 (21)
Naive at 24 months of cART, %, median (IQR)	36 (12)^b^	30 (16)	55 (16)^b^	37 (15)
EM at baseline, %, median (IQR)	12 (7)	17 (14)	16 (12)	12 (6)
EM at 24 months of cART, %, median (IQR)	9 (4)^a^	15 (6)^a^	6 (7)	7 (5)
CM at baseline, %, median (IQR)	26 (6)	20 (22)	24 (10)	24 (10)
CM at 24 months of cART, %, median (IQR)	24 (14)	30 (23)	24 (8)	32 (16)
LD at baseline, %, median (IQR)	5 (4)^b^	3 (12)	1 (2)^b^	5 (8)
LD at 24 months of cART, %, median (IQR)	9 (16)^a^,^b^	1 (1)^a^	3 (2)^b^	9 (13)
TSLP at baseline, pg/mL, median (IQR)	2.8 (2.3)^a^,^b^,^c^	1.7 (0.8)^a^	2.1 (1.4)^b^	1.9 (0.7)^c^
TSLP at 24 months of cART, pg/mL, median (IQR)	3.9 (2.8)^a^,^c^	1.3 (1.4)^a^	2.4 (2.9)	2.1 (1.0)^c^

Abbreviations: LP-AD, late presenters with advanced disease; LP-nonAD, late presenters without advanced disease; EP, early presenters; PHI, primary HIV infection. Comparing the four HIV groups by using Kruskal–Wallis test. If significant (<0.05) then Mann–Whitney was used to compare PHI group with the other chronic groups. Only significant differences are marked

a PHI versus LP-AD

b PHI versus LP-nonAD

c PHI versus EP.

# Sub article: P346 Neuroasymptomatic cerebrospinal fluid (CSF) viral escape (aCVE) is associated with increased intrathecal immune activation but not with CSF signs of neuronal injury and alteration of CSF/serum albumin ratio

**Published:** 2016-10-23

**Authors:** Carmela Pinnetti, Valentina Fedele, Stefania Carta, Patrizia Lorenzini, Valentina Mazzotta, Veronica Bordoni, Francesco Baldini, Susanna Grisetti, Maria Rosaria Capobianchi, Federico Martini, Francesca Ceccherini-Silberstein, Adriana Ammassari, Carlo Federico Perno, Andrea Antinori

**Affiliations:** 1 Clinical Department, National Institute for Infectious Diseases, Rome, Italy; 2 Antiretroviral Drug Monitoring Unit, National Institute for Infectious Diseases, Rome, Italy; 3 Laboratory of Virology, National Institute for Infectious Diseases, Rome, Italy; 4 Laboratory of Immunology, National Institute for Infectious Diseases, Rome, Italy; 5 Experimental Medicine and Surgery, University of Rome Tor Vergata, Rome, Italy

## Abstract

**Introduction:** aCVE is of rising interest in the HIV setting, although preliminary data described it as an uncommon finding. CSF viral escape (CVE) predictors are not definitively assessed. Pathogenesis and clinical significance of aCVE remain uncertain, particularly regarding intrathecal immune activation/inflammation and neuroinjury markers during aCVE.

**Materials and methods:** Single-center retrospective study on CSF/plasma paired samples collected on neurologically asymptomatic HIV-positive patients undergoing lumbar puncture (LP) for CNS staging of lymphoma during ART exposure. aCVE was defined: a) detectable CSF HIV RNA with concurrent plasma levels <50 copies/mL, or b) CSF HIV RNA >1.0 log higher than concomitant plasma HIV RNA level. CSF neopterin, and neurofilament light-chain (NFL) concentrations were determined by ELISA assays. aCVE adjusted ORs were calculated by fitting a logistic multivariate regression model.

**Results:** Two hundred and ninety-one CSF/plasma pairs from 92 patients were included: 88% male, median age 42 years, heterosexual 47%, MSM 26%, IDU 21%. CD4 cells/mm^3^ was <200 in 48% and >500 in 22%; 98% CDC stage C. CSF was collected in 44% during 2004 to 2008, in 56% during 2009 to 2014. At LP, all patients were receiving cART: 67.7% TDF/FTC, 11% ABC/3TC, 2.8% ZDV/3TC, 40% EFV, 33% LPV/r, 9% ATV/r and 6.5% DRV/r. Hundred and forty-nine (51.2%) had HIV RNA <50 copies/mL, 206 (70.8%) <200 copies/mL. CVE was detected in 24/291 samples (8.2%): 62.5% diagnosed with criterion a); 37.5% with criterion b). Mean CSF HIV RNA log was 1.95. At multivariable analysis, male gender (OR 0.20; 95% CI 0.04–0.90 vs. female), CD4 >350 (0.11; 0.02–0.82 vs. CD4 <200) and 2009 to 2014 period (0.10; 0.03–0.38 vs. 2004–2008) were all independently associated with a decreased risk of CVE. Using TDF/FTC as reference, receiving ABC/3TC at LP (OR 4.38; 1.13–16.96) was independently related to an increased CVE risk. Age, nadir CD4 <200, duration of cART, 2010 CSF CNS penetration effectiveness score, third ARV drug received, and CSF/serum albumin ratio were not associated with CVE. Patients with CVE showed a higher concentration of CSF neopterin (p=0.015) comparing to patients without, while no significant difference for CSF NFL level (Figure 1). No difference was found in CSF/plasma albumin ratio.

**Conclusions:** In 291 CSF/plasma paired samples obtained from HIV-positive asymptomatic patients on cART, CVE occurred in 8.2% of cases. Higher CD4 at LP was associated with a lower CVE probability, while use of ABC/3TC is associated with increased risk of CVE, but this finding needs further investigation. An increased intrathecal immune activation, but not neuroinjury, was found, suggesting that an inflammatory response concomitant with aCVE does not immediately translate into ongoing axonal injury and BBB damage.

**Abstract P346–Figure 1:** Cerebrospinal fluid (CSF) neurofilament light protein (NFL) and neopterin concentration by the presence of an asymptomatic CSF viral escape (CVE).

# Sub article: P347 Cardiovascular risk in HIV-positive subjects: analyses of T-cell phenotype and CD49d expression

**Published:** 2016-10-23

**Authors:** Maria Antonella Zingaropoli, Alessandra D'Abramo, Marco Iannetta, Alessandra Oliva, Gabriella d'Ettorre, Miriam Lichtner, Claudio Maria Mastroianni, Maria Rosa Ciardi, Vincenzo Vullo

**Affiliations:** 1 Department of Public Health and Infectious Diseases, Sapienza University of Rome, Rome, Italy

## Abstract

**Introduction:** It is well known that HIV-positive subjects have a higher risk of non-AIDS-related comorbidities than general population. Chronic immune activation of T-cells plays an important role in HIV pathogenesis and related comorbidities. In this context, the integrin-alpha4 (CD49d), a transmembrane co-stimulatory molecule, is involved in the lymphocyte homing from peripheral compartment to the gut (alpha4beta7) and to the central nervous system (alpha4beta1). The aim of the study was to evaluate CD49d expression in T-lymphocyte subsets and the relationship with cardiovascular damage in HIV+ individuals on effective cART.

**Materials and methods:** Thirty HIV+ subjects (6 females and 24 males) with a mean age (±standard deviation (SD)) of 52±10.1 years on effective cART and 15 age- and sex-matched healthy donors (HD) were enroled. T-lymphocyte immunophenotype and CD49d expression, measured as median fluorescence intensity (MFI), were assessed by flow cytometry. Carotid intima-media thickness (c-IMT) was measured with ultrasonography. Normal and pathological c-IMT were defined as IMT <0.9 mm and >0.9 mm, respectively.

**Results:** HIV+ subjects showed a lower count of CD4+ T-lymphocytes (p=0.04) and increased levels of immune activation (CD4+ and CD8+ HLA-DR+CD38+, p<0.001 and p<0.001, respectively) and immunosenescence (CD4+ and CD8+ CD28–CD57+, p=0.02 and p<0.001, respectively) than HD. A decrease in CD4+ and CD8+ naïve (N) (p=0.02 and p=0.01) and an increase in CD8+ effector memory (EM) (p=0.007) percentages were observed in HIV+ subjects, compared to HD. In HIV+ subjects, CD49d expression was increased on CD4+ (N: p=0.01; central memory (CM): p<0.001; EM: p<0.001; effector (E): p=0.05) and CD8+ (N: p=0.0006; CM: p<0.001; EM: p<0.001; E: p=0.003; and intermediate (I): p<0.001) T-lymphocyte subsets, compared to HD. A positive correlation between CD49d expression in CD4+ T-cells and CD4+ HLA-DR+CD38+ (p=0.0012) was observed in HIV+ subjects. c-IMT was higher in the HIV+ group than HD (mean±SD: 0.85±0.17 vs. 0.28±0.24 mm, p<0.001). Among HIV+ patients, 15 (50%) had a normal c-IMT and 15 (50%) a pathological c-IMT. CD4+T-cell CD49d expression and CD4+HLA-DR+CD38+ positively correlated with c-IMT (p=0.04, p=0.085, respectively). Moreover, HIV+ subjects with pathological c-IMT showed higher levels of CD4+CM CD49d expression (p=0.02) than HIV+ subjects with normal c-IMT.

**Conclusions:** The increase of CD49d expression in T-lymphocytes could be considered an early marker of immune activation during HIV infection. Furthermore, integrin-alpha4 could represent a potential therapeutic target for the immune system modulation in the context of HIV infection aiming to reduce non-AIDS-related comorbidities, especially cardiovascular diseases.

# Sub article: P348 Baseline myeloid and lymphoid activation markers can predict time to viral load reduction under 50 copies/mL and CD4 recovery, respectively, after highly active antiretroviral therapy initiation

**Published:** 2016-10-23

**Authors:** Marco Iannetta, Miriam Lichtner, Raffaella Rossi, Stefano Savinelli, Serena Vita, Claudia Mascia, Paola Zuccalà, Raffaella Marocco, Maria Antonella Zingaropoli, Maria Rosa Ciardi, Gabriella d'Ettorre, Claudio Maria Mastroianni, Vincenzo Vullo

**Affiliations:** 1 Public Health and Infectious Diseases, Sapienza University, Rome, Italy

## Abstract

**Introduction**: During HIV infection myeloid and lymphoid activation has been reported [1], together with elevation of monocyte/macrophage inflammation markers, such us soluble (s)CD14 and sCD163 [2,3]. We evaluated both myeloid and lymphoid activation markers and correlated them with CD4 recovery after 12 months of ARV treatment, and the time (in days) needed to achieve a viral load below 50 copies/mL.

**Materials and methods**: HIV+ treatment-naïve patients were enroled and followed up for 1 year after treatment initiation. Blood samples were collected before treatment initiation (T0). Monocyte (Mo), dendritic cell (DC) and lymphocyte phenotypes were assessed by flow-cytometry using a lyse-no-wash protocol. sCD14 and sCD163 were measured in plasma with ELISA. Seventeen age- and sex-matched healthy donors (HD) were enroled.

**Results**: We recruited 34 naïve patients (eight women, nine AIDS presenters). Fifteen, ten and six patients started an ARV therapy containing a protease, a non-nucleoside reverse-transcriptase and an integrase inhibitor, respectively. Three patients did not start any treatment. No differences in HIV viral load and CD4 cell counts were observed at T0, according to ARV therapy. At T0, HIV+ subjects showed lower levels of pDC (3976 vs 7043 cells/mL, p<0.001), slanDC (11,644 vs 24,538 cells/mL, p=0.02) and higher levels of CD14+CD16+ Mo (19,369 vs 7157 cells/mL, p<0.001) compared to HD. HLA-DR was reduced on mDC of HIV+ subjects (22,556 vs 37,358, p<0.001) and increased on slanDC (13,680 vs. 9979, p=0.005) compared to HD. Levels of CD4+ and CD8+ T-lymphocyte immune-activation were higher in HIV+ subjects than HD (6.0% vs. 1.8%, p<0.001 and 9.4% vs. 1.1%, p<0.001). Myeloid activation soluble markers sCD14 and sCD163 were higher in HIV+ subjects compared to HD (2163 vs. 1363 ng/mL, p<0.001 and 272.6 vs. 149.1 ng/mL, p=0.08). CD14+CD16+ Mo and CD8 immune-activation were not correlated with the clinical stage of HIV subjects, but positively correlated with HIV viral load. After 1 year of ARV therapy, CD4 recovery positively correlated with basal levels of CD8 immune-activation (Figure 1a), while the choice of protease, non-nucleoside reverse-transcriptase or integrase inhibitor did not affect CD4 recovery. The Kaplan-Meier analysis showed that higher baseline CD14+CD16+ Mo counts were predictive of a lower rate of subjects with a viral load <50 copies/mL, within 150 days from ARV therapy initiation (p=0.03) (Figure 1b).

**Conclusions**: Global evaluation of lymphoid and myeloid activation markers can be useful in predicting time to undetectable viral load and immunological recovery in HIV patients starting ARV therapy.

**Abstract P348–Figure 1:** a) Correlation between activated CD8+HLA-DR+CD38+ T-lymphocytes and CD4 recovery after 1 year of ART (Spearman r: 0.5; p=0.005). b) Kaplan-Meier analysis with endpoint=viral load <50 copies/mL over a period of 150 days (log-rank test p=0.03).

## References

[1] Appay V, Kelleher AD (2016). Immune activation and immune aging in HIV infection. Curr Opin HIV AIDS.

[2] Dutertre CA, Amraoui S, DeRosa A, Jourdain JP, Vimeux L, Goguet M (2012). Pivotal role of M-DC8^+^ monocytes from viremic HIV-infected patients in TNFa overproduction in response to microbial products. Blood.

[3] McKibben RA, Margolick JB, Grinspoon S, Li X, Palella FJJr, Kingsley LA (2015). Elevated levels of monocyte activation markers are associated with subclinical atherosclerosis in men with and those without HIV infection. J Infect Dis.

# Sub article: P349 Impact of oestrogen plasma levels in modulation of immune activation among HIV-infected women and men undergoing successful antiretroviral therapy

**Published:** 2016-10-23

**Authors:** Raffaella Marocco, Miriam Lichtner, Tiziana Tieghi, Valeria Belvisi, Irene Pozzetto, Claudia Mascia, Paola Zuccalà, Raffaella Rossi, Fabio Mengoni, Claudio Maria Mastroianni

**Affiliations:** 1 Deptartment of Public Health and Infectious Diseases Santa Maria Goretti Hospital, Sapienza University, Latina, Italy; 2 Depatment of Public Health and Infectious Diseases Santa Maria Goretti Hospital, Sapienza University, Rome, Italy

## Abstract

**Introduction:** Several sex differences have been described in the natural course of HIV-1 disease [1]. Higher levels of TLR 7-mediated IFN-alpha production together with greater levels of activated CD8-T cells were described in women compared with men for a given HIV viral load [2]. The role of sexual hormones in antiretroviral treatment (ART)-treated women is not completely understood and seems to be crucial to individualize possible eradication strategy in women that could be different than in men [3]. The aim of this study was to investigate the role of sexual hormones in determining innate immunity and immune activation in a cohort of HIV-infected subjects undergoing effective ART.

**Materials and methods:** Seventy-four HIV-infected (41 F, 33 M) subjects receiving stable ART were studied. Immunological test including plasmocytoid and myeloid dendritic cells (DC) count, slan DC and typical, atypical and intermediate monocytes and T cell activation (HLA-DR+/CD38+CD4+ and HLA-DR+/CD38+CD8+). Sex hormones (oestradiol, progesterone and testosterone) were determined using CLIA kit. HIV RNA zenith, delta of CD4+, years of disease and CD4/CD8 ratio were also collected. Non-parametric Mann–Whitney test and Spearman coefficient correlation were used.

**Results:** There were no significant differences in levels of circulating DC (mDC, pDC) between HIV+ women and men; however, a positive correlation was found between mDC and serum oestradiol (p=0.03, r=0.30). We did not observe statistically significant differences among the sub-populations of monocytes and MDC-8, but there was a trend of increased number of atypical inflammatory monocytes and MDC-8 in women. Interestingly, a significant augmentation of DR+38+ CD4+ T cells was found in men (p=0.02), and a negative correlation between DR+38+CD8+ T cells and serum oestradiol levels in all HIV subjects and in women was observed (respectively p=0.0002; r=−0.67; p=0.006, r=−0.50). Moreover, only in women a negative correlation between mDC and DR+38+CD8+ T cells was found (p=0.02; r=−0.43). Progesterone and testosterone levels were not associated with the immune variables considered. Regarding soluble markers of monocytes activation, we did not observe differences although women have lower levels of sCD14 than men (pg/mL, median 2249 and 2685 pg/mL).

**Conclusions:** In HIV aviraemic ART-treated subjects, high levels of oestrogens seem to be associated with an expansion of mDC and a lower activation of CD8 T cells, underlying the importance of considering hormonal status and not only gender and age in designing immunological and therapeutic studies.

## References

[1] Addo MM, Altfeld M (2014). Sex-based differences in HIV type 1 pathogenesis. J Infect Dis.

[2] Meier A, Chang JJ, Chan ES, Pollard RB, Sidhu HK, Kulkarni S (2009). Sex differences in the Toll-like receptor-mediated response of plasmacytoid dendritic cells to HIV-1. Nat Med.

[3] Gianella S, Tsibris A, Barr L, Godfrey C (2016). Barriers to a cure for HIV in women. J Int AIDS Soc.

# Sub article: P350 Active TGF-β1 may be involved in viral suppression during ART in HIV-1 infected patients

**Published:** 2016-10-23

**Authors:** Edward Maina, Elizabeth Bukusi, Martha Sedegah, Margaret Lartey, William Ampofo

**Affiliations:** 1 Virology, Noguchi Memorial Institute for Medical Research, Accra, Ghana; 2 Centre for Microbiology Research, Kenya Medical Research Institute, Nairobi, Kenya; 3 Malaria Program, Naval Medical Research Centre, Silver Spring, MD, USA; 4 Medicine, University of Ghana, Accra, Ghana

## Abstract

**Introduction**: Studies have demonstrated that cytokine-mediated non-cytopathic suppression of viral replication may provide an alternative therapeutic strategy for the treatment of viral infection. We hypothesized that active transforming growth factor (TGF)-β1 cytokine response is involved in HIV-1 suppression during ART.

**Methods**: Fifty-nine HIV-1 infected individual categorized according to virologic and immunologic outcomes after ART as, virologic responder (VR), immune-virologic failure (IVF) and viral suppressed (VS) were included in this cross-sectional study. For comparison, 12 ART-naïve long-term non-progressors (LTNPs) and 10 healthy controls were also included in the study. CD4+ T cell counts and plasma HIV-1 RNA were measured using flow cytometry and HIV-1 TaqMan assays, respectively. Plasma levels of active TGF-β1 were determined using MILLIPLEX^®^ MAP TGF-β1 single plex magnetic bead kit on LuminexMAP^®^ 200 system.

**Results**: Whereas LTNPs had high HIV-1 RNA and CD4+ cell counts, plasma TGF-β1 was low. In patients on ART, VR had high CD4+ cell counts and low HIV-1 RNA contrasted with high TGF-β1 while IVF exhibited high HIV-1 RNA with low CD4+ cell count and TGF-β1. Of note, VS had undetectable HIV-1 RNA with high CD4+ cell counts and plasma concentration of TGF-β1. Furthermore, a negative correlation was observed between HIV-1 RNA and TGF-β1 in patients on ART.

**Conclusions**: Overall, plasma concentration of active TGF-β1 was not significantly different between healthy controls and LTNPs. However, VR and VS displayed a significantly high TGF-β1 and a significantly low and undetectable HIV-1 RNA, respectively. Compared to VR, IVF had low TGF-β1 and higher HIV-1 RNA. This suggests immunoregulatory mechanisms to be involved in suppressing HIV-1 in HIV-infected patients on ART.

# Sub article: P351 Geno2pheno (coreceptor-hiv2): a new diagnostic tool for the genotypic determination of HIV-2 coreceptor usage

**Published:** 2016-10-23

**Authors:** Matthias Döring, Pedro Borrego, Joachim Büch, Andreia Martins, Georg Friedrich, Ricardo Jorge Camacho, Josef Eberle, Rolf Kaiser, Thomas Lengauer, Nuno Taveira, Nico Pfeifer

**Affiliations:** 1 Computational Biology and Applied Algorithmics, Max Planck Institute for Informatics, Saarbrücken, Germany; 2 Research Institute for Medicines (iMed.ULisboa), University of Lisbon, Lisbon, Portugal; 3 Department of Microbiology and Immunology, Rega Institute for Medical Research, KU Leuven, Leuven, Belgium; 4 Department of Virology, Max von Pettenkofer-Institute, Ludwig-Maximilians-University, Munich, Germany; 5 Institute for Virology, University of Cologne, Cologne, Germany

## Abstract

**Introduction**: Maraviroc is a coreceptor antagonist that prevents HIV cell entry by blocking the CCR5-coreceptor. Before initiating treatment with maraviroc, viral coreceptor usage should be determined to ensure that HIV can use only the CCR5 coreceptor (R5) and cannot evade the drug by using the CXCR4 coreceptor (X4-capable). Although maraviroc is a treatment option for individuals infected with HIV-2, no online tool for the genotypic identification of HIV-2 coreceptor usage was available until now. Therefore, our research was concerned with developing, validating and implementing a data-driven web service for the prediction of HIV-2 coreceptor usage from the V3 loop of the HIV-2 glycoprotein.

**Methods**: Several support vector machines (SVMs) were trained and validated on a data set of 73 R5 and 52 X4-capable V3 amino acid samples with known phenotypic coreceptor usage. We compared the nested cross-validation predictions from SVMs with the results from the rules-based method developed by Visseaux et al. [1] using McNemar's test and investigated the predictive performance of individual discriminatory features in the V3 loop using Fisher's exact test with multiple hypothesis correction (Benjamini-Hochberg method at a false discovery rate of 5%).

**Results**: After comparing the predictive performance of all trained SVMs using 10 runs of 10-fold cross-validation, we selected a linear SVM as the model for geno2pheno (coreceptor-hiv2), because it performed best (area under the ROC curve of 0.95). In our evaluations of predictive performance using 10-fold nested cross-validation, SVMs had a sensitivity of 73.5% and a specificity of 96% for identifying X4-capable variants. We found that the predictive performance of SVMs was not significantly different (p=0.37) from the rules-based approach developed by Visseaux et al. Moreover, on a test set containing nine new V3 sequences together with the corresponding coreceptor usage phenotypes, geno2pheno (coreceptor-hiv2) achieved a predictive accuracy of 100% and outperformed the rules-based approach. Using SVMs, we could not only reproduce the established markers of CXCR4-usage but could also identify novel markers: the substitutions 27K, 15G and 8S were significantly predictive of CXCR4-usage.

**Conclusions**: In this study, we developed geno2pheno (coreceptor-hiv2), the first online tool for the prediction of HIV-2 coreceptor usage from the V3 loop. The tool can aid clinicians in deciding whether maraviroc is a treatment option and allows for broader epidemiological studies on HIV-2 coreceptor usage. Moreover, our research indicates that HIV-2 coreceptor usage is not only influenced by the V3 loop but also by the V1/V2 regions. Geno2pheno (coreceptor-hiv2) is available at www.coreceptor-hiv2.geno2pheno.org.

## References

[1] Visseaux B, Hurtado-Nedelec M, Charpentier C, Collin G, Storto A, Matheron S (2012). Molecular determinants of HIV-2 R5–X4 tropism in the V3 loop: development of a new genotypic tool. J Infect Dis.

# Sub article: P352 High rates of multi-class drug resistance in HIV-1 infected individuals monitored with CD4 count in Uganda

**Published:** 2016-10-23

**Authors:** Amrei von Braun, Alexandra Scherrer, Christine Sekaggya, Joseph Kirangwa, Deogratius Ssemwanga, Pontiano Kaleebu, Huldrych Günthard, Andrew Kambugu, Barbara Castelnuovo, Jan Fehr

**Affiliations:** 1 Infectious Diseases Institute, College of Health Sciences, Makerere University, Research, Kampala, Uganda; 2 Infectious Diseases and Hospital Epidemiology, University Hospital of Zurich, University of Zurich, Zurich, Switzerland; 3 Research, MRC/UVRI Uganda Research Unit on AIDS, Research, Entebbe, Uganda

## Abstract

**Introduction:** Until a recent change in guidelines, HIV-infected patients on antiretroviral therapy (ART) in Uganda were monitored using CD4 cell counts only. So far, little is known about prevalence of drug resistance among HIV-infected patients with virological failure (VF) after immunological treatment monitoring in Uganda.

**Methods:** From 4 June to 30 September 2015, viral load measurements were done in HIV-infected adults (18 years) on ART for at least 6 months presenting to the infectious diseases institute (IDI) in Kampala. In case of VF (>1000 copies/mL), HIV genotyping was requested. Sequencing of partial polymerase gene was conducted using an in-house protocol. All sequences were submitted to the Stanford University HIV Drug Resistance database, and the surveillance drug resistance mutations were identified using the 2009 World Health Organization mutations list. HIV-1 subtypes were determined using REGA version 3.0.

**Results:** Viral load measurements were done in 2511 patients, who had been on ART for a median time of 4.7 years (interquartile range (IQR) 2.5–8.7). A total of 199 patients (7.9%) had VF with a median viral load of 4.4 log10 copies/mL (IQR 3.9–4.9). The majority of patients with VF (140, 70.4%) were on first-line ART, 138 (69.3%) were female and the median age was 37 years (IQR 30–43). HIV genotyping tests were available in 163 (81.9%). HIV-1 subtypes A (46%) and D (34%) were most common. Relevant drug resistance mutations were observed in 135 (82.8%), of which 103 (63.2%) had resistance to two drug classes, and 11 (6.8%) had resistance to all three drug classes available in Uganda (Figure 1).

**Conclusions:** With 92% of all patients virologically suppressed, the overall prevalence of VF was low and is in line with the third of the 90–90–90 UNAIDS targets. However, the majority of failing patients had developed resistance to more than one drug class, suggesting that failing regimens - not identified as such by CD4 monitoring - had been in place for a prolonged period of time.

**Figure 1:** Type and frequency of most prevalent resistance-associated mutations observed. **NNRTI, non-nucleoside/nucleotide reverse transcriptase inhibitors; NRTI, nucleoside/nucleotide reverse transcriptase inhibitors; PI, protease inhibitors; TAM, thymidine analogue mutation**

# Sub article: P353 Prevalence and impact of transmitted drug resistance in recent HIV-1 infections, Germany 2013 to 2015

**Published:** 2016-10-23

**Authors:** Andrea Hauser, Alexandra Hofmann, Kirsten Hanke, Viviane Bremer, Barbara Bartmeyer, Claudia Kücherer, Norbert Bannert

**Affiliations:** 1 HIV and Other Retroviruses, Robert Koch Institute, Berlin, Germany; 2 HIV/AIDS, STI and Blood-borne Infections, Robert Koch Institute, Berlin, Germany

## Abstract

**Introduction**: Transmitted drug resistance (TDR) in new HIV infections has significant clinical consequences for the treatment success. Therefore, monitoring of TDR in currently circulating HIV strains is an important public health issue. We aim to estimate the prevalence of TDR to protease and reverse transcriptase inhibitors (PIs; RTIs) and to assess the impact on antiretroviral treatment according to the currently recommended first-line regimens (European AIDS Clinical Society (EACS) HIV Guidelines version 8.0^1^].

**Materials and methods**: Diagnostic laboratories provided dried serum spots (DSS) of ~60% of all newly diagnosed HIV infections in Germany reported to the Robert Koch Institute (2013–2015). HIV-1 genotyping was performed from “recent infections” (<155 days) as classified by the commercial BED HIV-1 Incidence EIA (Sedia Biosciences Corporation, Oregon USA); exclusive cases with CD4<100 cells/L, CDC C) to identify resistance-associated mutations according to the WHO surveillance drug resistance mutations [2].

**Results**: Between 2013 and 2015, 3,114/9 and 799 DSS were classified as recent infection and 1460 were sequenced. Overall prevalence of TDR was 10.8% (102/1460), comprising 3.8% NRTI-, 2.8% NNRTI-, 2.9% PI-mono-resistance and 1.2% multi-class-resistance. 80% (82/102) of NRTI mutations were thymidine analogue mutations (TAMs) and T215 revertants, namely M41L (1.4%, 20/1460), K219NQR (1.0%, 15/1460), D67EGN (0.7%, 10/1460), T215Y, K70R, L210W (each 1/1460) and T215CDEIS (2.3%, 34/1460), conferring intermediate resistance to zidovudine and stavudine. 60% of NNRTI-resistance was caused by K103NS (38/1460) conferring resistance to efavirenz and nevaripine. The most frequent PI mutations M46IL (1.5%; 22/1460) and V82FL (0.8%; 12/1460) are associated with low/intermediate resistance to tipranavir, nelfinavir and/or fosamprenavir. Considering only primary resistance mutations which impact to drugs currently recommended in first-line regimens [1], the prevalence of TDR was only 5.4% (0.8% NRTI; 3.1% NNRTI; 0.6% PI; 0.9% multi-drug-resistance).

**Conclusions**: TDR prevalence in recent HIV-1 infections in Germany (2013–2015) remained stably high (>10%) and is comparable to other European countries. TDR was mainly caused by the first-generation NNRTI-selected K103NS, by long-term persisting TAMs and the PI-selected M46IL and V82FL. While the K103NS is associated with failure of current efavirenz-containing first-line regimens, the impact of TAMs and frequent PI-mutations on the success of current first-line therapies is predicted to be low and halves the TDR prevalence (10.8% to 5.4%). However, to allow an optimal therapeutic sequencing genotypic resistance testing prior to treatment initiation is important and should also include the HIV-integrase.

## References

[1] European AIDS Clinical Society (EACS) http://www.eacsociety.org/guidelines/eacs-guidelines/eacs-guidelines.html.

[2] Bennett D, Camacho R, Otelea D, Kuritzkes D, Fleury H, Kiuchi M (2009). Drug resistance mutations for surveillance of transmitted HIV-1 drugresistance: 2009 update. PLoS One.

# Sub article: P354 Higher rates for transmission of NNRTI-resistant viruses for subtype A versus subtype B strains in Southern Greece

**Published:** 2016-10-23

**Authors:** Evangelia-Georgia Kostaki, Vana Sypsa, Georgios Nikolopoulos, Panagiotis Gargalianos, Georgios Xylomenos, Marios Lazanas, Maria Chini, Athanasios Skoutelis, Vasileios Papastamopoulos, Anastasia Antoniadou, Antonios Papadopoulos, Mina Psichogiou, Georgios Daikos, Georgios Chrysos, Vasileios Paparizos, Sofia Kourkounti, Helen Sambatakou, Nikolaos Sipsas, Malvina Lada, Periklis Panagopoulos, Efstratios Maltezos, Angelos Hatzakis, Dimitrios Paraskevis

**Affiliations:** 1 Department of Hygiene, Epidemiology and Medical Stastistics, Medical School, National and Kapoditrian University of Athens, Athens, Greece; 2 HIV Department, Hellenic Center for Diseases Control and Prevention, Athens, Greece; 3 1st Department of Internal Medicine, G. Gennimatas General Hospital, Athens, Greece; 4 3rd Internal Medicine Department of Infectious Disease, Red Cross General Hospital, Athens, Greece; 5 5th Department of Medicine and Infectious Diseases, Evangelismos General Hospital, Athens, Greece; 6 4th Department of Medicine, Attikon General Hospital, Medical School, National and Kapodistrian University of Athens, Athens, Greece; 7 1st Department of Medicine, Laiko General Hospital, Medical School, National and Kapodistrian University of Athens, Athens, Greece; 8 Department of Internal Medicine, Tzaneion General Hospital, Piraeus, Greece; 9 HIV/AIDS Unit, “A. Syngros” Hospital of Dermatology and Venereology, Athens, Greece; 10 2nd Department of Internal Medicine, Hippokration General Hospital, Medical School, National and Kapodistrian University of Athens, HIV Unit, Athens, Greece; 11 Department of Pathophysiology, Medical School, Laiko General Hospital, National and Kapodistrian University of Athens, Athens, Greece; 12 2nd Department of Internal Medicine, Sismanogleion General Hospital, Athens, Greece; 13 Department of Internal Medicine, University General Hospital, Democritus University of Thrace, Alexandroupolis, Greece

## Abstract

**Introduction**: We have previously found that the most prevalent NNRTI-resistant mutations among HIV-1 drug-naïve individuals in Southern Greece were E138A and K103N. Our aim was to estimate the transmission dynamics of E138A- and K103N-resistant strains and to investigate for potential differences in these dynamics between subtypes A and B.

**Materials and methods**: We analyzed all sequences with E138A from 179 and 68 HIV-1 treatment-naïve individuals sampled in Southern Greece infected with subtype A and B, respectively. Similarly, we analyzed 56 and 18 sequences with K103N from subtypes A and B. Phylodynamic analyses were performed using the birth-death model (BDM) in BEAST version 1.8.

**Results**: The distributions of transmission risk groups were similar for subtypes A and B for both E138A and K103N. Men who have sex with men (MSM) represented 69% (N=124) and 63% (N=43) of infections with E138A in subtypes A and B, respectively (p=0.586). Similarly, MSM comprised 68% (N=38) and 61% (N=11) of individuals with K103N in subtypes A and B, respectively (p=0.355). The time of the most recent common ancestor (tMRCA) for E138A was estimated in 1992.0 (95% HPD 1987.6–1995.6) and 1982.6 (95% HPD 1973.7–1990.6) for subtypes A and B, respectively. For K103N, the tMRCA was in 1999.0 (95% HPD 1994.7–2002.5) and 1991.8 (95% HPD 1979.1–2000.8) for subtypes A and B, respectively. Notably, the slope of the number of lineages (transmissions) over time estimated at the exponential phase of the BDM skyline for E138A sequences of subtype A (10.13, 95% CI 9.30–10.90) was 10 times that of subtype B (1.04, 95% CI 0.96–1.11) (Figure 1a). For K103N, the slope for subtype A transmissions was approximately 2.5 times (6.16, 95% CI 5.80–6.52) that for subtype B (2.50, 95% CI 2.45–2.55) (Figure 1b).

**Conclusions**: Our study suggests that E138A and K103N HIV-1 resistant mutations are transmitted at higher rates in subtype A than in subtype B strains. Given that the distributions of transmission risk groups were similar between the two clades, observed differences in transmission dynamics could be due to higher transmissibility of subtype A or different risk behaviour of the individuals infected with this subtype. This is one of the few studies highlighting differences in transmission dynamics of resistant strains belonging to different subtypes.

**Figure 1:** Transmission dynamics of resistant strains with E138A for subtypes A and B. (b) Transmission dynamics of resistant strains with K103N for subtypes A and B.

# Sub article: P355 The absence of drug resistance against dolutegravir in first-line therapy is attributable to reduced viral replicative fitness and durable anti-HIV immune responsiveness

**Published:** 2016-10-23

**Authors:** Mark Wainberg, Thibault Mesplede

**Affiliations:** 1 McGill University AIDS Centre, Jewish General Hospital, Montreal, Canada

## Abstract

**Introduction:** Dolutegravir (DTG) is an integrase strand transfer inhibitor (INSTI) against which drug resistance in first-line therapy has never been observed. However, a R263K mutation that confers low-level resistance (3–4-fold) to DTG was selected by us in culture and also developed in several patients who received DTG as an INSTI after having failed other drugs. The absence of resistance to DTG is due to a high fitness cost that is exacted by the R263K mutation, and the fact that compensatory mutations for R263K have not occurred.

**Methods:** We measured levels of integrated HIV DNA in cells infected by HIV containing R263K and other INSTI and non-INSTI resistance mutations. We also monitored immune responsiveness to HIV in patients receiving DTG-based therapy.

**Results:** The R263K substitution alone conferred an approximate 3-fold level of resistance to DTG, a 40% loss inviral replicative capacity and a 40% drop in recombinant integrase activity. A continuation of DTG drug pressure led to secondary mutations at positions H51Y, E138K or T66I that did not individually affect DTG resistance or enzyme activity. However, the combination of R263K with H51Y or E138K slightly increased DTG resistance but also caused a 90% loss in each of viral replication capacity and integrase activity as measured both biochemically and by PCR. Most importantly, the continued propagation in culture of viruses containing both R263K and H51Y yielded progressively less integrated viral DNA in successive infections, beginning at 30% of wild-type and dramatically decreasing to non-detectability thereafter. In addition, our data show that HIV that is subjected to DTG pressure is unable to evolve and remains durably susceptible to anti-HIV neutralizing antibodies and Tcell immune responses.

**Conclusions:** Our findings explain why drug resistance to DTG has not been observed after first-line therapy for more than 3 years since its approval by regulatory agencies. The use of DTG in first-line therapy may be compatible with treatment interruption strategies aimed at attaining a functional HIV cure because of the non-development of drug resistance.

# Sub article: P356 Low prevalence of pre-treatment HIV-1 drug resistance in Ugandan adults

**Published:** 2016-10-23

**Authors:** Amrei von Braun, Christine Sekaggya, Alexandra Scherrer, Brian Magambo, Deogratius Ssemwanga, Pontiano Kaleebu, Huldrych Günthard, Andrew Kambugu, Jan Fehr, Barbara Castelnuovo

**Affiliations:** 1 Infectious Diseases Institute, College of Health Sciences, Makerere University, Research, Kampala, Uganda; 2 Infectious Diseases and Hospital Epidemiology, University Hospital of Zurich, University of Zurich, Zurich, Switzerland; 3 MRC/UVRI Uganda Research Unit on AIDS, Research, Entebbe, Uganda

## Abstract

**Introduction**: Previous studies on pre-treatment drug resistance from sub-Saharan Africa have shown the highest prevalence in Uganda, particularly in Kampala, with a prevalence of 12.3%. Antiretroviral therapy (ART) has been publicly available in Uganda since 2000, with initial use - although limited - of mono/dual thymidine analogues. This study aims to describe type and frequency of pre-treatment resistance in HIV-infected Ugandan adults seeking care at one of the largest public-sector providers in Kampala, Uganda.

**Methods**: From 4 June to 30 September 2015, ART-naïve adults (18 years) presenting to the Infectious Diseases Institute (IDI) in Kampala and willing to participate in this study were asked to give a plasma sample for pre-treatment HIV genotyping. Sequencing of partial polymerase gene was conducted using an in-house protocol. All sequences were submitted to the Stanford University HIV Drug Resistance database, and the surveillance drug resistance mutations were identified using the 2009 World Health Organization mutations list.

**Results**: Pre-treatment drug resistance testing was available from 152 ART-naïve HIV-infected adults, of which 96 (63.2%) were female with a median age of 33 years (interquartile range (IQR) 26–41), and a median CD4 cell count of 511 cells/µL (IQR 284–713). Mutations associated with HIV drug resistance were found in 9/152 (5.9%) patients. Five patients (5/152, 3.3%) harboured nucleoside reverse transcriptase inhibitors (NRTI) mutations, and 8/152 (5.3%) had non-nucleoside reverse transcriptase Inhibitors (NNRTI) mutations. Five (3.3%) patients had one-class mutations, and four (2.6%) showed double class resistance. Protease inhibitor mutations were not observed (for specific mutations see Table 1).

**Conclusions**: Contrary to previous reports we found a low prevalence of pre-treatment drug resistance among Ugandan adults in Kampala. We hypothesize that the use of mono/dual thymidine analogues in the past contributed to a higher circulation of thymidine analogue mutations (TAMs), as observed in developed settings. The subsequent swift scale-up of triple ART in the region may have reduced pre-treatment resistance over time.

**Table 1:** Observed pre-treatment drug resistance mutations

Drug class and mutations	Total N = 152 (%)
Any NRTI mutation	5 (3.3)
K65R	1 (0.7)
M184V	2 (1.3)
Other (M41L, T215I)	2 (1.3)
Any NNRTI mutation	8 (5.3)
K101E	3 (2.0)
Y181C	2 (1.3)
K103N	2 (1.3)
Other (M230L, G190A/S, Y188L)	4 (2.6)

# Sub article: P357 Prevalence of resistance mutations to rilpivirine and etravirine in people starting antiretrovirals in Argentina

**Published:** 2016-10-23

**Authors:** Emiliano Bissio, María Gabriela Barbás, María Belén Bouzas, Analía Cudolá, Carlos Falistocco, Horacio Salomón

**Affiliations:** 1 Clinical Research, Fundación Centro de Estudios Infectológicos, Buenos Aires, Argentina; 2 Biología Molecular, Laboratorio Central de Córdoba, Córdoba, Argentina; 3 División Análisis Clínicos, Hospital Muñiz, Buenos Aires, Argentina; 4 Dirección de Sida, Ministerio de Salud de la Nación, Buenos Aires, Argentina; 5 Facultad de Medicina, Instituto de Investigaciones Biomédicas en Retrovirus y Sida (INBIRS), Buenos Aires, Argentina

## Abstract

**Introduction**: According to many reports, the prevalence of resistance mutations to non-nucleoside reverse transcriptase inhibitors (NNRTIs) in antiretroviral-naïve patients is increasing; and reaches or exceeds 10% in some regions. Recently, a surveillance study performed in Argentina determined that the prevalence of resistance to first-generation NNRTIs among people starting ART and no history of previous exposure was 10%. However, some mutations conferring resistance to newer-generation NNRTIs were not considered since these are not yet recommended as first-line therapy in this country. Rilpivirine-based regimens are now preferred or alternative first-line regimens according to many guidelines. The aim of this study was to analyze the prevalence of resistance mutations to newer-generation NNRTIs (rilpivirine and etravirine) in the population starting ART in Argentina.

**Methods:** We analyzed the prevalence of resistance mutations obtained through a nationally representative pretreatment HIV-drug resistance (PDR) surveillance study performed in Argentina from 2014 to 2015. Briefly, 30 ART-dispensing sites throughout the country were randomly chosen to enrol 330 adults starting ART (without prior exposure or re-starting ART); to generate a point prevalence estimate of resistance-associated mutations (RAMs) with a maximum 5% confidence interval (for both the total population and for those without ARV exposure). Samples were processed with Trugene (Siemens^®^), and analyzed using the Stanford algorithm HIVdb Program, Genotype Resistance Interpretation, version 6.3.1. This report incorporated in the analysis the mutations that, according to the IAS list [1], confer resistance to rilpivirine or etravirine (and were not considered for the original analysis).

**Results:** Between August 2014 and March 2015, we obtained 330 samples from people starting ART in the selected sites. Mean (SD) age was 35 (11.0) years; 63.4% were male; median (IQR) CD4 count was 275/mm^3^ (106–461) and 16.6% had prior ARV exposure. For the population without prior exposure, the prevalence of RAMs was 13% (±4%), and prevalence of first-generation NNRTI RAMs was 10% (±4%). The prevalence of resistance mutations for second-generation NNRTIs was 7% (±3%) (17 samples with mutations out of 239 successfully sequenced samples). The most frequent mutations conferring resistance to rilpivirine or etravirine were: E138A (n=6) and E190A (n=4).

**Conclusions:** This PDR surveillance study showed concerning levels of HIVDR in Argentina, not only for first-generation NNRTIs but also to rilpivirine and etravirine. In our setting, performing resistance testing would be necessary before prescription of ART even if the person would start a second-generation NNRTI-based regimen.

## References

[1] Wensing AM, Calvez V, Günthard HF, Johnson VA, Paredes R, Pillay D (2015). 2015 update of the drug resistance mutations in HIV-1. Top Antivir Med.

# Sub article: P358 Frequency of additional resistance relevant mutations in 2% and 1% population proportions in next-generation sequencing (NGS) in routine HIV-1 resistance diagnostics

**Published:** 2016-10-23

**Authors:** Robert Ehret, Andrew Moritz, Marcel Schuetze, Martin Obermeier

**Affiliations:** 1 Laboratory, Medical Center for Infectious Diseases Berlin, Berlin, Germany

## Abstract

**Introduction:** NGS technologies have made their way into routine diagnostics in HIV-1 resistance testing. The report of mutations of at least 10% of the viral population is chosen by many laboratories due to its equivalency to Sanger sequencing minority detection. The relevance of mutation detected in lower frequencies is still a subject of debate. We here report the frequency of additional mutations in population proportions of greater than 2% and 1% in routine laboratory testing.

**Materials and methods:** All HIV-1 resistance tests (RTI and PI) performed between October 2014 and April 2016 with an in-house PCR followed by NGS (Illumina MiSeq, sequences reported with >100 reads only) were analyzed. Sequences were interpreted by HIV-GRADE (www.hiv-grade.de) for resistance mutations using 10%, 2% and 1% minority cut-offs. Besides the subtype and the overall increase in mutations, a specific focus were differences in reported resistance-associated mutations. We analyzed potential increase in resistance levels (e.g. additional drug class or further drugs in the same class).

**Results:** We performed 645 NGS resistance tests for HIV-1 reverse transcriptase/protease. Four hundred and eighty-three (74.9%) were identified as subtype B. No drug resistance-associated mutations were reported by HIV-GRADE for 44% with a 10% cut-off, 29.5% and 19.7% with 2% and 1%, respectively. With a cut-off of 10% in 148 samples (105 non-B subtype), only PI relevant mutations were detected. We found mutations only relevant for NRTIs in 21 samples and for NNRTIs in 100 samples. At a cut-off of 2%, we detected mutations in 94 more samples increasing to 157 samples when utilizing a cut-off of 1%. A relevant increase in resistance levels compared with a 10% cut-off was observed for 102 samples at a cut-off of 2% and for 229 samples in the 1% cut-off group. The increase of resistance when lowering the cut-off could be shown for all drug classes with the highest proportions in the NNRTI drug class.

**Conclusions:** A relative high portion (56%) of investigated sequences showed resistance mutations at a minority cut-off of 10%. Even removing the non-B subtype sequences, containing only secondary or subtype specific mutations, still left 50% with resistance-associated mutations. This high percentage of resistance increases substantially lowering the cut-off range to 2 or 1%. That's true not only for the numbers of mutations but also regarding resistance-levels. There is a clear need for clinical evaluation of the relevance of mutations in the low percentage range in NGS for resistance interpretation due to its broader use in clinical routine.

# Sub article: P359 Impact of baseline NNRTI resistance in antiretroviral-naïve patients in a large urban clinic

**Published:** 2016-10-23

**Authors:** Samantha Steinberg, Fred Crouzat, Ina Sandler, Brenda Varriano, Graham Smith, Colin Kovacs, Jason Brunetta, Benny Chang, Barry Merkley, David Tilley, David Fletcher, Megan Acsai, David Knox, Malika Sharma, Mona Loutfy

**Affiliations:** 1 Biomedical Sciences, University of Guelph, Toronto, Canada; 2 Family medicine, Maple Leaf Medical Clinic, Toronto, Canada; 3 Statistics, Maple Leaf Medical Clinic, Toronto, Canada; 4 Institute of Medical Science, University of Toronto, Toronto, Canada; 5 Department of Medicine, University of Toronto, Toronto, Canada; 6 Infectious Diseases, Maple Leaf Medical Clinic, Toronto, Canada

## Abstract

**Introduction:** NNRTIs are prone to baseline resistance and potential treatment failure. We investigated the NNRTI resistance profiles of antiretroviral–naïve patients in a large urban clinic setting and assessed their response to initial ART.

**Materials and methods:** This was a retrospective clinical chart review of ART-naïve patients with baseline genotypes available. We assessed the frequency of NNRTI mutations. Of those who started ART, we conducted Cox regression to determine correlates of virologic suppression (defined as viral load (VL) ≤40 or 50 copies/mL by 6 months) with presence of baseline NNRTI resistance as the primary correlate. Of those with virologic suppression, we conducted Cox regression to determine correlates of virologic rebound (defined as VL ≥200 copies/mL). Censoring occurred for those who did not have any follow-up VLs and at last VL or visit date for those without evidence of viral suppression.

**Results:** Of the 1338 that fit the inclusion criteria, 90 (8.4%) had baseline NNRTI resistance (39 with 103N, 20 with 138A/G/K, 17 with 181C and 8 with 101E/H/P). Of the 90, nine (10%) had 184V, 23 (26%) had NRTI mutations and six (7%) had PI mutations. One thousand two hundred and eighteen (91%) of the ART-naïve patients were started on ART. Patients without NNRTI mutations were most commonly started on NNRTI-based regimens (41%), followed by PI-based (30%) and integrase inhibitor (INI)-based regimens (11%). Patients with baseline NNRTI resistance (n=83) were most commonly started on PI-based regimens (41%), followed by INI-based regimens (19%). Virologic suppression was observed for 963 out of 1218 individuals (79%) that started ART. Eighty-five percent and 90% of patients with and without NNRTI mutations achieved suppression, respectively. In univariate Cox regression, the presence of baseline NNRTI resistance did not impact virologic suppression (HR 0.98; 95% CI 0.76–1.24). In multivariable analysis, adjusting for age, gender, baseline VL and CD4 count, duration of HIV and baseline PI mutations, the presence of NNRTI mutations still did not impact virologic suppression (aHR 0.96; 95% CI 0.74–1.24). For virologic rebound, the presence of baseline NNRTI resistance also did not impact its occurrence (HR 1.11; 95% CI 0.68–1.81). In multivariable analysis, after adjusting for age, gender, baseline VL and CD4 count, duration of HIV and baseline PI mutations, the presence of NNRTI mutations also did not impact virologic rebound (aHR 1.09; 95% CI 0.66–1.78).

**Conclusions:** Baseline NNRTI mutations were present in 8.4% of our antiretroviral-naïve patients. Despite having baseline NNRTI mutations, the majority of the patients reached virologic suppression and did not experience changes in virologic rebound.

# Sub article: P360 Enhanced surveillance to study HIV-1 drug resistance among naïve individuals in Southern Greece: the added value of molecular epidemiology to public health

**Published:** 2016-10-23

**Authors:** Dimitrios Paraskevis, Evangelia-Georgia Kostaki, Emmanouil Magiorkinis, Panagiotis Gargalianos, Georgios Xylomenos, Marios Lazanas, Maria Chini, Athanasios Skoutelis, Vasileios Papastamopoulos, Anastasia Antoniadou, Antonios Papadopoulos, Mina Psichogiou, Georgios Daikos, Assimina Zavitsanou, Georgios Chrysos, Vasileios Paparizos, Sofia Kourkounti, Martha Oikonomopoulou, Helen Sambatakou, Nikolaos Sipsas, Malvina Lada, Periklis Panagopoulos, Efstratios Maltezos, Stylianos Drimis, Angelos Hatzakis

**Affiliations:** 1 Department of Hygiene, Epidemiology and Medical Statistics, Medical School, National and Kapoditrian University of Athens, Athens, Greece; 2 1st Department of Internal Medicine, G Genimatas General Hospital, Athens, Greece; 3 3rd Internal Medicine Department, Infectious Diseases Unit, Red Cross General Hospital, Athens, Greece; 4 5th Department of Medicine and Infectious Diseases, Evangelismos General Hospital, Athens, Greece; 5 4th Department of Medicine, Attikon General Hospital, Medical School, National and Kapodistrian University of Athens, Athens, Greece; 6 1st Department of Medicine, Laiko General Hospital, Medical School, National and Kapodistrian University of Athens, Athens, Greece; 7 Department of Internal Medicine, Tzaneion General Hospital, Piraeus, Greece; 8 HIV/AIDS Unit, “A. Syngros” Hospital of Dermatology and Venereology, Athens, Greece; 9 HIV Unit, 2nd Department of Internal Medicine, Hippokration General Hospital, Medical School, National and Kapodistrian University of Athens, Athens, Greece; 10 Department of Pathophysiology, Laiko General Hospital, Medical School, National and Kapodistrian University of Athens, Athens, Greece; 11 2nd Department of Internal Medicine, Sismanogleion General Hospital, Athens, Greece; 12 Department of Internal Medicine, University General Hospital, Democritus University of Thrace, Alexandroupolis, Greece

## Abstract

**Introduction:** The prevalence of resistance to NNRTI was previously estimated to be 16.9% among drug-naïve individuals in Greece. Our aim was to investigate the dispersal patterns of HIV-1 resistant strains and to estimate the effective reproductive number (*Re*) and transmission dynamics for locally transmitted resistance.

**Materials and methods:** We analyzed sequences from 3428 HIV-1 treatment-naïve individuals sampled in Southern Greece during 1 January 2003 to 31 June 2015. Phylogenetic analysis was performed on subtype A (N=235) and B (N=86) sequences with NNRTI resistance (K103N and E138A), along with sequences isolated from seropositives without resistance from Greece sampled during 1998 to 2013 (subtype A: N=904; subtype B: N=1615) and a randomly selected global dataset (subtype A: N=5907; subtype B: N=3984). Phylogenetic trees were inferred by maximum likelihood method as implemented in RAxML. Phylodynamic analyses were performed using birth-death models (BDM) as implemented in BEAST2.

**Results:** Phylogenetic analyses revealed that for subtype A, the majority of individuals infected with resistant strains (209 out of 235, 88.9%) belonged to monophyletic clusters (local transmission networks, LTNs). Specifically, 48 out of 56 (85.7%) of sequences with K103N, and 148 out of 179 (82.7%) with E138A belonged to one and four LTNs, respectively. These findings suggest that the viruses with the most prevalent resistance mutations spread locally. For subtype B, either non-clustered sequences or small LTNs (2–6 sequences), were identified. The time of the most recent common ancestor (tMRCA) was in 2007 (95% HPD: 2004–2009) for the K103N cluster versus 1995 (95% HPD: 1991–1999), 1996 (95% HPD: 1989–2000), 1997 (95% HPD: 1991–2001) and 2004 (95% HPD: 2000–2007) for E138A LTNs (Figure 1). For the K103N sub-outbreak the *Re* was higher than 1 between 2008 and the first half of 2013 (maximum value of median *Re*=2.8) (Figure 1). On the contrary, for all E138A LTNs the *Re* was higher between 2011 and 2015, except the most recent one where the *Re* was approximately equal to 1.

**Conclusions:** Our study suggests that the most prevalent mutations associated with resistance to NNRTIs were transmitted through local networks in Greece. Notably, phylodynamic analysis allows estimating that resistance in the last few years has been actively propagated with an increasing incidence. Those belonging to the active TDR networks are the priority population for prevention (TasP). Our study highlights the added value of the latest advances in molecular epidemiology to public health since these allow us to estimate critical epidemiologic parameters and therefore the priority population to intervene.

**Figure 1:** Phylodynamic characteristics of the major sub-outbreaks with resistant mutations in Southern Greece.

# Sub article: P361 Transmission patterns of HIV-1 subtype A resistant strains across Greece: evidence for country and regional level transmission networks

**Published:** 2016-10-23

**Authors:** Dimitrios Paraskevis, Lemonia Skoura, Evangelia-Georgia Kostaki, Panagiotis Gargalianos, Georgios Xylomenos, Marios Lazanas, Maria Chini, Symeon Metallidis, Athanasios Skoutelis, Vasileios Papastamopoulos, Anastasia Antoniadou, Antonios Papadopoulos, Mina Psichogiou, Georgios Daikos, Dimitrios Pilalas, Assimina Zavitsanou, Georgios Chrysos, Vasileios Paparizos, Sofia Kourkounti, Dimitrios Chatzidimitriou, Helen Sambatakou, Nikolaos Sipsas, Malvina Lada, Periklis Panagopoulos, Efstratios Maltezos, Stylianos Drimis, Angelos Hatzakis

**Affiliations:** 1 Department of Hygiene, Epidemiology and Medical St, Medical School, National and Kapoditrian University of Athens, Athens, Greece; 2 National AIDS Reference Centre of Northern Greece, School of Medicine, Aristotle University of Thessaloniki, Thessaloniki, Greece; 3 1st Department of Internal Medicine, G. Gennimatas General Hospital, Athens, Greece; 4 3rd Internal Medicine Department-Infectious Disease, Red Cross General Hospital, Athens, Greece; 5 1st Internal Medicine Department, School of Medicine, Aristotle University of Thessaloniki, Thessaloniki, Greece; 6 5th Department of Medicine and Infectious Diseases, Evangelismos General Hospital, Athens, Greece; 7 Attikon General Hospital, Medical School, National and Kapodistrian University of Athens, 4th Department of Medicine, Athens, Greece; 8 1st Department of Medicine, Laiko General Hospital, Medical School, National and Kapodistrian University of Athens, Athens, Greece; 9 Department of Internal Medicine, Tzaneion General Hospital, Piraeus, Greece; 10 HIV/AIDS Unit, “A. Syngros” Hospital of Dermatology and Venereology, Athens, Greece; 11 HIV Unit, 2nd Department of Internal Medicine, Hippokration General Hospital, Medical School, National and Kapodistrian University of Athens, Athens, Greece; 12 Department of Pathophysiology, Laiko General Hospital, Medical School, National and Kapodistrian University of Athens, Athens, Greece; 13 2nd Department of Internal Medicine, Sismanogleion General Hospital, Athens, Greece; 14 Department of Internal Medicine, University General Hospital, Democritus University of Thrace, Alexandroupolis, Greece

## Abstract

**Introduction**: The prevalence of mutations conferring resistance to NNRTIs was previously reported to be higher than 15% among drug-naïve individuals both in Northern and Southern Greece. The most prevalent resistance mutations were E138A, K103N and Y181C associated mostly with subtype A1. Our aim was to investigate the dispersal patterns of HIV-1 resistant strains across Greece.

**Materials and methods**: We analyzed sample of subtype A1 sequences (N=1104) obtained between 1999 and middle-2015 from both areas in Greece. We included sequences only from Greece since we have shown previously that subtype A1 sequences have been mostly found within a single monophyletic cluster. Phylogenetic trees were inferred by maximum likelihood method as implemented in RAxML using the GTR+G as nucleotide substitution model with bootstrapping.

**Results**: Phylogenetic analyses revealed that E138A and K103N resistant strains have spread through large monophyletic clusters spanning both Northern and Southern Greece, suggesting that all transmissions within these clusters occurred regionally. Conversely, Y181C formed a subnetwork (monophyletic cluster) limited in Northern Greece with only a single spill over to Southern Greece. Specifically, for K103N strains we found a large (N=49) and a small cluster (N=5) including sequences from both areas. Sequences from Northern Greece formed two specific subnetworks, suggesting local dispersal. Similarly sequences with E138A from Northern Greece formed two specific subnetworks within the E138A monophyletic clades found for Greece. The latter consisted of four major clades of 53, 41, 29 and 25 sequences from both regions. Overall, we found that E138A and K103N spread through common networks across the country with evidence of local transmissions in Northern Greece. On the contrary, Y181C has spread only in Northern Greece with very limited dispersal to Southern Greece.

**Conclusions**: A high prevalence of NNRTI resistance mutations was previously reported for the subtype A1 strains circulating in Greece and especially in Northern Greece. Our study provides evidence that the majority of these resistant viruses were transmitted within common transmission networks. Notably, significant clustering of sequences from Northern Greece as well as the existence of a regional cluster suggest high transmission networking of the population in this area; a finding that might explain the higher prevalence of transmitted drug resistance (TDR) in Northern Greece. Our study highlights the priority population to prevent TDR in the future.

# Sub article: P362 Occurrence and risk factors for primary integrase resistance-associated mutations in Austria in the years 2008-2013

**Published:** 2016-10-23

**Authors:** Alexander Zoufaly, Claudia Kraft, Caroline Schmidbauer, Elisabeth Puchhammer

**Affiliations:** 1 Department of Medicine IV, Kaiser Franz Josef Hospital Vienna, Vienna, Austria; 2 Department of Virology, Medical University of Vienna, Vienna, Austria

## Abstract

**Introduction:** In Europe, country-specific treatment guidelines often do not advocate testing for integrase inhibitor resistance-associated mutations (IRAM) before initiation of first-line ART given the extremely low prevalence of mutations found in older surveillance studies. However, increased use of integrase inhibitors (INSTI) might have led to the emergence of treatment-limiting mutations in more recent years. We aimed to determine the prevalence of IRAM in Austria in the 5 years following introduction of INSTI and to analyze trends and factors associated with their detection.

**Methods:** Samples of antiretroviral treatment-naïve patients in Austria between 2008 and 2013 were analyzed for the existence of IRAM using bulk sequencing with published primers and drug-resistant mutation penalty scores (DPS, Stanford HIVdb algorithm) were calculated to estimate response to antiretroviral drugs. Demographic and virologic data including age, sex, viral subtype, drug resistance-associated mutations to PI and RTI were extracted from a database. Comparative statistics and logistic regression models were used to analyze risk factors for the occurrence of IRAM.

**Results:** A total of 303 samples were analyzed. Seventy-eight percent were male and median age was 36 years. HIV subtype B was most common (62%) followed by subtype C (12%). Overall prevalence of IRAM was 2.3%. Six percent had a DPS ≥10 for raltegravir or elvitegravir, respectively, indicating at least potential low-level resistance. One percent had a DPS ≥10 for dolutegravir. One major mutation was observed (F121Y) in a patient sample from 2012 leading to 5- to 10-fold reduced susceptibility to raltegravir and elvitegravir. Two patients carried the major accessory mutations E138K and G140A, respectively, which both lie on the Q148 pathway. No temporal trend was observed (p=0.16). Presence of any IRAM was not significantly associated with male sex (OR 0.78, 95% CI 0.14–4.30), older age (OR 0.98, 95% CI 0.91–1.05), calendar year (OR 1.28, 95% CI 0.80–2.03) or occurrence of any other drug resistance mutations (OR 1.41, 95% CI 0.16–12.23) in a multivariable logistic regression.

**Discussion:** Major primary IRAM are rarely found despite increasing use of INSTI in Austria but there is potential for reduced susceptibility to these drugs in selected patients. In the absence of predictors for occurrence of IRAM routine resistance testing seems prudent to avoid the consequences including accumulation of further mutations and therapeutic failure.

# Sub article: P363 Transmission of HIV-1 drug resistance in Tel Aviv, Israel, 2010–2015

**Published:** 2016-10-23

**Authors:** Dan Turner, Shirley Girshengorn, Adi Braun, Luba Tau, Ari Leshno, Danny Alon, Tal Pupko, Irena Zeldis, Natasha Matus, Simona Gielman, Svetlana Ahsanov, Inbal Schweitzer, Boaz Avidor

**Affiliations:** 1 Crusaid Kobler AIDS Center Tel Aviv, Tel Aviv Sourasky Medical Center, Tel Aviv, Israel; 2 Department of Cell Research and Immunology, Tel Aviv University, Tel Aviv, Israel

## Abstract

**Introduction:** The HIV-1 infected population in Israel is unique in its diversity. Until recently, the rate of transmitted drug-resistance mutations (TDRs) was relatively high mainly to NNRTIs. The prevalence of TDRs is regularly evaluated in treatment-naïve patients in Tel Aviv.

**Methods:** All blood samples obtained from treatment-naïve patients between 2010 and 2015 were analyzed for reverse transcriptase (RT) and protease resistance-associated mutations. Phylogeny on 614 sequences of subtypes A, B and C viruses (the main subtypes represented) was inferred by pol sequences.

**Results:** Viral sequences from 672 patients were tested. Men who have sex with men (MSM) was the major exposure risk category group (n=375), 76% among them were born in Israel, and 88% harbor subtype B viruses. Other groups include intravenous drug users (IVUs) (n=99); 78% of them were born in the former Soviet Union countries and 86% harbour subtype A viruses. The heterosexuals group is very heterogeneous and includes patients born in Israel, Ethiopian immigrants, former Soviet Union and worker immigrants or refugees mainly from Africa. The resistance rate decreased from 15.9% in 2010 to 5.9% in 2013 (p<0.05). In 2014 and 2015, it increased to 13.8% and 14.2% respectively. Same pattern was observed among MSM. Phylogenetic analysis of subtype B viruses supported clustered transmission among MSM. In 2010 to 2011 a cluster represented by the protease inhibitor mutation L90M was observed, and in 2014 to 2015 a cluster represented by NNRTIs (K103N)-associated mutations. In subtype A, a cluster among IVUs was found at 2012 during an outbreak, without resistance-associated mutations. However, a cluster harboring resistance mutation at position 103 in RT was observed in four MSM with subtype A virus. Subtype C viruses were not represented by specific clusters.

**Conclusions:** TDRs among patients followed in Tel Aviv were represented by clusters in MSM. These clusters were containing resistance-associated mutations to drugs less prescribed in recent years in Israel. Although the integrase inhibitor (InI) region is not analyzed routinely in treatment-naïve patients low rate of InI TDRs is reported in other studies. Regular assessment of genotyping in treatment-naïve populations including the integrase region is essential in order to understand the potential epidemiologic transmission of HIV clusters and effect of resistance on current ARV strategies.

# Sub article: P364 Development of T66I-mediated integrase inhibitor cross-resistance against elvitegravir under dolutegravir-containing first-line therapy

**Published:** 2016-10-23

**Authors:** Frank Wiesmann, Martin Däumer, Gudrun Naeth, Heribert Knechten, Patrick Braun, Jörg-Andres Rump

**Affiliations:** 1 HIV & Hepatitis Research, PraxenZentrum Blondelstrasse Aachen, Aachen, Germany; 2 Medical Diagnostics, Institute for Immunology and Genetics, Kaiserslautern, Germany; 3 Specialist in Internal Medicine, Medical Center Freiburg, Freiburg, Germany

## Abstract

**Introduction:** As second-generation integrase inhibitor (INI), dolutegravir (DTG) has shown a superior barrier to resistance as compared with profiles of raltegravir (RAL) or elvitegravir (EVG). Current findings suggest that resistance mutations against INIs extreme rarely occur under DTG-containing first-line ART. However, this case report unveils a possible development of a T66I-mediated cross-resistance against EVG under DTG first-line regimen.

**Methods:** A first-line treatment with lamivudine/abacavir, lopinavir and dolutegravir was initiated by a 44-year-old man with a diagnosis of HIV in November 2015 (CDC status B2, CD4 nadir 219/µL, HIV-1 RNA 350,000 copies/mL). Ultra-deep sequencing was performed by using population sequencing and ultra-deep sequencing (UDS, Illumina MiSeq) at baseline and at time of therapy failure. Resistance interpretation was estimated by using the HIV-Grade 12/2015, Stanford HIVdb version 7.0.1, Rega version 9.1.0 and the ANRS 25_09/2015 database. Viral load was quantified with Abbott Realtime.

**Results:** Before start of therapy, no resistance-associated variants could be detected neither by population nor by UDS in HIV protease, reverse transcriptase and integrase. After start of DTG first-line therapy, HIV viral load dropped from 300,000 copies/mL to 2400 copies/mL within 4 weeks of follow-up and was undetectable at week 8. CD4 cell counts increased from 219/µL to 479/µL (13.4%). However, 20 weeks after initiation of ART, HIV viral load increased to 105 copies/mL and maintained low viremic 4 weeks later at 112 copies/mL most likely due to inadequate adherence although plasma drug levels turned out to be above critical limits. More importantly, the development of the INI resistance mutation T66I was then verified by UDS showing a minority population of 36.1%. The variant T66I is a non-polymorphic mutation and reduces EVG susceptibility by ~15-fold while susceptibility to RAL or DTG is reported to be unaffected. There was no evidence for protease or reverse transcriptase resistance mutations at this time. Twenty-eight weeks after start of therapy the viral load decreased to undetectable levels without any changes.

**Conclusion:** Although being extreme rarely observed, INI-resistant HIV variants may also occur under DTG first-line treatment. The T66I alone does not necessarily limit the susceptibility to DTG itself but could be a first step of resistance development against DTG. It is reported that T66I confers high-level resistance against EVG and may also putatively lower the resistance barrier against RAL.

# Sub article: P365 Patterns of emergent resistance-associated mutations after initiation of non-nucleoside reverse-transcriptase inhibitor-containing regimens in Taiwan: a multicentre cohort study

**Published:** 2016-10-23

**Authors:** Chien-Yu Cheng, Yi-Ching Su, Mao-Song Tsai, Chia-Jui Yang, Wen-Chun Liu, Shu-Hsing Cheng, Hsin-Yun Sun, Chien-Ching Hung, Sui-Yuan Chang

**Affiliations:** 1 Department of Internal Medicine, Taoyuan General Hospital, Ministry of Health and Welfare, Taoyuan, Taiwan; 2 Department of Internal Medicine, National Taiwan University Hospital, Taipei, Taiwan; 3 Department of Internal Medicine, Far Eastern Memorial Hospital, New Taipei, Taiwan; 4 Department of Laboratory Medicine, National Taiwan University Hospital, Taipei, Taiwan

## Abstract

**Introduction**: Non-nucleoside reverse-transcriptase inhibitor (NNRTI)-containing ART remains the recommended first-line regimens for adults infected with HIV in many resource-limited countries. Increasing trends of resistance-associated mutations (RAMs) to NNRTIs have caused concerns about the effectiveness of the regimens in national programs in these regions. In this multicentre study, we aimed to investigate the incidence of emergent RAMs of HIV-1 to ARVs in HIV-positive adults who developed virologic failure to first-line NNRTI-containing ART in Taiwan.

**Materials and methods**: Between June 2012 and March 2016, ARV-naïve HIV-positive adults who initiated two NRTIs plus NNRTI at participating hospitals were included for analysis. Plasma HIV RNA load (PVL) was determined at baseline, and week 4 to 6 and subsequently every 12 to 16 weeks after ART initiation. Virologic failure was defined as a decrease of PVL <1.0 log10 copies/mL in 4 to 6 weeks of ART initiation; or PVL >200 copies/mL at 6 months of ART initiation; or confirmed HIV RNA ≥200 copies/mL after viral suppression (PVL <50 copies/mL). Population sequencing was used to detect RAMs. Detection of RAMs at baseline was performed retrospectively. RAMs were interpreted using the IAS-USA 2015 mutations list.

**Results**: During the 3.5-year study period, 1642 patients initiated NNRTI-containing regimens, and 454 (27.4%) had to switch first-line ART because of adverse effects or intolerance (n=323, 19.7%), retrospective detection of RAMs at baseline (41, 2.5%) and virologic failure (83, 5.1%). Virologic failure to two NRTIs plus nevirapine, efavirenz and rilpivirine was 9.7% (41/422), 4.2% (40/946) and 0.7% (2/277), respectively. In 68 patients, (3.8%) emergent RAMs were identified: 42 patients (62.7%) with NRTI RAMs; 28 (41.2%), one (1.5%) and 48 patients (71.6%) with NNRTI, protease inhibitors (PI) and any ARV RAMs, respectively, and 21 (31.3%) with resistance to two or more classes of ARV. The common emergent RAMs to NRTIs were K65R (25%), M184I (10.3%) and M184V (36.8%), and RAMs to NNRTIs included V90I (5.9%), K101E (5.9%), K103N (19.1%), V108I (7.4%), Y181C (11.8%) and G190A (5.9%) (Figure 1).

**Conclusions**: While a substantial proportion of the patients discontinued first-line NNRTI-containing regimens due to adverse effects, virologic response to NNRTI-containing regimens remained good in patients who were able to tolerate the regimens in Taiwan. Most common RAMs in those with virologic failure were related to exposure to tenofovir disoproxil fumarate, lamivudine, nevirapine and efavirenz.

**Figure 1:** Patterns of emergent resistance-associated mutations after initiation of three different non-nucleoside reverse-transcriptase inhibitor-containing regimens.

# Sub article: P366 Association of therapeutic failure with low-level viremia in HIV-infected patients in the Arevir/RESINA cohort in Germany

**Published:** 2016-10-23

**Authors:** Nadine Lübke, Alejandro Pironti, Elena Knops, Eugen Schülter, Björn Jensen, Mark Oette, Stefan Esser, Thomas Lengauer, Rolf Kaiser

**Affiliations:** 1 Institute of Virology, Heinrich-Heine-University, Düsseldorf, Germany; 2 Department of Computational Biology and Applied Al, Max Planck Institute for Informatics, Saarbrücken, Germany; 3 Institute of Virology, University of Cologne, Cologne, Germany; 4 Department of Gastroenterology, Heinrich-Heine-University, Hepatology and Inf, Düsseldorf, Germany; 5 Department of Inner Medicine, Hospital of the Augustinerinnen, Gastroenterology and, Cologne, Germany; 6 Department of Dermatology, University of Duisburg-Essen, Essen, Germany

## Abstract

**Introduction:** The German-Austrian guidelines for the treatment of HIV infection define therapeutic success as the reduction of the HIV-1 viral load (VL) below 50 copies/mL. Low-level viremia (LLV) is defined as repeated VL measurements between 50 and 200 copies/mL after initial therapeutic success. LLV has been previously associated with virologic failure (VF). Here, we provide an independent analysis of the association of LLV and other factors with VF.

**Materials and methods:** The Arevir database comprises clinical and virologic data of therapy-naïve and therapy-experienced HIV-1-infected patients in North Rhine-Westphalia, Germany, including the data of the RESINA cohort. We queried the Arevir database for patients who attained confirmed therapeutic success under ART and who experienced confirmed LLV thereafter. We constrained our query to therapies in which the VL was measured at least once every 24 weeks. We define VF as a confirmed VL greater than 200 copies/mL following therapeutic success. P-values were calculated with Fisher's exact and Wilcoxon rank sum test.

**Results:** The database query resulted in 2485 first-line and 3657 further-line therapies. LLV occurred in 294 (4.8%) of these therapies, specifically in 47 (1.9%) first-line and in 247 (6.8%) further-line therapies. The mean time to LLV was 27 months (σ=20.7), with no significant differences between first- or further-line therapies (p=0.4597). The majority of patients showing LLV were treated with PI-based therapies (165/294; 56%), followed by NNRTI-based regimes (76/294; 26%). Fifty-three out of 294 (18%) patients experienced VF after LLV with a median VL at failure of 472 copies/mL (range 203–116,590 copies/mL) after a mean LLV episode of 77.4 weeks (σ=68.0). The failure rate was increased in therapy-experienced patients (48/247; 19.4%), as compared with therapy-naïve patients (5/47; 10.4%; p=0.2129). There was no difference in VF between PI-based and NNRTI-based therapies regardless of the backbone (33/165; 20% and 13/76; 17.1%, respectively; p=0.6049). Among all drug classes, VF was never related to entry inhibitors, integrase inhibitors or the more recently approved compounds DRV, TPV and RPV (45/204 vs 0/83, respectively; p<0.0001).

**Conclusions:** The prevalence of LLV in patients on suppressive ART is low (4.8%). Nevertheless, 18% of patients with LLV experienced VF thereafter. The strongest predictor for VF after LLV was a treatment regimen exclusively containing drugs of the older generations. Therefore, episodes of LLV in patients treated with drugs with high potency and a high barrier to resistance are not predictive of VF.

# Sub article: P367 Drug resistance mutations (DRM) among pregnant HIV-positive women in the Duesseldorf University Hospital, Germany, 2009 to 2016

**Published:** 2016-10-23

**Authors:** Ulrike Elisabeth Haars, Nadine Luebke, Bjoern Erik Ole Jensen, Dieter Haeussinger

**Affiliations:** 1 Department of Dermatology and Venerology, University Hospital Essen, Essen, Germany; 2 Institute for Virology, Heinrich-Heine-University, Duesseldorf, Germany; 3 Department of Gastroenterology, Hepatology and ID, Heinrich-Heine-University, Duesseldorf, Germany

## Abstract

**Introduction**: cART has resulted in significant reduction of mother-to-child-transmission (MTCT) from 40% to 1 to 2% in the last 2 decades. Choosing an individualized cART is one key factor for successful suppression of viral load until delivery. Thus, drug resistance testing during pregnancy before cART initiation or in case of increasing viral load is recommended. The prevalence of drug resistance mutations (DRM) in pregnant women in Germany has not been characterized yet.

**Materials and methods**: Between January 2009 and March 2016, HIV drug resistance of all HIV-positive pregnant women was analyzed. Resistance testing was performed by using Sanger sequencing and next generation sequencing (NGS) by means of Illumina MiSeq-technology. Resistance interpretation was performed by the HIV-GRADE HIV-1-Tool (www.hiv-grade.de).

**Results**: Data of 85 HIV-positive pregnant women and 103 live births were analyzed. In 64/85 cases (75%), resistance testing was requested, with 61/64 successful analyses. The majority of patients were migrants (88%), 75% of these women had their origin in Sub-Saharan Africa (SSA). The majority of the patients were infected with non-B-subtypes (54/61, 88%), mainly 02_AG (23/61, 38%), followed by C (8/61, 13%) and A (7/61, 11%). In 14/61 (23%) resistance tests, DRM were found, in 9/14 due to ART history whereas five patients were therapy-naïve with presumably transmitted DRM (tDRM) or DRM due to immunological mechanisms like APOBEC3G/F (e.g. M184I, M230I) [1]. Five of 14 patients contained a two-class resistance against NRTI/NNRTI (Figure 1). Most common mutations were: M184VI (5/14), T215Y/F/N (4/14), Y181C (3/14) and K103N (3/14). NGS analysis showed additional mutations in 4/14 patients. No MTCT has been observed.

**Conclusions**: In 23% (14/61), of all HIV-positive pregnant women in our study DRM have been observed, in 8% tDRM (5/61). The prevalence of tDRM in pregnant women is lower than in general German population of HIV-positive individuals. Using resistance testing by NGS resulted in the identification of additional relevant DRM compared to Sanger. Considering the importance of viral load suppression in pregnancy and the limited amount of time to achieve this goal, the choice of cART should be optimal and take these mutations into account. Genotypic resistance testing should be therefore considered for all pregnant women to optimize the success of cART and hence prevent mother-to-child transmission.

**Figure 1:** Drug resistance mutations in pregnancy.

## References

[1] Noguera-Julian M, Cozzi-Lepri A, di Giallonardo F, Schuurman R, Daümer M, Aitken S M184I and M230I minority variants in ART-naïve patients are linked to APOBEC3G/F activity.

# Sub article: P368 Prevalence of HIV type 1 drug resistance mutations in treatment-naïve patients participating in the GARDEL study

**Published:** 2016-10-23

**Authors:** Maria Ines Figueroa, Patricia Patterson, Pedro Cahn, Jaime Andrade-Villanueva, José Arribas, José Gatell, Javier Lama, Michael Norton, Juan Sierra Madero, Omar Sued, Maria Jose Rolon

**Affiliations:** 1 Clinical Research, Fundacion Huesped, Buenos Aires, Argentina; 2 HIV Unit, Hospital Civil de Guadalajara Fray Antonio Alcalde, Guadalajara, Mexico; 3 Internal Medicine, IdiPAZ, Hospital Universitario La Paz, Madrid, Spain; 4 Infectious Unit, Hospital Clínic/IDIBAPS, University of Barcelona, Barcelona, Spain; 5 Asociación Civil Impacta, Salud y Educación, Lima, Peru; 6 Global Pharmaceutical Research and Development, AbbVie, Chicago, IL, USA; 7 Department of Infectious Diseases, Instituto Nacional de Ciencias Médicas y Nutrición Salvador Zubirán, Mexico City, Mexico

## Abstract

**Introduction**: Combination antiretroviral therapy has greatly reduced the rate of morbidity and mortality among HIV-1 infected patients. However, high mutation and recombination rates of HIV-1 lead to the emergence of various subtypes and drug resistance viruses, rendering first-line ARV therapy ineffective in many patients. The aim of this sub-study is to describe the prevalence of HIV-1 subtypes and the patterns of drug resistance mutations among ARV-naïve HIV-1 infected patients from six different countries participating in the GARDEL study [1].

**Materials and methods**: A total of 543 naïve patients from six countries (Argentina, Chile, Spain, Mexico, Peru and US) were screened between December 2010 and May 2012, and 534 HIV sequences were analyzed following the IAS-USA 2014 Drug Resistance Mutations Panel [2]. Genotypic assays performed at screening visit were: PhenoSense HIV assay (Monogram Biosciences, San Francisco, CA, USA), ViroSeq HIV-1 (ViroSeq HIV-1 Genotyping System version 2.0, Celera, Alameda, CA, USA) and TRUGENE^®^ HIV-1 Genotyping Assay (Siemens Healthcare Diagnostics, Munich, Germany), according to availability at each site.

**Results**: Of the 534 patients screened, 74% were Hispanic/Latino. Median time of infection at SCR was 10.5 months. CDC stage A: 82%. Of 450 viral subtypes available, the most frequent was subtype B in all three regions (Latin America (LA): 72% B, 17.6% BF; US/Mexico: 92% B; Spain: 91.2% B). A total of 113 samples (21.2%) had major resistant mutations; 22 samples (4.1%) had major protease mutations (M46I was the most common mutation: 1.5%), 85 samples (15.9%) had NNRTIs mutations (K103N/S was the most common mutation: 4.9%) and 17 samples had mutations to NRTIs (3.2%) and M41L (1.3%) was the most common mutation. PIs: only two patients had more than one major mutation (2/22). The more frequent minor mutations were: M36I/L/V (216/534), L63P (120/534), L10I/F/V/R (115/534) and K20R/M/I: 59/534. The global resistance analysis by regions showed 21% for LA, 22.8% for US/Mexico and 14.7% for Spain, being NNRTI resistance by regions 16.4%, 15.4% and 11.8% respectively. PI resistance was 3.1% for LA and Mexico/US and NRTI resistance was 3.1% for LA, 3.4% for US/Mexico and 2.9% for Spain. No Q151M, 69ss or K65R were identified.

**Conclusions**: In our study, we found a primary resistance rate of 21.2%, similar in LA and US/Mexico. Levels of NNRTI resistance are similar in the three analyzed regions, as previously reported in naïve populations, and reinforce the need of performing genotypic testing in ARV-naïve patients, especially in LA where the first-line therapy is still based on NNRTI drugs.

## References

[1] Cahn P, Andrade-Villanueva J, Arribas JR, Gatell JM, Lama JR, Norton M (2014). Dual therapy with lopinavir and ritonavir plus lamivudine versus triple therapy with lopinavir and ritonavir plus two nucleoside reverse transcriptase inhibitors in antiretroviral-therapy-naïve adults with HIV-1 infection: 48 week results of the randomised, open label, non-inferiority GARDEL trial. Lancet Infect Dis.

[2] Wensing AM, Calvez V, Günthard HF, Johnson VA, Paredes R, Pillay D 2014 update of the drug resistance mutations in HIV-1. Top Antivir Med.

# Sub article: P369 High prevalence of transmitted antiretroviral drug resistance in newly HIV-1 diagnosed Cuban patients

**Published:** 2016-10-23

**Authors:** Lissette Perez Santos, Liuber Yan Machado, Vivian Kouri Cardella, Hector Diaz, Carlos Aragones, Yoan Aleman, Eladio Silva, Consuelo Correa, Madelin Blanco de Armas, Jorge Perez, Marta Dubed, Yudira Soto, Nancy Ruiz, Celia Limia, Carmen Nibot, Neysi Valdés, Maria Ortega, Dania Romay, Yohana Baños, Barbara Rivero, Jorge Campos

**Affiliations:** 1 Virology Department, Tropical Medicine Institute “Pedro Kourí”, Havana, Cuba; 2 AIDS Research Laboratory, Mayabeque, Cuba; 3 Computer Science Department, Tropical Medicine Institute “Pedro Kourí”, Havana, Cuba; 4 Medical Care Department, Tropical Medicine Institute “Pedro Kourí”, Havana, Cuba

## Abstract

**Introduction**: The highly effective antiretroviral therapy has changed the natural history of HIV/AIDS, delaying the disease progression and improving the quality of life of the infected individuals. In treated HIV-1 population in Cuba, several factors might have contributed to high drug resistance levels such as prescription of suboptimal regimens containing non-boosted PI, prolonged exposure to failing therapies due to limited access to laboratory monitoring and limited options for antiviral drug substitutions if required. This might also result in the subsequent spread of drug-resistant strains. The performed studies in untreated population have shown high levels of HIV resistance to the antiretroviral therapy ranging from 12 to 21%. The aim of this study is to determine the levels of primary HIV drug resistance in newly diagnosed Cuban patients on a representative sample of the country.

**Materials and methods**: Demographic, clinical and laboratory data were collected from 263 recently HIV-1 diagnostic patients from April 2013 to April 2014. The HIV-1 pol gene was sequenced using Sanger sequencing and drug resistance was interpreted according to the World Health Organization surveillance drug-resistance mutations (SDRM) list, version 2009. HIV-1 subtyping was performed using the Rega subtyping tool, version 3.

**Results**: Experiments were successful for 189. The mean age at sampling was 33.5 years (17–74), 80.9% of the patients were men and the major transmission route was MSM (80.3%). 72.4% had recent infection and 38.6% were from Havana. The median value viral load was 58,000 RNA copies/mL and CD4 count value was 371 cells/mm^3^. In 17.4% (33/189) of the studied viruses, transmitted resistance mutations were detected, 22 (66.6%) were HSH, 26 (78.8%) were a recent HIV-1 infection, 13 (39.4%) were from Havana and 9 (27.2%) were infected with CRF19_cpx. Simple non-nucleoside mutants contributed the highest amount (45.5%), followed by double class resistance against NRTI and NNRTI (27.3%) and single mutants to the IP (12.1%). The most common mutation associated with resistance to NRTI was M184V (24.2%), for NNRTI was K103N (45.4%) and Y181C (30.3%) and for PI was D30N (6%).

**Conclusions**: This study confirms the high levels of resistance in untreated population, it demonstrates the commitment of first-line therapies used in the country and could put at risk future therapies. It highlights the need for studies to elucidate the factors that are influencing detected high levels of resistance in newly diagnosed population. It also shows the need for resistance testing in patients who are starting the therapy.

# Sub article: P370 Viroseq protocol optimized for the detection of HIV-1 drug mutations in patients with low viral load

**Published:** 2016-10-23

**Authors:** Fátima Monteiro, Gilberto Tavares, Marina Ferreira, Ana Amorim, Pedro Bastos, Carolina Rocha, Dina Hortelão, Claudia Vaz, Carmo Koch, Fernando Araujo, Rosário Serrão, António Sarmento

**Affiliations:** 1 Immunohemotherapy, Molecular Biology Center, São João Hospital, Porto, Portugal; 2 Infectious Diseases, São João Hospital, Porto, Portugal

## Abstract

**Introduction**: Genotypic resistance testing is paramount for the monitorization of the emergence of antiretroviral drug-resistant virus. The Viroseq HIV-1 genotyping system version 2.0 is an IVD assay for sequencing of HIV-1 from plasma but only feasible if the viral load is at least 1000 copies/mL. However, some patients have a persistent low HIV-1 viremia inferior to 1000 copies/mL, being resistance testing and antiretroviral therapy hampered by this. So, for their clinical management, resistance testing solutions must be made available [1]. With this regard, we developed an in-house assay adapting the Viroseq version 2.0 with a nested-PCR protocol.

**Materials and methods**: Blood samples from 36 patients on HAART with a viral load between 20 copies/mL and 1000 copies/mL (range 36–934 copies/mL; mean 357 copies/mL) were collected in K3EDTA and the plasma separated 6 hours after sampling and stored at −80°C. HIV-1 was concentrated by centrifugation of 1 mL of plasma at 24,000 g for 1 hour at 4°C. After removal of the supernatant, 1 mL of plasma was added and the sample thoroughly homogenized. RNA extraction was performed in the QIASymphonySP equipment from QIAGEN (Hilden, Germany) using the QIAsymphony Virus/Pathogen Mini Kit and an in-house protocol, rendering a final volume of 30 µL. The Viroseq protocol was performed according to the manufacturer instructions, followed by a nested-PCR protocol previously described by Mackie et al. [2]. The 50 µL PCR mix contained 0.5 µM of each primer, 1x Incomplete NH_4+_ Reaction Buffer (DFS-Taq DNA Polymerase, Bioron Life Science), 0.2 mM of deoxyribonucleotide, 2.5 units of DFS-Taq DNA Polymerase and 5 µL from the products of the first PCR. The PCR was performed on a Perkin Elmer PE9700 thermocycler and consisted on an initial denaturation for 5 minutes at 95°C, followed by 40 cycles of 95°C for 30 seconds; 55°C for 30 seconds, 72°C for 120 seconds and a extension at 72°C for 7 minutes. PCR products were sequenced on the 3130xl DNA Analyzer (Applied Biosystems) and analyzed in Viroseq version 2.8.

**Results**: Sequencing and drug resistance testing was successful in 70% (9/13) of the samples with a viral load 36 to 200 copies/mL; in 93% (13/14) of the samples comprising 200 to 500 copies/mL and in 100% (9 /9) of the samples with 500 to 1000 copies/mL.

**Conclusion**: Genotypic resistance testing is essential for the monitorization of the emergence of antiretroviral drug-resistant virus being necessary for the development of assays for patients with low viral loads.

## References

[1] Ryscavage P, Kelly S, Li Z, Harrigan PR, Taiwo B (2014). Significance and clinical management of persistent low-level viremia and very-low-level viremia in HIV-1-infected patients. Antimicrob Agents Chemother.

[2] Mackie NE, Phillips AN, Kaye S, Booth C, Geretti AM (2010). Antiretroviral drug resistance in HIV-1-infected patients with low-level viremia. J Infect Dis.

# Sub article: P371 The role of presepsin (sCD14-ST) as an indirect marker of microbial translocation and immune activation

**Published:** 2016-10-23

**Authors:** Paola Columpsi, Valentina Zuccaro, Serena Cima, Paolo Sacchi, Raffaele Bruno

**Affiliations:** 1 Infectious Diseases, IRCCS Policlinico San Matteo Pavia, Pavia, Italy

## Abstract

**Introduction**: Presepsin, a newly discovered soluble fragment of CD14, has been studied as a sepsis biomarker. The mechanism of its secretion is involved in the TLR4 activation cascade and it is related to mCD14 and sCD14, which are monocyte activation markers, indirectly representing the presence of bacterial translocation. Therefore presepsin could be employed as an immune activation marker, and it could allow for the estimation of bacterial translocation rates [1]. The aim of this study was to assess the correlations between presepsin serum concentration and bacterial translocation, immune activation and fibrosis markers in subjects with HIV and hepatitis C virus (HCV) mono-infections and in HIV/HCV co-infection, compared to healthy controls.

**Materials and methods**: This cross-sectional study included patients with HCV and HIV mono-infections, HIV/HCV co-infection and healthy controls (20 subjects/group). Peripheral blood was analyzed to determine the levels of presepsin, Forkhead box 3 (Foxp3+) T cells, TGF-β1, CD14 (soluble and surface isoforms), IL-17 and bacterial translocation products. These measurements were correlated to the severity of liver fibrosis, measured with the FIB-4 score and transient elastography.

**Results**: Presepsin concentration was significantly higher in the HIV patients (HIV mono-infected and HIV/HCV co-infected). The same group showed increased levels of sCD14 and mCD14, expression of immune activation. Statistical analysis shows a significant correlation between presepsin and both forms of CD14 only in HIV/HCV group, where the percentage of bacterial translocation and chronic inflammation is high, as shown by the significant increase in bacterial DNA levels, sCD14, mCD14 and IL-17. Presepsin is associated with FIB-4 values in the HCV group.

**Conclusions**: Presepsin is a biomarker of chronic immune activation, as demonstrated by its correlations with sCD14, mCD14 and CD4+CD25+Foxp3+ lymphocytes, particularly in HIV infection. Its concentration is correlated to liver fibrosis markers, such as FIB-4, particularly in HCV mono-infected patients. Considering presepsin and a direct correlation between the levels of fibrosis and an inverse correlation with Treg cells in this group, the low levels of Treg cells may be involved in increasing the state fibrosis in chronic HCV patients.

## References

[1] Yaegashi Y, Shirakawa K, Sato N, Suzuki Y, Kojika M, Imai S (2005). Evaluation of a newly identified soluble CD14 subtype as a marker for sepsis. J Infect Chemother.

# Sub article: P372 CRF19_cpx variant emergence in a cluster in naïve patients of southern Spain: clinical and phylogenetic characterization

**Published:** 2016-10-23

**Authors:** Carmen-María González-Domenech, Isabel Viciana, Marisa Mayorga, Rosario Palacios, Javier de la Torre, Francisco Jarilla, Manuel Castaño, Alfonso del Arco, Manuel Márquez, Encarnación Clavijo, Jesús Santos

**Affiliations:** 1 UGC Enfermedades Infecciosas y Microbiología, Hospital Virgen de la Victoria, Málaga, Spain; 2 UGC Enfermedades Infecciosas, Hospital Carlos Haya, Málaga, Spain; 3 Medicina Interna, Hospital Costa del Sol, Marbella, Spain; 4 Medicina Interna, Hospital Antequera, Antequera, Spain

## Abstract

**Introduction:** HIV CRF19_cpx has been described as a highly pathogenic recombinant from Cuba [1]. Furthermore, these infections are typically associated to higher viral load (VL) at diagnosis and rapid progression to AIDS [2]. Here, we describe the emergence of this CRF19_cpx variant in southern Spain, clustering in men having sex with men (MSM).

**Materials and methods:** The study was undertaken at the Virgen de la Victoria Hospital, a reference centre for the analysis of HIV-1 genotypic drug resistance in Malaga (Spain). The subtype for each FASTA sequence provided was assigned through REGA version 3.0. Sequences consigned as a CRF19_cpx variant were confirmed by phylogenetic analysis with other 195 reference sequences retrieved from LANL. Protease and reverse transcriptase (RT) genes were aligned by ClustalX and the phylogenetic reconstruction inferred by maximum likelihood method (RAxML). The reliability of the clades was supported on bootstrapping, with 1000 replications. For analysis of RT and protease resistance mutations, Standford algorithm version 7.1.1 was used. Additionally, we collected epidemiologic, clinical and immunologic data.

**Results:** Genotypic test was performed in 2298 naïve patients from four hospitals during 2011 to 2016, finding the CRF19_cpx variant in 49 of them (2.1%). These recombinants, except one, were clustered together (bootstrap=93%), with phylogenetic relation to CRF19_cpx from Israel, Bulgaria and Cuba. Seven well-supported subclusters with different number of patients were also found: A, D, F and G (n=2); B and E (n=8); and C (n=3) (Figure 1). Non-nucleoside RT inhibitor G190A resistance mutation was found in 24 patients (48.9%), among them, clades C, D, E and F. All the patients were MSM, 21 of them (42.8%) had a prior negative HIV test, with a median time of seroconversion of 15 months (IQR 10.7–22.8). All were Spanish, except for two patients from Argentina and one from France. The mean age was 35.0 years (26.3–41.5), the initial CD4 count was 361/µL (254–416) and VL 4.9 log (4.5–5.4), being lower in patients with G190A mutation (4.6 vs 5.1, p=0.02). Three cases of AIDS (6.1%) and one death occurred (acute myocardial infarction). All the patients treated with first-line combination ART responded.

**Conclusions:** CRF19_cpx variant has emerged affecting MSM naïve patients from southern Spain; all cases but one are related to a local cluster. Half of patients showed the G190A resistance mutation. Unlike previous studies, the variant from Malaga seems less pathogenic, with few cases of AIDS and excellent response to ARV.

**Figure 1:** Phylogenetic tree with CRF19_cpx reference sequences, and subtree containing the clustering of our 49 patients obtained by maximum likelihood. Only support values ≥70% are shown.

## References

[1] Casado G, Thomson MM, Sierra M, Nájera R (2005). Identification of a novel HIV-1 circulating ADG intersubtype recombinant form (CRF19_cpx) in Cuba. J Acquir Immune Defic Syndr.

[2] Kouri V, Khouri R, Aleman Y, Abrahantes Y, Vercauteren J, Pineda-Peña AC (2015). CRF19_cpx is an evolutionary fit HIV-1 variant strongly associated with rapid progression to AIDS in Cuba. EBioMedicine.

# Sub article: P373 One-step real-time PCR for HIV-2 group A and B RNA plasma viral load in LightCycler 2.0

**Published:** 2016-10-23

**Authors:** Pedro Bastos, Fátima Monteiro, Gilberto Tavares, Ana Amorim, Marina Ferreira, Dina Hortelão, Carolina Rocha, Claudia Vaz, Carmo Koch, Fernando Araujo, Rosário Serrão, António Sarmento

**Affiliations:** 1 Immunohemotherapy, Molecular Biology Center, São João Hospital, Porto, Portugal; 2 Infectious Diseases, São João Hospital, Porto, Portugal

## Abstract

**Introduction**: Although with a lower prevalence than HIV-1, HIV-2 is responsible for localized epidemics, being Portugal the non-African country with the greatest expression of the infection. Clinical management of the infection is hampered by the lack of validated commercial RNA viral load assays, thus there an in-house development using the available equipment is mandatory.

**Materials and methods**: HIV-2 was confirmed by Innolia^©^ (Innogenetics, Gent, Belgium). Blood samples were collected in K_3_EDTA and the plasma separated 6 hours after sampling and stored at −80°C. The BIOQ HIV-2 RNA group A quantification panel (Biocentric) was used as an external standard. RNA extraction was performed from 1000 µL of plasma in the QIASymphonySP equipment from QIAGEN (Hilden, Germany) using the QIAsymphony Virus/Pathogen Mini Kit and an in-house protocol, rendering a final volume of 60 µL. RNA from the samples and standards was isolated under the same conditions. The protocol was based on the previously described by Avettand-Fenoel et al. [1]. The forward and reverse primers for the LTR region were 5'TCTTTAAGCAAGCAAGCGT GG-3 and 5’-AGCAGGTAGAGCCTGGGTGTT-3 and for the gag region F3 5’-GCGCGAGAAACTCCGTCTTG-3 and R1 5’-TTCGCTGCCCACACAATATGTT-3. The probe for the LTR region was 5'FAM-CTTGGCCGGYRCTGGGCAGA-BHQ1-3 and for the gag region S65GAG2 5'FAM-TAGGTTACGGCCCGGCGGAAAGA-BHQ1-3. The one-step RT-PCR was performed on the LightCycler 2.0 (Roche Diagnostics, Mannheim, Germany). The LightCycler RNA Virus Master Kit from Roche (Roche Molecular Biochemicals) was used. The 20 µL reaction mixture contained 0.5 µM of each primer, 0.25 µM of each probe, 0.4 µL of Enzyme Blend and 7.5 µL of the isolated RNA. The thermocycling consisted of 10 minutes at 60°C and 60 seconds at 95°C, followed by 50 cycles of 95°C for 5 seconds, 60°C for 50 seconds and 72°C for 10 seconds.

**Results**: The standard curve generated by the LightCycler software (version 4.05) presented an efficiency of 2.079 and an error of 0.0657. This RT-PCR provides an increment in sensibility to 50 copies/mL and the detection of HIV-2 B subtypes. In comparison to the RT-PCR previously used in routine, no deviation higher than 0.5 log was found in the testing of 21 clinical samples and several dilutions of the NIBSC HIV-2 NIH-Z strain.

**Conclusions**: This assay allows us to quantificate HIV-2 A and B subtypes with satisfactory sensibility and linearity, supporting the clinical management of the infection.

## References

[1] Avettand-Fenoel V, Damond F, Gueudin M, Matheron S, Mélard A, Collin G (2014). New sensitive one-step real-time duplex PCR method for group A and B HIV-2 RNA load. J Clin Microbiol.

# Sub article: P374 The association between high pre-HAART CD8 counts and poorer immunological outcome following antiretroviral therapy

**Published:** 2016-10-23

**Authors:** Chun Kwan Bonnie Wong, Ngar Sze Wong, Shui Shan Lee

**Affiliations:** 1 Special Preventive Programme, Department of Health, Hong Kong Special Administrative Region, Hong Kong, Hong Kong; 2 Stanley Ho Centre for Emerging Infectious Diseases, The Chinese University of Hong Kong, Shatin, Hong Kong

## Abstract

**Introduction**: Nadir CD4 counts, advanced age and hepatitis C co-infections are known predictors of poorer immune recovery following HAART. As a high CD8 count was associated with inflammatory non-AIDS-related clinical events, it could be another useful marker for prognostic monitoring.

**Methods**: Anonymous clinical data from Integrated Treatment Centre, the largest HIV service in Hong Kong, were accessed. Adult HIV+ patients with available negative HIV testing result within 3 years before HIV diagnosis were targeted for the collection of the following data: (a) CD4, CD8 and viral loads at all time points of testing, (b) timing of AIDS diagnosis, as appropriate, (c) antiretroviral treatment date and regimens. Cumulative viral load was estimated. All eligible patients were divided into two groups by their pre-HAART CD8 counts, that is, either >800/L or ≤800/L, followed by multivariable logistic regression.

**Results**: As of the end of 2012, records of 199 treatment-naïve patients (median age 36) who had been on HAART continuously for ≥4 years were analyzed. A majority (90%) were male with men who have sex with men (MSM) accounting for 58% of the study population. Their median interval from diagnosis to the latest assessment was 12.7 years. Either a protease inhibitor-based (70%) or non-nucleoside reverse transcriptase inhibitor-based (30%) regimen was prescribed. The pre-HAART median CD4 and CD8 counts were 158/L and 790/L, which were positively correlated (r=0.51, p<0.001). The median treatment duration was 78 months (IQR 57–112). At the end of a 4-year observation period, about half (56%) had CD4 reaching 500/L or above, of which 45 (40.5%) gave a CD4:CD8 ratio of ≥0.8. After adjusting for baseline CD4, patients with low pre-HAART CD8 (≤800/L) had a higher chance of achieving a higher CD4 count (aOR 1.002, 95% CI 1.00–1.004). A low pre-HAART CD8 was also associated with optimal immune outcome defined as a CD4 count ≥500/L in conjunction with a CD4:CD8 ratio ≥0.8, with an increased odds (aOR 6.64, 95% CI 2.53–17.40) after adjusting for pre-HAART CD4. Cumulative viral load from the time of estimated seroconversion was not associated with pre-HAART CD8 count.

**Conclusion**: A pre-HAART CD8 count of >800/L gave a high odds of poorer immune outcome. Pre-HAART CD8 count is an independent predictor of an outcome measure comprising CD4 count and CD4:CD8 ratio. While CD4 is a useful prognostic marker, the strength of prediction increased with the addition of baseline CD8 count using a cutoff of 800/mL.

# Sub article: AUTHOR INDEX

**Published:** 2016-10-23

**Table:** 

**A**	
Abbate, I	P053
Abe, E	P081
Abeli, C	P235
Aboromman, M	P236
Aboud, M	P035, P210
Abram, M	P043, P093
Abram, ME	O125
Abrescia, N	P021
Abreu, I	P297
Abreu, M	P299
Abreu, RC	P041
Achilles, S	P302
Acsai, M	P359
Adams, T	P209
Adepoju, A	P019*
Adland, E	P002
Aebi-Popp, K	P001*, P254*
Afdhal, N	P259
Afonina, L	O335B
Afonso, C	P343
Agaba, P	P339
Agarwal, K	P259
Agbaji, O	P339
Agher, R	P023
Aghokeng, A	O122
Agüero, F	P050, P051
Agurto, C	P242
Ahmed, N	P338
Ahsanov, S	P363
Airoldi, G	P136, P141
Aitken, C	P064
Ait-Mohand, H	P044, P217
Ajassa, C	P160
Alba, L	P216, P190
Alcalde, M	P294
Alcantarini, C	P291, P304
Alcaraz, A	P178
Alcaraz, B	P294
Alcaraz, M	P265, P274, P280, P284
Aldámiz-Echevarría, T	P265, P274, P280, P284
Aldir, I	P029, P122, P292, P296
Aleixo, MJ	P267
Alejos, B	O331, P165*
Aleman, Y	P369
Al-Harbi, M	P006
Alimohammadi, A	P027, P061*, P065, P067, P238
Allegrini, F	P089
Almeida, F	P132, P344
Alon, D	P108, P363
Altice, F	P139
Alvarez, JC	P081*
Alvarez, M	P196
A´lvarez-A´lvarez, B	P265, P274, P284
Alves, J	P122
Alves, L	P270, P295
Alves, S	P096
Amara, A	P307
Amaral, DC	P222
Amat, K	P081
Ambaraghassi, G	P134*
Ambrosioni, J	P050, P051*
Amendola, A	P053
Amiel, C	O332
Ammassari, A	P053, P216, P315, P232, P237*, P346
Amorim, A	P370, P373
Ampofo, W	P350
Anand, T	P014*, P322*
Ananworanich, J	P014, P322
Ancona, G	P100
Anderson, J	O215
Andrade-Villanueva, J	P368
Andreoni, M	P253
Andreotti, M	P110
Andreu-Crespo, A	P070
Andrinopoulou, E	O333
Angarano, G	P156, P278, P287
Angeli, G	P235
Angioni, G	P141
Ankiersztejn-Bartczak, M	P020, P327
Antalis, E	P040
Antequera, I	P152
Antinori, A	O121, P021, P048*, P053, P086, P101, P136, P141, P156, P190, P216, P232, P250, P315, P346
Antoniadi, C	P320
Antoniadou, A	P040, P162, P172, P227, P354, P360, P361
Antunes, A	P215
Antunes, I	P029, P292, P296
Anzoua-Kacou, J-B	P182
Apornpong, T	P014, P322
Appiah, LT	P059, P219
Aragão, F	P154
Aragones, C	P369
Araujo, F	P370, P373
Arazo, P	P088
Arez, L	P007, P042
Argenteri, B	P099
Argyropoulou, M	P040
Arias, A	P276*, P280
Ariaudo, A	P291, P304
Arida, K	P179
Arluck, J	P245
Arnbjerg, C	P123
Arnould, B	P138
Arponen, S	P126*, P274
Arribas, J	O331*, P368
Arribas, JR	P198
Arruda, EAG	P003, P206
Artigues, F	P051
Ascai, M	P174
Ascenção, B	P187*, P335
Asensi, V	P165
Asher, I	P135
Ashida, M	P312
Assoumou, L	P081
Atzori, C	P213
Atzori, C	P317
Audagnotto, S	P213
Avidor, B	P108, P363
Avihingsanon, A	O334
Avramovic, G	P127
Awosusi, F	P006*
Ayarza, R	P242
Ayerbe, CG	P116, P203, P293, P329, P328*
Aylott, A	P035
Azevedo, F	P007
Azevedo, T	P296
**B**	
Back, D	P094
Bado, G	O122
Baecke, C	P318
Baggott, A	P148*
Bai, F	P156, P279
Bailón, L	P285
Baiocchini, A	P237
Bakker, J	P229
Baldelli, F	P021, P235
Baldelli, S	P309
Baldin, G	P097, P106*
Baldin, GM	P190
Baldini, F	P346
Balestra, P	P216, P232
Ballester, J-E	P281
Balogh, A	P060, P117
Bandera, A	P086, P190
Banica, L	P283*
Bannert, N	P353
Baños, Y	P369
Baptista, P	P183
Baptista, T	P029, P292, P296
Baraia-Etxaburu, JM	P128
Barbarini, G	P099
Barbaro, F	P298
Barbás, MG	P357
Barber, M	P251
Barco, A	P291*, P304
Barnes, J	P301
Barros, C	P265, P280, P284
Barros, H	P017
Barrufet, P	O331
Bartmeyer, B	P353
Bastos, P	P370, P373
Batan, I	P283
Battegay, E	O211*
Battegay, M	O224
Bauer, S	P177*
Baugh, B	P310*
Baumgarten, A	P046
Bax, H	O333
Bayés-Genis, A	P180
Bedimo, R	P170
Begovac, J	P124, P324
Béguelin, C	P264*
Bekker, L-G	KL3*
Belaunzarán-Zamudio, P	P340*
Belda, L	P265
Bell, D	P272
Bellacosa, C	P230, P234
Bellagamba, R	P232, P315
Bellcave, P	O332
Bellet, J	P081
Beloukas, A	P059, P219
Belvisi, V	P349
Benatti, S	P083
Benítez, L	P274, P284
Benitez-Gutierrez, L	P276
Benjamins, L	P033
Berenguer, J	P165, P265, P274*, P280, P284*
Bergamasco, A	P290
Bernal, E	P294
Bernardino, JI	P198
Bernasconi, E	P254, P264
Berrocal, AG	P087
Bessong, P	P057
Betkova, S	P270, P295
Bezalel-Rosenberg, S	P135
Bhagani, S	P248
Bhagani, S	P301
Bianco, GD	P033
Biasi, L	P290
Bichko, VV	P024
Bickel, M	P130
Bielec, D	P120
Bierau, J	P229
Bierman, W	O333
Bilinski, S	P039*
Bini, T	P100
Birkholtz, L-M	P028
Bisbal, O	P271
Bissett, B	P064
Bissio, E	P357*
Blanc, C	P023
Blanch, J	P228
Blanch, K	O213
Blanche, S	P034
Blanco, J	O213, P228
Blanco, J	P180
Blanco, JR	P282
Blasco, M	P126
Blaxhult, A	O114
Bleak, S	P320
Bludzin, W	P120
Blum, L	P217
Boasso, A	P208
Boccino, S	P012
Bociaga-Jasik, M	P120
Bocket, L	O332
Boffito, M	O215, P094, P209, P307, P308, P305*, O315
Bogner, J	O335B, P189
Bohr, U	P130
Bolton, M	P209
Bonafont-Pujol, X	P070
Bonfanti, P	P230, P234, P263
Bonjoch, A	P180
Bonnet, F	O212, O224
Bonora, S	P090, P213, P250, P291, P304, P305, P308
Bordoni, V	P216, P346
Boretzki, J	P060
Borg, P	P314
Borges, A	P189*
Borges, F	P029, P122, P292, P296
Borghetti, A	O121, P106
Borghi, V	P277
Borrego, P	P351
Borstein, R	P247
Bossini, N	P223
Botelho-Nevers, E	P151, P225
Bouchaud, O	P044
Boucher, C	O333
Bouzas, MB	P357
Bower, M	P189, P194
Bracchi, M	P094, P194, P209
Bradley, C	P125*
Brainard, D	P259
Brau, N	P259
Braun, A	P363
Braun, D	P264
Braun, P	P364
Bravo, I	P085
Bravo, J	P178
Bravo, SP	P316
Brefort, S	P217
Bremer, V	P353
Bress, A	P170, P175
Brianese, N	P223
Briganti, E	P089
Brighi, S	P089
Brito, AP	P187, P335
Brodt, H-R	P036, P039
Brofman, T	P108
Brogueira, P	P292*
Brown, K	P063*
Brun, A	P044
Brunetta, J	P359
Bruno, G	P278, P287*
Bruno, R	P253, P263, P371
Brust, J	P130
Brust, J	P133
Büch, J	P351
Buchanan, A	P035
Bucher, H	P264
Buda, S	P286
Budd, D	P072*
Bugliosi, E	P163, P158
Bukusi, E	P350
Bulteel, N	P272
Burchell, A	P191
Burke, M	P135
Burns, F	P058
Bush, S	O314
Buzibye, A	P142
Bygrave, H	O134*
Byrne, R	P194
**C**	
Cabo, J	P270*, P295*
Cabras, O	P095
Caby, F	P023*
Caceres, G	P241
Cadieux, M	P011
Cahn, P	O334*, P368
Cai, W	O336
Caixas, U	P075, P215*, P313*,
Calcagno, A	P158, P163, P213, P304*
Caldas, C	P344
Caldeira, L	P183, P343
Calin, R	P023
Calistru, P	P150
Callebaut, C	O123*, P043*
Callegaro, A	P068, P186
Callens, S	P167
Calmy, A	P189, P254
Calonge, ME	P328
Calvet, A	P070
Calvez, V	O332, P023
Calvo, MJ	P274, P284
Calvo, MJ	P280
Calvo, M	P265
Calza, L	P230, P234
Camacho, RJ	P351
Cambiano, V	O312*
Campagna, M	P160, P269
Campbell, T	P314
Campoa, E	P007*, P042
Campos, J	P369
Campos, M	P292
Campos, MJ	P017, P029, P296
Campos, N	P078
Campos, P	P226
Canalejo, E	P274
Cancellieri, C	P089
Cano, A	P178
Cao, F	P119
Cao, H	O124
Capetti, A	P106
Capetti, AF	P097*, P099*
Capobianchi, MR	P053, P237, P346
Capozzi, MC	P294
Caputo, SL	P021
Caramello, P	P253
Cardella, VK	P369
Cardellino, C	P213
Cardinal, H	P134
Cardoso, O	P270, P295
Cardoso, S	P270, P295
Carenzi, L	P230, P234
Carli, F	P056, P155, P158, P163, P164
Carlo Federico Perno, CF	P216
Carmena, J	P281
Caro-Vega, Y	P340
Carrasco, I	P276
Carta, S	P216, P346
Carvalho, A	P267
Carvalho, AC	P297
Casabianca, A	P100
Casado, JL	P111
Casado, JL	P103, P116, P288, P289, P293, P328
Casana, M	P279
Casari, S	P223, P290
Cascio, A	P173
Casella, I	P187
Casella, MI	P132, P335*
Casella, MI	P267
Cassells, Y	P064
Cassidy, R	P156, P157
Cassola, G	P243, P250
Castagna, A	O121, P021, P048, P090, P190
Castaño, M	P054, P372
Castelli, F	P223, P290
Castelnuovo, B	P142, P352, P356
Castelnuovo, F	P290
Cattaneo, D	P090, P309, P303*
Cattelan, A	P298
Cattelan, AM	P090, P158, P163
Cattelan, AM	P097
Cauda, R	O121, P086, P136
Cavassini, M	P197, P254, P341
Cavassini, M	P264
Cavinato, S	P090, P298*
Cazanave, C	O212*
Cazorla, C	P225
Cazzaniga, F	P100
Ceausu, E	P150
Cebas, AL	P316
Ceccherini-Silberstein, F	P216, P263, P346
Cecilio, A	P282
Ceia, F	P297
Celesia, B	P163
Celesia, BM	P097, P099, P158, P230, P234
Cenderello, G	P097, P243
Cervero, M	O331, P265, P311
Cesari, M	P155
Cevik, M	P147*, P342*, P148
Chabrol, A	P044
Chadwick, D	P059, P218, P219
Chan, KL	P221
Chan, PCD	P221, P319
Chang, B	P174, P359
Chang, C-J	P207
Chang, H-Y	P112*, P201, P266
Chang, H-Y	P047
Chang, L-H	P013, P246
Chang, S-C	P013, P112, P255, P257, P266, P268, P275
Chang, S-Y	P091
Chang, S-Y	P257, P266, P365
Chaponda, M	P212
Chappell, C	P302*
Charest, L	P011
Charpentier, C	O332, P095*
Charrière, E	P151
Chassany, O	P140, P236
Chatzidimitriou, D	P361
Chaves, HL	P206
Chen, B	P302
Chen, G-J	P255, P268*
Cheng, A	O124, O125, P093
Cheng, C-Y	P091*, P365*
Cheng, S-H	P091, P365
Chetchotisakd, P	P045
Cheung, CYS	P221
Chilengi, R	P177
Chinello, P	O121
Chini, M	P162, P354, P360, P361
Chirianni, A	P101, P141
Chirico, C	P223*
Chkhartishvili, N	O233*, O114
Cholewinska, G	P247
Chou, T-C	P207
Christaki, E	P076
Christensen, S	P153, P159*
Chrysos, G	P162
Chrysos, G	P354, P360, P361
Chuang, Y-C	P255, P257, P268
Ciaffi, L	O122*
Ciardi, MR	P347, P348
Cicalini, S	P315
Cima, S	P097, P371
Cingolani, A	P190*, P048
Clarke, A	O125, P045, P209
Clavijo, E	P152, P372
Clementi, E	P303, P309, P317
Clotet, B	P070, P085
Clotet Sala, B	P180
Cobos, N	P294
Cockett, M	P022
Coelho, P	P183
Coffie, P	P182
Cogliandro, V	P100
Cohen, J	P072
Cohen-Poradosu, R	P108
Cohn, S	P302
Colafigli, M	O121, P105, P106
Colella, E	P107
Coletta, E	P249
Coll, J	P070
Collier, J	P301
Collins, B	P324
Colomba, C	P173
Colombo, G	P068
Columpsi, P	P371*
Comi, L	P186
Compston, J	P169
Conde, MS	P203
Conde, MS	P329
Contreras, G	P033*
Conway, B	P027, P061, P065, P067, P238
Cooper, V	P161
Copenhaver, M	P071, P139
Corbin, V	P034
Cordier, L	P234
CoRIS, C	P165
Correa, C	P369
Correa, EA	P003
Correia, M	P215, P313
Corsilli, D	P134
Cortés, C	P340
Cortes-Martins, H	P114
Corti, N	P142
Cossu, MV	P097, P099, P106
Costagliola, D	P081
Cota-Medeiros, F	P343*
Cotter, A	P127, P169
Coutinho, D	P145
Cozzi, V	P303
Cozzi-Lepri, A	P021, P156, P253, P263
Crabtree-Ramírez, B	P340
Crandall, B	P331
Crauwels, H	P032*, P310
Cripps, S	P301
Cristini, G	P290
Crofoot, G	O124
Crook, J	P170, P175
Crouzat, F	P174*, P359
Crovato, E	P286
Croxford, S	P323
Cruces-Moreno, MT	P084
Cruciani, M	P176*
Crusells, MJ	P088
Cruz, I	P296
Cruz-Martos, E	P265, P280
Cruz-Martos, E	P274, P284
Cudolá, A	P357
Cuervas-Mons, V	P276
Cuevas-Tascon, G	P265
Cunha, JS	P267
Cuomo, G	P277*
Curran, A	O331
Cursley, A	P305
Curtis, L	P210
Custodio, J	P306*
**D**	
D’Abramo, A	P347
D’Amico, R	P260
D’Angelo, C	P279
d’Armini Monforte, A	O224
d’Arminio Monforte, A	P021*, P048, P100, P190, P250, P253, P263, P279
D’Arminio Monforte, A	P101, P156
D’Avino, A	P086, P105
D’Avolio, A	P291, P304, P305, P308
d’Ettorre, G	P110, P160, P273, P347, P348
D’Ettorre, G	O121, P269
da Cunha, JS	P211
Da Ponte, DV	P206
da Silva, ARD	P164
da Silva, MG	P222
Daamen, C	P324
Daar, E	O125, P314
Dabis, F	O212
Dadem, N	P339
Daeumer, M	P252
Dahl, M-L	P031
Daikos, G	P179
Daikos, G	P354, P360, P361
Dantas, RS	P003
Darakjian, V	P320
Darchy, N	P217
Darin, K	P302
Darkwa, J	P028
Darling, K	P197*, P341
Das, M	O123, P045, P314
Däumer, M	P364
Davies, M-A	P177
Davis, J	P022
Davis, K	P073
Day-Weber, I	O315
de Armas, MB	P369
de Bobadilla, EL	P329
De Bona, A	P279
de Brito Neves, AP	P206
De Francesco, D	O215*, P155*
de Guzmán, MT	P274
de Jesus, S	P088*
de Jesús, SE	P082*
de Jesus, SE	P084*
De Jesus, SE	P196
de la Fuente Moral, S	P102*
de la Fuente, B	P166
de la Peña, M	P128
de la Torre, J	P054, P088, P372
de Lacerda, AP	P183*
de Lazzari, E	O213, P050, P051, P228
de Leuw, P	P036
De Leuw, P	P039
De Luca, A	O121, P048, P086, P105, P106, P250, P263
de Magalhaes, FB	P222*
De Marco, R	P106
De Mendoza Fernandez, C	P102
De Mendoza, C	P276
De Miguel Buckley, R	P087
De Miguel, J	P274
de Munain, JL	P128
De Nicolò, A	P304
de Queiroga, MF	P206
de Sales, CY	P003
de Santiago, AD	P102
De Socio, G	P158, P163, P230, P234*
De Socio, GV	P235*, P097, P099
De Truchis, P	P081, P217
De Vries-Sluijs, D	O333
De Wet, J	O124
de Wit, S	O224
De Wit, S	P131*, P137*, P010, P167
Dedes, N	P324
Deeks, SG	O321*
Defferrière, H	P217
DeJesus, E	O124
del Arco, A	P372
del Campo, S	P329*
Del Campo, S	P111, P288, P293, P311, P328
del Forge, M	P167
Del Nonno, F	P237
Del Pin, B	O121
del Pino, GF	P084
Delaporte, E	O122
Delaugerre, C	O332, P044, P217
Dell’Acqua, R	P278, P287
Dell’Omo, M	P235
Delpech, V	P324
Demeester, R	P131, P137
Denarie, M	P245
Denman, J	P181*
Derksen, M	O333
Descamps, D	O332, P095
Dessler, S	P320
Detoc, M	P151
Deulina, M	P199
Di Biagio, A	P048, P099, P105, P230, P234, P243
Di Carlo, P	P173*
Di Cristo, V	P107
Di Filippo, E	P068, P186
Di Giambenedetto, S	O121, P086, P099, P105, P106
Di Matteo, S	P068
Di Perri, G	P141, P158, P163, P213, P291, P304, P305, P308
Di Sora, F	P136
Diallo, M	O122
Diamantis, S	P044
Dias, C	P226, P313
Diaz, H	P369
Dickinson, L	P209, P094*
Dieterich, D	P260*
Digaetano, M	P083
Dimas, D	P184
Dimi, S	P140
Dimitrijevic, B	P188
Dina, J	O332
Ding, W	P259
Diniz, A	P114*
Diogo, L	P215, P226, P313
Doering, M	P249
Doi, T	P312
Dolci, G	P056, P155, P158, P163, P164
Domingo, P	O331, P166
Domínguez, L	P274
Dominguez, L	P284, P285
Dominguez, MR	P329
Domínguez-Domínguez, L	P271*
Dominguez-Dominguez, L	P280
Dominiguez-Dominguez, L	P265
Dominkovic, Z	P324
Dorey, D	P118
Döring, M	P351*
Doroana, M	P308
dos Santos Araujo, AC	P206
Downs, J	P200
Dragovic, G	P188
Dragunova, J	P261
Dravid, A	P195*, P214*
Drimis, S	P360, P361
Dronda, F	P116
Duarte, F	P041
Dubed, M	P369
Dudoit, Y	P023
Duffau, P	O212
Duken, EE	P005*
Dunay, G	P077
Duncan, A	P202, P204, P205
Duppenthaler, A	P001
Duracinsky, M	P236*, P240*, P081, P140
Duro, R	P344
Duvivier, C	P154
**E**	
Eaton, E	P026
Eberhard, N	P142
Eberle, J	P351
Echeverría, P	P180*
Edwards, S	P154
Egesemba, G	P019
Egesimba, G	P113
Egger, M	P177
Eholie, SP	P182
Ehret, R	P358*
Ehrlich, H	P078
El Hamad, I	P290
Elbirt, D	P135*
Elias, PP	P328
Elinav, H	O114
Elliot, E	P094, P307*
Ellman, T	P337
Ello, NF	P182*
Else, L	P094, P307
Emiliozzi, A	P106
Ene, L	P150
Engler, K	P069
Eriksen, J	P031
Ernst, J	O335A
Erscoiu, S	P150
Escribano, P	P294*
Esker, S	P170
Espinoza-Armenta, B	P171
Esposti, LD	P286
Esquivias, J	P193, P196
Esser, S	P049, P104*, P252, P366
Esteva, V	P126
Estrada, V	P166, P280, P284
Etta, E	P057*
Eymard-Duvernay, S	O122
**F**	
Fabbiani, M	O121, P086
Fabbri, G	P053, P315*
Fabrizio, C	P278*, P287
Faetkenheuer, G	P049, P305
Fafi-Kremer, S	O332
Faísca, R	P267
Falasca, F	P160
Falasca, K	P230
Falistocco, C	P357
Fallentin, E	P123
Falvella, FS	P317
Faraut, B	P240
Farfour, E	P140
Faria, D	P007, P042, P267
Farrel, J	P324
Fauci, A	KL1*
Favata, F	P291, P304
Fazio, S	P315
Fedele, V	P216, P346
Fehr, J	P142, P254, P352, P356
Fernandes, C	P211
Fernandes, R	P324
Fernandez, C	P212*
Fernandez, E	P050
Ferrara, M	P090, P304
Ferrara, SM	P092*
Ferraz, RV	P344*
Ferreira, M	P370, P373
Ferrer, A	P052
Ferrero, OL	P128*
Festa, E	P290
Figueiredo, C	P297
Figueroa, MI	P368*
Finn, T	P108
Firlag-Burkacka, E	P020, P220, P247, P300
Fischer, B	P123
Fischer, J	P077
Fischer, S	P038
Fischer, T	P001
Fisher, M	P161
Fitzgerald, D	P200
Flamholc, L	P115
Flandre, P	O332
Fletcher, D	P174, P359
Florence, E	P131, P137
Flores, J	P281
Florez, A	P242
Focà, E	O121, P158, P163, P223, P290
Folguera, C	P102
Fombellida, K	P167
Fontecha-Ortega, M	P103*, P289*
Fontecha-Ortega, M	P203
Force, G	P217*
Fordyce, M	O123, P306
Forleo, MA	P223
Formenti, T	P107
Forni, J	P194
Fortin, C	P134
Fortin, M-C	P134
Fortunak, J	P251
Foschi, A	P279
Fouere, S	P140
Fox, R	P272
Fragkou, P	P040
Francisci, D	O121, P235
Franzetti, M	P107, P234
Fredrick, L	P248
Freeman, J	P256
Freeman, J	P256
Freitas, F	P145
Frésard, A	P151, P225
Friborg, S	O125
Friedrich, G	P351
Frikke-Schmidt, R	P189
Fritz, C	P334
Froguel, E	P044
Fuchs, D	P208
Fuentes, JG	P320
Furrer, H	P177
**G**	
Gagliardini, R	O121*, P086*, P105
Gagneux-Brunon, A	P151*, P225*
Galera, C	P178
Galindo, M	P052, P166
Galindo, MJ	P088
Galindo, M-J	P281
Galizzi, N	P190
Gallant, J	O125
Gallego, MJV	P203
Gallego, MJV	P329
Galli, L	P190
Galli, M	P107, P309, P317
Gallo, A	P315
Gálvez, C	P054
Gama, N	P028*
Gambino, A	P173
Gane, E	P248
Gao, S	P245
Garbuglia, AR	P237
Garcel, A	P078
Garcia, A	P294
Garcia, J	P294
Garcia-Benayas, MT	P280
Garcia-Deltoro, M	P088
García-Deltoro, M	P281
Garcia-Fraile, L	P165
García-Navarro, MJ	P126
García-Vallecillos, C	P082, P084, P088
Gargalianos, P	P162, P354, P360, P361
Gargalianos-Kakolyri, P	P189
Garrait, V	P044
Garrido, N	P270, P295
Garrote, AR	P270, P295
Gasiorowski, J	P120
Gaspar, G	P265, P280, P284, P311
Gaspar-García, E	P126
Gatell, J	O213, P050, P228
Gatell, J	P368
Gatey, C	P035, P044*
Gawron, N	P247
Gaytán-Martínez, J	P171
Geijo, P	P166
Geit, M	P121
Gelpi, M	P345*
Genberg, B	P339
Gentile, M	P237
Geretti, AM	P059, P219
Germano, I	P075
Gerstoft, J	P345
Gervsasoni, C	P309*
Gervsasoni, C	P283, P303
Ghosn, J	P240
Ghout, I	P217
Giacomelli, A	P107, P317*
Giacometti, A	P106
Giacomini, M	P243
Giancarlo, O	P230
Giannini, B	P243
Gianotti, N	P021, P101
Gielman, S	P363
Gil Anguita, C	P193, P196
Gil-Avila, A	P171
Gilbert, H	P339
Gilleece, Y	P231
Gillespie, R	P063
Gil-Martin, A	P265, P280
Gil-Martin, Á	P284
Gil-Martín, Á	P274
Gimeno-García, A	P126
Gimeno-Garcia, A	P280
Gineste, P	P078
Girard, PM	P081
Girard, P-M	P248
Girardi, E	P156
Girshengorn, S	P363
Gisinger, M	P018
Gisslén, M	P115
Giustini, N	P110
Glass, T	P001
Glass, TR	P334
Glaunsinger, T	P130, P133
Glover, C	P272
Goes, AT	P132, P187, P335
Goeser, T	P252
Goff, L	P202, P204, P205
Goffard, J-C	P131, P137, P167
Gogos, C	P162
Golovin, S	P261
Gomez Ayerbe, C	P111
Gómez, C	P103, P289
Gomez-Ayerbe, C	P311
Gómez-Mora, E	P180
Gomez Verdu, JM	P178
Gonçalves, C	P187
Gonçalves, C	P299
Gonçalves, R	P270, P295
González, J	P285
Gonzalez-Baeza, A	P241*
Gonzalez-Cordon, A	O213, P228
González-Domenech, C-M	P054, P372*
González-Domenech, CM	P152
Gonzalez-Garcia, J	P198, P265*, P280*
González-García, J	P274, P284
Goodwin, B	P026, P073, P119*, P233
Göpel, S	P036, P039
Gordhan, B	P028
Gori, A	P086, P136, P234, P250, P263
Gori, C	P053
Gotham, D	P251
Gotuzzo, E	P340
Gotuzzo, L	P274
Goulder, P	P002
Goyvaerts, N	P032
Grabar, S	P240
Gracia, M	P282
Graefe, K	P038
Granados-Reyes, ER	O335A*
Grant-Freemantle, M	P127
Grasa, J	P070
Grawe, C	P001
Grazina, S	P122
Grazina, SD	P029
Gregis, G	P186
Gregoire-Faucher, V	P044
Grennan, T	P016
Grilo, N	P215, P313
Grima, P	O121, P105
Grinsztejn, B	P340
Grisetti, S	P216, P346
Grossi, P	P136
Grosso, C	P089
Grude, M	O332
Grulich, A	O224, P189
Grycner, E	P220
Grzeszczuk, A	P120
Guaraldi, G	P056*, P155, P158*, P163, P164
Gubernick, S	P245*
Guglielmi, G	P173
Guglielminotti, C	P225
Guillemi, S	P016
Guillén, SM	P329
Guimarães, M	P096*
Guimarães, M	P041
Gulick, RM	O322*
Gulminetti, R	P083, P090, P230
Gunter, J	P167*
Günthard, H	P352, P356
Guo, S	P045, P314
Gurjar, R	P308*
Guschina, U	P332
Gustafsson, L	P031
Gutierrez, F	P165
Gutierrez, M	P193, P196
Gutierrez, R	P242
Gutteck, U	P142
Guzmán, MM	P185
**H**	
Haars, UE	P367*
Haas, B	P121
Haas, R	P077
Haberl, A	P036, P039
Haeussinger, D	P049, P077, P367
Häggblom, A	P115*
Hahn, R	P073
Hahn, V	P217
Hakobyan, S	P027, P061, P067, P238
Hallett, T	P156, P157
Hamet, G	P044
Hammarskjold, M-L	P057
Hamzah, L	P218
Hanhoff, N	P038
Hanke, K	P353
Hanna, G	O335A, O335B
Hansen, A	P123
Hansen, B	P229
Harjivan, S	P215
Harris, M	P016
Hartling, HJ	P345
Harvey, R	P189
Hassin, D	P108
Hasson, H	P291
Hatleberg, C	O224
Hatzakis, A	P354, P360, P361
Haubrich, R	P055, P314
Hauser, A	P353*
Heger, E	P049, P249, P252
Heinzkill, M	P055
Hejdeman, B	P115
Heldwein, S	P117, P244
Henegar, C	P260
Henning, L	P142
Heresi, G	P033
Hernández-Quero, J	P084
Hernando, A	P271
Hernando, V	P165
Herrmann, E	P036, P039
Herrmann, M	P133
Herweijer, E	P115
Heuchel, T	P130*, P055
Hidalgo, J	P242*
Hidalgo-Tenorio, C	P193*, P196*, P054, P082, P084, P088
Higgs, C	P208, P307
Hijzen, A	P310
Hill, A	P251*, P256*, P261*, KL2*, O315, P262
Hillenbrand, H	P046, P130
Hirano, A	P312
Hodgson, I	P320
Hoesli, I	P001
Hoffmann, C	O214
Hoffmann, M	P254
Hofmann, A	P353
Hogg, R	P066, P191, P192
Hogue, S	P073
Hohaus, S	P190
Holzendorf, V	P104
Hontañon, V	P265
Hontañon-Antoñana, V	P241
Horban, A	P020, P220, P247, P300, P336
Horta, A	P299
Hortelão, D	P370, P373
Hower, M	P049
Hows, J	P323
Hristea, A	P144
Hsieh, S-M	P201, P255, P257, P268, P275
Hu, J	O336
Hu, Y	P248
Huang, J	P118
Huang, K-C	P259
Huang, Y-C	P275*
Huang, Y-S	P257
Huedo-Medina, T	P071
Huelsenbeck, J	P046*
Huettig, F	P077
Hull, M	P016, P191, P192
Humeniuk, R	P306
Hung, C-C	P266*, P013*, P047, P112, P201, P246, P255, P257*, P268, P275, P365
**I**	
Ianache, I	P150
Iannetta, M	P347, P348*
Ibañez, PP	P085
Ibarra, S	P128
Igene, P	P019
Iklo, C	P182
Imperiale, D	P213
Inberg, Y	P135
Iribarren, J	O331
Irigoyen, C	P282
Izard, S	O122
Izzo, I	P223, P290*
**J**	
Jabbari, S	P016, P191, P192
Jablonowska, E	P120
Jachnowitsch, K	P320
Jacomet, C	P034
Jadav, G	P079*
Jaegel-Guedes, E	P244
Jägel-Guedes, E	P117
Jäger, H	P117, P244
Jakobsen, SF	P323
James, C	P324
Jarilla, F	P372
Jarrin, I	P265, P274, P280, P284
Jaspard, M	P023
Javier, R	P193, P196
Jeantils, V	P044
Jeffreys, G	P256
Jennifer Fusco	P260
Jensen, B	P049, P077
Jensen, B	P366
Jensen, BEO	P367
Jespersen, S	P123
Jessen, H	P055
Jeulin, H	O332
Jevtovic, D	P188
Jilich, D	P189
Jimenez, P	P311
Jiménez-Nacher, I	P285
Jimeno, A	P294
Jipa, R	P144*
Joana, S	P335
Johannesen, H	P123
Johnson, C	O131*
Johnson, M	O215, P035*
Joly, V	P095
Jones, R	P218
Jordao, S	P041
Jose, S	P218*, P239*
Joshi, S	O335A, O335B
Jusdado, JJ	P284
**K**	
Kahlert, C	P001
Kaiser, R	P049, P077, P249, P252, P351, P366
Kakalou, E	P184*
Kakkavas, T	P184
Kalaghatgi, P	P252
Kaleebu, P	P352, P356
Kalkounou, I	P224*
Kalluvya, S	P200
Kambugu, A	P142, P352, P356
Kamerys, J	P120
Kamya, M	P142
Kana, B	P028
Kanagalinghan, U	P012
Kanatschnig, M	P121
Kane, KT	O122
Kangiwa, U	P019
Kaplan, R	O334
Karki, P	P071
Karpov, I	O114
Kartashev, V	P249
Kataraih, J	P200
Katchman, E	P108
Katlama, C	P023, P081
Kato, P	P222
Katsarolis, I	P172, P224
Katsarou, O	P162
Kavanagh, E	P169
Kavatha, D	P040, P172, P227
Kazeka, V	P062
Kearney, B	P306
Keegan, M	P208*
Keitel, V	P252
Kelly, D	P320*
Kennedy, A	P256
Kerr, S	P014, P322
Khaykin, P	P036
Khaykin, P	P039
Khoo, S	P094, P307
Khwairakpam, G	P262
Kiani, G	P027*, P061, P065, P067, P238
Kiertiburanakul, S	P045
Kirangwa, J	P352
Kirk, O	O114, P154
Kirkland, S	P155
Kitas, G	P179
Kitchen, M	P018
Kjær, A	P123
Klimkait, T	P334
Klinker, H	P248
Knecht, G	P038
Knechten, H	P049, P364
Knobel, H	O331
Knoble, N	P138
Knops, E	P049*, P077*, P252, P366
Knox, D	P174, P359
Knysz, B	O114
Kobbe, G	P077
Kocbach, A	P120
Koch, C	P370, P373
Koeppe, S	P046
Kögl, C	P117
Kolawole, G	P339*
Kolb, M	O214
Kolin, J	P113
Kolodziej, J	P015
Konopnicki, D	O222*
Konstantonis, G	P179
Kore, S	P195, P214
Korologou-Linden, R	O315, P261, P262*
Kostaki, EG	P354
Kostaki, E-G	P360, P361
Koteff, J	P210
Kouakou, AG	P182
Koulla-Shiro, S	O122
Kourkounti, S	P224, P354, P360, P361
Kovacs, C	P174, P359
Kovacs, T	P324
Kovari, H	O114, P264
Kowalska, J	P020*, P220, P300*, P327*, P336
Kozirev, OA	P024
Kozyrina, N	P332
Kpamor, Z	P113
Kraft, C	P362
Kravchenko, A	P024*
Kravtchenko, A	P199
Krivine, A	O332
Krystal, M	P022*
Krznaric, I	P060
Ku, S-Y	P091
Kücherer, C	P353
Kulkarni, M	P195, P214
Kulkarni, P	P195
Kumar, K	P028
Kundro, M	P168*
Kusic, J	P188*
Kuznik, A	P143
Kuzovatova, E	O114
**L**	
La Galla, R	P173
Labhardt, ND	P334*
Lachowsky, N	P016
Lackey, P	P260
Lada, M	P354, P360, P361
LaFleur, J	P170, P175
Lagarde, M	O331
Lagarde, M	P271
Laguno, M	O213, P228
Laird, A	P063
Lalanne, C	P140, P236
Lama, J	P368
Lamadrid, JV	P185
LaMarca, A	O124
Lambert, J	P127*
Lambert, J	P169
Lamonica, S	P105
Lamorde, M	P142, P143, P302
Lampreia, F	P075
Lamy, S	P044
Landman, R	P081, P095
Landuyt, EV	P310
Lapa, D	P237
Lapadula, G	P250*, P086
Lapresta, C	P282
Largu, A	P004, P009
Lari, C	P279
Larroze, M	P217
Lartey, M	P350
Lataillade, M	O335A, O335B
Latini, A	O121, P086, P105, P106
Laut, K	P154
Laut, KG	O114*
Lavoie, S	P011
Law, M	O224
Lazanas, M	P354, P360, P361
Lazarini, A	P179
Lazaro, E	O212
Lazarus, JV	O133*
Lazzarin, A	O124, P035, P093, P248
Le Du, D	P081, P217
Le Guillou-Guillemette, H	O332
Le Marec, F	O212
Le Moing, V	O122
Lê, M	P095
Leavitt, R	O334
Lebouché, B	P069
Lecornu, C	P217
Ledergerber, B	P018, P121, P142
Lee, K	P301
Lee, MP	P221
Lee, SS	P221, P319, P374
Lee, Y-C	P013
Leen, C	P342
Leers, M	P229
Léger, D	P240
Leierer, G	P018*, P121
Lejone, TI	P334
Leleux, O	O212
Lemos, A	P226, P313
Lènàrt, A	P069
Lengauer, T	P252, P351, P366
León, M	O335A, O335B
Lepej, SZ	P124
Lepri, AC	P048, P190
Lersch, C	P060
Lesens, O	P034*
Leshno, A	P108, P363
Lessard, D	P069
Leszczyszyn-Pynka, M	P120*
Leval, A	P115
Lhopitallier, L	P341*
Li, Z	P022
Liarte, AG	P087*
Libertone, R	P053, P232, P237, P315
Libois, A	P010*
Lichtner, M	P160, P263, P269, P273, P347, P348, P349
Ligero, C	P050
Limia, C	P369
Lin, K-Y	P047, P255*, P268
Lin, M-H	P091
Lin, WCA	P333
Liña-Amador, MA	P171
Lindbäck, S	P115
Ling, KH	P306
Lino, S	P270, P295
Lisi, L	P105
Liu, C-H	P266
Liu, H	O124
Liu, L	P259
Liu, W-C	P013, P112, P246, P255, P257, P266, P275, P365
Llamoso, C	O335A, O335B*
Lledó, G	P276
Llibre, J	P070
Llibre, JM	P085*
Lo Caputo, S	P048, P278
Lo Casto, A	P173
Lo Re, G	P173
Lo, Y-C	P255
Loff, J	P114
Lojek, E	P247
Lombardi, F	P106
Lonca, M	O213, P228
Longpré, D	P011
Lopes, S	P154*
López-Azkarreta, I	P128
López-Martínez, M	P128
López-Ruz, MA	P084, P193, P196
Lorenzini, P	P021, P053, P101, P216, P232, P250, P346
Losa, J	P265, P280
Losa, JE	P284
Losso, M	O114, P168
Lou, Y-Z	P201
Loutfy, M	P174, P359
Lowe, S	P229
Lozano, A	P054
Lozano, F	P088, P166
Lu, H	O336
Lu, R	O336
Lübke, N	P366*
Lucht, F	P151, P225
Luebke, N	P049, P077, P367
Luethi, A	P254
Luetkemeyer, A	P248
Lüftenegger, D	P130
Lugo-Torres, O	O314
Lui, CYG	P221*
Luís, N	P187
Luís, NP	P335
Lukas, D	P124
Lukoda, N	P331
Lun, W	O336
Lundgren, J	O112*, O114, O224, P154, P189
Luo, Y-Z	P047, P112
Lupi, F	P237, P315
Lupo, A	P107
Lutz, T	P046
**M**	
Macdonald, D	P301
Machado, LY	P369
Maciejewska, K	P154
Macken, A	P127, P169
Madeddu, G	O121, P106, P230*, P235, P263
Madero, JS	P185, P368
Magambo, B	P356
Maganga, E	P200
Maggi, P	P235
Maggiolo, F	P068*, P083*, P093, P186*
Magiorkinis, E	P360
Magnani, G	P156
Magnani, M	P100
Magnè, F	P235
Magnuson, D	O314
Mahajan, U	P214
Mahjoub, N	P217
Mahlab-Guri, K	P135
Maillard, A	O332
Maina, E	P350*
Maio, A	P145
Majerholc, C	P140
Majka, A	P025
Makras, P	P172
Makumbi, M	P113
Malagoli, A	P056, P155, P158, P163, P164
Malaise, J	P134
Malinverni, S	P010
Mallolas, J	O213, P228
Mallon, P	O215, P127
Mallon, P	P169
Malmartel, A	P240
Malmierca, E	P284
Maltez, F	O114, P270, P295
Maltezos, E	P354, P360, P361
Mammone, A	P156
Man, C	P035
Manata, MJ	P270, P295
Manavi, K	P181
Manciuc, C	P004
Manciuc, CD	P009
Mancusi, D	P136*, P141
Manea, E	P144
Manhaeve, C	P057
Manosuthi, W	P035
Mansinho, K	P029, P122, P189, P292, P296
Manzardo, C	P050, P051
Mao, J	P119
Mar Cámara, M	P128
Marcelin, A-G	O332*
Marchetti, G	P048, P100*, P253*, P263
Marcos, MA	P051
Margolis, D	P118
Margot, N	O123
Marigot-Outtandy, D	P217
Marín, I	P178
Marinaro, L	P291, P304
Marini, G	P298
Markowitz, J	P138*
Marocco, R	P160, P269, P273, P348, P349*
Maroto, A A-M	P102
Marques, M	P215
Marques, M	P299
Marques, N	P211*, P267
Márquez, M	P152, P372
Marra, F	P272
Marshall, N	P058, P301
Martel-Laferrière, V	P134
Martin, A	P311
Martin, P	O314
Martín-Carbonero, L	P274, P285*
Martin-Carbonero, L	P198
Martinelli, C	P230, P234
Martinez de Tejada, B	P001
Martinez, E	O213*, P052, P166, P228*
Martinez, L	P294
Martinez, LP	P282*
Martinez, M	P178
Martinez, O	P178, P294
Martinez-Picado, J	P077
Martinez-Rebollar, M	O213, P228
Martini, F	P216, P346
Martins, A	P351
Martins, T	P270, P295
Martins, Y	P075
Mascia, C	P160, P269, P348, P349
Masini, M	P105
Mastrangelo, A	P110
Mastroianni, A	P089*
Mastroianni, C	P250
Mastroianni, CM	P110, P160, P269, P273, P347, P348, P349
Mata-Marín, JA	P171*
Matarranz, M	P271
Mateos, M	P289
Mathez, D	P081
Matin, N	P338
Matlosz, B	P220*
Matus, N	P363
May, M	P239
Mayer, W	P244
Mayorga, M	P054, P372
Mazepa, MM	P222
Mazzarello, G	P243
Mazzotta, F	P021
Mazzotta, V	P190, P216
Mazzotta, V	P232, P346
Mbouyap, P	O122
McCallister, S	O123, P045, P314
McClure, M	O315
McCombe, G	P127
McCormack, S	O311*
McDonagh, T	P022
McFaul, K	P194
McGann, H	P147, P148
McGinness, P	P272
McGinty, T	P169*
McGonigle, K	P057
McGowan, C	P340
McGowan, J	P314
McHutchison, J	P259
McLean, L	P272
McNally, J	P259
McNicholl, I	P314*
McNulty, M	P058
Medeiros, MS	P003*, P206*
Meftah, N	P080
Meireles, P	P017
Mejía, F	P340
Melachrinopoulos, N	P040
Melo, M	P183
Mendez, J	P299
Menendez, AP	P185*
Menendez, MA	P265
Mengoni, F	P269, P349
Menozzi, M	P056, P164*
Menzaghi, B	P230
Mera-Giler, R	O314
Meraviglia, P	P141, P303, P309, P317
Merchant, S	P194
Mercie, P	O212
Merisor, S	P144
Merkley, B	P174, P359
Merlano, C	P243
Merli, M	P291
Merlini, E	P100
Mertens, M	P259*
Merz, L	P197
Mesplede, T	P355
Mesquita, M	P132
Messiaen, P	P318*
Messias, AC	P335
Metallidis, S	P076
Metallidis, S	P361
Metcalfe, R	P272*
Meurer, A	P060
Meuris, C	P167
Meyer, D	P028
Mezzaroma, I	P105
Mezzaroma, I	P110
Mezzaroma, I	P273
Micale, M	P106
Michie, K	P212
Middleton, L	P321*
Mikula, T	P015
Milano, E	P278
Milano, E	P287
Milella, M	P278
Milella, M	P287
Milesi, M	P291
Milesi, M	P304
Millard, J	P231*
Miller, M	O123
Miller, M	P025
Miller, M	P043
Mills, A	O124
Milosevic, C	P063
Mineo, M	P136
Mingo, A	P267
Minguela, A	P178
Minguez, C	P281*
Minisci, D	P303
Minisci, D	P309
Mirand, A	O332
Miranda, A	P292
Miranda, AC	P029
Miranda, AC	P296*
Miranda, AC	P122
Miranda, RF	P185
Miró, J	P050
Miro, J	P051
Mladenovic, N	P320
Mocroft, A	O114, O224, P020, P154, P189
Moguel, JO	P185
Mok, J	P202*, P204*, P205*
Molina, J-m	O313*
Molina, J-M	O334, P044, P305
Moll, A	P055
Mols, P	P010
Molteni, C	P230, P235
Moltó, J	P085
Mohamed-Balghata, MO	P193
Mondi, A	O121
Moneti, V	P292
Monno, L	P278, P287
Monsalvo, M	P289
Montaner, J	O111*, P066, P191, P192
Montaner, JG	P016
Montefiori, M	P243
Monteiro, E	P215, P226, P313
Monteiro, F	P370*, P373*
Montejano, R	P198*
Montemayor, JCG	P329
Montero, M	P052, P165, P166, P281
Montero-Hernández, C	P126
Montes, B	O332, P080
Montes, J	P241
Montes, M	P285
Montes, ML	P198
Montes-Ramírez, ML	P280
Montessori, V	P016
Montrucchio, C	P213, P291, P304
Moore, D	P016
Morand-Joubert, L	O332
Mora-Peris, B	P209
Moreira, HP	P003, P206
Morell, EB	P178*
Morello, J	P226
Morena, V	P158, P163
Moreno, A	P111, P116, P274, P284, P288, P293, P311, P328
Moreno, F	P284, P285
Moreno, J	P282
Moreno, S	P111, P116, P165, P288, P293, P328
Moreno, V	P198
Morgan, H	P301
Moritz, A	P358
Morrison, E	P337
Morton, J	P320
Mosalvo, R	P265
Moschopoulos, C	P040
Mosquera, M	P050, P051
Mossou, C	P182
Mota, M	P132, P267
Moulin, E	P341
Mourez, T	O332
Moutschen, M	P131, P137, P167
Moyle, G	P307
Mueller, C	P049
Mueller, M	P055, P133
Muhairwe, J	P334
Mukelabai, N	P320
Müller, D	P142
Muller, K	P120
Muñoz, A	P178
Muñoz, CG	P316
Muñoz, J	P128
Muñoz-Medina, L	P054
Muñoz-Mendoza, V	P103, P203*, P289
Muñoz-Sanchez, P	P166
Muñoz-Sanz, A	P166
Muriel, A	P311, P328
Murphy, A	P272
Murphy, J	P033
Murray, M	P073*, P125
Murungi, A	P210
Musaazi, J	P142
Mussi, C	P155, P158, P163
Mussini, C	O114, P021, P048, P056, P083, P155, P164, P189, P253, P277
Mutch, C	P342
Mwanza, M	P177
Myers, J	P170*
Myers, J	P175
**N**	
Naeth, G	P364
Naftalin, C	P221
Naggie, S	P259
Nagimova, FI	P024
Naidoo, V	P002
Nakalema, S	P302
Nakauchi, T	P312
Nakijoba, R	P142
Nasta, P	P290
Nava, DAS	P185
Navarra, P	P105
Navas, E	P116, P288
Navér, L	P031
Ndulue, N	P113
Ndung’u, T	P002
Neaga, E	P283
Neau, D	O212
Negredo, E	P180
Nelson, M	P094, P194, P208, P209, P218, P248, P301
Néré, M-L	P217
Neumann-Fraune, M	P049, P249, P252
Neves, I	P041
Ngoc Nieu, PT	P256
Ngom Gueye, NF	O122
Nguyen, B-Y	O334
Nguyen, V-K	P011
Nguyen-Cleary, T	P045
Nguyen-Ngoc, T	P197
Nibot, C	P369
Nicolás, D	P051
Nicolau, C	P004*, P009*
Niculae, C	P144
Nielsen, SD	P123*, P345
Niero, F	P097
Nieto, L	P249
Nijhuis, M	P077
Nikolopoulos, G	P354
Nina, J	P029, P292, P296
Nitpolprasert, C	P014, P322
Nixon, E	P161
Noble, C	P002*
Noe, S	P117, P244
Nogueira, ME	P211
Noori, T	O316*, P324
Norton, M	P368
Nouwen, J	O333
Novikov, N	P025
Nozza, S	P163*, P048, P158
Nunes, S	P145
Nuñez, FD	P329
Nuño, E	P152
Nwabueze, E	P113*
Nwokolo, N	O315*, P012*
Nyman, H	P170
**O**	
Obeid, L	P200
Obermeier, M	P252, P358
Ocejo, DN	P050*
Ochoa, AC	P185
Odolini, S	P290
Oette, M	P049, P366
Ogilvie, G	P016
O’Halloran, J	P127
Oikonomopoulou, M	P360
Oka, S	P045*
Okechukwu, E	P113
Okonkwo, P	P339
Olaru, I	P144
Oldenbuettel, C	P060, P244
Oldenbüttel, C	P117*
Oliva, A	P347
Oliveira, C	P145
Oliveira, V	P183
Olwedo, MA	P331*
Omar, M	P054
O’Neil, S	P245
Oprea, C	P150*
Opsomer, M	P310
Orcamo, P	P243
Orchi, N	P053
Oreni, L	P107, P317
Orkin, C	O124*, O125, P058*, P248*
Orlandi, C	P100
Orlova-Morozova, EA	P024
Orofino, G	O121, P097, P099, P105, P136, P158, P163, P234
Ortega, E	P281
Ortega, M	P369
Ortiz, ZYV	P185
Osinusi, A	P259
Osorio, JLC	P203, P329
Österberg, E	P031
Otelea, D	P283
Owen, A	P308
**P**	
Pacheco, P	P226
Pacheco, P	P313
Padgett, D	P340
Padilla, M	P228
Paduta, D	P189
Pagani, N	P194, P209*, P307
Pagnucco, L	P083, P090
Palacios, R	P054*, P152*, P372
Palacios, Z	P088
Pallier, C	O332
Palmborg, A	P115
Panagopoulos, P	P354, P360, P361
Panchatcharam, N	P245
Pantazis, N	P162*
Paola, B	P230
Paoletti, F	P269
Papadopoulos, A	P040, P172, P227, P354, P360, P361
Papadopoulou, M	P179
Papaioannou, T	P179
Paparizos, V	P162, P224, P354, P360, P361
Papastamopoulos, V	P184, P354, P360, P361
Pape, J	P340
Pâquet, M	P134
Paraninfo, G	P290
Paraschiv, S	P283
Parashar, S	P066
Paraskevis, D	P354*, P360*, P361*
Parczewski, M	O114, P120
Paredes, R	P085
Parera, M	P050
Parisi, S	P176, P298
Parruti, G	P101
Pascucci, R	P117, P244
Pasquau, J	P082, P084, P088, P193, P196
Passavanti, G	P273
Patacca, A	P235
Patel, K	P259
Patey, O	P044
Paton, Y	P064
Patrigeon, SP	P185
Patterson, B	P337*
Patterson, P	P368
Patti, F	P291
Paula, VX	P206
Pauli, R	P055
Paulo, CO	P041*
Paulussen, A	P229
Pauser, I	P252
Pavone, P	P110*, P273*
Payeras, A	O331
Payvandy, N	P210
Peck, R	P200
Pedersen, K	P123
Pedro, L	P007
Peel, J	P016*
Peeters, M	O122
Pellicanò, G	P230
Peltenburg, C	P229*
Pena, ER	P041
Peñafiel, J	O213, P051, P228
Penco, G	P243*, P230
Peng, H	O336
Penteado, SS	P222
Pereira, J	P029, P292, P296
Pereira, K	P029*
Pereira, N	P344
Pereira, S	P215, P226, P313
Peres, S	P029, P122, P292, P296
Perez Elias, M	P111, P288, P293, P328, P329, P311*
Perez, J	P369
Perez, N	P033
Pérez, OR	P185
Pérez-Alvarez, N	P180
Perez-Elias, MJ	P116
Pérez-Valero, I	O331
Perez-Valero, I	P198, P241
Periklis, P	P162
Perno, CF	P346
Perrier, M	P095
Perrone, V	P286*
Perronne, C	P081
Peruzzu, F	P234
Peters, B	P202, P204, P205
Peters, E	P272
Peters, S	P197
Peters, SE	P063, P064*
Petkovic, N	P320
Petrikkos, P	P179
Petrovych, S	P008
Peytavin, G	P095, P217
Pfeifer, N	P351
Phanuphak, N	P014, P322
Phanuphak, P	P014, P322
Philipp, K	P036
Phillips, A	O224, P218
Phillips, RO	P059, P219
Phung, B	P095
Picarelli, C	P086
Pick, N	P191, P192
Piconi, S	P158, P163
Pieraccini, F	P089
Pietraszkiewicz, E	P220, P300
Pilalas, D	P361
Pinedo, Y	P242
Piñeiro, C	P132*, P297
Pinero, C	P344
Pinho, R	P007, P042*
Pinnetti, C	P053*, P216*, P232*, P346*
Piontkowsky, D	P045
Piquero, JMF	P316
Pironti, A	P366
Piroth, L	P236
Pirriatore, V	P213
Piryns, H	P131, P137
Piselli, P	P237
Plana, M	P050, P051
Plazzi, MM	P053, P232
Plomgaard, P	P189
Poças, J	P187, P267, P335
Podzamczer, D	O124, P035, P314
Pokrovskaya, A	P332*
Pokrovskiy, V	P332
Poli, A	P090
Popa, I	P150
Popescu, CI	P283
Popova, A	P199*
Porter, D	O124
Portilla, R	P052
Posokhova, S	P008*
Post, F	O215, P093*, P218, P219
Postel, N	P055, P130, P133*
Póvoas, D	P270, P295
Power, L	P323*, P324
Pozniak, A	P251, P305, P308
Pozzetto, I	P160, P269, P349
Pradier, C	O224
Prajapati, G	P026*, P233*
Prata, M	P267
Pratt, A	P301
Pria, AD	P194*
Prince, M	P301
Prisacariu, L	P009
Proença, P	P267
Protogerou, A	P179
Protopapas, K	P040*, P172, P227
Psichogiou, M	P162, P179*, P354, P360, P361
Puchhammer, E	P362
Puig, J	P180
Pulido, F	O331, P316*, P271, P285
Puoti, M	P021, P250
Pupko, T	P363
Puskas, C	P066
Puzzolante, C	P277
Pyziak-Kowalska, K	P300, P336*
**Q**	
Quercia, R	P210*
Quereda, C	P111, P116, P265, P288, P293
Quirino, T	P141, P234
Quirk, E	O124
Qurishi, N	P252
**R**	
Raben, D	O114, P323, P324
Radice, A	P279
Radoi, R	O114, P150, P189
Raffi, F	O125*, P069, P305, P308
Raha, D	P342
Ram, R	O123, P043
Ramgopal, M	O124
Rami, A	P236
Ramírez, BC	P185
Ramirez, F	P270, P295
Rana, A	P076
Ranieri, R	P279*
Ranin, J	P188
Rapaport, S	P108
Rappold, M	P121*, P018
Raskevich, Y	P320
Rassool, M	O334
Ratanasuwan, W	O334
Ratcliffe, L	P212
Rathod, N	P195, P214
Rauch, A	O221*, P001, P254, P264
Rausch, M	P133
Ravaglia, G	P089
Rawlings, K	O314*
Rawlins, S	O334
Raycraft, T	P027, P238*, P061, P065, P067
Raymond, S	O332
Razavi, H	P167
Reader, J	P028
Rebeiro, P	P340
Regea, D	P005
Reiss, P	O224
Rekosh, D	P057
Retsas, T	P224
Reus, S	P281
Reverte, C	P328
Reynes, J	P080*, O122, P093
Rhee, M	O125, P093
Ribeiro, EA	P003
Ribera, E	O331
Riccardi, N	P097
Ricci, E	P230, P234, P235
Richert, L	P305, P308
Rico, M	P285
Ricottini, M	P232
Riddler, S	P302
Ridolfo, AL	P107
Rieger, A	P121
Rieke, A	P055
Rijnders, B	O124, O333*
Ringera, I	P334
Rios, MJ	P088
Ripamonti, D	P083
Ristola, M	P189
Ritchings, C	P175*
Ritter, A	P117
Riva, A	P090*, P158, P163, P303
Rivero, A	O331
Rivero, B	P369
Rivero, M	P165
Rizzardini, G	P099, P248, P303
Rizzi, M	P068
Roberts, G	P118
Roberts, J	P210
Roca, B	P109*, P166
Rocha, C	P370, P373
Rocha, M	P017*
Rocha, S	P211
Rockstroh, J	O124, P248
Rockwood, K	P155
Rodallec, A	O332
Rodgers, A	O334
Rodrigues, C	P075*
Rodriguez, A	O213, P228
Rodriguez, F	O331
Rodriguez, G	P033
Rodriguez, L	P242
Rodríguez, MA	P103
Rodriguez, MA	P116
Rodríguez-Sagrado, MA	P289
Rodriguez-Sagrado, MA	P311
Roe, J	P194
Roemer, K	P038*
Roemer, K	P252
Rojano, R	P294
Rojas, E	O334
Rojas, J	O213, P228
Rojo, P	O113*
Rokx, C	O333
Roldan, EQ	O121, P090
Rolon, MJ	P368
Romaine, J	P125
Romay, D	P369
Romero, A	P311
Romito, A	P213
Ron, R	P285
Ronat, V	P151
Rosa, AC	P211
Rosenberg, T	P184
Rosenblatt, L	P170, P175
Rossetti, B	P105*
Rossi, R	P160, P348, P349
Rougemont, M	P264
Rouleau, D	P134
Roura, S	P180
Roussel, C	O332
Routier, C	P044
Rouzier, V	P340
Roxo, F	P267
Rozenbaum, W	P044
Rozera, G	P053
Ruane, P	O124
Ruas, R	P297
Rubin, J	P031
Rubio, R	P271, P285
Rubio, T	P282
Rudin, C	P001
Ruggiero, A	P059, P219
Ruiz, E	P294
Ruiz, N	P369
Ruiz-Giardin, J	P280
Ruiz-Morales, J	P152
Rump, J-A	P364
Rusconi, S	P107*, O121, P048, P090, P097, P101, P105, P106, P156, P230, P317
Rutkowski, J	P327
Ryan, P	O331, P284
Ryom, L	O224
Ryu, J	P045
**S**	
Sá, J	P187
Saag, M	P026
Sabin, C	O215, O224, P169, P218, P239
Sabranski, M	O214*
Sacchi, P	P371
Sako, R	P312
Sala-Piñol, F	P070*
Salazar-Vizcaya, L	P254
Saleh, A	P200*
Salemovic, D	P188
Sallie, R	P256
Salomón, H	P357
Salters, K	P191*, P192*, P066
Salvaggio, SE	P317
Samani, A	P194
Sambatakou, H	P154, P162, P354, P360, P361
Samji, H	P066
Samperiz, G	P088, P282
Sanchez, G	O213
Sanchez-Brown, E	P129*
Sánchez-Conde, M	P103
Sanchez-Conde, M	P111, P116, P288, P293
Sanchez-Palomino, S	P050
Sánchez-Palomino, S	P051
Sandler, I	P174, P359
Sandoval, CO	P185
Sandrini, S	P223
Sangiorgi, D	P286
Sanjoaquin, I	P282
Santantonio, TA	P092
Santoro, A	P056, P164
Santoro, CR	P235
Santoro, M	P250
Santos, AS	P297
Santos, C	P007, P042
Santos, C	P007
Santos, C	P096
Santos, C	P328
Santos, I	P265, P274, P284
Santos, J	O331, P054, P152, P372
Santos, JR	P085
Santos, LP	P369*
Sanz, J	O331, P284
Sanz, J	P280
Sanz, JS	P087
Saracino, A	P278, P287
Saraf, C	P195, P214
Sarcletti, M	P018, P121
Sarfo, FS	P059, P219
Sarmento, A	P297, P344, P370, P373
Sarmento e Castro, R	P299
Sasset, L	P097
Sauvage, M	P217
Savage, A	P256
Savinelli, S	P269, P348
Sawadogo, A	O122
Sax, P	O334, P175
Sayers, I	P301
Scabini, S	P213
Scarsi, K	P302
Schembri, G	P093
Scheri, GC	P110
Scherrer, A	P142, P352, P356
Scherrer, D	P078*
Schiaroli, E	P235
Schietroma, I	P110
Schippers, J	P229
Schlaefper, E	P078
Schleenvoigt, B	P055*
Schmid, P	P264
Schmidbauer, C	P362
Schmied, B	P121
Schofield, J	P272
Scholten, S	P049, P130, P133, P252
Schoolmeesters, A	P307
Schuebel, N	P049
Schuelter, E	P049
Schuetze, M	P358
Schulman, K	P260
Schülter, E	P252, P366
Schulz, C	P055, P130
Schurink, K	O333
Schüttfort, G	P036*, P039
Schweitzer, I	P363
Schwimmer, C	P308
Scott, D	P338
Seabra, J	P299
Sedegah, M	P350
Seixas, D	P270, P295
Sekaggya, C	P142*, P352, P356
Selby, P	P301
Sellier, P	P044
SenGupta, D	O124, P306
Sequeira, C	P215
Sequeira, F	P122*, P132
Serafino, S	P110
Serra, N	P173
Serrano, R	P280, P311
Serrão, R	P132, P297*, P344, P370, P373
Setti, M	P243
Sfikakis, P	P179
Shahi, R	P027, P061, P065, P067*, P238
Shanyinde, M	P263*
Shanyinde, M	P253
Shao, Y	O336
Sharma, M	P174, P359
Shasha, D	P108
Sheehan, G	P127, P169
Sheng, W-H	P201, P255, P257, P268, P275,
Shepherd, B	P340
Shepherd, L	P189, O114, O224*, P020
Shevchenko, S	P008
Shieh, H-T	P091
Shimonova, TE	P024
Shipulina, O	P199
Shirasaka, T	P312
Shoveller, J	P191, P192
Shrestha, R	P071*, P139*
Shulman, N	P248
Siakalis, G	P172*
Siakalis, G	P040, P227
Sias, C	P237
Sica, S	P092
Sierra, S	P249*, P252*
Sierra-Aragon, S	P049
Sierra-Madero, J	P340
Siewaszewicz, E	P327
Sighinolfi, L	P101
Signori-Schmuck, A	O332
Silebi, V	P098*
Silva, E	P369
Silva-Pinto, A	P297
Silverberg, M	P189
Simão, M	P007
Simões, P	P270, P295
Simon, A	P023
Singh, A	P027, P061, P065*, P066*, P067, P238
Sipsas, N	P162, P354, P360, P361
Siyum, O	P005
Skoura, L	P076, P361
Skoutelis, A	P162, P184, P354, P360, P361
Skwara, P	P120
Slama, L	O125
Slim, J	P314
Sloan, L	O335A
Smart, L	P200
Smiatacz, T	P189
Smit, M	P157*
Smit, S	P156*
Smith, C	P058, P059, P219
Smith, D	P191, P192
Smith, G	P174, P359
Smith, K	P035
Soares, F	P183
Soares, J	P297
Soares, MC	P222
Soars, M	P022
Sobiesczcyk, M	P337
Soeiro, C	P299*
Soeria-Atmadja, S	P031*
Soldatovic, I	P188
Solingen, RV	P032
Son, O	P044
Soria, A	P250
Soto, K	P226, P313
Soto, Y	P369
Soto-Malave, R	P248
Soto-Ramirez, LE	P129
Souza, RS	P206
Spagnuolo, V	P090
Spano, J-P	O223*
Spanos, A	P040
Speck, R	P078
Speer, M	P038
Sperle, I	P324*
Spinetti, A	P290
Spinner, C	P055, P060*, P117, P244*
Spire, B	O317*, P069
Squillace, N	P230, P234, P235
Squires, K	O334
Ssemwanga, D	P352, P356
Stancil, B	P118
Staub, T	O114
Steens, J-M	P078
Steib-Bauert, M	P038
Steinberg, S	P174, P359*
Stellbrink, H-J	O214, P055
Stempkowska, J	P120
Stephan, C	P036, P039, P046, P055, P130, P154
Sterrantino, G	P086, P097, P099, P105, P106, P141
Sthoeger, Z	P135
Stock, D	O335A, O335B
Stockdale, A	P059, P219
Stoeckle, M	O124, P254, P264
Stoehr, A	P130, P133
Stohr, W	P305
Su, L-H	P266
Su, Y-C	P246, P257, P365
Suarez-Lozano, I	P166
Sued, O	P368
Sulkowski, M	P259
Sullivan, PS	O132*
Sun, H-Y	P013, P047, P112, P201, P255, P257, P266, P268, P275, P365
Sun, N-L	P091
Sun, Y	O336
Sun, Y	P022
Supparatpinyo, K	P045
Sutcliffe, L	P258*
Suter-Riniker, F	P254
Sutter, A	P264
Suvorova, Z	P332
Svedhem, V	P115
Swabe, J	P301
Swaden, L	P058, P301
Sweet, D	P072
Sypsa, V	P354
Szymanska, B	P247*
Szymczak, A	P120
**T**	
Taboada, JR	P193, P196
Takahashi, M	P312
Tamhane, A	P026
Tardei, G	P150
Tartaglia, A	P092
Tashima, K	P314
Tau, L	P108*, P363
Tavares, AP	P299
Tavares, G	P370, P373
Taveira, N	P351
Taylor, N	P121
Tazi, J	P078
Tebas, P	P170
Tebit, D	P057
Teira, R	P052*, P166*
Teixeira, GFD	P003
Teixeira, T	P132
Tejo, S	P211
Téllez, M	O331
Tellez, M	P265
Tempestilli, M	P315
Teofili, L	P190
Teppler, H	O334
Termini, R	P141*, P136
Terron, JA	P088
Thalme, A	O124
Theou, O	P155
Thiagarajah, S	P118*
Thilakarathne, P	P131, P137
Thomas, R	P011*
Thompson, M	P314
Thomson, D	P063
Thomson, E	P258, P272
Thomson-Glover, R	P212
Thorsteinsson, K	P345
Tieghi, T	P269, P273, P349
Tilley, D	P174, P359
Timelli, T	P315
Timm, J	P252
Timsit, J	P140
Tinago, W	P127
Tincati, C	P100
Tiri, B	P235
Togami, H	P312
Toibaro, J	P168
Tomazic, J	P189
Tomishima, K	P312
Torkington, A	P301*
Tornero, C	P088
Torres, A	P178
Torres, B	O213, P228
Torres, R	P274, P285
Tosato, C	P160
Tossa, HG	P011
Tosti, A	P105
Touloumi, G	P162
Toupin, I	P069*
Trabaud, M-A	O332
Tran, JN	P119
Tran-Muchowski, C	P093
Trentalange, A	P304
Trêpa, J	P211
Trevelion, R	P218
Treviño, A	P276
Trezzi, M	P105
Tricas, A	O213, P228
Trigo, D	P226*, P313
Trinh, R	P248
Trofimora, T	O114
Troisvallets, D	P217
Trøseid, M	P123
Trottier, H	P011
Troya, J	P274
Trujillo, J	P294
Trunfio, M	P213*
Tsachouridou, O	P076*
Tsai, M-S	P047*, P365
Tsiang, M	P025
Tsilika, M	P227*
Tsiodras, S	P040
Tubiana, R	P023
Turner, D	P363*, P108
Turner, L	P044
Turpault, I	P044
Turriziani, O	P160
**U**	
Uberti-Foppa, C	P291
Uehira, T	P312
Ullum, H	P345
Underwood, J	O215
Ungureanu, E	P150
Uranga, A	P328
Urbinati, L	P290
Uribe, JG	P185
Usadel, S	P038
Usó, J	P281
Ustianowski, A	P301
Uvera, SR	P185
Uyanik, D	P320
**V**	
Vaghjiani, T	P301
Vai, D	P213
Valadas, E	P183
Valantin, M-A	P023
Valdés, N	P369
Valencia, E	P198
Valente, C	P267*
Valenti, D	P068, P083, P186
Valero, S	P178
Valet, S	O332
Valgardsson, S	P115
Valsecchi, L	P230
van den Bosch, B	P229
van den Bulcke, C	P167
Van der Cam, B	O124
Van der Ende, I	O333
van der Hilst, J	P318
Van Gorp, E	O333
Van Kampen, J	O333
van Sighem, A	P018
Van Wijngaerden, E	P131, P137
Vandekerckhove, L	P131, P137, P305
Vandenhende M-A	O212
Vandercam, B	P131, P137
Vanveggel, S	P032, P310
Varriano, B	P174, P359
Vasconcelos, O	P299
Vata, A	P004
Vavassori, A	P290
Vavro, C	P035
Vaz, C	P370, P373
Vaz Pinto, I	P096, P267
Vecchiet, J	P263
Vegas, A	P280
Velez, J	P145*
Veloci, S	P106
Veloso, V	P340
Vendemiati, G	P291
Ventura, F	P029, P292, P296
Venturini, A	P243
Vera, F	P178, P294
Vera, J	O215, P161
Verbon, A	O333, P229
Verdejo, G	P088
Vergas, J	P274
Verma, S	P231
Viale, P	P101
Viani, R	P248
Viard, J-P	P189, P240
Vichi, F	P230, P234, P235
Viciana, I	P372
Viciana, P	P165, P166
Vignale, F	P105
Villa, G	P059*, P219*
Villar, SS	P116
Viloria, G	P168
Vincent, R	P147
Vincenzo, V	P160
Vinikoor, M	P177
Vinuesa-García, D	P084
Viscoli, C	P243
Vita, S	P160*, P269, P348
Vivancos, MJ	P103, P280, P311
Vivancos, MJ	P328
Vivancos-Gallego, M	P111*
Vivancos-Gallego, MJ	P116*, P288*, P293*
Vlaicu, O	P283
von Braun, A	P352*, P356*, P142
von Hahn, T	P252
von Krosigk, A	P117, P244
Von Lingen, A-I	P324
Vostokova, NV	P024
Vullo, V	P110, P269, P273, P347, P348
**W**	
Wagner-Cardoso, S	P340
Wainberg, M	P355*
Waizmann, M	P055
Walker, A	P252
Walli, RK	P130, P133
Wandeler, G	P177, P254
Wang, H	O336
Wang, H-Y	P207
Wang, L	P066
Wang, M	O336
Wang, M	O336
Wang, X	O315
Wardhana, A	P262
Ware, N	P339
Wasserman, A	P108
Watanabe, D	P312
Waters, L	P338*, P209
Weber, J	P189
Weiser-Smeke, AE	P171
Weiss, L	P236
Welz, T	O214
Wensel, D	P022
Wensing, A	P077
West, B	P320, P324
West, S	P306
Westermayer, B	P130, P133
White, J	P119
White, K	P025*, P043
Whitlock, G	O315, P012, P338
Wiercinska-Drapalo, A	P015*
Wiese, C	P060, P117, P244
Wiesmann, F	P364*
Wieszner, C	P055
Wigger, A	P130
Wijting, I	O333
Williams, D	P218
Williams, I	O215
Wiltshire, CS	P143*
Winston, A	O215, P208, P209
Wiseman, S	P192
Witor, A	P120
Wójcik, G	P327
Wójcik-Cichy, K	P120
Wolf, E	O214, P046, P060, P117, P153*, P159, P244
Wolf, T	P036, P039
Wong, CKB	P319*, P333*, P374*
Wong, J	P016
Wong, KH	P333
Wong, NS	P221
Wong, NS	P374
Wright, J	P161
Wright, M	P301
Wu, A	P138
Wu, C-H	P013, P246, P266
Wu, H	O336
Wu, P-Y	P201*, P047, P112, P255, P266, P275
Wyen, C	O214, P133
Wyles, D	P248, P259
Wyndham-Thomas, C	P167
Wynne, B	P035
**X**	
Xia, W	O336
Xie, D	O336*
Xing, H	O336
Xu, X	O334
Xylomenos, G	P354, P360, P361
**Y**	
Yagura, H	P312*
Yakovlev, A	O335B
Yamazaki, K	P312
Yan, M	O125
Yan, M	P093
Yang, C-J	P207*
Yang, C-J	P246*
Yang, C-J	P047
Yang, C-J	P365
Yang, S-P	P266
Yang, S-P	P047, P112, P201, P275
Yao, C	O336
Yazdanpanah, Y	P093, P095, P308
Ye, M	P191, P192
Yllescas, M	O331
Yoshino, M	P312
Youssef, E	P161*
Yu, J	O336
Yurin, O	P332
Yust, I	P108, P189
**Z**	
Zaccarelli, M	P086, P101*, P232, P253, P315
Zagalo, A	P343
Zakowicz, A	P324
Zaltron, S	P290
Zamora, AMM	P329
Zamudio, FB	P185
Zangerle, R	O114, P018, P121
Zari, A	P306
Zavitsanou, A	P360, P361
Zekan, Š	P124*
Zeldis, I	P363
Zelt, S	P260
Zempekakis, P	P076
Zhang, D	P233
Zhang, J-Y	P047, P112, P201, P266
Zhang, S	P022
Zhang, T	O336
Zhang, W	P016
Zhao, M	O336
Zhao, Q	O336
Zheng, Q	O336
Zheng, Y	O336
Zhu, B	O336
Zhyvytsia, D	P062*
Zilmer, K	O114
Zimmer, M	P301
Zimmermann, S	P197
Zingaropoli, MA	P347*, P348
Zink, A	P060
Ziv-Baran, T	P108
Zona, S	P056, P155
Zoufaly, A	P362*
Zoungrana, J	O122
Zouzia, E	P184
Zozulya, OV	P024
Zubero, Z	P128
Zuccalà, P	P160, P269*, P348, P349
Zuccaro, V	P371
Zucker, J	P337
Zucman, D	P140*
Zuin, S	P298
Zyambo, Z	P177
